# Design of an Organocatalytic Asymmetric (4 + 3) Cycloaddition of 2-Indolylalcohols with Dienolsilanes

**DOI:** 10.1021/jacs.2c02216

**Published:** 2022-05-06

**Authors:** Jie Ouyang, Rajat Maji, Markus Leutzsch, Benjamin Mitschke, Benjamin List

**Affiliations:** † Max-Planck-Institut für Kohlenforschung, D45470 Mülheim an der Ruhr, Germany; ‡ Institute for Chemical Reaction Design and Discovery (WPI-ICRedd), Hokkaido University, Sapporo 001-0021, Japan

## Abstract

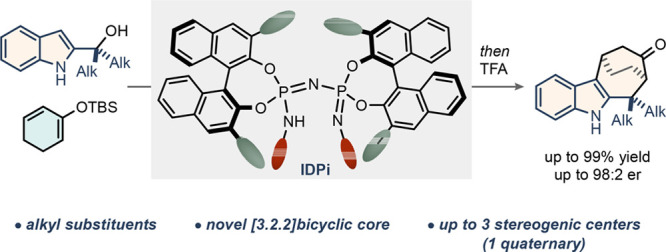

Here we present the design of a highly enantioselective, catalytic (4 + 3) cycloaddition of *gem*-dialkyl 2-indolyl alcohols and dienolsilanes, enabled by strong and confined IDPi Lewis acids. The method furnishes novel bicyclo[3.2.2]cyclohepta[*b*]indoles with up to three stereogenic centers, one of which is quaternary. A broad substrate scope is accompanied by versatile downstream chemical modifications. Density functional theory-supported mechanistic studies shed light on the importance of the in situ generated silylium species in an overall concerted yet asynchronous cycloaddition.

Asymmetric cycloadditions are prominently sought-after disconnection strategies and immensely valuable for the rapid construction of stereochemical complexity while retaining a rational sense of modularity.^1^ Yet, to date, in contrast to well-developed asymmetric (4 + 2) and (3 + 2) cycloadditions, asymmetric (4 + 3) cycloadditions are underdeveloped.^2^ While a few pioneering achievements have been recorded, highly active furans have invariably been used to react with preactivated oxyallyl precursors, thus limiting the generality and diversity of these methods.^3^ Therefore, expanding the structural diversity of catalytic asymmetric (4 + 3) cycloadditions appears to be a worthwhile endeavor. In this context, highly reactive dearomatized indole frameworks display a privileged role in nature as well as in chemical synthesis (Scheme 1A). For example, Martin and Rawal et al. found that 2-methide-2*H*-indoles rapidly undergo efficient (4 + 3) cycloadditions with electron-rich dienolsilanes to furnish racemic cyclohepta[*b*]indoles, which were further applied to the nonasymmetric synthesis of natural products actinophyllic acid and ambiguine P.^4^

**Scheme 1:**
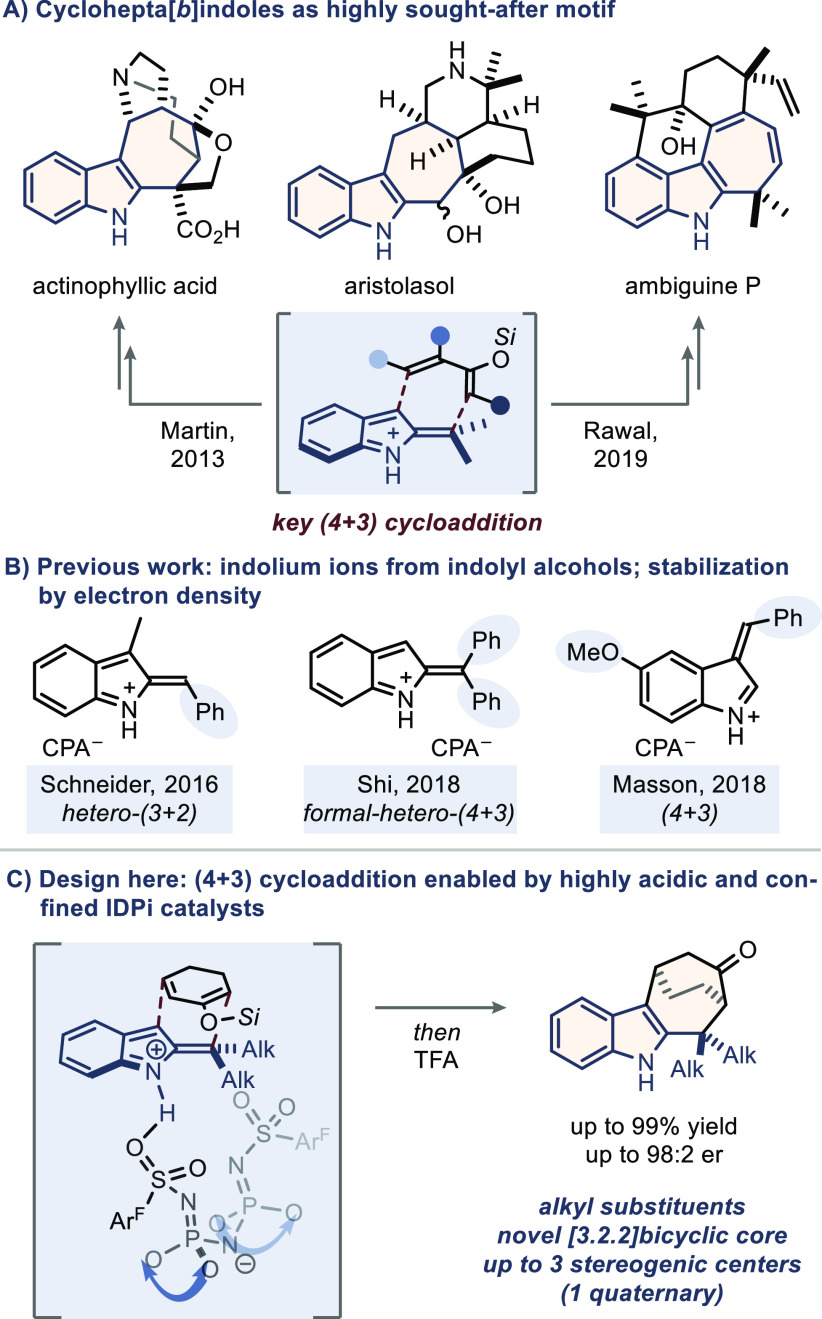
(A) The Privileged Cyclohepta[*b*]indole Motif in Several Natural Products; (B) Previously Applied Dearomatized Indole Frameworks in Asymmetric Organocatalysis; (C) This Work: Newly Designed (4 + 3) Cycloaddition of 2-Indolyl Alcohols with Dienolsilanes

The high demand for a catalytic asymmetric solution to this problem has been recognized.^5^ Due to the high-energy nature of the reactive intermediate, in situ generation of the critical cationic species is generally required, and acid catalysis offers a broad spectrum of opportunities in combination with the respective 2- or 3-indolyl alcohols (Scheme 1B). Schneider and co-workers subjected 2-indolyl alcohols to the action of chiral phosphoric acid catalysts in hetero-(3 + 2) cycloadditions with 2-vinylindoles,^6^ and, more recently, Shi et al. applied a similar concept to a formal hetero-(4 + 3) cycloaddition with in situ generated *ortho*-quinone methides.^7^ Additionally, Masson and co-workers demonstrated viable (4 + 3) cycloadditions of 3-methide-3*H*-indoles with dienamines.^3f^ Notably, the common ground for all of these reactions is the necessity of electron-donating substituents for two reasons: (1) stabilization of the electron-poor reactive intermediate and (2) prevention of any side reactions because of the high basicity of the chiral phosphate anion. For this reason, a general approach to this challenge still remains elusive.

We reasoned that, by virtue of a stronger acid, the higher energetic barrier toward the generation of more diversely substituted intermediates from the corresponding indolyl alcohol might be overcome. Encouraged by our recent studies on silylium-based asymmetric counteranion-directed catalysis (Si-ACDC),^8^ an IDPi catalyst would generate a strong Lewis acid in the presence of a dienolsilane, followed by the generation of the protonated 2-methide-2*H*-indole and subsequent (4 + 3) cycloaddition to deliver bicyclo[3.2.2]cyclohepta[*b*]indoles. The IDPi anion’s inherent confined microenvironment is expected to benefit stereoinduction as well as chemoselectivity. Herein, we report on the realization of this newly designed cycloaddition with high enantioselectivity (Scheme 1C).

We initiated our studies by reacting indolyl alcohol **1a** with dienolsilane **2a** in the presence of Tf_2_NH (p*K*_a_ = 0.3 in acetonitrile) at 25 °C, which cleanly provided product **3a**. In contrast, weak acids such as chiral phosphoric acid (CPA) **6a** (p*K*_a_ = 13.6 in acetonitrile) and *N*-(perfluoronaphthalen-2-yl)sulfonyl-phosphoramide **6b** did not yield any product, most likely due to the formation of a covalently silylated CPA species displaying insufficient Lewis acidity for turnover. Remarkably, however, in the absence of dienolsilane **2a**, CPA **6a** rapidly furnished dimer **5** within minutes (Table 1, entry 3). This is mainly attributed to the rapid formation of a highly nucleophilic 2-vinyl-1*H*-indole, which would directly capture the reactive intermediate in a dimerization event already under weakly Brønsted acidic conditions.^9^ Using IDPi **6c** and **6d** as catalysts at 25 °C, the desired product is indeed formed, along with dimer **5**. IDPi **6e** with a modified 3-biphenyl substitution pattern in the BINOL backbone affords a clean reaction profile but with poor enantioselectivity. Lowering the temperature to −50 °C could also suppress side product formation and furnished the desired product with excellent yield and promising enantioselectivity of 66:34 (entry 8). Because of the confined active site of the IDPi catalyst, fine-tuning of the structural parameters was necessary in order to further improve the enantioselectivity. Extensive screenings effectively showed 2-tetrahydronaphthalenyl-substitution on the 3,3′-positions of the BINOL backbone as a privileged motif (IDPi **6f**, entry 9). A modification of the inner sulfonamide core from trifluoromethyl to perfluoronaphthalen-2-yl enhanced the enantiomeric ratio from 86:14 to 95.5:4.5, maintaining the excellent yield and **3a**:**5** ratio (entry 12).

**Table 1:**
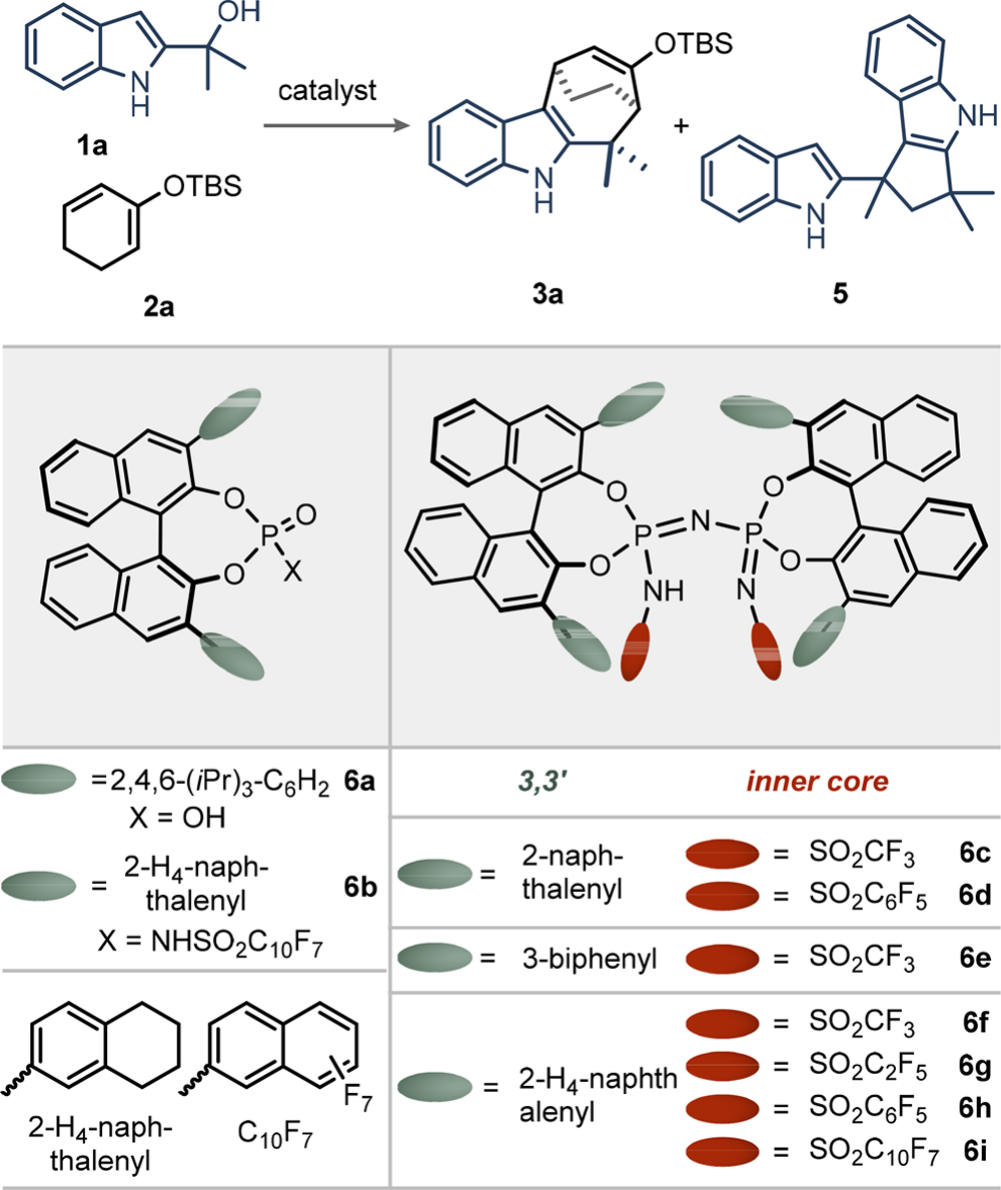
Reaction Development^a^

entry	catalyst	*T* (°C)	**3a** yield (%)	**3a**:**5**	**4a** er^b^
1	Tf_2_NH	25	90	>25:1	–
2	**6a**	25	NR	–	–
3^c^	**6a**	25	–	<1:100	–
4	**6b**	25	NR	–	–
5	**6c**	25	87	20:1	56:44
6	**6d**	25	42	2:1	64:36
7	**6e**	25	87	20:1	55:45
8	**6c**	–50	98	>25:1	66:34
9	**6f**	–50	99	>25:1	86:14
10	**6g**	–50	98	>25:1	90:10
11	**6h**	–50	98	>25:1	94:6
12	**6i**	–50	98	>25:1	95.5:4.5

a Reactions were performed with substrate **1a** (0.01 mmol), catalyst (2.5 mol %), **2a** (4.0 equiv) in CH_2_Cl_2_ (0.4 mL); yield of indole **3a** and the ratio of **3a**:**5** was determined by ^1^H NMR analysis with 1,3,5-trimethoxybenzene as an internal standard.

b After the deprotection by trifluoroacetic acid (10 μL), enantiomeric ratios (er) of ketone **4a** were measured by HPLC.

c Without **2a**. NR, no reaction.

Having identified the optimal catalyst and reaction conditions, we were keen to explore differently substituted 2-indolyl alcohols (Scheme 2A). First, we evaluated systematic methyl substitution patterns on the indole backbone at the 4-, 5-, 6-, and 7-position (**1b**–**1e**), as well as tetrahydrocyclopenta[*g*]indole **1f** and benzo[*g*]indole **1g**. Gratifyingly, these substrates are well tolerated under our optimal reaction conditions. Employing IDPis **6h** or **6i**, we could obtain products **4b**–**4g** with excellent yields and enantioselectivities. Additionally, electronic modification of the aromatic system via halogenation (**1h** to **1j**) and introduction of a methoxy group (**1k**) enable highly enantioselective access to products **4h**–**4k** as well. Having established a reactivity platform for the transformation of *gem*-dialkyl-substituted 2-indolyl alcohols, the possibility of introducing two nonidentical alkyl substituents arises, generating an additional quaternary stereogenic center. We were delighted that substrates **1l**–**1p** readily engaged in this (4 + 3) cycloaddition to give **4l**–**4p** with good diastereoselectivities and excellent yields as well as enantioselectivities. Notably, a terminal alkene and TMS-alkyne (**4n**, **4p**) were also well tolerated, providing opportunities for further elaboration of the products. Substrate **1q**, as representative of an aryl-substituted 2-indolyl alcohol, was converted with moderate yield and enantioselectivity of 85:15 er.

**Scheme 2:**
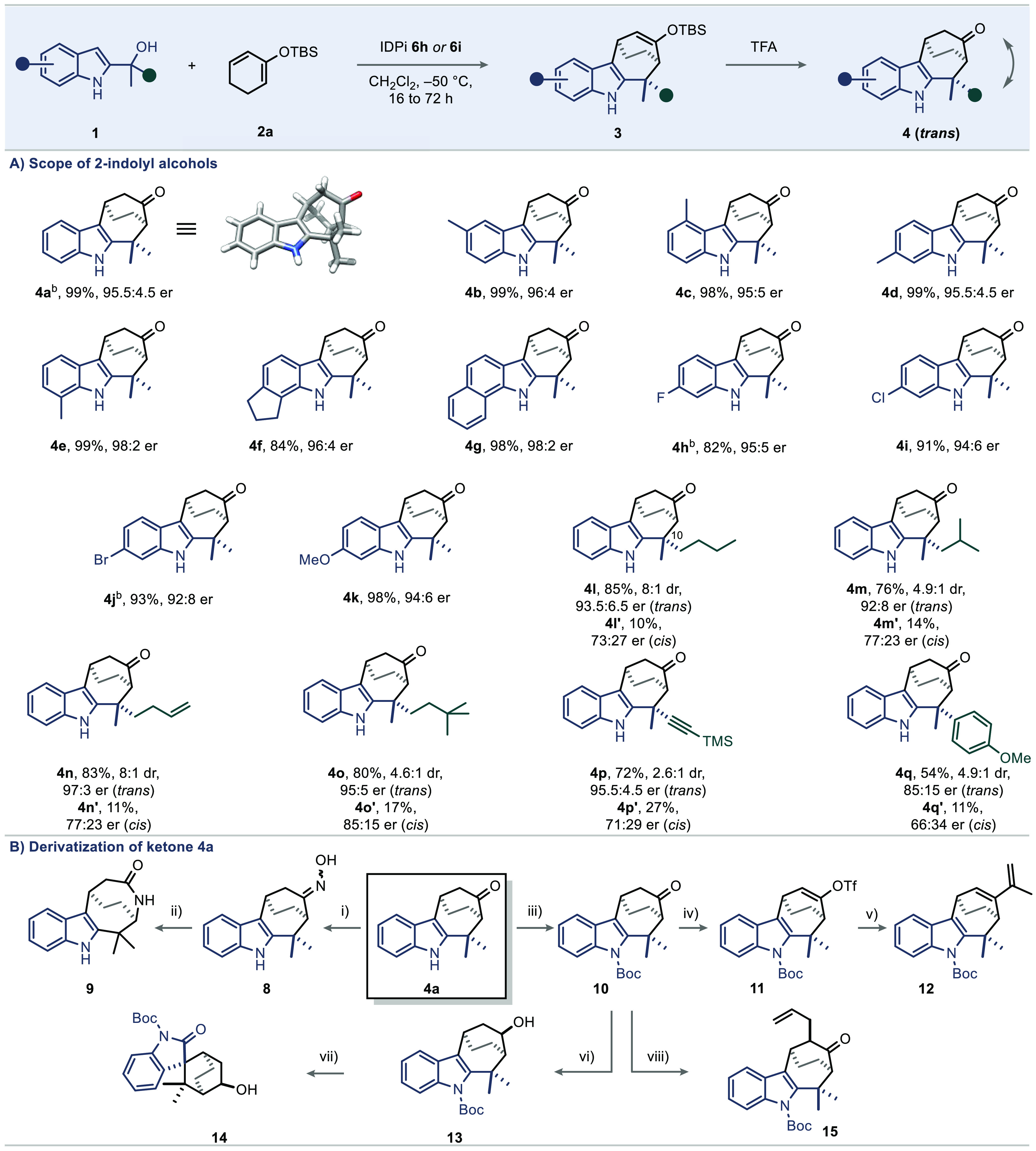
(A) Scope; (B) Derivatizations of Product **4a** Reactions were carried out with 0.08–0.10 mmol of substrate **1**, IDPi **6h** (2.0 to 2.5 mol %), diene **2a** (4.0 equiv) in CH_2_Cl_2_ (0.05 M) at −50 °C unless noted otherwise. IDPi **6i** was used. Enantiomeric ratios (er) were measured by HPLC or GC and unless otherwise indicated, all diastereomeric ratios (at C10) of products **4l**–**4q** were measured by GC. (B) Reagents and conditions: (i) **4a** (1.0 equiv, 95.5:4.5 er), Hydroxylamine hydrochloride (10 equiv), 25 °C, pyridine/MeOH (1:1, 0.4 mL), 84%. (ii) **8** (1.0 equiv), TsCl (1.0 equiv), triethylamine (2.0 equiv), 0 to 25 °C, CH_2_Cl_2_, 53%, 95:5 er. (iii) **4a** (1.0 equiv), DMAP (1.5 equiv), Boc_2_O (12.0 equiv), toluene (0.1 mL), 95 °C, 78%. (iv) **10** (1.0 equiv), LiHMDS (10.0 equiv), Comins’ reagent (1.7 equiv), THF, −78 to 25 °C, 93%. (v) **11** (1.0 equiv), isopropenylboronic acid pinacol ester (4.0 equiv), Pd(PPh_3_)_4_ (10 mol %), K_2_CO_3_ (2.0 M, 80.0 equiv), 1,4-dioxane, 80 °C, 90%, 96:4 er. (vi) NaBH_4_, MeOH, 0 °C, 95%, dr >20:1. (vii) Pb(OAc)_4_ (1.7 equiv), benzene, 80 °C, 30%, 94:6 er. (viii) **10** (1.0 equiv), LiHMDS (6.0 equiv), allylic bromide (2.0 equiv), THF, −78 to 25 °C, 5 h, 40% (b.r.s.m. 80%), 95:5 er. TsCl, *p*-toluenesulfonyl chloride. DMAP, 4-(dimethylamino)pyridine. LiHMDS, lithium bis(trimethylsilyl)amide. Comins’ reagent, *N*-(5-chloro-2-pyridyl)bis(trifluoromethanesulfonimide). THF, tetrahydrofuran.

Considering the potential significance and accessibility of the novel and enantioenriched bicyclo[3.2.2]cyclohepta[*b*]indole frameworks, we sought to explore various synthetic transformations starting from adduct **4a** (Scheme 2B). First, a two-step Beckmann rearrangement was employed to prepare lactam **9** without deterioration of enantiopurity.^10^ After *N*-Boc-protection, ketone **10** could be converted into corresponding enol triflate **11** via Comins’ reagent and was applied in a Suzuki coupling to furnish diene **12**, which could be used for further transformations such as a Diels–Alder reaction. Besides, in an attempt to oxidatively cyclize the single diastereoisomer of alcohol **13** in a Suarez-type alkoxy radical-mediated reaction with Pb(OAc)_4_,^11^ we isolated rearranged spirooxindole **14** as the sole product. Allylation of ketone **10** proceeded to give **15** as a single diastereoisomer.

Finally, we were keen on understanding the underlying reaction mechanism. Reaction with cyclopentadiene or dienolether **2b** exclusively yielded complex reaction mixtures along with traces of (3 + 2) cycloaddition dimer (Scheme 3A, (i)). Moreover, addition of molecular sieves completely shut down reactivity, suggesting the crucial influence of the active species in the catalytic cycle (Scheme 3A, (ii)). When *N*-methylated 2-indolyl alcohol **1s** was used, no reactivity was observed, pointing toward NH hydrogen bonding as prerequisite for conversion. To gain deeper insight into the reaction progress, a ^31^P NMR study was conducted (for ^1^H NMR reaction progress kinetic analysis with different electronically biased substrates, see Supporting Information). Isolated IDPi **6d** (1.0 equiv) resonates as a sharp singlet at −15.0 ppm (Scheme 3B, (i)) and was almost completely converted into a new species showing two doublets (*J*_*pp*_ = 111.3 Hz) at −8.6 and −12.7 ppm upon addition of an excess of dienolsilane **2a** (Scheme 3B, (ii)). This desymmetrization can most likely be traced back to immediate silylation of one of the diastereotopic oxygen atoms of the IDPi’s inner sulfonamide core. Interestingly, after addition of 1.0 equiv of starting material **1a**, protonated IDPi **6d** was regenerated, indicating silyl transfer.

**Scheme 3:**
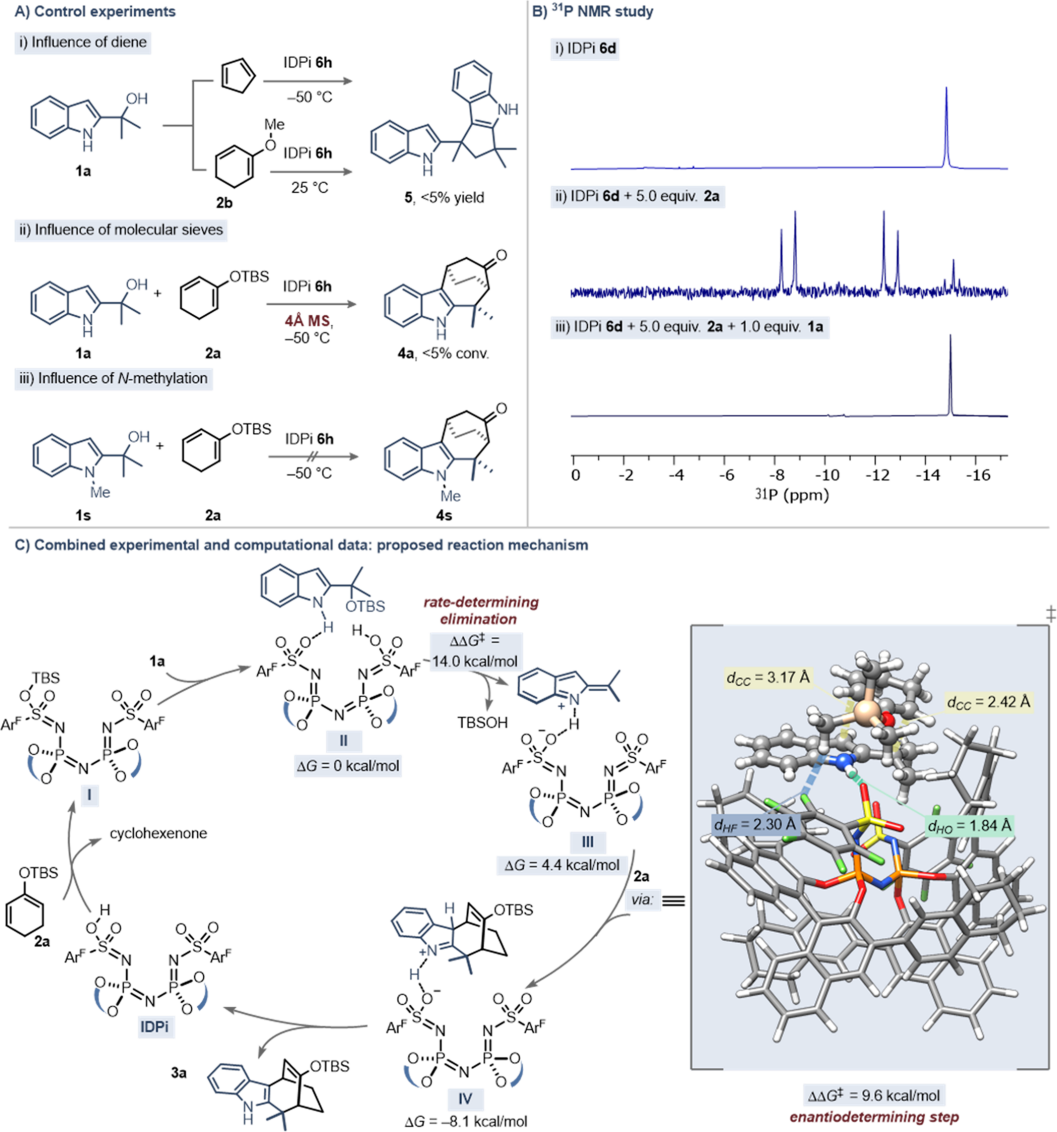
Mechanistic Studies (A) Control experiments. (B) ^31^P NMR study. (C) DFT-supported catalytic cycle (B3LYP-D3(BJ)/def2-TZVP+CPCM-(dichloromethane)//PBE-D3/def2-SVP level with TMS-dienolsilane as model).

Taking all of the experimentally gathered data into account, we propose a mechanistic cycle based on DFT ground-state and transition state energies. The ionization step occurring after substrate silylation was indeed found to be rate-limiting (14.0 kcal/mol). Besides, hydrogen bonding between the 2*H*-indolium and an inner sulfonamide core oxygen atom was shown to be a crucial catalyst–substrate interaction (*d*(NH–O) = 1.84 Å). Moreover, in line with previous reports by Jacobsen^3c^ and Houk,^12^ all our attempts to identify a stepwise mechanism remained futile. However, we identified a concerted yet highly asynchronous (4 + 3) cycloaddition, demonstrating a significant bond length difference in the major transition state between C10–C11 (2.42 Å) compared to C9–C14 (3.17 Å) (Scheme 3C). Additionally, the enantiofacial discrimination of the dienolsilane appears to be controlled by a nonclassical hydrogen bond between one of the TMS methyl groups and an aromatic fluorine atom of the inner catalyst core (2.30 Å, see the Supporting Information). All these indications lay the foundation for the mechanism of this catalytic asymmetric (4 + 3) cycloaddition. The strong Brønsted IDPi as the resting state commences to react with dienolsilane **2a** to form the active silylium Lewis acid **I**. The following reaction with the substrate leads to the formation of complex **II**, which reacts further via C–O bond cleavage to liberate TBSOH and intermediate **III** in the rate-limiting step. Subsequent highly exergonic asynchronous-concerted cycloaddition immediately yields **IV**, followed by rearomatization to deliver product **3a** and the IDPi.

In conclusion, we present a newly designed and powerful catalytic asymmetric (4 + 3) cycloaddition of *gem*-dialkyl-substituted 2-indolyl alcohols with dienolsilanes. This transformation was made possible by the application of strongly acidic and confined IDPi catalysts, thus overcoming major limitations of previously investigated systems. Using this new method, a variety of bicyclo[3.2.2]cyclohepta[*b*]indoles are readily accessible in excellent yields and enantioselectivities. By a combination of kinetic and computational studies, we disclose the crucial influence of the silylium species within the catalytic cycle and expect our approach to be valuable for the rapid and efficient asymmetric synthesis of several potentially biologically active natural products.

## Supporting Information Available

Experimental details and analytical data for all new compounds, HPLC traces, NMR spectra, and computational studies (optimized structures, and Cartesian coordinates) (PDF)

## Accession Codes

CCDC 2155912 contains the supplementary crystallographic data for this paper. These data can be obtained free of charge via www.ccdc.cam.ac.uk/data_request/cif, or by emailing data_request@ccdc.cam.ac.uk, or by contacting The Cambridge Crystallographic Data Centre, 12 Union Road, Cambridge CB2 1EZ, UK; fax: +44 1223 336033.
