# Supplementary material for: Design of an Organocatalytic Asymmetric (4 + 3) Cycloaddition of 2-Indolylalcohols with Dienolsilanes
Source: J Am Chem Soc. 2022 May 6;144(19):8460–6. doi: 10.1021/jacs.2c02216 (PMC9121375; doi:10.1021/jacs.2c02216)
Supplement: Supplementary file 1 — ja2c02216_si_001.pdf [file ja2c02216_si_001.pdf]

# Design of an Organocatalytic Asymmetric (4+3) Cycloaddition of 2-Indolylalcohols with Dienolsilanes

Jie Ouyang, Rajat Maji, Markus Leutzsch, Benjamin Mitschke, and Benjamin List*

Max-Planck-Institut für Kohlenforschung, Kaiser-Wilhelm-Platz 1, 45470 Mülheim an der Ruhr, Germany

*Email: list@kofo.mpg.de

# Supporting information

## Contents

1. General Information ........ S2
2. Evaluation of IDPis Catalysts ........ S4
3. General Procedure for the Synthesis of Substrates **1**: ........ S5
4. General Procedure of the Catalytic Asymmetric (4+3) Cycloaddition ........ S12
5. Derivatization of Ketone **4a** ........ S23
6. Synthesis of catalysts ........ S28
7. Mechanistic Investigations ........ S35
8. Reaction Progress Analysis by NMR ........ S36
9. Crystal Data of **4a** ........ S41
10. Control Experiments ........ S47
11. Limitations of this (4+3) Cycloaddition ........ S47
12. Computational Studies ........ S49
  12.1 Method ........ S49
  12.2 Analysis ........ S49
  12.3 Optimized Cartesian Coordinates PBE-D3/def2-SVP ........ S55
13. References ........ S126
14. NMR ans HPLC traces ........ S127

# 1. General Information

**Chemicals**

Chemicals were purchased from commercial suppliers (including abcr, Acros, Alfa Aesar, Fluorochem, TCI and Sigma-Aldrich) and used without further purification unless otherwise stated.

**Solvents**

Solvents ($CH_2Cl_2$, $CHCl_3$, $Et_2O$, THF, toluene) were dried by distillation from an appropriate drying agent in the technical department of the Max-Planck-Institut für Kohlenforschung and received in Schlenk flasks under argon. In addition, more solvents (acetone, benzene, cyclohexane, methylcyclohexane, 1,4-dioxane, DMF, DMSO, EtOAc, EtOH, MeCN, MeOH, MTBE, *n*-hexane, *n*-heptane, *n*-pentane, dichloromethylene) were purchased from commercial suppliers and dried over molecular sieves.

**Inert Gas**

Dry argon was purchased from Air Liquide with > 99.5% purity.

**Thin Layer chromatography**

Thin-layer chromatography (TLC) was performed using silica gel pre-coated glass plates (SIL G-25, with fluorescent indicator UV254; Macherey-Nagel) and aluminium oxide pre-coated plastic sheets (Polygram Alox N, 0.2 mm, with fluorescent indicator UV254; Macherey-Nagel), which were visualized by irradiation with UV light ($\lambda$ = 254 or 366 nm), basic $KMnO_4$, and/or phosphomolybdic acid (PMA). Preparative thin-layer chromatography was performed on silica gel pre-coated glass plates SIL G-100, with fluorescent indicator UV254 (Macherey-Nagel).

**Column Chromatography**

Column chromatography (CC) was carried out using Merck silica gel (60 Å, 230−400 mesh, particle size 0.040−0.063 mm) using technical grade solvents. Elution was accelerated using compressed air. All reported yields, unless otherwise specified, refer to spectroscopically and chromatographically pure compounds.

**Nuclear Magnetic Resonance Spectroscopy**

$^{1}H$, $^{13}C$, $^{19}F$, $^{31}P$ nuclear magnetic resonance (NMR) spectra were recorded on a Bruker AV-500, AV-400 spectrometer in a suitable deuterated solvent. The solvent employed and respective measuring frequency are indicated for each experiment. Chemical shifts are reported with tetramethylsilane (TMS) serving as a universal reference of all nuclides and with two or one digits after the comma. The resonance multiplicity is described as s (singlet), d (doublet), t (triplet), q (quartet), m (multiplet), and bs (broad singlet). All spectra were recorded at 298 K unless otherwise noted, processed with program MestReNova 14.0, and coupling constants are reported as observed. The residual deuterated solvent signal relative to tetramethylsilane (TMS) was used as the internal reference in $^{1}H$ NMR spectra ($CDCl_3$ $\delta$ 7.26, $CD_2Cl_2$ $\delta$ 5.32, $C_6D_6$ $\delta$ 7.16, $CD_3OD$ $\delta$ 3.31), and are reported as follows: chemical shift $\delta$ in ppm (multiplicity, coupling constant *J* in Hz, number of protons). $^{13}C$ NMR spectra reported in ppm from tetramethylsilane (TMS) with the solvent resonance as the internal standard ($CDCl_3$ $\delta$ 77.2, $CD_2Cl_2$ $\delta$ 53.8, $C_6D_6$ $\delta$ 128.1, $CD_3OD$ $\delta$ 49.0). $^{19}F$ and $^{31}P$ NMR shifts are reported relative to $CFCl_3$ ($\delta$=0) and $H_2PO_3$($\delta$=0 ppm) respectively. All spectra are broadband decoupled unless otherwise noted.

**Mass Spectrometry**

Electron impact (EI) mass spectrometry (MS) was performed on a Finnigan MAT 8200 (70 eV) or MAT 8400 (70 eV) spectrometer. Electrospray ionization (ESI) mass spectrometry was conducted on a Bruker ESQ 3000 spectrometer. High resolution mass spectrometry (HRMS) was performed on a Finnigan MAT 95 (EI) or Bruker APEX III FTMS (7T magnet, ESI). The ionization method and mode of detection employed is indicated for the respective experiment and all masses are reported in atomic units per elementary charge (m/z) with an intensity normalized to the most intense peak.

**High Performance Liquid Chromatography**

High performance liquid chromatography (HPLC) was performed on Shimadzu LC-20AD liquid chromatograph (SIL-20AC auto sampler, CMB-20A communication bus module, DGU-20A5 degasser, CTO-20AC column oven, SPD-M20A diode array detector), Shimadzu LC-20AB liquid chromatograph (SIL-20ACHT auto sampler, DGU-20A5 degasser, CTO-20AC column oven, SPD-M20A diode array detector), or Shimadzu LC-20AB liquid chromatograph (reversed phase, SIL-20ACHT auto sampler, CTO-20AC column oven, SPD-M20A diode array detector) using Daicel columns with a chiral stationary phase. All solvents used were HPLC-grade solvents purchased from Sigma-Aldrich. The column employed and respective solvent mixture are indicated for each experiment.

## 2. Evaluation of IDPis Catalysts

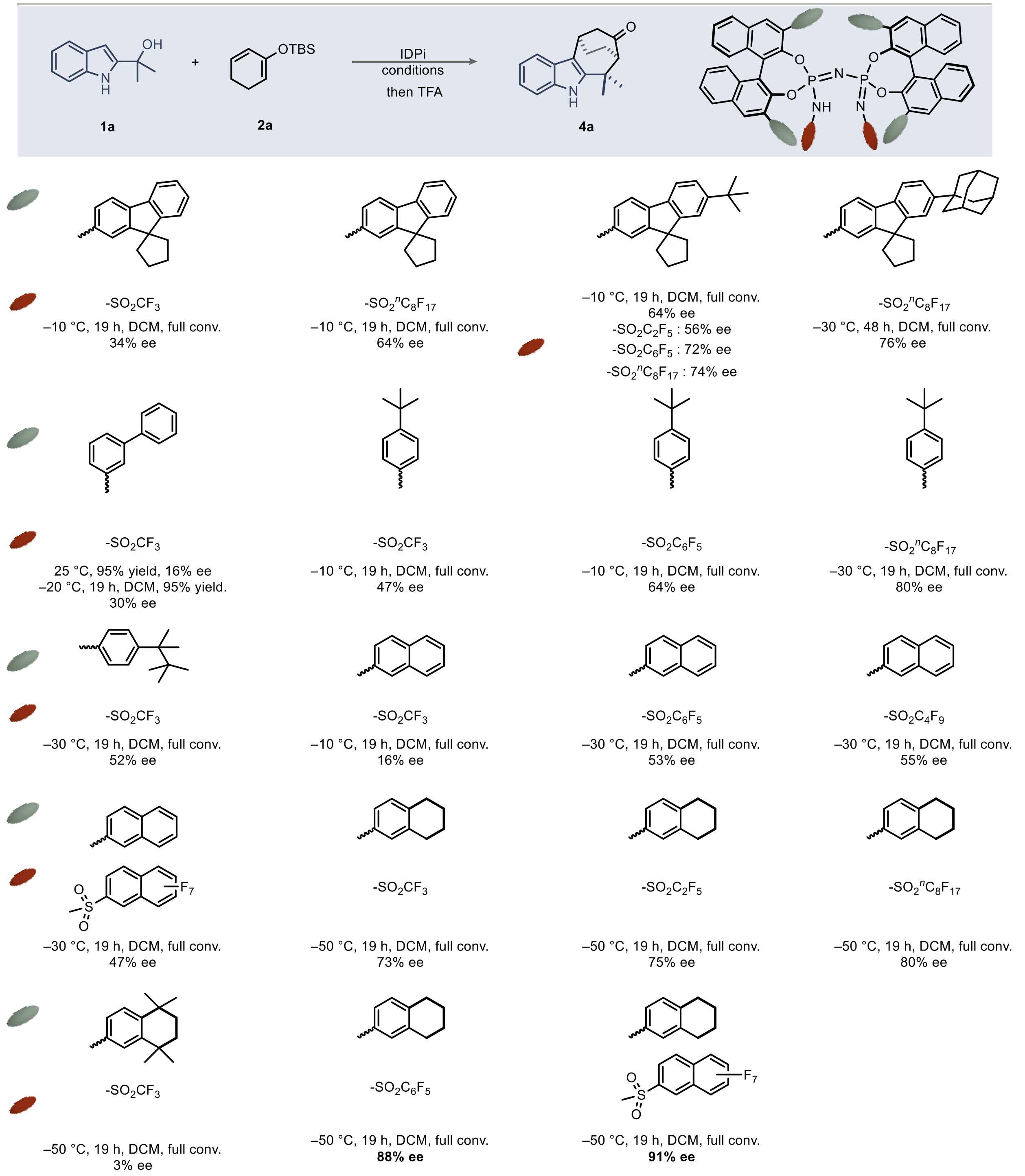


Figure 1. Catalyst screening

[a]Reactions were performed with substrate **1a** (0.01 mmol), catalyst (2.5 mol %), in indicated solvent (0.4 mL); the conversion was obtained by $^1H$ NMR analysis with 1,3,5-trimethoxybenzene as an internal standard; after deprotection by trifluoroacetic acid (10 uL), enantiomeric ratios (er) of **4** were measured by HPLC. DCM, dichloromethane;

## 3. General Procedure for the Synthesis of Substrates 1:

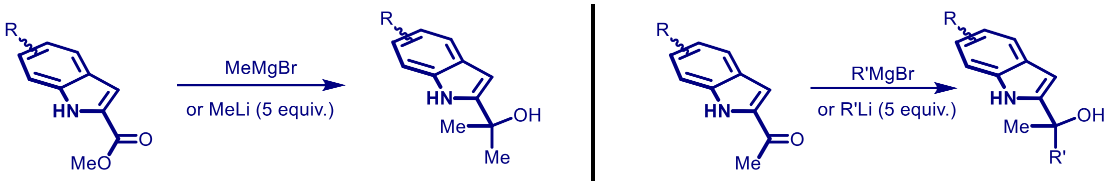


A solution of the corresponding indole ester or ketone in dryTHF under Ar was cooled with dry ice-acetone bath. After the addition of the indicated Grignard or organolithium reagent (5.0 equiv.), the solution was allowed to warm up to 25 °C under Ar. The reaction was stirred until full consumption of the starting material; the reaction mixture was treated with distilled $H_2O$ and extracted with ethyl acetate twice. The combined organic layer was collected, dried over anhydrous $MgSO_4$, concentrated under reduced pressure. The residue was purified by neutral alox column chromatography (alox was deactivated with 10% water, hexane: EtOAc = 10:1 to 4:1) to afford a yellow solid, which was crystallized in ethyl acetate and hexane to afford the desired products for the cycloadditions.

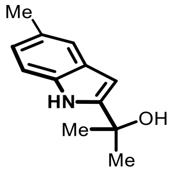


**2-(5-Methyl-1*H*-indol-2-yl)propan-2-ol (1b)**

Following the general procedure with 5-methylindole-2carboxylic acid ethyl ester (1.0 g, 4.9 mmol) and methyl lithium (1.6 M in $Et_2O$, 5.0 equiv) in THF (20 mL), the title compound was isolated as solid (810 mg, 87%).

$^1$H NMR (500 MHz, $CD_2Cl_2$) δ 8.44 (s, 1H), 7.32 (s, 1H), 7.23 (d, *J* = 8.3 Hz, 1H), 6.96 (d, *J* = 9.9 Hz, 0H), 6.21 (d, *J* = 2.3 Hz, 1H), 2.41 (s, 3H), 2.03 (s, 1H), 1.65 (s, 6H).

$^{13}$C NMR (125 MHz, $CD_2Cl_2$) δ 146.4, 134.3, 129.2, 123.5, 120.3, 110.8, 96.8, 69.9, 30.9, 21.5.

API-HRMS: calculated for $C_{12}H_{14}N_1O_1$ ($[M-H]^-$): 188.108088, found: 188.108150.

IR (film): 3465, 3257, 2985, 1169, 798.

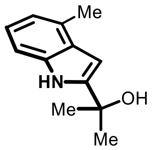


**2-(4-Methyl-1*H*-indol-2-yl)propan-2-ol (1c)**

Following the general procedure with 4-methylindole-2-carboxylic acid ethyl ester (1.27 g, 6.4 mmol) and methyl lithium (1.6 M in Et2O, 5.0 equiv) in THF (20 mL), the title compound was isolated as solid (1g, 84.5%).

$^1$H NMR (501 MHz, $CD_2Cl_2$) δ 8.52 (s, 1H), 7.18 (d, *J* = 8.8 Hz, 0H), 7.09 – 6.95 (m, 1H), 6.85 (d, *J* = 8.0 Hz, 0H), 6.32 (dd, *J* = 2.2, 0.9 Hz, 1H), 2.51 (s, 3H), 1.99 (s, 1H), 1.68 (s, 6H).

$^{13}$C NMR (126 MHz, $CD_2Cl_2$) δ 145.2, 135.2, 129.7, 128.3, 121.6, 119.7, 108.3, 95.3, 69.5, 30.6, 18.4.

ESI-HRMS: calculated for $C_{12}H_{14}N_1O_1$ ($[M-H]^-$): 188.108089, found: 188.108170.

IR (film): 3517, 3275, 2974, 1263,767,763.

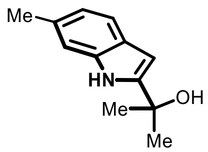

**2-(6-Methyl-1*H*-indol-2-yl)propan-2-ol (1d)**

Following the general procedure with 6-methylindole-2-carboxylic acid ethyl ester (1 g, 4.9 mmol) and methyl lithium (1.6 M in $Et_2O$, 5.0 equiv) in THF (15 mL), the title compound was isolated as solid (820 mg, 88%).

$^1$H NMR (501 MHz, $CD_2Cl_2$) δ 8.44 (s, 1H), 7.41 (d, *J* = 8.0 Hz, 1H), 7.13 (s, 1H), 6.90 (dd, *J* = 8.1, 1.5 Hz, 1H), 6.24 (dd, *J* = 2.3, 0.9 Hz, 1H), 2.44 (s, 3H), 2.09 (s, 0H), 1.65 (s, 6H).

$^{13}$C NMR (126 MHz, $CD_2Cl_2$) δ 145.6, 136.5, 131.8, 126.6, 121.7, 120.2, 111.1, 97.0, 69.9, 30.8, 21.8.

ESI-HRMS: calculated for $C_{12}H_{14}N_1O_1$ ([M-H]$^-$): 188.108089, found: 188.108130.

IR (film): 3437, 3288, 2976, 808.

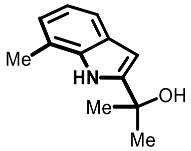

**2-(7-Methyl-1*H*-indol-2-yl)propan-2-ol (1e)**

Following the general procedure with 7-methylindole-2-carboxylic acid methyl ester (515 mg, 2.72 mmol) and methyl lithium (1.6 M in $Et_2O$, 5.0 equiv) in THF (15 mL), the title compound was isolated as solid (490 mg, 95%).

$^1$H NMR (501 MHz, $CD_3CN$) δ 9.06 (s, 1H), 7.32 (d, *J* = 7.5 Hz, 1H), 6.99 – 6.59 (m, 2H), 6.27 (q, *J* = 1.4, 0.9 Hz, 1H), 3.32 (s, 0H), 2.49 (s, 3H), 1.61 (s, 3H).

$^{13}$C NMR (126 MHz, $CD_3CN$) δ 147.9, 136.4, 128.9, 122.6, 121.4, 120.4, 118.5, 97.8, 69.6, 30.9, 17.2.

ESI-HRMS: calculated for $C_{12}H_{14}N_1O_1$ ([M-H]$^-$): 188.108089, found: 188.108100.

IR (film): 3502, 3289, 2973, 1298, 1128, 794.

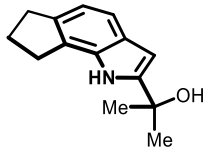

**2-(1,6,7,8-Tetrahydrocyclopenta[g]indol-2-yl)propan-2-ol (1f)**

Following the general procedure with 1,6,7,8-tetrahydrocyclopenta[g]indole-2-carboxylate (83 mg, 0.38 mmol) and methyl lithium (1.6 M in $Et_2O$, 5.0 equiv) in THF (5 mL), the title compound was isolated as solid (75 mg, 90%).

$^1$H NMR (501 MHz, $CD_2Cl_2$) δ 8.37 (s, 1H), 7.32 (d, *J* = 7.9 Hz, 1H), 6.98 (d, *J* = 8.0 Hz, 1H), 6.28 (d, *J* = 2.2 Hz, 1H), 3.03 (dt, *J* = 14.5, 7.3 Hz, 4H), 2.20 (p, *J* = 7.4 Hz, 2H), 2.03 (s, 1H), 1.67 (s, 6H).

$^{13}$C NMR (126 MHz, $CD_2Cl_2$) δ 145.4, 138.5, 133.2, 127.4, 125.6, 118.6, 116.9, 97.7, 69.9, 33.5, 30.9, 30.3, 25.9.

ESI-HRMS: calculated for $C_{14}H_{17}N_1O_1Na_1$ ([M+Na]$^+$): 238.120233, found: 238.120490.

IR (film): 3485, 3261, 2966, 1172, 810.

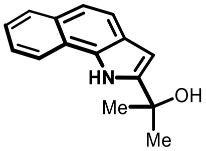

**2-(1*H*-benzo[g]indol-2-yl)propan-2-ol (1g)**

Following the general procedure with ethyl 1*H*-benzo[g]indole-2-carboxylate (170 mg, 0.71 mmol) and methyl lithium (1.6 M in $Et_2O$, 5.0 equiv) in THF (10 mL), the title compound was isolated as solid (150 mg, 94%).

$^1$H NMR (501 MHz, $CD_3CN$) δ 9.98 (s, 1H), 8.28 (d, $J$ = 8.2 Hz, 1H), 7.90 (d, $J$ = 8.1 Hz, 1H), 7.63 (d, $J$ = 8.5 Hz, 1H), 7.56 – 7.49 (m, 1H), 7.46 (d, $J$ = 8.5 Hz, 1H), 7.40 (t, $J$ = 7.5 Hz, 1H), 6.44 (d, $J$ = 2.5 Hz, 1H), 3.34 (d, $J$ = 2.0 Hz, 1H), 1.67 (s, 5H).

$^{13}$C NMR (126 MHz, $CD_3CN$) δ 146.6, 131.0, 129.4, 126.2, 125.0, 124.4, 122.9, 121.5, 121.3, 120.9, 99.2, 69.6, 31.1.

ESI-HRMS: calculated for $C_{15}H_{14}N_1O_1$ ([M-H]$^-$): 224.108089, found: 224.108240.

IR (film): 3530, 3284, 1124, 818, 747.

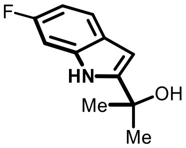

**2-(6-Fluoro-1*H*-indol-2-yl)propan-2-ol (1h)**

Following the general procedure with ethyl 6-fluoro-1*H*-indole-2-carboxylate (1.08 g, 4.83 mmol) and methyl lithium (1.6 M in $Et_2O$, 5.0 equiv) in THF (20 mL), the title compound was isolated as solid (900 mg, 96%).

$^1$H NMR (501 MHz, $CD_2Cl_2$) δ 8.69 (s, 1H), 7.46 (dd, $J$ = 8.6, 5.4 Hz, 1H), 7.04 (dd, $J$ = 9.8, 2.3 Hz, 1H), 6.95 – 6.72 (m, 1H), 6.29 (d, $J$ = 2.1 Hz, 1H), 2.20 (d, $J$ = 3.9 Hz, 0H), 1.66 (s, 6H).

$^{13}$C NMR (126 MHz, $CD_2Cl_2$) δ 160.0 (d, $J$ = 235.8 Hz), 146.8 (d, $J$ = 3.7 Hz), 136.0 (d, $J$ = 12.9 Hz), 125.4, 121.4 (d, $J$ = 10.1 Hz), 108.5 (d, $J$ = 24.5 Hz), 97.5 (d, $J$ = 26.2 Hz), 97.2, 69.9, 30.8.

ESI-HRMS: calculated for $C_{11}H_{11}N_1O_1F_1$ ([M-H]$^-$): 192.083017, found: 192.083220.

IR (film):3492, 3249, 2975, 1139, 817.

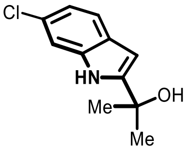

**2-(6-Chloro-1H-indol-2-yl)propan-2-ol (1i)**

Following the general procedure with ethyl 6-chloro-1*H*-indole-2-carboxylate (1.08 g, 4.47 mmol) and methyl lithium (1.6 M in $Et_2O$, 5.0 equiv) in THF (20 mL), the title compound was isolated as solid (800 mg, 85%).

$^1$H NMR (501 MHz, $CD_2Cl_2$) δ 8.75 (s, 1H), 7.47 (d, $J$ = 8.4 Hz, 1H), 7.34 (d, $J$ = 1.9 Hz, 1H), 7.05 (dd, $J$ = 8.4, 1.9 Hz, 1H), 6.29 (d, $J$ = 2.2 Hz, 1H), 2.27 (s, 1H), 1.66 (s, 5H).

$^{13}$C NMR (126 MHz, $CD_2Cl_2$) δ 147.2, 136.4, 127.5 (d, $J$ = 8.1 Hz), 121.6, 120.6, 111.1, 97.3, 70.0, 30.8.

API-HRMS: calculated for $C_{11}H_{11}N_1O_1Cl_1$ ([M-H]$^-$): 208.053467, found: 208.053580.

IR (film): 3465, 3255, 2978, 812.

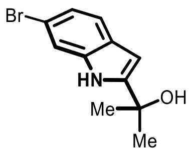

**2-(6-Bromo-1H-indol-2-yl)propan-2-ol (1j)**

Following the general procedure with ethyl 6-bromo-1H-indole-2-carboxylate (500 mg, 1.97 mmol) and methylmagnesium bromide solution (3 M in THF, 5.0 equiv.) in THF (10 mL), the title compound was isolated as solid (105 mg, 21%).

$^1$H NMR (501 MHz, $CD_3CN$) δ 9.39 (s, 1H), 7.54 (s, 0H), 7.41 (d, *J* = 8.4 Hz, 1H), 7.12 (dd, *J* = 8.4, 1.8 Hz, 1H), 6.27 (dd, *J* = 2.2, 0.9 Hz, 1H), 3.33 (s, 0H), 1.57 (s, 6H).

$^{13}$C NMR (126 MHz, $CD_2Cl_2$) δ 147.2, 136.8, 127.9, 123.2, 121.9, 115.0, 114.1, 97.3, 69.9, 30.9.

DIP-HRMS: calculated for $C_{11}H_{11}N_1O_1Br_1$ ($[M]^+$): 253.009689, found: 253.009910.

IR (film): 3459, 3257, 2984, 812.

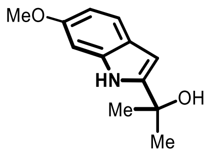

**2-(6-Methoxy-1H-indol-2-yl)propan-2-ol (1k)**

Following the general procedure with ethyl 1-(6-methoxy-1H-indol-2-yl)ethan-1-one (1 g, 4.56 mmol) and methyl lithium (1.6 M in $Et_2O$, 5.0 equiv) in THF (20 mL), the title compound was isolated as solid (700 mg, 75%).

$^1$H NMR (501 MHz, $CD_2Cl_2$) δ 8.41 (s, 1H), 7.38 (d, *J* = 8.5 Hz, 1H), 6.84 (d, *J* = 2.2 Hz, 1H), 6.71 (ddd, *J* = 8.5, 2.2, 0.8 Hz, 1H), 6.21 (d, *J* = 2.1 Hz, 1H), 3.81 (s, 3H), 1.64 (s, 6H).

$^{13}$C NMR (126 MHz, $CD_2Cl_2$) δ 156.6, 145.1, 136.8, 123.0, 121.2, 109.9, 97.0, 94.8, 69.8, 55.9, 30.8.

API-HRMS: calculated for $C_{12}H_{14}N_1O_2$ ($[M-H]^-$): 204.103004, found: 204.102990.

IR (film): 3410, 2975, 1247, 1154.

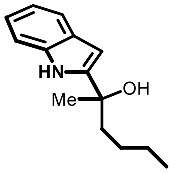

**2-(1*H*-indol-2-yl)hexan-2-ol (ll)**

Following the general procedure with 2-acetylindole (106 mg, 0.66 mmol) and *n*-butyllithium solution (2.5 M in hexane, 2.8 equiv.) in THF (10 mL), the title compound was isolated as solid (78 mg, 78%).

$^1$H NMR (501 MHz, $CD_2Cl_2$) δ 8.54 (s, 1H), 7.53 (dd, *J* = 7.8, 1.0 Hz, 1H), 7.35 (dd, *J* = 8.1, 1.0 Hz, 1H), 7.11 (ddd, *J* = 8.2, 7.1, 1.2 Hz, 1H), 7.04 (ddd, *J* = 8.1, 7.0, 1.1 Hz, 1H), 6.26 (dd, *J* = 2.2, 0.9 Hz, 1H), 1.97 (d, *J* = 2.3 Hz, 1H), 1.90 – 1.81 (m, 2H), 1.63 (s, 3H), 1.34 – 1.13 (m, 4H), 0.87 (t, *J* = 7.0 Hz, 3H).

$^{13}$C NMR (126 MHz, $CD_2Cl_2$) δ 145.5, 135.8, 129.1, 121.7, 120.5, 119.9, 111.2, 97.6, 72.5, 43.7, 29.3, 26.8, 23.4, 14.2.

API-HRMS: calculated for $C_{14}H_{18}N_1O_1$ ($[M-H]^-$): 216.139389, found: 216.139230.

IR (film): 3414, 3350, 2956, 1457, 788.

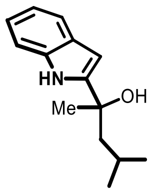

**2-(1*H*-indol-2-yl)-4-methylpentan-2-ol (1m)**

To a flame-dried two neck flask was added magnesium (366 mg, 15.07 mmol, 12 equiv.) in dry THF (10 mL), followed by the addition of iodine (one seed). To this mixture was added the 1-bromo-2-methylpropane (2 g, 14.6 mmol, 11.6 equiv.) at 25 °C. The resulting mixture was heated to 65 °C for 2 h to afford the Grignard solution for the next step use. To a solution of ethyl 2-acetylindole (200 mg, 1.25 mmol) in dry THF (10 mL) was added freshly prepared Grignard reagent (11 equiv.) at 25 °C under Ar. The solution was stirred overnight, and then the reaction workup proceeded following the general procedure to afford the title compound as a solid (210 mg, 77%).

$^1$H NMR (501 MHz, $CD_3CN$) δ 9.25 (s, 1H), 7.06 (ddd, *J* = 8.2, 7.0, 1.2 Hz, 1H), 6.99 (ddd, *J* = 8.0, 7.0, 1.1 Hz, 1H), 6.24 (dd, *J* = 2.2, 0.9 Hz, 1H), 3.15 (s, 1H), 1.75 (d, *J* = 5.8 Hz, 2H), 1.69 – 1.59 (m, 1H), 1.57 (s, 3H), 0.82 (d, *J* = 6.7 Hz, 3H), 0.74 (d, *J* = 6.6 Hz, 3H).

$^{13}$C NMR (126 MHz, $CD_3CN$) δ 147.4, 136.7, 129.5, 121.7, 120.7, 120.1, 111.8, 97.7, 72.5, 52.8, 30.3, 25.3, 24.6, 24.4.

ESI-HRMS: calculated for $C_{14}H_{18}N_1O_1$ ($[M-H]^-$): 216.139389, found: 216.139370.

IR (film): 3407, 3309, 2920, 813.

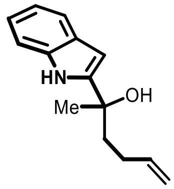

**2-(1*H*-indol-2-yl)hex-5-en-2-ol (1n)**

To a solution of 2-acetylindole (150 mg, 0.94 mmol) in dry THF (10 mL) was added freshly prepared 3-butenylmagnesium bromide solution (0.5 M in THF, 9 mL, 5 equiv.) at 25 °C under Ar. The solution was stirred overnight, and then the reaction workup proceeded following the general procedure to afford the title compound as a solid (189 mg, 93%).

$^1$H NMR (501 MHz, $CD_3CN$) δ 9.26 (s, 1H), 7.49 (dd, *J* = 7.9, 1.1 Hz, 1H), 7.37 (dd, *J* = 8.1, 1.0 Hz, 1H), 7.08 (ddd, *J* = 8.1, 7.0, 1.2 Hz, 1H), 7.00 (ddd, *J* = 8.0, 7.1, 1.1 Hz, 1H), 6.25 (dd, *J* = 2.2, 0.9 Hz, 1H), 5.81 (ddt, *J* = 16.9, 10.3, 6.3 Hz, 1H), 4.97 (dd, *J* = 17.2, 1.9 Hz, 1H), 4.92 – 4.81 (m, 1H), 3.27 (s, 1H), 2.11 – 2.04 (m, 1H), 1.98 – 1.87 (m, 3H), 1.58 (s, 3H).

$^{13}$C NMR (126 MHz, $CD_3CN$) δ 146.9, 139.9, 136.8, 129.5, 121.8, 120.7, 120.1, 114.6, 111.9, 97.8, 72.0, 43.4, 29.3 (d, *J* = 19.9 Hz).

API-HRMS: calculated for $C_{14}H_{16}N_1O_1$ ($[M-H]^-$): 214.123739, found: 214.123870.

IR (film): 3415, 3380, 2976, 1457, 737.

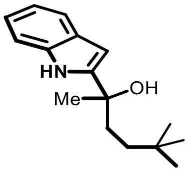

**2-(1*H*-indol-2-yl)-5,5-dimethylhexan-2-ol (lo)**

To a flame-dried two neck flask was added magnesium (160 mg, 6.6 mmol, 5 equiv.) in dry THF (10 mL), followed by the addition of iodine (one seed). To this mixture was added the 1-bromo-3,3-dimethylbutane (871 mg, 5.3 mmol, 4 equiv.) at 25 °C. The resulting mixture was heated to 65 °C for 2 h to afford the Grignard solution for the next step use. To a solution of 2-acetylindole (210 mg, 1.25 mmol) in dry THF (10 mL) was added freshly prepared Grignard reagent (4 equiv.) at 25 °C under Ar. The solution was stirred overnight, and then the reaction workup proceeded following the general procedure to afford the title compound as a solid (197 mg, 60%).

$^{1}$H NMR (501 MHz, $CD_3CN$) δ 9.23 (s, 1H), 7.48 (d, *J* = 7.8 Hz, 1H), 7.36 (d, *J* = 8.1 Hz, 1H), 7.07 (ddd, *J* = 8.2, 7.0, 1.2 Hz, 1H), 7.04 – 6.86 (m, 1H), 6.23 (d, *J* = 2.2 Hz, 1H), 3.17 (t, *J* = 1.4 Hz, 1H), 1.81 (dd, *J* = 9.8, 7.5 Hz, 2H), 1.55 (s, 3H), 1.18 (ddd, *J* = 13.2, 9.9, 7.4 Hz, 1H), 1.07 (ddd, *J* = 13.2, 9.8, 7.4 Hz, 1H), 0.84 (s, 9H).

$^{13}$C NMR (126 MHz, $CD_3CN$) δ 147.3, 136.8, 129.5, 121.7, 120.7, 120.0, 111.8, 97.7, 72.2, 39.0, 38.6, 30.4, 29.6, 29.3.

DIP-HRMS: calculated for $C_{16}H_{23}N_1O_1$ ([M]$^+$): 245.177414, found: 245.177440.

IR (film): 3418, 3315, 2953, 1364, 749.

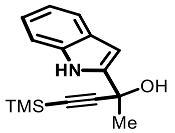


**2-(1*H*-indol-2-yl)-4-(trimethylsilyl)but-3-yn-2-ol (1p)**

To a solution of trimethylsilylacetylene (493 mg, 5 mmol, 4 equiv.) in dry THF was added n-butyllithium (2.5 M in hexane, 2 mL, 5 mmol, 4 equiv.) at –78 °C under Ar. The solution was stirred for 30 min at this temperature. To this solution was added the 2-acetylindole (200 mg, 1.25 mmol) in dry THF (2 mL), the resulting mixture was stirred for 1 h and was allowed to warm to 25 °C until the all ketone was consumed (monitored by TLC). The reaction proceeded following the general procedure to afford the title compound as solid (270 mg, 83%).

$^{1}$H NMR (300 MHz, $CD_3CN$) δ 9.34 (s, 1H), 7.53 (ddt, *J* = 7.8, 1.4, 0.8 Hz, 1H), 7.41 (dq, *J* = 8.1, 0.9 Hz, 1H), 7.13 (ddd, *J* = 8.2, 7.1, 1.3 Hz, 1H), 7.03 (ddd, *J* = 8.1, 7.1, 1.1 Hz, 1H), 6.46 (dd, *J* = 2.2, 0.9 Hz, 1H), 4.08 (s, 1H), 1.80 (s, 3H), 0.20 (s, 9H).

$^{13}$C NMR (75 MHz, $CD_3CN$) δ 143.6, 137.3, 128.9, 122.7, 121.3, 120.4, 112.1, 109.3, 99.0, 88.4, 66.1, 31.6.

DIP-HRMS: calculated for $C_{15}H_{19}N_1O_1Si_1$ ([M]$^+$): 257.123042, found: 257.123310.

IR (film): 3408, 3304, 2171, 1456, 841.

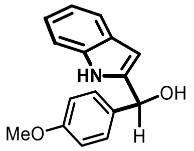


**(1*H*-indol-2-yl)(4-methoxyphenyl)methanol (1q)**

To a flame-dried two-neck flask was charged with indole-2-carboxaldehyde (200 mg, 1.38 mmol), dissolved in dry THF (10 mL), followed by the addition of 4-methoxyphenylmagnesium bromide solution (0.5 M in THF, 4.6 mL, 4 equiv.) at 25 °C. The solution was stirred for 4 h until all aldehyde was consumed, and then the reaction mixture proceeded following the general procedure to afford the title compound as solid (269 mg, 77%)

$^{1}$H NMR (501 MHz, $CD_3CN$) δ 9.27 (s, 1H), 7.47 (dd, *J* = 7.9, 1.1 Hz, 1H), 7.38 – 7.31 (m, 3H), 7.08 (ddd, *J* = 8.2, 7.0, 1.2 Hz, 1H), 6.99 (ddd, *J* = 8.0, 7.0, 1.1 Hz, 1H), 6.91 (d, *J* = 6.8 Hz, 2H), 6.25 – 6.11 (m, 1H), 5.89 (d, *J* = 4.4 Hz, 1H), 3.95 (d, *J* = 4.5 Hz, 1H), 3.78 (s, 3H).

$^{1}H$ NMR (501 MHz, $CD_3CN$) δ 9.27 (s, 1H), 7.47 (dd, *J* = 7.9, 1.1 Hz, 1H), 7.38 – 7.31 (m, 3H), 7.08 (ddd, *J* = 8.2, 7.0, 1.2 Hz, 1H), 6.99 (ddd, *J* = 8.0, 7.0, 1.1 Hz, 1H), 6.91 (d, *J* = 6.8 Hz, 2H), 6.25 – 6.11 (m, 1H), 5.89 (d, *J* = 4.4 Hz, 1H), 3.95 (d, *J* = 4.5 Hz, 1H), 3.78 (s, 3H).
DIP-HRMS: calculated for $C_{16}H_{15}N_1O_2$ ($[M]^+$): 253.109729, found: 253.110020.
IR (film): 3399, 2836, 1510, 592.

## 4. General Procedure of the Catalytic Asymmetric (4+3) Cycloaddition

A flame-dried 5 mL vial equipped with a magnetic stirrer was charged with substrate **1** (0.1 mmol to 0.2 mmol, 1.0 equiv.), diene **2a**[1] (4.0 equiv.) and dichloromethane (2 mL) was added at room temperature. The resulting solution was cooled to –78 °C in dry ice, IDPi (2 mol %) was added under Ar, and the vial was transferred to a –50 °C cryostat. The reaction was stirred for 16 h to 3 days (monitored by TLC). After full consumption of the starting material, the reaction mixture was treated with triethylamine (5.0 mol %), the vial was allowed to warm up to room temperature, and trifluoroacetic acid (1.2 equiv.) was added. After complete of desilylation, the crude mixture was concentrated under reduced pressure, and the residue was purified by silica gel column chromatography (hexane:EtOAc = 10:1 to 4:1) to afford the desired product.

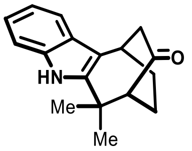


**(7*R*,10*R*)-6,6-dimethyl-6,7,9,10-tetrahydro-7,10-ethanocyclohepta[b]indol-8(5H)-one (4a)**
Indolyl alcohol **1a**[2] (20 mg, 0.11 mmol), diene **2a** (96 mg, 0.45 mmol, 4 equiv.), and IDPi **6i** (4.7 mg, 2 mol%) were subjected to the general procedure (16 h) to afford **4a** as a white solid (28.9 mg, 99%).
$^1$H NMR (500 MHz, $CD_2Cl_2$) δ 7.98 (s, 1H), 7.50 (d, *J* = 7.7 Hz, 1H), 7.31 (d, *J* = 7.7 Hz, 1H), 7.13 (t, *J* = 7.5 Hz, 1H), 7.08 (t, *J* = 7.5 Hz, 1H), 3.57 (dt, *J* = 4.7, 2.3 Hz, 1H), 2.68 (dd, *J* = 18.4, 4.5 Hz, 1H), 2.55 (dt, *J* = 18.4, 2.6 Hz, 1H), 2.41 – 2.37 (m, 1H), 2.37 – 2.25 (m, 1H), 2.15 – 1.96 (m, 3H), 1.47 (s, 3H), 1.40 (s, 3H).
$^{13}$C NMR (125 MHz, $CD_2Cl_2$) δ 214.5, 139.7, 135.9, 126.8, 121.8, 119.5, 117.9, 116.9, 110.8, 59.0, 47.6, 38.8, 30.4, 28.6, 28.2, 26.9, 20.7.
EI-HRMS: calculated for $C_{17}H_{19}N_1O_1$ ($[M]^+$): 253.146114, found: 253.146500.
HPLC (AD-3, *n*-heptane/*i*-PrOH=94:6, 1.2 mL/min, 298 K, 282 nm): $t_R$(minor) = 8.86 min, $t_R$(major) = 10.07 min. e.r. = 95.5:4.5 (91% ee).
$[\alpha]_D^{25}$ = +26 (c = 0.83, $CH_2Cl_2$).
IR (film): 3369, 2931, 1703, 745.

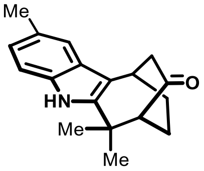


**(7*R*,10*R*)-2,6,6-trimethyl-6,7,9,10-tetrahydro-7,10-ethanocyclohepta[b]indol-8(5H)-one (4b)**
Indolyl alcohol **1b** (20 mg, 0.1 mmol), diene **2a** (89 mg, 0.42 mmol, 4 equiv.), and IDPi **6h** (3.5 mg, 2 mol%) were subjected to the general procedure (16 h) to afford **4b** as a white solid (28 mg, 99%).
$^1$H NMR (501 MHz, $CD_2Cl_2$) δ 7.82 (s, 1H), 7.27 – 7.23 (m, 1H), 7.16 (d, *J* = 8.2 Hz, 1H), 6.92 (dd, *J* = 8.2, 1.6 Hz, 1H), 3.50 (dp, *J* = 4.7, 2.4 Hz, 1H), 2.64 (ddd, *J* = 18.3, 4.4, 0.8 Hz, 1H), 2.50 (dt, *J* = 18.4, 2.5 Hz, 1H), 2.41 (s, 3H), 2.36 – 2.32 (m, 1H), 2.32 – 2.20 (m, 1H), 2.07 – 2.00 (m, 2H), 1.95 (ddq, *J* = 12.5, 7.6, 2.6 Hz, 1H), 1.42 (s, 3H), 1.35 (s, 3H).
$^{13}$C NMR (126 MHz, $CD_2Cl_2$) δ 214.5, 139.8, 134.2, 128.8, 127.0, 123.3, 117.7, 116.5, 110.4, 59.0, 47.6, 38.8, 30.4, 28.6, 28.2, 26.9, 21.6, 20.7.
EI-HRMS: calculated for $C_{18}H_{21}N_1O_1$ ($[M]^+$): 267.161764, found: 267.161980.

HPLC (AD-3, *n*-heptane/*i*-PrOH=94:6, 1.2 mL/min, 298 K, 282 nm): $t_R$(minor) = 6.89 min, $t_R$(major) = 7.66 min. e.r. = 96:4 (92% ee).
$[\alpha]_D^{25}$ = +25 (c = 0.85, $CH_2Cl_2$).
IR (film): 3371, 2926, 1701, 1466, 1308, 794.

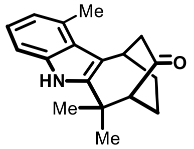

**(7*R*,10*R*)-1,6,6-trimethyl-6,7,9,10-tetrahydro-7,10-ethanocyclohepta[b]indol-8(5H)-one (4c)**
Indolyl alcohol **1c** (20 mg, 0.1 mmol), diene **2a** (89 mg, 0.42 mmol, 4 equiv.), and IDPi **6h** (3.5 mg, 2 mol%) were subjected to the general procedure (16 h) to afford **4c** as a white solid (28 mg, 98%).
$^1$H NMR (500 MHz, $CD_2Cl_2$) δ 8.00 (s, 1H), 7.14 (d, *J* = 8.1 Hz, 1H), 6.98 (t, *J* = 7.6 Hz, 1H), 6.79 (d, *J* = 7.1 Hz, 1H), 4.02 (tt, *J* = 4.7, 2.5 Hz, 1H), 2.72 – 2.67 (m, 1H),2.67 (s, 3H), 2.62 (dt, *J* = 18.4, 2.2 Hz, 1H), 2.37 (d, *J* = 4.9 Hz, 1H), 2.32 (ddd, *J* = 11.0, 9.0, 2.0 Hz, 1H), 2.15 – 1.99 (m, 3H), 1.47 (s, 3H), 1.40 (s, 3H).
$^{13}$C NMR (126 MHz, $CD_2Cl_2$) δ 214.7, 139.6, 135.9, 130.3, 125.0, 121.9, 121.6, 118.1, 108.9, 58.9, 47.7, 38.6, 30.5, 28.8, 28.3, 28.3, 21.4, 20.8.
EI-HRMS: calculated for $C_{18}H_{21}N_1O_1$ ([M]$^+$): 267.161764, found: 267.161940.
HPLC (AD-3, *n*-heptane/*i*-PrOH=94:6, 1.2 mL/min, 298 K, 280 nm): $t_R$(minor) = 5.49 min, $t_R$(major) = 7.11 min. e.r. = 95:5 (90% ee).
$[\alpha]_D^{25}$ = +15 (c = 0.55, $CH_2Cl_2$).
IR (film): 3372, 2928, 2864, 1705, 1461, 752.

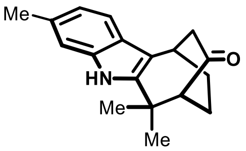

**(7*R*,10*R*)-3,6,6-trimethyl-6,7,9,10-tetrahydro-7,10-ethanocyclohepta[b]indol-8(5H)-one (4d)**
Indolyl alcohol **1d** (20 mg, 0.1 mmol), diene **2a** (89 mg, 0.42 mmol, 4 equiv.), and IDPi **6h** (3.5 mg, 2 mol%) were subjected to the general procedure (16 h) to afford **4d** as a white solid (28 mg, 99%).
$^1$H NMR (501 MHz, $CD_2Cl_2$) δ 7.78 (s, 1H), 7.33 (d, *J* = 8.0 Hz, 1H), 7.08 (d, *J* = 1.0 Hz, 1H), 6.89 (dd, *J* = 8.0, 1.4 Hz, 1H), 3.49 (tt, *J* = 4.7, 2.4 Hz, 1H), 2.63 (ddd, *J* = 18.3, 4.5, 0.8 Hz, 1H), 2.50 (dt, *J* = 18.3, 2.5 Hz, 1H), 2.42 (s, 3H), 2.34 – 2.32 (m, 1H), 2.30 – 2.21 (m, 1H), 2.11 – 1.87 (m, 3H), 1.42 (s, 3H), 1.35 (s, 3H).
$^{13}$C NMR (125 MHz, $CD_2Cl_2$) δ 214.5, 138.8, 136.3, 131.6, 124.7, 121.2, 117.6, 116.8, 110.7, 59.0, 47.6, 38.8, 30.4, 28.6, 28.2, 27.0, 21.7, 20.7.
EI-HRMS: calculated for $C_{18}H_{21}N_1O_1$ ([M]$^+$): 267.161764, found: 267.162120.
HPLC (AD-3, *n*-heptane/*i*-PrOH=94:6, 1.2 mL/min, 298 K, 282 nm): $t_R$(minor) = 8.56 min, $t_R$(major) = 9.98 min. e.r. = 95.5:4.5 (91% ee).
$[\alpha]_D^{25}$ = +14 (c = 0.42, $CH_2Cl_2$). IR (film): 3373, 2924, 1695, 1254, 773.

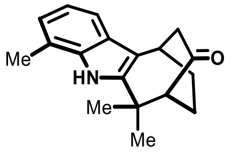

**(7*R*,10*R*)-4,6,6-trimethyl-6,7,9,10-tetrahydro-7,10-ethanocyclohepta[b]indol-8(5H)-one (4e)**
Indolyl alcohol **1e** (20 mg, 0.1 mmol), diene **2a** (89 mg, 0.42 mmol, 4 equiv.), and IDPi **6h** (3.5 mg, 2 mol%) were subjected to the general procedure (16 h) to afford **4e** as a white solid (28 mg, 99%).
$^{1}$H NMR (500 MHz, $CD_2Cl_2$) δ 7.81 (s, 1H), 7.35 (d, *J* = 7.9 Hz, 1H), 7.01 (t, *J* = 7.5 Hz, 1H), 6.94 (dt, *J* = 7.1, 1.0 Hz, 1H), 3.55 (tt, *J* = 4.7, 2.4 Hz, 1H), 2.68 (dd, *J* = 18.3, 4.4 Hz, 1H), 2.55 (dt, *J* = 18.3, 2.5 Hz, 1H), 2.49 (s, 3H), 2.41 – 2.37 (m, 1H), 2.36 – 2.24 (m, 1H), 2.16 – 1.94 (m, 3H), 1.49 (s, 3H), 1.42 (s, 3H).
$^{13}$C NMR (125 MHz, $CD_2Cl_2$) δ 214.5, 139.3, 135.3, 126.3, 122.5, 120.1, 119.8, 117.5, 115.6, 59.1, 47.6, 38.9, 30.4, 28.6, 28.2, 27.1, 20.7, 16.8.
EI-HRMS: calculated for $C_{18}H_{21}N_1O_1$ ($[M]^+$): 267.161764, found: 267.162140.
HPLC (IB-3, *n*-heptane/*i*-PrOH=94:6, 1.0 mL/min, 298 K, 282 nm): $t_R$(minor) = 7.23 min, $t_R$(major) = 9.06 min. e.r. = 98:2 (96% ee).
$[\alpha]_D^{25}$ = +14 (c = 0.6, $CH_2Cl_2$).
IR (film): 3374, 2932, 1699, 1463, 1308, 743.

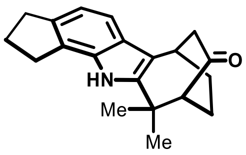

**(6*R*,9*R*)-10,10-dimethyl-2,3,6,7,8,9,10,11-octahydro-1H-6,9-ethanocyclohepta[b]cyclopenta[g]indol-12-one (4f)**
Indolyl alcohol **1f** (20 mg, 0.093 mmol), diene **2a** (78 mg, 0.37 mmol, 4 equiv.), and IDPi **6h** (3.8 mg, 2.5 mol%) were subjected to the general procedure (16 h) to afford **4f** as a white solid (23 mg, 84%).
$^{1}$H NMR (501 MHz, $CD_2Cl_2$) δ 7.69 (s, 1H), 7.28 (d, *J* = 8.0 Hz, 1H), 7.01 (d, *J* = 8.0 Hz, 1H), 3.53 (dt, *J* = 4.7, 2.3 Hz, 1H), 3.03 (td, *J* = 7.5, 4.4 Hz, 4H), 2.67 (dd, *J* = 18.3, 4.5 Hz, 1H), 2.53 (dt, *J* = 18.3, 2.5 Hz, 1H), 2.39 – 2.36 (m, 1H), 2.34 – 2.26 (m, 1H), 2.21 (p, *J* = 7.4 Hz, 2H), 2.12 – 2.05 (m, 2H), 1.99 (ddtt, *J* = 9.6, 6.8, 4.4, 2.2 Hz, 1H), 1.47 (s, 3H), 1.40 (s, 3H).
$^{13}$C NMR (125 MHz, $CD_2Cl_2$) δ 214.5, 138.6, 138.5, 132.9, 125.4, 125.3, 117.5, 116.4, 115.9, 59.1, 47.6, 38.8, 33.4, 30.4, 30.2, 28.7, 28.2, 27.2, 26.0, 20.7.
EI-HRMS: calculated for $C_{20}H_{23}N_1O_1$ ($[M]^+$): 293.177414, found: 293.177800.
HPLC (IB-3, *n*-heptane/*i*-PrOH=94:6, 1.0 mL/min, 298 K, 282 nm): $t_R$(minor) = 6.25 min, $t_R$(major) = 8.69 min. e.r. = 96:4 (92% ee).
$[\alpha]_D^{25}$ = +8.3 (c = 0.24, $CH_2Cl_2$).
IR (film): 3375, 2926, 1703, 1453, 1318, 800.

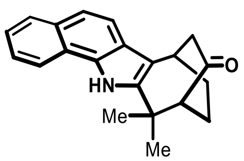

**(7*R*,10*R*)-11,11-dimethyl-7,10,11,12-tetrahydro-7,10-ethanobenzo[g]cyclohepta[b]indol-**

**9(8H)-one (4g)**
Indolyl alcohol **1g** (20 mg, 0.093 mmol), diene **2a** (78 mg, 0.37 mmol, 4.0 equiv.), and IDPi **6h** (3.0 mg, 2 mol%) were subjected to the general procedure (16 h) to afford **4g** as a white solid (27.6 mg, 98%).
$^{1}$H NMR (500 MHz, $CD_2Cl_2$) δ 8.67 (s, 1H), 8.05 (dd, *J* = 8.2, 1.1 Hz, 1H), 7.93 (d, *J* = 8.1 Hz, 1H), 7.64 (d, *J* = 8.6 Hz, 1H), 7.57 – 7.54 (m, 1H), 7.53 – 7.50 (m, 1H), 7.43 (ddd, *J* = 8.1, 6.9, 1.2 Hz, 1H), 3.66 (dt, *J* = 4.7, 2.3 Hz, 1H), 2.73 (dd, *J* = 18.4, 4.5 Hz, 1H), 2.60 (dt, *J* = 18.4, 2.5 Hz, 1H), 2.48 – 2.42 (m, 1H), 2.40 – 2.31 (m, 1H), 2.21 – 2.01 (m, 3H), 1.55 (s, 3H), 1.49 (s, 3H). $^{13}$C NMR (126 MHz, $CD_2Cl_2$) δ 214.5, 138.1, 130.6, 130.0, 129.2, 125.8, 123.9, 122.3, 121.7, 120.4, 119.7, 118.9, 118.4, 59.1, 47.7, 38.9, 30.8, 29.0, 28.3, 27.1, 20.7.
EI-HRMS: calculated for $C_{21}H_{21}N_1O_1$ ($[M]^+$): 303.161764, found: 303.162340.
HPLC (IB-3, *n*-heptane/*i*-PrOH=94:6, 1.0 mL/min, 298 K, 282 nm): $t_R$(minor) = 9.60 min, $t_R$(major) = 15.47 min. e.r. = 98:2 (96% ee).
$[\alpha]_D^{25}$ = –13.5 (c = 0.4, $CH_2Cl_2$).
IR (film): 3334, 2923, 1682, 1388, 807, 751.

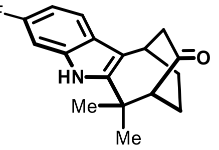


**(7*R*,10*R*)-3-fluoro-6,6-dimethyl-6,7,9,10-tetrahydro-7,10-ethanocyclohepta[b]indol-8(5H)-one (4h)**
Indolyl alcohol **1h** (20 mg, 0.1 mmol), diene **2a** (87 mg, 0.41 mmol, 4 equiv.), and IDPi **6i** (3.8 mg, 2 mol%) were subjected to the general procedure (16 h) to afford **4h** as a white solid (23 mg, 82%).
$^{19}$F NMR (471 MHz, $CD_2Cl_2$) δ -122.8.
$^{1}$H NMR (501 MHz, $CD_2Cl_2$) δ 7.97 (s, 1H), 7.42 (dd, *J* = 8.7, 5.3 Hz, 1H), 7.02 (dd, *J* = 9.7, 2.3 Hz, 1H), 6.86 (ddd, *J* = 9.7, 8.6, 2.3 Hz, 1H), 3.56 – 3.44 (m, 1H), 2.68 (dd, *J* = 18.3, 4.5 Hz, 1H), 2.54 (dt, *J* = 18.3, 2.5 Hz, 1H), 2.43 – 2.37 (m, 1H), 2.35 – 2.23 (m, 1H), 2.15 – 1.95 (m, 3H), 1.46 (s, 3H), 1.39 (s, 3H).
$^{13}$C NMR (125 MHz, $CD_2Cl_2$) δ 214.2, 160.0 (d, *J* = 235.9 Hz), 140.0 (d, *J* = 3.7 Hz), 135.7 (d, *J* = 12.5 Hz), 123.5, 118.6 (d, *J* = 10.1 Hz), 117.0, 107.9 (d, *J* = 24.4 Hz), 97.2 (d, *J* = 26.1 Hz), 58.9, 47.5, 38.8, 30.3, 28.5, 28.1, 26.9, 20.6.
EI-HRMS: calculated for $C_{17}H_{18}N_1O_1F_1$ ($[M]^+$): 271.136692, found: 271.136750.
HPLC (AD-3, *n*-heptane/*i*-PrOH=94:6, 1.2 mL/min, 298 K, 282 nm): $t_R$(minor) = 8.52 min, $t_R$(major) = 9.93 min. e.r. = 95:5 (90% ee).
$[\alpha]_D^{25}$ = +20 (c = 0.25, $CH_2Cl_2$).
IR (film): 3361, 2924, 1695, 1466, 1123, 858.

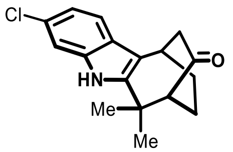


**(7*R*,10*R*)-3-chloro-6,6-dimethyl-6,7,9,10-tetrahydro-7,10-ethanocyclohepta[b]indol-8(5H)-one (4i)**
Indolyl alcohol **1i** (20 mg, 0.1 mmol), diene **2a** (87 mg, 0.41 mmol, 4 equiv.), and IDPi **6h** (3.2 mg, 2 mol%) were subjected to the general procedure (16 h) to afford **4i** as a white solid (25 mg, 91%).

$^1$H NMR (500 MHz, $CD_2Cl_2$) δ 8.06 (s, 1H), 7.42 (d, *J* = 8.5 Hz, 1H), 7.32 (d, *J* = 1.9 Hz, 1H), 7.06 (dd, *J* = 8.4, 1.9 Hz, 1H), 3.52 (dp, *J* = 4.7, 2.4 Hz, 1H), 2.88 – 2.64 (m, 1H), 2.54 (dt, *J* = 18.4, 2.5 Hz, 1H), 2.39 (d, *J* = 1.9 Hz, 1H), 2.35 – 2.26 (m, 1H), 2.16 – 1.93 (m, 3H), 1.46 (s, 3H), 1.39 (s, 3H).
$^{13}$C NMR (125 MHz, $CD_2Cl_2$) δ 214.2, 140.6, 136.2, 127.4, 125.5, 120.1, 118.9, 117.1, 110.7, 58.8, 47.5, 38.8, 30.3, 28.5, 28.1, 26.9, 20.6.
EI-HRMS: calculated for $C_{17}H_{18}N_1O_1Cl_1$ ($[M]^+$): 287.107142, found: 287.107040.
HPLC (AD-3, *n*-heptane/*i*-PrOH=94:6, 1.2 mL/min, 298 K, 282 nm): $t_R$(minor) = 9.17 min, $t_R$(major) = 10.92 min. e.r. = 94:6 (88% ee).
$[\alpha]_D^{25}$ = +15 (c = 0.55, $CH_2Cl_2$).
IR (film): 3357, 2925, 1704, 1464, 933, 736.

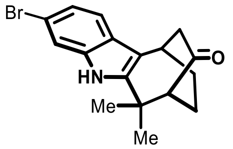


**(7*R*,10*R*)-3-bromo-6,6-dimethyl-6,7,9,10-tetrahydro-7,10-ethanocyclohepta[b]indol-8(5H)-one (4j)**
Indolyl alcohol **1j** (19 mg, 0.075 mmol), diene **2a** (66 mg, 0.31 mmol, 4.2 equiv.), and IDPi **6i** (2.87 mg, 2 mol%) were subjected to the general procedure (16 h) to afford **4j** as a colorless oil (23 mg, 93%).
$^1$H NMR (500 MHz, $CD_3CN$) δ 9.26 (s, 1H), 7.45 (d, *J* = 1.8 Hz, 1H), 7.37 (d, *J* = 8.4 Hz, 1H), 7.13 (dd, *J* = 8.4, 1.8 Hz, 1H), 3.49 (dt, *J* = 4.6, 2.3 Hz, 1H), 2.63 (dd, *J* = 17.7, 4.4 Hz, 1H), 2.41 (dt, *J* = 18.3, 2.5 Hz, 1H), 2.32 – 2.29 (m, 1H), 2.26 (td, *J* = 10.2, 1.6 Hz, 1H), 2.09 – 2.01 (m, 2H), 1.91 – 1.81 (m, 1H), 1.41 (s, 3H), 1.32 (s, 3H).
$^{13}$C NMR (125 MHz, $CD_3CN$) δ 214.6, 141.9, 137.4, 126.3, 122.7, 119.9, 117.3, 114.7, 114.2, 59.2, 47.7, 39.3, 30.1, 28.4, 28.2, 27.2, 20.7.
EI-HRMS: calculated for $C_{17}H_{18}N_1O_1Br_1$ ($[M]^+$): 331.056639, found: 331.056860.
HPLC (AD-3, *n*-heptane/*i*-PrOH=94:6, 1.2 mL/min, 298 K, 282 nm): $t_R$(minor) = 9.81 min, $t_R$(major) = 11.77 min. e.r. = 92:8 (84% ee).
$[\alpha]_D^{25}$ = +18 (c = 0.4 $CH_2Cl_2$).
IR (film): 3355, 2899, 1702, 1463, 737.

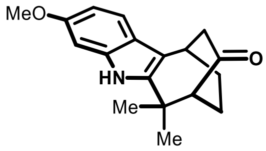


**(7R,10R)-3-methoxy-6,6-dimethyl-6,7,9,10-tetrahydro-7,10-ethanocyclohepta[b]indol-8(5H)-one (4k)**
Indolyl alcohol **1k** (20 mg, 0.097 mmol), diene **2a** (82 mg, 0.39 mmol, 4 equiv.), and IDPi **6h** (3.2 mg, 2 mol%) were subjected to the general procedure (16 h) to afford **4k** as a white solid (27 mg, 98%).
$^1$H NMR (500 MHz, $CD_2Cl_2$) δ 7.79 (s, 1H), 7.35 (d, *J* = 8.6 Hz, 1H), 6.83 (d, *J* = 2.3 Hz, 1H), 6.74 (dd, *J* = 8.6, 2.2 Hz, 1H), 3.83 (s, 3H), 3.49 (dp, *J* = 4.6, 2.3 Hz, 1H), 2.66 (dd, *J* = 18.3, 4.4 Hz, 1H), 2.53 (dt, *J* = 18.3, 2.5 Hz, 1H), 2.39 – 2.34 (m, 1H), 2.33 – 2.24 (m, 1H), 2.12 – 1.94 (m, 3H), 1.44 (s, 3H), 1.37 (s, 3H).
$^{13}$C NMR (125 MHz, $CD_2Cl_2$) δ 214.4, 156.7, 138.1, 136.5, 121.2, 118.5, 116.8, 109.2, 94.7, 59.0,

56.0, 47.6, 38.8, 30.4, 28.6, 28.2, 27.0, 20.6.
ESI-HRMS: calculated for $C_{18}H_{21}N_1O_2Na_1$ ([M+Na]$^+$): 306.146448, found: 306.146360.
HPLC (IB-3, *n*-heptane/*i*-PrOH=94:6, 1.0 mL/min, 298 K, 280 nm): $t_R$(minor) = 12.24 min, $t_R$(major) = 13.70 min. e.r. = 94:6 (88% ee).
$[\alpha]_D^{25}$ = +12 (c = 0.23 $CH_2Cl_2$).
IR (film): 3368, 2925, 1704, 1463, 803.

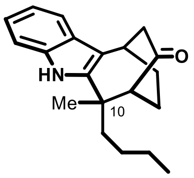


**(6S,7R,10R)-6-butyl-6-methyl-6,7,9,10-tetrahydro-7,10-ethanocyclohepta[b]indol-8(5H)-one (4l)**
Indolyl alcohol **1l** (20 mg, 0.077 mmol), diene **2a** (65 mg, 0.31 mmol, 4 equiv.), and IDPi **6h** (2.6 mg, 2 mol%) were subjected to the general procedure (3 d, dr = 8:1 as determined by GC analysis of the crude reaction mixture) to afford **4l** as a white solid (19.5 mg, 85%).
$^1$H NMR (600 MHz, $CDCl_3$) δ 7.75 (s, 1H), 7.49 (ddt, *J* = 7.9, 1.4, 0.7 Hz, 1H), 7.29 (ddd, *J* = 8.0, 0.8 Hz, 1H), 7.14 (ddd, *J* = 8.0, 7.1, 1.3 Hz, 1H), 7.09 (ddd, *J* = 7.8, 7.1, 1.1 Hz, 1H), 3.59 – 3.54 (m, 1H), 2.66 (ddd, *J* = 18.2, 4.1, 0.6 Hz, 1H), 2.57 (dt, *J* = 18.3, 2.5 Hz, 1H), 2.55 (dd, *J* = 5.3, 1.7 Hz, 1H), 2.27 – 2.20 (m, 1H), 2.15 – 2.07 (m, 1H), 2.06 – 1.97 (m, 2H), 1.83 (td, *J* = 13.8, 12.2, 4.2 Hz, 1H), 1.69 (ddd, *J* = 13.8, 11.9, 4.3 Hz, 1H), 1.45 – 1.37 (m, 1H), 1.37 (s, 3H), 1.38 – 1.27 (m, 2H), 0.94 (t, *J* = 7.1 Hz, 3H).
$^{13}$C NMR (150 MHz, $CDCl_3$) δ 214.9, 139.9, 135.3, 126.3, 121.7, 119.5, 117.7, 117.2, 110.6, 54.8, 47.2, 41.1, 40.5, 28.0, 26.6, 26.3, 26.1, 23.4, 19.8, 14.2.
EI-HRMS: calculated for $C_{20}H_{25}N_1O_1$ ([M]$^+$): 295.193064, found: 295.193430.
HPLC (IB-3, *n*-heptane/*i*-PrOH=94:6, 1.0 mL/min, 298 K, 282 nm): $t_R$(minor) = 7.42 min, $t_R$(major) = 10.47 min. e.r. = 94:6 (88% ee).
$[\alpha]_D^{25}$ = +21 (c = 0.41 $CH_2Cl_2$).
IR (film): 3375, 2932, 1699, 1464, 746.

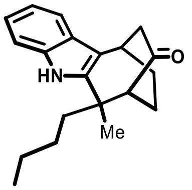


**(6S,7R,10R)-6-butyl-6-methyl-6,7,9,10-tetrahydro-7,10-ethanocyclohepta[b]indol-8(5H)-one (4l')**
**4l'** (2.5 mg, 10%, colorless oil)
$^1$H NMR (501 MHz, $CD_2Cl_2$) δ 7.93 (s, 1H), 7.51 (dd, *J* = 7.9, 1.2 Hz, 1H), 7.32 (dt, *J* = 8.0, 1.0 Hz, 1H), 7.13 (ddd, *J* = 8.1, 7.1, 1.3 Hz, 1H), 7.08 (ddd, *J* = 8.1, 7.0, 1.1 Hz, 1H), 3.56 (tt, *J* = 5.6, 2.8 Hz, 1H), 2.67 (ddd, *J* = 18.6, 4.8, 0.9 Hz, 1H), 2.62 – 2.53 (m, 2H), 2.29 – 2.16 (m, 1H), 2.13 – 2.03 (m, 1H), 1.98 (dddd, *J* = 10.8, 5.7, 3.3, 1.7 Hz, 2H), 1.72 – 1.57 (m, 3H), 1.39 – 1.24 (m, 3H), 0.96 – 0.90 (m, 3H).
$^{13}$C NMR (126 MHz, $CD_2Cl_2$) δ 214.9, 140.2, 135.8, 126.8, 121.7, 119.5, 117.9, 116.4, 110.7, 55.7, 48.1, 43.1, 28.1, 27.4, 26.3, 25.8, 25.2, 23.7, 20.8, 14.2.
EI-HRMS: calculated for $C_{20}H_{25}N_1O_1$ ([M]$^+$): 295.193064, found: 295.193190.

HPLC (IJ-3R, acetonitrile/water=70:30, 1.0 mL/min, 298 K, 301 nm): $t_R$(major) = 3.96 min, $t_R$(minor) = 4.92 min. e.r. = 73:27 (46% ee).
$[\alpha]_D^{25}$ = +1.1 (c = 0.18 $CH_2Cl_2$).
IR (film): 3389, 2932, 1695, 1463, 1260,749.

A hypothesis sheds the light of the moderate enantioselectivity of minor diastereoisomers. The *N–H* bonding plays a crucial role in the interaction between catalyst and substrate. This interaction positions the indolium cation within the chiral anionic pocket. The favored transition state with the large substituent pointing outside of the pocket leads to the desired product. In the unfavored transition state the large substituent points inward and the resulting steric repulsion from the catalyst will give rise to the minor isomer. Presumably, this improper positioning in the (4+3) cycloaddition leads to a moderate er (as shown in fig. 2).

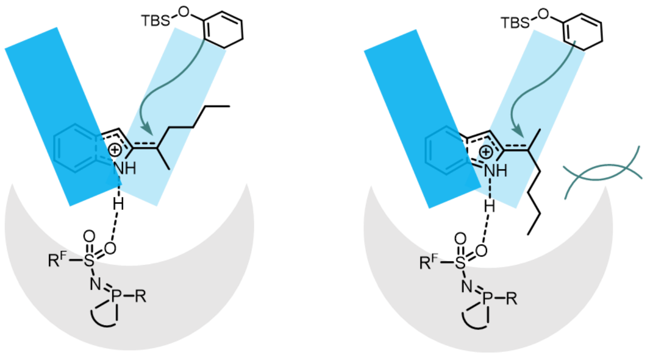


Figure 2. A hypothesis for the moderate enantioselectivity of minor diastereoisomers

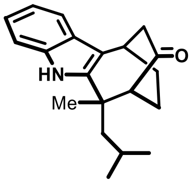


**(6*S*,7*R*,10*R*)-6-isobutyl-6-methyl-6,7,9,10-tetrahydro-7,10-ethanocyclohepta[b]indol-8(5H)-one (4m)**
Indolyl alcohol **1m** (20 mg, 0.092 mmol), diene **2a** (77 mg, 0.36 mmol, 4 equiv.), and IDPi **6h** (3.0 mg, 2 mol%) were subjected to the general procedure (3 d, dr = 4.9:1as determined by GC analysis of the crude reaction mixture) to afford **4m** as a white solid (18 mg, 78%).
$^1$H NMR (501 MHz, $CD_2Cl_2$) δ 7.94 (s, 1H), 7.47 (d, *J* = 7.7 Hz, 1H), 7.27 (d, *J* = 7.9 Hz, 1H), 7.13 – 6.98 (m, 1H), 3.60 – 3.52 (m, 1H), 2.68 – 2.60 (m, 2H), 2.50 (dt, *J* = 18.2, 2.5 Hz, 1H), 2.30 – 2.20 (m, 1H), 2.14 – 1.95 (m, 2H), 1.89 – 1.80 (m, 1H), 1.71 – 1.60 (m, 1H), 1.39 (s, 3H), 1.04 (d, *J* = 6.4 Hz, 3H), 0.98 (d, *J* = 6.5 Hz, 3H), 0.92 (ddt, *J* = 11.8, 5.3, 2.5 Hz, 1H).
$^{13}$C NMR (126 MHz, $CD_2Cl_2$) δ 214.6, 140.5, 135.8, 126.7, 121.8, 119.5, 117.9, 116.9, 110.7, 55.2, 49.2, 47.7, 42.2, 28.3, 27.6, 26.8, 25.5 (d, *J* = 7.9 Hz), 24.4, 20.6.
EI-HRMS: calculated for $C_{20}H_{25}N_1O_1$ ($[M]^+$): 295.193064, found: 295.193210.
HPLC (IB-3, *n*-heptane/*i*-PrOH=95:5, 1.0 mL/min, 298 K, 267 nm): $t_R$(minor) = 6.51 min, $t_R$(major) = 8.00 min. e.r. = 92:8 (84% ee).
$[\alpha]_D^{25}$ = +14 (c = 0.8 $CH_2Cl_2$).

IR (film): 3370, 2953, 1697, 1463, 741.

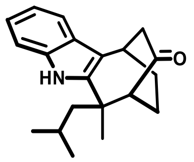

**(6*R*,7*R*,10*R*)-6-isobutyl-6-methyl-6,7,9,10-tetrahydro-7,10-ethanocyclohepta[b]indol-8(5H)-one (4m')**

**4m'** (3.3 mg, 14% a colorless oil).

$^{1}$H NMR (600 MHz, $CD_2Cl_2$) δ 7.47 (d, *J* = 7.8 Hz, 1H), 7.29 (ddd, *J* = 8.0, 0.8 Hz, 1H), 7.10 (ddd, *J* = 8.0, 7.1, 1.3 Hz, 1H), 7.05 (ddd, *J* = 7.8, 7.1, 1.1 Hz, 1H), 3.52 (tt, *J* = 4.7, 2.5 Hz, 1H), 2.64 (ddd, *J* = 18.5, 5.0, 1.0 Hz, 3H), 2.63 (ddd, *J* = 6.9, 1.8, 1.0 Hz, 1H), 2.56 (dt, *J* = 18.6, 2.3 Hz, 2H), 2.20 (dddd, *J* = 14.5, 10.0, 5.7, 1.4 Hz, 2H), 2.04 (dddd, *J* = 14.5, 9.8, 6.9, 4.8 Hz, 1H), 1.60 (dd, *J* = 14.4, 3.9 Hz, 2H), 1.55 (dd, *J* = 14.4, 6.6 Hz, 2H), 0.93 (d, *J* = 6.6 Hz, 3H), 0.92 (d, *J* = 6.7 Hz, 3H).

$^{13}$C NMR (150 MHz, $CD_2Cl_2$) δ 215.0, 140.2, 135.7, 126.8, 121.7, 119.5, 117.9, 116.5, 110.7, 56.0, 51.5, 48.0, 28.2, 27.4, 26.0, 25.3, 25.2, 24.5, 20.9, 1.2.

EI-HRMS: calculated for $C_{20}H_{25}N_1O_1$ ($[M]^+$): 295.193064, found: 295.193770.

HPLC (IB-3, *n*-heptane/*i*-PrOH=94:6, 1.0 mL/min, 298 K, 282 nm): $t_R$(major) = 8.67 min, $t_R$(minor) = 9.35 min. e.r. = 77:23 (54% ee).

$[\alpha]_D^{25}$ = +6 (c = 0.1 $CH_2Cl_2$).

IR (film): 3375, 2925, 1695, 1464, 747.

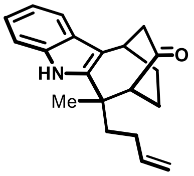

**(6*S*,7*R*,10*R*)-6-(but-3-en-1-yl)-6-methyl-6,7,9,10-tetrahydro-7,10-ethanocyclohepta[b]indol-8(5H)-one (4n)**

Indolyl alcohol **1n** (20 mg, 0.077 mmol), diene **2a** (65 mg, 0.31 mmol, 4 equiv.), and IDPi **6h** (2.6 mg, 2 mol%) were subjected to the general procedure procedure (3 d, dr = 8:1 as determined by GC analysis of the crude reaction mixture) to afford **4n** as a white solid (19 mg, 83%).

$^{1}$H NMR (501 MHz, $CD_2Cl_2$) δ 7.89 (s, 1H), 7.47 (dd, *J* = 7.7, 1.2 Hz, 1H), 7.31 – 7.23 (m, 1H), 7.10 (ddd, *J* = 8.1, 7.0, 1.3 Hz, 1H), 7.05 (td, *J* = 7.4, 1.2 Hz, 1H), 5.86 (ddt, *J* = 16.8, 10.2, 6.5 Hz, 1H), 5.08 (dq, *J* = 17.1, 1.7 Hz, 1H), 4.98 (dq, *J* = 10.1, 1.4 Hz, 1H), 3.56 (ddt, *J* = 5.5, 4.2, 2.1 Hz, 1H), 2.66 (dd, *J* = 18.1, 3.9 Hz, 1H), 2.55 – 2.46 (m, 2H), 2.30 – 2.16 (m, 2H), 2.16 – 2.09 (m, 2H), 2.07 – 1.97 (m, 2H), 1.91 (ddd, *J* = 13.9, 12.1, 4.6 Hz, 1H), 1.84 – 1.77 (m, 1H), 1.38 (s, 3H). $^{13}$C NMR (126 MHz, $CD_2Cl_2$) δ 214.1, 139.8, 138.6, 135.8, 126.6, 121.9, 119.5, 117.9, 115.0, 110.8, 55.0, 47.3, 41.3, 40.1, 28.6, 28.3, 26.6, 26.5, 20.1.

EI-HRMS: calculated for $C_{20}H_{23}N_1O_1$ ($[M]^+$): 293.177414, found: 293.177690.

HPLC (IB-3, *n*-heptane/*i*-PrOH=94:6, 1.0 mL/min, 298 K, 282 nm): $t_R$(minor) = 10.09 min, $t_R$(major) = 11.98 min. e.r. = 97:3 (94% ee).

$[\alpha]_D^{25}$ = +22 (c = 0.28 $CH_2Cl_2$).

IR (film): 3370, 2935, 1697, 1463, 744

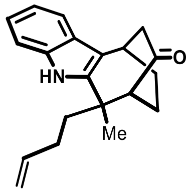

**(6*R*,7*R*,10*R*)-6-allyl-6-methyl-6,7,9,10-tetrahydro-7,10-ethanocyclohepta[b]indol-8(5H)-one (4n')**

**4n'** (2.5 mg, 11%, colorless oil)

$^{1}$H NMR (600 MHz, $CD_2Cl_2$) δ 7.93 (s, 1H), 7.51 (dtd, *J* = 7.9, 0.9, 0.5 Hz, 1H), 7.32 (ddd, *J* = 8.0, 0.8 Hz, 1H), 7.13 (ddd, *J* = 8.0, 7.1, 1.3 Hz, 1H), 7.08 (ddd, *J* = 7.8, 7.1, 1.1 Hz, 1H), 5.82 (dddd, *J* = 17.0, 10.2, 6.7, 6.3 Hz, 1H), 5.05 (ddt, *J* = 17.1, 2.0, 1.6 Hz, 1H), 4.96 (ddt, *J* = 10.2, 2.0, 1.2 Hz, 1H), 3.61 – 3.51 (m, 1H), 2.67 (ddd, *J* = 18.6, 4.8, 0.9 Hz, 1H), 2.64 – 2.56 (m, 2H), 2.43 – 2.34 (m, 1H), 2.26 – 2.18 (m, 1H), 2.17 – 2.07 (m, 2H), 2.01 – 1.96 (m, 2H), 1.76 (ddd, *J* = 13.9, 12.1, 4.7 Hz, 1H), 1.69 (ddd, *J* = 13.9, 12.2, 4.7 Hz, 1H), 1.46 (d, *J* = 0.5 Hz, 3H).

$^{13}$C NMR (150 MHz, $CD_2Cl_2$) δ 214.8, 139.7, 138.9, 135.8, 126.8, 121.8, 119.5, 118.0, 116.6, 114.7, 110.8, 55.5, 48.1, 42.4, 28.5, 28.1, 27.3, 25.2, 20.

EI-HRMS: calculated for $C_{20}H_{23}N_1O_1$ ($[M]^+$): 293.177414, found: 293.177760.

HPLC (IB-3, *n*-heptane/*i*-PrOH=94:6, 1.0 mL/min, 298 K, 282 nm): $t_R$(major) = 10.47 min, $t_R$(minor) = 11.64 min. e.r. = 77:23 (54% ee).

$[\alpha]_D^{25}$ = +2 (c = 0.1 $CH_2Cl_2$).

IR (film): 3374, 2926, 1694, 1264, 734.

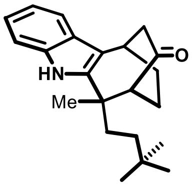

**(6*S*,7*R*,10*R*)-6-(3,3-dimethylbutyl)-6-methyl-6,7,9,10-tetrahydro-7,10-ethanocyclohepta[b]indol-8(5H)-one (4o)**

Indolyl alcohol **1o** (20 mg, 0.082 mmol), diene **2a** (68 mg, 0.32 mmol, 4 equiv.), and IDPi **6h** (3.3 mg, 2.5 mol%) were subjected to the general procedure (3 d, dr = 4.6:1 as determined by GC analysis of the crude reaction mixture) to afford **4o** as a white solid (21 mg, 80%).

$^{1}$H NMR (501 MHz, $CD_2Cl_2$) δ 7.95 (s, 1H), 7.47 (dd, *J* = 7.8, 1.3 Hz, 1H), 7.28 (dt, *J* = 8.0, 1.0 Hz, 1H), 7.09 (ddd, *J* = 8.0, 7.0, 1.3 Hz, 1H), 7.05 (ddd, *J* = 8.1, 7.1, 1.2 Hz, 1H), 3.56 (ddq, *J* = 7.0, 4.1, 1.8 Hz, 1H), 2.65 (dd, *J* = 18.1, 4.0 Hz, 1H), 2.56 – 2.47 (m, 2H), 2.29 – 2.17 (m, 1H), 2.17 – 2.09 (m, 1H), 2.04 – 1.93 (m, 2H), 1.83 (td, *J* = 13.3, 4.4 Hz, 1H), 1.69 (td, *J* = 13.3, 4.5 Hz, 1H), 1.38 – 1.29 (m, 4H), 1.28 – 1.18 (m, 1H), 0.93 (s, 9H).

$^{13}$C NMR (126 MHz, $CD_2Cl_2$) δ 214.5, 140.6, 135.7, 126.6, 121.8, 119.5, 117.9, 117.5, 110.7, 54.6, 47.4, 41.2, 37.6, 35.6, 30.5, 29.5, 28.3, 26.7, 26.4, 19.9.

EI-HRMS: calculated for $C_{22}H_{29}N_1O_1$ ($[M]^+$): 323.224363, found: 323.224960.

HPLC (OD-3, *n*-heptane/*i*-PrOH=95:5, 1.0 mL/min, 298 K, 282 nm): $t_R$(minor) = 6.37 min, $t_R$(major) = 14.95 min. e.r. = 95:5 (90% ee).

$[\alpha]_D^{25}$ = +15 (c = 0.5 $CH_2Cl_2$).

IR (film): 3369, 2952, 1703, 1261, 749.

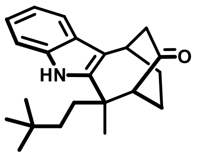

**(6*R*,7*R*,10*R*)-6-(3,3-dimethylbutyl)-6-methyl-6,7,9,10-tetrahydro-7,10-ethanocyclohepta[b]indol-8(5*H*)-one (4o')**

**4o'** (4.6 mg, 17%, colorless oil)

$^1$H NMR (501 MHz, $CD_2Cl_2$) δ 7.93 (s, 1H), 7.48 (d, *J* = 7.8 Hz, 1H), 7.30 (d, *J* = 7.9 Hz, 1H), 7.14 – 7.07 (m, 1H), 7.09 – 7.02 (m, 1H), 3.53 (dq, *J* = 5.6, 3.2 Hz, 1H), 2.68 – 2.51 (m, 3H), 2.23 – 2.12 (m, 1H), 2.08 – 2.00 (m, 1H), 1.95 (ddd, *J* = 8.0, 5.6, 3.1 Hz, 2H), 1.70 – 1.47 (m, 3H), 1.39 (s, 3H), 1.24 – 1.14 (m, 1H), 0.87 (s, 9H).

$^{13}$C NMR (126 MHz, $CD_2Cl_2$) δ 214.6, 140.5, 135.8, 126.8, 121.7, 119.5, 117.9, 116.4, 110.7, 55.2, 48.1, 41.8, 37.9, 37.2, 29.4, 28.2, 27.4, 25.1, 20.8, 1.2.

EI-HRMS: calculated for $C_{22}H_{29}N_1O_1$ ($[M]^+$): 323.224364, found: 323.224770.

HPLC (IC-3, *n*-heptane/*i*-PrOH=99:1, 1.0 mL/min, 298 K, 282 nm): $t_R$(major) = 8.71 min, $t_R$(minor) = 10.52 min. e.r. = 85:15 (70% ee).

$[\alpha]_D^{25}$ = +6 (c = 0.1 $CH_2Cl_2$).

IR (film): 3387, 2951, 1695, 1463, 739.

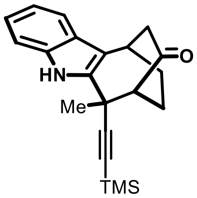

**(6*S*,7*R*,10*R*)-6-methyl-6-((trimethylsilyl)ethynyl)-6,7,9,10-tetrahydro-7,10-ethanocyclohepta[b]indol-8(5*H*)-one (4p)**

Indolyl alcohol **1p** (20 mg, 0.078 mmol), diene **2a** (65 mg, 0.31 mmol, 4 equiv.), and IDPi **6h** (3.2 mg, 2.5 mol%) were subjected to the general procedure (3 d, dr = 2.6:1 as determined by GC analysis of the crude reaction mixture) to afford **4p** as a white solid (19 mg, 72%).

$^1$H NMR (501 MHz, $CD_2Cl_2$) δ 8.13 (s, 1H), 7.50 (dq, *J* = 7.8, 0.9 Hz, 1H), 7.34 (dt, *J* = 8.2, 0.9 Hz, 1H), 7.15 (ddd, *J* = 8.2, 7.1, 1.3 Hz, 1H), 7.08 (ddd, *J* = 8.1, 7.1, 1.0 Hz, 1H), 3.62 – 3.44 (m, 1H), 2.70 – 2.51 (m, 4H), 2.20 – 2.10 (m, 1H), 2.07 – 1.99 (m, 2H), 0.91 (s, 3H), 0.16 (s, 9H).

$^{13}$C NMR (126 MHz, $CD_2Cl_2$) δ 213.9, 137.2, 136.0, 127.7, 123.4, 120.8, 119.3, 118.5, 112.1, 109.8, 88.8, 58.3, 49.2, 42.0, 31.2, 29.2, 28.0, 26.8, 23.1, 1.1.

EI-HRMS: calculated for $C_{21}H_{25}N_1O_1Si_1$ ($[M]^+$): 335.169992, found 335.170130.

HPLC (IB-3, *n*-heptane/*i*-PrOH=94:6, 0.8 mL/min, 298 K, 280 nm): $t_R$(minor) = 5.64 min, $t_R$(major) = 9.64 min. e.r. = 95.5:4.5 (91% ee).

$[\alpha]_D^{25}$ = +45 (c = 0.35 $CH_2Cl_2$).

IR (film): 3393, 2954, 2162, 1706, 1462, 842.

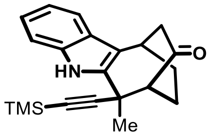

**(6*R*,7*R*,10*R*)-6-methyl-6-((trimethylsilyl)ethynyl)-6,7,9,10-tetrahydro-7,10-**

**ethanocyclohepta[b]indol-8(5H)-one (4p')**

**4p'** (7 mg, 27%, white solid)

$^1$H NMR (501 MHz, $CD_2Cl_2$) δ 8.11 (s, 1H), 7.50 (dd, *J* = 7.9, 1.1 Hz, 1H), 7.34 (dt, *J* = 8.2, 0.9 Hz, 1H), 7.15 (ddd, *J* = 8.2, 7.1, 1.3 Hz, 1H), 7.08 (ddd, *J* = 8.0, 7.1, 1.1 Hz, 1H), 3.55 (dp, *J* = 4.8, 2.4 Hz, 1H), 2.71 – 2.62 (m, 2H), 2.61 – 2.50 (m, 1H), 2.24 – 2.15 (m, 1H), 2.14 – 2.00 (m, 2H), 1.94 (dddt, *J* = 13.1, 7.3, 4.9, 2.8 Hz, 1H), 1.70 (s, 3H), 0.13 (s, 8H).

$^{13}$C NMR (126 MHz, $CD_2Cl_2$) δ 212.1, 137.1, 135.9, 127.7, 123.4, 120.8, 119.3, 118.9, 112.1, 110.0, 89.7, 58.7, 48.1, 39.9, 29.4, 29.0, 28.0, 21.3, 1.0.

EI-HRMS: calculated for $C_{21}H_{25}N_1O_1Si_1$ ($[M]^+$): 335.169992, found: 335.170080.

HPLC (OD-3, *n*-heptane/*i*-PrOH=95:5, 1.0 mL/min, 298 K, 282 nm): $t_R$(minor) = 4.13 min, $t_R$(major) = 8.11 min. e.r. = 71:29 (42% ee).

$[\alpha]_D^{25}$ = +9 (c = 0.35 $CH_2Cl_2$).

IR (film): 3452, 2958, 2163, 1713, 1264, 733.

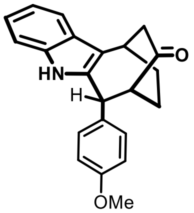


**(6S,7R,10R)-6-(4-methoxyphenyl)-6,7,9,10-tetrahydro-7,10-ethanocyclohepta[b]indol-8(5H)-one (4q)**

Indolyl alcohol **1q** (20 mg, 0.079 mmol), diene **2a** (66 mg, 0.31 mmol, 4 equiv.), and IDPi **6h** (2.6 mg, 2 mol%) were subjected to the general procedure (3 d, dr = 4.9:1 as determined by GC analysis of the crude reaction mixture) to afford **4q** as a white solid (14 mg, 54%).

$^1$H NMR (501 MHz, $CD_2Cl_2$) δ 7.77 (s, 1H), 7.56 – 7.48 (m, 1H), 7.23 – 7.18 (m, 1H), 7.10 (pd, *J* = 7.1, 1.4 Hz, 2H), 7.04 (dd, *J* = 9.0, 2.7 Hz, 2H), 6.89 – 6.83 (m, 2H), 4.71 (d, *J* = 4.7 Hz, 1H), 3.78 (s, 3H), 3.66 (dtd, *J* = 5.3, 3.4, 1.6 Hz, 1H), 2.76 (td, *J* = 5.2, 1.8 Hz, 1H), 2.72 (dd, *J* = 17.5, 3.6 Hz, 1H), 2.63 (dt, *J* = 17.4, 2.8 Hz, 1H), 2.26 – 2.16 (m, 1H), 2.15 – 2.07 (m, 1H), 1.93 – 1.77 (m, 2H).

$^{13}$C NMR (126 MHz, $CD_2Cl_2$) δ 213.9, 159.3, 136.4, 133.1, 132.9, 130.0, 129.9, 126.8, 122.2, 119.6, 118.0, 114.4, 110.9, 55.7, 53.7, 47.6, 46.9, 29.3, 26.4, 18.0.

ESI-HRMS: calculated for $C_{22}H_{21}N_1O_1Na_1$ ($[M+Na]^+$): 354.146448, found: 354.146950.

HPLC (IB-3, *n*-heptane/*i*-PrOH=94:6, 1.0 mL/min, 298 K, 282 nm): $t_R$(major) = 13.56 min, $t_R$(minor) = 17.74 min. e.r. = 85:15 (70% ee).

$[\alpha]_D^{25}$ = –7 (c = 0.27 $CH_2Cl_2$).

IR (film): 3384, 2926, 1711, 1509, 1241, 742.

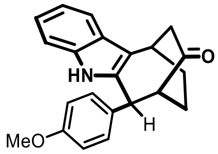


**(6S,7R,10R)-6-(4-methoxyphenyl)-6,7,9,10-tetrahydro-7,10-ethanocyclohepta[b]indol-8(5H)-one (4q')**

**4q'** (2.8 mg, 11%, white solid)

$^{1}$H NMR (501 MHz, $CD_2Cl_2$) δ 7.69 (s, 1H), 7.54 (dd, *J* = 7.0, 1.9 Hz, 1H), 7.23 – 7.18 (m, 1H), 7.12 – 7.05 (m, 2H), 6.91 (d, *J* = 8.7 Hz, 1H), 6.80 (d, *J* = 8.7 Hz, 2H), 4.47 (d, *J* = 4.4 Hz, 1H), 3.76 (s, 3H), 3.66 – 3.62 (m, 1H), 2.83 (dd, *J* = 7.3, 4.4 Hz, 1H), 2.69 – 2.59 (m, 2H), 2.37 – 2.26 (m, 1H), 2.23 – 2.12 (m, 2H), 2.09 – 2.02 (m, 1H).
ESI-HRMS: calculated for C22H21N1O1Na1 ([M+Na]+): 354.146448, found: 354.146950.
HPLC (IB-3, *n*-heptane/*i*-PrOH=94:6, 1.0 mL/min, 298 K, 282 nm): $t_R$(minor) = 18.68 min, $t_R$(major) = 21.30 min. e.r. = 66:34 (32% ee).
$[\alpha]_D^{25}$ = +2 (c = 0.1 $CH_2Cl_2$).
IR (film): 3361, 2925, 1710, 1461, 1260, 748.

## 5. Derivatization of Ketone 4a

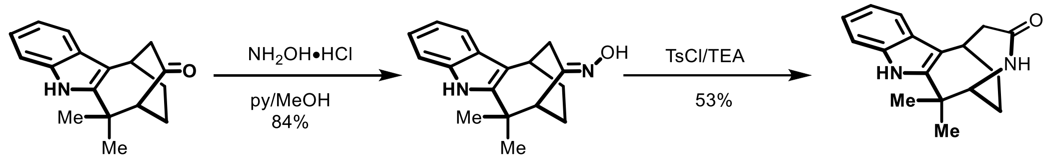


4a 8 9

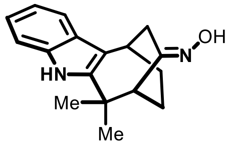


**(7R,10R,E)-6,6-dimethyl-6,7,9,10-tetrahydro-7,10-ethanocyclohepta[b]indol-8(5H)-one oxime (8)**

To a solution of ketone **4a** (12.3 mg, 0.048 mmol) in pyridine/MeOH (0.4 mL, 1:1 *v/v*) was added hydroxylamine hydrochloride (33 mg, 10 equiv.) at 25 °C. The resulting solution was stirred for 2 days until all ketone was consumed (monitored by TLC), then concentrated under vacuum to remove the excess pyridine. The crude mixture was purified by silica-gel chromatography (hexane: ethyl acetate = 1:4) to afford a white solid **8** (11 mg, 84%.). This compound was treated as an intermediate.
$^{1}$H NMR (501 MHz, $CD_2Cl_2$) δ 7.89 (s, 1H), 7.54 – 7.41 (m, 1H), 7.26 (dd, *J* = 7.6, 1.3 Hz, 1H), 7.11 – 7.00 (m, 2H), 3.39 (q, *J* = 2.8, 2.4 Hz, 1H), 2.84 (dd, *J* = 18.6, 5.1 Hz, 1H), 2.65 (dt, *J* = 18.6, 2.2 Hz, 1H), 2.51 – 2.39 (m, 1H), 2.22 – 2.11 (m, 1H), 1.90 (dddd, *J* = 16.3, 9.8, 7.8, 4.9 Hz, 3H), 1.43 (s, 3H), 1.29 (s, 3H).
$^{13}$C NMR (126 MHz, $CD_2Cl_2$) δ 162.7, 140.3, 135.9, 127.1, 121.4, 119.4, 117.9, 116.2, 110.6, 48.4, 39.9, 34.1, 30.7, 28.6, 28.4, 26.2, 21.6.
EI-HRMS: calculated for $C_{17}H_{20}N_2O_1$ ($[M]^+$): 268.157012, found: 268.157120.

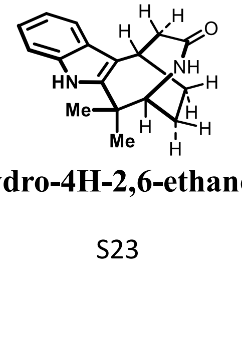


**(6R)-1,1-dimethyl-1,2,3,5,6,11-hexahydro-4H-2,6-ethanoazocino[4,5-b]indol-4-one (9)**

Yield, 54%.
A flame-dried vial was charged with oxime **8** (7.8 mg, 0.029 mmol), triethylamine (0.0081 mL, 0.058 mmol, 2 equiv.) and dry DCM (0.5 mL), following the addition of 4-toluenesulfonyl chloride (5.5 mg, 0.029 mmol, 1 equiv.). The solution was stirred for 1 h until the starting materials was fully consumed (monitored by TLC). The crude mixture was directly subjected to silica gel chromatography to afford a colorless oil **9** (4.2 mg, 54%).
$^1$H NMR (600 MHz, $CD_2Cl_2$) δ 7.44 (dq, *J* = 7.9, 1.0 Hz, 1H), 7.31 (dt, *J* = 8.0, 1.1, 0.7 Hz, 1H), 7.11 (ddd, *J* = 8.0, 7.1, 1.2 Hz, 1H), 7.06 (ddd, *J* = 7.9, 7.1, 1.1 Hz, 1H), 6.30 (d, *J* = 6.7 Hz, 1H), 3.51 (tddd, *J* = 6.0, 2.5, 2.0, 0.7 Hz, 2H), 3.22 (td, *J* = 6.7, 6.0, 2.3 Hz, 1H), 2.87 (dd, *J* = 17.5, 2.4 Hz, 1H), 2.82 (dddd, *J* = 17.5, 6.0, 1.6, 1.1 Hz, 5H), 2.47 (ddddt, *J* = 15.5, 8.7, 6.0, 1.7, 0.9 Hz, 2H), 2.42 (dddd, *J* = 15.5, 11.5, 8.5, 2.2 Hz, 1H), 2.19 (dddd, *J* = 14.0, 8.5, 6.0, 1.6 Hz, 1H), 2.13 (dddt, *J* = 14.0, 11.5, 8.7, 1.6 Hz, 1H).
$^{13}$C NMR (151 MHz, $CD_2Cl_2$) δ 175.3, 139.5, 135.7, 127.8, 121.7, 119.6, 117.8, 114.9, 110.6, 61.8, 41.7, 41.1, 31.9, 28.9, 28.8, 28.7, 27.4.
EI-HRMS: calculated for $C_{17}H_{20}N_2O_1$ ([M]$^+$): 268.157012, found: 268.157110.
HPLC (IC-3, Acetonitrile/water=40:60, 0.8 mL/min, 298 K, 282 nm): $t_R$(minor) = 5.27 min, $t_R$(major) = 6.42 min. e.r. = 95:5 (90% ee).
$[\alpha]_D^{25}$ = +68 (c = 0.1 $CH_2Cl_2$).
IR (film): 3325, 3245, 1618, 1594, 729.

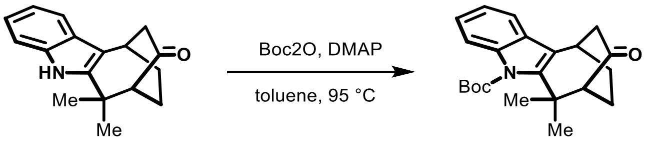


4a 10

**Tert-butyl(7R,10R)-6,6-dimethyl-8-oxo-7,8,9,10-tetrahydro-7,10-ethanocyclohepta[b]indole-5(6H)-carboxylate (10)**
To a flame-dried vial (10 mL) was charged with the ketone **4a** (35 mg, 0.14 mmol), DMAP (25 mg, 0.21mmol, 1.5 equiv.), di-*tert*-butyl dicarbonate (261 mg, 0.4 mL, 12 equiv.) and toluene (1 mL). The vial was sealed with a plastic cap and was heated to 95 °C for 1 h. After the ketone fully consumed (monitored by TLC), the crude mixture was loaded on a silica gel column (hexane: wash out the toluene and non-polar impurities, hexane:ethyl acetate 1:20) to afford a white solid **10** (38 mg, 78%). This compound was treated as an intermediate.
$^1$H NMR (501 MHz, $CD_2Cl_2$) δ 7.76 (d, *J* = 8.2 Hz, 1H), 7.48 – 7.41 (m, 1H), 7.29 – 7.21 (m, 1H), 7.20 – 7.15 (m, 1H), 3.55 (dd, *J* = 4.8, 2.7 Hz, 1H), 2.62 (dd, *J* = 18.3, 4.7 Hz, 1H), 2.51 (dt, *J* = 18.5, 1.8 Hz, 1H), 2.37 – 2.27 (m, 1H), 2.27 – 2.19 (m, 1H), 2.04 – 1.88 (m, 3H), 1.69 (s, 10H), 1.59 (s, 3H), 1.55 (s, 2H).
$^{13}$C NMR (126 MHz, $CD_2Cl_2$) δ 214.3, 151.8, 140.7, 136.9, 127.9, 124.2, 123.9, 122.2, 118.0, 114.4, 84.6, 62.6, 46.1, 41.2, 28.7, 28.3, 27.5, 26.8, 26.6, 20.9.
ESI-HRMS: calculated for $C_{22}H_{27}N_1O_3Na_1$ ([M+Na]$^+$): 376.188313, found: 376.188380.

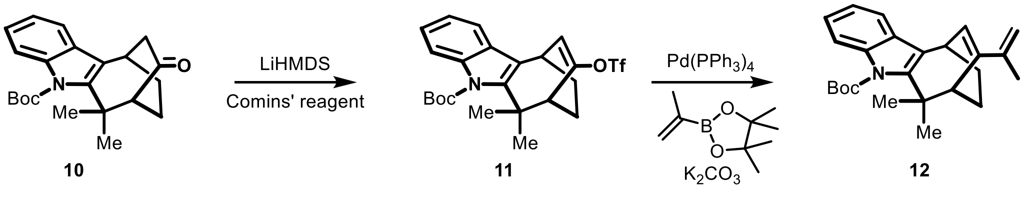


10 11 12

***Tert*-butyl(7R,10R)-6,6-dimethyl-8-(((trifluoromethyl)sulfonyl)oxy)-7,10-dihydro-7,10-ethanocyclohepta[b]indole-5(6H)-carboxylate (11)**

To a solution of ketone **8** in dry THF under Ar at –78 °C was added LiHMDS (1 M in THF, 0.22 mL, 0.23 mmol, 10 equiv.). The mixture was allowed to warm up to 25 °C for 30 min, and cooled to –78 °C, followed by the addition of Comins' reagent (16 mg, 0.04 mmol, 1.8 equiv.). The mixture was stirred for 15 min at –78 °C and 30 min at 25 °C until all ketone was consumed (monitored by TLC). The reaction was quenched with distilled $H_2O$, and extracted with ethyl ether (2x10 mL). The combined organic layer was collected, dried over anhydrous $MgSO_4$, and the solvent was removed under vacuum. The residue was purified by chromatography ($Et_2O$ :hexane= 1:10) to afford **11** (10.2 mg, 93%). This compound was treated as an intermediate.

$^1$H NMR (501 MHz, $CD_2Cl_2$) δ 7.78 – 7.69 (m, 1H), 7.47 (dd, *J* = 7.7, 1.6 Hz, 1H), 7.31 – 7.13 (m, 2H), 6.52 (dd, *J* = 8.4, 2.4 Hz, 1H), 3.87 (dt, *J* = 8.4, 3.1 Hz, 1H), 2.50 (dd, *J* = 6.9, 2.5 Hz, 1H), 2.18 (ddd, *J* = 13.9, 9.4, 6.7 Hz, 1H), 2.03 – 1.88 (m, 2H), 1.83 (dddd, *J* = 12.4, 10.7, 6.7, 3.6 Hz, 1H), 1.70 (s, 3H), 1.69 (s, 9H), 1.53 (d, *J* = 3.4 Hz, 3H).

$^{13}$C NMR (126 MHz, $CD_2Cl_2$) δ 151.6 (d, *J* = 8.1 Hz), 140.2, 136.0, 127.8, 124.9, 124.0, 122.2, 120.4, 117.8, 117.7, 114.7, 84.6, 53.0, 40.8, 29.9, 29.8, 28.3, 28.1, 25.8, 21.1.

ESI-HRMS: calculated for $C_{23}H_{27}N_1O_5S_1F_3$ ($[M+H]^+$): 486.155657, found: 486.155950.

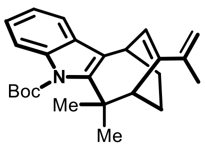


***Tert*-butyl(7*S*,10*R*)-6,6-dimethyl-8-(prop-1-en-2-yl)-7,10-dihydro-7,10-ethanocyclohepta[b]indole-5(6H)-carboxylate (12)**

To a flame-dried Schlenk flask was added **11** (10 mg, 0.02 mmol), (Tetrakis(triphenylphosphin)palladium(0)) (1.2 mg, 5 mol%), isopropenylboronic acid pinacol ester (13.8 mg, 0.082 mmol, 4 equiv.). The flask was vacuumed and backfilled with Ar for 3 times, then dry 1,4-dioxane (0.5 mL), and degassed $K_2CO_3$ (2 M aqueous, 0.5 mL) were added under Ar. The resulting mixture was heated to 80 °C for 2 h until all starting materials were consumed. To the cooled reaction was added distilled water (2 mL), and the mixture was extracted with ethyl ether (3x5 mL). The organic layers were combined, dried over anhydrous $MgSO_4$. The solvent was remove under vacuum, and the residue was purified by chromatography to afford a colorless oil **12** (7 mg, 90%).

$^1$H NMR (600 MHz, $CD_2Cl_2$) δ 7.72 – 7.68 (m, 1H), 7.51 – 7.46 (m, 1H), 7.21 – 7.15 (m, 1H), 7.18 – 7.13 (m, 1H), 6.61 (dd, *J* = 7.7, 1.9 Hz, 1H), 5.17 (p, *J* = 1.4, 0.7 Hz, 1H), 4.96 (p, *J* = 2.2, 1.4 Hz, 1H), 3.76 (ddd, *J* = 7.8, 4.1, 2.3 Hz, 1H), 2.89 (dt, *J* = 6.8, 1.5 Hz, 1H), 2.23 (dddd, *J* = 14.1, 9.9, 5.5, 1.1 Hz, 1H), 1.94 (dd, *J* = 1.3, 0.6 Hz, 3H), 1.90 (ddt, *J* = 12.3, 9.5, 2.7 Hz, 1H), 1.71 (dddd, *J* = 12.3, 11.2, 5.5, 4.1 Hz, 1H), 1.67 (s, 8H), 1.63 (dddd, *J* = 14.1, 11.2, 6.8, 2.7 Hz, 1H), 1.58 (s, 3H), 1.44 (s, 3H).

$^{13}$C NMR (151 MHz, $CD_2Cl_2$) δ 151.9, 143.9, 143.2, 141.2, 136.2, 131.7, 128.1, 123.4, 121.9, 120.1, 117.8, 114.5, 110.8, 84.1, 47.7, 41.4, 30.4, 29.8, 28.3, 27.2, 21.0, 20.8.

ESI-HRMS: calculated for $C_{25}H_{31}N_1O_2Na_1$ ($[M+Na]^+$): 400.224697, found: 400.225160.

GC: The enantiomeric ratio is measured by GC analysis on a chiral column (BGB 176 column: 25.0 m; i.D. 0.25 mm). Temperature: 350 °C (detector), 80 °C (oven, 3 min, iso) to 120 °C (50 °C/min, 15 min iso) to 220 °C (15 °C/min, 10 min iso); Gas: Helium (0.4 bar); tR = 15.39 min(minor), tR = 17.11 min (major), e.r. = 96:4

$[\alpha]_D^{25}$ = +110 (c = 0.33 $CH_2Cl_2$). IR (film): 2967, 2928, 1745, 1260, 749.

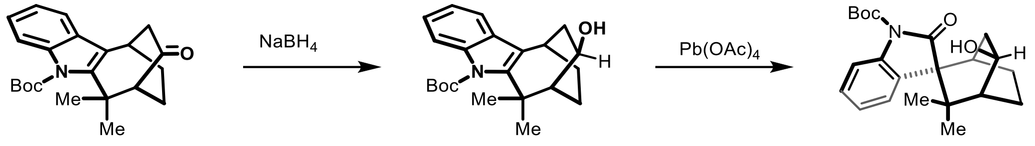



**_Tert_-butyl(7_R_,8_R_,10_R_)-8-hydroxy-6,6-dimethyl-7,8,9,10-tetrahydro-7,10-ethanocyclohepta[_b_]indole-5(6_H_)-carboxylate (13)**

To a solution of ketone **10** (21 mg, 0.059 mmol) in methanol (2 mL) was added sodium borohydride (6.7 mg, 0.18 mmol, 3 equiv.) at 0 °C. The reaction was stirred for 1 h until all ketone was consumed, following by the addition of acetone. The volatiles were removed under vacuum, and the crude mixture was purified by chromatography to afford a colorless alcohol **13** as a single diastereoisomer (20 mg, 95%). This compound was treated as an intermediate.

$^{1}$H NMR (500 MHz, $C_6D_6$) δ 7.96 (dt, *J* = 8.3, 1.0 Hz, 1H), 7.35 (d, *J* = 7.6 Hz, 1H), 7.27 (ddd, *J* = 8.3, 7.2, 1.4 Hz, 1H), 7.20 (td, *J* = 7.6, 1.0 Hz, 1H), 3.95 (td, *J* = 8.9, 8.2, 4.1 Hz, 1H), 3.07 (dtd, *J* = 6.8, 3.3, 1.2 Hz, 1H), 2.18 (dddd, *J* = 13.2, 8.9, 6.8, 0.9 Hz, 1H), 1.79 (ddt, *J* = 13.2, 8.2, 1.3 Hz, 1H), 1.71 – 1.63 (m, 1H), 1.67 – 1.57 (m, 1H), 1.47 – 1.42 (m, 1H), 1.41 – 1.33 (m, 1H), 0.93 – 0.86 (m, 1H).

$^{13}$C NMR (126 MHz, $C_6D_6$) δ 152.0, 142.1, 137.5, 128.4, 125.4, 123.6, 122.1, 118.0, 114.5, 83.2, 71.8, 51.9, 42.5, 38.4, 30.0, 29.2, 28.0, 27.6, 24.7, 22.9.

ESI-HRMS: calculated for $C_{22}H_{29}N_1O_3Na_1$ ([M+Na]$^+$): 378.203963, found: 378.204040.

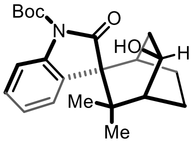



**Tert-butyl(1R,2S,4R,5S)-5-hydroxy-3,3-dimethyl-2'-oxospiro[bicyclo[2.2.2]octane-2,3'-indoline]-1'-carboxylate (14)**

To a vial was added alcohol **13** (5 mg, 0.014 mmol), benzene (0.5 mL) and lead tetraacetate (6.2 mg, 1 equiv.). The resulting mixture was heated to 80 °C, and was stirred for 1 h until all alcohol was consumed (monitored by TLC). The crude was directly purified by chromatography to afford compound **14** (1.6 mg, 30%.).

$^{1}$H NMR (500 MHz, $CD_2Cl_2$) δ 7.76 (ddd, *J* = 8.1, 1.3, 0.5 Hz, 1H), 7.69 (dd, *J* = 7.8, 1.3 Hz, 1H), 7.29 (ddd, *J* = 8.1, 7.5, 1.2 Hz, 1H), 7.11 (ddd, *J* = 7.9, 7.5, 1.3 Hz, 2H), 4.03 (d, *J* = 10.6 Hz, 0H), 3.79 (s, 1H), 2.62 (dqd, *J* = 14.7, 3.6, 0.7 Hz, 2H), 2.30 – 2.21 (m, 1H), 2.14 – 2.05 (m, 1H), 1.91 (ddd, *J* = 14.7, 10.6, 2.9 Hz, 1H), 1.71 (p, *J* = 2.9 Hz, 1H).

$^{13}$C NMR (126 MHz, $CD_2Cl_2$) δ 178.2, 149.4, 139.8, 131.0, 128.0, 127.9, 123.3, 114.4, 84.7, 71.8, 57.1, 44.4, 36.2, 36.1, 33.0, 31.5, 29.6, 28.2, 21.9, 21.5.

ESI-HRMS: calculated for $C_{22}H_{29}N_1O_4Na_1$ ([M+Na]$^+$): 394.198877, found: 394.199330.

HPLC (AD-3, *n*-heptane/*i*-PrOH=98:2, 1.0 mL/min, 298 K, 282 nm): $t_R$(major) = 8.54 min, $t_R$(major) = 9.51 min. e.r. = 94:6 (88% ee).

$[\alpha]_D^{25}$ = +28 (c = 0.1 $CH_2Cl_2$).

IR (film): 3433, 2926, 1781, 1728, 1150, 748.

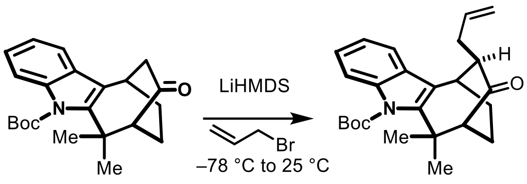

**10** **15**

**Tert-butyl(7R,9R,10R)-9-allyl-6,6-dimethyl-8-oxo-7,8,9,10-tetrahydro-7,10-ethanocyclohepta[b]indole-5(6H)-carboxylate (15)**

To a flame-dried Schlenk flask was added ketone **10** (8.87 mg, 0.025 mmol) and dry THF (1 mL) under Ar. The flask was cooled to –78 °C, and followed by the addition of LiHMDS (1 M in THF, 0.15 mL, 6 equiv.). The resulting mixture was stirred for 20 min at this temperature, and to the solution was added allylbromide (7 mg, 0.058 mmol, 2 equiv.). The cooling bath was removed, and the reaction was stirred at 25 °C for 6 h (no further conversion by TLC). The reaction was treated with distilled water (3 mL), and was extracted with ethyl ether (3x5 mL ). The layers were separated, and the organic layers was combined, and dried over anhydrous $MgSO_4$, and the solvent was removed under vacuum. The crude mixture was purified by chromatography to recover the remaining starting material **10** (4.5 mg) and afford desired compound **15** as single isomer (4 mg, 40%, b.r.s.m 80%).

$^1$H NMR (600 MHz, $CD_2Cl_2$) δ 7.71 (ddd, *J* = 8.3, 1.0 Hz, 32H), 7.42 (ddt, *J* = 7.8, 1.3, 0.6 Hz, 1H), 7.21 (ddd, *J* = 8.4, 7.1, 1.3 Hz, 1H), 7.16 (ddd, *J* = 7.8, 7.1, 1.0 Hz, 1H), 5.86 (dddd, *J* = 17.1, 10.2, 8.4, 5.3 Hz, 1H), 5.06 (dtd, *J* = 10.2, 1.8, 0.8 Hz, 3H), 4.91 (dtd, *J* = 17.1, 2.0, 1.1 Hz, 1H), 3.50 (ddd, *J* = 5.2, 3.4, 1.8 Hz, 1H), 2.62 (dt, *J* = 10.4, 3.5 Hz, 1H), 2.56 (dddt, *J* = 14.7, 5.4, 3.6, 2.0 Hz, 1H), 2.38 – 2.30 (m, 2H), 2.34 – 2.30 (m, 1H), 2.14 – 2.05 (m, 1H), 2.07 – 2.01 (m, 1H), 2.03 – 1.97 (m, 1H), 1.69 (s, 8H), 1.65 (s, 3H), 1.59 (dddt, *J* = 14.6, 10.4, 8.4, 1.1 Hz, 9H), 1.50 (s, 3H).

$^{13}$C NMR (151 MHz, $CD_2Cl_2$) δ 214.5, 151.7, 140.9, 137.5, 136.7, 129.0, 124.0, 122.0, 121.9, 118.7, 116.8, 114.2, 84.6, 61.8, 54.2, 41.4, 32.7, 29.7, 28.3, 28.3, 28.1, 26.9, 20.2.

ESI-HRMS: calculated for $C_{25}H_{31}N_1O3Na_1$ ($[M+Na]^+$): 416.219612, found: 416.219890.

HPLC (AD-3, Acetonitrile/water=40:60, 1.2 mL/min, 298 K, 282 nm): $t_R$(major) = 11.77 min, $t_R$(major) = 13.86 min. e.r. = 95:5 (90% ee).

$[\alpha]_D^{25}$ = –2 (c = 0.2 $CH_2Cl_2$).

IR (film): 2926, 1740, 1708, 1149, 818.

## 6. Synthesis of catalysts

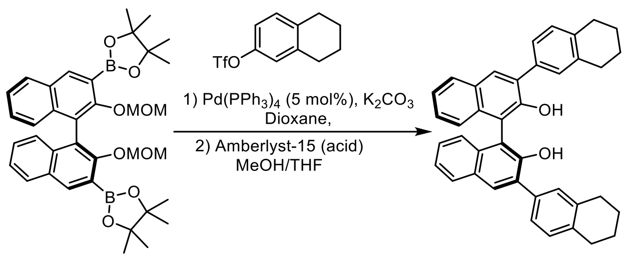


**(S)-BINOL**

To a flame dried two-neck round-bottom flask with a condenser was added 2,2'-(2,2'-bis(methoxymethoxy)-[1,1'-binaphthalene]-3,3'-diyl)bis(4,4,5,5-tetramethyl-1,3,2-dioxaborolane) (1 g, 1.6 mmol, 1.0 equiv), 5,6,7,8-tetrahydronaphthalen-2-yl trifluoromethanesulfonate (1.3 g, 4.78 mmol, 3 equiv.), tetrakis(triphenylphosphine)palladium (92 mg, 0.08 mmol, 0.05 equiv.) and barium hydroxide octahydrate (1.5 g, 4.8 mmol, 3 equiv.). After degassing the reaction mixture with argon for 20 min, 1,4-dioxane (30 ml) and degassed $H_2O$ (10 mL) were sequentially added. The mixture was then heated to 85 °C and stirred at that temperature overnight. After cooling the reaction to room temperature, the mixture was filtered through a pad of celite, washing with DCM (3 x 50 mL). The layers were separated, and the aqueous was extracted with DCM (2 x 50 mL). The combined organic phases were washed with brine (100 mL), dried over anhydrous $MgSO_4$, and filtered. The solvent was removed under vacuum to afford a light yellow solid, which was directly used for the next step. Subsequently, the solid was dissolved in methanol (15 mL), THF (15 mL) and amberlyst (excess, 3 g) was added to this mixture at room temperature. This mixture was heated to 80 °C and stirred overnight until the starting material was consumed (as monitored by TLC analysis). The reaction was then cooled to room temperature and quenched with water (50 mL). The resulting mixture was extracted with DCM (2 x 50 mL), then the layers were separated. The aqueous was extracted with DCM (50 mL), and the combined organic layers were dried over anhydrous $MgSO_4$. Following filtration of the suspension through celite, the volatiles were removed under reduced pressure. The resulting residue was purified by column chromatography (EtOAc: isohexane = 1:50 to 1: 9) to afford (*S*)- 5,5''',6,6''',7,7''',8,8'''-octahydro-[2,2':4',1'':3'',2'''-quaternaphthalene]-2'',3'-diol (700 mg, 1.59 mmol, 80%) as a light yellow solid.

**(*S*)- 5,5''',6,6''',7,7''',8,8'''-octahydro-[2,2':4',1'':3'',2'''-quaternaphthalene]-2'',3'-diol**

$^1$H NMR (501 MHz, $CD_2Cl_2$) δ 7.98 (s, 2H), 7.93 (dd, *J* = 8.2, 1.3 Hz, 2H), 7.45 – 7.36 (m, 6H), 7.30 (ddd, *J* = 8.3, 6.8, 1.3 Hz, 2H), 7.19 (dd, *J* = 8.2, 4.1 Hz, 4H), 5.45 (s, 2H), 2.85 (d, *J* = 6.2 Hz, 8H), 1.85 (p, *J* = 3.1 Hz, 8H).

$^{13}$C NMR (126 MHz, $CD_2Cl_2$) δ 150.6, 137.9, 137.5, 134.9, 133.4, 131.4, 131.2, 130.5, 129.9,

129.7, 128.7, 127.3, 127.0, 124.6, 124.5, 113.2, 29.6, 23.7. (two carbons were overlap at 23.7 ppm)
ESI-HRMS: calculated for $C_{40}H_{33}O_2$ ([M-H]$^-$): 545.248605, found: 545.249000

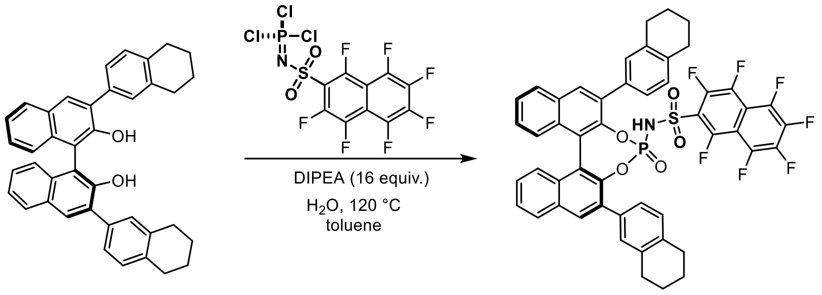


(S)-BINOL (S)-IDPi 6b

To a flame-dried Schlenk flask was charged with (*S*)- 5,5''',6,6''',7,7''',8,8'''-octahydro-[2,2':4',1'':3'',2'''-quaternaphthalene]-2'',3'-diol (30 mg, 0.054 mmol, 1 equiv.) under Ar, then dry toluene (3 mL) was added. The solution was flushed with Ar until the solvent was removed, and this procedure was repeated twice. To this flask was added dry toluene (2 mL) under to dissolve the solid, then *N, N*-diisopropylethylamine (0.077 mL, 0.44 mmol, 8 equiv.) was added, followed by the addition of *N*-Tf trichlorophosphazene (26 mg, 0.55 mmol, 1 equiv.).[3] The solution was stirred for 30 min, and then $H_2O$ (19 µL, 0.091 mmol, 1.5 equiv.) was added. The mixture was heated to 80 °C overnight. The reaction mixture was cooled to room temperature, and DCM (5 mL) was added to dilute the solution, treated with HCl (1 M, aq., 5 mL). The layers were separated, and the aqueous phase was further extracted with DCM (2 x 5 mL). The combined organic layers were dried over anhydrous $MgSO_4$, filtered. The solvent was removed under vacuum to afford a yellow mixture, which was purified by column chromatography to furnish a light yellow solid. The resulting solid was dissolved in DCM (5 mL) and to this solution was added HCl (6 M aq., 5 mL). The mixture was vigorously stirred for 20 min, then the organic layer was separated, and the aqueous was extracted with DCM (2 x 5 mL). The combined organic solvent was removed in vacuo, and the resulting solid was placed under the higher vacuum at 25 °C to afford a dry IDPi **6b** catalyst (40 mg, 79%).

**1,3,4,5,6,7,8-Heptafluoro-*N*-(4-oxido-2,6-bis(5,6,7,8-tetrahydronaphthalen-2-yl)dinaphtho[2,1-d:1',2'-f][1,3,2]dioxaphosphepin-4-yl)naphthalene-2-sulfonamide 6b**

$^{31}$P NMR (203 MHz, $CD_2Cl_2$) δ -5.7.
$^{19}$F NMR (471 MHz, $CD_2Cl_2$) δ -111.19 (dd, *J* = 77.8, 18.2 Hz), -136.52 (d, *J* = 17.7 Hz), -140.80 (dt, *J* = 77.7, 17.4 Hz), -144.70 (dt, *J* = 58.5, 17.2 Hz), -146.29 (dt, *J* = 58.8, 18.6 Hz), -148.63 (d, *J* = 19.3 Hz), -153.79.
$^{1}$H NMR (501 MHz, $CD_2Cl_2$) δ 8.09 (s, 1H), 8.03 (s, 1H), 7.99 (d, *J* = 8.2 Hz, 1H), 7.96 (d, *J* = 8.2 Hz, 1H), 7.54 (ddd, *J* = 8.1, 6.4, 1.4 Hz, 1H), 7.50 – 7.40 (m, 3H), 7.39 (d, *J* = 2.0 Hz, 1H), 7.35 – 7.25 (m, 3H), 7.22 (dd, *J* = 8.5, 6.6 Hz, 2H), 6.88 (d, *J* = 7.9 Hz, 1H), 2.86 (q, *J* = 5.4 Hz, 2H), 2.77

– 2.66 (m, 6H), 1.76 (pd, $J$ = 14.7, 13.1, 7.5 Hz, 8H).
$^{13}$C NMR (126 MHz, $CD_2Cl_2$) δ 144.4, 144.3, 138.3, 138.1, 137.5, 137.2, 134.3, 134.3, 133.7, 133.6, 133.5, 133.3, 132.4, 132.3, 132.1, 131.9, 130.9, 130.7, 129.9, 128.9, 128.8, 128.7, 127.3, 127.3, 127.2, 126.9, 126.9, 126.9, 126.7, 29.8, 29.5, 29.4, 23.6, 23.5. (the missing carbon singals were due to overlap)
ESI-HRMS: calculated for $C_{50}H_{34}N_1O_5S_1P_1F_7$ ($[M+H]^+$): 924.177810, found: 924.177460

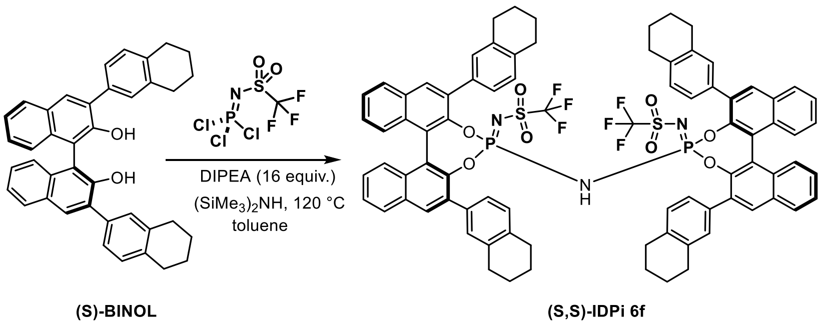


To a flame-dried Schlenk flask was charged with (*S*)- 5,5''',6,6''',7,7''',8,8'''-octahydro-[2,2':4',1'':3'',2'''-quaternaphthalene]-2'',3'-diol (100 mg, 0.18 mmol, 2 equiv.) under Ar, then dry toluene (3 mL) was added. The solution was flushed with Ar until the solvent was removed, and this procedure was repeated twice. The solid was dried under high vacuum at 50 °C overnight. To this flask was added dry toluene (3 mL) until dissolution of all solids, then *N, N*-diisopropylethylamine (0.25 mL, 1.46 mmol, 16 equiv.) was added, followed by the addition of *N*-Tf trichlorophosphazene (52 mg, 0.18 mmol, 2.1 equiv.).[3] The solution was stirred for 30 min, and then hexamethyldisilazane (19 μL, 0.091 mmol, 1.0 equiv.) was added. The mixture was heated to 120 °C for 48 h. The reaction mixture was cooled to room temperature, and DCM (5 mL) was added to dilute the solution, quenched with HCl (1 M, aq., 5 mL). The layers were separated, and the aqueous phase was further extracted with DCM (2 x 5 mL). The combined organic layers were dried over anhydrous $MgSO_4$, filtered. The solvent was removed under vacuum to afford a yellow mixture, which was purified by column chromatography to furnish a light yellow solid. The resulting solid was dissolved in DCM (5 mL) and to this solution was added HCl (6 M aq., 5 mL). The mixture was vigorously stirred for 20 min, then the organic layer was separated, and the aqueous was extracted with DCM (2 x 5 mL). The combined organic solvent was removed in vacuo, and the resulting solid was placed under the higher vacuum at 25 °C to afford a dry IDPi **6e** catalyst (50 mg, 37%).

***N,N'*-(azanediylbis(2,6-bis(5,6,7,8-tetrahydronaphthalen-2-yl)-4l5-dinaphtho[2,1-d:1',2'-f][1,3,2]dioxaphosphepine-4-yl-4-ylidene))bis(1,1,1-trifluoromethanesulfonamide) IDPi 6f**
$^{31}$P NMR (203 MHz, $CD_2Cl_2$) δ -17.91.
$^{19}$F NMR (471 MHz, $CD_2Cl_2$) δ -78.78.

$^{1}H$ NMR (501 MHz, $CD_2Cl_2$) δ 8.15 (s, 2H), 8.06 (d, $J$ = 8.2 Hz, 2H), 7.92 (d, $J$ = 8.2 Hz, 2H), 7.81 (ddd, $J$ = 8.1, 6.7, 1.2 Hz, 2H), 7.70 (d, $J$ = 8.5 Hz, 2H), 7.61 (ddd, $J$ = 8.3, 6.7, 1.3 Hz, 2H), 7.56 (ddd, $J$ = 8.2, 5.0, 3.0 Hz, 2H), 7.37 – 7.29 (m, 4H), 7.09 (d, $J$ = 1.9 Hz, 2H), 6.92 (d, $J$ = 8.0 Hz, 2H), 6.87 (d, $J$ = 8.0 Hz, 2H), 6.83 (s, 2H), 6.72 (d, $J$ = 8.0 Hz, 2H), 6.67 – 6.61 (m, 2H), 6.37 (d, $J$ = 1.9 Hz, 2H), 2.70 (ddt, $J$ = 38.2, 12.0, 6.4 Hz, 12H), 2.50 (dt, $J$ = 16.9, 5.9 Hz, 2H), 2.13 – 2.03 (m, 2H), 1.83 – 1.72 (m, 8H), 1.66 – 1.47 (m, 8H).
$^{13}C$ NMR (126 MHz, $CD_2Cl_2$) δ 144.6, 143.3, 137.8, 137.4, 137.3, 134.3, 133.7, 133.1, 132.9, 132.6, 132.2, 132.0, 131.8, 131.4, 131.2, 131.1, 129.8, 129.6, 129.1, 129.0, 128.8, 127.8, 127.4, 127.3, 127.3, 127.0, 126.8, 126.7, 123.5, 122.1, 118.2, 29.7, 29.6, 29.5, 29.4, 23.6, 23.6, 23.4, 23.4. . (the missing carbons signal are due to the overlap)
ESI-HRMS: calculated for $C_{82}H_{64}N_3O_8F_6P_2S_2$ ($[M-H]^-$): 1458.351986, found: 1458.352990.

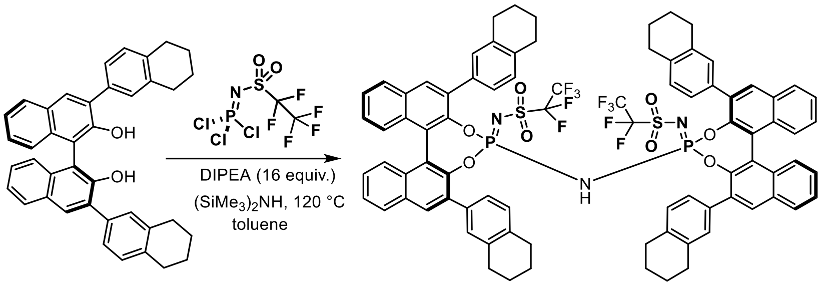


**(S)-BINOL** **(S,S)-IDPi 6g**

To a flame-dried Schlenk flask was charged with ($S$)- 5,5''',6,6''',7,7''',8,8'''-octahydro-[2,2':4',1'':3'',2'''-quaternaphthalene]-2'',3'-diol (100 mg, 0.18 mmol, 2 equiv.) under Ar, then dry toluene (3 mL) was added. The solution was flushed with Ar until the solvent was removed, and this procedure was repeated twice. The solid was dried under high vacuum at 50 °C overnight. To this flask was added dry toluene (3 mL) under to dissolve the solid, then $N$, $N$-diisopropylethylamine (0.25 mL, 1.46 mmol, 16 equiv.) was added, followed by the addition of perfluoroethyl-sulfonyl-phosphorimidoyl trichloride (61 mg, 0.18 mmol, 2.1 equiv.).[4] The solution was stirred for 30 min, and then hexamethyldisilazane (19 μL, 0.091 mmol, 1.0 equiv.) was added. The mixture was heated to 120 °C for 48 h. The reaction mixture was cooled to room temperature, and DCM (5 mL) was added to dilute the solution, quenched with HCl (1 M, aq., 5 mL). The layers were separated, and the aqueous phase was further extracted with DCM (2 x 5 mL). The combined organic layers were dried over anhydrous $MgSO_4$, filtered. The solvent was removed under vacuum to afford a yellow mixture, which was purified by column chromatography to furnish a light yellow solid. The resulting solid was dissolved in DCM (5 mL) and to this solution was added HCl (6 M aq., 5 mL). The mixture was vigorously stirred for 20 min, then the organic layer was separated, and the aqueous was extracted with DCM (2 x 5 mL). The combined organic solvent was removed in vacuo, and the resulting solid was placed under the higher vacuum at 25 °C to afford a dry IDPi

**6f** catalyst (70 mg, 49%).

**N,N'-(azanediylbis(2,6-bis(5,6,7,8-tetrahydronaphthalen-2-yl)-4l5-dinaphtho[2,1-d:1',2'-f][1,3,2]dioxaphosphepine-4-yl-4-ylidene))bis(1,1,1-trifluoromethanesulfonamide) IDPi 6g**

$^{19}$F NMR (471 MHz, $CD_2Cl_2$) δ -79.21, -116.33.
$^{31}$P NMR (203 MHz, $CD_2Cl_2$) δ -18.17.
$^{1}$H NMR (501 MHz, $CD_2Cl_2$) δ 8.13 (s, 2H), 8.05 (d, *J* = 8.3 Hz, 2H), 7.90 (d, *J* = 8.4 Hz, 2H), 7.81 (ddd, *J* = 8.1, 6.6, 1.2 Hz, 2H), 7.69 (d, *J* = 8.4 Hz, 2H), 7.62 (ddd, *J* = 8.4, 6.6, 1.3 Hz, 2H), 7.56 (ddd, *J* = 8.1, 6.2, 1.7 Hz, 2H), 7.36 – 7.27 (m, 4H), 7.11 (d, *J* = 1.9 Hz, 2H), 6.88 (dd, *J* = 8.0, 3.7 Hz, 4H), 6.79 (s, 2H), 6.67 (td, *J* = 7.4, 6.8, 1.9 Hz, 4H), 6.39 (d, *J* = 2.0 Hz, 2H), 2.85 – 2.61 (m, 12H), 2.50 (dt, *J* = 17.0, 5.9 Hz, 2H), 1.85 – 1.74 (m, 8H), 1.59 (tdd, *J* = 23.3, 17.4, 12.2 Hz, 8H).
$^{13}$C NMR (126 MHz, $CD_2Cl_2$) δ 145.0, 144.9, 144.9, 144.7, 137.7, 136.7, 136.5, 136.5, 135.2, 134.7, 133.9, 133.7, 132.3, 132.1, 132.1, 131.4, 130.7, 130.1, 129.3, 129.1, 128.9, 128.4, 128.2, 127.7, 127.5, 127.4, 126.8, 126.7, 126.3, 126.2, 123.4, 122.9, 29.5, 29.5, 29.5, 29.4, 23.7, 23.5. . (the missing carbons signal are due to the overlap)
ESI-HRMS: calculated for $C_{84}H_{64}N_3O_8F_{10}P_2S_2$ ($[M-H]^-$): 1558.345600, found: 1558.345820.

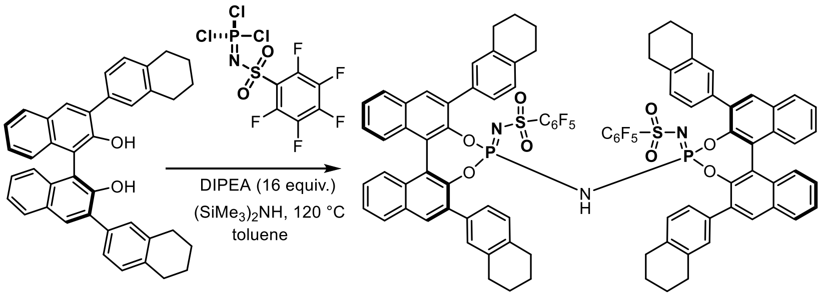


(S)-BINOL (S,S)-IDPi 6h

To a flame-dried Schlenk flask was charged with (*S*)-5,5''',6,6''',7,7''',8,8'''-octahydro-[2,2':4',1'':3'',2'''-quaternaphthalene]-2'',3'-diol (250 mg, 0.46 mmol, 2 equiv.) under Ar, then dry toluene (4 mL) was added. The solution was flushed with Ar until the solvent was removed, and this procedure was repeated twice. The solid was dried under high vacuum at 50 °C overnight. To this flask was added dry toluene (4 mL) under to dissolve the solid, then *N, N*-diisopropylethylamine (0.58 mL, 3.32 mmol, 16 equiv.) was added, followed by the addition of ((perfluorophenyl)sulfonyl)phosphorimidoyl trichloride (175 mg, 0.46 mmol, 2.1 equiv.).[5] The solution was stirred for 30 min, and then hexamethyldisilazane (43 µL, 0.02 mmol, 1.0 equiv.) was added. The mixture was heated to 130 °C for 72 h. The reaction mixture was cooled to room temperature, and DCM (5 mL) was added to dilute the solution, quenched with HCl (1 M, aq., 5 mL). The layers were separated, and the aqueous phase was further extracted with DCM (2 x 5 mL). The combined organic layers were dried over anhydrous $MgSO_4$, filtered. The solvent was removed under vacuum to afford a yellow mixture, which was purified by column chromatography

to furnish a light yellow solid. The resulting solid was dissolved in DCM (5 mL) and to this solution was added HCl (6 M aq., 5 mL). The mixture was vigorously stirred for 20 min, then the organic layer was separated, and the aqueous was extracted with DCM (2 x 5 mL). The combined organic solvent was removed in vacuo, and the resulting solid was placed under the higher vacuum at 25 °C to afford a dry IDPi **6g** catalyst (200 mg, 58%).

***N,N'*-(azanediylbis(2,6-bis(5,6,7,8-tetrahydronaphthalen-2-yl)-4l5-dinaphtho[2,1-d:1',2'-f][1,3,2]dioxaphosphepine-4-yl-4-ylidene))bis(2,3,4,5,6-pentafluorobenzenesulfonamide) IDPi 6h**

$^{19}$F NMR (471 MHz, $CD_2Cl_2$) δ -136.25 (s, 2F), -147.41 (s, 1F), -160.34(s, 2F).
$^{31}$P NMR (203 MHz, $CD_2Cl_2$) δ -15.25.
$^{1}$H NMR (501 MHz, $CD_2Cl_2$) δ 8.11 (s, 2H), 8.03 (d, $J$ = 8.3 Hz, 2H), 7.94 (d, $J$ = 8.2 Hz, 2H), 7.74 (ddd, $J$ = 7.9, 5.6, 2.0 Hz, 2H), 7.62 – 7.46 (m, 6H), 7.32 (t, $J$ = 7.7 Hz, 2H), 7.26 (d, $J$ = 8.5 Hz, 2H), 7.10 – 6.92 (m, 8H), 6.62 – 6.50 (m, 3H), 6.46 (s, 2H), 2.88 – 2.61 (m, 8H), 2.49 (tq, $J$ = 11.6, 8.3, 5.8 Hz, 6H), 2.16 – 1.96 (m, 2H), 1.84 – 1.72 (m, 8H), 1.48 (dddd, $J$ = 42.6, 20.6, 10.6, 5.6 Hz, 8H).
$^{13}$C NMR (126 MHz, $CD_2Cl_2$) δ 137.2, 137.0, 136.9, 136.7, 133.6, 133.3, 132.8, 132.6, 131.9, 131.8, 131.5, 131.4, 130.8, 129.8, 129.5, 129.0, 128.9, 128.4, 128.1, 127.3, 126.8, 126.7, 126.6, 126.5, 126.5, 126.4, 126.2, 122.2, 29.7, 29.2, 29.1, 29.0, 29.0, 23.1, 23.1, 22.9, 22.9. (the missing carbons signal are due to the overlap)
ESI-HRMS: calculated for $C_{92}H_{64}F_{10}N_3O_8P_2S_2$ ($[M-H]^-$): 1654.345600, found: 1654.346370.

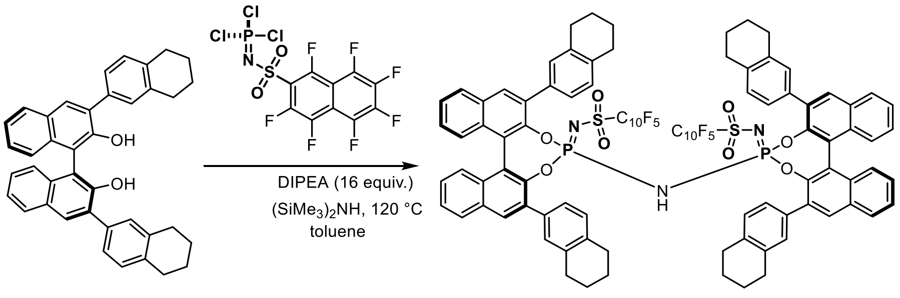


(S)-BINOL

(S,S)-IDPi 6i

To a flame-dried Schlenk flask was charged with (*S*)-5,5''',6,6''',7,7''',8,8'''-octahydro-[2,2':4',1'':3'',2'''-quaternaphthalene]-2'',3'-diol (250 mg, 0.46 mmol, 2 equiv.) under Ar, then dry toluene (4 mL) was added. The solution was flushed with Ar until the solvent was removed, and this procedure was repeated twice. The solid was dried under high vacuum at 50 °C overnight. To this flask was added dry toluene (4 mL) under to dissolve the solid, then *N, N*-diisopropylethylamine (0.58 mL, 3.32 mmol, 16 equiv.) was added, followed by the addition of ((perfluorophenyl)sulfonyl)phosphorimidoyl trichloride (175 mg, 0.46 mmol, 2.1 equiv.).[6] The solution was stirred for 30 min, and then hexamethyldisilazane (43 µL, 0.02 mmol, 1.0 equiv.) was added. The mixture was heated to 130 °C for 72 h. The reaction mixture was cooled to room temperature, and DCM (5 mL) was added to dilute the solution, quenched with HCl (1 M, aq., 5

mL). The layers were separated, and the aqueous phase was further extracted with DCM (2 x 5 mL). The combined organic layers were dried over anhydrous $MgSO_4$, filtered. The solvent was removed under vacuum to afford a yellow mixture, which was purified by column chromatography to furnish a light yellow solid. The resulting solid was dissolved in DCM (5 mL) and to this solution was added HCl (6 M aq., 5 mL). The mixture was vigorously stirred for 20 min, then the organic layer was separated, and the aqueous was extracted with DCM (2 x 5 mL). The combined organic solvent was removed in vacuo, and the resulting solid was placed under the higher vacuum at 25 °C to afford a dry IDPi **6h** catalyst (90 mg, 23 %).

**1,3,4,5,6,7,8-Heptafluoro-*N*-(4-((4-(((1,4,5,6,7,8-hexafluoronaphthalen-2-yl)sulfonyl)imino)-2,6-bis(5,6,7,8-tetrahydronaphthalen-2-yl)-4l5-dinaphtho[2,1-d:1',2'-f][1,3,2]dioxaphosphepin-4-yl)amino)-2,6-bis(5,6,7,8-tetrahydronaphthalen-2-yl)-4l5-dinaphtho[2,1-d:1',2'-f][1,3,2]dioxaphosphepin-4-ylidene)naphthalene-2-sulfonamide IDPi 6i**

$^{31}$P NMR (203 MHz, $CD_2Cl_2$) δ -11.75.

$^{19}$F NMR (471 MHz, $CH_2Cl_2$) δ -111.84 (dd, *J* = 77.4, 18.1 Hz, 1F), -133.89 (d, *J* = 17.2 Hz, 1F), -140.40 – -141.88 (m, 1F), -145.16 (dt, *J* = 58.5, 16.9 Hz, 1F), -146.99 (dt, *J* = 57.1, 18.8 Hz, 1F), -150.00 (t, *J* = 17.8 Hz, 1F), -154.72 (t, *J* = 18.6 Hz, 1F).

$^{1}$H NMR (501 MHz, $CD_2Cl_2$) δ 8.02 (t, *J* = 4.2 Hz, 4H), 7.83 (d, *J* = 8.3 Hz, 2H), 7.71 (t, *J* = 7.5 Hz, 2H), 7.44 (dd, *J* = 16.1, 8.0 Hz, 4H), 7.34 (s, 2H), 7.29 (d, *J* = 8.6 Hz, 2H), 7.15 (d, *J* = 7.2 Hz, 8H), 7.03 (d, *J* = 8.4 Hz, 2H), 6.73 (s, 2H), 6.54 – 6.30 (m, 2H), 6.04 (d, *J* = 7.8 Hz, 2H), 2.87 (dt, *J* = 17.1, 5.9 Hz, 2H), 2.78 – 2.53 (m, 8H), 2.41 – 2.23 (m, 4H), 2.10 (ddd, *J* = 16.6, 9.2, 4.5 Hz, 2H), 1.75 (dp, *J* = 12.4, 6.5 Hz, 8H), 1.56 – 1.35 (m, 8H).

$^{13}$C NMR (126 MHz, $CD_2Cl_2$) δ 143.7, 143.2, 137.3, 137.2, 136.6, 136.3, 133.9, 133.1, 132.5, 132.3, 132.0, 131.5, 131.4, 131.1, 130.8, 130.4, 129.9, 129.8, 129.5, 128.9, 128.1, 127.9, 127.0, 126.9, 126.7, 126.4, 126.4, 126.3, 126.2, 126.1, 122.8, 122.3, 29.3, 29.0, 29.0, 28.9, 23.2, 23.1, 23.0, 22.9. (the missing carbons signal are due to the overlap)

ESI-HRMS: calculated for $C_{100}H_{64}F_{14}N_3O_8P_2S_2$ ($[M-H]^-$): 1826.339214, found: 1826.338680.

## 7. Mechanistic Investigations

### General Procedure for the $^{31}P$ NMR

To a flame-dried NMR tube was charged with catalyst (10 to 15 mg), dry $CD_2Cl_2$ (0.5 mL), and the diene **2a** was added via a microsyringe, followed by the measurement of $^{31}P$ NMR at 25 °C.

**Silylation of chiral phosphoric acids 6a**

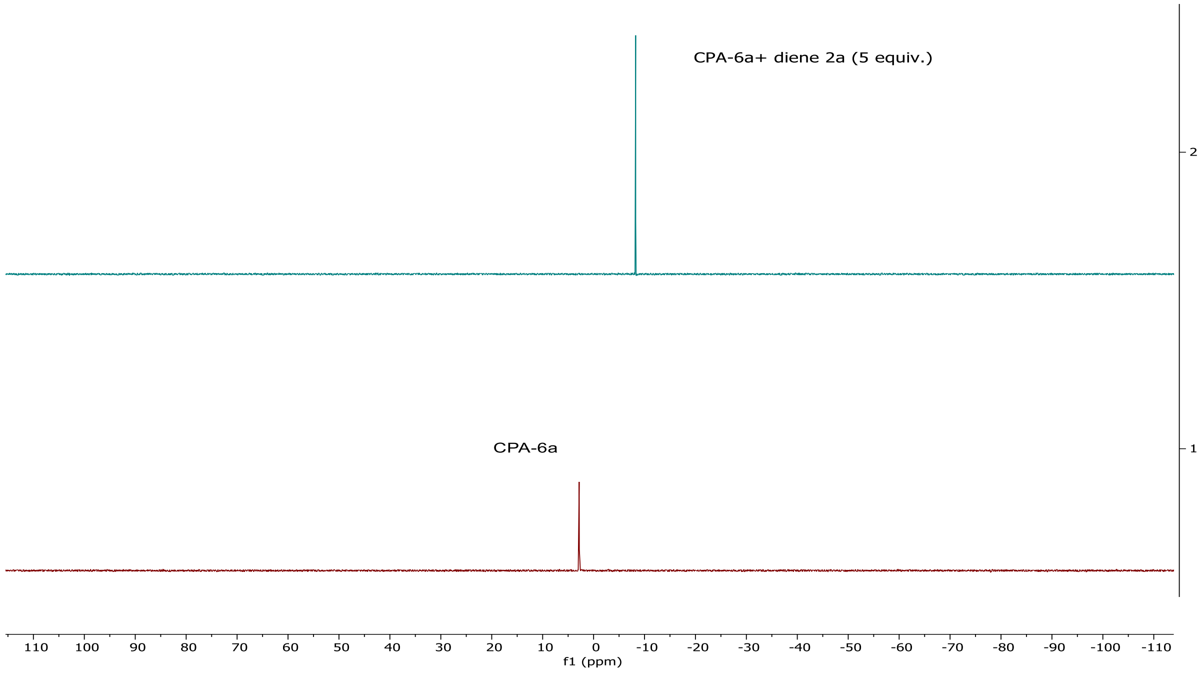


### Silylation of IDPi 6d

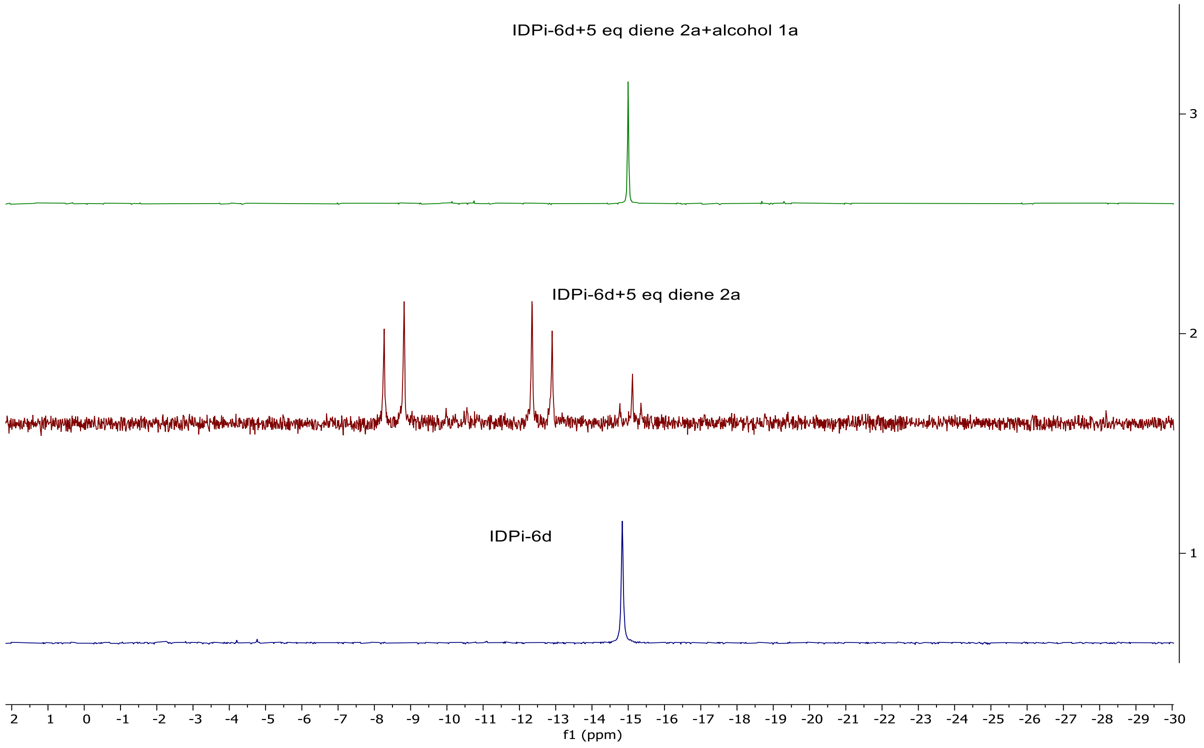

## 8. Reaction Progress Analysis by NMR

### General Procedure for the Sample Preparation

To six flame-dried NMR tubes were charged with alcohols **1a, 1d, 1h, 1i, 1j, 1k** (0.0143 mmol) separately, and added dry $CD_2Cl_2$ (0.5 mL). The diene **2a** (4 equiv.) was added to the above NMR tubes under Ar. The NMR tubes were cooled in a dry ice bath. After the parameters of NMR were set, the catalyst **6i** (1.8 mol% in 30 uL $CD_2Cl_2$) were added via microsyringe slowly at this temperature. The NMR tube was then transferred quickly to NMR spectrometer precooled to 223K (–50°C) and after fast shimming, single scan $^1$H NMR spectra were acquired every 2 min until the starting material was fully consumed. The data was then imported into MNOVA 14.2 with the reaction monitoring plugin and processed therein. All plots shown below showing the concentration plots in the linear time range, which were used for the initial rate extraction. (as shown in fig. 4).

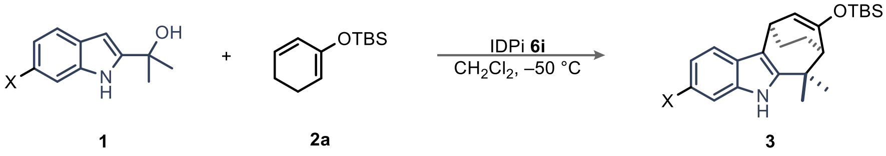


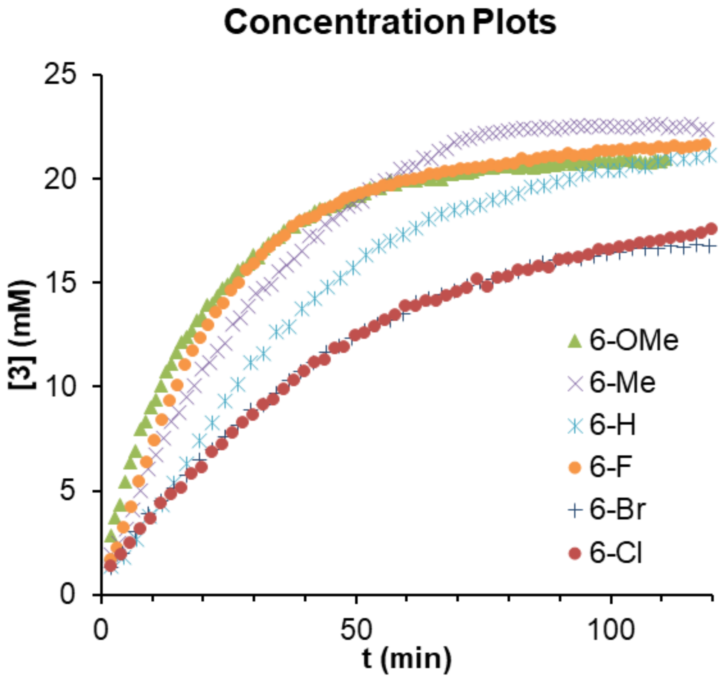


Figure 3. Concentration plots showing the formation of **3** obtained from NMR reaction progress monitoring.

To evaluate the reaction mechanism experimentally, a Hammet analysis with various substituents in the 6-position was performed (Figure 3). The 6-F substrate was found as outlier even after repeating the experiment a second time. As the indolium system is different compared to classic benzylic systems other resonance effect might explain this outlier. The other five substrates have a linear correlation with a negative slope ($\rho$=-0.91 ± 0.09; $R^2$=0.9724) when plotting $\log(k_X/k_H)$ against $\sigma_p$. This is consistent with the proposed indolium-cation formation in the rate-determining step.

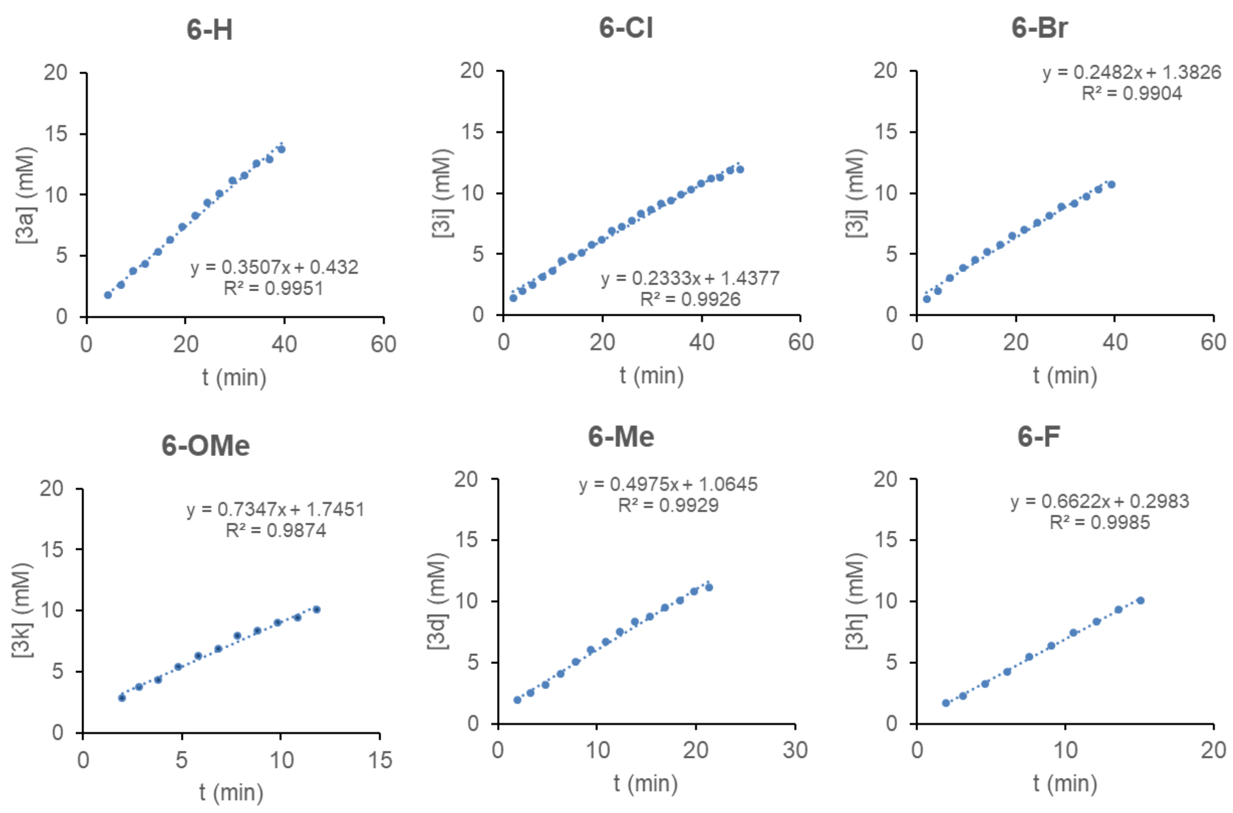


Figure 4. Linear region of the concentration plots obtained from NMR reaction progress monitoring used for reaction rate determination

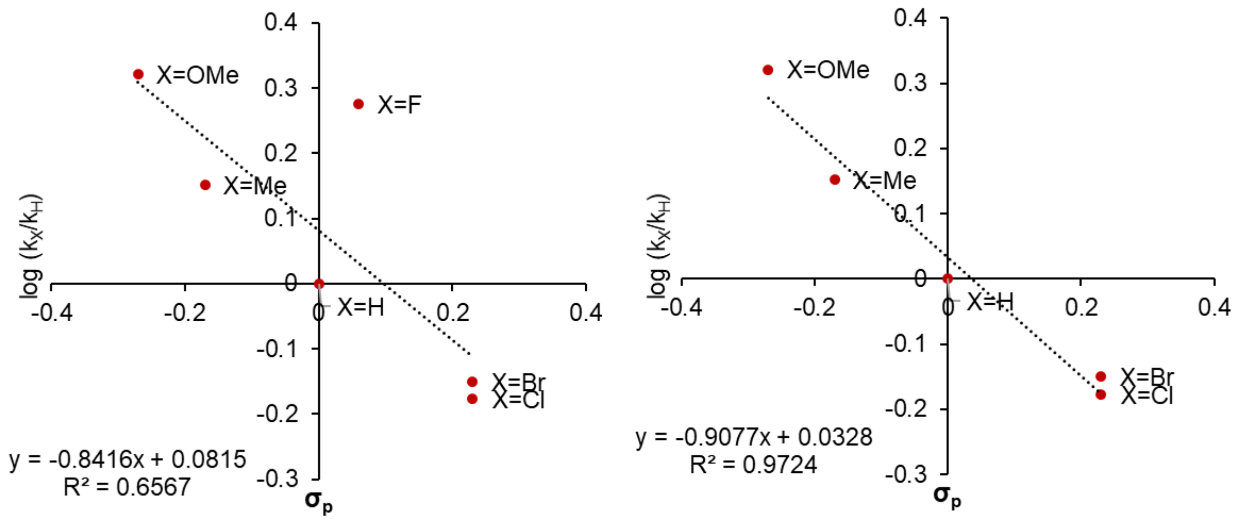


Figure 5: Hammet analysis of the (4+3) cycloaddition reaction. Left shows the analysis with 6-F and right without the 6-F substrate.

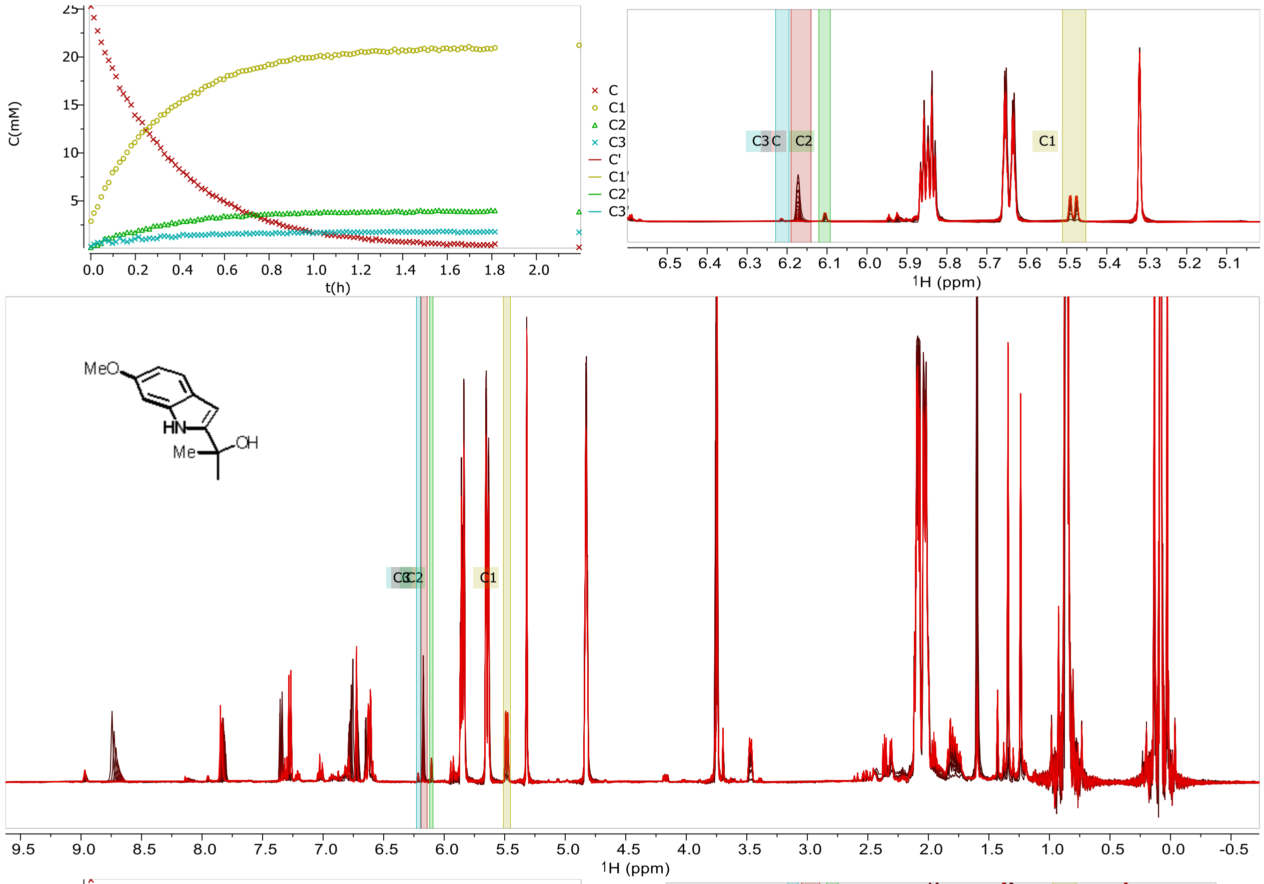
C(mM)
t(h)
C
C1
C2
C3
C'
C1'
C2'
C3'
$^{1}$H (ppm)
MeO
HN
Me
OH

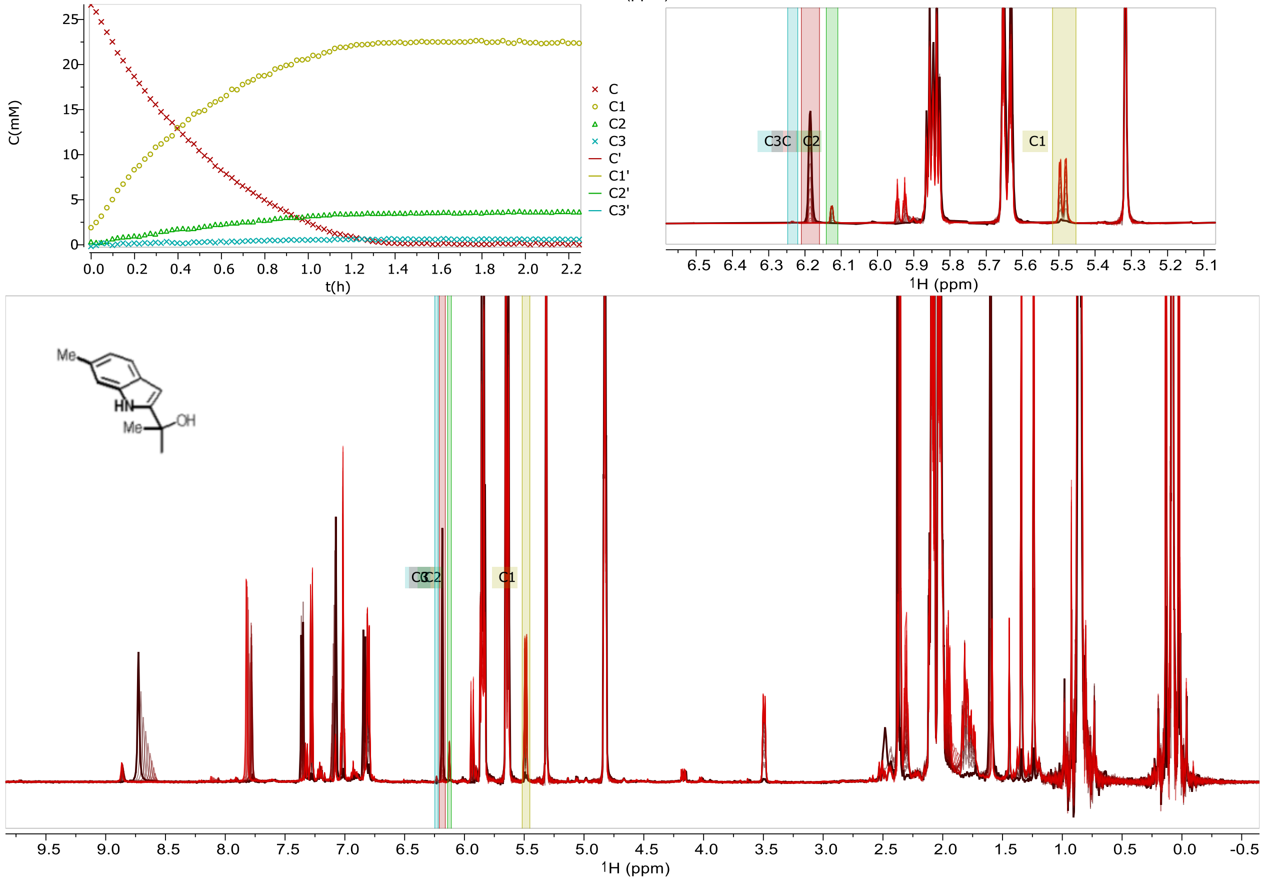
C(mM)
t(h)
C
C1
C2
C3
C'
C1'
C2'
C3'
$^{1}$H (ppm)
Me
HN
Me
OH

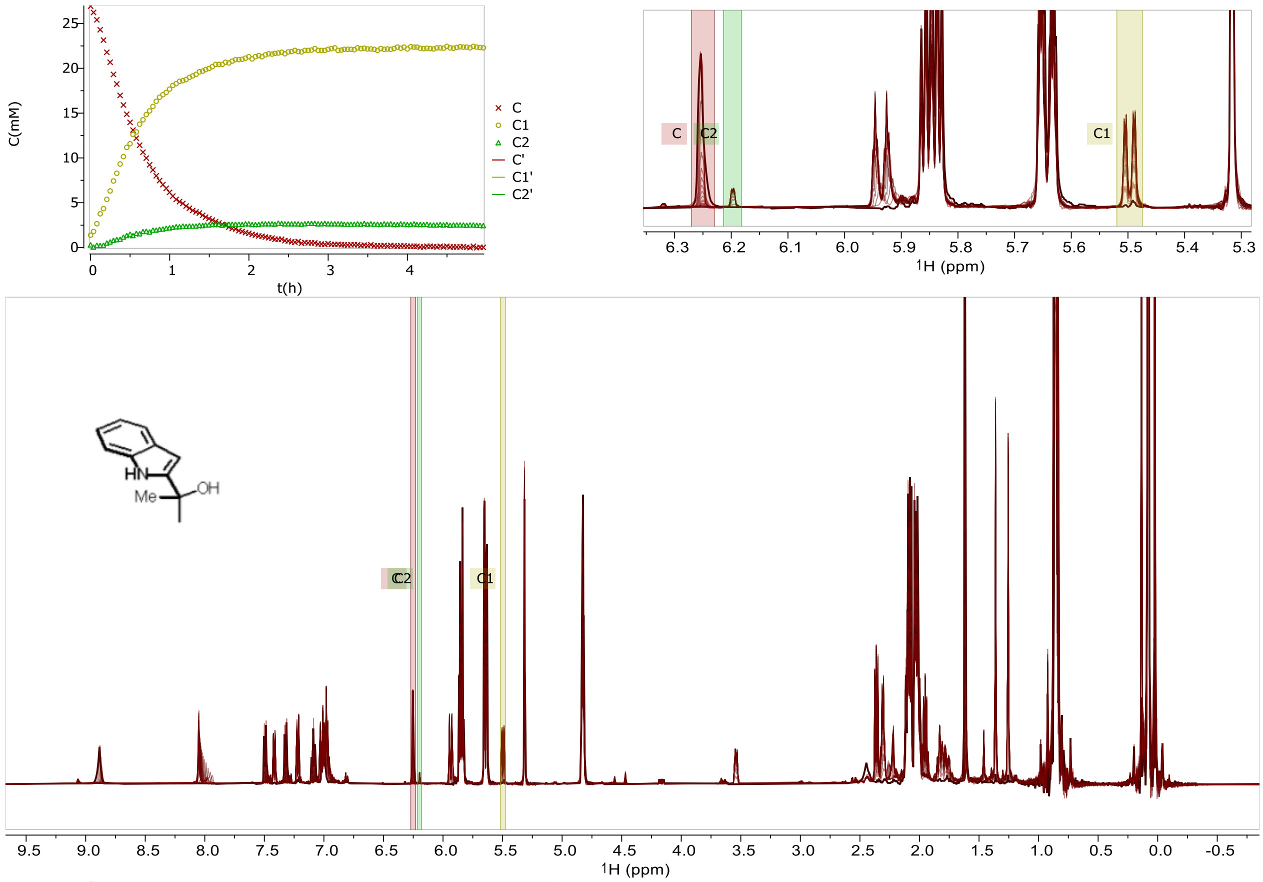
C(mM)
t(h)
C
C1
C2
C'
C1'
C2'
1H (ppm)
HN
Me
OH

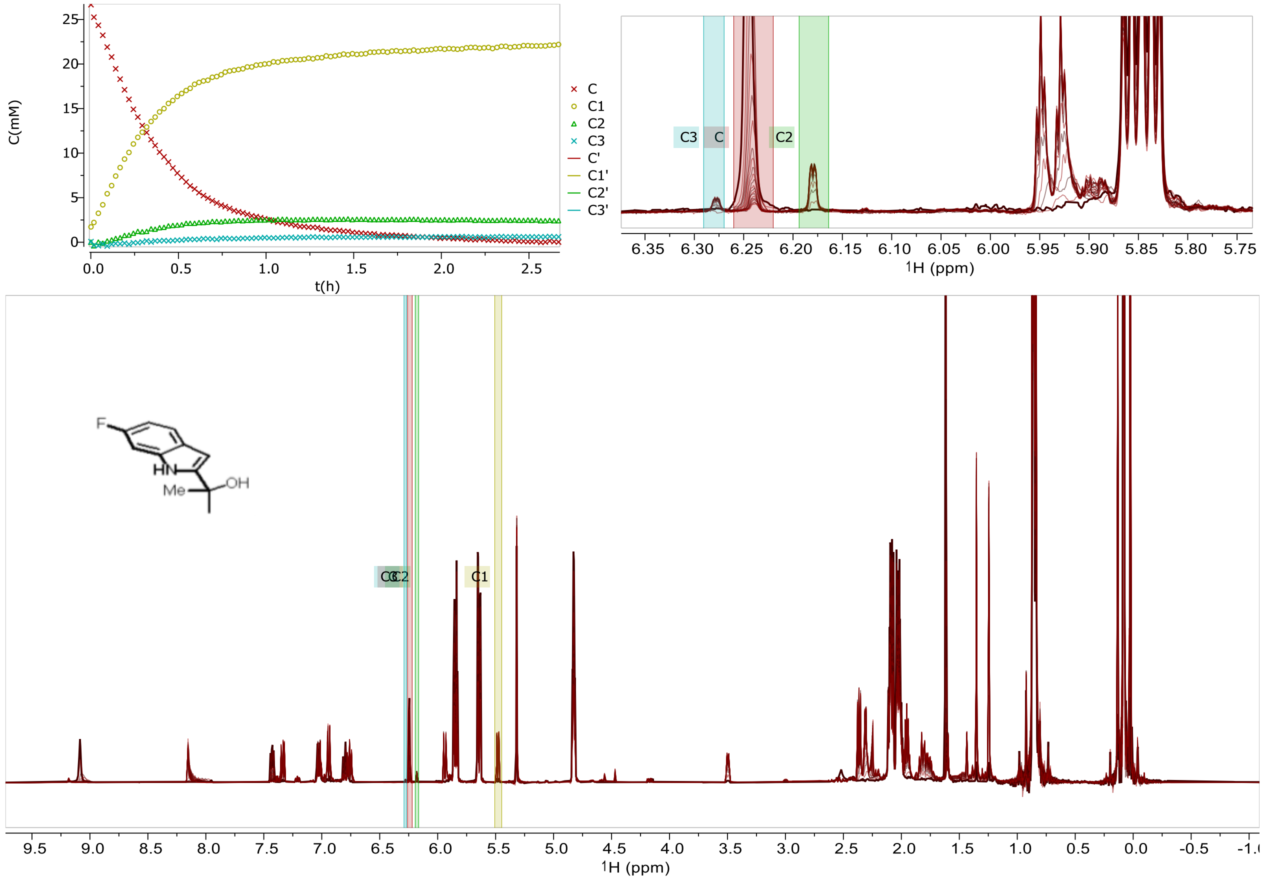
C(mM)
t(h)
C
C1
C2
C3
C'
C1'
C2'
C3'
1H (ppm)
F
HN
Me
OH

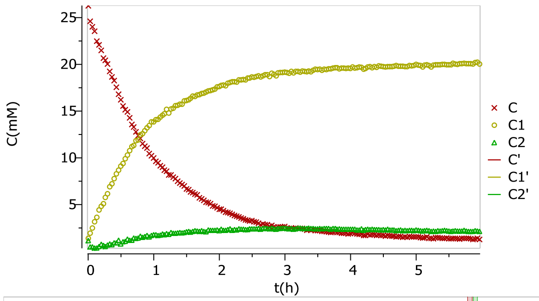
C(mM)
t(h)
C
C1
C2
C'
C1'
C2'

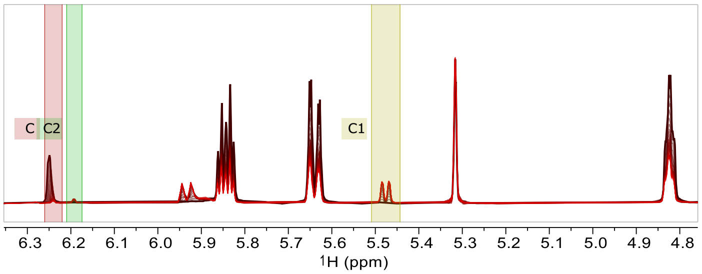
C
C2
C1
1H (ppm)

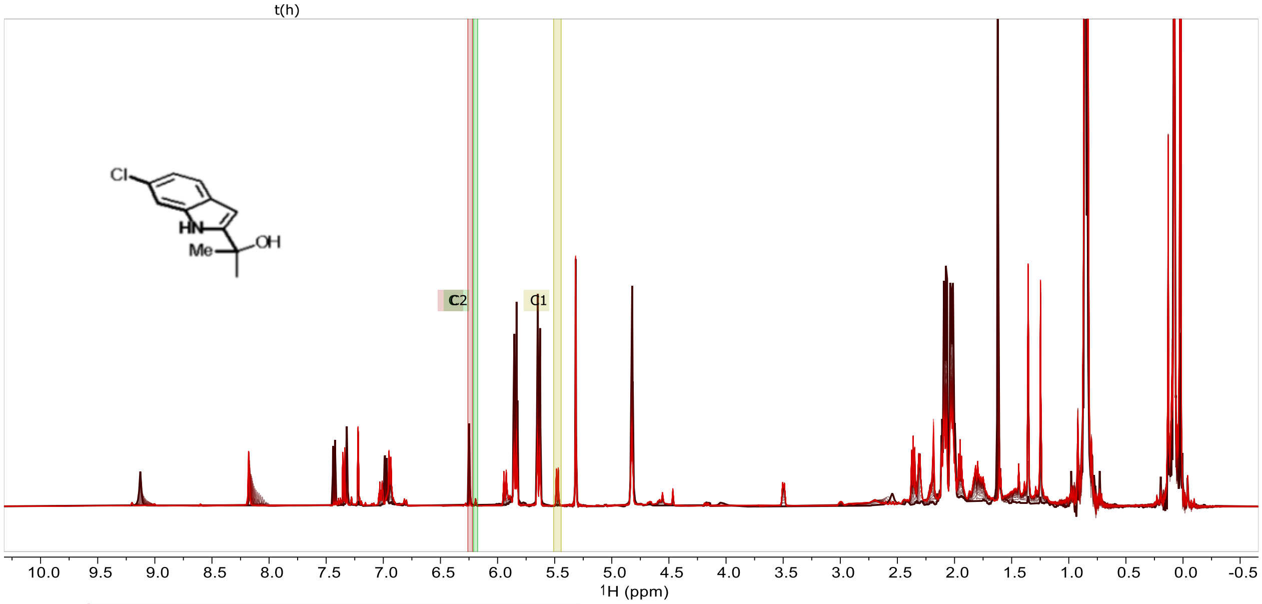
Cl
HN
Me
OH
C2
C1
1H (ppm)

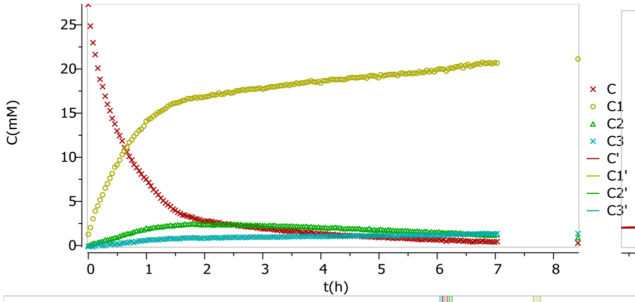
C(mM)
t(h)
C
C1
C2
C3
C'
C1'
C2'
C3'

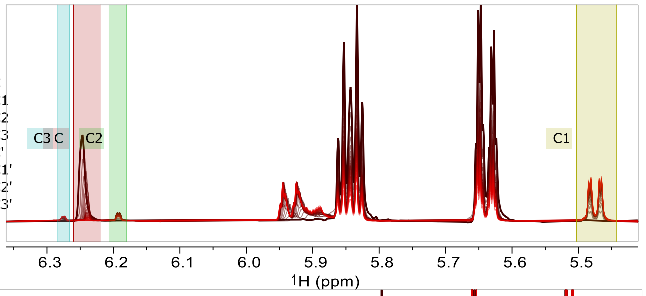
C3
C
C2
C1
1H (ppm)

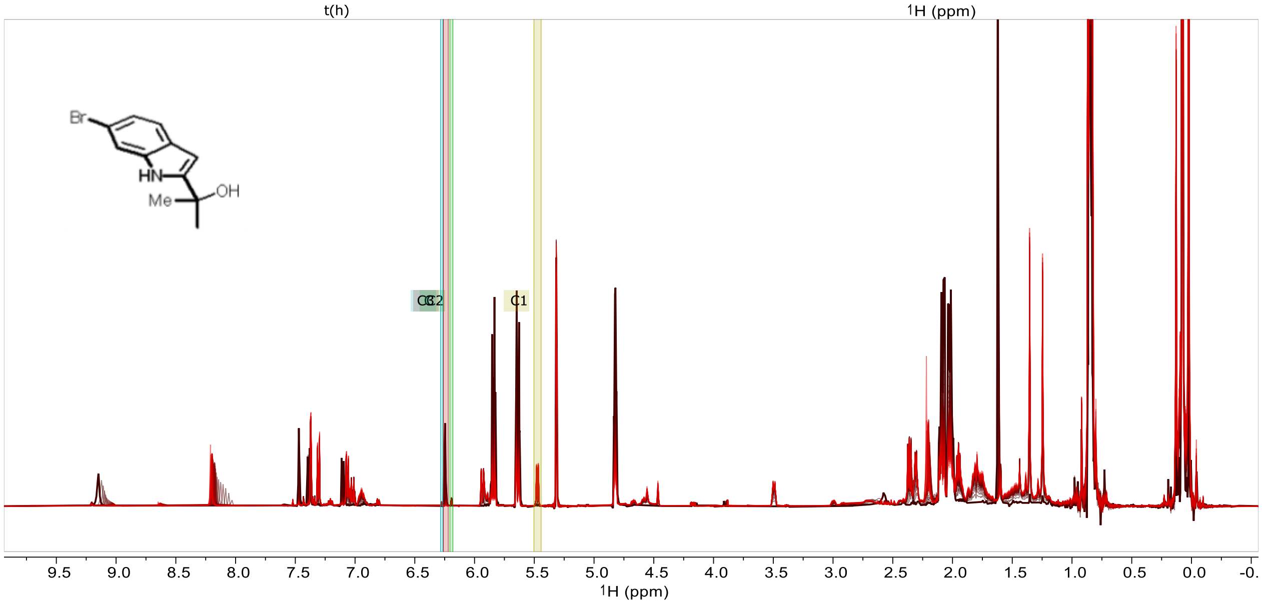
Br
HN
Me
OH
C1
1H (ppm)

## 9. Crystal Data of 4a

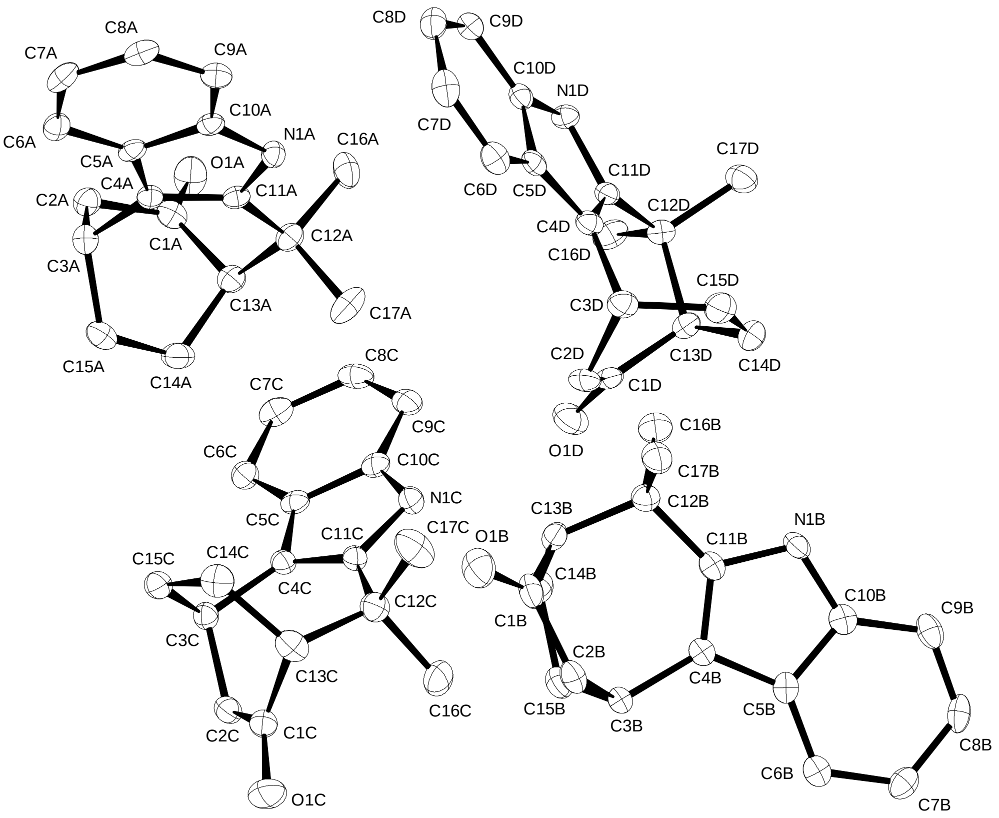


**Table 1. Crystal data of 4a and structure refinement.**

| | | |
|---|---|---|
| Identification code | 13763 | |
| Empirical formula | $C_{17} H_{19} N O$ | |
| Color | colourless | |
| Formula weight | 253.33 g·mol$^{-1}$ | |
| Temperature | 100(2) K | |
| Wavelength | 1.54178 Å | |
| Crystal system | ORTHORHOMBIC | |
| Space group | **$P2_12_12_1$, (no. 19)** | |
| Unit cell dimensions | a = 9.0263(3) Å | $\alpha$= 90°. |
| | b = 22.8189(8) Å | $\beta$= 90°. |
| | c = 26.6067(9) Å | $\gamma$ = 90°. |
| Volume | 5480.2(3) Å$^3$ | |
| Z | 16 | |
| Density (calculated) | 1.228 Mg · m$^{-3}$ | |
| Absorption coefficient | 0.589 mm$^{-1}$ | |
| F(000) | 2176 e | |

| | | |
|---|---|---|
| Crystal size | 0.267 x 0.160 x 0.120 $mm^3$ | |
| θ range for data collection | 2.551 to 62.944°. | |
| Index ranges | $-10 \le h \le 10$, $-26 \le k \le 26$, $-30 \le l \le 30$ | |
| Reflections collected | 166345 | |
| Independent reflections | 8823 [$R_{int}$ = 0.0453] | |
| Reflections with I>2σ(I) | 8590 | |
| Completeness to θ = 62.944° | 99.6 % | |
| Absorption correction | Gaussian | |
| Max. and min. transmission | 0.95 and 0.89 | |
| Refinement method | Full-matrix least-squares on $F^2$ | |
| Data / restraints / parameters | 8823 / 0 / 709 | |
| Goodness-of-fit on $F^2$ | 1.050 | |
| Final R indices [I>2σ(I)] | $R_1$ = 0.0272 | $wR^2$ = 0.0678 |
| R indices (all data) | $R_1$ = 0.0285 | $wR^2$ = 0.0686 |
| Absolute structure parameter | 0.05(5) | |
| Largest diff. peak and hole | 0.2 and -0.2 e · $Å^{-3}$ | |

**Table 2. Bond lengths [Å] and angles [°].**

| | | | |
|---|---|---|---|
| O(1A)-C(1A) | 1.217(3) | N(1A)-H(1A) | 0.87(3) |
| N(1A)-C(10A) | 1.379(3) | N(1A)-C(11A) | 1.385(3) |
| C(1A)-C(2A) | 1.502(3) | C(1A)-C(13A) | 1.522(3) |
| C(2A)-C(3A) | 1.535(3) | C(3A)-C(4A) | 1.506(3) |
| C(3A)-C(15A) | 1.536(3) | C(4A)-C(5A) | 1.432(3) |
| C(4A)-C(11A) | 1.368(3) | C(5A)-C(6A) | 1.416(3) |
| C(5A)-C(10A) | 1.408(3) | C(6A)-C(7A) | 1.382(3) |
| C(7A)-C(8A) | 1.397(3) | C(8A)-C(9A) | 1.376(3) |
| C(9A)-C(10A) | 1.396(3) | C(11A)-C(12A) | 1.512(3) |
| C(12A)-C(13A) | 1.566(3) | C(12A)-C(16A) | 1.537(3) |
| C(12A)-C(17A) | 1.541(3) | C(13A)-C(14A) | 1.543(3) |
| C(14A)-C(15A) | 1.532(3) | O(1B)-C(1B) | 1.217(3) |
| N(1B)-H(1B) | 0.85(3) | N(1B)-C(10B) | 1.378(3) |
| N(1B)-C(11B) | 1.385(3) | C(1B)-C(2B) | 1.508(3) |
| C(1B)-C(13B) | 1.514(3) | C(2B)-C(3B) | 1.539(3) |
| C(3B)-C(4B) | 1.505(3) | C(3B)-C(15B) | 1.534(3) |
| C(4B)-C(5B) | 1.447(3) | C(4B)-C(11B) | 1.363(3) |
| C(5B)-C(6B) | 1.405(3) | C(5B)-C(10B) | 1.404(3) |
| C(6B)-C(7B) | 1.385(3) | C(7B)-C(8B) | 1.405(3) |
| C(8B)-C(9B) | 1.379(3) | C(9B)-C(10B) | 1.395(3) |
| C(11B)-C(12B) | 1.516(3) | C(12B)-C(13B) | 1.562(3) |
| C(12B)-C(16B) | 1.537(3) | C(12B)-C(17B) | 1.538(3) |
| C(13B)-C(14B) | 1.555(3) | C(14B)-C(15B) | 1.540(3) |
| O(1C)-C(1C) | 1.218(3) | N(1C)-H(1C) | 0.94(3) |
| N(1C)-C(10C) | 1.377(3) | N(1C)-C(11C) | 1.381(3) |
| C(1C)-C(2C) | 1.503(3) | C(1C)-C(13C) | 1.513(3) |
| C(2C)-C(3C) | 1.539(3) | C(3C)-C(4C) | 1.508(3) |
| C(3C)-C(15C) | 1.532(3) | C(4C)-C(5C) | 1.446(3) |
| C(4C)-C(11C) | 1.368(3) | C(5C)-C(6C) | 1.406(3) |
| C(5C)-C(10C) | 1.412(3) | C(6C)-C(7C) | 1.385(3) |
| C(7C)-C(8C) | 1.398(3) | C(8C)-C(9C) | 1.382(3) |
| C(9C)-C(10C) | 1.393(3) | C(11C)-C(12C) | 1.515(3) |
| C(12C)-C(13C) | 1.561(3) | C(12C)-C(16C) | 1.541(3) |
| C(12C)-C(17C) | 1.534(3) | C(13C)-C(14C) | 1.561(3) |

| | | | |
|---|---|---|---|
| C(14C)-C(15C) | 1.528(3) | O(1D)-C(1D) | 1.223(3) |
| N(1D)-H(1D) | 0.84(3) | N(1D)-C(10D) | 1.377(3) |
| N(1D)-C(11D) | 1.391(3) | C(1D)-C(2D) | 1.506(3) |
| C(1D)-C(13D) | 1.519(3) | C(2D)-C(3D) | 1.532(3) |
| C(3D)-C(4D) | 1.508(3) | C(3D)-C(15D) | 1.540(3) |
| C(4D)-C(5D) | 1.433(3) | C(4D)-C(11D) | 1.369(3) |
| C(5D)-C(6D) | 1.402(3) | C(5D)-C(10D) | 1.412(3) |
| C(6D)-C(7D) | 1.384(3) | C(7D)-C(8D) | 1.402(3) |
| C(8D)-C(9D) | 1.385(3) | C(9D)-C(10D) | 1.397(3) |
| C(11D)-C(12D) | 1.509(3) | C(12D)-C(13D) | 1.572(3) |
| C(12D)-C(16D) | 1.542(3) | C(12D)-C(17D) | 1.539(3) |
| C(13D)-C(14D) | 1.539(3) | C(14D)-C(15D) | 1.536(3) |

| | | | |
|---|---|---|---|
| C(10A)-N(1A)-H(1A) | 124.1(17) | C(10A)-N(1A)-C(11A) | 109.18(17) |
| C(11A)-N(1A)-H(1A) | 125.9(17) | O(1A)-C(1A)-C(2A) | 120.89(18) |
| O(1A)-C(1A)-C(13A) | 121.97(19) | C(2A)-C(1A)-C(13A) | 117.12(17) |
| C(1A)-C(2A)-C(3A) | 111.54(16) | C(2A)-C(3A)-C(15A) | 108.67(17) |
| C(4A)-C(3A)-C(2A) | 111.09(17) | C(4A)-C(3A)-C(15A) | 111.26(17) |
| C(5A)-C(4A)-C(3A) | 126.03(18) | C(11A)-C(4A)-C(3A) | 126.91(18) |
| C(11A)-C(4A)-C(5A) | 107.04(18) | C(6A)-C(5A)-C(4A) | 133.81(19) |
| C(10A)-C(5A)-C(4A) | 107.34(18) | C(10A)-C(5A)-C(6A) | 118.84(18) |
| C(7A)-C(6A)-C(5A) | 118.4(2) | C(6A)-C(7A)-C(8A) | 121.37(19) |
| C(9A)-C(8A)-C(7A) | 121.7(2) | C(8A)-C(9A)-C(10A) | 117.4(2) |
| N(1A)-C(10A)-C(5A) | 107.25(17) | N(1A)-C(10A)-C(9A) | 130.38(19) |
| C(9A)-C(10A)-C(5A) | 122.37(19) | N(1A)-C(11A)-C(12A) | 121.03(18) |
| C(4A)-C(11A)-N(1A) | 109.17(18) | C(4A)-C(11A)-C(12A) | 129.79(18) |
| C(11A)-C(12A)-C(13A) | 110.03(17) | C(11A)-C(12A)-C(16A) | 110.83(16) |
| C(11A)-C(12A)-C(17A) | 109.26(17) | C(16A)-C(12A)-C(13A) | 108.91(17) |
| C(16A)-C(12A)-C(17A) | 108.19(18) | C(17A)-C(12A)-C(13A) | 109.58(17) |
| C(1A)-C(13A)-C(12A) | 111.72(16) | C(1A)-C(13A)-C(14A) | 107.86(17) |
| C(14A)-C(13A)-C(12A) | 116.76(17) | C(15A)-C(14A)-C(13A) | 115.79(17) |
| C(14A)-C(15A)-C(3A) | 111.87(17) | C(10B)-N(1B)-H(1B) | 129.4(17) |
| C(10B)-N(1B)-C(11B) | 108.84(18) | C(11B)-N(1B)-H(1B) | 121.5(17) |
| O(1B)-C(1B)-C(2B) | 121.6(2) | O(1B)-C(1B)-C(13B) | 121.3(2) |
| C(2B)-C(1B)-C(13B) | 117.12(18) | C(1B)-C(2B)-C(3B) | 111.64(18) |
| C(4B)-C(3B)-C(2B) | 111.61(16) | C(4B)-C(3B)-C(15B) | 112.23(17) |

| | | | |
|---|---|---|---|
| C(15B)-C(3B)-C(2B) | 107.20(17) | C(5B)-C(4B)-C(3B) | 126.65(18) |
| C(11B)-C(4B)-C(3B) | 126.71(19) | C(11B)-C(4B)-C(5B) | 106.62(18) |
| C(6B)-C(5B)-C(4B) | 133.8(2) | C(10B)-C(5B)-C(4B) | 106.99(18) |
| C(10B)-C(5B)-C(6B) | 119.25(19) | C(7B)-C(6B)-C(5B) | 118.7(2) |
| C(6B)-C(7B)-C(8B) | 120.9(2) | C(9B)-C(8B)-C(7B) | 121.4(2) |
| C(8B)-C(9B)-C(10B) | 117.5(2) | N(1B)-C(10B)-C(5B) | 107.78(18) |
| N(1B)-C(10B)-C(9B) | 130.0(2) | C(9B)-C(10B)-C(5B) | 122.27(19) |
| N(1B)-C(11B)-C(12B) | 121.25(18) | C(4B)-C(11B)-N(1B) | 109.77(18) |
| C(4B)-C(11B)-C(12B) | 128.97(19) | C(11B)-C(12B)-C(13B) | 110.47(16) |
| C(11B)-C(12B)-C(16B) | 110.01(18) | C(11B)-C(12B)-C(17B) | 109.58(17) |
| C(16B)-C(12B)-C(13B) | 108.81(17) | C(16B)-C(12B)-C(17B) | 108.78(18) |
| C(17B)-C(12B)-C(13B) | 109.17(18) | C(1B)-C(13B)-C(12B) | 113.13(17) |
| C(1B)-C(13B)-C(14B) | 108.99(17) | C(14B)-C(13B)-C(12B) | 113.87(18) |
| C(15B)-C(14B)-C(13B) | 113.45(18) | C(3B)-C(15B)-C(14B) | 111.77(17) |
| C(10C)-N(1C)-H(1C) | 124.2(15) | C(10C)-N(1C)-C(11C) | 108.61(18) |
| C(11C)-N(1C)-H(1C) | 127.1(15) | O(1C)-C(1C)-C(2C) | 122.0(2) |
| O(1C)-C(1C)-C(13C) | 120.9(2) | C(2C)-C(1C)-C(13C) | 117.17(17) |
| C(1C)-C(2C)-C(3C) | 111.33(17) | C(4C)-C(3C)-C(2C) | 112.60(16) |
| C(4C)-C(3C)-C(15C) | 110.89(16) | C(15C)-C(3C)-C(2C) | 106.92(16) |
| C(5C)-C(4C)-C(3C) | 126.76(18) | C(11C)-C(4C)-C(3C) | 126.55(18) |
| C(11C)-C(4C)-C(5C) | 106.64(18) | C(6C)-C(5C)-C(4C) | 134.43(19) |
| C(6C)-C(5C)-C(10C) | 118.99(19) | C(10C)-C(5C)-C(4C) | 106.55(17) |
| C(7C)-C(6C)-C(5C) | 118.5(2) | C(6C)-C(7C)-C(8C) | 121.3(2) |
| C(9C)-C(8C)-C(7C) | 121.5(2) | C(8C)-C(9C)-C(10C) | 117.4(2) |
| N(1C)-C(10C)-C(5C) | 108.10(18) | N(1C)-C(10C)-C(9C) | 129.6(2) |
| C(9C)-C(10C)-C(5C) | 122.31(19) | N(1C)-C(11C)-C(12C) | 121.52(18) |
| C(4C)-C(11C)-N(1C) | 110.09(18) | C(4C)-C(11C)-C(12C) | 128.39(19) |
| C(11C)-C(12C)-C(13C) | 110.04(17) | C(11C)-C(12C)-C(16C) | 109.23(18) |
| C(11C)-C(12C)-C(17C) | 110.45(18) | C(16C)-C(12C)-C(13C) | 110.06(18) |
| C(17C)-C(12C)-C(13C) | 108.32(19) | C(17C)-C(12C)-C(16C) | 108.72(19) |
| C(1C)-C(13C)-C(12C) | 111.82(19) | C(1C)-C(13C)-C(14C) | 109.35(17) |
| C(14C)-C(13C)-C(12C) | 113.21(18) | C(15C)-C(14C)-C(13C) | 113.09(18) |
| C(14C)-C(15C)-C(3C) | 111.00(17) | C(10D)-N(1D)-H(1D) | 125.7(17) |
| C(10D)-N(1D)-C(11D) | 109.24(17) | C(11D)-N(1D)-H(1D) | 125.0(17) |
| O(1D)-C(1D)-C(2D) | 122.0(2) | O(1D)-C(1D)-C(13D) | 121.3(2) |
| C(2D)-C(1D)-C(13D) | 116.61(17) | C(1D)-C(2D)-C(3D) | 110.86(17) |

| | | | |
|---|---|---|---|
| C(2D)-C(3D)-C(15D) | 110.14(18) | C(4D)-C(3D)-C(2D) | 109.51(17) |
| C(4D)-C(3D)-C(15D) | 111.45(18) | C(5D)-C(4D)-C(3D) | 126.90(18) |
| C(11D)-C(4D)-C(3D) | 125.54(19) | C(11D)-C(4D)-C(5D) | 107.56(17) |
| C(6D)-C(5D)-C(4D) | 133.89(19) | C(6D)-C(5D)-C(10D) | 119.22(19) |
| C(10D)-C(5D)-C(4D) | 106.88(17) | C(7D)-C(6D)-C(5D) | 118.9(2) |
| C(6D)-C(7D)-C(8D) | 120.9(2) | C(9D)-C(8D)-C(7D) | 121.7(2) |
| C(8D)-C(9D)-C(10D) | 117.2(2) | N(1D)-C(10D)-C(5D) | 107.54(17) |
| N(1D)-C(10D)-C(9D) | 130.32(19) | C(9D)-C(10D)-C(5D) | 122.14(19) |
| N(1D)-C(11D)-C(12D) | 121.30(18) | C(4D)-C(11D)-N(1D) | 108.77(18) |
| C(4D)-C(11D)-C(12D) | 129.93(18) | C(11D)-C(12D)-C(13D) | 110.28(17) |
| C(11D)-C(12D)-C(16D) | 109.61(17) | C(11D)-C(12D)-C(17D) | 110.68(16) |
| C(16D)-C(12D)-C(13D) | 108.23(16) | C(17D)-C(12D)-C(13D) | 109.72(17) |
| C(17D)-C(12D)-C(16D) | 108.26(18) | C(1D)-C(13D)-C(12D) | 111.47(16) |
| C(1D)-C(13D)-C(14D) | 107.71(18) | C(14D)-C(13D)-C(12D) | 116.32(17) |
| C(15D)-C(14D)-C(13D) | 114.62(18) | C(14D)-C(15D)-C(3D) | 111.36(17) |

## 10. Control Experiments

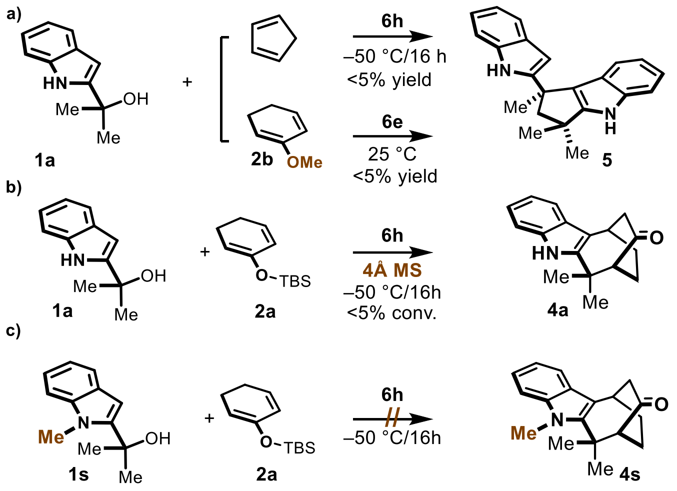

Scheme 1. Control experiments

a) To a flame-dried vial equipped with a stirring bar was added **1a** (2 mg, 0.011 mmol) and cyclopentadiene (4.0 equiv.) and $CD_2Cl_2$ (0.4 mL). The vial was placed into a dry-ice bath for 15 min. Under Ar, to this vial was added catalyst **6g**, then the vial was transferred to a –50 °C cryostat. After the reaction was stirred overnight, triethylamine was added to quench the reaction and the mixture was submitted to $^{1}$H NMR. The crude nmr shows a complicated profile, in which no desired product formed, but trace amount of the dimer was identified. The same procedure was applied to the diene **2b**[7], **1a,** IDPi **6d**, and no desired product was observed as well, and along with trace of dimer.

b) To a flame-dried vial equipped with a stirring bar was added **1a** (2 mg, 0.011 mmol) and diene **2a** (4.0 equiv.), dry molecular sieves (20 mg, 4Å) and $CD_2Cl_2$ (0.4 mL). The vial was placed into dry-ice bath for 15 min. Under Ar, to this vial was added catalyst **6g**, then was transferred to a –50 °C cryostat. After the mixture was stirred overnight, the reaction was monitored by TLC. No conversion of the starting material was observed.

c) To a flame-dried vial was equipped with a stirring bar was added **1s** (2 mg, 0.01 mmol) and diene **2a** (4 equiv.), and $CD_2Cl_2$ (0.4 mL). The vial was placed into dry-ice bath for 15 min. Under Ar, to this vial was added the catalyst **6g**, then the vial was transferred to a –50 °C cryostat. After the reaction was stirred overnight, the reaction was monitored by TLC. No conversion of the starting material was observed.

## 11. Limitations of this (4+3) Cycloaddition

Although this (4+3) cycloaddition had enabled a broad of scope of 2-indolyl alcohol cycloadditions, there are still limitations that are not solved yet. The unstable **1t** exclusively polymerized under our optimal conditions. The *gem*-diethyl indolyl alcohol **1u** and activated substrates **1w** or the diene **2d** only generate the desired product with moderate enantioselective ratios. Diene **2d** reacts with **1a** with a higher reactivity in a 88:12 enantioselective ratio. For the acyclic diene **2e** only give a $S_N1$ substitution without the ring closure. Interestingly, Martin's seven-membered dienolsilane **2f**, which was used in a synthesis of actinophonic acid, led to a Gramine-type fragmentation and gave

the corresponding product in 82:18 er.

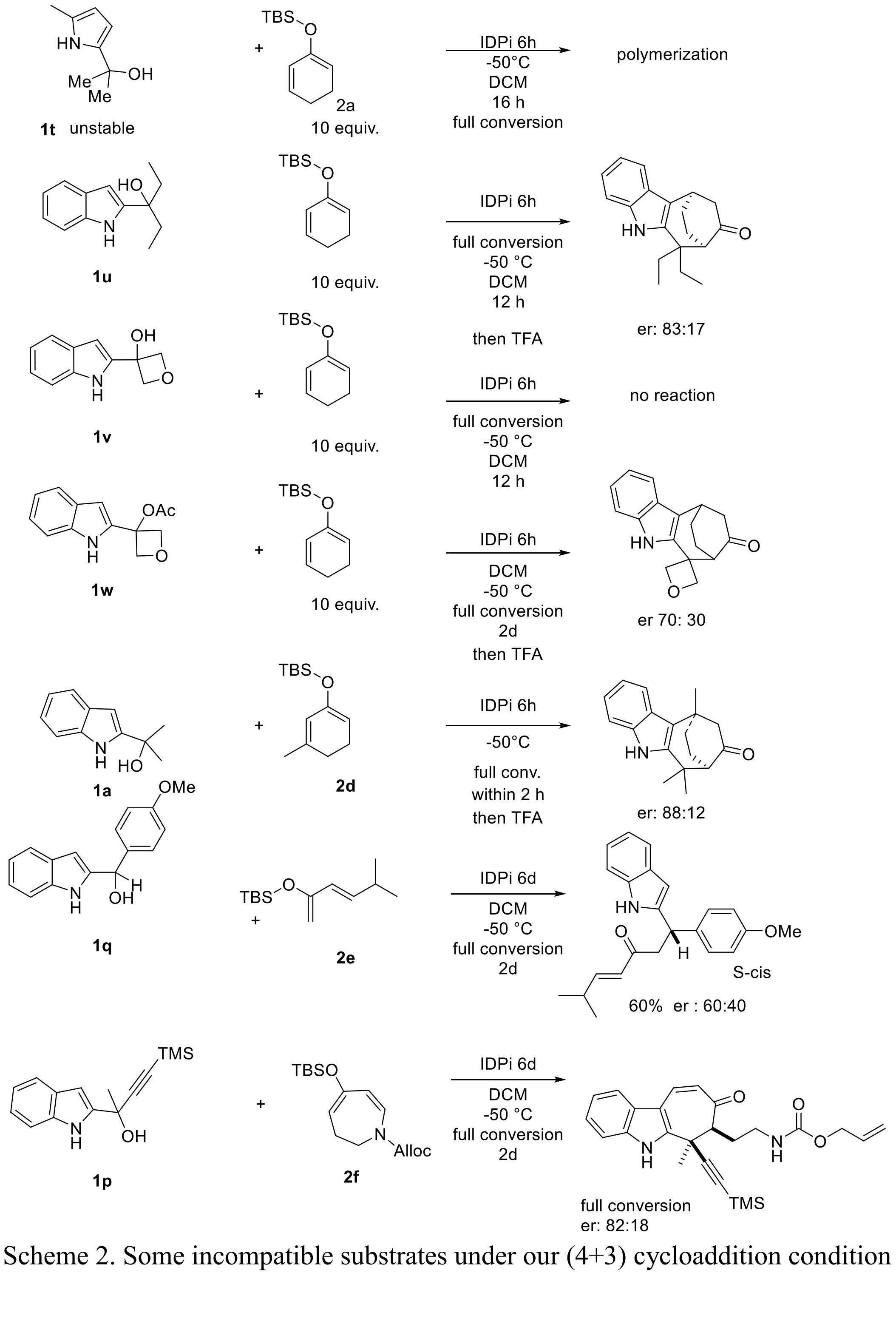


Scheme 2. Some incompatible substrates under our (4+3) cycloaddition condition

# 12. Computational Studies

## 12.1 Method

All calculations presented in this paper were carried out with a development version of the ORCA suite of programs based on version 4.2.[8] Molecular geometries were optimized in the gas-phase using the PBE functional[9] in conjunction with the D3 version of Grimme's dispersion correction with Becke-Johnson damping function,[10] using the resolution of identity approximation. The def2-SVP basis set was used for all atoms with matching auxiliary basis.[11] Analytic frequency calculations were performed to verify the nature of all stationary points (minima and transition states) and to calculate free energies and enthalpies at 223 K by using the rigid-rotor harmonic oscillator (RRHO) approximation. Solvation effect has been accounted by using CPCM(DCM) solvation model,[12] as implemented in ORCA. An exhaustive manual conformational search has been performed on possible catalyst substrate orientations. Analytic frequency calculations were performed to verify the nature of all stationary points (minima and transition states) and to calculate free energies and enthalpies at 223 K by using the rigid-rotor harmonic oscillator (RRHO) approximation. Transition state structures were verified by the presence of a single imaginary vibrational frequency. Single-point energies are calculated at B3LYP-D3(BJ)/def2-TZVP [13] and B3LYP/def2-TZVP[14] level of theory. A Distortion-interaction study,[15] NCI analysis[16] have been performed to qualitatively understand the reason behind stereoinduction. Molecular structures were generated using CYLview program.[17]

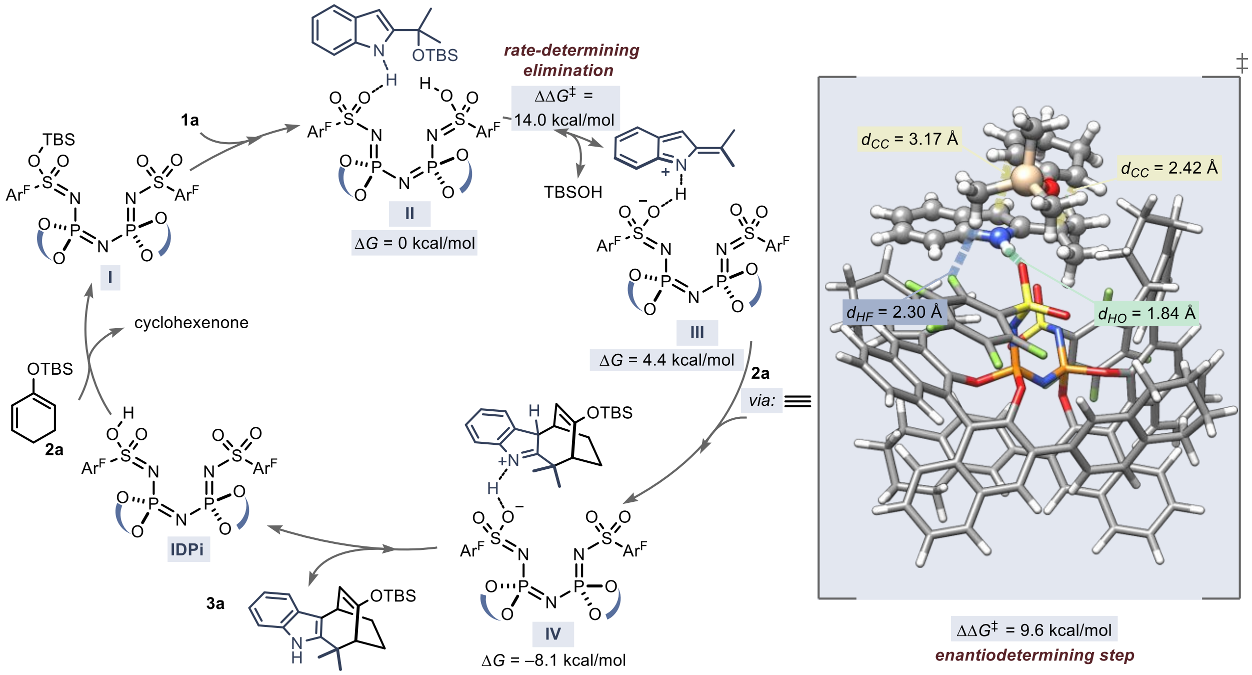


Figure 6. Possible mechanism

## 12.2 Analysis

Based on previous computational reports,[18] we anticipated that the C–C bond forming to be the stereo selectivity-determining step. To gain computational expediency, TBS group has been replaced by TMS group. Presence of several key orientations has been taken care of by a rigorous conformational search. One can envision two possible pathways to obtain the product (stepwise and concerted: see Fig. 10). However, all our attempts to identify a stepwise pathway remains futile, in line with the previous reports by Jacobsen [18] and Houk[19]. Optimized concerted pathway [4+3] cycloaddition) is highly asynchronous in

nature where in the formation of **III** C10–C11 bond is shorter (~2.4 Å) compared to the C9–C14 bond (3.1 Å). Computed free energy difference [1.38 kcal/mol, (*e.r*: 95.7:4.3)] at the *B3LYP-D3(BJ)/def2-TZVP+CPCM(*DCM*)//PBE-D3/def2-SVP level* is in excellent agreement to that of experimentally observed value [1.22 kcal/mol, (*er* : 94:6)]. Visual inspection of both stereodeterming TS structure revealed the presence of several week non-covalent interactions (e.g. CH...O, NH…O, CH…F *etc.*) in substrate recognition within the catalyst cavity. Among other notable structural features, both TS contains two perflourinated stacking interaction between its sulfonyl C6F5 substituents and the BINOL moiety. Moreover, only one of the two methyl group is pointed away from the confined cavity, the other methyl group stays inside (see Fig. 9). Such TS arrangement explains why the substitution on both Me group was not tolerated experimentally.

Special emphasis has been placed to understand the reason behind stereo induction. Gas phase energy difference between the two stereodeterming structure at the *B3LYP-D3(BJ)/def2-TZVP* found to be 5.2 kcal/mol, highlighting that these reactions are enthalpically driven. Subsequently, performing the Houk-Ess-Bickelhaupt distortion-Interaction analysis, [8] we observed the net distortion (see Table 5) is less for minor isomer (2.9 kcal/mol). Therefore, the major TS isomer enjoys 7.1 kcal/mol additional stabilization in gas phase. Consistent with this, we have identified several stabilizing interactions (such as π–π stacking interaction, CH..O interactions) are more prominent in the TS major. Additionally, a NCI plot [9] of two competing TS structures have identified more stabilizing non-covalent interactions arising from the superior stacking with the indole moiety with the catalyst arm, and stabilizing CH..O interaction originating from the catalyst and the TMS group (See Fig. 9).

***Notably, while dispersion corrected B3LYP functionals (B3LYP-D3) can correctly capture the reaction outcome, in absence of added dispersion (only B3LYP), the energetic ordering is incorrect (See Table S4): This also supports the importance of non-covalent interactions in controlling selectivity.***

Next, we turned our attention to understand the rate determining step for this transformation. Previous reports on a similar transformation by the Jacobsen and co-workers had identified that ionization to be the rate determining step.[18] Our initial NMR study also suggested a similar situation might be operative here. Therefore, we have undertaken a computational investigation of the ionization step and compare its activation barrier to that of the cyclization step. Indeed, the computed activation barrier for this reaction at the *B3LYP-D3(BJ)/def2-TZVP+CPCM(*DCM*)//PBE-D3/def2-SVP level is 13.98 kcal/mol while the activation barrier for the cycloaddition step is* ***9.56*** *kcal/mol.* (See Fig. 10). Taken altogether, this suggests that formation of the cation is the rate determining step for this transformation.

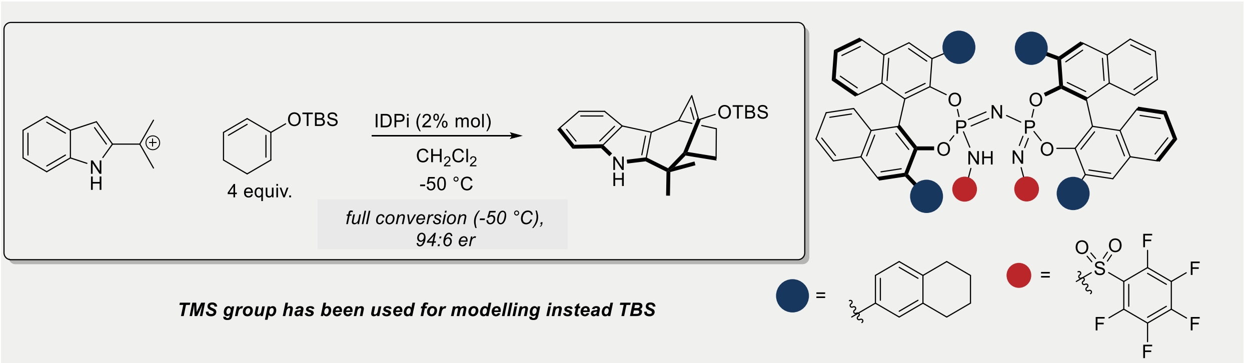


Figure 7. Summary of the overall computational approaches undertaken for this work

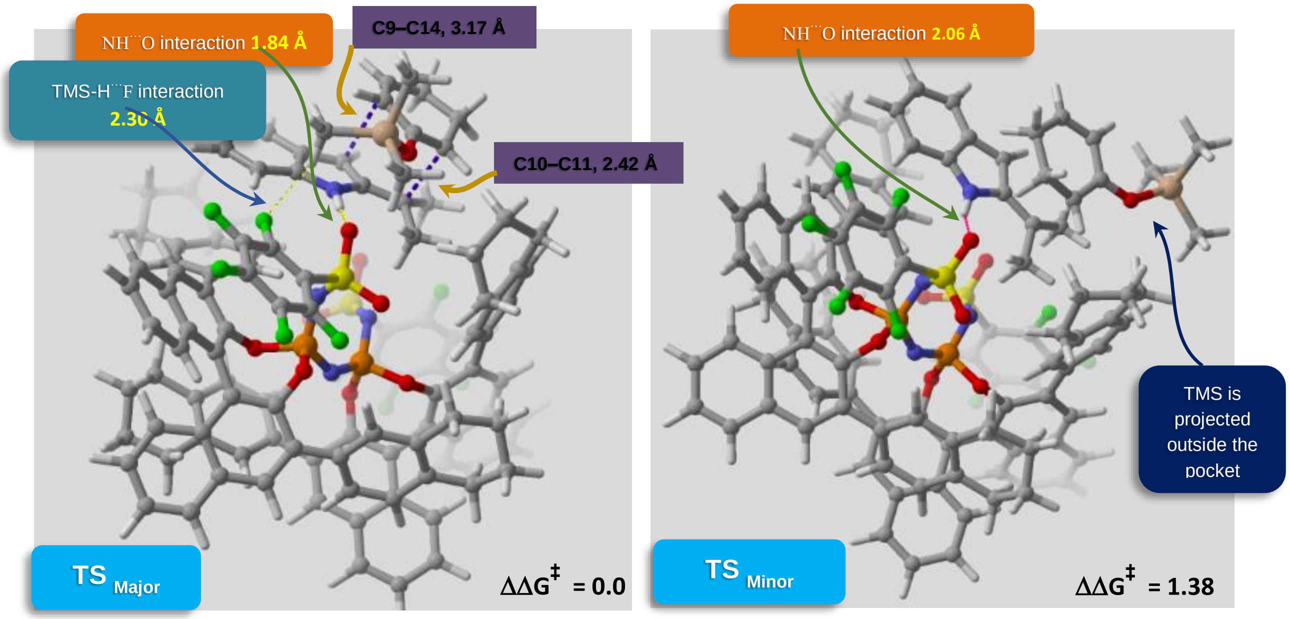


Figure 8. Stereo determining TS computed at the *B3LYP-D3(BJ)/def2-TZVP + CPCM(*DCM*)//PBE-D3/def2-SVP level of theory.* Diff in free energy (ΔG) and enthalpy difference (within parenthesis) in kcal/mol at 223K.

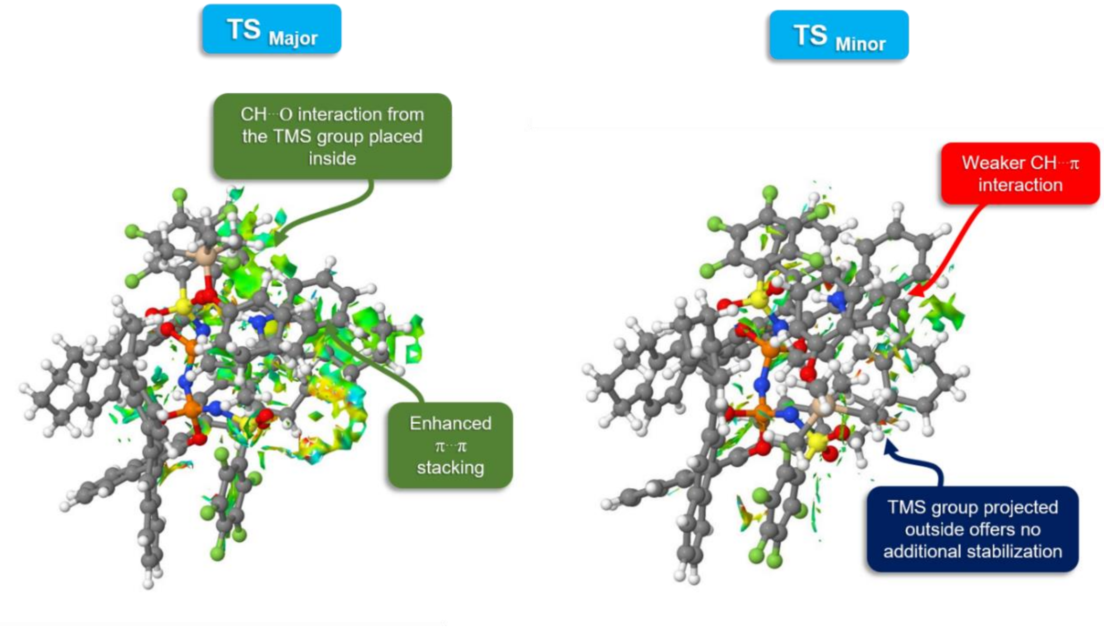


**Figure 9.** NCI plot comparison of two stereo determining transition state (isosurface value 0.3). Presence of greater stabilizing non-covalent interaction (green areas) is evident in the TS $_{\text{major}}$.

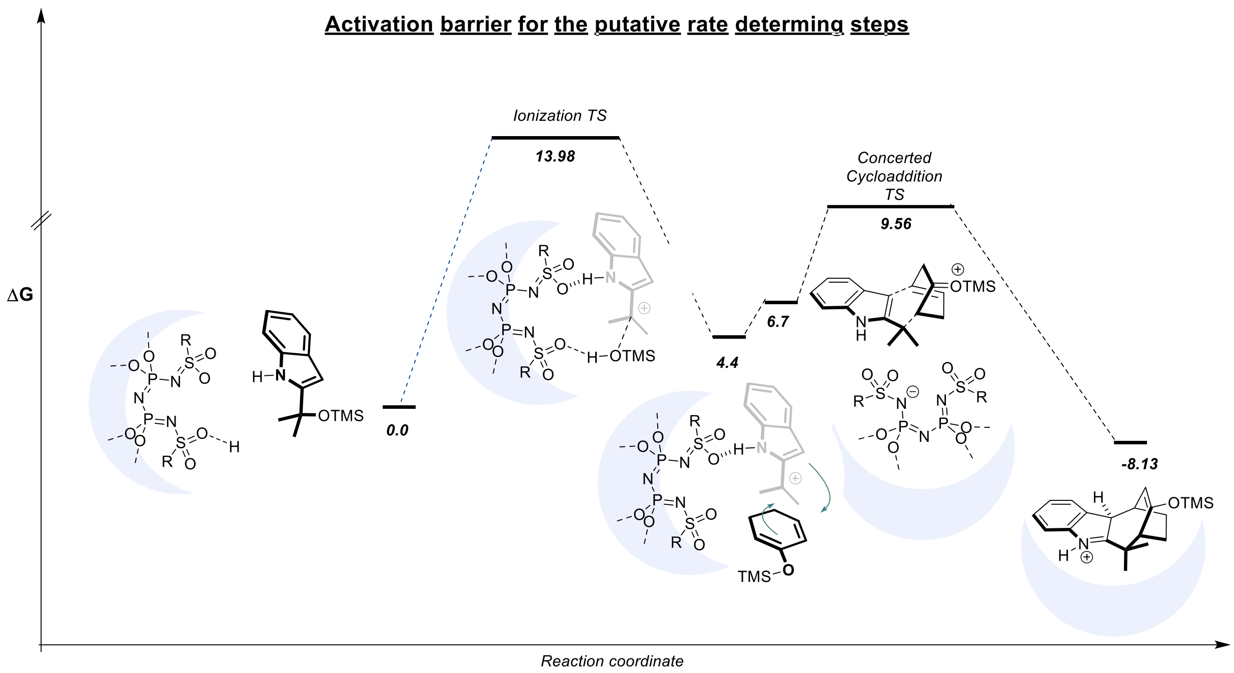


Figure 10. Computed activation energy barrier for the ionization pathway and the cycloaddition pathway at the *B3LYP-D3-(BJ)/def2-TZVP + CPCM(DCM)//PBE-D3/def2-SVP level of theory.*

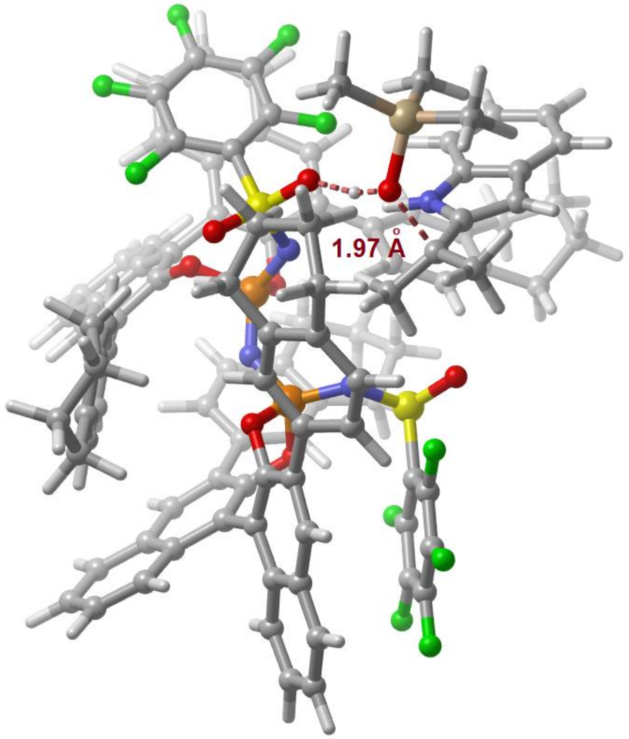


Figure 11. Optimized TS corresponding to the ionization pathway ($\Delta\Delta G^{\ddagger}$= 13.98 kcal/mol) at the *B3LYP-D3-(BJ)/def2-TZVP + CPCM(DCM)//PBE-D3/def2-SVP level of theory.*

**Table 3.** Nucleophilic SKA addition energetics leading to the TS Major at the *B3LYP-D3-(BJ)/def2-TZVP + CPCM(DCM)//PBE-D3/def2-SVP level of theory*

| TS No | PBE RRHO corrections | *B3LYP-D3/def2-TZVP* single point (DCM solv) | *Imaginary Freq.* | Δ G(TS) Final | Relative TS Energy (kcal/mol) |
|---|---|---|---|---|---|
| Major Isomer | | | | | |
| TS-A1 | 0.15539375 | -7985.92546 | -137.65 | -7985.7701 | 0.0 |
| TS-A2 | 0.15374280 | -7985.9205889 | -149.39 | -7985.7668 | 2.07 |
| TS-A3 | 0.15589373 | -7985.91329 | -238.52 | -7985.7577 | 7.78 |
| TS-A4 | 0.15591183 | -7985.91297217 | -238.76 | -7985.7568 | 8.22 |
| TS-A5 | 0.15286251 | -7985.9073418 | -235.94 | -7985.7545 | 9.78 |
| TS-A6 | 0.15524679 | -7985.9071531 | -240.10 | -7985.7515 | 11.67 |

**Table 4.** Nucleophilic SKA addition energetics leading to the TS Minor at the *B3LYP-D3(BJ)/def2-TZVP + CPCM(DCM)//PBE-D3/def2-SVP level of theory*

| TS No | PBE RRHO corrections | *B3LYP-D3(BJ)/def2-TZVP* single point (DCM solv) | *Imaginary Freq.* | Δ G(TS) Final | Relative TS Energy (kcal/mol) |
|---|---|---|---|---|---|
| Minor Isomer | | | | | |
| TS-B1 | 0.15392019 | -7985.92187 | -132.08 | -7985.7679 | 1.38 |
| TS-B2 | 0.15576248 | -7985.92243261 | -155.98 | -7985.7667 | 2.13 |
| TS-B3 | 0.15557145 | -7985.91672078 | -180.71 | -7985.7612 | 5.56 |
| TS-B4 | 0.15662907 | -7985.91572128 | -154.29 | -7985.7591 | 6.90 |
| TS-B5 | 0.15417433 | -7985.91017287 | -189.40 | -7985.756 | 9.51 |
| TS-B6 | 0.15385802 | -7985.908294 | -160.22 | -7985.7543 | 9.9 |
| TS-B7 | 0.15666907 | -7985.9095311 | -157.71 | -7985.7529 | 10.8 |

Based on the optimized structures, predicted selectivity (*er*) at the *B3LYP-D3(BJ)/def2-TZVP+ CPCM(DCM)//PBE-D3/def2-SVP level of theory* at 223 K (*er*) 95.7:4.3, provides an excellent agreement to the experimentally observed enantioselectivity (*er*) 94:6.

**Table 5.** Distortion-Interaction analysis to understand reason behind stereoselectivity.

| TS No | *B3LYP-D3(BJ)/def2-TZVP* single point (gas phase) | Relative Energy (kcal/mol) Δ E |
|---|---|---|
| TS-A1 | -7985.891522210371 | 5.20 kcal |
| TS-B1 | -7985.883248193860 | |
| Substrates Only | | |
| Subst_TS-A1 | -1198.108260497917 | -0.56 kcal/mol |
| Subst_TS-B1 | -1198.109176741957 | |

| Catalyst counteranion Only | | |
|---|---|---|
| Cat_TS-A1 | -6787.616702983154 | -2.32 kcal/mol |
| Cat_TS-B1 | -6787.620434006118 | |
| Total Distortion | | -2.88 kcal/mol |
| Total Interaction | | 7.1 kcal |

**Table 6.** TS energetics at the *B3LYP/def2-TZVP + CPCM(DCM)//PBE-D3/def2-SVP level of theory*

| TS No | PBE RRHO corrections | *B3LYP/def2-TZVP* single point (DCM solv) | *Imaginary Freq.* | Δ G(TS) Final | Relative TS Energy (kcal/mol) |
|---|---|---|---|---|---|
| (1) Only lowest energy TS energies have been computed | | | | | |
| TS-A1 | 0.15539375 | -7985.108729305454 | -137.65 | -7984.9534 | 4.31 |
| TS-B1 | 0.15392019 | -7985.11423407592 | -132.08 | -7984.9603 | 0.0 |

In absence of any dispersion correction, predicted selectivity (*e.r*) at the *B3LYP-D3/def2-TZVP+ CPCM(DCM)//PBE-D3/def2-SVP level of theory* at 223 K is opposite to that of experimentally observed value. This highlights the importance of dispersion driven non-covalent interactions for these reactions.

**Table 7.** Activation barrier comparison for between the ionization and the cyclization pathway at the *B3LYP-D3(BJ)/def2-TZVP + CPCM(DCM)//PBE-D3/def2-SVP level of theory*

| Saddle point | Δ G Final | Relative free Energy (kcal/mol) |
|---|---|---|
| (A) Ionization Step | | |
| Ionization Pre-reaction complex | -7753.6218 | 0.0 |
| Ionization TS (***TS-C1***) ( img freq: -103.1) | -7753.5995 | 13.98 |
| Cycloaddition Pre-reaction complex | -7753.6148082 | 4.4 |
| (B) Cycloaddition Step | | |
| Pre-reaction complex for Cycloaddition | -7985.77465 | 6.70 |
| Cycloaddition TS | -7985.7701 | 9.56 |
| Pdt complex for Cycloaddition | -7985.7982 | -8.13 |

**12.3 Optimized Cartesian Coordinates PBE-D3/def2-SVP**
**Major ISOMER**

***TS-A1***

***Imaginary frequency = - 137.65***

| | | | |
|---|---|---|---|
| 6 | 1.682620000 | 1.369670000 | 2.230500000 |
| 6 | -1.875460000 | -0.707300000 | 0.074410000 |
| 6 | 2.587860000 | 2.327830000 | 1.714550000 |
| 6 | 1.805400000 | 0.031570000 | 1.809450000 |
| 6 | -2.272520000 | 0.444890000 | 0.786060000 |
| 6 | -1.894190000 | -1.966250000 | 0.687030000 |
| 1 | -1.591130000 | -0.631630000 | -0.981700000 |
| 6 | 3.527460000 | 1.929160000 | 0.746850000 |
| 6 | 2.776170000 | -0.365100000 | 0.889940000 |
| 1 | 1.103640000 | -0.724530000 | 2.196150000 |
| 6 | -2.277670000 | 1.812840000 | 0.201400000 |
| 6 | -2.698870000 | 0.288020000 | 2.126630000 |
| 6 | -2.287080000 | -2.110360000 | 2.025480000 |
| 1 | -1.580350000 | -2.850310000 | 0.113940000 |
| 6 | 3.643320000 | 0.592560000 | 0.311350000 |
| 1 | 4.168450000 | 2.703910000 | 0.295630000 |
| 1 | 2.831430000 | -1.420830000 | 0.608610000 |
| 6 | -1.150680000 | 2.361020000 | -0.495370000 |
| 6 | -3.360900000 | 2.664820000 | 0.417980000 |
| 6 | -2.699620000 | -0.966720000 | 2.751470000 |
| 1 | -3.001810000 | 1.176120000 | 2.704790000 |
| 6 | 4.631970000 | 0.243270000 | -0.738720000 |
| 8 | -0.067690000 | 1.525390000 | -0.758990000 |
| 6 | -1.064660000 | 3.711200000 | -0.867240000 |
| 1 | -4.244140000 | 2.273210000 | 0.944940000 |
| 6 | -3.387510000 | 4.005850000 | -0.052130000 |
| 6 | 4.380130000 | -0.771260000 | -1.718670000 |
| 6 | 5.842700000 | 0.924800000 | -0.867210000 |
| 15 | 0.148400000 | 1.131940000 | -2.348740000 |
| 6 | -2.229680000 | 4.542120000 | -0.721030000 |
| 6 | 0.236460000 | 4.267480000 | -1.332570000 |
| 6 | -4.535370000 | 4.836820000 | 0.116590000 |
| 8 | 3.208120000 | -1.508460000 | -1.635650000 |
| 6 | 5.239740000 | -1.084130000 | -2.770970000 |
| 1 | 6.106600000 | 1.688820000 | -0.120650000 |
| 6 | 6.749190000 | 0.688140000 | -1.936410000 |
| 8 | 0.287020000 | 2.614810000 | -3.053970000 |
| 7 | 1.507330000 | 0.342970000 | -2.462710000 |
| 7 | -1.235520000 | 0.485810000 | -2.880780000 |
| 6 | -2.294430000 | 5.869170000 | -1.240910000 |
| 6 | 0.871040000 | 5.364680000 | -0.642580000 |
| 6 | 0.920080000 | 3.655330000 | -2.385000000 |

| | | | |
|---|---|---|---|
| 1 | -5.413800000 | 4.418730000 | 0.631720000 |
| 6 | -4.558060000 | 6.131510000 | -0.376650000 |
| 15 | 1.972590000 | -1.160460000 | -2.665580000 |
| 6 | 4.875550000 | -2.174280000 | -3.720390000 |
| 6 | 6.448370000 | -0.319070000 | -2.924650000 |
| 6 | 7.940960000 | 1.460940000 | -2.078210000 |
| 16 | -1.964150000 | 0.842560000 | -4.253510000 |
| 6 | -3.430930000 | 6.646370000 | -1.071290000 |
| 1 | -1.430110000 | 6.267540000 | -1.790580000 |
| 6 | 0.298640000 | 6.017200000 | 0.490880000 |
| 6 | 2.176810000 | 5.793000000 | -1.083760000 |
| 6 | 2.232550000 | 4.030640000 | -2.807660000 |
| 1 | -5.454610000 | 6.756230000 | -0.249150000 |
| 8 | 2.749840000 | -1.134730000 | -4.129910000 |
| 7 | 0.948680000 | -2.399870000 | -2.597660000 |
| 6 | 3.656210000 | -2.149350000 | -4.416050000 |
| 6 | 5.774020000 | -3.278830000 | -3.931580000 |
| 6 | 7.334830000 | -0.474840000 | -4.030470000 |
| 1 | 8.164540000 | 2.222900000 | -1.315230000 |
| 6 | 8.790080000 | 1.276290000 | -3.158600000 |
| 8 | -1.077570000 | 0.968560000 | -5.431690000 |
| 8 | -3.127860000 | -0.096020000 | -4.385420000 |
| 1 | -3.465000000 | 7.662130000 | -1.491980000 |
| 1 | -0.669450000 | 5.675200000 | 0.876740000 |
| 6 | 0.954600000 | 7.062300000 | 1.125990000 |
| 6 | 2.821600000 | 6.873710000 | -0.410750000 |
| 6 | 2.820480000 | 5.107860000 | -2.149090000 |
| 6 | 2.972490000 | 3.285900000 | -3.854540000 |
| 16 | 0.943060000 | -3.486410000 | -1.386280000 |
| 6 | 3.320380000 | -3.105270000 | -5.433970000 |
| 6 | 5.475870000 | -4.226170000 | -4.973270000 |
| 6 | 6.931120000 | -3.497900000 | -3.126200000 |
| 6 | 8.476730000 | 0.305760000 | -4.147010000 |
| 1 | 7.094520000 | -1.211100000 | -4.809070000 |
| 1 | 9.699770000 | 1.887390000 | -3.258270000 |
| 1 | 0.489330000 | 7.543180000 | 1.999570000 |
| 6 | 2.223730000 | 7.503620000 | 0.668990000 |
| 1 | 3.815500000 | 7.189860000 | -0.763810000 |
| 1 | 3.821190000 | 5.440810000 | -2.463660000 |
| 6 | 2.387550000 | 2.929460000 | -5.084870000 |
| 6 | 4.325250000 | 2.945340000 | -3.631880000 |
| 8 | 1.089470000 | -2.901590000 | -0.037740000 |
| 8 | -0.199180000 | -4.407260000 | -1.607760000 |
| 6 | 4.272240000 | -4.089180000 | -5.710380000 |
| 6 | 2.068780000 | -3.081900000 | -6.237680000 |
| 6 | 6.381000000 | -5.301440000 | -5.220040000 |
| 6 | 7.778140000 | -4.568420000 | -3.373950000 |

| | | | |
|---|---|---|---|
| 1 | 7.137460000 | -2.814800000 | -2.291090000 |
| 1 | 9.139560000 | 0.175460000 | -5.015720000 |
| 1 | 2.735130000 | 8.332790000 | 1.180560000 |
| 6 | 3.119290000 | 2.263640000 | -6.085100000 |
| 1 | 1.337200000 | 3.187960000 | -5.280990000 |
| 6 | 5.051720000 | 2.264650000 | -4.610380000 |
| 1 | 4.798190000 | 3.187150000 | -2.668380000 |
| 1 | 4.081770000 | -4.799310000 | -6.527650000 |
| 6 | 1.450110000 | -1.882730000 | -6.661100000 |
| 6 | 1.529760000 | -4.305120000 | -6.705450000 |
| 1 | 6.142720000 | -6.013630000 | -6.024760000 |
| 6 | 7.516200000 | -5.465190000 | -4.443730000 |
| 1 | 8.655560000 | -4.728090000 | -2.729650000 |
| 6 | 4.466570000 | 1.903630000 | -5.839740000 |
| 1 | 6.101660000 | 1.998920000 | -4.419430000 |
| 6 | 0.403860000 | -1.894630000 | -7.598380000 |
| 1 | 1.806640000 | -0.919470000 | -6.272530000 |
| 6 | 0.468630000 | -4.323970000 | -7.618570000 |
| 1 | 1.941800000 | -5.252580000 | -6.330530000 |
| 1 | 8.198720000 | -6.307280000 | -4.631930000 |
| 6 | -0.085750000 | -3.125240000 | -8.100390000 |
| 1 | 0.070150000 | -5.289110000 | -7.972330000 |
| 6 | -2.988940000 | 4.854790000 | -4.521200000 |
| 6 | -4.182370000 | 4.907110000 | -3.785880000 |
| 6 | -2.285490000 | 3.642510000 | -4.648210000 |
| 6 | -4.652440000 | 3.755080000 | -3.136480000 |
| 6 | -2.756730000 | 2.473410000 | -4.020540000 |
| 6 | -3.913510000 | 2.568430000 | -3.225120000 |
| 6 | 2.500590000 | -5.290480000 | -2.855190000 |
| 6 | 2.446510000 | -4.492950000 | -1.698370000 |
| 6 | 3.596140000 | -6.119270000 | -3.129340000 |
| 6 | 3.553040000 | -4.512520000 | -0.828310000 |
| 6 | 4.677860000 | -6.153120000 | -2.236110000 |
| 6 | 4.668220000 | -5.325850000 | -1.103870000 |
| 9 | 3.617450000 | -3.767790000 | 0.273680000 |
| 9 | 5.721230000 | -5.321270000 | -0.281820000 |
| 9 | 5.719280000 | -6.949890000 | -2.472100000 |
| 9 | 3.621000000 | -6.860680000 | -4.244630000 |
| 9 | 1.503440000 | -5.277320000 | -3.749860000 |
| 9 | -4.862810000 | 6.049120000 | -3.699880000 |
| 9 | -4.370980000 | 1.514120000 | -2.536820000 |
| 9 | -1.180720000 | 3.664400000 | -5.390820000 |
| 9 | -5.785870000 | 3.794170000 | -2.431600000 |
| 9 | -2.526050000 | 5.961720000 | -5.107370000 |
| 6 | -2.250850000 | -3.421140000 | 2.765960000 |
| 6 | -3.517570000 | -3.673490000 | 3.618960000 |
| 1 | -4.288390000 | -4.149610000 | 2.980470000 |

| | | | |
|---|---|---|---|
| 1 | -3.271290000 | -4.411600000 | 4.409730000 |
| 6 | 5.261750000 | 1.111350000 | -6.857010000 |
| 6 | 4.721450000 | 1.251020000 | -8.283380000 |
| 1 | 5.250570000 | 0.550930000 | -8.962610000 |
| 1 | 4.927080000 | 2.277540000 | -8.661420000 |
| 6 | -1.176390000 | -3.061180000 | -9.137740000 |
| 6 | -2.304800000 | -2.078870000 | -8.749050000 |
| 1 | -2.918680000 | -1.872340000 | -9.650300000 |
| 1 | -2.984030000 | -2.571780000 | -8.024620000 |
| 6 | 0.591240000 | 1.763440000 | 3.203030000 |
| 6 | 0.300630000 | 3.266050000 | 3.187170000 |
| 1 | -0.190590000 | 3.536300000 | 2.225660000 |
| 1 | -0.414020000 | 3.528940000 | 3.994720000 |
| 6 | 1.607180000 | 4.051750000 | 3.330960000 |
| 1 | 2.104910000 | 3.748490000 | 4.279200000 |
| 1 | 1.413300000 | 5.141500000 | 3.409010000 |
| 6 | 2.533850000 | 3.782060000 | 2.140790000 |
| 1 | 3.562190000 | 4.140860000 | 2.359140000 |
| 1 | 2.192530000 | 4.384690000 | 1.274290000 |
| 1 | -0.322970000 | 1.177010000 | 2.974240000 |
| 1 | 0.894970000 | 1.460640000 | 4.232230000 |
| 6 | -3.135280000 | -1.188140000 | 4.177780000 |
| 1 | -2.244220000 | -1.401200000 | 4.812720000 |
| 1 | -3.603710000 | -0.273430000 | 4.594810000 |
| 6 | -4.105780000 | -2.383780000 | 4.248510000 |
| 1 | -4.406300000 | -2.566850000 | 5.300630000 |
| 1 | -5.029480000 | -2.093990000 | 3.704920000 |
| 1 | -2.072170000 | -4.258970000 | 2.063060000 |
| 1 | -1.366440000 | -3.399830000 | 3.442850000 |
| 6 | -0.243350000 | -0.641010000 | -8.125430000 |
| 1 | 0.120290000 | -0.457420000 | -9.163130000 |
| 1 | 0.053070000 | 0.232750000 | -7.516330000 |
| 6 | -1.778040000 | -0.762050000 | -8.123250000 |
| 1 | -2.108260000 | -0.687430000 | -7.070460000 |
| 1 | -2.209980000 | 0.118880000 | -8.639080000 |
| 1 | -1.583970000 | -4.072260000 | -9.344560000 |
| 1 | -0.720660000 | -2.713170000 | -10.093060000 |
| 1 | 6.332660000 | 1.397750000 | -6.799630000 |
| 1 | 5.227490000 | 0.036730000 | -6.559780000 |
| 6 | 2.470920000 | 2.011410000 | -7.433130000 |
| 1 | 2.425490000 | 2.984360000 | -7.974680000 |
| 1 | 1.412800000 | 1.714010000 | -7.281340000 |
| 6 | 3.212090000 | 0.991570000 | -8.303640000 |
| 1 | 3.020020000 | -0.036310000 | -7.927520000 |
| 1 | 2.816010000 | 1.021420000 | -9.339970000 |
| 6 | -5.138880000 | -3.983920000 | 0.102310000 |
| 6 | -5.753020000 | -2.911290000 | 0.742280000 |

| | | | |
|---|---|---|---|
| 6 | -5.656940000 | -1.591800000 | 0.218830000 |
| 6 | -4.418550000 | -3.739710000 | -1.095080000 |
| 6 | -4.347330000 | -2.398420000 | -1.617180000 |
| 6 | -4.961120000 | -1.315300000 | -0.958200000 |
| 6 | -3.672000000 | -4.557690000 | -1.982940000 |
| 6 | -3.144320000 | -3.722470000 | -2.995530000 |
| 7 | -3.590050000 | -2.424840000 | -2.761930000 |
| 6 | -2.295560000 | -4.060200000 | -4.095840000 |
| 6 | -1.650800000 | -5.408360000 | -4.120320000 |
| 1 | -2.293180000 | -6.225010000 | -3.747600000 |
| 1 | -0.781870000 | -5.329320000 | -3.429730000 |
| 1 | -1.261580000 | -5.655240000 | -5.125720000 |
| 6 | -1.557140000 | -2.967060000 | -4.787130000 |
| 1 | -1.069030000 | -3.318900000 | -5.711620000 |
| 1 | -0.741070000 | -2.644870000 | -4.091040000 |
| 1 | -2.179510000 | -2.080690000 | -5.004450000 |
| 1 | -3.336820000 | -1.585630000 | -3.320010000 |
| 6 | -4.238250000 | -5.969580000 | -5.780420000 |
| 6 | -5.115820000 | -6.324340000 | -4.563560000 |
| 6 | -3.873300000 | -4.508800000 | -5.878530000 |
| 1 | -4.787100000 | -6.229450000 | -6.715750000 |
| 1 | -3.329520000 | -6.602610000 | -5.796770000 |
| 6 | -4.813220000 | -3.554310000 | -5.502450000 |
| 6 | -5.968010000 | -5.188110000 | -4.080530000 |
| 1 | -4.499670000 | -6.674780000 | -3.707430000 |
| 1 | -5.767480000 | -7.192210000 | -4.802050000 |
| 6 | -5.870290000 | -3.922740000 | -4.567150000 |
| 1 | -3.119130000 | -4.212570000 | -6.621800000 |
| 8 | -4.673140000 | -2.289100000 | -5.900500000 |
| 1 | -6.705710000 | -5.402080000 | -3.289980000 |
| 1 | -6.519840000 | -3.129980000 | -4.172130000 |
| 14 | -5.879050000 | -1.031850000 | -6.083090000 |
| 6 | -7.304310000 | -1.838780000 | -7.016870000 |
| 6 | -6.427910000 | -0.342450000 | -4.425900000 |
| 1 | -6.937040000 | -1.076380000 | -3.771220000 |
| 1 | -7.139250000 | 0.490460000 | -4.609310000 |
| 1 | -5.555840000 | 0.067140000 | -3.883930000 |
| 1 | -6.951180000 | -2.266560000 | -7.977240000 |
| 1 | -8.082540000 | -1.082100000 | -7.247910000 |
| 1 | -7.785240000 | -2.649970000 | -6.434390000 |
| 1 | -6.312920000 | -3.077490000 | 1.674660000 |
| 1 | -6.131720000 | -0.763710000 | 0.766090000 |
| 1 | -5.199430000 | -5.001740000 | 0.516460000 |
| 1 | -4.867060000 | -0.292410000 | -1.339100000 |
| 1 | -3.467260000 | -5.627000000 | -1.872150000 |
| 6 | -5.030050000 | 0.273050000 | -7.115900000 |
| 1 | -4.741750000 | -0.121640000 | -8.109570000 |

| | | | |
|---|---|---|---|
| 1 | -5.712680000 | 1.134510000 | -7.267150000 |
| 1 | -4.119490000 | 0.625890000 | -6.596100000 |

***TS-A2***

***Imaginary frequency = - 149.39***

| | | | |
|---|---|---|---|
| 6 | 1.682622000 | 1.369672000 | 2.230497000 |
| 6 | -1.875461000 | -0.707296000 | 0.074408000 |
| 6 | 2.587861000 | 2.327831000 | 1.714552000 |
| 6 | 1.805395000 | 0.031569000 | 1.809452000 |
| 6 | -2.272518000 | 0.444891000 | 0.786057000 |
| 6 | -1.894186000 | -1.966253000 | 0.687030000 |
| 1 | -1.591127000 | -0.631629000 | -0.981699000 |
| 6 | 3.527465000 | 1.929164000 | 0.746853000 |
| 6 | 2.776174000 | -0.365097000 | 0.889941000 |
| 1 | 1.103640000 | -0.724528000 | 2.196147000 |
| 6 | -2.277666000 | 1.812838000 | 0.201402000 |
| 6 | -2.698870000 | 0.288015000 | 2.126630000 |
| 6 | -2.287084000 | -2.110360000 | 2.025479000 |
| 1 | -1.580352000 | -2.850308000 | 0.113936000 |
| 6 | 3.643317000 | 0.592564000 | 0.311348000 |
| 1 | 4.168454000 | 2.703909000 | 0.295633000 |
| 1 | 2.831428000 | -1.420827000 | 0.608611000 |
| 6 | -1.150676000 | 2.361023000 | -0.495366000 |
| 6 | -3.360896000 | 2.664815000 | 0.417982000 |
| 6 | -2.699618000 | -0.966716000 | 2.751465000 |
| 1 | -3.001810000 | 1.176120000 | 2.704791000 |
| 6 | 4.631973000 | 0.243272000 | -0.738722000 |
| 8 | -0.067689000 | 1.525385000 | -0.758992000 |
| 6 | -1.064662000 | 3.711195000 | -0.867241000 |
| 1 | -4.244136000 | 2.273209000 | 0.944939000 |
| 6 | -3.387512000 | 4.005853000 | -0.052131000 |
| 6 | 4.380126000 | -0.771256000 | -1.718670000 |
| 6 | 5.842704000 | 0.924801000 | -0.867213000 |
| 15 | 0.148399000 | 1.131937000 | -2.348743000 |
| 6 | -2.229680000 | 4.542122000 | -0.721033000 |
| 6 | 0.236461000 | 4.267481000 | -1.332568000 |
| 6 | -4.535371000 | 4.836822000 | 0.116591000 |
| 8 | 3.208117000 | -1.508459000 | -1.635653000 |
| 6 | 5.239740000 | -1.084134000 | -2.770975000 |
| 1 | 6.106598000 | 1.688818000 | -0.120649000 |
| 6 | 6.749195000 | 0.688139000 | -1.936407000 |
| 8 | 0.287016000 | 2.614810000 | -3.053969000 |
| 7 | 1.507330000 | 0.342973000 | -2.462705000 |
| 7 | -1.235518000 | 0.485813000 | -2.880780000 |

| | | | |
|---|---|---|---|
| 6 | -2.294429000 | 5.869167000 | -1.240915000 |
| 6 | 0.871039000 | 5.364678000 | -0.642583000 |
| 6 | 0.920076000 | 3.655333000 | -2.385001000 |
| 1 | -5.413797000 | 4.418729000 | 0.631724000 |
| 6 | -4.558060000 | 6.131513000 | -0.376654000 |
| 15 | 1.972591000 | -1.160458000 | -2.665575000 |
| 6 | 4.875554000 | -2.174279000 | -3.720391000 |
| 6 | 6.448374000 | -0.319074000 | -2.924649000 |
| 6 | 7.940957000 | 1.460939000 | -2.078215000 |
| 16 | -1.964146000 | 0.842558000 | -4.253510000 |
| 6 | -3.430929000 | 6.646370000 | -1.071288000 |
| 1 | -1.430110000 | 6.267544000 | -1.790581000 |
| 6 | 0.298640000 | 6.017198000 | 0.490881000 |
| 6 | 2.176815000 | 5.793002000 | -1.083761000 |
| 6 | 2.232553000 | 4.030639000 | -2.807655000 |
| 1 | -5.454605000 | 6.756225000 | -0.249152000 |
| 8 | 2.749837000 | -1.134732000 | -4.129906000 |
| 7 | 0.948679000 | -2.399867000 | -2.597662000 |
| 6 | 3.656210000 | -2.149353000 | -4.416045000 |
| 6 | 5.774023000 | -3.278830000 | -3.931579000 |
| 6 | 7.334834000 | -0.474845000 | -4.030471000 |
| 1 | 8.164542000 | 2.222901000 | -1.315230000 |
| 6 | 8.790076000 | 1.276294000 | -3.158598000 |
| 8 | -1.077572000 | 0.968557000 | -5.431687000 |
| 8 | -3.127860000 | -0.096023000 | -4.385415000 |
| 1 | -3.465000000 | 7.662133000 | -1.491977000 |
| 1 | -0.669449000 | 5.675196000 | 0.876740000 |
| 6 | 0.954601000 | 7.062296000 | 1.125988000 |
| 6 | 2.821596000 | 6.873711000 | -0.410751000 |
| 6 | 2.820482000 | 5.107855000 | -2.149091000 |
| 6 | 2.972493000 | 3.285901000 | -3.854537000 |
| 16 | 0.943055000 | -3.486409000 | -1.386278000 |
| 6 | 3.320378000 | -3.105266000 | -5.433965000 |
| 6 | 5.475873000 | -4.226169000 | -4.973271000 |
| 6 | 6.931116000 | -3.497903000 | -3.126202000 |
| 6 | 8.476729000 | 0.305762000 | -4.147014000 |
| 1 | 7.094518000 | -1.211105000 | -4.809074000 |
| 1 | 9.699771000 | 1.887389000 | -3.258267000 |
| 1 | 0.489330000 | 7.543182000 | 1.999571000 |
| 6 | 2.223734000 | 7.503618000 | 0.668987000 |
| 1 | 3.815500000 | 7.189856000 | -0.763807000 |
| 1 | 3.821192000 | 5.440807000 | -2.463657000 |
| 6 | 2.387548000 | 2.929458000 | -5.084868000 |
| 6 | 4.325250000 | 2.945343000 | -3.631884000 |
| 8 | 1.089470000 | -2.901587000 | -0.037745000 |
| 8 | -0.199180000 | -4.407256000 | -1.607761000 |
| 6 | 4.272243000 | -4.089176000 | -5.710380000 |

| | | | |
|---|---|---|---|
| 6 | 2.068775000 | -3.081901000 | -6.237683000 |
| 6 | 6.381000000 | -5.301437000 | -5.220036000 |
| 6 | 7.778142000 | -4.568424000 | -3.373946000 |
| 1 | 7.137463000 | -2.814797000 | -2.291093000 |
| 1 | 9.139556000 | 0.175455000 | -5.015717000 |
| 1 | 2.735130000 | 8.332787000 | 1.180559000 |
| 6 | 3.119287000 | 2.263643000 | -6.085098000 |
| 1 | 1.337205000 | 3.187963000 | -5.280988000 |
| 6 | 5.051718000 | 2.264652000 | -4.610383000 |
| 1 | 4.798186000 | 3.187150000 | -2.668377000 |
| 1 | 4.081772000 | -4.799309000 | -6.527645000 |
| 6 | 1.450115000 | -1.882726000 | -6.661104000 |
| 6 | 1.529760000 | -4.305121000 | -6.705450000 |
| 1 | 6.142716000 | -6.013625000 | -6.024762000 |
| 6 | 7.516204000 | -5.465193000 | -4.443732000 |
| 1 | 8.655557000 | -4.728086000 | -2.729654000 |
| 6 | 4.466573000 | 1.903630000 | -5.839741000 |
| 1 | 6.101664000 | 1.998921000 | -4.419428000 |
| 6 | 0.403857000 | -1.894626000 | -7.598376000 |
| 1 | 1.806638000 | -0.919469000 | -6.272527000 |
| 6 | 0.468633000 | -4.323973000 | -7.618569000 |
| 1 | 1.941804000 | -5.252577000 | -6.330526000 |
| 1 | 8.198717000 | -6.307278000 | -4.631934000 |
| 6 | -0.085754000 | -3.125240000 | -8.100386000 |
| 1 | 0.070152000 | -5.289108000 | -7.972328000 |
| 6 | -2.988939000 | 4.854792000 | -4.521198000 |
| 6 | -4.182372000 | 4.907108000 | -3.785879000 |
| 6 | -2.285492000 | 3.642515000 | -4.648209000 |
| 6 | -4.652440000 | 3.755076000 | -3.136484000 |
| 6 | -2.756732000 | 2.473414000 | -4.020540000 |
| 6 | -3.913514000 | 2.568431000 | -3.225116000 |
| 6 | 2.500594000 | -5.290475000 | -2.855186000 |
| 6 | 2.446511000 | -4.492954000 | -1.698374000 |
| 6 | 3.596141000 | -6.119271000 | -3.129335000 |
| 6 | 3.553037000 | -4.512524000 | -0.828310000 |
| 6 | 4.677860000 | -6.153119000 | -2.236110000 |
| 6 | 4.668221000 | -5.325850000 | -1.103868000 |
| 9 | 3.617449000 | -3.767788000 | 0.273677000 |
| 9 | 5.721225000 | -5.321274000 | -0.281817000 |
| 9 | 5.719278000 | -6.949886000 | -2.472095000 |
| 9 | 3.621001000 | -6.860683000 | -4.244626000 |
| 9 | 1.503440000 | -5.277320000 | -3.749856000 |
| 9 | -4.862813000 | 6.049120000 | -3.699884000 |
| 9 | -4.370977000 | 1.514119000 | -2.536815000 |
| 9 | -1.180723000 | 3.664400000 | -5.390824000 |
| 9 | -5.785873000 | 3.794174000 | -2.431603000 |
| 9 | -2.526055000 | 5.961721000 | -5.107368000 |

| | | | |
|---|---|---|---|
| 6 | -2.250845000 | -3.421141000 | 2.765961000 |
| 6 | -3.517575000 | -3.673490000 | 3.618960000 |
| 1 | -4.288391000 | -4.149614000 | 2.980471000 |
| 1 | -3.271292000 | -4.411598000 | 4.409735000 |
| 6 | 5.261749000 | 1.111350000 | -6.857013000 |
| 6 | 4.721447000 | 1.251021000 | -8.283380000 |
| 1 | 5.250566000 | 0.550933000 | -8.962608000 |
| 1 | 4.927079000 | 2.277537000 | -8.661423000 |
| 6 | -1.176386000 | -3.061184000 | -9.137740000 |
| 6 | -2.304799000 | -2.078874000 | -8.749055000 |
| 1 | -2.918678000 | -1.872337000 | -9.650301000 |
| 1 | -2.984026000 | -2.571780000 | -8.024615000 |
| 6 | 0.591235000 | 1.763442000 | 3.203030000 |
| 6 | 0.300629000 | 3.266049000 | 3.187167000 |
| 1 | -0.190594000 | 3.536301000 | 2.225661000 |
| 1 | -0.414019000 | 3.528942000 | 3.994724000 |
| 6 | 1.607178000 | 4.051754000 | 3.330960000 |
| 1 | 2.104910000 | 3.748491000 | 4.279205000 |
| 1 | 1.413304000 | 5.141501000 | 3.409011000 |
| 6 | 2.533853000 | 3.782056000 | 2.140786000 |
| 1 | 3.562194000 | 4.140863000 | 2.359138000 |
| 1 | 2.192532000 | 4.384693000 | 1.274288000 |
| 1 | -0.322973000 | 1.177009000 | 2.974236000 |
| 1 | 0.894971000 | 1.460636000 | 4.232231000 |
| 6 | -3.135282000 | -1.188137000 | 4.177775000 |
| 1 | -2.244220000 | -1.401203000 | 4.812722000 |
| 1 | -3.603713000 | -0.273425000 | 4.594805000 |
| 6 | -4.105785000 | -2.383778000 | 4.248515000 |
| 1 | -4.406296000 | -2.566849000 | 5.300630000 |
| 1 | -5.029476000 | -2.093993000 | 3.704918000 |
| 1 | -2.072172000 | -4.258966000 | 2.063058000 |
| 1 | -1.366437000 | -3.399833000 | 3.442851000 |
| 6 | -0.243350000 | -0.641013000 | -8.125430000 |
| 1 | 0.120289000 | -0.457424000 | -9.163126000 |
| 1 | 0.053072000 | 0.232749000 | -7.516325000 |
| 6 | -1.778043000 | -0.762050000 | -8.123252000 |
| 1 | -2.108261000 | -0.687433000 | -7.070459000 |
| 1 | -2.209982000 | 0.118876000 | -8.639081000 |
| 1 | -1.583966000 | -4.072260000 | -9.344558000 |
| 1 | -0.720660000 | -2.713166000 | -10.093056000 |
| 1 | 6.332658000 | 1.397746000 | -6.799628000 |
| 1 | 5.227486000 | 0.036733000 | -6.559780000 |
| 6 | 2.470922000 | 2.011410000 | -7.433127000 |
| 1 | 2.425491000 | 2.984356000 | -7.974679000 |
| 1 | 1.412796000 | 1.714013000 | -7.281343000 |
| 6 | 3.212086000 | 0.991568000 | -8.303641000 |
| 1 | 3.020021000 | -0.036312000 | -7.927524000 |

| | | | |
|---|---|---|---|
| 1 | 2.816006000 | 1.021424000 | -9.339966000 |
| 6 | -5.138883000 | -3.983922000 | 0.102310000 |
| 6 | -5.753022000 | -2.911290000 | 0.742279000 |
| 6 | -5.656937000 | -1.591796000 | 0.218829000 |
| 6 | -4.418549000 | -3.739706000 | -1.095080000 |
| 6 | -4.347325000 | -2.398417000 | -1.617183000 |
| 6 | -4.961122000 | -1.315304000 | -0.958202000 |
| 6 | -3.671999000 | -4.557686000 | -1.982944000 |
| 6 | -3.144322000 | -3.722466000 | -2.995534000 |
| 7 | -3.590053000 | -2.424844000 | -2.761933000 |
| 6 | -2.295560000 | -4.060202000 | -4.095845000 |
| 6 | -1.650803000 | -5.408360000 | -4.120323000 |
| 1 | -2.293177000 | -6.225011000 | -3.747599000 |
| 1 | -0.781871000 | -5.329315000 | -3.429732000 |
| 1 | -1.261578000 | -5.655239000 | -5.125719000 |
| 6 | -1.557138000 | -2.967060000 | -4.787135000 |
| 1 | -1.069032000 | -3.318900000 | -5.711623000 |
| 1 | -0.741070000 | -2.644866000 | -4.091043000 |
| 1 | -2.179514000 | -2.080690000 | -5.004454000 |
| 1 | -3.336820000 | -1.585632000 | -3.320014000 |
| 6 | -4.238247000 | -5.969581000 | -5.780417000 |
| 6 | -5.115817000 | -6.324336000 | -4.563560000 |
| 6 | -3.873303000 | -4.508803000 | -5.878533000 |
| 1 | -4.787101000 | -6.229446000 | -6.715749000 |
| 1 | -3.329523000 | -6.602613000 | -5.796774000 |
| 6 | -4.813216000 | -3.554310000 | -5.502448000 |
| 6 | -5.968007000 | -5.188108000 | -4.080533000 |
| 1 | -4.499672000 | -6.674777000 | -3.707428000 |
| 1 | -5.767485000 | -7.192210000 | -4.802053000 |
| 6 | -5.870287000 | -3.922743000 | -4.567146000 |
| 1 | -3.119132000 | -4.212568000 | -6.621796000 |
| 8 | -4.673136000 | -2.289102000 | -5.900501000 |
| 1 | -6.705707000 | -5.402084000 | -3.289982000 |
| 1 | -6.519843000 | -3.129985000 | -4.172125000 |
| 14 | -5.879047000 | -1.031845000 | -6.083094000 |
| 6 | -7.304308000 | -1.838784000 | -7.016875000 |
| 6 | -6.427912000 | -0.342452000 | -4.425896000 |
| 1 | -6.937043000 | -1.076383000 | -3.771222000 |
| 1 | -7.139249000 | 0.490456000 | -4.609311000 |
| 1 | -5.555837000 | 0.067137000 | -3.883930000 |
| 1 | -6.951181000 | -2.266560000 | -7.977244000 |
| 1 | -8.082541000 | -1.082100000 | -7.247910000 |
| 1 | -7.785236000 | -2.649970000 | -6.434389000 |
| 1 | -6.312923000 | -3.077490000 | 1.674661000 |
| 1 | -6.131722000 | -0.763706000 | 0.766086000 |
| 1 | -5.199435000 | -5.001744000 | 0.516460000 |
| 1 | -4.867058000 | -0.292407000 | -1.339100000 |

| | | | |
|---|---|---|---|
| 1 | -3.467259000 | -5.626999000 | -1.872146000 |
| 6 | -5.030047000 | 0.273054000 | -7.115904000 |
| 1 | -4.741750000 | -0.121639000 | -8.109573000 |
| 1 | -5.712684000 | 1.134506000 | -7.267149000 |
| 1 | -4.119487000 | 0.625894000 | -6.596097000 |

***TS-A3***

***Imaginary frequency = -238.52***

| | | | |
|---|---|---|---|
| 6 | 2.416250000 | 2.088312000 | 2.068854000 |
| 6 | -3.965729000 | -0.487044000 | 0.737635000 |
| 6 | 2.831584000 | 0.736178000 | 2.004407000 |
| 6 | 2.987839000 | 3.015140000 | 1.175173000 |
| 6 | -2.666601000 | 0.071761000 | 0.652189000 |
| 6 | -4.164189000 | -1.796482000 | 1.189235000 |
| 1 | -4.833275000 | 0.104841000 | 0.412626000 |
| 6 | 3.754366000 | 0.342388000 | 1.018098000 |
| 6 | 3.901231000 | 2.615359000 | 0.198137000 |
| 1 | 2.684616000 | 4.071182000 | 1.233663000 |
| 6 | -2.509719000 | 1.493091000 | 0.243193000 |
| 6 | -1.574851000 | -0.759297000 | 1.001143000 |
| 6 | -3.073290000 | -2.611841000 | 1.530674000 |
| 1 | -5.185823000 | -2.205239000 | 1.243618000 |
| 6 | 4.284561000 | 1.259168000 | 0.087400000 |
| 1 | 4.047283000 | -0.714550000 | 0.973760000 |
| 1 | 4.281241000 | 3.352710000 | -0.523782000 |
| 6 | -1.318034000 | 2.025077000 | -0.358150000 |
| 6 | -3.550740000 | 2.401927000 | 0.451196000 |
| 6 | -1.766463000 | -2.084166000 | 1.417811000 |
| 1 | -0.550698000 | -0.373398000 | 0.942987000 |
| 6 | 5.172127000 | 0.854191000 | -1.030854000 |
| 8 | -0.229787000 | 1.184968000 | -0.542907000 |
| 6 | -1.170405000 | 3.367977000 | -0.728032000 |
| 1 | -4.461111000 | 2.065112000 | 0.966667000 |
| 6 | -3.498944000 | 3.750864000 | 0.014904000 |
| 6 | 4.903270000 | -0.311291000 | -1.816378000 |
| 6 | 6.248726000 | 1.638433000 | -1.436147000 |
| 15 | 0.172279000 | 0.723450000 | -2.078050000 |
| 6 | -2.306262000 | 4.244796000 | -0.622616000 |
| 6 | 0.145513000 | 3.887402000 | -1.195485000 |
| 6 | -4.617374000 | 4.624801000 | 0.168710000 |
| 8 | 3.897908000 | -1.162168000 | -1.384473000 |
| 6 | 5.592796000 | -0.658686000 | -2.980226000 |
| 1 | 6.515856000 | 2.525962000 | -0.842582000 |
| 6 | 6.998809000 | 1.352844000 | -2.609220000 |
| 8 | 0.220484000 | 2.178903000 | -2.853785000 |

| | | | |
|---|---|---|---|
| 7 | 1.621368000 | 0.115388000 | -1.871173000 |
| 7 | -0.995309000 | -0.136419000 | -2.762362000 |
| 6 | -2.312485000 | 5.571925000 | -1.145661000 |
| 6 | 0.771584000 | 4.999634000 | -0.528660000 |
| 6 | 0.811775000 | 3.286807000 | -2.267227000 |
| 1 | -5.521581000 | 4.237026000 | 0.662338000 |
| 6 | -4.578847000 | 5.921012000 | -0.317060000 |
| 15 | 2.436310000 | -1.202290000 | -2.130407000 |
| 6 | 5.186876000 | -1.862064000 | -3.764118000 |
| 6 | 6.652113000 | 0.215063000 | -3.427098000 |
| 6 | 8.058416000 | 2.213234000 | -3.025355000 |
| 16 | -1.705622000 | 0.197294000 | -4.184579000 |
| 6 | -3.423452000 | 6.389214000 | -0.998111000 |
| 1 | -1.428110000 | 5.937282000 | -1.685178000 |
| 6 | 0.271207000 | 5.563355000 | 0.682067000 |
| 6 | 1.991501000 | 5.532759000 | -1.079932000 |
| 6 | 2.047096000 | 3.768897000 | -2.811892000 |
| 1 | -5.452754000 | 6.579909000 | -0.204977000 |
| 8 | 2.877872000 | -1.165718000 | -3.724816000 |
| 7 | 1.819363000 | -2.649987000 | -1.741083000 |
| 6 | 3.854446000 | -2.044218000 | -4.168590000 |
| 6 | 6.155577000 | -2.849474000 | -4.171578000 |
| 6 | 7.349640000 | 0.036993000 | -4.657790000 |
| 1 | 8.316361000 | 3.070938000 | -2.384775000 |
| 6 | 8.734019000 | 1.994577000 | -4.216027000 |
| 8 | -0.748215000 | 0.549545000 | -5.259996000 |
| 8 | -2.701989000 | -0.861190000 | -4.463411000 |
| 1 | -3.413606000 | 7.403561000 | -1.423983000 |
| 1 | -0.630625000 | 5.134932000 | 1.139295000 |
| 6 | 0.922865000 | 6.620980000 | 1.301241000 |
| 6 | 2.621396000 | 6.638799000 | -0.434032000 |
| 6 | 2.571888000 | 4.919274000 | -2.221014000 |
| 6 | 2.772499000 | 3.092761000 | -3.916832000 |
| 16 | 2.370230000 | -3.600691000 | -0.569846000 |
| 6 | 3.452116000 | -3.046359000 | -5.112948000 |
| 6 | 5.774307000 | -3.859191000 | -5.127530000 |
| 6 | 7.479499000 | -2.885759000 | -3.639238000 |
| 6 | 8.363177000 | 0.903279000 | -5.044010000 |
| 1 | 7.069256000 | -0.793548000 | -5.317769000 |
| 1 | 9.543256000 | 2.672078000 | -4.527461000 |
| 1 | 0.525751000 | 7.028470000 | 2.243161000 |
| 6 | 2.100442000 | 7.175537000 | 0.732935000 |
| 1 | 3.547306000 | 7.043184000 | -0.872087000 |
| 1 | 3.489234000 | 5.361144000 | -2.637136000 |
| 6 | 2.123437000 | 2.398032000 | -4.959167000 |
| 6 | 4.186493000 | 3.163856000 | -3.955928000 |
| 8 | 2.576403000 | -2.942941000 | 0.736851000 |

| | | | |
|---|---|---|---|
| 8 | 1.527591000 | -4.838179000 | -0.564324000 |
| 6 | 4.435755000 | -3.896002000 | -5.608191000 |
| 6 | 2.051886000 | -3.084356000 | -5.618390000 |
| 6 | 6.743480000 | -4.817783000 | -5.549658000 |
| 6 | 8.394352000 | -3.838647000 | -4.061610000 |
| 1 | 7.763383000 | -2.153689000 | -2.870795000 |
| 1 | 8.875397000 | 0.745421000 | -6.005041000 |
| 1 | 2.604507000 | 8.019161000 | 1.228068000 |
| 6 | 2.838515000 | 1.796104000 | -6.012260000 |
| 1 | 1.029873000 | 2.319161000 | -4.974251000 |
| 6 | 4.900607000 | 2.596484000 | -5.010318000 |
| 1 | 4.736224000 | 3.653368000 | -3.139161000 |
| 1 | 4.161552000 | -4.647819000 | -6.363005000 |
| 6 | 1.803358000 | -2.902018000 | -6.995751000 |
| 6 | 0.950693000 | -3.225227000 | -4.746708000 |
| 1 | 6.438567000 | -5.583669000 | -6.279493000 |
| 6 | 8.029661000 | -4.806564000 | -5.035005000 |
| 1 | 9.403143000 | -3.856694000 | -3.623412000 |
| 6 | 4.248158000 | 1.910212000 | -6.054080000 |
| 1 | 5.998347000 | 2.676377000 | -5.025521000 |
| 6 | 0.493482000 | -2.790557000 | -7.488067000 |
| 1 | 2.652332000 | -2.795341000 | -7.688697000 |
| 6 | -0.359059000 | -3.116844000 | -5.233130000 |
| 1 | 1.126648000 | -3.381801000 | -3.674106000 |
| 1 | 8.762828000 | -5.559662000 | -5.360121000 |
| 6 | -0.602701000 | -2.879641000 | -6.596340000 |
| 1 | -1.205681000 | -3.153309000 | -4.530390000 |
| 6 | -3.240176000 | 4.068262000 | -4.140468000 |
| 6 | -4.402521000 | 3.918428000 | -3.370752000 |
| 6 | -2.408393000 | 2.961428000 | -4.391418000 |
| 6 | -4.693461000 | 2.675583000 | -2.788368000 |
| 6 | -2.705919000 | 1.699501000 | -3.843280000 |
| 6 | -3.850323000 | 1.582578000 | -3.031053000 |
| 6 | 4.081836000 | -5.176523000 | -2.131233000 |
| 6 | 4.010684000 | -4.216388000 | -1.104028000 |
| 6 | 5.293018000 | -5.797456000 | -2.465844000 |
| 6 | 5.205892000 | -3.831592000 | -0.466402000 |
| 6 | 6.470508000 | -5.430319000 | -1.796723000 |
| 6 | 6.431654000 | -4.423202000 | -0.823360000 |
| 9 | 5.248789000 | -2.903880000 | 0.486093000 |
| 9 | 7.562346000 | -4.033214000 | -0.230323000 |
| 9 | 7.624540000 | -6.021094000 | -2.102488000 |
| 9 | 5.341706000 | -6.734442000 | -3.420067000 |
| 9 | 2.989351000 | -5.531194000 | -2.826807000 |
| 9 | -5.208075000 | 4.960828000 | -3.168788000 |
| 9 | -4.169475000 | 0.428330000 | -2.433088000 |
| 9 | -1.334657000 | 3.182790000 | -5.149095000 |

| | | | |
|---|---|---|---|
| 9 | -5.767832000 | 2.535550000 | -2.002796000 |
| 9 | -2.934231000 | 5.266675000 | -4.647051000 |
| 6 | -3.185928000 | -4.043881000 | 1.978259000 |
| 6 | -2.308540000 | -4.314888000 | 3.220997000 |
| 1 | -2.809080000 | -3.865113000 | 4.102951000 |
| 1 | -2.276916000 | -5.408099000 | 3.409051000 |
| 6 | 5.056184000 | 1.298371000 | -7.179279000 |
| 6 | 4.216983000 | 1.019074000 | -8.428110000 |
| 1 | 4.815800000 | 0.455852000 | -9.174100000 |
| 1 | 3.925947000 | 1.978821000 | -8.910812000 |
| 6 | -1.971218000 | -2.627093000 | -7.169451000 |
| 6 | -1.952054000 | -1.327826000 | -8.005101000 |
| 1 | -2.929051000 | -1.205482000 | -8.515614000 |
| 1 | -1.852531000 | -0.489044000 | -7.289251000 |
| 6 | 1.335954000 | 2.533075000 | 3.033090000 |
| 6 | 1.141557000 | 1.575513000 | 4.213119000 |
| 1 | 2.005157000 | 1.662082000 | 4.910157000 |
| 1 | 0.238328000 | 1.862340000 | 4.790632000 |
| 6 | 1.035872000 | 0.131193000 | 3.713842000 |
| 1 | 0.172635000 | 0.051677000 | 3.018257000 |
| 1 | 0.822920000 | -0.565144000 | 4.551831000 |
| 6 | 2.321641000 | -0.292429000 | 2.996755000 |
| 1 | 2.195668000 | -1.266013000 | 2.480712000 |
| 1 | 3.120325000 | -0.461993000 | 3.755671000 |
| 1 | 1.545856000 | 3.566528000 | 3.380848000 |
| 1 | 0.380900000 | 2.603820000 | 2.460979000 |
| 6 | -0.631604000 | -3.014619000 | 1.747434000 |
| 1 | -0.546773000 | -3.773846000 | 0.937668000 |
| 1 | 0.339907000 | -2.486505000 | 1.749516000 |
| 6 | -0.869963000 | -3.739364000 | 3.087301000 |
| 1 | -0.112487000 | -4.541503000 | 3.199461000 |
| 1 | -0.680819000 | -3.018886000 | 3.909673000 |
| 1 | -4.242813000 | -4.319236000 | 2.171064000 |
| 1 | -2.842804000 | -4.696558000 | 1.143581000 |
| 6 | 0.162785000 | -2.496386000 | -8.927457000 |
| 1 | -0.341794000 | -3.384836000 | -9.371594000 |
| 1 | 1.084250000 | -2.328972000 | -9.521263000 |
| 6 | -0.787513000 | -1.279630000 | -9.032165000 |
| 1 | -0.190790000 | -0.359513000 | -8.866383000 |
| 1 | -1.176143000 | -1.217834000 | -10.069648000 |
| 1 | -2.717478000 | -2.540311000 | -6.356597000 |
| 1 | -2.270148000 | -3.484012000 | -7.817482000 |
| 1 | 5.925985000 | 1.949837000 | -7.408014000 |
| 1 | 5.494825000 | 0.339558000 | -6.818533000 |
| 6 | 2.068867000 | 1.063955000 | -7.095934000 |
| 1 | 1.501113000 | 1.815561000 | -7.690809000 |
| 1 | 1.295813000 | 0.425506000 | -6.620189000 |

| | | | |
|---|---|---|---|
| 6 | 2.962287000 | 0.240785000 | -8.027578000 |
| 1 | 3.278094000 | -0.686099000 | -7.502639000 |
| 1 | 2.384347000 | -0.085545000 | -8.917353000 |
| 6 | -4.341992000 | -4.593877000 | -1.631672000 |
| 6 | -4.255269000 | -3.216177000 | -1.796066000 |
| 6 | -2.996296000 | -2.556941000 | -1.876242000 |
| 6 | -3.133323000 | -5.336938000 | -1.562681000 |
| 6 | -1.872084000 | -4.650182000 | -1.677082000 |
| 6 | -1.791481000 | -3.254234000 | -1.813215000 |
| 6 | -2.824876000 | -6.714061000 | -1.409999000 |
| 6 | -1.405939000 | -6.845903000 | -1.434421000 |
| 7 | -0.863514000 | -5.585598000 | -1.636846000 |
| 6 | -0.608371000 | -8.031570000 | -1.400306000 |
| 6 | -1.138589000 | -9.213907000 | -0.630916000 |
| 1 | -2.239166000 | -9.315034000 | -0.666872000 |
| 1 | -0.872187000 | -9.066046000 | 0.438573000 |
| 1 | -0.672021000 | -10.164641000 | -0.954167000 |
| 6 | 0.889407000 | -7.890551000 | -1.399757000 |
| 1 | 1.383750000 | -8.877413000 | -1.479787000 |
| 1 | 1.211197000 | -7.416293000 | -0.446566000 |
| 1 | 1.267839000 | -7.241075000 | -2.209692000 |
| 1 | 0.127643000 | -5.319230000 | -1.438907000 |
| 6 | -2.455942000 | -9.193344000 | -3.533426000 |
| 6 | -3.296508000 | -8.151061000 | -4.305419000 |
| 6 | -0.968632000 | -8.910416000 | -3.492317000 |
| 1 | -2.598023000 | -10.194258000 | -3.991466000 |
| 1 | -2.850603000 | -9.301394000 | -2.502308000 |
| 6 | -0.441490000 | -7.835051000 | -4.220499000 |
| 6 | -2.636146000 | -6.808929000 | -4.419072000 |
| 1 | -4.303517000 | -8.051240000 | -3.855093000 |
| 1 | -3.478266000 | -8.505366000 | -5.347135000 |
| 6 | -1.280530000 | -6.690117000 | -4.509582000 |
| 1 | -0.288229000 | -9.757417000 | -3.313618000 |
| 8 | 0.866111000 | -7.785239000 | -4.482000000 |
| 1 | -3.268220000 | -5.916970000 | -4.556538000 |
| 1 | -0.797063000 | -5.727373000 | -4.735051000 |
| 14 | 1.591699000 | -7.767323000 | -6.075500000 |
| 6 | 1.198656000 | -9.460589000 | -6.798276000 |
| 6 | 0.843357000 | -6.395480000 | -7.104449000 |
| 1 | -0.240706000 | -6.548431000 | -7.276326000 |
| 1 | 1.339564000 | -6.366404000 | -8.097139000 |
| 1 | 0.984824000 | -5.403229000 | -6.632283000 |
| 1 | 1.598451000 | -10.274084000 | -6.159734000 |
| 1 | 1.646076000 | -9.566940000 | -7.808155000 |
| 1 | 0.103429000 | -9.605678000 | -6.900076000 |
| 1 | -5.169138000 | -2.607440000 | -1.855827000 |
| 1 | -2.959894000 | -1.469712000 | -1.993708000 |

| | | | |
|---|---|---|---|
| 1 | -5.315450000 | -5.101807000 | -1.555667000 |
| 1 | -0.828968000 | -2.728265000 | -1.881609000 |
| 1 | -3.541890000 | -7.529893000 | -1.270798000 |
| 6 | 3.405548000 | -7.533293000 | -5.715859000 |
| 1 | 3.807755000 | -8.364152000 | -5.103645000 |
| 1 | 3.986327000 | -7.486047000 | -6.659695000 |
| 1 | 3.569900000 | -6.593442000 | -5.156650000 |

***TS-A4***

***Imaginary frequency = - 238.76***

| | | | |
|---|---|---|---|
| 6 | 2.245355000 | 2.096783000 | 1.861592000 |
| 6 | -4.064399000 | -0.623255000 | 0.476200000 |
| 6 | 2.687176000 | 0.751781000 | 1.834641000 |
| 6 | 2.815595000 | 3.014619000 | 0.957833000 |
| 6 | -2.774297000 | -0.042438000 | 0.401210000 |
| 6 | -4.247244000 | -1.925668000 | 0.953933000 |
| 1 | -4.936565000 | -0.054981000 | 0.122664000 |
| 6 | 3.634338000 | 0.354032000 | 0.873381000 |
| 6 | 3.753812000 | 2.611048000 | 0.006187000 |
| 1 | 2.491703000 | 4.065714000 | 0.987474000 |
| 6 | -2.635934000 | 1.372588000 | -0.035528000 |
| 6 | -1.674143000 | -0.845424000 | 0.787481000 |
| 6 | -3.148143000 | -2.712876000 | 1.333014000 |
| 1 | -5.262132000 | -2.351914000 | 0.999670000 |
| 6 | 4.163942000 | 1.260225000 | -0.067902000 |
| 1 | 3.947544000 | -0.697895000 | 0.857365000 |
| 1 | 4.133173000 | 3.339416000 | -0.725136000 |
| 6 | -1.443484000 | 1.914133000 | -0.626790000 |
| 6 | -3.696928000 | 2.266043000 | 0.134827000 |
| 6 | -1.849211000 | -2.164054000 | 1.230466000 |
| 1 | -0.656236000 | -0.442020000 | 0.738860000 |
| 6 | 5.078706000 | 0.847866000 | -1.161229000 |
| 8 | -0.337044000 | 1.090655000 | -0.774960000 |
| 6 | -1.313598000 | 3.251691000 | -1.021937000 |
| 1 | -4.610218000 | 1.923152000 | 0.641030000 |
| 6 | -3.661700000 | 3.606516000 | -0.328308000 |
| 6 | 4.844582000 | -0.338335000 | -1.926608000 |
| 6 | 6.148523000 | 1.642720000 | -1.563586000 |
| 15 | 0.100155000 | 0.605073000 | -2.292958000 |
| 6 | -2.466802000 | 4.109452000 | -0.954523000 |
| 6 | 0.000810000 | 3.785535000 | -1.477297000 |
| 6 | -4.798366000 | 4.462556000 | -0.212126000 |
| 8 | 3.846454000 | -1.197612000 | -1.494589000 |
| 6 | 5.561198000 | -0.697573000 | -3.070359000 |
| 1 | 6.389048000 | 2.547077000 | -0.984220000 |

| | | | |
|---|---|---|---|
| 6 | 6.924946000 | 1.346115000 | -2.716557000 |
| 8 | 0.136462000 | 2.044882000 | -3.097986000 |
| 7 | 1.556013000 | 0.027785000 | -2.048777000 |
| 7 | -1.039954000 | -0.289371000 | -2.979459000 |
| 6 | -2.487506000 | 5.425660000 | -1.504171000 |
| 6 | 0.594502000 | 4.922935000 | -0.823317000 |
| 6 | 0.696898000 | 3.175333000 | -2.524407000 |
| 1 | -5.704179000 | 4.068086000 | 0.273182000 |
| 6 | -4.774479000 | 5.749345000 | -0.723196000 |
| 15 | 2.399439000 | -1.279586000 | -2.265105000 |
| 6 | 5.190461000 | -1.923903000 | -3.836107000 |
| 6 | 6.613308000 | 0.185503000 | -3.516302000 |
| 6 | 7.976905000 | 2.216449000 | -3.131366000 |
| 16 | -1.729161000 | 0.002081000 | -4.421215000 |
| 6 | -3.615529000 | 6.225125000 | -1.392862000 |
| 1 | -1.600232000 | 5.796655000 | -2.034991000 |
| 6 | 0.062655000 | 5.502230000 | 0.366454000 |
| 6 | 1.813931000 | 5.467253000 | -1.364615000 |
| 6 | 1.932878000 | 3.668726000 | -3.057310000 |
| 1 | -5.662022000 | 6.394140000 | -0.639968000 |
| 8 | 2.869155000 | -1.268425000 | -3.851819000 |
| 7 | 1.801588000 | -2.729816000 | -1.855944000 |
| 6 | 3.868765000 | -2.138341000 | -4.259805000 |
| 6 | 6.183481000 | -2.901937000 | -4.206193000 |
| 6 | 7.336784000 | -0.005639000 | -4.729928000 |
| 1 | 8.207752000 | 3.091767000 | -2.504423000 |
| 6 | 8.678296000 | 1.985177000 | -4.304590000 |
| 1 | -3.616220000 | 7.230868000 | -1.838780000 |
| 1 | -0.839082000 | 5.066628000 | 0.817020000 |
| 6 | 0.683687000 | 6.584659000 | 0.974154000 |
| 6 | 2.411851000 | 6.598217000 | -0.731625000 |
| 6 | 2.425718000 | 4.840942000 | -2.482055000 |
| 6 | 2.691130000 | 2.982689000 | -4.133745000 |
| 16 | 2.348134000 | -3.645288000 | -0.654935000 |
| 6 | 3.500878000 | -3.166733000 | -5.189917000 |
| 6 | 5.837045000 | -3.937439000 | -5.147697000 |
| 6 | 7.498271000 | -2.903864000 | -3.650438000 |
| 6 | 8.342087000 | 0.870359000 | -5.115817000 |
| 1 | 7.083506000 | -0.854691000 | -5.377162000 |
| 1 | 9.481230000 | 2.670380000 | -4.615484000 |
| 1 | 0.262604000 | 7.004175000 | 1.900251000 |
| 6 | 1.860609000 | 7.149344000 | 0.414525000 |
| 1 | 3.337710000 | 7.010668000 | -1.162195000 |
| 1 | 3.342103000 | 5.291072000 | -2.891367000 |
| 6 | 2.074925000 | 2.252661000 | -5.171894000 |
| 6 | 4.104042000 | 3.080011000 | -4.148487000 |
| 8 | 2.519612000 | -2.956300000 | 0.640616000 |

| | | | |
|---|---|---|---|
| 8 | 1.527341000 | -4.897227000 | -0.637290000 |
| 6 | 4.508120000 | -4.008103000 | -5.650663000 |
| 6 | 2.110668000 | -3.241080000 | -5.718504000 |
| 6 | 6.830139000 | -4.886907000 | -5.533283000 |
| 6 | 8.437006000 | -3.848567000 | -4.037348000 |
| 1 | 7.755843000 | -2.151477000 | -2.892434000 |
| 1 | 8.874829000 | 0.701580000 | -6.063745000 |
| 1 | 2.340185000 | 8.012500000 | 0.900225000 |
| 6 | 2.820815000 | 1.641410000 | -6.197891000 |
| 1 | 0.983545000 | 2.152189000 | -5.205090000 |
| 6 | 4.848311000 | 2.503422000 | -5.176686000 |
| 1 | 4.628992000 | 3.597764000 | -3.332812000 |
| 1 | 4.260641000 | -4.779837000 | -6.394510000 |
| 6 | 1.883320000 | -3.093434000 | -7.103703000 |
| 6 | 0.996839000 | -3.382791000 | -4.863093000 |
| 1 | 6.551536000 | -5.672590000 | -6.252506000 |
| 6 | 8.106639000 | -4.842335000 | -4.996838000 |
| 1 | 9.438068000 | -3.839638000 | -3.581497000 |
| 6 | 4.228591000 | 1.781823000 | -6.216530000 |
| 1 | 5.944486000 | 2.603774000 | -5.173498000 |
| 6 | 0.580612000 | -3.016272000 | -7.620890000 |
| 1 | 2.742286000 | -2.986319000 | -7.784139000 |
| 6 | -0.305857000 | -3.308252000 | -5.374356000 |
| 1 | 1.156448000 | -3.513491000 | -3.784453000 |
| 1 | 8.858550000 | -5.588574000 | -5.293732000 |
| 6 | -0.529487000 | -3.105356000 | -6.746507000 |
| 1 | -1.163970000 | -3.344144000 | -4.685617000 |
| 6 | -4.784564000 | 2.454735000 | -3.130268000 |
| 6 | -4.504706000 | 3.690461000 | -3.732884000 |
| 6 | -3.918524000 | 1.372023000 | -3.335676000 |
| 6 | -3.331299000 | 3.845234000 | -4.484647000 |
| 6 | -2.761725000 | 1.492775000 | -4.129571000 |
| 6 | -2.476141000 | 2.748411000 | -4.698123000 |
| 6 | 4.113910000 | -5.223948000 | -2.151592000 |
| 6 | 4.008179000 | -4.242945000 | -1.147316000 |
| 6 | 5.341635000 | -5.829279000 | -2.452840000 |
| 6 | 5.185352000 | -3.822554000 | -0.498721000 |
| 6 | 6.500887000 | -5.426057000 | -1.772741000 |
| 6 | 6.427465000 | -4.398919000 | -0.822583000 |
| 9 | 5.195484000 | -2.873925000 | 0.433876000 |
| 9 | 7.540789000 | -3.975310000 | -0.219826000 |
| 9 | 7.670367000 | -6.001945000 | -2.046506000 |
| 9 | 5.423250000 | -6.785548000 | -3.385432000 |
| 9 | 3.039852000 | -5.614082000 | -2.856794000 |
| 9 | -5.331810000 | 4.722268000 | -3.566944000 |
| 9 | -1.392740000 | 2.973337000 | -5.440813000 |
| 9 | -4.228456000 | 0.224595000 | -2.719998000 |

| | | | |
|---|---|---|---|
| 9 | -3.037041000 | 5.038392000 | -5.010218000 |
| 9 | -5.870488000 | 2.311566000 | -2.361332000 |
| 6 | -3.243138000 | -4.135969000 | 1.812210000 |
| 6 | -2.382693000 | -4.361855000 | 3.075659000 |
| 1 | -2.906248000 | -3.900436000 | 3.938020000 |
| 1 | -2.335177000 | -5.449571000 | 3.290337000 |
| 6 | 5.069174000 | 1.160645000 | -7.312359000 |
| 6 | 4.259062000 | 0.835284000 | -8.569156000 |
| 1 | 4.882503000 | 0.266419000 | -9.290275000 |
| 1 | 3.958442000 | 1.777264000 | -9.080148000 |
| 6 | -1.891716000 | -2.889500000 | -7.348920000 |
| 6 | -1.879678000 | -1.607998000 | -8.211672000 |
| 1 | -2.849724000 | -1.512621000 | -8.740841000 |
| 1 | -1.805857000 | -0.752885000 | -7.512239000 |
| 6 | 1.139381000 | 2.541780000 | 2.796249000 |
| 6 | 0.939850000 | 1.606053000 | 3.992839000 |
| 1 | 1.787508000 | 1.724400000 | 4.704626000 |
| 1 | 0.020008000 | 1.887634000 | 4.546210000 |
| 6 | 0.871706000 | 0.149463000 | 3.523555000 |
| 1 | 0.024262000 | 0.037781000 | 2.813149000 |
| 1 | 0.655653000 | -0.532571000 | 4.372444000 |
| 6 | 2.179291000 | -0.264061000 | 2.841024000 |
| 1 | 2.083145000 | -1.251723000 | 2.345785000 |
| 1 | 2.966416000 | -0.399244000 | 3.618714000 |
| 1 | 1.324124000 | 3.586009000 | 3.125625000 |
| 1 | 0.194162000 | 2.583078000 | 2.205239000 |
| 6 | -0.703964000 | -3.066144000 | 1.600338000 |
| 1 | -0.592661000 | -3.842233000 | 0.810026000 |
| 1 | 0.258019000 | -2.520935000 | 1.605737000 |
| 6 | -0.952326000 | -3.763929000 | 2.952633000 |
| 1 | -0.182959000 | -4.549591000 | 3.096161000 |
| 1 | -0.789900000 | -3.021406000 | 3.760992000 |
| 1 | -4.298183000 | -4.425602000 | 1.993884000 |
| 1 | -2.874510000 | -4.801853000 | 0.999100000 |
| 6 | 0.270213000 | -2.759611000 | -9.071931000 |
| 1 | -0.211280000 | -3.666333000 | -9.504627000 |
| 1 | 1.198909000 | -2.589293000 | -9.653514000 |
| 6 | -0.698763000 | -1.562202000 | -9.219955000 |
| 1 | -0.120757000 | -0.628369000 | -9.064860000 |
| 1 | -1.070540000 | -1.530263000 | -10.264936000 |
| 1 | -2.654104000 | -2.798931000 | -6.551605000 |
| 1 | -2.163924000 | -3.764988000 | -7.983759000 |
| 1 | 5.930051000 | 1.824031000 | -7.540583000 |
| 1 | 5.519893000 | 0.219534000 | -6.921212000 |
| 6 | 2.085853000 | 0.870131000 | -7.278537000 |
| 1 | 1.516662000 | 1.596965000 | -7.902154000 |
| 1 | 1.315033000 | 0.228795000 | -6.803069000 |

| | | | |
|---|---|---|---|
| 6 | 3.012428000 | 0.042118000 | -8.172610000 |
| 1 | 3.336165000 | -0.864893000 | -7.618518000 |
| 1 | 2.457937000 | -0.317511000 | -9.064458000 |
| 6 | -4.325759000 | -4.778202000 | -1.808486000 |
| 6 | -4.259552000 | -3.403190000 | -2.002279000 |
| 6 | -3.010753000 | -2.724204000 | -2.076042000 |
| 6 | -3.106066000 | -5.498519000 | -1.702420000 |
| 6 | -1.854857000 | -4.792742000 | -1.810944000 |
| 6 | -1.795539000 | -3.398998000 | -1.977235000 |
| 6 | -2.776983000 | -6.866382000 | -1.513511000 |
| 6 | -1.355787000 | -6.974051000 | -1.510705000 |
| 7 | -0.831425000 | -5.709400000 | -1.732326000 |
| 6 | -0.538994000 | -8.144796000 | -1.436871000 |
| 6 | -1.062802000 | -9.319381000 | -0.651290000 |
| 1 | -2.160825000 | -9.439613000 | -0.702962000 |
| 1 | -0.816627000 | -9.144772000 | 0.419010000 |
| 1 | -0.575408000 | -10.268736000 | -0.946662000 |
| 6 | 0.956057000 | -7.978394000 | -1.412687000 |
| 1 | 1.467871000 | -8.958454000 | -1.459845000 |
| 1 | 1.252439000 | -7.476233000 | -0.465575000 |
| 1 | 1.338975000 | -7.342275000 | -2.231090000 |
| 1 | 0.151504000 | -5.421447000 | -1.523684000 |
| 6 | -2.328132000 | -9.383273000 | -3.574818000 |
| 6 | -3.172455000 | -8.374678000 | -4.386198000 |
| 6 | -0.846821000 | -9.073228000 | -3.514775000 |
| 1 | -2.445087000 | -10.397224000 | -4.010715000 |
| 1 | -2.739233000 | -9.473049000 | -2.548393000 |
| 6 | -0.326232000 | -8.004121000 | -4.256885000 |
| 6 | -2.534949000 | -7.023055000 | -4.516678000 |
| 1 | -4.189958000 | -8.284478000 | -3.957977000 |
| 1 | -3.326563000 | -8.755190000 | -5.423128000 |
| 6 | -1.180275000 | -6.881064000 | -4.585001000 |
| 1 | -0.154885000 | -9.904083000 | -3.306862000 |
| 8 | 0.984528000 | -7.936041000 | -4.497340000 |
| 1 | -3.180486000 | -6.146092000 | -4.684614000 |
| 1 | -0.709983000 | -5.914690000 | -4.822477000 |
| 14 | 1.736847000 | -7.939488000 | -6.078591000 |
| 6 | 1.390813000 | -9.656817000 | -6.767728000 |
| 6 | 0.977951000 | -6.607657000 | -7.151644000 |
| 1 | -0.099747000 | -6.787008000 | -7.337513000 |
| 1 | 1.490105000 | -6.591596000 | -8.136508000 |
| 1 | 1.091252000 | -5.601822000 | -6.701081000 |
| 1 | 1.797082000 | -10.446732000 | -6.104164000 |
| 1 | 1.856312000 | -9.777038000 | -7.767824000 |
| 1 | 0.300547000 | -9.827113000 | -6.882862000 |
| 1 | -5.182417000 | -2.811805000 | -2.091006000 |
| 1 | -2.990683000 | -1.639333000 | -2.216907000 |

| | | | |
|---|---|---|---|
| 1 | -5.291673000 | -5.301052000 | -1.737524000 |
| 1 | -0.840964000 | -2.858168000 | -2.041264000 |
| 1 | -3.482280000 | -7.691371000 | -1.368448000 |
| 6 | 3.538833000 | -7.658558000 | -5.694255000 |
| 1 | 3.947710000 | -8.465769000 | -5.055450000 |
| 1 | 4.134819000 | -7.621222000 | -6.629007000 |
| 1 | 3.673509000 | -6.702322000 | -5.155185000 |
| 8 | -0.757994000 | 0.349118000 | -5.485946000 |
| 8 | -2.701337000 | -1.079666000 | -4.696028000 |

***TS-A5***

***Imaginary frequency = -235.94***

| | | | |
|---|---|---|---|
| 6 | 2.413867000 | 1.688004000 | 2.326057000 |
| 6 | -0.674704000 | -0.427940000 | 1.338758000 |
| 6 | 2.950525000 | 2.689038000 | 1.481611000 |
| 6 | 2.826627000 | 0.353487000 | 2.136302000 |
| 6 | -1.963006000 | 0.005475000 | 0.939623000 |
| 6 | -0.479602000 | -1.702835000 | 1.878554000 |
| 1 | 0.197319000 | 0.221340000 | 1.208553000 |
| 6 | 3.835457000 | 2.314554000 | 0.454120000 |
| 6 | 3.716488000 | -0.008521000 | 1.123718000 |
| 1 | 2.423055000 | -0.430743000 | 2.796574000 |
| 6 | -2.189922000 | 1.344889000 | 0.332685000 |
| 6 | -3.044222000 | -0.883756000 | 1.150076000 |
| 6 | -1.558215000 | -2.576866000 | 2.079508000 |
| 1 | 0.542346000 | -2.035369000 | 2.113398000 |
| 6 | 4.228074000 | 0.977592000 | 0.248113000 |
| 1 | 4.195996000 | 3.092797000 | -0.238058000 |
| 1 | 3.994810000 | -1.061645000 | 1.005812000 |
| 6 | -1.168559000 | 2.018522000 | -0.418580000 |
| 6 | -3.399771000 | 2.026229000 | 0.469946000 |
| 6 | -2.860730000 | -2.151578000 | 1.724342000 |
| 1 | -4.057023000 | -0.595507000 | 0.827138000 |
| 6 | 5.129292000 | 0.649461000 | -0.884097000 |
| 8 | 0.021339000 | 1.343412000 | -0.637726000 |
| 6 | -1.289662000 | 3.318230000 | -0.926551000 |
| 1 | -4.208650000 | 1.573829000 | 1.062590000 |
| 6 | -3.633081000 | 3.299261000 | -0.119680000 |
| 6 | 4.895112000 | -0.487939000 | -1.718955000 |
| 6 | 6.204097000 | 1.462299000 | -1.231688000 |
| 15 | 0.290421000 | 0.777438000 | -2.163362000 |
| 6 | -2.572989000 | 3.963187000 | -0.838070000 |
| 6 | -0.089516000 | 4.006931000 | -1.487351000 |
| 6 | -4.901664000 | 3.944656000 | -0.010890000 |
| 8 | 3.849211000 | -1.331032000 | -1.375850000 |
| 6 | 5.641709000 | -0.808958000 | -2.854376000 |

| | | | |
|---|---|---|---|
| 1 | 6.440259000 | 2.334486000 | -0.603407000 |
| 6 | 7.004340000 | 1.212425000 | -2.379535000 |
| 8 | 0.264413000 | 2.193539000 | -3.005967000 |
| 7 | 1.747550000 | 0.160882000 | -2.056735000 |
| 7 | -0.945748000 | -0.095170000 | -2.707092000 |
| 6 | -2.848968000 | 5.218572000 | -1.456349000 |
| 6 | 0.355834000 | 5.270218000 | -0.948222000 |
| 6 | 0.690631000 | 3.396253000 | -2.475768000 |
| 1 | -5.704921000 | 3.430466000 | 0.538904000 |
| 6 | -5.129900000 | 5.177705000 | -0.599952000 |
| 15 | 2.433313000 | -1.250209000 | -2.206812000 |
| 6 | 5.272618000 | -1.996389000 | -3.681610000 |
| 6 | 6.718553000 | 0.078814000 | -3.227326000 |
| 6 | 8.070465000 | 2.093858000 | -2.729095000 |
| 16 | -1.611872000 | 0.108317000 | -4.174736000 |
| 6 | -4.095722000 | 5.814639000 | -1.335832000 |
| 1 | -2.060778000 | 5.705830000 | -2.046773000 |
| 6 | -0.279286000 | 5.918677000 | 0.153698000 |
| 6 | 1.536907000 | 5.883571000 | -1.506903000 |
| 6 | 1.900318000 | 3.955375000 | -3.002345000 |
| 1 | -6.117101000 | 5.656027000 | -0.516596000 |
| 8 | 2.976502000 | -1.272801000 | -3.770956000 |
| 7 | 1.657588000 | -2.617310000 | -1.825555000 |
| 6 | 3.962352000 | -2.162691000 | -4.159247000 |
| 6 | 6.254642000 | -2.984666000 | -4.052525000 |
| 6 | 7.493471000 | -0.081682000 | -4.413217000 |
| 1 | 8.279332000 | 2.948145000 | -2.066484000 |
| 6 | 8.815110000 | 1.896981000 | -3.881624000 |
| 8 | -0.619520000 | 0.399183000 | -5.238673000 |
| 8 | -2.577979000 | -0.989180000 | -4.410209000 |
| 1 | -4.290287000 | 6.776148000 | -1.833166000 |
| 1 | -1.142363000 | 5.439788000 | 0.633199000 |
| 6 | 0.190792000 | 7.129038000 | 0.643803000 |
| 6 | 1.982783000 | 7.137060000 | -0.990702000 |
| 6 | 2.270244000 | 5.208945000 | -2.519351000 |
| 6 | 2.749975000 | 3.229846000 | -3.979580000 |
| 16 | 2.074396000 | -3.658310000 | -0.670520000 |
| 6 | 3.603227000 | -3.147630000 | -5.141433000 |
| 6 | 5.920096000 | -3.970299000 | -5.050216000 |
| 6 | 7.544290000 | -3.046068000 | -3.444403000 |
| 6 | 8.513177000 | 0.803933000 | -4.734406000 |
| 1 | 7.271396000 | -0.917059000 | -5.089164000 |
| 1 | 9.629223000 | 2.590075000 | -4.141845000 |
| 1 | -0.315753000 | 7.601321000 | 1.499046000 |
| 6 | 1.323151000 | 7.754765000 | 0.059744000 |
| 1 | 2.878956000 | 7.595925000 | -1.436760000 |
| 1 | 3.168926000 | 5.694880000 | -2.928146000 |

| | | | |
|---|---|---|---|
| 6 | 2.212323000 | 2.452911000 | -5.025823000 |
| 6 | 4.158945000 | 3.320895000 | -3.878537000 |
| 8 | 2.267691000 | -3.071610000 | 0.670073000 |
| 8 | 1.141906000 | -4.824101000 | -0.755266000 |
| 6 | 4.608995000 | -3.990553000 | -5.602200000 |
| 6 | 2.227657000 | -3.156386000 | -5.712537000 |
| 6 | 6.903800000 | -4.928486000 | -5.437409000 |
| 6 | 8.473225000 | -3.999716000 | -3.833564000 |
| 1 | 7.789062000 | -2.332921000 | -2.645187000 |
| 1 | 9.086070000 | 0.658283000 | -5.662562000 |
| 1 | 1.683759000 | 8.717411000 | 0.452350000 |
| 6 | 3.032034000 | 1.791081000 | -5.957679000 |
| 1 | 1.125537000 | 2.352332000 | -5.134315000 |
| 6 | 4.979452000 | 2.687696000 | -4.813607000 |
| 1 | 4.613320000 | 3.877222000 | -3.044661000 |
| 1 | 4.374970000 | -4.726180000 | -6.385372000 |
| 6 | 2.038964000 | -2.959522000 | -7.097698000 |
| 6 | 1.088175000 | -3.241927000 | -4.884185000 |
| 1 | 6.634942000 | -5.675116000 | -6.200595000 |
| 6 | 8.158657000 | -4.939886000 | -4.850516000 |
| 1 | 9.454613000 | -4.037992000 | -3.337921000 |
| 6 | 4.437901000 | 1.926689000 | -5.869679000 |
| 1 | 6.073647000 | 2.773553000 | -4.722698000 |
| 6 | 0.755646000 | -2.763045000 | -7.633594000 |
| 1 | 2.915965000 | -2.895417000 | -7.760288000 |
| 6 | -0.193622000 | -3.044615000 | -5.411967000 |
| 1 | 1.207972000 | -3.409578000 | -3.806574000 |
| 1 | 8.903240000 | -5.692423000 | -5.150062000 |
| 6 | -0.373874000 | -2.778669000 | -6.779560000 |
| 1 | -1.063520000 | -3.020937000 | -4.737250000 |
| 6 | -3.255548000 | 3.912988000 | -4.572617000 |
| 6 | -4.477066000 | 3.783307000 | -3.897619000 |
| 6 | -2.366084000 | 2.825029000 | -4.643911000 |
| 6 | -4.777893000 | 2.586885000 | -3.230480000 |
| 6 | -2.665441000 | 1.605938000 | -4.006484000 |
| 6 | -3.871419000 | 1.518617000 | -3.283637000 |
| 6 | 3.742450000 | -5.277185000 | -2.245499000 |
| 6 | 3.690923000 | -4.370659000 | -1.169547000 |
| 6 | 4.932386000 | -5.931345000 | -2.593280000 |
| 6 | 4.884646000 | -4.094328000 | -0.475838000 |
| 6 | 6.106696000 | -5.669947000 | -1.871033000 |
| 6 | 6.089590000 | -4.724353000 | -0.837314000 |
| 9 | 4.948176000 | -3.230787000 | 0.534973000 |
| 9 | 7.219496000 | -4.433891000 | -0.188246000 |
| 9 | 7.237788000 | -6.299447000 | -2.183783000 |
| 9 | 4.962937000 | -6.803337000 | -3.608127000 |
| 9 | 2.652910000 | -5.541127000 | -2.985079000 |

| | | | |
|---|---|---|---|
| 9 | -5.335206000 | 4.803787000 | -3.866148000 |
| 9 | -4.204644000 | 0.414933000 | -2.604456000 |
| 9 | -1.243507000 | 3.022332000 | -5.335032000 |
| 9 | -5.926431000 | 2.464626000 | -2.557019000 |
| 9 | -2.945652000 | 5.072942000 | -5.160851000 |
| 6 | -1.386347000 | -3.981133000 | 2.598883000 |
| 6 | -2.205724000 | -4.951626000 | 1.731163000 |
| 1 | -1.772215000 | -4.921451000 | 0.711061000 |
| 1 | -2.081196000 | -5.993138000 | 2.094439000 |
| 6 | 5.353624000 | 1.260238000 | -6.874500000 |
| 6 | 4.637372000 | 0.886455000 | -8.173719000 |
| 1 | 5.311981000 | 0.292235000 | -8.824928000 |
| 1 | 4.373476000 | 1.806807000 | -8.741394000 |
| 6 | -1.702584000 | -2.423169000 | -7.390536000 |
| 6 | -1.561269000 | -1.130775000 | -8.225050000 |
| 1 | -2.512239000 | -0.932809000 | -8.760523000 |
| 1 | -1.415983000 | -0.302157000 | -7.504259000 |
| 6 | 1.408837000 | 2.029330000 | 3.408011000 |
| 6 | 0.728057000 | 3.381956000 | 3.180080000 |
| 1 | 0.041049000 | 3.310806000 | 2.306607000 |
| 1 | 0.100967000 | 3.647130000 | 4.056219000 |
| 6 | 1.782816000 | 4.458086000 | 2.909055000 |
| 1 | 2.486909000 | 4.500008000 | 3.770260000 |
| 1 | 1.318644000 | 5.462014000 | 2.826812000 |
| 6 | 2.544747000 | 4.142172000 | 1.619348000 |
| 1 | 3.440303000 | 4.791188000 | 1.519703000 |
| 1 | 1.901346000 | 4.399717000 | 0.752824000 |
| 1 | 0.658245000 | 1.214217000 | 3.481225000 |
| 1 | 1.931277000 | 2.045341000 | 4.392657000 |
| 6 | -4.001076000 | -3.095185000 | 2.018902000 |
| 1 | -4.211277000 | -3.023085000 | 3.110326000 |
| 1 | -4.926199000 | -2.756873000 | 1.509371000 |
| 6 | -3.705454000 | -4.580212000 | 1.677231000 |
| 1 | -4.282136000 | -5.219427000 | 2.377790000 |
| 1 | -4.091450000 | -4.802714000 | 0.664101000 |
| 1 | -0.312860000 | -4.257462000 | 2.591497000 |
| 1 | -1.731275000 | -4.044441000 | 3.657704000 |
| 6 | 0.491881000 | -2.450743000 | -9.083100000 |
| 1 | -0.055818000 | -3.306112000 | -9.541107000 |
| 1 | 1.440707000 | -2.348027000 | -9.647999000 |
| 6 | -0.371540000 | -1.174072000 | -9.223141000 |
| 1 | 0.281672000 | -0.293868000 | -9.053759000 |
| 1 | -0.730316000 | -1.099068000 | -10.270475000 |
| 1 | -2.460090000 | -2.278451000 | -6.595799000 |
| 1 | -2.050652000 | -3.256287000 | -8.045298000 |
| 1 | 6.234202000 | 1.910029000 | -7.062855000 |
| 1 | 5.766871000 | 0.334311000 | -6.415035000 |

| | | | |
|---|---|---|---|
| 6 | 2.376968000 | 0.965545000 | -7.049265000 |
| 1 | 1.848715000 | 1.657788000 | -7.744372000 |
| 1 | 1.580458000 | 0.338950000 | -6.595782000 |
| 6 | 3.367018000 | 0.103661000 | -7.836808000 |
| 1 | 3.650827000 | -0.777129000 | -7.221862000 |
| 1 | 2.879677000 | -0.296287000 | -8.750118000 |
| 6 | -4.580105000 | -4.200733000 | -1.866674000 |
| 6 | -4.362744000 | -2.829291000 | -1.790782000 |
| 6 | -3.045487000 | -2.290496000 | -1.747679000 |
| 6 | -3.446106000 | -5.056946000 | -1.899559000 |
| 6 | -2.125946000 | -4.479266000 | -1.895879000 |
| 6 | -1.911018000 | -3.096361000 | -1.804746000 |
| 6 | -3.263702000 | -6.464033000 | -1.901290000 |
| 6 | -1.860675000 | -6.719285000 | -1.897544000 |
| 7 | -1.204935000 | -5.499030000 | -1.954349000 |
| 6 | -1.163569000 | -7.964502000 | -1.922078000 |
| 6 | -1.814651000 | -9.150671000 | -1.258812000 |
| 1 | -2.916824000 | -9.163386000 | -1.348884000 |
| 1 | -1.590700000 | -9.093164000 | -0.170985000 |
| 1 | -1.405064000 | -10.111609000 | -1.626266000 |
| 6 | 0.339940000 | -7.937683000 | -1.853630000 |
| 1 | 0.765977000 | -8.949069000 | -1.995953000 |
| 1 | 0.652203000 | -7.570402000 | -0.850842000 |
| 1 | 0.799224000 | -7.257108000 | -2.593664000 |
| 1 | -0.207129000 | -5.326177000 | -1.701753000 |
| 6 | -3.017164000 | -8.820764000 | -4.197399000 |
| 6 | -3.735423000 | -7.651695000 | -4.909517000 |
| 6 | -1.514299000 | -8.674553000 | -4.087772000 |
| 1 | -3.227673000 | -9.767253000 | -4.737615000 |
| 1 | -3.457098000 | -8.973528000 | -3.190442000 |
| 6 | -0.867104000 | -7.599058000 | -4.708542000 |
| 6 | -2.955818000 | -6.369703000 | -4.902969000 |
| 1 | -4.745155000 | -7.495621000 | -4.481888000 |
| 1 | -3.910870000 | -7.910131000 | -5.980136000 |
| 6 | -1.592427000 | -6.368889000 | -4.944493000 |
| 1 | -0.916627000 | -9.587402000 | -3.940187000 |
| 8 | 0.453728000 | -7.634715000 | -4.901593000 |
| 1 | -3.500757000 | -5.416534000 | -4.996436000 |
| 1 | -1.016244000 | -5.442569000 | -5.089570000 |
| 14 | 1.273177000 | -7.661646000 | -6.444669000 |
| 6 | 0.963629000 | -9.387802000 | -7.129923000 |
| 6 | 0.546977000 | -6.346628000 | -7.561155000 |
| 1 | -0.533512000 | -6.513741000 | -7.744965000 |
| 1 | 1.062915000 | -6.369307000 | -8.543872000 |
| 1 | 0.676651000 | -5.330585000 | -7.138597000 |
| 1 | 1.348826000 | -10.167802000 | -6.442440000 |
| 1 | 1.468217000 | -9.516297000 | -8.109895000 |

| | | | |
|---|---|---|---|
| 1 | -0.120330000 | -9.566556000 | -7.284772000 |
| 1 | -5.215732000 | -2.135103000 | -1.767887000 |
| 1 | -2.901278000 | -1.208215000 | -1.685372000 |
| 1 | -5.597982000 | -4.618876000 | -1.887277000 |
| 1 | -0.902622000 | -2.660155000 | -1.791504000 |
| 1 | -4.051936000 | -7.223187000 | -1.863728000 |
| 6 | 3.053795000 | -7.366622000 | -5.983672000 |
| 1 | 3.443591000 | -8.173311000 | -5.332777000 |
| 1 | 3.691424000 | -7.312452000 | -6.889441000 |
| 1 | 3.148114000 | -6.413510000 | -5.430025000 |

***TS-A6***

***Imaginary frequency = - 240.10***

| | | | |
|---|---|---|---|
| 6 | 2.370528000 | 1.114134000 | 2.545758000 |
| 6 | -3.651563000 | -0.250417000 | 1.051889000 |
| 6 | 2.622776000 | 2.219232000 | 1.698163000 |
| 6 | 3.101329000 | -0.073794000 | 2.342568000 |
| 6 | -2.380080000 | 0.341729000 | 0.849487000 |
| 6 | -3.777543000 | -1.543280000 | 1.571890000 |
| 1 | -4.559634000 | 0.293486000 | 0.755330000 |
| 6 | 3.564685000 | 2.091060000 | 0.659024000 |
| 6 | 4.033293000 | -0.194122000 | 1.311069000 |
| 1 | 2.914806000 | -0.940644000 | 2.996543000 |
| 6 | -2.296990000 | 1.716958000 | 0.287660000 |
| 6 | -1.241964000 | -0.430619000 | 1.190218000 |
| 6 | -2.640631000 | -2.317424000 | 1.854875000 |
| 1 | -4.779614000 | -1.979649000 | 1.710025000 |
| 6 | 4.267105000 | 0.892012000 | 0.437293000 |
| 1 | 3.704220000 | 2.934365000 | -0.037439000 |
| 1 | 4.552431000 | -1.148350000 | 1.161864000 |
| 6 | -1.143604000 | 2.220511000 | -0.406305000 |
| 6 | -3.375225000 | 2.599312000 | 0.400085000 |
| 6 | -1.362777000 | -1.748752000 | 1.650369000 |
| 1 | -0.234513000 | -0.015847000 | 1.076109000 |
| 6 | 5.145119000 | 0.746153000 | -0.751794000 |
| 8 | -0.020682000 | 1.412305000 | -0.503376000 |
| 6 | -1.063001000 | 3.509947000 | -0.948201000 |
| 1 | -4.261291000 | 2.294970000 | 0.974646000 |
| 6 | -3.394878000 | 3.879150000 | -0.211916000 |
| 6 | 4.954374000 | -0.363859000 | -1.631424000 |
| 6 | 6.120985000 | 1.666597000 | -1.115165000 |
| 15 | 0.350883000 | 0.753405000 | -1.972769000 |
| 6 | -2.239505000 | 4.337606000 | -0.938111000 |
| 6 | 0.231215000 | 4.029785000 | -1.471506000 |

| | | | |
|---|---|---|---|
| 6 | -4.550038000 | 4.716320000 | -0.147084000 |
| 8 | 3.999706000 | -1.300856000 | -1.269206000 |
| 6 | 5.646456000 | -0.571580000 | -2.823968000 |
| 1 | 6.311691000 | 2.533231000 | -0.463637000 |
| 6 | 6.888351000 | 1.517484000 | -2.306022000 |
| 8 | 0.445332000 | 2.120999000 | -2.896834000 |
| 7 | 1.756035000 | 0.077414000 | -1.679712000 |
| 7 | -0.850690000 | -0.128288000 | -2.566458000 |
| 6 | -2.318281000 | 5.581121000 | -1.632799000 |
| 6 | 0.783201000 | 5.255688000 | -0.945106000 |
| 6 | 0.962170000 | 3.317426000 | -2.429805000 |
| 1 | -5.425967000 | 4.358462000 | 0.415315000 |
| 6 | -4.581385000 | 5.937100000 | -0.799914000 |
| 15 | 2.525853000 | -1.263549000 | -1.985861000 |
| 6 | 5.295656000 | -1.736053000 | -3.691119000 |
| 6 | 6.649452000 | 0.400754000 | -3.191842000 |
| 6 | 7.887574000 | 2.475560000 | -2.651619000 |
| 16 | -1.550872000 | 0.083737000 | -4.015871000 |
| 6 | -3.462378000 | 6.362006000 | -1.565536000 |
| 1 | -1.462848000 | 5.908159000 | -2.239505000 |
| 6 | 0.202772000 | 5.976350000 | 0.141636000 |
| 6 | 2.015650000 | 5.748734000 | -1.502635000 |
| 6 | 2.191871000 | 3.786971000 | -2.999995000 |
| 1 | -5.483020000 | 6.565773000 | -0.753647000 |
| 8 | 2.951874000 | -1.172045000 | -3.586436000 |
| 7 | 1.870410000 | -2.696838000 | -1.618056000 |
| 6 | 3.966903000 | -1.971861000 | -4.086917000 |
| 6 | 6.312044000 | -2.640626000 | -4.167744000 |
| 6 | 7.400468000 | 0.331963000 | -4.401578000 |
| 1 | 8.061095000 | 3.318475000 | -1.964556000 |
| 6 | 8.624992000 | 2.356858000 | -3.819841000 |
| 8 | -0.584337000 | 0.349556000 | -5.108617000 |
| 8 | -2.540371000 | -1.000113000 | -4.213336000 |
| 1 | -3.509367000 | 7.308941000 | -2.123400000 |
| 1 | -0.708326000 | 5.591313000 | 0.617273000 |
| 6 | 0.786215000 | 7.137873000 | 0.626292000 |
| 6 | 2.579250000 | 6.959348000 | -0.997092000 |
| 6 | 2.661853000 | 5.014309000 | -2.530168000 |
| 6 | 2.950599000 | 3.053113000 | -4.043968000 |
| 16 | 2.494986000 | -3.767773000 | -0.587860000 |
| 6 | 3.606662000 | -2.943314000 | -5.083868000 |
| 6 | 5.977563000 | -3.598976000 | -5.190444000 |
| 6 | 7.636212000 | -2.652672000 | -3.635689000 |
| 6 | 8.370362000 | 1.277222000 | -4.704560000 |
| 1 | 7.200257000 | -0.481462000 | -5.109844000 |
| 1 | 9.394945000 | 3.102899000 | -4.067861000 |
| 1 | 0.322649000 | 7.664830000 | 1.473889000 |

| | | | |
|---|---|---|---|
| 6 | 1.978164000 | 7.645828000 | 0.045186000 |
| 1 | 3.514939000 | 7.327615000 | -1.446058000 |
| 1 | 3.573420000 | 5.440774000 | -2.973008000 |
| 6 | 2.338982000 | 2.230759000 | -5.013978000 |
| 6 | 4.352481000 | 3.235992000 | -4.127765000 |
| 8 | 2.823243000 | -3.243925000 | 0.751194000 |
| 8 | 1.616509000 | -4.979226000 | -0.629663000 |
| 6 | 4.635882000 | -3.690573000 | -5.650185000 |
| 6 | 2.202106000 | -3.055032000 | -5.566438000 |
| 6 | 6.993235000 | -4.469352000 | -5.687599000 |
| 6 | 8.596490000 | -3.524541000 | -4.127450000 |
| 1 | 7.884112000 | -1.969421000 | -2.811889000 |
| 1 | 8.932285000 | 1.195784000 | -5.647209000 |
| 1 | 2.427656000 | 8.572692000 | 0.432247000 |
| 6 | 3.077704000 | 1.625357000 | -6.049041000 |
| 1 | 1.255866000 | 2.058590000 | -4.992444000 |
| 6 | 5.084299000 | 2.669943000 | -5.168842000 |
| 1 | 4.879957000 | 3.820725000 | -3.360572000 |
| 1 | 4.399639000 | -4.417222000 | -6.440464000 |
| 6 | 1.925811000 | -2.962042000 | -6.948649000 |
| 6 | 1.113983000 | -3.175632000 | -4.674754000 |
| 1 | 6.724126000 | -5.193624000 | -6.471830000 |
| 6 | 8.280681000 | -4.426665000 | -5.177977000 |
| 1 | 9.605310000 | -3.526939000 | -3.688723000 |
| 6 | 4.468083000 | 1.866943000 | -6.149326000 |
| 1 | 6.169335000 | 2.843763000 | -5.220774000 |
| 6 | 0.607576000 | -2.882635000 | -7.422458000 |
| 1 | 2.758473000 | -2.878028000 | -7.663047000 |
| 6 | -0.204001000 | -3.103417000 | -5.141331000 |
| 1 | 1.297662000 | -3.289810000 | -3.599333000 |
| 1 | 9.050892000 | -5.112461000 | -5.561160000 |
| 6 | -0.473518000 | -2.922526000 | -6.507815000 |
| 1 | -1.036775000 | -3.106560000 | -4.421204000 |
| 6 | -3.178785000 | 3.891476000 | -4.402582000 |
| 6 | -4.356607000 | 3.786356000 | -3.649222000 |
| 6 | -2.299123000 | 2.797641000 | -4.499788000 |
| 6 | -4.616463000 | 2.612446000 | -2.925976000 |
| 6 | -2.571192000 | 1.596167000 | -3.818956000 |
| 6 | -3.727582000 | 1.532478000 | -3.017396000 |
| 6 | 4.034264000 | -5.218246000 | -2.431559000 |
| 6 | 4.070513000 | -4.367957000 | -1.310012000 |
| 6 | 5.198795000 | -5.816842000 | -2.931334000 |
| 6 | 5.320978000 | -4.089953000 | -0.724580000 |
| 6 | 6.431392000 | -5.567041000 | -2.308934000 |
| 6 | 6.496441000 | -4.673221000 | -1.231763000 |
| 9 | 5.466326000 | -3.269763000 | 0.313440000 |
| 9 | 7.678813000 | -4.385479000 | -0.682474000 |

| | | | |
|---|---|---|---|
| 9 | 7.539221000 | -6.151712000 | -2.760635000 |
| 9 | 5.151675000 | -6.626639000 | -3.997101000 |
| 9 | 2.880656000 | -5.480101000 | -3.064819000 |
| 9 | -5.205753000 | 4.812640000 | -3.593299000 |
| 9 | -4.018076000 | 0.445138000 | -2.293582000 |
| 9 | -1.209923000 | 2.970357000 | -5.248204000 |
| 9 | -5.705270000 | 2.525461000 | -2.153464000 |
| 9 | -2.900665000 | 5.035655000 | -5.035519000 |
| 6 | -2.676125000 | -3.750633000 | 2.309884000 |
| 6 | -1.697923000 | -4.000141000 | 3.479987000 |
| 1 | -2.144998000 | -3.571852000 | 4.400595000 |
| 1 | -1.618286000 | -5.092733000 | 3.656180000 |
| 6 | 5.295633000 | 1.274999000 | -7.271266000 |
| 6 | 4.445851000 | 0.837351000 | -8.466241000 |
| 1 | 5.071625000 | 0.290268000 | -9.201867000 |
| 1 | 4.042581000 | 1.731766000 | -8.991651000 |
| 6 | -1.849196000 | -2.668331000 | -7.062551000 |
| 6 | -1.815116000 | -1.399100000 | -7.942901000 |
| 1 | -2.803514000 | -1.257852000 | -8.425864000 |
| 1 | -1.662292000 | -0.542338000 | -7.257516000 |
| 6 | 1.339416000 | 1.188513000 | 3.656029000 |
| 6 | 0.346037000 | 2.342893000 | 3.490357000 |
| 1 | -0.364112000 | 2.108065000 | 2.667110000 |
| 1 | -0.265420000 | 2.453015000 | 4.409667000 |
| 6 | 1.089045000 | 3.640234000 | 3.159712000 |
| 1 | 1.812814000 | 3.868805000 | 3.974066000 |
| 1 | 0.388609000 | 4.499843000 | 3.109295000 |
| 6 | 1.825073000 | 3.498440000 | 1.826621000 |
| 1 | 2.473363000 | 4.377219000 | 1.628477000 |
| 1 | 1.077561000 | 3.501654000 | 1.006484000 |
| 1 | 0.803603000 | 0.217381000 | 3.723410000 |
| 1 | 1.873895000 | 1.302485000 | 4.627636000 |
| 6 | -0.175746000 | -2.636914000 | 1.900206000 |
| 1 | -0.126252000 | -3.391246000 | 1.082553000 |
| 1 | 0.775532000 | -2.075584000 | 1.833973000 |
| 6 | -0.291368000 | -3.379089000 | 3.245762000 |
| 1 | 0.497760000 | -4.156657000 | 3.289197000 |
| 1 | -0.064115000 | -2.661644000 | 4.061527000 |
| 1 | -3.706479000 | -4.057704000 | 2.582047000 |
| 1 | -2.380806000 | -4.390087000 | 1.446221000 |
| 6 | 0.248386000 | -2.654063000 | -8.866968000 |
| 1 | -0.282062000 | -3.553903000 | -9.254853000 |
| 1 | 1.157312000 | -2.532132000 | -9.490445000 |
| 6 | -0.684669000 | -1.427001000 | -9.008380000 |
| 1 | -0.067866000 | -0.509894000 | -8.913643000 |
| 1 | -1.106919000 | -1.415498000 | -10.034469000 |
| 1 | -2.575356000 | -2.536222000 | -6.237439000 |

| | | | |
|---|---|---|---|
| 1 | -2.180497000 | -3.540946000 | -7.673093000 |
| 1 | 6.080133000 | 2.000435000 | -7.574156000 |
| 1 | 5.847500000 | 0.389658000 | -6.879091000 |
| 6 | 2.348810000 | 0.757822000 | -7.058267000 |
| 1 | 1.695059000 | 1.413626000 | -7.677731000 |
| 1 | 1.652582000 | 0.080098000 | -6.521345000 |
| 6 | 3.290810000 | -0.034009000 | -7.969342000 |
| 1 | 3.718651000 | -0.887579000 | -7.399820000 |
| 1 | 2.720460000 | -0.473271000 | -8.814004000 |
| 6 | -4.261247000 | -4.465550000 | -1.181803000 |
| 6 | -4.134810000 | -3.086144000 | -1.302363000 |
| 6 | -2.864310000 | -2.470442000 | -1.484624000 |
| 6 | -3.083931000 | -5.255702000 | -1.267600000 |
| 6 | -1.814717000 | -4.610180000 | -1.485648000 |
| 6 | -1.689053000 | -3.213687000 | -1.571179000 |
| 6 | -2.817872000 | -6.649461000 | -1.212412000 |
| 6 | -1.415412000 | -6.829749000 | -1.393626000 |
| 7 | -0.846953000 | -5.581804000 | -1.597384000 |
| 6 | -0.663402000 | -8.039901000 | -1.502988000 |
| 6 | -1.150085000 | -9.243600000 | -0.738551000 |
| 1 | -2.250701000 | -9.301303000 | -0.648136000 |
| 1 | -0.754071000 | -9.166222000 | 0.297713000 |
| 1 | -0.765861000 | -10.191399000 | -1.163236000 |
| 6 | 0.829901000 | -7.944160000 | -1.661042000 |
| 1 | 1.278016000 | -8.940769000 | -1.836232000 |
| 1 | 1.269378000 | -7.522462000 | -0.730292000 |
| 1 | 1.138876000 | -7.269861000 | -2.480650000 |
| 1 | 0.165987000 | -5.360433000 | -1.466367000 |
| 6 | -2.762170000 | -9.053354000 | -3.474256000 |
| 6 | -3.663350000 | -7.959550000 | -4.090852000 |
| 6 | -1.274194000 | -8.794713000 | -3.589348000 |
| 1 | -2.977017000 | -10.026545000 | -3.962716000 |
| 1 | -3.039245000 | -9.209436000 | -2.411509000 |
| 6 | -0.811413000 | -7.688139000 | -4.314923000 |
| 6 | -2.993927000 | -6.621903000 | -4.206277000 |
| 1 | -4.612334000 | -7.872429000 | -3.526280000 |
| 1 | -3.965774000 | -8.253605000 | -5.123245000 |
| 6 | -1.655684000 | -6.518650000 | -4.446728000 |
| 1 | -0.594755000 | -9.659227000 | -3.532003000 |
| 8 | 0.459655000 | -7.634476000 | -4.712707000 |
| 1 | -3.619308000 | -5.715062000 | -4.222410000 |
| 1 | -1.177750000 | -5.553738000 | -4.673410000 |
| 14 | 1.059209000 | -7.517288000 | -6.353254000 |
| 6 | 1.097167000 | -9.296641000 | -6.965195000 |
| 6 | -0.106739000 | -6.468138000 | -7.378969000 |
| 1 | -1.140159000 | -6.869030000 | -7.380115000 |
| 1 | 0.251860000 | -6.454101000 | -8.429637000 |

| | | | |
|---|---|---|---|
| 1 | -0.129181000 | -5.419680000 | -7.019101000 |
| 1 | 1.744746000 | -9.925404000 | -6.321361000 |
| 1 | 1.492857000 | -9.346158000 | -8.000799000 |
| 1 | 0.079808000 | -9.738893000 | -6.971458000 |
| 1 | -5.025090000 | -2.443074000 | -1.247144000 |
| 1 | -2.794067000 | -1.380541000 | -1.563151000 |
| 1 | -5.242775000 | -4.939085000 | -1.027374000 |
| 1 | -0.716871000 | -2.722438000 | -1.717380000 |
| 1 | -3.548845000 | -7.445892000 | -1.037580000 |
| 6 | 2.750308000 | -6.768695000 | -6.155437000 |
| 1 | 3.395942000 | -7.379969000 | -5.497312000 |
| 1 | 3.246775000 | -6.667421000 | -7.141949000 |
| 1 | 2.656645000 | -5.761792000 | -5.704999000 |

***<u>Minor Isomer</u>***

***TS-B1***

***Imaginary frequency = - 132.08***

| | | | |
|---|---|---|---|
| 6 | 2.262140000 | 2.106960000 | 2.421560000 |
| 6 | -3.965700000 | -0.369450000 | 0.942420000 |
| 6 | 2.717090000 | 0.768390000 | 2.340870000 |
| 6 | 2.767830000 | 3.051460000 | 1.506450000 |
| 6 | -2.679140000 | 0.220560000 | 0.949530000 |
| 6 | -4.154940000 | -1.701220000 | 1.328940000 |
| 1 | -4.829440000 | 0.213590000 | 0.593470000 |
| 6 | 3.617890000 | 0.404890000 | 1.323200000 |
| 6 | 3.667360000 | 2.684050000 | 0.503860000 |
| 1 | 2.427220000 | 4.095840000 | 1.572310000 |
| 6 | -2.533430000 | 1.650880000 | 0.574360000 |
| 6 | -1.586670000 | -0.584540000 | 1.351680000 |
| 6 | -3.063500000 | -2.493310000 | 1.718720000 |
| 1 | -5.166450000 | -2.136010000 | 1.308450000 |
| 6 | 4.095180000 | 1.342550000 | 0.385340000 |
| 1 | 3.936360000 | -0.644860000 | 1.259350000 |
| 1 | 4.004090000 | 3.433860000 | -0.226890000 |
| 6 | -1.359600000 | 2.189920000 | -0.052670000 |
| 6 | -3.574600000 | 2.551380000 | 0.813330000 |
| 6 | -1.767870000 | -1.922060000 | 1.731120000 |
| 1 | -0.573650000 | -0.163990000 | 1.374450000 |
| 6 | 4.987380000 | 0.960180000 | -0.735480000 |
| 8 | -0.272120000 | 1.351640000 | -0.267410000 |
| 6 | -1.234960000 | 3.530590000 | -0.439560000 |

| | | | |
|---|---|---|---|
| 1 | -4.470800000 | 2.200920000 | 1.345280000 |
| 6 | -3.540670000 | 3.902510000 | 0.379830000 |
| 6 | 4.740330000 | -0.214980000 | -1.512060000 |
| 6 | 6.054050000 | 1.758910000 | -1.139140000 |
| 15 | 0.051100000 | 0.871520000 | -1.811780000 |
| 6 | -2.371200000 | 4.401180000 | -0.297180000 |
| 6 | 0.056770000 | 4.040750000 | -0.979250000 |
| 6 | -4.657710000 | 4.771930000 | 0.566370000 |
| 8 | 3.722460000 | -1.062220000 | -1.106350000 |
| 6 | 5.460900000 | -0.571720000 | -2.650590000 |
| 1 | 6.296840000 | 2.660220000 | -0.555710000 |
| 6 | 6.830960000 | 1.463210000 | -2.293370000 |
| 8 | 0.011880000 | 2.312230000 | -2.619010000 |
| 7 | 1.517090000 | 0.294480000 | -1.745740000 |
| 7 | -1.181630000 | 0.000830000 | -2.385010000 |
| 6 | -2.401970000 | 5.726010000 | -0.825760000 |
| 6 | 0.738470000 | 5.145670000 | -0.356920000 |
| 6 | 0.655990000 | 3.416610000 | -2.075910000 |
| 1 | -5.542760000 | 4.382350000 | 1.092230000 |
| 6 | -4.643110000 | 6.065580000 | 0.072010000 |
| 15 | 2.285680000 | -1.078490000 | -1.901210000 |
| 6 | 5.082120000 | -1.787550000 | -3.424480000 |
| 6 | 6.527540000 | 0.295350000 | -3.085790000 |
| 6 | 7.878860000 | 2.335190000 | -2.715060000 |
| 16 | -1.948990000 | 0.278100000 | -3.765770000 |
| 6 | -3.512740000 | 6.537610000 | -0.647450000 |
| 1 | -1.535050000 | 6.095030000 | -1.390800000 |
| 6 | 0.290920000 | 5.753100000 | 0.853680000 |
| 6 | 1.962500000 | 5.622050000 | -0.951690000 |
| 6 | 1.886790000 | 3.845910000 | -2.667260000 |
| 1 | -5.516310000 | 6.720420000 | 0.210460000 |
| 8 | 2.794630000 | -1.069400000 | -3.482460000 |
| 7 | 1.559430000 | -2.454850000 | -1.509640000 |
| 6 | 3.769880000 | -1.962360000 | -3.894440000 |
| 6 | 6.064920000 | -2.792340000 | -3.731570000 |
| 6 | 7.262360000 | 0.087530000 | -4.290460000 |
| 1 | 8.102930000 | 3.219240000 | -2.097890000 |
| 6 | 8.588030000 | 2.092660000 | -3.881340000 |
| 8 | -2.981130000 | -0.796970000 | -3.931700000 |
| 8 | -1.082160000 | 0.535500000 | -4.938400000 |
| 1 | -3.521580000 | 7.550990000 | -1.075730000 |
| 1 | -0.614120000 | 5.368720000 | 1.342610000 |
| 6 | 0.996840000 | 6.798490000 | 1.432580000 |
| 6 | 2.651900000 | 6.713090000 | -0.342270000 |
| 6 | 2.485770000 | 4.971200000 | -2.101240000 |
| 6 | 2.537610000 | 3.116950000 | -3.783730000 |
| 16 | 2.039650000 | -3.473720000 | -0.349280000 |

| | | | |
|---|---|---|---|
| 6 | 3.403120000 | -2.991380000 | -4.830200000 |
| 6 | 5.730550000 | -3.819400000 | -4.682410000 |
| 6 | 7.345770000 | -2.833370000 | -3.107160000 |
| 6 | 8.266770000 | 0.963280000 | -4.679170000 |
| 1 | 7.012150000 | -0.768100000 | -4.930750000 |
| 1 | 9.387930000 | 2.779350000 | -4.196700000 |
| 1 | 0.638450000 | 7.241840000 | 2.373830000 |
| 6 | 2.181240000 | 7.293460000 | 0.825070000 |
| 1 | 3.581120000 | 7.072760000 | -0.811140000 |
| 1 | 3.413650000 | 5.360980000 | -2.546150000 |
| 6 | 1.815970000 | 2.538630000 | -4.848730000 |
| 6 | 3.947000000 | 3.005570000 | -3.802230000 |
| 8 | 2.516450000 | -2.818460000 | 0.887650000 |
| 8 | 1.007460000 | -4.530900000 | -0.204890000 |
| 6 | 4.424720000 | -3.855380000 | -5.233660000 |
| 6 | 2.048250000 | -3.140890000 | -5.429640000 |
| 6 | 6.711690000 | -4.796090000 | -5.026920000 |
| 6 | 8.267140000 | -3.816830000 | -3.438290000 |
| 1 | 7.588240000 | -2.085140000 | -2.340050000 |
| 1 | 8.810480000 | 0.784150000 | -5.619000000 |
| 1 | 2.730090000 | 8.126220000 | 1.289970000 |
| 6 | 2.458390000 | 1.856270000 | -5.899160000 |
| 1 | 0.722450000 | 2.621720000 | -4.876430000 |
| 6 | 4.592170000 | 2.333220000 | -4.840280000 |
| 1 | 4.542250000 | 3.420150000 | -2.975990000 |
| 1 | 4.217180000 | -4.609880000 | -6.004790000 |
| 6 | 1.672430000 | -4.399770000 | -5.965200000 |
| 6 | 1.142750000 | -2.065540000 | -5.596680000 |
| 1 | 6.444940000 | -5.573340000 | -5.759580000 |
| 6 | 7.958330000 | -4.793480000 | -4.421700000 |
| 1 | 9.241150000 | -3.844420000 | -2.927680000 |
| 6 | 3.868620000 | 1.732970000 | -5.889160000 |
| 1 | 5.689160000 | 2.258620000 | -4.837180000 |
| 6 | 0.513670000 | -4.559930000 | -6.733510000 |
| 1 | 2.308110000 | -5.278950000 | -5.781890000 |
| 6 | -0.035890000 | -2.226330000 | -6.340540000 |
| 1 | 1.362430000 | -1.081750000 | -5.167620000 |
| 1 | 8.699170000 | -5.565110000 | -4.678560000 |
| 6 | -0.345140000 | -3.452200000 | -6.949070000 |
| 1 | -0.712830000 | -1.364600000 | -6.444170000 |
| 6 | -3.451430000 | 4.155140000 | -3.854400000 |
| 6 | -4.565160000 | 4.056280000 | -3.006290000 |
| 6 | -2.646380000 | 3.028470000 | -4.106420000 |
| 6 | -4.844040000 | 2.839170000 | -2.365520000 |
| 6 | -2.927030000 | 1.796640000 | -3.486300000 |
| 6 | -4.013300000 | 1.736110000 | -2.596330000 |
| 6 | 3.223490000 | -5.220840000 | -2.174840000 |

| | | | |
|---|---|---|---|
| 6 | 3.462700000 | -4.360540000 | -1.087010000 |
| 6 | 4.237640000 | -6.028600000 | -2.701730000 |
| 6 | 4.766560000 | -4.290980000 | -0.563130000 |
| 6 | 5.519640000 | -5.998460000 | -2.130140000 |
| 6 | 5.791020000 | -5.105340000 | -1.083590000 |
| 9 | 5.097320000 | -3.464830000 | 0.426730000 |
| 9 | 7.024480000 | -5.037020000 | -0.575190000 |
| 9 | 6.479590000 | -6.794760000 | -2.601260000 |
| 9 | 3.992030000 | -6.821460000 | -3.756990000 |
| 9 | 2.016580000 | -5.291220000 | -2.756570000 |
| 9 | -5.344680000 | 5.116640000 | -2.805110000 |
| 9 | -4.296780000 | 0.606750000 | -1.936130000 |
| 9 | -1.625910000 | 3.196060000 | -4.945350000 |
| 9 | -5.884120000 | 2.732630000 | -1.531390000 |
| 9 | -3.165930000 | 5.324210000 | -4.432780000 |
| 6 | -3.165820000 | -3.933500000 | 2.142600000 |
| 6 | -2.420130000 | -4.175850000 | 3.474720000 |
| 1 | -3.036850000 | -3.759150000 | 4.297400000 |
| 1 | -2.356410000 | -5.268170000 | 3.659140000 |
| 6 | 4.595220000 | 0.940570000 | -6.956060000 |
| 6 | 3.790660000 | 0.800950000 | -8.251600000 |
| 1 | 4.303400000 | 0.103330000 | -8.946110000 |
| 1 | 3.741710000 | 1.785690000 | -8.768340000 |
| 6 | -1.509270000 | -3.671780000 | -7.879930000 |
| 6 | -1.022570000 | -4.355570000 | -9.174870000 |
| 1 | -1.891760000 | -4.581380000 | -9.825870000 |
| 1 | -0.398720000 | -3.626240000 | -9.730580000 |
| 6 | 1.213310000 | 2.517250000 | 3.435110000 |
| 6 | 1.118210000 | 1.560740000 | 4.628270000 |
| 1 | 2.018080000 | 1.678280000 | 5.272830000 |
| 1 | 0.241660000 | 1.822100000 | 5.256710000 |
| 6 | 1.027950000 | 0.111360000 | 4.140510000 |
| 1 | 0.127090000 | 0.003990000 | 3.498330000 |
| 1 | 0.885600000 | -0.587200000 | 4.991290000 |
| 6 | 2.281490000 | -0.277540000 | 3.350020000 |
| 1 | 2.150310000 | -1.249250000 | 2.830240000 |
| 1 | 3.123880000 | -0.436760000 | 4.062120000 |
| 1 | 1.402360000 | 3.559650000 | 3.768060000 |
| 1 | 0.226850000 | 2.547030000 | 2.914910000 |
| 6 | -0.646070000 | -2.815520000 | 2.182540000 |
| 1 | -0.446930000 | -3.577290000 | 1.398300000 |
| 1 | 0.301000000 | -2.253340000 | 2.281220000 |
| 6 | -1.005170000 | -3.531120000 | 3.500990000 |
| 1 | -0.231240000 | -4.295640000 | 3.715480000 |
| 1 | -0.949030000 | -2.791680000 | 4.326620000 |
| 1 | -4.225470000 | -4.252680000 | 2.212530000 |
| 1 | -2.700310000 | -4.565330000 | 1.351920000 |

| | | | |
|---|---|---|---|
| 6 | 0.120100000 | -5.853700000 | -7.398680000 |
| 1 | -0.780870000 | -6.258650000 | -6.886390000 |
| 1 | 0.913560000 | -6.617940000 | -7.273280000 |
| 6 | -0.199320000 | -5.644560000 | -8.898250000 |
| 1 | 0.755230000 | -5.598570000 | -9.460640000 |
| 1 | -0.737070000 | -6.538500000 | -9.276350000 |
| 1 | -2.023180000 | -2.715330000 | -8.101300000 |
| 1 | -2.264250000 | -4.331970000 | -7.395980000 |
| 1 | 5.592300000 | 1.391890000 | -7.142850000 |
| 1 | 4.802890000 | -0.076920000 | -6.548060000 |
| 6 | 1.629040000 | 1.320230000 | -7.052410000 |
| 1 | 1.315420000 | 2.189640000 | -7.675380000 |
| 1 | 0.686150000 | 0.890020000 | -6.657190000 |
| 6 | 2.373090000 | 0.314700000 | -7.937580000 |
| 1 | 2.435100000 | -0.666190000 | -7.418710000 |
| 1 | 1.797060000 | 0.134250000 | -8.869040000 |
| 6 | -6.464010000 | -4.405440000 | -0.260650000 |
| 6 | -7.443830000 | -3.418160000 | -0.217980000 |
| 6 | -7.256330000 | -2.169890000 | -0.878680000 |
| 6 | -5.263150000 | -4.145500000 | -0.971390000 |
| 6 | -5.084420000 | -2.867420000 | -1.618370000 |
| 6 | -6.088720000 | -1.876160000 | -1.581540000 |
| 6 | -4.090470000 | -4.898700000 | -1.234040000 |
| 6 | -3.224190000 | -4.081360000 | -2.004580000 |
| 7 | -3.846720000 | -2.854380000 | -2.208320000 |
| 6 | -1.938510000 | -4.416830000 | -2.548380000 |
| 6 | -1.247040000 | -5.619790000 | -1.998350000 |
| 1 | -1.935750000 | -6.429580000 | -1.699220000 |
| 1 | -0.654480000 | -5.286150000 | -1.116610000 |
| 1 | -0.500890000 | -6.006720000 | -2.714460000 |
| 6 | -1.044480000 | -3.337320000 | -3.057080000 |
| 1 | -0.186810000 | -3.748320000 | -3.618530000 |
| 1 | -0.599960000 | -2.811470000 | -2.181050000 |
| 1 | -1.562420000 | -2.576580000 | -3.664840000 |
| 1 | -3.443030000 | -2.044360000 | -2.726140000 |
| 6 | -2.659350000 | -6.417550000 | -4.437790000 |
| 6 | -3.882110000 | -7.031500000 | -3.958270000 |
| 6 | -2.629490000 | -5.045250000 | -4.682980000 |
| 6 | -3.902160000 | -4.299510000 | -4.991580000 |
| 6 | -5.029730000 | -6.300270000 | -3.859200000 |
| 6 | -5.183290000 | -4.904990000 | -4.372990000 |
| 1 | -1.696130000 | -4.640200000 | -5.098160000 |
| 1 | -5.923770000 | -6.769640000 | -3.415630000 |
| 1 | -5.583550000 | -4.273750000 | -3.547480000 |
| 1 | -8.380510000 | -3.599040000 | 0.329980000 |
| 1 | -8.055090000 | -1.414450000 | -0.829290000 |
| 1 | -6.609200000 | -5.370660000 | 0.247680000 |

| | | | |
|---|---|---|---|
| 1 | -5.942240000 | -0.907450000 | -2.073400000 |
| 1 | -3.867840000 | -5.910850000 | -0.885200000 |
| 1 | -3.792710000 | -3.230450000 | -4.719530000 |
| 1 | -3.993750000 | -4.292050000 | -6.101180000 |
| 1 | -6.009740000 | -4.905860000 | -5.117760000 |
| 1 | -3.856050000 | -8.075040000 | -3.611360000 |
| 8 | -1.522720000 | -7.105430000 | -4.551470000 |
| 14 | -0.971850000 | -8.726400000 | -4.268140000 |
| 6 | -1.331900000 | -9.266730000 | -2.501780000 |
| 1 | -0.812050000 | -8.614270000 | -1.772700000 |
| 1 | -2.412090000 | -9.272660000 | -2.255380000 |
| 1 | -0.952260000 | -10.300330000 | -2.359410000 |
| 6 | -1.833070000 | -9.819900000 | -5.532150000 |
| 1 | -1.645150000 | -9.446920000 | -6.559430000 |
| 1 | -1.452270000 | -10.860320000 | -5.471670000 |
| 1 | -2.929920000 | -9.850700000 | -5.373400000 |
| 6 | 0.869110000 | -8.573420000 | -4.569800000 |
| 1 | 1.382700000 | -9.535780000 | -4.369330000 |
| 1 | 1.072030000 | -8.287040000 | -5.620830000 |
| 1 | 1.320810000 | -7.799370000 | -3.917060000 |

***TS-B2***

***Imaginary frequency = -155.98***

| | | | |
|---|---|---|---|
| 6 | 1.752605000 | 1.323275000 | 2.194276000 |
| 6 | -1.723675000 | -0.802594000 | 0.167487000 |
| 6 | 2.611082000 | 2.307293000 | 1.648179000 |
| 6 | 1.917677000 | -0.014938000 | 1.788040000 |
| 6 | -2.186626000 | 0.348827000 | 0.840024000 |
| 6 | -1.744840000 | -2.054726000 | 0.794767000 |
| 1 | -1.398336000 | -0.739849000 | -0.876739000 |
| 6 | 3.541718000 | 1.932175000 | 0.662593000 |
| 6 | 2.881873000 | -0.386725000 | 0.851247000 |
| 1 | 1.254533000 | -0.792065000 | 2.200132000 |
| 6 | -2.229940000 | 1.703737000 | 0.227791000 |
| 6 | -2.662566000 | 0.199534000 | 2.165263000 |
| 6 | -2.200494000 | -2.190850000 | 2.113530000 |
| 1 | -1.392217000 | -2.939659000 | 0.246141000 |
| 6 | 3.696572000 | 0.595924000 | 0.238860000 |
| 1 | 4.141336000 | 2.724565000 | 0.185981000 |
| 1 | 2.971223000 | -1.443612000 | 0.582335000 |
| 6 | -1.123745000 | 2.270180000 | -0.486901000 |
| 6 | -3.337693000 | 2.527929000 | 0.430274000 |
| 6 | -2.666402000 | -1.046584000 | 2.805804000 |
| 1 | -3.009326000 | 1.087590000 | 2.717980000 |
| 6 | 4.666904000 | 0.273208000 | -0.836488000 |

| | | | |
|---|---|---|---|
| 8 | -0.032270000 | 1.450350000 | -0.761663000 |
| 6 | -1.069802000 | 3.618817000 | -0.870775000 |
| 1 | -4.210124000 | 2.122018000 | 0.964524000 |
| 6 | -3.397857000 | 3.863557000 | -0.051114000 |
| 6 | 4.423956000 | -0.759712000 | -1.798959000 |
| 6 | 5.846722000 | 0.997433000 | -1.008375000 |
| 15 | 0.154418000 | 1.048705000 | -2.354445000 |
| 6 | -2.252138000 | 4.425404000 | -0.719614000 |
| 6 | 0.211279000 | 4.198295000 | -1.365166000 |
| 6 | -4.566987000 | 4.665497000 | 0.113605000 |
| 8 | 3.282871000 | -1.539347000 | -1.671631000 |
| 6 | 5.259537000 | -1.051798000 | -2.875691000 |
| 1 | 6.104597000 | 1.778805000 | -0.277873000 |
| 6 | 6.729414000 | 0.780949000 | -2.101346000 |
| 8 | 0.266997000 | 2.528094000 | -3.064224000 |
| 7 | 1.518591000 | 0.268757000 | -2.484600000 |
| 7 | -1.234440000 | 0.396556000 | -2.874073000 |
| 6 | -2.347767000 | 5.751806000 | -1.236982000 |
| 6 | 0.830754000 | 5.322821000 | -0.707122000 |
| 6 | 0.887932000 | 3.589895000 | -2.424384000 |
| 1 | -5.436981000 | 4.225253000 | 0.624529000 |
| 6 | -4.616149000 | 5.962713000 | -0.370661000 |
| 15 | 2.003558000 | -1.231412000 | -2.657018000 |
| 6 | 4.909253000 | -2.168021000 | -3.800222000 |
| 6 | 6.436177000 | -0.246068000 | -3.071404000 |
| 6 | 7.890759000 | 1.591020000 | -2.281646000 |
| 16 | -1.941198000 | 0.775343000 | -4.263990000 |
| 6 | -3.498949000 | 6.505184000 | -1.060278000 |
| 1 | -1.496535000 | 6.169095000 | -1.792556000 |
| 6 | 0.270681000 | 5.972279000 | 0.434133000 |
| 6 | 2.108800000 | 5.785461000 | -1.192884000 |
| 6 | 2.176461000 | 3.997141000 | -2.891510000 |
| 1 | -5.526609000 | 6.566803000 | -0.242862000 |
| 8 | 2.718705000 | -1.240948000 | -4.154951000 |
| 7 | 0.994875000 | -2.470872000 | -2.500065000 |
| 6 | 3.667243000 | -2.210705000 | -4.456078000 |
| 6 | 5.853532000 | -3.230980000 | -4.029194000 |
| 6 | 7.300445000 | -0.385535000 | -4.196533000 |
| 1 | 8.108013000 | 2.368181000 | -1.532281000 |
| 6 | 8.718964000 | 1.421649000 | -3.380668000 |
| 8 | -1.014929000 | 1.015153000 | -5.394066000 |
| 8 | -3.033007000 | -0.226894000 | -4.476259000 |
| 1 | -3.554731000 | 7.521158000 | -1.478021000 |
| 1 | -0.673948000 | 5.603824000 | 0.852854000 |
| 6 | 0.911159000 | 7.045922000 | 1.036524000 |
| 6 | 2.737151000 | 6.895892000 | -0.553291000 |
| 6 | 2.743559000 | 5.106508000 | -2.267713000 |

| | | | |
|---|---|---|---|
| 6 | 2.912490000 | 3.248867000 | -3.939349000 |
| 16 | 1.029405000 | -3.572370000 | -1.306095000 |
| 6 | 3.352962000 | -3.191231000 | -5.459598000 |
| 6 | 5.580166000 | -4.191826000 | -5.065033000 |
| 6 | 7.034847000 | -3.396085000 | -3.246976000 |
| 6 | 8.413558000 | 0.429540000 | -4.349733000 |
| 1 | 7.067434000 | -1.139181000 | -4.960314000 |
| 1 | 9.605557000 | 2.060633000 | -3.509451000 |
| 1 | 0.456859000 | 7.523514000 | 1.917679000 |
| 6 | 2.151377000 | 7.521013000 | 0.535868000 |
| 1 | 3.709360000 | 7.239215000 | -0.939887000 |
| 1 | 3.721561000 | 5.471122000 | -2.616470000 |
| 6 | 2.292351000 | 2.764585000 | -5.108572000 |
| 6 | 4.297194000 | 3.018223000 | -3.774196000 |
| 8 | 1.253883000 | -3.010351000 | 0.041022000 |
| 8 | -0.141047000 | -4.465842000 | -1.488431000 |
| 6 | 4.351636000 | -4.117738000 | -5.768460000 |
| 6 | 2.066400000 | -3.271594000 | -6.203202000 |
| 6 | 6.532176000 | -5.219627000 | -5.335335000 |
| 6 | 7.926641000 | -4.425247000 | -3.513371000 |
| 1 | 7.224238000 | -2.705877000 | -2.413746000 |
| 1 | 9.058949000 | 0.309742000 | -5.232940000 |
| 1 | 2.650604000 | 8.373142000 | 1.021080000 |
| 6 | 3.015248000 | 2.072097000 | -6.097391000 |
| 1 | 1.218056000 | 2.932536000 | -5.265374000 |
| 6 | 5.020413000 | 2.327119000 | -4.747303000 |
| 1 | 4.802478000 | 3.355140000 | -2.856789000 |
| 1 | 4.175613000 | -4.838763000 | -6.579275000 |
| 6 | 1.322103000 | -2.143998000 | -6.606127000 |
| 6 | 1.599996000 | -4.544638000 | -6.618012000 |
| 1 | 6.311991000 | -5.941160000 | -6.137104000 |
| 6 | 7.689083000 | -5.330238000 | -4.581600000 |
| 1 | 8.822319000 | -4.544207000 | -2.885556000 |
| 6 | 4.398944000 | 1.836500000 | -5.912601000 |
| 1 | 6.096572000 | 2.152538000 | -4.600556000 |
| 6 | 0.198825000 | -2.250611000 | -7.453835000 |
| 1 | 1.621556000 | -1.146154000 | -6.258993000 |
| 6 | 0.480247000 | -4.661392000 | -7.441052000 |
| 1 | 2.110165000 | -5.450865000 | -6.263781000 |
| 1 | 8.408252000 | -6.137335000 | -4.786156000 |
| 6 | -0.226076000 | -3.527635000 | -7.893041000 |
| 1 | 0.135759000 | -5.662447000 | -7.747987000 |
| 6 | -3.143246000 | 4.745946000 | -4.328698000 |
| 6 | -4.364568000 | 4.698688000 | -3.641229000 |
| 6 | -2.385914000 | 3.574980000 | -4.512239000 |
| 6 | -4.801926000 | 3.489871000 | -3.079685000 |
| 6 | -2.827964000 | 2.346651000 | -3.981969000 |

| | | | |
|---|---|---|---|
| 6 | -4.027511000 | 2.333309000 | -3.245095000 |
| 6 | 2.470278000 | -5.381489000 | -2.873881000 |
| 6 | 2.501256000 | -4.583752000 | -1.715715000 |
| 6 | 3.567008000 | -6.168186000 | -3.249151000 |
| 6 | 3.677358000 | -4.574947000 | -0.941786000 |
| 6 | 4.721533000 | -6.175805000 | -2.451152000 |
| 6 | 4.786381000 | -5.356749000 | -1.315079000 |
| 9 | 3.814897000 | -3.829999000 | 0.152914000 |
| 9 | 5.904832000 | -5.326854000 | -0.585354000 |
| 9 | 5.760149000 | -6.940211000 | -2.785487000 |
| 9 | 3.526692000 | -6.900774000 | -4.371349000 |
| 9 | 1.396611000 | -5.395605000 | -3.673746000 |
| 9 | -5.096511000 | 5.803798000 | -3.507046000 |
| 9 | -4.485122000 | 1.210190000 | -2.680138000 |
| 9 | -1.254082000 | 3.697857000 | -5.202270000 |
| 9 | -5.951089000 | 3.440945000 | -2.400160000 |
| 9 | -2.704695000 | 5.910205000 | -4.814664000 |
| 6 | -2.205380000 | -3.494322000 | 2.866344000 |
| 6 | -3.544476000 | -3.741094000 | 3.598486000 |
| 1 | -4.274903000 | -4.135196000 | 2.863997000 |
| 1 | -3.397791000 | -4.539219000 | 4.354811000 |
| 6 | 5.199089000 | 1.044616000 | -6.925723000 |
| 6 | 4.567372000 | 1.050064000 | -8.320594000 |
| 1 | 5.119918000 | 0.362285000 | -8.993790000 |
| 1 | 4.651100000 | 2.067170000 | -8.764948000 |
| 6 | -1.448376000 | -3.710606000 | -8.772501000 |
| 6 | -1.975486000 | -2.409254000 | -9.388142000 |
| 1 | -1.332366000 | -2.118984000 | -10.248143000 |
| 1 | -2.993443000 | -2.570710000 | -9.799567000 |
| 6 | 0.664199000 | 1.690567000 | 3.180634000 |
| 6 | 0.317112000 | 3.181101000 | 3.146944000 |
| 1 | -0.198341000 | 3.418513000 | 2.189305000 |
| 1 | -0.394711000 | 3.429346000 | 3.961591000 |
| 6 | 1.595570000 | 4.016538000 | 3.259419000 |
| 1 | 2.117501000 | 3.746607000 | 4.204700000 |
| 1 | 1.362755000 | 5.099557000 | 3.324117000 |
| 6 | 2.514815000 | 3.763320000 | 2.059810000 |
| 1 | 3.533694000 | 4.157692000 | 2.260581000 |
| 1 | 2.143439000 | 4.344912000 | 1.191340000 |
| 1 | -0.231525000 | 1.067280000 | 2.977100000 |
| 1 | 0.996689000 | 1.416183000 | 4.208916000 |
| 6 | -3.158379000 | -1.263588000 | 4.213771000 |
| 1 | -2.289781000 | -1.470922000 | 4.880426000 |
| 1 | -3.643927000 | -0.348803000 | 4.610423000 |
| 6 | -4.127740000 | -2.463202000 | 4.261096000 |
| 1 | -4.418859000 | -2.665734000 | 5.312235000 |
| 1 | -5.056346000 | -2.164753000 | 3.731430000 |

| | | | |
|---|---|---|---|
| 1 | -1.962782000 | -4.337072000 | 2.189028000 |
| 1 | -1.385863000 | -3.460660000 | 3.620229000 |
| 6 | -0.532920000 | -0.997690000 | -7.886728000 |
| 1 | 0.043023000 | -0.521258000 | -8.713692000 |
| 1 | -0.542651000 | -0.266427000 | -7.055058000 |
| 6 | -1.961120000 | -1.272784000 | -8.362073000 |
| 1 | -2.594187000 | -1.536690000 | -7.485058000 |
| 1 | -2.401701000 | -0.348718000 | -8.788928000 |
| 1 | -2.246546000 | -4.189783000 | -8.159957000 |
| 1 | -1.217984000 | -4.453207000 | -9.567117000 |
| 1 | 6.243950000 | 1.418499000 | -6.950906000 |
| 1 | 5.270991000 | -0.008384000 | -6.565218000 |
| 6 | 2.304058000 | 1.647018000 | -7.369172000 |
| 1 | 2.105946000 | 2.564308000 | -7.969995000 |
| 1 | 1.301290000 | 1.247612000 | -7.111953000 |
| 6 | 3.092491000 | 0.650049000 | -8.225881000 |
| 1 | 3.028425000 | -0.365709000 | -7.779665000 |
| 1 | 2.633312000 | 0.573630000 | -9.233581000 |
| 6 | -5.396827000 | -3.905219000 | 0.146323000 |
| 6 | -5.909731000 | -2.814704000 | 0.840422000 |
| 6 | -5.610526000 | -1.481872000 | 0.437478000 |
| 6 | -4.567534000 | -3.666645000 | -0.981270000 |
| 6 | -4.287840000 | -2.308681000 | -1.380600000 |
| 6 | -4.807541000 | -1.207920000 | -0.667553000 |
| 6 | -3.881965000 | -4.507580000 | -1.894536000 |
| 6 | -3.191387000 | -3.672286000 | -2.808655000 |
| 7 | -3.453732000 | -2.348378000 | -2.470421000 |
| 6 | -2.342432000 | -4.058102000 | -3.895223000 |
| 6 | -1.880145000 | -5.481487000 | -3.937288000 |
| 1 | -2.657094000 | -6.208999000 | -3.640213000 |
| 1 | -1.042990000 | -5.545949000 | -3.207958000 |
| 1 | -1.486253000 | -5.742491000 | -4.936229000 |
| 6 | -1.406740000 | -3.066478000 | -4.490526000 |
| 1 | -0.937140000 | -3.456546000 | -5.408888000 |
| 1 | -0.582814000 | -2.900925000 | -3.750538000 |
| 1 | -1.862707000 | -2.081038000 | -4.688943000 |
| 1 | -3.130179000 | -1.523160000 | -3.004542000 |
| 6 | -4.261164000 | -5.227068000 | -5.653194000 |
| 6 | -5.323684000 | -5.710862000 | -4.793026000 |
| 6 | -3.927135000 | -3.876464000 | -5.628918000 |
| 6 | -4.952168000 | -2.850920000 | -5.216786000 |
| 6 | -6.073682000 | -4.838681000 | -4.058799000 |
| 6 | -6.007739000 | -3.352454000 | -4.203729000 |
| 1 | -3.142762000 | -3.547112000 | -6.323319000 |
| 8 | -3.511254000 | -6.061604000 | -6.383864000 |
| 1 | -6.811647000 | -5.240175000 | -3.344468000 |
| 1 | -5.856476000 | -2.910958000 | -3.192604000 |

| | | | |
|---|---|---|---|
| 14 | -3.578392000 | -7.754029000 | -6.748568000 |
| 6 | -2.144530000 | -7.946762000 | -7.938132000 |
| 6 | -3.310027000 | -8.777649000 | -5.192143000 |
| 1 | -2.349133000 | -8.512992000 | -4.707322000 |
| 1 | -3.267179000 | -9.853441000 | -5.462909000 |
| 1 | -4.118561000 | -8.654167000 | -4.444713000 |
| 1 | -2.253888000 | -7.259397000 | -8.800982000 |
| 1 | -2.082978000 | -8.984427000 | -8.324652000 |
| 1 | -1.186778000 | -7.710902000 | -7.431647000 |
| 1 | -6.551636000 | -2.977209000 | 1.719136000 |
| 1 | -6.017915000 | -0.642999000 | 1.021445000 |
| 1 | -5.618650000 | -4.935515000 | 0.463096000 |
| 1 | -4.570373000 | -0.179378000 | -0.960630000 |
| 1 | -3.829804000 | -5.599541000 | -1.871177000 |
| 6 | -5.237317000 | -8.107548000 | -7.559925000 |
| 1 | -5.381305000 | -7.460998000 | -8.449138000 |
| 1 | -5.283897000 | -9.164278000 | -7.895419000 |
| 1 | -6.086490000 | -7.933825000 | -6.869259000 |
| 1 | -7.020512000 | -2.992656000 | -4.491175000 |
| 1 | -4.453796000 | -1.930213000 | -4.857032000 |
| 1 | -5.459981000 | -2.538705000 | -6.158051000 |
| 1 | -5.463715000 | -6.795301000 | -4.673466000 |

***TS-B3***

***Imaginary frequency = - 180.71***

| | | | |
|---|---|---|---|
| 6 | 2.675074000 | 2.037722000 | 1.778308000 |
| 6 | -4.247079000 | -0.025278000 | 0.567345000 |
| 6 | 2.825943000 | 0.630220000 | 1.760285000 |
| 6 | 3.370104000 | 2.804384000 | 0.822457000 |
| 6 | -2.899737000 | 0.405538000 | 0.617458000 |
| 6 | -4.600652000 | -1.333690000 | 0.917770000 |
| 1 | -5.021252000 | 0.663631000 | 0.201030000 |
| 6 | 3.622050000 | 0.032329000 | 0.766636000 |
| 6 | 4.147654000 | 2.201656000 | -0.168163000 |
| 1 | 3.272918000 | 3.900161000 | 0.842857000 |
| 6 | -2.569730000 | 1.807829000 | 0.251348000 |
| 6 | -1.923864000 | -0.529970000 | 1.036392000 |
| 6 | -3.622666000 | -2.259597000 | 1.314705000 |
| 1 | -5.653441000 | -1.651272000 | 0.849105000 |
| 6 | 4.270344000 | 0.794165000 | -0.227553000 |
| 1 | 3.715243000 | -1.061061000 | 0.767595000 |
| 1 | 4.625003000 | 2.826466000 | -0.936988000 |
| 6 | -1.334236000 | 2.184140000 | -0.375452000 |
| 6 | -3.482413000 | 2.836056000 | 0.491771000 |

| | | | |
|---|---|---|---|
| 6 | -2.270898000 | -1.844631000 | 1.376960000 |
| 1 | -0.869030000 | -0.233685000 | 1.092856000 |
| 6 | 5.017451000 | 0.181570000 | -1.354517000 |
| 8 | -0.376949000 | 1.202417000 | -0.584685000 |
| 6 | -1.026797000 | 3.494651000 | -0.764475000 |
| 1 | -4.419084000 | 2.607748000 | 1.020743000 |
| 6 | -3.264464000 | 4.170920000 | 0.060341000 |
| 6 | 4.518716000 | -0.967114000 | -2.047960000 |
| 6 | 6.186209000 | 0.746415000 | -1.857715000 |
| 15 | -0.118603000 | 0.672011000 | -2.127722000 |
| 6 | -2.039791000 | 4.508477000 | -0.619872000 |
| 6 | 0.318233000 | 3.832560000 | -1.314145000 |
| 6 | -4.255461000 | 5.180662000 | 0.250425000 |
| 8 | 3.412720000 | -1.606562000 | -1.513199000 |
| 6 | 5.082186000 | -1.502017000 | -3.209323000 |
| 1 | 6.628758000 | 1.610254000 | -1.338873000 |
| 6 | 6.814845000 | 0.267854000 | -3.038996000 |
| 8 | 0.077987000 | 2.098469000 | -2.929334000 |
| 7 | 1.256371000 | -0.103893000 | -1.972936000 |
| 7 | -1.419106000 | -0.030592000 | -2.751438000 |
| 6 | -1.899723000 | 5.824725000 | -1.152102000 |
| 6 | 1.117166000 | 4.877485000 | -0.726741000 |
| 6 | 0.841674000 | 3.125780000 | -2.401636000 |
| 1 | -5.183664000 | 4.910823000 | 0.776902000 |
| 6 | -4.069888000 | 6.461719000 | -0.241721000 |
| 15 | 1.924818000 | -1.503154000 | -2.196892000 |
| 6 | 4.445650000 | -2.671628000 | -3.887822000 |
| 6 | 6.245614000 | -0.846796000 | -3.758973000 |
| 6 | 7.974787000 | 0.915068000 | -3.560348000 |
| 16 | -2.146738000 | 0.415663000 | -4.137803000 |
| 6 | -2.891238000 | 6.777273000 | -0.968470000 |
| 1 | -0.998969000 | 6.074406000 | -1.728737000 |
| 6 | 0.766148000 | 5.537490000 | 0.487506000 |
| 6 | 2.353763000 | 5.242314000 | -1.371358000 |
| 6 | 2.099389000 | 3.421824000 | -3.023739000 |
| 1 | -4.847919000 | 7.226700000 | -0.100816000 |
| 8 | 2.297344000 | -1.589909000 | -3.805716000 |
| 7 | 1.177245000 | -2.862728000 | -1.709319000 |
| 6 | 3.084595000 | -2.648822000 | -4.230278000 |
| 6 | 5.209595000 | -3.840114000 | -4.249830000 |
| 6 | 6.840932000 | -1.216418000 | -5.000603000 |
| 1 | 8.401319000 | 1.757381000 | -2.993715000 |
| 6 | 8.539689000 | 0.515942000 | -4.761687000 |
| 1 | -2.768432000 | 7.780917000 | -1.402015000 |
| 1 | -0.150140000 | 5.237997000 | 1.013467000 |
| 6 | 1.574705000 | 6.531038000 | 1.022173000 |
| 6 | 3.146990000 | 6.288969000 | -0.812190000 |

| | | | |
|---|---|---|---|
| 6 | 2.791698000 | 4.519934000 | -2.512314000 |
| 6 | 2.683068000 | 2.602373000 | -4.117497000 |
| 16 | 1.674425000 | -3.760610000 | -0.477044000 |
| 6 | 2.462871000 | -3.628069000 | -5.076587000 |
| 6 | 4.602449000 | -4.848104000 | -5.084641000 |
| 6 | 6.540007000 | -4.059474000 | -3.781983000 |
| 6 | 7.956041000 | -0.551527000 | -5.492056000 |
| 1 | 6.399522000 | -2.033970000 | -5.584596000 |
| 1 | 9.427844000 | 1.032177000 | -5.156037000 |
| 1 | 1.288876000 | 7.014784000 | 1.968340000 |
| 6 | 2.768623000 | 6.923484000 | 0.360798000 |
| 1 | 4.083270000 | 6.565460000 | -1.321752000 |
| 1 | 3.733073000 | 4.827683000 | -2.990983000 |
| 6 | 1.908243000 | 1.937021000 | -5.091525000 |
| 6 | 4.091995000 | 2.481560000 | -4.207067000 |
| 8 | 2.043790000 | -3.028884000 | 0.751054000 |
| 8 | 0.684543000 | -4.873791000 | -0.304046000 |
| 6 | 3.252747000 | -4.691082000 | -5.506453000 |
| 6 | 1.071678000 | -3.429576000 | -5.566084000 |
| 6 | 5.368370000 | -5.993672000 | -5.456839000 |
| 6 | 7.253733000 | -5.189241000 | -4.154284000 |
| 1 | 6.992758000 | -3.322648000 | -3.104421000 |
| 1 | 8.384685000 | -0.852318000 | -6.459860000 |
| 1 | 3.397459000 | 7.718910000 | 0.788355000 |
| 6 | 2.495172000 | 1.172138000 | -6.118269000 |
| 1 | 0.814179000 | 2.005259000 | -5.070420000 |
| 6 | 4.682687000 | 1.757490000 | -5.242146000 |
| 1 | 4.733953000 | 2.944045000 | -3.443624000 |
| 1 | 2.813345000 | -5.451297000 | -6.171350000 |
| 6 | 0.811164000 | -3.463222000 | -6.954794000 |
| 6 | 0.002581000 | -3.135088000 | -4.692052000 |
| 1 | 4.894753000 | -6.753174000 | -6.098584000 |
| 6 | 6.669099000 | -6.160865000 | -5.009425000 |
| 1 | 8.272316000 | -5.341779000 | -3.768063000 |
| 6 | 3.904692000 | 1.094815000 | -6.212528000 |
| 1 | 5.780132000 | 1.689636000 | -5.298648000 |
| 6 | -0.455721000 | -3.131039000 | -7.463872000 |
| 1 | 1.633909000 | -3.677547000 | -7.655581000 |
| 6 | -1.266187000 | -2.828111000 | -5.191004000 |
| 1 | 0.163190000 | -3.148992000 | -3.606969000 |
| 1 | 7.243761000 | -7.053706000 | -5.297584000 |
| 6 | -1.503616000 | -2.792413000 | -6.573800000 |
| 1 | -2.074139000 | -2.564491000 | -4.494978000 |
| 6 | -4.727114000 | 3.267515000 | -2.671428000 |
| 6 | -4.288677000 | 4.460807000 | -3.265034000 |
| 6 | -4.039710000 | 2.073367000 | -2.928025000 |
| 6 | -3.134585000 | 4.457007000 | -4.061810000 |

| | | | |
|---|---|---|---|
| 6 | -2.908407000 | 2.040871000 | -3.765067000 |
| 6 | -2.459111000 | 3.251321000 | -4.325434000 |
| 6 | 3.048532000 | -5.675483000 | -1.979326000 |
| 6 | 3.184039000 | -4.647577000 | -1.032297000 |
| 6 | 4.102454000 | -6.537297000 | -2.299179000 |
| 6 | 4.458924000 | -4.429792000 | -0.477449000 |
| 6 | 5.358136000 | -6.337155000 | -1.707736000 |
| 6 | 5.542029000 | -5.259524000 | -0.827588000 |
| 9 | 4.707386000 | -3.450002000 | 0.386222000 |
| 9 | 6.752807000 | -5.030705000 | -0.315128000 |
| 9 | 6.375645000 | -7.147102000 | -1.994837000 |
| 9 | 3.905105000 | -7.555708000 | -3.154625000 |
| 9 | 1.878183000 | -5.888000000 | -2.600341000 |
| 9 | -4.946352000 | 5.599858000 | -3.049803000 |
| 9 | -1.383175000 | 3.327294000 | -5.107581000 |
| 9 | -4.495493000 | 0.967598000 | -2.329688000 |
| 9 | -2.686245000 | 5.605121000 | -4.578920000 |
| 9 | -5.790823000 | 3.270807000 | -1.858986000 |
| 6 | -3.900721000 | -3.701380000 | 1.640756000 |
| 6 | -3.205095000 | -4.138882000 | 2.950214000 |
| 1 | -3.799658000 | -3.758989000 | 3.806164000 |
| 1 | -3.238343000 | -5.245797000 | 3.019937000 |
| 6 | 4.580515000 | 0.303443000 | -7.312320000 |
| 6 | 3.659990000 | 0.033971000 | -8.504134000 |
| 1 | 4.151706000 | -0.659204000 | -9.218362000 |
| 1 | 3.468833000 | 0.980475000 | -9.057716000 |
| 6 | -2.793298000 | -2.309660000 | -7.180661000 |
| 6 | -2.479400000 | -1.205460000 | -8.213734000 |
| 1 | -3.414358000 | -0.899194000 | -8.725836000 |
| 1 | -2.125364000 | -0.325269000 | -7.640146000 |
| 6 | 1.742280000 | 2.715582000 | 2.760116000 |
| 6 | 1.413829000 | 1.853443000 | 3.982584000 |
| 1 | 2.304844000 | 1.790251000 | 4.646819000 |
| 1 | 0.607756000 | 2.329647000 | 4.578445000 |
| 6 | 1.009784000 | 0.443499000 | 3.541937000 |
| 1 | 0.124265000 | 0.514077000 | 2.873167000 |
| 1 | 0.692739000 | -0.170834000 | 4.410269000 |
| 6 | 2.161470000 | -0.247077000 | 2.805037000 |
| 1 | 1.828125000 | -1.191242000 | 2.327897000 |
| 1 | 2.936932000 | -0.549769000 | 3.546240000 |
| 1 | 2.160991000 | 3.699975000 | 3.057381000 |
| 1 | 0.796761000 | 2.952460000 | 2.218264000 |
| 6 | -1.264803000 | -2.879068000 | 1.804361000 |
| 1 | -1.125845000 | -3.616757000 | 0.983395000 |
| 1 | -0.266129000 | -2.428707000 | 1.962716000 |
| 6 | -1.741732000 | -3.626360000 | 3.065315000 |
| 1 | -1.047695000 | -4.466451000 | 3.270955000 |

| | | | |
|---|---|---|---|
| 1 | -1.657586000 | -2.935399000 | 3.929382000 |
| 1 | -4.991027000 | -3.897553000 | 1.687062000 |
| 1 | -3.509810000 | -4.321030000 | 0.800619000 |
| 6 | -0.766071000 | -3.009082000 | -8.933634000 |
| 1 | -1.486986000 | -3.808214000 | -9.220292000 |
| 1 | 0.142263000 | -3.171797000 | -9.548770000 |
| 6 | -1.404666000 | -1.633576000 | -9.250777000 |
| 1 | -0.598100000 | -0.872851000 | -9.280533000 |
| 1 | -1.834800000 | -1.669649000 | -10.272995000 |
| 1 | -3.462646000 | -1.917035000 | -6.391554000 |
| 1 | -3.319517000 | -3.154098000 | -7.683887000 |
| 1 | 5.513481000 | 0.820159000 | -7.622120000 |
| 1 | 4.913476000 | -0.671747000 | -6.889449000 |
| 6 | 1.594858000 | 0.464063000 | -7.114031000 |
| 1 | 1.105668000 | 1.233002000 | -7.754799000 |
| 1 | 0.764382000 | -0.026233000 | -6.565303000 |
| 6 | 2.337671000 | -0.542965000 | -7.996055000 |
| 1 | 2.554954000 | -1.456643000 | -7.402034000 |
| 1 | 1.685980000 | -0.866829000 | -8.833760000 |
| 6 | -4.879465000 | -4.520528000 | -2.767333000 |
| 6 | -4.827609000 | -3.142706000 | -2.599657000 |
| 6 | -3.651000000 | -2.492262000 | -2.119471000 |
| 6 | -3.724126000 | -5.287840000 | -2.442080000 |
| 6 | -2.545645000 | -4.601711000 | -1.958706000 |
| 6 | -2.498685000 | -3.203777000 | -1.798907000 |
| 6 | -3.402861000 | -6.666144000 | -2.478968000 |
| 6 | -2.058102000 | -6.804156000 | -2.025933000 |
| 7 | -1.575417000 | -5.536618000 | -1.714912000 |
| 6 | -1.264524000 | -7.985137000 | -1.931233000 |
| 6 | -1.981944000 | -9.306283000 | -2.011592000 |
| 1 | -2.775521000 | -9.319168000 | -2.780424000 |
| 1 | -2.460437000 | -9.509943000 | -1.028823000 |
| 1 | -1.282511000 | -10.136717000 | -2.219640000 |
| 6 | -0.055323000 | -7.978569000 | -1.031224000 |
| 1 | 0.612634000 | -8.832777000 | -1.247078000 |
| 1 | -0.407114000 | -8.098452000 | 0.018128000 |
| 1 | 0.522428000 | -7.039269000 | -1.065863000 |
| 1 | -0.660323000 | -5.302108000 | -1.269318000 |
| 6 | -0.742053000 | -8.863507000 | -4.574187000 |
| 6 | -2.003373000 | -8.648291000 | -5.250196000 |
| 6 | -0.117034000 | -7.788879000 | -3.933228000 |
| 6 | -0.330361000 | -6.395300000 | -4.467774000 |
| 6 | -2.462856000 | -7.381774000 | -5.473939000 |
| 6 | -1.659841000 | -6.152849000 | -5.214856000 |
| 1 | 0.859083000 | -7.965907000 | -3.459440000 |
| 8 | -0.299674000 | -10.125837000 | -4.507319000 |
| 1 | -3.450466000 | -7.248254000 | -5.947028000 |

| | | | |
|---|---|---|---|
| 1 | -2.290724000 | -5.405712000 | -4.686996000 |
| 14 | 1.364285000 | -10.664631000 | -4.551820000 |
| 6 | 1.187687000 | -12.407553000 | -5.215161000 |
| 6 | 2.313293000 | -9.531588000 | -5.714273000 |
| 1 | 1.763435000 | -9.377961000 | -6.664738000 |
| 1 | 3.292184000 | -9.994483000 | -5.958367000 |
| 1 | 2.518770000 | -8.543168000 | -5.257630000 |
| 1 | 0.549680000 | -13.021511000 | -4.547866000 |
| 1 | 2.178856000 | -12.899856000 | -5.288749000 |
| 1 | 0.727132000 | -12.404477000 | -6.223287000 |
| 1 | -5.698922000 | -2.522782000 | -2.856510000 |
| 1 | -3.639257000 | -1.400083000 | -2.030845000 |
| 1 | -5.786595000 | -5.014723000 | -3.146798000 |
| 1 | -1.586568000 | -2.691245000 | -1.466010000 |
| 1 | -4.059484000 | -7.489076000 | -2.777482000 |
| 6 | 2.104941000 | -10.658317000 | -2.823436000 |
| 1 | 1.410295000 | -11.091202000 | -2.075764000 |
| 1 | 3.027586000 | -11.275599000 | -2.820340000 |
| 1 | 2.392660000 | -9.638052000 | -2.502067000 |
| 8 | -1.206192000 | 0.623996000 | -5.264096000 |
| 8 | -3.304485000 | -0.480858000 | -4.363626000 |
| 1 | -2.577453000 | -9.537883000 | -5.550302000 |
| 1 | -0.203726000 | -5.649731000 | -3.665180000 |
| 1 | 0.523608000 | -6.193894000 | -5.150551000 |
| 1 | -1.462432000 | -5.657147000 | -6.192052000 |

***TS-B4***

***Imaginary frequency = - 154.29***

| | | | |
|---|---|---|---|
| 6 | 2.520289000 | 2.300099000 | 2.031801000 |
| 6 | -3.966549000 | -0.443646000 | 0.843518000 |
| 6 | 2.925596000 | 0.943330000 | 2.026156000 |
| 6 | 3.065262000 | 3.173596000 | 1.070229000 |
| 6 | -2.681555000 | 0.150646000 | 0.835550000 |
| 6 | -4.143574000 | -1.775547000 | 1.236121000 |
| 1 | -4.831480000 | 0.133239000 | 0.486544000 |
| 6 | 3.813808000 | 0.491257000 | 1.033279000 |
| 6 | 3.940537000 | 2.714970000 | 0.084132000 |
| 1 | 2.769444000 | 4.233302000 | 1.081779000 |
| 6 | -2.537768000 | 1.570826000 | 0.422286000 |
| 6 | -1.584743000 | -0.646988000 | 1.240294000 |
| 6 | -3.045276000 | -2.564698000 | 1.614623000 |
| 1 | -5.150368000 | -2.222402000 | 1.211118000 |
| 6 | 4.313129000 | 1.352304000 | 0.034740000 |
| 1 | 4.099581000 | -0.569194000 | 1.035633000 |
| 1 | 4.296996000 | 3.408966000 | -0.690899000 |
| 6 | -1.363997000 | 2.086764000 | -0.225243000 |

| | | | |
|---|---|---|---|
| 6 | -3.575097000 | 2.478805000 | 0.642335000 |
| 6 | -1.754157000 | -1.985400000 | 1.619490000 |
| 1 | -0.573476000 | -0.222833000 | 1.250522000 |
| 6 | 5.150667000 | 0.876001000 | -1.093563000 |
| 8 | -0.284900000 | 1.237247000 | -0.414220000 |
| 6 | -1.231808000 | 3.416974000 | -0.643935000 |
| 1 | -4.470539000 | 2.146813000 | 1.187240000 |
| 6 | -3.537106000 | 3.817237000 | 0.171251000 |
| 6 | 4.834651000 | -0.328813000 | -1.797736000 |
| 6 | 6.217007000 | 1.621065000 | -1.588347000 |
| 15 | 0.070670000 | 0.717978000 | -1.943498000 |
| 6 | -2.367774000 | 4.293283000 | -0.522734000 |
| 6 | 0.062323000 | 3.917711000 | -1.189677000 |
| 6 | -4.651654000 | 4.693342000 | 0.337587000 |
| 8 | 3.834785000 | -1.133515000 | -1.273723000 |
| 6 | 5.470799000 | -0.756577000 | -2.964922000 |
| 1 | 6.517517000 | 2.539240000 | -1.061061000 |
| 6 | 6.915555000 | 1.254161000 | -2.770537000 |
| 8 | 0.061467000 | 2.141940000 | -2.774676000 |
| 7 | 1.541910000 | 0.152161000 | -1.762236000 |
| 7 | -1.102056000 | -0.185491000 | -2.558238000 |
| 6 | -2.398786000 | 5.602504000 | -1.088066000 |
| 6 | 0.718880000 | 5.056682000 | -0.602184000 |
| 6 | 0.681473000 | 3.270184000 | -2.262859000 |
| 1 | -5.536794000 | 4.318814000 | 0.874134000 |
| 6 | -4.634428000 | 5.974208000 | -0.188872000 |
| 15 | 2.341216000 | -1.187250000 | -1.950500000 |
| 6 | 5.013400000 | -1.995449000 | -3.661239000 |
| 6 | 6.524740000 | 0.072505000 | -3.502483000 |
| 6 | 7.966141000 | 2.074482000 | -3.279842000 |
| 16 | -1.894179000 | 0.093248000 | -3.950134000 |
| 6 | -3.506478000 | 6.421922000 | -0.926620000 |
| 1 | -1.536417000 | 5.952196000 | -1.671662000 |
| 6 | 0.273298000 | 5.676589000 | 0.602314000 |
| 6 | 1.914469000 | 5.559959000 | -1.230804000 |
| 6 | 1.897324000 | 3.716281000 | -2.878301000 |
| 1 | -5.505765000 | 6.634596000 | -0.066039000 |
| 8 | 2.720924000 | -1.259057000 | -3.560832000 |
| 7 | 1.708718000 | -2.583189000 | -1.418740000 |
| 6 | 3.662073000 | -2.177913000 | -3.997491000 |
| 6 | 5.949529000 | -3.019762000 | -4.053368000 |
| 6 | 7.174813000 | -0.190619000 | -4.743842000 |
| 1 | 8.257106000 | 2.966786000 | -2.703936000 |
| 6 | 8.592205000 | 1.775297000 | -4.479950000 |
| 8 | -1.003771000 | 0.414184000 | -5.090344000 |
| 8 | -2.895663000 | -0.981639000 | -4.138006000 |
| 1 | -3.515042000 | 7.421966000 | -1.385147000 |

| | | | |
|---|---|---|---|
| 1 | -0.608504000 | 5.272707000 | 1.117389000 |
| 6 | 0.952657000 | 6.759467000 | 1.143207000 |
| 6 | 2.572646000 | 6.692954000 | -0.664576000 |
| 6 | 2.447887000 | 4.891088000 | -2.364119000 |
| 6 | 2.581096000 | 2.970931000 | -3.966448000 |
| 16 | 2.347879000 | -3.505199000 | -0.268944000 |
| 6 | 3.202284000 | -3.224647000 | -4.866665000 |
| 6 | 5.511676000 | -4.075039000 | -4.932838000 |
| 6 | 7.292028000 | -3.053235000 | -3.570536000 |
| 6 | 8.180128000 | 0.638413000 | -5.222332000 |
| 1 | 6.864257000 | -1.058604000 | -5.338584000 |
| 1 | 9.394543000 | 2.422998000 | -4.864130000 |
| 1 | 0.597864000 | 7.210838000 | 2.082010000 |
| 6 | 2.104265000 | 7.284042000 | 0.498476000 |
| 1 | 3.478867000 | 7.074186000 | -1.160718000 |
| 1 | 3.350548000 | 5.308248000 | -2.834472000 |
| 6 | 1.895468000 | 2.205431000 | -4.933754000 |
| 6 | 3.993654000 | 3.028233000 | -4.057937000 |
| 8 | 2.737211000 | -2.804167000 | 0.970352000 |
| 8 | 1.458835000 | -4.698692000 | -0.094023000 |
| 6 | 4.151032000 | -4.125846000 | -5.342453000 |
| 6 | 1.788727000 | -3.268285000 | -5.326370000 |
| 6 | 6.450422000 | -5.066770000 | -5.347850000 |
| 6 | 8.175165000 | -4.042048000 | -3.978675000 |
| 1 | 7.617123000 | -2.288051000 | -2.852342000 |
| 1 | 8.654097000 | 0.413919000 | -6.189709000 |
| 1 | 2.630485000 | 8.148025000 | 0.931572000 |
| 6 | 2.572810000 | 1.509068000 | -5.953191000 |
| 1 | 0.801520000 | 2.136944000 | -4.911899000 |
| 6 | 4.669938000 | 2.373115000 | -5.086593000 |
| 1 | 4.573541000 | 3.574180000 | -3.299780000 |
| 1 | 3.830683000 | -4.930747000 | -6.021517000 |
| 6 | 1.507794000 | -3.347238000 | -6.709742000 |
| 6 | 0.704809000 | -3.178004000 | -4.427573000 |
| 1 | 6.105508000 | -5.862972000 | -6.025304000 |
| 6 | 7.758751000 | -5.046190000 | -4.892659000 |
| 1 | 9.200167000 | -4.055703000 | -3.579533000 |
| 6 | 3.981150000 | 1.605511000 | -6.047304000 |
| 1 | 5.767075000 | 2.443270000 | -5.143523000 |
| 6 | 0.190631000 | -3.264987000 | -7.188887000 |
| 1 | 2.339888000 | -3.401294000 | -7.429540000 |
| 6 | -0.610930000 | -3.115950000 | -4.897324000 |
| 1 | 0.890433000 | -3.157563000 | -3.346914000 |
| 1 | 8.468176000 | -5.823788000 | -5.212664000 |
| 6 | -0.882942000 | -3.135388000 | -6.273700000 |
| 1 | -1.437025000 | -2.997827000 | -4.182832000 |
| 6 | -3.468644000 | 3.946259000 | -3.946438000 |

| | | | |
|---|---|---|---|
| 6 | -4.600398000 | 3.800275000 | -3.131402000 |
| 6 | -2.631276000 | 2.844440000 | -4.200205000 |
| 6 | -4.855653000 | 2.567829000 | -2.511165000 |
| 6 | -2.895193000 | 1.592824000 | -3.613000000 |
| 6 | -4.009820000 | 1.478596000 | -2.760808000 |
| 6 | 3.723554000 | -5.182743000 | -2.009731000 |
| 6 | 3.866659000 | -4.241422000 | -0.977091000 |
| 6 | 4.798342000 | -5.956534000 | -2.458994000 |
| 6 | 5.153868000 | -4.010823000 | -0.459777000 |
| 6 | 6.069011000 | -5.758583000 | -1.895411000 |
| 6 | 6.251353000 | -4.759324000 | -0.927370000 |
| 9 | 5.396744000 | -3.093832000 | 0.472579000 |
| 9 | 7.474622000 | -4.526737000 | -0.446678000 |
| 9 | 7.103940000 | -6.494066000 | -2.296805000 |
| 9 | 4.617895000 | -6.871900000 | -3.420742000 |
| 9 | 2.526214000 | -5.389041000 | -2.587664000 |
| 9 | -5.410892000 | 4.837605000 | -2.923373000 |
| 9 | -4.294028000 | 0.330536000 | -2.135157000 |
| 9 | -1.586892000 | 3.059104000 | -4.999195000 |
| 9 | -5.896968000 | 2.436176000 | -1.680060000 |
| 9 | -3.196404000 | 5.136494000 | -4.490276000 |
| 6 | -3.129248000 | -4.024437000 | 1.966169000 |
| 6 | -2.334213000 | -4.356391000 | 3.250236000 |
| 1 | -2.941822000 | -4.051878000 | 4.126938000 |
| 1 | -2.218998000 | -5.457624000 | 3.323603000 |
| 6 | 4.750155000 | 0.884249000 | -7.134399000 |
| 6 | 3.871917000 | 0.482488000 | -8.321272000 |
| 1 | 4.446455000 | -0.159063000 | -9.021770000 |
| 1 | 3.568957000 | 1.388256000 | -8.892840000 |
| 6 | -2.252128000 | -2.896356000 | -6.852209000 |
| 6 | -2.167802000 | -1.737294000 | -7.870280000 |
| 1 | -3.151643000 | -1.605414000 | -8.365207000 |
| 1 | -1.975471000 | -0.815352000 | -7.285741000 |
| 6 | 1.476627000 | 2.803931000 | 3.007191000 |
| 6 | 1.316428000 | 1.910487000 | 4.241158000 |
| 1 | 2.204911000 | 2.023586000 | 4.902168000 |
| 1 | 0.436661000 | 2.235286000 | 4.834541000 |
| 6 | 1.181443000 | 0.443829000 | 3.820523000 |
| 1 | 0.294658000 | 0.339195000 | 3.158396000 |
| 1 | 0.989343000 | -0.206954000 | 4.698959000 |
| 6 | 2.438180000 | -0.029907000 | 3.083570000 |
| 1 | 2.284496000 | -1.025462000 | 2.618461000 |
| 1 | 3.259337000 | -0.174164000 | 3.823327000 |
| 1 | 1.705270000 | 3.852293000 | 3.292868000 |
| 1 | 0.503135000 | 2.853430000 | 2.464744000 |
| 6 | -0.612467000 | -2.877909000 | 2.022924000 |
| 1 | -0.411913000 | -3.606466000 | 1.206761000 |

| | | | |
|---|---|---|---|
| 1 | 0.326836000 | -2.303828000 | 2.138369000 |
| 6 | -0.947526000 | -3.655746000 | 3.310112000 |
| 1 | -0.143636000 | -4.394255000 | 3.504714000 |
| 1 | -0.927707000 | -2.943074000 | 4.160460000 |
| 1 | -4.184112000 | -4.353865000 | 2.057177000 |
| 1 | -2.696998000 | -4.600140000 | 1.114837000 |
| 6 | -0.169261000 | -3.187159000 | -8.649819000 |
| 1 | -0.743735000 | -4.097621000 | -8.935369000 |
| 1 | 0.739393000 | -3.176955000 | -9.285395000 |
| 6 | -1.046894000 | -1.940860000 | -8.927190000 |
| 1 | -0.388312000 | -1.048474000 | -8.941514000 |
| 1 | -1.476413000 | -2.027405000 | -9.946669000 |
| 1 | -2.969033000 | -2.644919000 | -6.047288000 |
| 1 | -2.621121000 | -3.815757000 | -7.364672000 |
| 1 | 5.613233000 | 1.505767000 | -7.454134000 |
| 1 | 5.199286000 | -0.035624000 | -6.694790000 |
| 6 | 1.766946000 | 0.686012000 | -6.941195000 |
| 1 | 1.195926000 | 1.383229000 | -7.596281000 |
| 1 | 0.996426000 | 0.111563000 | -6.387346000 |
| 6 | 2.627059000 | -0.240677000 | -7.803829000 |
| 1 | 2.950840000 | -1.110027000 | -7.192050000 |
| 1 | 2.021214000 | -0.655239000 | -8.636335000 |
| 6 | -4.105053000 | -4.799909000 | -2.109016000 |
| 6 | -4.074387000 | -3.418571000 | -1.979601000 |
| 6 | -2.863179000 | -2.728989000 | -1.672607000 |
| 6 | -2.892212000 | -5.522591000 | -1.920034000 |
| 6 | -1.675946000 | -4.797592000 | -1.628421000 |
| 6 | -1.654349000 | -3.397146000 | -1.499365000 |
| 6 | -2.544017000 | -6.891165000 | -1.936829000 |
| 6 | -1.150231000 | -6.993471000 | -1.649688000 |
| 7 | -0.651937000 | -5.701841000 | -1.500877000 |
| 6 | -0.353481000 | -8.164026000 | -1.516083000 |
| 6 | -1.031026000 | -9.445178000 | -1.117245000 |
| 1 | -2.050563000 | -9.547397000 | -1.528219000 |
| 1 | -1.118171000 | -9.456260000 | -0.007638000 |
| 1 | -0.444352000 | -10.335733000 | -1.412031000 |
| 6 | 1.071197000 | -8.008591000 | -1.056767000 |
| 1 | 1.628178000 | -8.961920000 | -1.122871000 |
| 1 | 1.075315000 | -7.686240000 | 0.008774000 |
| 1 | 1.617482000 | -7.227965000 | -1.613842000 |
| 1 | 0.270308000 | -5.417934000 | -1.102763000 |
| 6 | -1.023124000 | -9.101461000 | -4.236264000 |
| 6 | -1.770007000 | -8.065144000 | -4.928513000 |
| 6 | 0.259458000 | -8.836239000 | -3.761466000 |
| 6 | 1.034433000 | -7.698325000 | -4.376211000 |
| 6 | -1.228967000 | -6.827684000 | -5.114973000 |
| 6 | 0.163814000 | -6.480232000 | -4.719453000 |

| | | | |
|---|---|---|---|
| 1 | 0.818091000 | -9.686119000 | -3.341283000 |
| 8 | -1.588483000 | -10.282981000 | -3.954759000 |
| 1 | -1.844834000 | -6.020478000 | -5.545621000 |
| 1 | 0.091050000 | -5.771138000 | -3.865486000 |
| 14 | -3.118812000 | -11.002889000 | -4.348443000 |
| 6 | -2.959893000 | -12.700906000 | -3.574639000 |
| 6 | -4.513837000 | -10.022875000 | -3.547525000 |
| 1 | -4.375693000 | -9.960525000 | -2.448744000 |
| 1 | -5.479579000 | -10.538803000 | -3.730206000 |
| 1 | -4.605461000 | -8.992700000 | -3.944551000 |
| 1 | -2.104818000 | -13.255891000 | -4.009877000 |
| 1 | -3.879621000 | -13.297047000 | -3.744737000 |
| 1 | -2.798957000 | -12.623926000 | -2.480452000 |
| 1 | -4.988491000 | -2.824977000 | -2.124715000 |
| 1 | -2.871365000 | -1.636436000 | -1.591916000 |
| 1 | -5.038544000 | -5.331943000 | -2.347462000 |
| 1 | -0.722365000 | -2.851349000 | -1.299886000 |
| 1 | -3.212362000 | -7.733632000 | -2.141087000 |
| 6 | -3.280678000 | -11.095558000 | -6.218151000 |
| 1 | -2.405477000 | -11.615534000 | -6.657757000 |
| 1 | -4.189278000 | -11.671844000 | -6.490128000 |
| 1 | -3.359504000 | -10.097369000 | -6.692194000 |
| 1 | -2.807599000 | -8.265297000 | -5.231589000 |
| 1 | 1.885967000 | -7.393947000 | -3.739286000 |
| 1 | 1.504562000 | -8.090776000 | -5.309352000 |
| 1 | 0.630293000 | -5.859739000 | -5.513450000 |

***TS-B5***

***Imaginary frequency = - 189.40***

| | | | |
|---|---|---|---|
| 6 | 2.745487000 | 1.668265000 | 2.189129000 |
| 6 | -4.131128000 | 0.033673000 | 0.645158000 |
| 6 | 3.448307000 | 2.537900000 | 1.321407000 |
| 6 | 2.868229000 | 0.278205000 | 1.994256000 |
| 6 | -2.809621000 | 0.538985000 | 0.679942000 |
| 6 | -4.415904000 | -1.272955000 | 1.059018000 |
| 1 | -4.937867000 | 0.658898000 | 0.237025000 |
| 6 | 4.172659000 | 1.991242000 | 0.245459000 |
| 6 | 3.623232000 | -0.257261000 | 0.950769000 |
| 1 | 2.343348000 | -0.409977000 | 2.676828000 |
| 6 | -2.551664000 | 1.926250000 | 0.213948000 |
| 6 | -1.787156000 | -0.314790000 | 1.159840000 |
| 6 | -3.389980000 | -2.125117000 | 1.497023000 |
| 1 | -5.449118000 | -1.651131000 | 1.001408000 |
| 6 | 4.265712000 | 0.602050000 | 0.027318000 |
| 1 | 4.645887000 | 2.682675000 | -0.470677000 |

| | | | |
|---|---|---|---|
| 1 | 3.684906000 | -1.345616000 | 0.846188000 |
| 6 | -1.337999000 | 2.308695000 | -0.449801000 |
| 6 | -3.514111000 | 2.923868000 | 0.375109000 |
| 6 | -2.063469000 | -1.632988000 | 1.548897000 |
| 1 | -0.751792000 | 0.045835000 | 1.220046000 |
| 6 | 4.986511000 | 0.091308000 | -1.165690000 |
| 8 | -0.341460000 | 1.353662000 | -0.591093000 |
| 6 | -1.095140000 | 3.597388000 | -0.944965000 |
| 1 | -4.437629000 | 2.693357000 | 0.926082000 |
| 6 | -3.363576000 | 4.227234000 | -0.167296000 |
| 6 | 4.484125000 | -1.016658000 | -1.920082000 |
| 6 | 6.144406000 | 0.691948000 | -1.652184000 |
| 15 | -0.097015000 | 0.705650000 | -2.090988000 |
| 6 | -2.159376000 | 4.566400000 | -0.882102000 |
| 6 | 0.235526000 | 3.953674000 | -1.515673000 |
| 6 | -4.402476000 | 5.200099000 | -0.054540000 |
| 8 | 3.369455000 | -1.670656000 | -1.426188000 |
| 6 | 5.053310000 | -1.505575000 | -3.097960000 |
| 1 | 6.589647000 | 1.528771000 | -1.093031000 |
| 6 | 6.771700000 | 0.269596000 | -2.856017000 |
| 8 | 0.102220000 | 2.077370000 | -2.984246000 |
| 7 | 1.265009000 | -0.085238000 | -1.884431000 |
| 7 | -1.404516000 | -0.025272000 | -2.669545000 |
| 6 | -2.089877000 | 5.836253000 | -1.529216000 |
| 6 | 0.969858000 | 5.096104000 | -1.030511000 |
| 6 | 0.811283000 | 3.170717000 | -2.522459000 |
| 1 | -5.313830000 | 4.930791000 | 0.500789000 |
| 6 | -4.283302000 | 6.442117000 | -0.655281000 |
| 15 | 1.886873000 | -1.509684000 | -2.109015000 |
| 6 | 4.426467000 | -2.659909000 | -3.810839000 |
| 6 | 6.219465000 | -0.828652000 | -3.614075000 |
| 6 | 7.927191000 | 0.945331000 | -3.350389000 |
| 16 | -2.128385000 | 0.352567000 | -4.077360000 |
| 6 | -3.126024000 | 6.751592000 | -1.418412000 |
| 1 | -1.207918000 | 6.077996000 | -2.137397000 |
| 6 | 0.547015000 | 5.882297000 | 0.083091000 |
| 6 | 2.212671000 | 5.442481000 | -1.675457000 |
| 6 | 2.072122000 | 3.458687000 | -3.140575000 |
| 1 | -5.097724000 | 7.177101000 | -0.572768000 |
| 8 | 2.265228000 | -1.599622000 | -3.718327000 |
| 7 | 1.097016000 | -2.840172000 | -1.613078000 |
| 6 | 3.064448000 | -2.645751000 | -4.153078000 |
| 6 | 5.204502000 | -3.812681000 | -4.193928000 |
| 6 | 6.835474000 | -1.162852000 | -4.855363000 |
| 1 | 8.339279000 | 1.776261000 | -2.756897000 |
| 6 | 8.509641000 | 0.583454000 | -4.555197000 |
| 1 | -3.057119000 | 7.717351000 | -1.940648000 |

| | | | |
|---|---|---|---|
| 1 | -0.367605000 | 5.600989000 | 0.620788000 |
| 6 | 1.279236000 | 6.984061000 | 0.503237000 |
| 6 | 2.932743000 | 6.591439000 | -1.229010000 |
| 6 | 2.719864000 | 4.617879000 | -2.714231000 |
| 6 | 2.686950000 | 2.583617000 | -4.170789000 |
| 16 | 1.617426000 | -3.807297000 | -0.442104000 |
| 6 | 2.456859000 | -3.620488000 | -5.015833000 |
| 6 | 4.614458000 | -4.806619000 | -5.056742000 |
| 6 | 6.531158000 | -4.033860000 | -3.716054000 |
| 6 | 7.949297000 | -0.474764000 | -5.316607000 |
| 1 | 6.410831000 | -1.971488000 | -5.463413000 |
| 1 | 9.395374000 | 1.120313000 | -4.926784000 |
| 1 | 0.932087000 | 7.569008000 | 1.368214000 |
| 6 | 2.475435000 | 7.354136000 | -0.166111000 |
| 1 | 3.873757000 | 6.849117000 | -1.739569000 |
| 1 | 3.663152000 | 4.910976000 | -3.198547000 |
| 6 | 1.931511000 | 1.885287000 | -5.135894000 |
| 6 | 4.096973000 | 2.462320000 | -4.222419000 |
| 8 | 2.066685000 | -3.146602000 | 0.798051000 |
| 8 | 0.593862000 | -4.890514000 | -0.270099000 |
| 6 | 3.262243000 | -4.660836000 | -5.471828000 |
| 6 | 1.060128000 | -3.445559000 | -5.495396000 |
| 6 | 5.396169000 | -5.932583000 | -5.455215000 |
| 6 | 7.259345000 | -5.146898000 | -4.110212000 |
| 1 | 6.968811000 | -3.313457000 | -3.011595000 |
| 1 | 8.394215000 | -0.748815000 | -6.285008000 |
| 1 | 3.045081000 | 8.232963000 | 0.171506000 |
| 6 | 2.540254000 | 1.095975000 | -6.129884000 |
| 1 | 0.836600000 | 1.949686000 | -5.134668000 |
| 6 | 4.709554000 | 1.710875000 | -5.225472000 |
| 1 | 4.718757000 | 2.949524000 | -3.456631000 |
| 1 | 2.830318000 | -5.415088000 | -6.148101000 |
| 6 | 0.791051000 | -3.480628000 | -6.882239000 |
| 6 | -0.008810000 | -3.179179000 | -4.612689000 |
| 1 | 4.935828000 | -6.679262000 | -6.120963000 |
| 6 | 6.695186000 | -6.097167000 | -5.002354000 |
| 1 | 8.274025000 | -5.301930000 | -3.714691000 |
| 6 | 3.951792000 | 1.024510000 | -6.196332000 |
| 1 | 5.807964000 | 1.643899000 | -5.258021000 |
| 6 | -0.484565000 | -3.169960000 | -7.381624000 |
| 1 | 1.612583000 | -3.675992000 | -7.589549000 |
| 6 | -1.286475000 | -2.895099000 | -5.102063000 |
| 1 | 0.160148000 | -3.192260000 | -3.528850000 |
| 1 | 7.282288000 | -6.975115000 | -5.310452000 |
| 6 | -1.532629000 | -2.855162000 | -6.483320000 |
| 1 | -2.093946000 | -2.647699000 | -4.399432000 |
| 6 | -4.825959000 | 3.205437000 | -2.848539000 |

| | | | |
|---|---|---|---|
| 6 | -4.426079000 | 4.364727000 | -3.530127000 |
| 6 | -4.077466000 | 2.028666000 | -2.987550000 |
| 6 | -3.248837000 | 4.351388000 | -4.292020000 |
| 6 | -2.925376000 | 1.979936000 | -3.795208000 |
| 6 | -2.511775000 | 3.161941000 | -4.437483000 |
| 6 | 2.851464000 | -5.692187000 | -2.095112000 |
| 6 | 3.066805000 | -4.726282000 | -1.099627000 |
| 6 | 3.847192000 | -6.593268000 | -2.484684000 |
| 6 | 4.363607000 | -4.610471000 | -0.565475000 |
| 6 | 5.122378000 | -6.502563000 | -1.908342000 |
| 6 | 5.388079000 | -5.483288000 | -0.980936000 |
| 9 | 4.688608000 | -3.688108000 | 0.335651000 |
| 9 | 6.620442000 | -5.352434000 | -0.486031000 |
| 9 | 6.082712000 | -7.357331000 | -2.255918000 |
| 9 | 3.576321000 | -7.547372000 | -3.393964000 |
| 9 | 1.655383000 | -5.804779000 | -2.693235000 |
| 9 | -5.141203000 | 5.485280000 | -3.428631000 |
| 9 | -1.415905000 | 3.225451000 | -5.193354000 |
| 9 | -4.500667000 | 0.955014000 | -2.310955000 |
| 9 | -2.835303000 | 5.475092000 | -4.886545000 |
| 9 | -5.911232000 | 3.224686000 | -2.065989000 |
| 6 | -3.589662000 | -3.564219000 | 1.886946000 |
| 6 | -2.880740000 | -3.896072000 | 3.219626000 |
| 1 | -3.490240000 | -3.483902000 | 4.049839000 |
| 1 | -2.874814000 | -4.996826000 | 3.358822000 |
| 6 | 4.648392000 | 0.216453000 | -7.271098000 |
| 6 | 3.747962000 | -0.076513000 | -8.472942000 |
| 1 | 4.255315000 | -0.776760000 | -9.169055000 |
| 1 | 3.558852000 | 0.860348000 | -9.043350000 |
| 6 | -2.832597000 | -2.390072000 | -7.081656000 |
| 6 | -2.544405000 | -1.268402000 | -8.104362000 |
| 1 | -3.484770000 | -0.989085000 | -8.622021000 |
| 1 | -2.222041000 | -0.382260000 | -7.521864000 |
| 6 | 1.875216000 | 2.205035000 | 3.307635000 |
| 6 | 1.519970000 | 3.683776000 | 3.130923000 |
| 1 | 0.802067000 | 3.796557000 | 2.288040000 |
| 1 | 1.008940000 | 4.066094000 | 4.038626000 |
| 6 | 2.783909000 | 4.491731000 | 2.825563000 |
| 1 | 3.516709000 | 4.345833000 | 3.650821000 |
| 1 | 2.562474000 | 5.577817000 | 2.783504000 |
| 6 | 3.390689000 | 4.042426000 | 1.493679000 |
| 1 | 4.406856000 | 4.469721000 | 1.359157000 |
| 1 | 2.784849000 | 4.461941000 | 0.665089000 |
| 1 | 0.958053000 | 1.582902000 | 3.384913000 |
| 1 | 2.409756000 | 2.070126000 | 4.276539000 |
| 6 | -1.005760000 | -2.598511000 | 2.010256000 |
| 1 | -0.842317000 | -3.360172000 | 1.216165000 |

| | | | |
|---|---|---|---|
| 1 | -0.026124000 | -2.098652000 | 2.137707000 |
| 6 | -1.436272000 | -3.325746000 | 3.299876000 |
| 1 | -0.707733000 | -4.133381000 | 3.515556000 |
| 1 | -1.366992000 | -2.610382000 | 4.145082000 |
| 1 | -4.667824000 | -3.818260000 | 1.937401000 |
| 1 | -3.158064000 | -4.201188000 | 1.080362000 |
| 6 | -0.802489000 | -3.032420000 | -8.848211000 |
| 1 | -1.521226000 | -3.829725000 | -9.145465000 |
| 1 | 0.104328000 | -3.179636000 | -9.469464000 |
| 6 | -1.446797000 | -1.653559000 | -9.135111000 |
| 1 | -0.644668000 | -0.887520000 | -9.122659000 |
| 1 | -1.857027000 | -1.658488000 | -10.166099000 |
| 1 | -3.507401000 | -2.018753000 | -6.286988000 |
| 1 | -3.343675000 | -3.239125000 | -7.592806000 |
| 1 | 5.584326000 | 0.731309000 | -7.574859000 |
| 1 | 4.977037000 | -0.750941000 | -6.827930000 |
| 6 | 1.659060000 | 0.360436000 | -7.122764000 |
| 1 | 1.171274000 | 1.112428000 | -7.784365000 |
| 1 | 0.826524000 | -0.127114000 | -6.573924000 |
| 6 | 2.422173000 | -0.655848000 | -7.976210000 |
| 1 | 2.639034000 | -1.556920000 | -7.363082000 |
| 1 | 1.785801000 | -0.998804000 | -8.818234000 |
| 6 | -4.986598000 | -4.462630000 | -2.635322000 |
| 6 | -4.909227000 | -3.086976000 | -2.458376000 |
| 6 | -3.715291000 | -2.459964000 | -1.990473000 |
| 6 | -3.838061000 | -5.250674000 | -2.337256000 |
| 6 | -2.640797000 | -4.587030000 | -1.870004000 |
| 6 | -2.570002000 | -3.192580000 | -1.691757000 |
| 6 | -3.537458000 | -6.633477000 | -2.395330000 |
| 6 | -2.183879000 | -6.794209000 | -1.979076000 |
| 7 | -1.676877000 | -5.537879000 | -1.663977000 |
| 6 | -1.397407000 | -7.985329000 | -1.931458000 |
| 6 | -2.137616000 | -9.296950000 | -1.969799000 |
| 1 | -2.966332000 | -9.302110000 | -2.700788000 |
| 1 | -2.573784000 | -9.485743000 | -0.964832000 |
| 1 | -1.461400000 | -10.139542000 | -2.204003000 |
| 6 | -0.153551000 | -7.993537000 | -1.077822000 |
| 1 | 0.495270000 | -8.855900000 | -1.318210000 |
| 1 | -0.463823000 | -8.105207000 | -0.014887000 |
| 1 | 0.433152000 | -7.061320000 | -1.138999000 |
| 1 | -0.752560000 | -5.319833000 | -1.229037000 |
| 6 | -1.010102000 | -8.879676000 | -4.576987000 |
| 6 | -2.298034000 | -8.662482000 | -5.198489000 |
| 6 | -0.359089000 | -7.804550000 | -3.957659000 |
| 6 | -0.587804000 | -6.411621000 | -4.491988000 |
| 6 | -2.760937000 | -7.395041000 | -5.410858000 |
| 6 | -1.937810000 | -6.169926000 | -5.202673000 |

| | | | |
|---|---|---|---|
| 1 | 0.640311000 | -7.983097000 | -3.535051000 |
| 8 | -0.569861000 | -10.142338000 | -4.527786000 |
| 1 | -3.767597000 | -7.259999000 | -5.841550000 |
| 1 | -2.542260000 | -5.404413000 | -4.670882000 |
| 14 | 1.085424000 | -10.697864000 | -4.647003000 |
| 6 | 0.860003000 | -12.442538000 | -5.290419000 |
| 6 | 1.983937000 | -9.579070000 | -5.861064000 |
| 1 | 1.397036000 | -9.441593000 | -6.791589000 |
| 1 | 2.955164000 | -10.040666000 | -6.136077000 |
| 1 | 2.200921000 | -8.583098000 | -5.426821000 |
| 1 | 0.251533000 | -13.047296000 | -4.588046000 |
| 1 | 1.841838000 | -12.944114000 | -5.411394000 |
| 1 | 0.348944000 | -12.440660000 | -6.273896000 |
| 1 | -5.774032000 | -2.451050000 | -2.697820000 |
| 1 | -3.683644000 | -1.369221000 | -1.893625000 |
| 1 | -5.908037000 | -4.938865000 | -3.003222000 |
| 1 | -1.645083000 | -2.697618000 | -1.365969000 |
| 1 | -4.211832000 | -7.443732000 | -2.688938000 |
| 6 | 1.907767000 | -10.689137000 | -2.956348000 |
| 1 | 1.245905000 | -11.110769000 | -2.173367000 |
| 1 | 2.823180000 | -11.315987000 | -2.994160000 |
| 1 | 2.220617000 | -9.669907000 | -2.656236000 |
| 8 | -1.183252000 | 0.522625000 | -5.206643000 |
| 8 | -3.275794000 | -0.564833000 | -4.271902000 |
| 1 | -2.888516000 | -9.551201000 | -5.467810000 |
| 1 | -0.444014000 | -5.666022000 | -3.693002000 |
| 1 | 0.247115000 | -6.207673000 | -5.196919000 |
| 1 | -1.765706000 | -5.699610000 | -6.196999000 |

***TS-B6***

***Imaginary frequency = - 160.22***

| | | | |
|---|---|---|---|
| 6 | 2.714217000 | 2.427019000 | 2.059268000 |
| 6 | -3.780194000 | -0.248908000 | 1.494753000 |
| 6 | 3.091260000 | 1.062245000 | 2.080280000 |
| 6 | 3.235667000 | 3.258176000 | 1.048299000 |
| 6 | -2.487884000 | 0.295503000 | 1.302106000 |
| 6 | -3.955736000 | -1.538799000 | 2.008100000 |
| 1 | -4.661621000 | 0.333988000 | 1.192601000 |
| 6 | 3.927521000 | 0.560313000 | 1.066410000 |
| 6 | 4.064165000 | 2.751360000 | 0.045627000 |
| 1 | 2.961946000 | 4.323786000 | 1.038050000 |
| 6 | -2.353233000 | 1.679489000 | 0.777363000 |
| 6 | -1.379053000 | -0.517909000 | 1.635281000 |
| 6 | -2.848198000 | -2.342399000 | 2.323204000 |

| | | | |
|---|---|---|---|
| 1 | -4.973200000 | -1.943558000 | 2.131773000 |
| 6 | 4.408658000 | 1.380703000 | 0.025731000 |
| 1 | 4.191599000 | -0.505322000 | 1.090101000 |
| 1 | 4.407489000 | 3.414584000 | -0.761576000 |
| 6 | -1.249174000 | 2.112960000 | -0.032143000 |
| 6 | -3.341385000 | 2.628446000 | 1.035051000 |
| 6 | -1.548085000 | -1.819420000 | 2.131043000 |
| 1 | -0.359249000 | -0.137839000 | 1.498305000 |
| 6 | 5.213453000 | 0.855386000 | -1.104294000 |
| 8 | -0.232873000 | 1.205074000 | -0.292306000 |
| 6 | -1.116797000 | 3.418907000 | -0.520320000 |
| 1 | -4.183010000 | 2.358779000 | 1.689552000 |
| 6 | -3.333439000 | 3.926424000 | 0.460196000 |
| 6 | 4.873126000 | -0.370896000 | -1.758155000 |
| 6 | 6.280192000 | 1.569411000 | -1.643160000 |
| 15 | 0.081236000 | 0.642389000 | -1.816458000 |
| 6 | -2.220188000 | 4.330333000 | -0.357659000 |
| 6 | 0.148406000 | 3.871819000 | -1.162023000 |
| 6 | -4.420919000 | 4.830261000 | 0.648565000 |
| 8 | 3.863900000 | -1.139544000 | -1.200560000 |
| 6 | 5.495936000 | -0.853543000 | -2.910747000 |
| 1 | 6.597750000 | 2.503636000 | -1.155535000 |
| 6 | 6.959177000 | 1.149422000 | -2.819008000 |
| 8 | 0.023368000 | 2.048018000 | -2.688782000 |
| 7 | 1.581996000 | 0.136283000 | -1.695842000 |
| 7 | -0.957620000 | -0.368266000 | -2.499997000 |
| 6 | -2.278088000 | 5.596861000 | -1.008324000 |
| 6 | 0.848714000 | 5.024095000 | -0.653188000 |
| 6 | 0.704972000 | 3.170152000 | -2.235063000 |
| 1 | -5.260437000 | 4.514660000 | 1.287617000 |
| 6 | -4.438087000 | 6.065583000 | 0.019896000 |
| 15 | 2.371705000 | -1.209266000 | -1.881265000 |
| 6 | 5.019445000 | -2.117555000 | -3.546617000 |
| 6 | 6.552306000 | -0.060728000 | -3.493153000 |
| 6 | 8.007274000 | 1.939755000 | -3.378434000 |
| 16 | -2.525985000 | -0.106668000 | -2.857037000 |
| 6 | -3.364219000 | 6.441572000 | -0.829372000 |
| 1 | -1.457836000 | 5.891869000 | -1.676997000 |
| 6 | 0.469789000 | 5.702912000 | 0.542493000 |
| 6 | 2.016269000 | 5.484948000 | -1.359473000 |
| 6 | 1.913395000 | 3.557029000 | -2.906029000 |
| 1 | -5.289908000 | 6.747684000 | 0.162385000 |
| 8 | 2.754532000 | -1.313541000 | -3.490675000 |
| 7 | 1.740814000 | -2.611029000 | -1.359672000 |
| 6 | 3.665923000 | -2.284063000 | -3.881072000 |
| 6 | 5.935115000 | -3.182301000 | -3.868941000 |
| 6 | 7.188138000 | -0.387289000 | -4.726790000 |

| | | | |
|---|---|---|---|
| 1 | 8.310972000 | 2.855182000 | -2.847083000 |
| 6 | 8.615990000 | 1.582709000 | -4.571723000 |
| 8 | -3.011234000 | -1.244972000 | -3.669350000 |
| 8 | -3.335096000 | 0.293610000 | -1.682103000 |
| 1 | -3.396012000 | 7.406778000 | -1.356898000 |
| 1 | -0.388181000 | 5.332700000 | 1.118737000 |
| 6 | 1.183506000 | 6.803014000 | 0.997454000 |
| 6 | 2.710963000 | 6.636961000 | -0.882142000 |
| 6 | 2.494359000 | 4.751451000 | -2.475978000 |
| 6 | 2.578253000 | 2.752564000 | -3.965637000 |
| 16 | 2.331378000 | -3.443589000 | -0.124043000 |
| 6 | 3.181652000 | -3.371030000 | -4.686774000 |
| 6 | 5.470415000 | -4.289071000 | -4.667304000 |
| 6 | 7.280422000 | -3.206124000 | -3.393131000 |
| 6 | 8.191315000 | 0.413648000 | -5.255133000 |
| 1 | 6.867802000 | -1.283169000 | -5.273687000 |
| 1 | 9.416467000 | 2.207881000 | -4.995161000 |
| 1 | 0.879440000 | 7.300256000 | 1.930859000 |
| 6 | 2.304976000 | 7.286991000 | 0.272673000 |
| 1 | 3.595038000 | 6.984069000 | -1.439569000 |
| 1 | 3.383151000 | 5.131922000 | -2.999924000 |
| 6 | 1.896466000 | 1.913212000 | -4.872119000 |
| 6 | 3.987065000 | 2.834592000 | -4.098310000 |
| 8 | 2.689883000 | -2.658397000 | 1.074192000 |
| 8 | 1.422009000 | -4.612806000 | 0.108327000 |
| 6 | 4.110388000 | -4.334146000 | -5.077455000 |
| 6 | 1.786210000 | -3.390399000 | -5.201331000 |
| 6 | 6.386249000 | -5.328464000 | -5.011763000 |
| 6 | 8.141011000 | -4.239622000 | -3.732788000 |
| 1 | 7.626040000 | -2.396156000 | -2.736275000 |
| 1 | 8.654920000 | 0.141056000 | -6.215178000 |
| 1 | 2.857997000 | 8.165420000 | 0.638037000 |
| 6 | 2.563398000 | 1.189019000 | -5.879473000 |
| 1 | 0.807073000 | 1.814500000 | -4.801725000 |
| 6 | 4.653815000 | 2.156046000 | -5.116832000 |
| 1 | 4.573252000 | 3.425212000 | -3.380434000 |
| 1 | 3.774199000 | -5.179281000 | -5.697201000 |
| 6 | 1.561730000 | -3.655058000 | -6.573239000 |
| 6 | 0.669588000 | -3.082157000 | -4.393107000 |
| 1 | 6.021436000 | -6.164731000 | -5.627624000 |
| 6 | 7.697428000 | -5.301357000 | -4.565631000 |
| 1 | 9.168947000 | -4.243907000 | -3.341055000 |
| 6 | 3.962673000 | 1.332765000 | -6.027326000 |
| 1 | 5.746628000 | 2.254451000 | -5.207101000 |
| 6 | 0.281831000 | -3.543128000 | -7.138100000 |
| 1 | 2.417238000 | -3.885048000 | -7.228376000 |
| 6 | -0.607755000 | -2.957965000 | -4.949177000 |

| | | | |
|---|---|---|---|
| 1 | 0.792351000 | -2.940264000 | -3.313578000 |
| 1 | 8.389019000 | -6.114824000 | -4.830752000 |
| 6 | -0.812039000 | -3.160319000 | -6.323802000 |
| 1 | -1.448830000 | -2.648740000 | -4.310570000 |
| 6 | -1.730868000 | 2.270130000 | -6.112402000 |
| 6 | -2.212876000 | 3.534372000 | -5.732898000 |
| 6 | -1.867006000 | 1.187403000 | -5.234398000 |
| 6 | -2.851895000 | 3.693239000 | -4.493218000 |
| 6 | -2.497861000 | 1.329751000 | -3.986923000 |
| 6 | -3.009147000 | 2.591239000 | -3.632188000 |
| 6 | 3.776720000 | -5.250869000 | -1.688970000 |
| 6 | 3.871441000 | -4.238668000 | -0.718676000 |
| 6 | 4.875968000 | -6.036682000 | -2.052503000 |
| 6 | 5.141545000 | -3.951923000 | -0.184418000 |
| 6 | 6.127875000 | -5.772453000 | -1.475912000 |
| 6 | 6.264533000 | -4.707761000 | -0.572266000 |
| 9 | 5.347802000 | -2.973228000 | 0.692939000 |
| 9 | 7.469570000 | -4.418915000 | -0.075589000 |
| 9 | 7.187906000 | -6.511642000 | -1.798895000 |
| 9 | 4.731707000 | -7.021916000 | -2.947567000 |
| 9 | 2.608381000 | -5.520128000 | -2.292706000 |
| 9 | -2.075479000 | 4.575270000 | -6.553740000 |
| 9 | -3.638423000 | 2.811165000 | -2.481969000 |
| 9 | -1.338778000 | 0.016490000 | -5.608018000 |
| 9 | -3.314045000 | 4.894963000 | -4.135874000 |
| 9 | -1.130886000 | 2.111654000 | -7.299494000 |
| 6 | -2.939424000 | -3.762922000 | 2.809680000 |
| 6 | -2.013311000 | -4.015359000 | 4.021604000 |
| 1 | -2.501972000 | -3.601716000 | 4.927490000 |
| 1 | -1.931927000 | -5.109444000 | 4.188387000 |
| 6 | 4.718508000 | 0.613359000 | -7.123883000 |
| 6 | 3.811447000 | 0.175379000 | -8.274991000 |
| 1 | 4.380781000 | -0.445656000 | -8.997544000 |
| 1 | 3.450286000 | 1.067578000 | -8.833893000 |
| 6 | -2.130670000 | -2.918246000 | -7.011236000 |
| 6 | -1.923939000 | -2.020262000 | -8.251527000 |
| 1 | -2.876744000 | -1.943769000 | -8.814216000 |
| 1 | -1.691459000 | -0.999039000 | -7.889719000 |
| 6 | 1.728639000 | 2.983731000 | 3.065648000 |
| 6 | 1.627187000 | 2.142707000 | 4.341967000 |
| 1 | 2.555875000 | 2.263872000 | 4.943707000 |
| 1 | 0.790401000 | 2.507739000 | 4.972876000 |
| 6 | 1.439223000 | 0.664485000 | 3.987832000 |
| 1 | 0.510171000 | 0.553664000 | 3.388190000 |
| 1 | 1.291377000 | 0.051369000 | 4.901308000 |
| 6 | 2.637309000 | 0.135266000 | 3.192801000 |
| 1 | 2.428931000 | -0.867950000 | 2.767428000 |

| | | | |
|---|---|---|---|
| 1 | 3.496989000 | -0.012281000 | 3.886722000 |
| 1 | 1.983400000 | 4.039827000 | 3.294894000 |
| 1 | 0.727383000 | 3.022412000 | 2.575286000 |
| 6 | -0.398041000 | -2.727767000 | 2.473586000 |
| 1 | -0.320336000 | -3.528788000 | 1.704891000 |
| 1 | 0.567912000 | -2.190705000 | 2.427581000 |
| 6 | -0.604096000 | -3.380056000 | 3.854599000 |
| 1 | 0.189281000 | -4.137118000 | 4.019919000 |
| 1 | -0.454184000 | -2.599772000 | 4.629311000 |
| 1 | -3.987076000 | -4.037154000 | 3.048170000 |
| 1 | -2.627569000 | -4.430528000 | 1.972907000 |
| 6 | -0.007376000 | -3.726563000 | -8.605916000 |
| 1 | -0.630450000 | -4.640477000 | -8.738457000 |
| 1 | 0.926452000 | -3.900821000 | -9.178137000 |
| 6 | -0.781276000 | -2.516057000 | -9.180954000 |
| 1 | -0.060424000 | -1.689855000 | -9.348687000 |
| 1 | -1.177458000 | -2.787294000 | -10.181122000 |
| 1 | -2.849870000 | -2.456417000 | -6.306550000 |
| 1 | -2.571864000 | -3.889855000 | -7.335791000 |
| 1 | 5.553540000 | 1.253626000 | -7.478404000 |
| 1 | 5.204948000 | -0.288499000 | -6.686970000 |
| 6 | 1.760163000 | 0.288745000 | -6.800146000 |
| 1 | 1.092988000 | 0.926640000 | -7.420798000 |
| 1 | 1.080753000 | -0.339940000 | -6.188430000 |
| 6 | 2.622053000 | -0.597667000 | -7.704513000 |
| 1 | 3.009263000 | -1.455074000 | -7.113910000 |
| 1 | 1.995418000 | -1.035893000 | -8.508267000 |
| 6 | -4.515502000 | -5.173787000 | -1.238386000 |
| 6 | -4.660625000 | -3.795351000 | -1.162186000 |
| 6 | -3.535470000 | -2.934683000 | -0.988579000 |
| 6 | -3.207387000 | -5.721906000 | -1.124532000 |
| 6 | -2.083722000 | -4.830645000 | -0.933958000 |
| 6 | -2.239104000 | -3.431701000 | -0.874642000 |
| 6 | -2.680282000 | -7.032605000 | -1.170514000 |
| 6 | -1.265557000 | -6.934712000 | -0.982736000 |
| 7 | -0.944897000 | -5.588487000 | -0.838634000 |
| 6 | -0.304117000 | -7.979067000 | -0.982270000 |
| 6 | -0.794751000 | -9.394804000 | -0.875533000 |
| 1 | -1.739453000 | -9.570463000 | -1.421774000 |
| 1 | -0.976845000 | -9.629254000 | 0.196668000 |
| 1 | -0.035112000 | -10.107290000 | -1.246726000 |
| 6 | 1.072289000 | -7.735197000 | -0.429692000 |
| 1 | 1.777313000 | -8.528291000 | -0.744298000 |
| 1 | 1.014457000 | -7.766665000 | 0.682066000 |
| 1 | 1.484676000 | -6.746096000 | -0.689854000 |
| 1 | -0.011157000 | -5.193485000 | -0.581527000 |
| 6 | -0.398343000 | -8.830348000 | -3.726617000 |

| | | | |
|---|---|---|---|
| 6 | -1.782678000 | -8.589230000 | -4.076343000 |
| 6 | 0.389872000 | -7.765389000 | -3.295226000 |
| 6 | 0.054205000 | -6.353453000 | -3.689477000 |
| 6 | -2.273958000 | -7.315183000 | -4.134396000 |
| 6 | -1.420896000 | -6.096558000 | -4.073218000 |
| 1 | 1.441312000 | -7.979216000 | -3.052794000 |
| 1 | -3.350775000 | -7.165922000 | -4.319083000 |
| 1 | -1.896782000 | -5.353124000 | -3.395784000 |
| 1 | -5.659499000 | -3.343109000 | -1.250120000 |
| 1 | -3.693251000 | -1.848544000 | -0.955650000 |
| 1 | -5.384292000 | -5.833315000 | -1.384570000 |
| 1 | -1.376935000 | -2.759129000 | -0.767711000 |
| 1 | -3.243575000 | -7.964364000 | -1.276792000 |
| 1 | 0.372228000 | -5.650546000 | -2.898793000 |
| 1 | 0.715689000 | -6.096910000 | -4.546214000 |
| 1 | -1.476935000 | -5.591912000 | -5.063098000 |
| 1 | -2.455801000 | -9.448327000 | -4.213242000 |
| 8 | 0.107658000 | -10.072631000 | -3.684231000 |
| 14 | -0.443748000 | -11.583432000 | -4.335365000 |
| 6 | -1.921625000 | -12.205031000 | -3.346526000 |
| 1 | -1.678423000 | -12.258618000 | -2.266065000 |
| 1 | -2.823122000 | -11.571803000 | -3.465406000 |
| 1 | -2.187730000 | -13.229178000 | -3.682040000 |
| 6 | -0.843376000 | -11.335847000 | -6.154660000 |
| 1 | 0.036777000 | -10.930266000 | -6.693556000 |
| 1 | -1.110509000 | -12.306466000 | -6.621542000 |
| 1 | -1.690897000 | -10.639597000 | -6.311191000 |
| 6 | 1.044491000 | -12.691675000 | -4.082027000 |
| 1 | 0.842130000 | -13.713276000 | -4.463670000 |
| 1 | 1.929758000 | -12.292783000 | -4.616508000 |
| 1 | 1.300445000 | -12.772967000 | -3.006372000 |

***TS-B7***

***Imaginary frequency = - 157.71***

| | | | |
|---|---|---|---|
| 6 | 2.666483000 | 1.884384000 | 2.445107000 |
| 6 | -3.793290000 | -0.393140000 | 0.946903000 |
| 6 | 3.236879000 | 2.879847000 | 1.616621000 |
| 6 | 3.039304000 | 0.540994000 | 2.239072000 |
| 6 | -2.536069000 | 0.258059000 | 0.928310000 |
| 6 | -3.912659000 | -1.723593000 | 1.364955000 |
| 1 | -4.681797000 | 0.135400000 | 0.573307000 |
| 6 | 4.073813000 | 2.487897000 | 0.555043000 |
| 6 | 3.905338000 | 0.164741000 | 1.212290000 |
| 1 | 2.622952000 | -0.240354000 | 2.895647000 |

| | | | |
|---|---|---|---|
| 6 | -2.455786000 | 1.661703000 | 0.446085000 |
| 6 | -1.405855000 | -0.477761000 | 1.360821000 |
| 6 | -2.779020000 | -2.459290000 | 1.745820000 |
| 1 | -4.898856000 | -2.214546000 | 1.348667000 |
| 6 | 4.407831000 | 1.140065000 | 0.317510000 |
| 1 | 4.437525000 | 3.263874000 | -0.138156000 |
| 1 | 4.157476000 | -0.894740000 | 1.092011000 |
| 6 | -1.305132000 | 2.190140000 | -0.231872000 |
| 6 | -3.537085000 | 2.530631000 | 0.603207000 |
| 6 | -1.515140000 | -1.821884000 | 1.743706000 |
| 1 | -0.415141000 | -0.005710000 | 1.380555000 |
| 6 | 5.210563000 | 0.783192000 | -0.878786000 |
| 8 | -0.189892000 | 1.376735000 | -0.363858000 |
| 6 | -1.235510000 | 3.494225000 | -0.741136000 |
| 1 | -4.418527000 | 2.192846000 | 1.167215000 |
| 6 | -3.563090000 | 3.832784000 | 0.039019000 |
| 6 | 4.884436000 | -0.364805000 | -1.668593000 |
| 6 | 6.260649000 | 1.570815000 | -1.341924000 |
| 15 | 0.160192000 | 0.755757000 | -1.855564000 |
| 6 | -2.417669000 | 4.315183000 | -0.689109000 |
| 6 | 0.039906000 | 4.022201000 | -1.304003000 |
| 6 | -4.720492000 | 4.662275000 | 0.142849000 |
| 8 | 3.882845000 | -1.197741000 | -1.200273000 |
| 6 | 5.514689000 | -0.719937000 | -2.862318000 |
| 1 | 6.569879000 | 2.450330000 | -0.756998000 |
| 6 | 6.946645000 | 1.284840000 | -2.554287000 |
| 8 | 0.176692000 | 2.135957000 | -2.759095000 |
| 7 | 1.609171000 | 0.148877000 | -1.621957000 |
| 7 | -1.024326000 | -0.153684000 | -2.441722000 |
| 6 | -2.516755000 | 5.572864000 | -1.355363000 |
| 6 | 0.612024000 | 5.251047000 | -0.811373000 |
| 6 | 0.725990000 | 3.319156000 | -2.300211000 |
| 1 | -5.586340000 | 4.284824000 | 0.707994000 |
| 6 | -4.767705000 | 5.899062000 | -0.478380000 |
| 15 | 2.385907000 | -1.197741000 | -1.868467000 |
| 6 | 5.059798000 | -1.925232000 | -3.618365000 |
| 6 | 6.567533000 | 0.140196000 | -3.349471000 |
| 6 | 7.989629000 | 2.140673000 | -3.019156000 |
| 16 | -1.797498000 | 0.091132000 | -3.850240000 |
| 6 | -3.664122000 | 6.345510000 | -1.253232000 |
| 1 | -1.674869000 | 5.918587000 | -1.970272000 |
| 6 | 0.073433000 | 5.977857000 | 0.292825000 |
| 6 | 1.814621000 | 5.749240000 | -1.432710000 |
| 6 | 1.941354000 | 3.772850000 | -2.909885000 |
| 1 | -5.671416000 | 6.521898000 | -0.402877000 |
| 8 | 2.759719000 | -1.211560000 | -3.482869000 |
| 7 | 1.745797000 | -2.605122000 | -1.378850000 |

| | | | |
|---|---|---|---|
| 6 | 3.708010000 | -2.105058000 | -3.956906000 |
| 6 | 6.001709000 | -2.928606000 | -4.050335000 |
| 6 | 7.232008000 | -0.059845000 | -4.594641000 |
| 1 | 8.270183000 | 3.005677000 | -2.398169000 |
| 6 | 8.627038000 | 1.905024000 | -4.227483000 |
| 8 | -0.891897000 | 0.393364000 | -4.983930000 |
| 8 | -2.790438000 | -0.993517000 | -4.031046000 |
| 1 | -3.724685000 | 7.303732000 | -1.790198000 |
| 1 | -0.809438000 | 5.585676000 | 0.813709000 |
| 6 | 0.660261000 | 7.156836000 | 0.730776000 |
| 6 | 2.384272000 | 6.972347000 | -0.966825000 |
| 6 | 2.432551000 | 5.001832000 | -2.469881000 |
| 6 | 2.664022000 | 2.995976000 | -3.948389000 |
| 16 | 2.402024000 | -3.581302000 | -0.281109000 |
| 6 | 3.253624000 | -3.125451000 | -4.860586000 |
| 6 | 5.569759000 | -3.952281000 | -4.969113000 |
| 6 | 7.343316000 | -2.976427000 | -3.566324000 |
| 6 | 8.234869000 | 0.798238000 | -5.024196000 |
| 1 | 6.934721000 | -0.902261000 | -5.231271000 |
| 1 | 9.425255000 | 2.578327000 | -4.574419000 |
| 1 | 0.227497000 | 7.692557000 | 1.589060000 |
| 6 | 1.818983000 | 7.668575000 | 0.089619000 |
| 1 | 3.297140000 | 7.343870000 | -1.458005000 |
| 1 | 3.335402000 | 5.416094000 | -2.942540000 |
| 6 | 2.003714000 | 2.206453000 | -4.913143000 |
| 6 | 4.076401000 | 3.072086000 | -4.013763000 |
| 8 | 2.826566000 | -2.939247000 | 0.977396000 |
| 8 | 1.500402000 | -4.769291000 | -0.134973000 |
| 6 | 4.208329000 | -3.998609000 | -5.375145000 |
| 6 | 1.837886000 | -3.176823000 | -5.311916000 |
| 6 | 6.513228000 | -4.922475000 | -5.422227000 |
| 6 | 8.231111000 | -3.945310000 | -4.010930000 |
| 1 | 7.663921000 | -2.240398000 | -2.816459000 |
| 1 | 8.720930000 | 0.621879000 | -5.995542000 |
| 1 | 2.273854000 | 8.606338000 | 0.442523000 |
| 6 | 2.706020000 | 1.515934000 | -5.918658000 |
| 1 | 0.910482000 | 2.119387000 | -4.903278000 |
| 6 | 4.777463000 | 2.419213000 | -5.027559000 |
| 1 | 4.632926000 | 3.635800000 | -3.250402000 |
| 1 | 3.891117000 | -4.782223000 | -6.079946000 |
| 6 | 1.548922000 | -3.232218000 | -6.694704000 |
| 6 | 0.758413000 | -3.126996000 | -4.404798000 |
| 1 | 6.172033000 | -5.693701000 | -6.129797000 |
| 6 | 7.821134000 | -4.913651000 | -4.965315000 |
| 1 | 9.255050000 | -3.970924000 | -3.609665000 |
| 6 | 4.113330000 | 1.639427000 | -5.996634000 |
| 1 | 5.874105000 | 2.505233000 | -5.068566000 |

| | | | |
|---|---|---|---|
| 6 | 0.228134000 | -3.157206000 | -7.163889000 |
| 1 | 2.376129000 | -3.259540000 | -7.421094000 |
| 6 | -0.560962000 | -3.077369000 | -4.865052000 |
| 1 | 0.949467000 | -3.130399000 | -3.324946000 |
| 1 | 8.534408000 | -5.675040000 | -5.314392000 |
| 6 | -0.841305000 | -3.064063000 | -6.239818000 |
| 1 | -1.383844000 | -2.991643000 | -4.142351000 |
| 6 | -3.501484000 | 3.878664000 | -4.072000000 |
| 6 | -4.657835000 | 3.723832000 | -3.293983000 |
| 6 | -2.600623000 | 2.808687000 | -4.223889000 |
| 6 | -4.874671000 | 2.522217000 | -2.602689000 |
| 6 | -2.830351000 | 1.581488000 | -3.574668000 |
| 6 | -3.967465000 | 1.464607000 | -2.752747000 |
| 6 | 3.734016000 | -5.209631000 | -2.109184000 |
| 6 | 3.900200000 | -4.306094000 | -1.046746000 |
| 6 | 4.797844000 | -5.968489000 | -2.608023000 |
| 6 | 5.198159000 | -4.103777000 | -0.543381000 |
| 6 | 6.078488000 | -5.801576000 | -2.057514000 |
| 6 | 6.282906000 | -4.841410000 | -1.055224000 |
| 9 | 5.465605000 | -3.221853000 | 0.416014000 |
| 9 | 7.514787000 | -4.635180000 | -0.584512000 |
| 9 | 7.101366000 | -6.527807000 | -2.504153000 |
| 9 | 4.598320000 | -6.840843000 | -3.605528000 |
| 9 | 2.525396000 | -5.392106000 | -2.671025000 |
| 9 | -5.527173000 | 4.728633000 | -3.184972000 |
| 9 | -4.220080000 | 0.343408000 | -2.067286000 |
| 9 | -1.533536000 | 3.028196000 | -4.991649000 |
| 9 | -5.938278000 | 2.389224000 | -1.801189000 |
| 9 | -3.264346000 | 5.047739000 | -4.675462000 |
| 6 | -2.797298000 | -3.917481000 | 2.113465000 |
| 6 | -1.982194000 | -4.191651000 | 3.397468000 |
| 1 | -2.582428000 | -3.854592000 | 4.267440000 |
| 1 | -1.856201000 | -5.287619000 | 3.516348000 |
| 6 | 4.903808000 | 0.938887000 | -7.082074000 |
| 6 | 4.041622000 | 0.539304000 | -8.281591000 |
| 1 | 4.633243000 | -0.079468000 | -8.988243000 |
| 1 | 3.724118000 | 1.448035000 | -8.840368000 |
| 6 | -2.215248000 | -2.821629000 | -6.805544000 |
| 6 | -2.143121000 | -1.631281000 | -7.788372000 |
| 1 | -3.130419000 | -1.489205000 | -8.273547000 |
| 1 | -1.950390000 | -0.727387000 | -7.176580000 |
| 6 | 1.666820000 | 2.239200000 | 3.527562000 |
| 6 | 1.064516000 | 3.635402000 | 3.346725000 |
| 1 | 0.379465000 | 3.634868000 | 2.469295000 |
| 1 | 0.450148000 | 3.904963000 | 4.230499000 |
| 6 | 2.180261000 | 4.657861000 | 3.115153000 |
| 1 | 2.886266000 | 4.624624000 | 3.975188000 |

| | | | |
|---|---|---|---|
| 1 | 1.775147000 | 5.689660000 | 3.074923000 |
| 6 | 2.920561000 | 4.348982000 | 1.811038000 |
| 1 | 3.857402000 | 4.941172000 | 1.740392000 |
| 1 | 2.297787000 | 4.684758000 | 0.957161000 |
| 1 | 0.870651000 | 1.464669000 | 3.553543000 |
| 1 | 2.170889000 | 2.183255000 | 4.520237000 |
| 6 | -0.332812000 | -2.670898000 | 2.121999000 |
| 1 | -0.135272000 | -3.389379000 | 1.295978000 |
| 1 | 0.591812000 | -2.068493000 | 2.210077000 |
| 6 | -0.601026000 | -3.477071000 | 3.408163000 |
| 1 | 0.219593000 | -4.210122000 | 3.544394000 |
| 1 | -0.548425000 | -2.786162000 | 4.274631000 |
| 1 | -3.836602000 | -4.290202000 | 2.216553000 |
| 1 | -2.344630000 | -4.486663000 | 1.268330000 |
| 6 | -0.141041000 | -3.045003000 | -8.620158000 |
| 1 | -0.712605000 | -3.950601000 | -8.926101000 |
| 1 | 0.763308000 | -3.012730000 | -9.261076000 |
| 6 | -1.026936000 | -1.796315000 | -8.857150000 |
| 1 | -0.373274000 | -0.900076000 | -8.848389000 |
| 1 | -1.461692000 | -1.855436000 | -9.876405000 |
| 1 | -2.931057000 | -2.600447000 | -5.990841000 |
| 1 | -2.578817000 | -3.727923000 | -7.344683000 |
| 1 | 5.761282000 | 1.575336000 | -7.386934000 |
| 1 | 5.359559000 | 0.019062000 | -6.649657000 |
| 6 | 1.923918000 | 0.676962000 | -6.912394000 |
| 1 | 1.332666000 | 1.362606000 | -7.561571000 |
| 1 | 1.170132000 | 0.076260000 | -6.362302000 |
| 6 | 2.809300000 | -0.218102000 | -7.783120000 |
| 1 | 3.151122000 | -1.088040000 | -7.182108000 |
| 1 | 2.217411000 | -0.633967000 | -8.624984000 |
| 6 | -4.085190000 | -4.722141000 | -2.066044000 |
| 6 | -4.028067000 | -3.346810000 | -1.890407000 |
| 6 | -2.801216000 | -2.689364000 | -1.573911000 |
| 6 | -2.883616000 | -5.472418000 | -1.915613000 |
| 6 | -1.651830000 | -4.778668000 | -1.613402000 |
| 6 | -1.603106000 | -3.384176000 | -1.435350000 |
| 6 | -2.559178000 | -6.845356000 | -1.983606000 |
| 6 | -1.164179000 | -6.980873000 | -1.717341000 |
| 7 | -0.642399000 | -5.703522000 | -1.530976000 |
| 6 | -0.385721000 | -8.168415000 | -1.626114000 |
| 6 | -1.083674000 | -9.447496000 | -1.254880000 |
| 1 | -2.101075000 | -9.527670000 | -1.676119000 |
| 1 | -1.179967000 | -9.477763000 | -0.146483000 |
| 1 | -0.507358000 | -10.340662000 | -1.561991000 |
| 6 | 1.043613000 | -8.047975000 | -1.169546000 |
| 1 | 1.590904000 | -9.002630000 | -1.282156000 |
| 1 | 1.056637000 | -7.775693000 | -0.090218000 |

| | | | |
|---|---|---|---|
| 1 | 1.594643000 | -7.247876000 | -1.693511000 |
| 1 | 0.288614000 | -5.447034000 | -1.134601000 |
| 6 | -1.090159000 | -9.002251000 | -4.364877000 |
| 6 | -1.793334000 | -7.918197000 | -5.028928000 |
| 6 | 0.200600000 | -8.801361000 | -3.879493000 |
| 6 | 1.021756000 | -7.680165000 | -4.464498000 |
| 6 | -1.202641000 | -6.698764000 | -5.179575000 |
| 1 | -2.837123000 | -8.068213000 | -5.339563000 |
| 6 | 0.200817000 | -6.418521000 | -4.768219000 |
| 1 | 0.723937000 | -9.685526000 | -3.485392000 |
| 8 | -1.702561000 | -10.167562000 | -4.117363000 |
| 1 | -1.783388000 | -5.855312000 | -5.588738000 |
| 1 | 0.149402000 | -5.738548000 | -3.889506000 |
| 14 | -3.253812000 | -10.820308000 | -4.545925000 |
| 6 | -4.618583000 | -9.820559000 | -3.718408000 |
| 6 | -3.405184000 | -10.842756000 | -6.418702000 |
| 1 | -3.451089000 | -9.827023000 | -6.858499000 |
| 1 | -4.328720000 | -11.381830000 | -6.715581000 |
| 1 | -2.543014000 | -11.373534000 | -6.870990000 |
| 1 | -4.488754000 | -9.808349000 | -2.616899000 |
| 1 | -5.601759000 | -10.290328000 | -3.930197000 |
| 1 | -4.665515000 | -8.772224000 | -4.073508000 |
| 1 | -4.932357000 | -2.732314000 | -2.007740000 |
| 1 | -2.788205000 | -1.600044000 | -1.459438000 |
| 1 | -5.030559000 | -5.228862000 | -2.312844000 |
| 1 | -0.658371000 | -2.861964000 | -1.231820000 |
| 1 | -3.244418000 | -7.668812000 | -2.208436000 |
| 6 | -3.158696000 | -12.548982000 | -3.831686000 |
| 1 | -3.000550000 | -12.516132000 | -2.734885000 |
| 1 | -4.098129000 | -13.105701000 | -4.026015000 |
| 1 | -2.321671000 | -13.118472000 | -4.282985000 |
| 1 | 0.694195000 | -5.788780000 | -5.538130000 |
| 1 | 1.886392000 | -7.428948000 | -3.822120000 |
| 1 | 1.472541000 | -8.064362000 | -5.410502000 |

**Ionization TS**

***TS-C1***

***Imaginary frequency = - 103.1***

| | | | |
|---|---|---|---|
| 6 | 1.742865000 | 1.750084000 | 1.937178000 |
| 6 | -1.787985000 | -0.903267000 | 0.038184000 |
| 6 | 2.166422000 | 0.403063000 | 1.831805000 |
| 6 | 2.361027000 | 2.721226000 | 1.124201000 |
| 6 | -2.388926000 | 0.231882000 | 0.627294000 |
| 6 | -1.866013000 | -2.159438000 | 0.653877000 |

| | | | |
|---|---|---|---|
| 1 | -1.282319000 | -0.828607000 | -0.928688000 |
| 6 | 3.147150000 | 0.063808000 | 0.883777000 |
| 6 | 3.339476000 | 2.375816000 | 0.189182000 |
| 1 | 2.046346000 | 3.771923000 | 1.214064000 |
| 6 | -2.388528000 | 1.589522000 | 0.020028000 |
| 6 | -3.048975000 | 0.057055000 | 1.870650000 |
| 6 | -2.523192000 | -2.319167000 | 1.881118000 |
| 1 | -1.395970000 | -3.026157000 | 0.166922000 |
| 6 | 3.736422000 | 1.027788000 | 0.042190000 |
| 1 | 3.447017000 | -0.988861000 | 0.799829000 |
| 1 | 3.766882000 | 3.149710000 | -0.465552000 |
| 6 | -1.263258000 | 2.150189000 | -0.672479000 |
| 6 | -3.489076000 | 2.431802000 | 0.204129000 |
| 6 | -3.119999000 | -1.192141000 | 2.496995000 |
| 1 | -3.484866000 | 0.930566000 | 2.380227000 |
| 6 | 4.730409000 | 0.655277000 | -0.991737000 |
| 8 | -0.173372000 | 1.329781000 | -0.950355000 |
| 6 | -1.181424000 | 3.506028000 | -1.020774000 |
| 1 | -4.384688000 | 2.034606000 | 0.704375000 |
| 6 | -3.515325000 | 3.779068000 | -0.242384000 |
| 6 | 4.537133000 | -0.488545000 | -1.828411000 |
| 6 | 5.865685000 | 1.423114000 | -1.241478000 |
| 15 | 0.117564000 | 0.982725000 | -2.540008000 |
| 6 | -2.345596000 | 4.335635000 | -0.870530000 |
| 6 | 0.113888000 | 4.077486000 | -1.483460000 |
| 6 | -4.672809000 | 4.599038000 | -0.081794000 |
| 8 | 3.414657000 | -1.280100000 | -1.619652000 |
| 6 | 5.397288000 | -0.859625000 | -2.860455000 |
| 1 | 6.065615000 | 2.299939000 | -0.606632000 |
| 6 | 6.766728000 | 1.127408000 | -2.301982000 |
| 8 | 0.128468000 | 2.472059000 | -3.238174000 |
| 7 | 1.547208000 | 0.332395000 | -2.619475000 |
| 7 | -1.197163000 | 0.223692000 | -3.139250000 |
| 6 | -2.401158000 | 5.676645000 | -1.354553000 |
| 6 | 0.763189000 | 5.137081000 | -0.755628000 |
| 6 | 0.767709000 | 3.520481000 | -2.581088000 |
| 1 | -5.561144000 | 4.164753000 | 0.401658000 |
| 6 | -4.685665000 | 5.907784000 | -0.535440000 |
| 15 | 2.148269000 | -1.139357000 | -2.663008000 |
| 6 | 5.091286000 | -2.072779000 | -3.669871000 |
| 6 | 6.532978000 | -0.022152000 | -3.142882000 |
| 6 | 7.880057000 | 1.974377000 | -2.583513000 |
| 16 | -1.888758000 | 0.625376000 | -4.530278000 |
| 6 | -3.544521000 | 6.444707000 | -1.190224000 |
| 1 | -1.526123000 | 6.092160000 | -1.873459000 |
| 6 | 0.238094000 | 5.691495000 | 0.449358000 |
| 6 | 2.037749000 | 5.616023000 | -1.232000000 |

| | | | |
|---|---|---|---|
| 6 | 2.049179000 | 3.945091000 | -3.049331000 |
| 1 | -5.586652000 | 6.526704000 | -0.411674000 |
| 8 | 2.909403000 | -1.195127000 | -4.132411000 |
| 7 | 1.209816000 | -2.419849000 | -2.472683000 |
| 6 | 3.860360000 | -2.190367000 | -4.330620000 |
| 6 | 6.042409000 | -3.146925000 | -3.775010000 |
| 6 | 7.402364000 | -0.243322000 | -4.251265000 |
| 1 | 8.053015000 | 2.845337000 | -1.932233000 |
| 6 | 8.715346000 | 1.724702000 | -3.661792000 |
| 8 | -0.976636000 | 0.931569000 | -5.650256000 |
| 8 | -2.951983000 | -0.412112000 | -4.790736000 |
| 1 | -3.572596000 | 7.472154000 | -1.582317000 |
| 1 | -0.706397000 | 5.302362000 | 0.851736000 |
| 6 | 0.915725000 | 6.693328000 | 1.129962000 |
| 6 | 2.698665000 | 6.659684000 | -0.517506000 |
| 6 | 2.637241000 | 5.010672000 | -2.369908000 |
| 6 | 2.762489000 | 3.248676000 | -4.145985000 |
| 16 | 1.181793000 | -3.404603000 | -1.186900000 |
| 6 | 3.549092000 | -3.273447000 | -5.218691000 |
| 6 | 5.762759000 | -4.233020000 | -4.678518000 |
| 6 | 7.232351000 | -3.203440000 | -2.991097000 |
| 6 | 8.466168000 | 0.611082000 | -4.507027000 |
| 1 | 7.209656000 | -1.093331000 | -4.919725000 |
| 1 | 9.563714000 | 2.393561000 | -3.871047000 |
| 1 | 0.494908000 | 7.097615000 | 2.062883000 |
| 6 | 2.150607000 | 7.192830000 | 0.638271000 |
| 1 | 3.666871000 | 7.021847000 | -0.896826000 |
| 1 | 3.612562000 | 5.386336000 | -2.714702000 |
| 6 | 2.122116000 | 2.847305000 | -5.334429000 |
| 6 | 4.141320000 | 2.974230000 | -4.004105000 |
| 8 | 1.301009000 | -2.710501000 | 0.112124000 |
| 8 | 0.043597000 | -4.341507000 | -1.372043000 |
| 6 | 4.537545000 | -4.243974000 | -5.394009000 |
| 6 | 2.284307000 | -3.387911000 | -5.991635000 |
| 6 | 6.712017000 | -5.289451000 | -4.813731000 |
| 6 | 8.123678000 | -4.258314000 | -3.127193000 |
| 1 | 7.429494000 | -2.405442000 | -2.262135000 |
| 1 | 9.116879000 | 0.426595000 | -5.375076000 |
| 1 | 2.676585000 | 7.990548000 | 1.183942000 |
| 6 | 2.820299000 | 2.191961000 | -6.365188000 |
| 1 | 1.051513000 | 3.052665000 | -5.472331000 |
| 6 | 4.837395000 | 2.308735000 | -5.013955000 |
| 1 | 4.661532000 | 3.249575000 | -3.075347000 |
| 1 | 4.363187000 | -5.058892000 | -6.111042000 |
| 6 | 1.619937000 | -2.266844000 | -6.542292000 |
| 6 | 1.780754000 | -4.674470000 | -6.299047000 |
| 1 | 6.487217000 | -6.111341000 | -5.510545000 |

| | | | |
|---|---|---|---|
| 6 | 7.874351000 | -5.299526000 | -4.060008000 |
| 1 | 9.027932000 | -4.290801000 | -2.501243000 |
| 6 | 4.195251000 | 1.897508000 | -6.198195000 |
| 1 | 5.907541000 | 2.090385000 | -4.881624000 |
| 6 | 0.563422000 | -2.424198000 | -7.455101000 |
| 1 | 1.952988000 | -1.253201000 | -6.280406000 |
| 6 | 0.715743000 | -4.836385000 | -7.192199000 |
| 1 | 2.228153000 | -5.556706000 | -5.820815000 |
| 1 | 8.590926000 | -6.128317000 | -4.159786000 |
| 6 | 0.124456000 | -3.722843000 | -7.812482000 |
| 1 | 0.352637000 | -5.849415000 | -7.430831000 |
| 6 | -3.399643000 | 4.480836000 | -4.425553000 |
| 6 | -4.567214000 | 4.318229000 | -3.664696000 |
| 6 | -2.566985000 | 3.378386000 | -4.692097000 |
| 6 | -4.871775000 | 3.064485000 | -3.110423000 |
| 6 | -2.881260000 | 2.108328000 | -4.173011000 |
| 6 | -4.020299000 | 1.979753000 | -3.355012000 |
| 6 | 2.745217000 | -5.324659000 | -2.469803000 |
| 6 | 2.694636000 | -4.409170000 | -1.402403000 |
| 6 | 3.883200000 | -6.107562000 | -2.704851000 |
| 6 | 3.825228000 | -4.282103000 | -0.573600000 |
| 6 | 4.993116000 | -5.993796000 | -1.853224000 |
| 6 | 4.974906000 | -5.059035000 | -0.807820000 |
| 9 | 3.877485000 | -3.429971000 | 0.449429000 |
| 9 | 6.049279000 | -4.921539000 | -0.026031000 |
| 9 | 6.069196000 | -6.754329000 | -2.050246000 |
| 9 | 3.921977000 | -6.950305000 | -3.745764000 |
| 9 | 1.718120000 | -5.456500000 | -3.315860000 |
| 9 | -5.375671000 | 5.355436000 | -3.460668000 |
| 9 | -4.339662000 | 0.809319000 | -2.783411000 |
| 9 | -1.491607000 | 3.598602000 | -5.444720000 |
| 9 | -5.960154000 | 2.908093000 | -2.353339000 |
| 9 | -3.089888000 | 5.685563000 | -4.907886000 |
| 6 | -2.628917000 | -3.629955000 | 2.611442000 |
| 6 | -4.076063000 | -3.886051000 | 3.083663000 |
| 1 | -4.682009000 | -4.156730000 | 2.195274000 |
| 1 | -4.090598000 | -4.768039000 | 3.756014000 |
| 6 | 4.964470000 | 1.120211000 | -7.246559000 |
| 6 | 4.318764000 | 1.179640000 | -8.634162000 |
| 1 | 4.835206000 | 0.482438000 | -9.325913000 |
| 1 | 4.442498000 | 2.200369000 | -9.060322000 |
| 6 | -0.940437000 | -3.820047000 | -8.872034000 |
| 6 | -2.111662000 | -2.855983000 | -8.597193000 |
| 1 | -2.758813000 | -2.809501000 | -9.497501000 |
| 1 | -2.726705000 | -3.286621000 | -7.783265000 |
| 6 | 0.603080000 | 2.142378000 | 2.855429000 |
| 6 | 0.336653000 | 1.122662000 | 3.967840000 |

| | | | |
|---|---|---|---|
| 1 | 1.146079000 | 1.179808000 | 4.730063000 |
| 1 | -0.609869000 | 1.373926000 | 4.491133000 |
| 6 | 0.278354000 | -0.292704000 | 3.386140000 |
| 1 | -0.524685000 | -0.336763000 | 2.623054000 |
| 1 | 0.007329000 | -1.032459000 | 4.168659000 |
| 6 | 1.608341000 | -0.677457000 | 2.736763000 |
| 1 | 1.506018000 | -1.615375000 | 2.155579000 |
| 1 | 2.363689000 | -0.883300000 | 3.530725000 |
| 1 | 0.787543000 | 3.155842000 | 3.271060000 |
| 1 | -0.316117000 | 2.239383000 | 2.231135000 |
| 6 | -3.781098000 | -1.426506000 | 3.829930000 |
| 1 | -2.986642000 | -1.607152000 | 4.590049000 |
| 1 | -4.331600000 | -0.524669000 | 4.166705000 |
| 6 | -4.716619000 | -2.655066000 | 3.783305000 |
| 1 | -5.030417000 | -2.909496000 | 4.816378000 |
| 1 | -5.639858000 | -2.366545000 | 3.240075000 |
| 1 | -2.265594000 | -4.462200000 | 1.976584000 |
| 1 | -1.954710000 | -3.597077000 | 3.498391000 |
| 6 | -0.118130000 | -1.264735000 | -8.136287000 |
| 1 | 0.271677000 | -1.188817000 | -9.177581000 |
| 1 | 0.134996000 | -0.314425000 | -7.630795000 |
| 6 | -1.649378000 | -1.436412000 | -8.173680000 |
| 1 | -2.033673000 | -1.202892000 | -7.162761000 |
| 1 | -2.080897000 | -0.665822000 | -8.843977000 |
| 1 | -1.303298000 | -4.863431000 | -8.973850000 |
| 1 | -0.478200000 | -3.557058000 | -9.851385000 |
| 1 | 6.016681000 | 1.473143000 | -7.275343000 |
| 1 | 5.019377000 | 0.056378000 | -6.915673000 |
| 6 | 2.096329000 | 1.864930000 | -7.657342000 |
| 1 | 1.972938000 | 2.813083000 | -8.229790000 |
| 1 | 1.065006000 | 1.529823000 | -7.421074000 |
| 6 | 2.827509000 | 0.845022000 | -8.535985000 |
| 1 | 2.718481000 | -0.172975000 | -8.103784000 |
| 1 | 2.359432000 | 0.807256000 | -9.541483000 |
| 6 | -6.099639000 | -4.104624000 | -0.432944000 |
| 6 | -6.597739000 | -3.096948000 | 0.388390000 |
| 6 | -6.093870000 | -1.768005000 | 0.308563000 |
| 6 | -5.073323000 | -3.782558000 | -1.358107000 |
| 6 | -4.560012000 | -2.436283000 | -1.401858000 |
| 6 | -5.079471000 | -1.417292000 | -0.579346000 |
| 6 | -4.389606000 | -4.512680000 | -2.372044000 |
| 6 | -3.476448000 | -3.633969000 | -2.977709000 |
| 7 | -3.579446000 | -2.392274000 | -2.358601000 |
| 6 | -2.581692000 | -3.876937000 | -4.085921000 |
| 6 | -2.590919000 | -5.238666000 | -4.715483000 |
| 1 | -3.598864000 | -5.680787000 | -4.793319000 |
| 1 | -1.959064000 | -5.898971000 | -4.083936000 |

| | | | |
|---|---|---|---|
| 1 | -2.121806000 | -5.193609000 | -5.715728000 |
| 6 | -1.271660000 | -3.178798000 | -4.122948000 |
| 1 | -0.789399000 | -3.236299000 | -5.115814000 |
| 1 | -0.624701000 | -3.719859000 | -3.394920000 |
| 1 | -1.293306000 | -2.135313000 | -3.765980000 |
| 1 | -3.082064000 | -1.546575000 | -2.658596000 |
| 8 | -3.490198000 | -2.838887000 | -5.503382000 |
| 14 | -5.201369000 | -2.692603000 | -5.858633000 |
| 6 | -5.763464000 | -4.430769000 | -6.296203000 |
| 6 | -6.159845000 | -1.977456000 | -4.412692000 |
| 1 | -6.225124000 | -2.674628000 | -3.555081000 |
| 1 | -7.194139000 | -1.748560000 | -4.744763000 |
| 1 | -5.696852000 | -1.034727000 | -4.060521000 |
| 1 | -5.095965000 | -4.893410000 | -7.050476000 |
| 1 | -6.788359000 | -4.399440000 | -6.719652000 |
| 1 | -5.793245000 | -5.081629000 | -5.399435000 |
| 1 | -7.396320000 | -3.324626000 | 1.110156000 |
| 1 | -6.512888000 | -0.995655000 | 0.970947000 |
| 1 | -6.496482000 | -5.129354000 | -0.377619000 |
| 1 | -4.695210000 | -0.392413000 | -0.638466000 |
| 1 | -4.521502000 | -5.569756000 | -2.624799000 |
| 6 | -5.276414000 | -1.504661000 | -7.314596000 |
| 1 | -4.786479000 | -1.917518000 | -8.217084000 |
| 1 | -6.333453000 | -1.274635000 | -7.561864000 |
| 1 | -4.770361000 | -0.554997000 | -7.046751000 |
| 1 | -3.117908000 | -1.920021000 | -5.302118000 |

# 13. References


(1) Mi, Y.; Schreiber, J. V.; Corey, E. J., Total Synthesis of (+)-α-Onocerin in Four Steps via Four-Component Coupling and Tetracyclization Steps. *J. Am. Chem. Soc.* **2002**, *124*, 11290–11291.

(2) Xu, J. S.; Rawal, V. H., Total Synthesis of (−)-Ambiguine P. *J. Am. Chem. Soc.* **2019**, *141*, 4820–4823.

(3) Kaib, P. S.; Schreyer, L.; Lee, S.; Properzi, R.; List, B., Extremely Active Organocatalysts Enable a Highly Enantioselective Addition of Allyltrimethylsilane to Aldehydes. *Angew. Chem. Int. Ed.* **2016**, *55*, 13200–13203.

(4) Liu, L.; Kim, H.; Xie, Y.; Fares, C.; Kaib, P. S. J.; Goddard, R.; List, B., Catalytic Asymmetric [4+2]-Cycloaddition of Dienes with Aldehydes. *J. Am. Chem. Soc.* **2017**, *139*, 13656–13659.

(5) Tsuji, N.; Kennemur, J. L.; Buyck, T.; Lee, S.; Prévost, S.; Kaib, P. S. J.; Bykov, D.; Farès, C.; List, B., Activation of olefins via asymmetric Brønsted acid catalysis. *Science* **2018**, *359*, 1501–1505.

(6) Properzi, R.; Kaib, P. S. J.; Leutzsch, M.; Pupo, G.; Mitra, R.; De, C. K.; Song, L.; Schreiner, P. R.; List, B., Catalytic enantiocontrol over a non-classical carbocation. *Nat. Chem.* **2020**, *12*, 1174–1179.

(7) de Waard, E. R.; Kattenberg, J.; Huisman, H. O., Synthesis and reactions of 3,4-dihydroanisole. *Tetrahedron Lett.* **1970**, *11*, 4427–4428.

(8) Neese, F., The ORCA program system. *WIREs Comput Mol Sci* **2012**, *2*, 73–78.

(9) Zhang, Y. K.; Yang, W. T., Comment on "Generalized Gradient Approximation Made Simple". *Phys. Rev. Lett.* **1998**, *80*, 890-890.

(10) (a) Grimme, S.; Antony, J.; Ehrlich, S.; Krieg, H., A consistent and accurate ab initio parametrization of density functional dispersion correction (DFT-D) for the 94 elements H-Pu. *J. Chem. Phys.* **2010**, *132*, 154104; (b) Grimme, S.; Ehrlich, S.; Goerigk, L., Effect of the damping function in dispersion corrected density functional theory. *J. Comput. Chem.* **2011**, *32*, 1456–1465.

(11) Weigend, F.; Ahlrichs, R., Balanced basis sets of split valence, triple zeta valence and quadruple zeta valence quality for H to Rn: Design and assessment of accuracy. *Phys. Chem. Chem. Phys.* **2005**, *7*, 3297–3305.

(12) Barone, V.; Cossi, M., Quantum Calculation of Molecular Energies and Energy Gradients in Solution by a Conductor Solvent Model. *J. Phys. Chem. A* **1998**, *102*, 1995–2001.

(13) Becke, A. D., Density - functional thermochemistry. III. The role of exact exchange. *J. Chem. Phys.* **1993**, *98*, 5648–5652.

(14) Lee, C.; Yang, W.; Parr, R. G., Development of the Colle-Salvetti correlation-energy formula into a functional of the electron density. *Phys. Rev. B* **1988**, *37*, 785–789.

(15) Bickelhaupt, F. M.; Houk, K. N., Analyzing Reaction Rates with the Distortion/Interaction-Activation Strain Model. *Angew. Chem. Int. Ed.* **2017**, *56*, 10070–10086.

(16) Contreras-García, J.; Johnson, E. R.; Keinan, S.; Chaudret, R.; Piquemal, J. P.; Beratan, D. N.; Yang, W., NCIPLOT: a program for plotting non-covalent interaction regions. *J. Chem. Theory. Comput.* **2011**, *7*, 625–632.

(17) Legault, C. Y. CYLview, 1.0b; Université de Sherbrooke, 2009 (http://www.cylview.org).

(18) Banik, S. M.; Levina, A.; Hyde, A. M.; Jacobsen, E. N., Lewis acid enhancement by hydrogen-bond donors for asymmetric catalysis. *Science* **2017**, *358*, 761–764.

(19) (a) Krenske, E. H.; Houk, K. N.; Lohse, A. G.; Antoline, J. E.; Hsung, R. P., Stereoselectivity in oxyallyl–furan (4 + 3) cycloadditions: control of intermediate conformations and dispersive stabilisation in cycloadditions involving oxazolidinone auxiliaries. *Chem. Sci.* **2010**, *1*, 387–392; (b) Krenske, E. H.; Lam, S.; Ng, J. P. L.; Lo, B.; Lam, S.; Chiu, P.; Houk, K. N., Concerted Ring Opening and Cycloaddition of Chiral Epoxy Enolsilanes with Dienes. *Angew. Chem. Int. Ed.* **2015**, *54*, 7422–7425.

## 14. NMR ans HPLC traces

NMR spectra for compound **1b**: $^{1}H$ (501 MHz) and $^{13}C$ (126 MHz) in $CD_2Cl_2$.

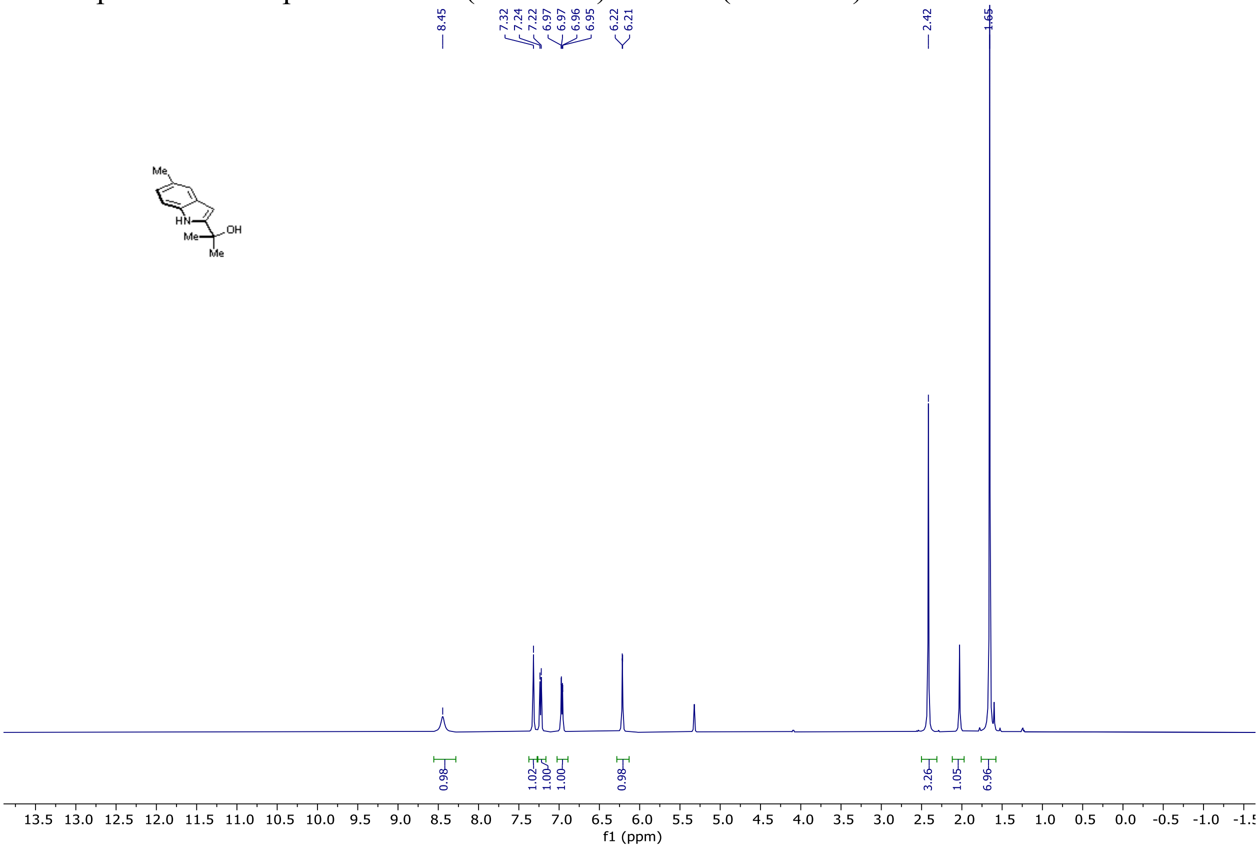


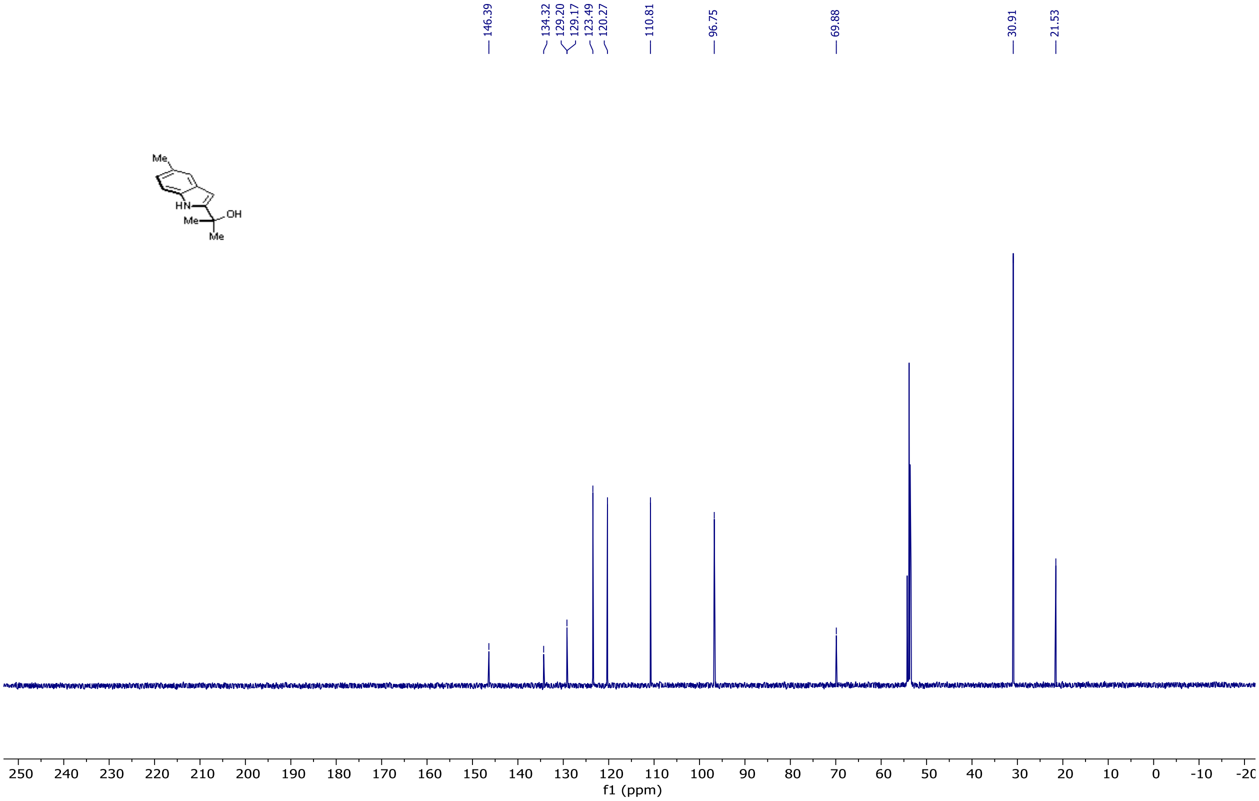

NMR spectra for compound 2**c**: $^{1}H$ (501 MHz) and $^{13}C$ (126 MHz) in $CD_2Cl_2$.

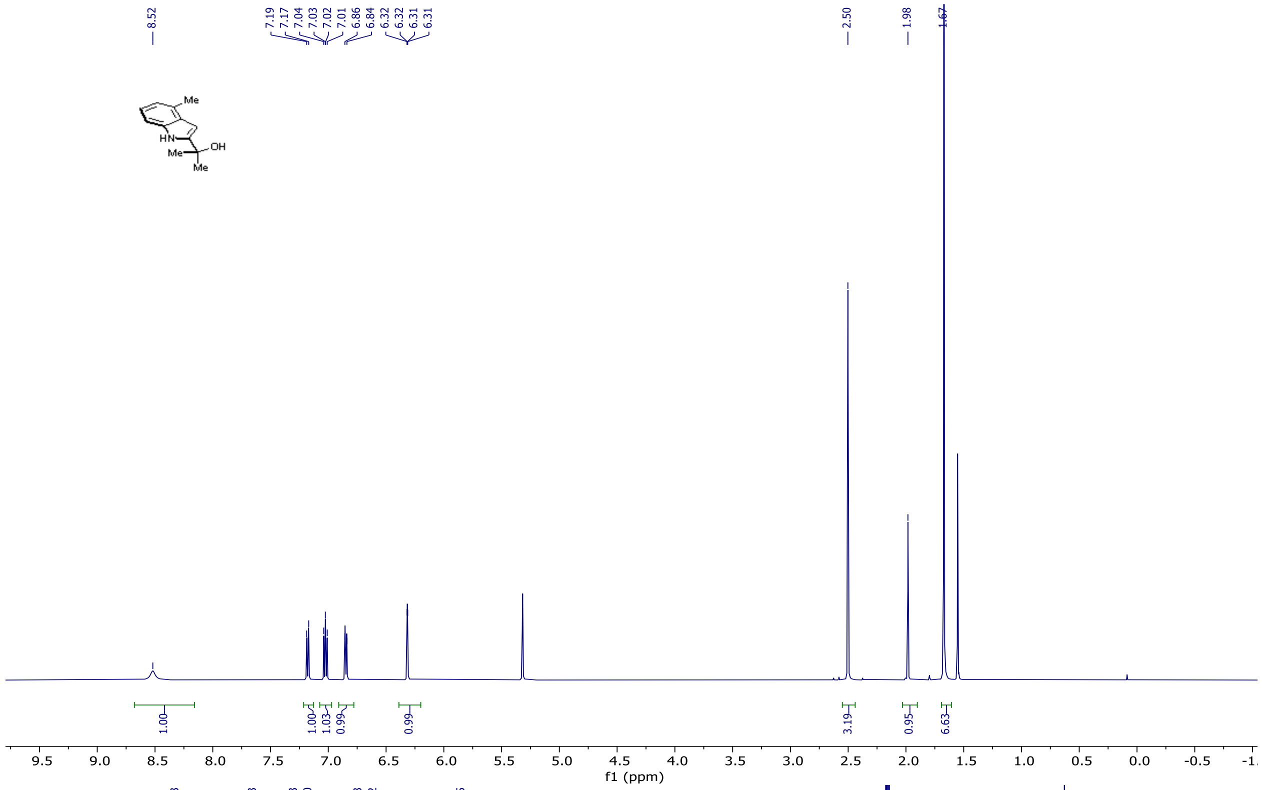


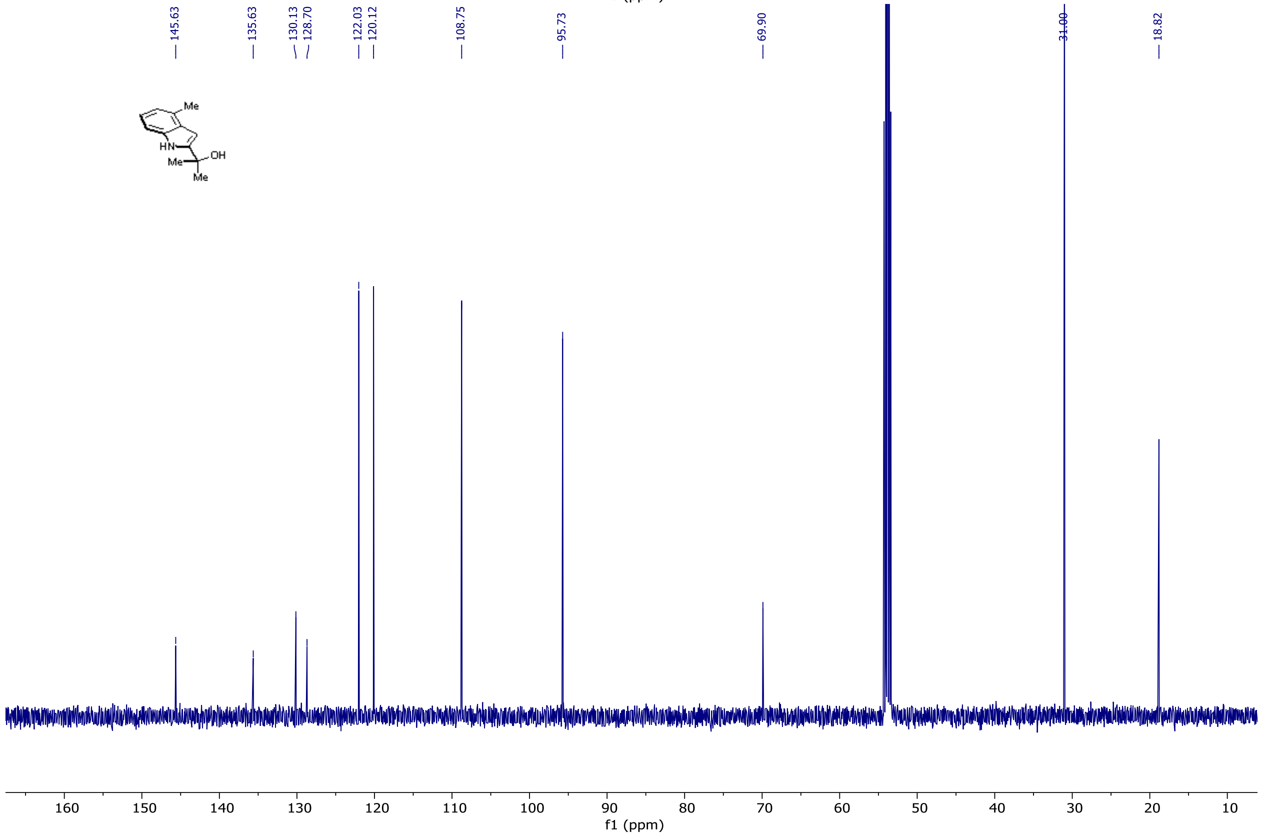

NMR spectra for compound **1d**: $^{1}H$ (501 MHz) and $^{13}C$ (126 MHz) in $CD_2Cl_2$.

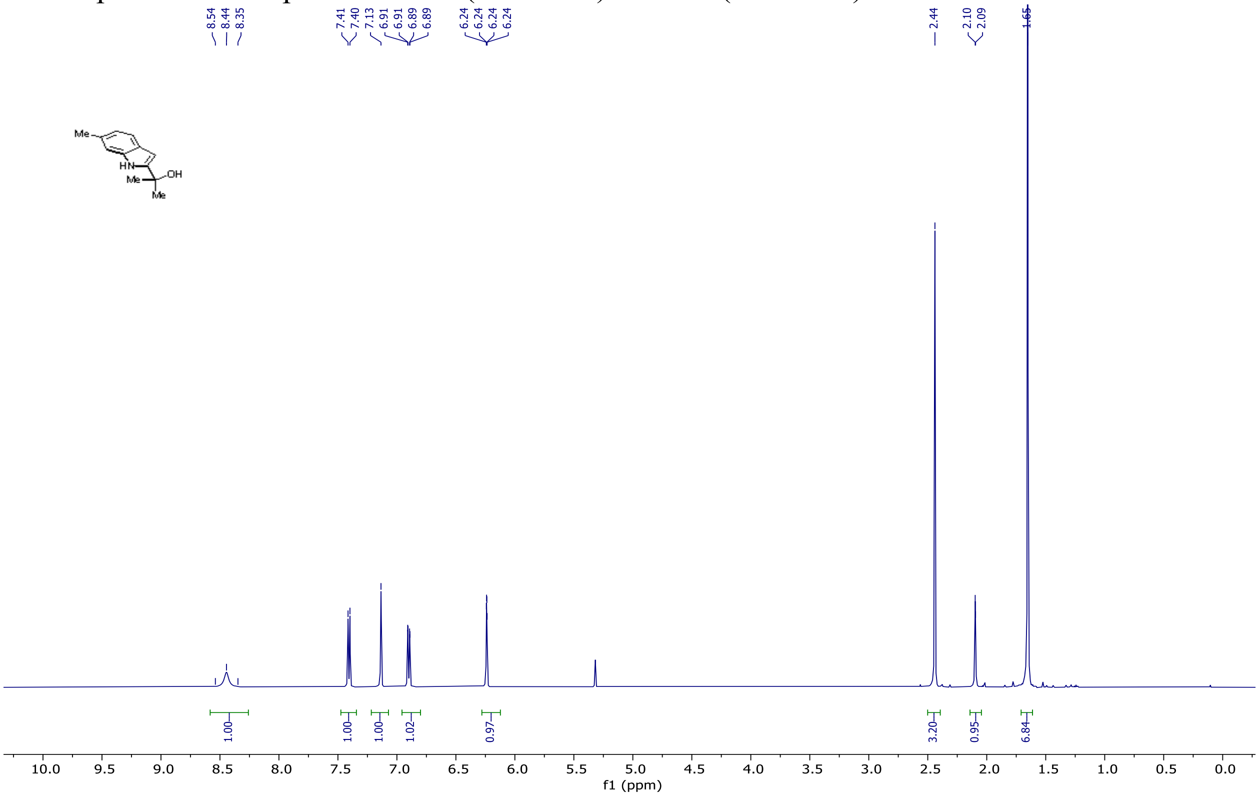


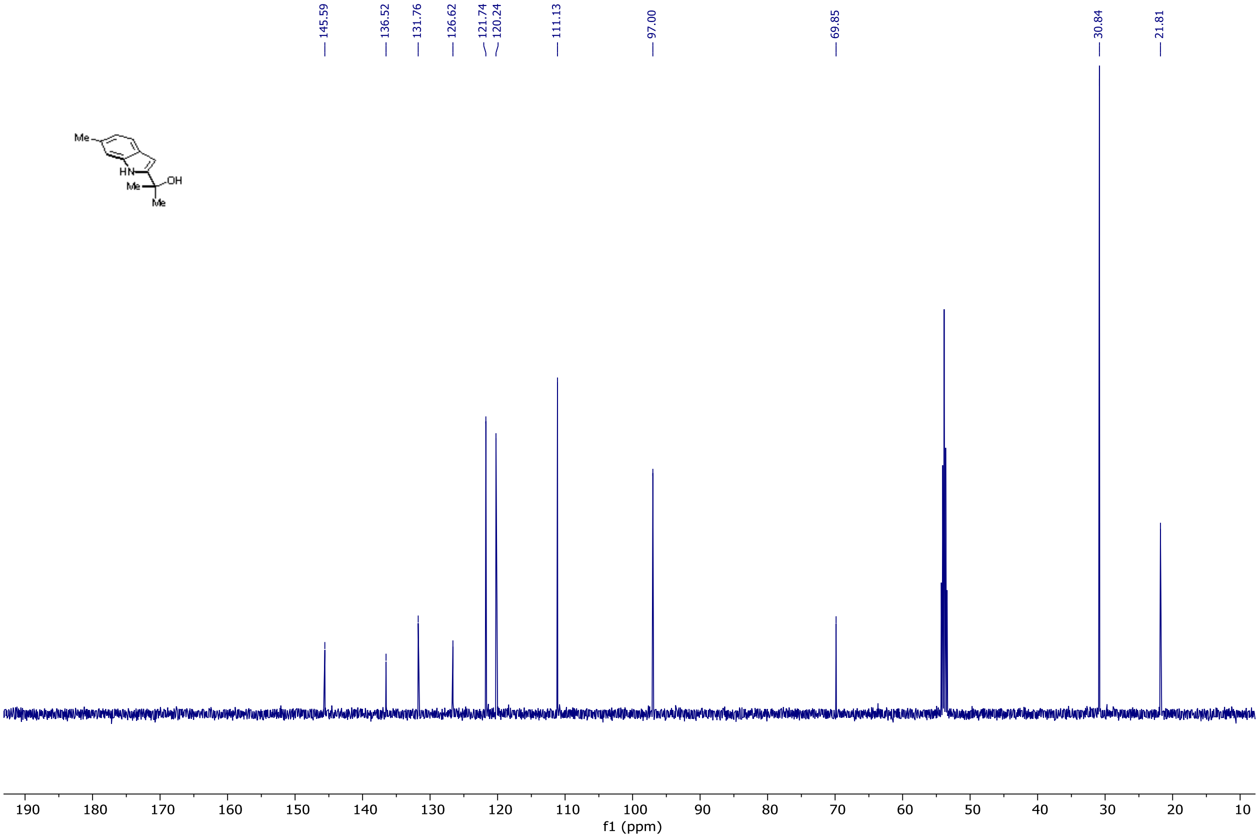

NMR spectra for compound **1e**: $^{1}H$ (501 MHz) and $^{13}C$ (126 MHz) in acetonitrile-d3.

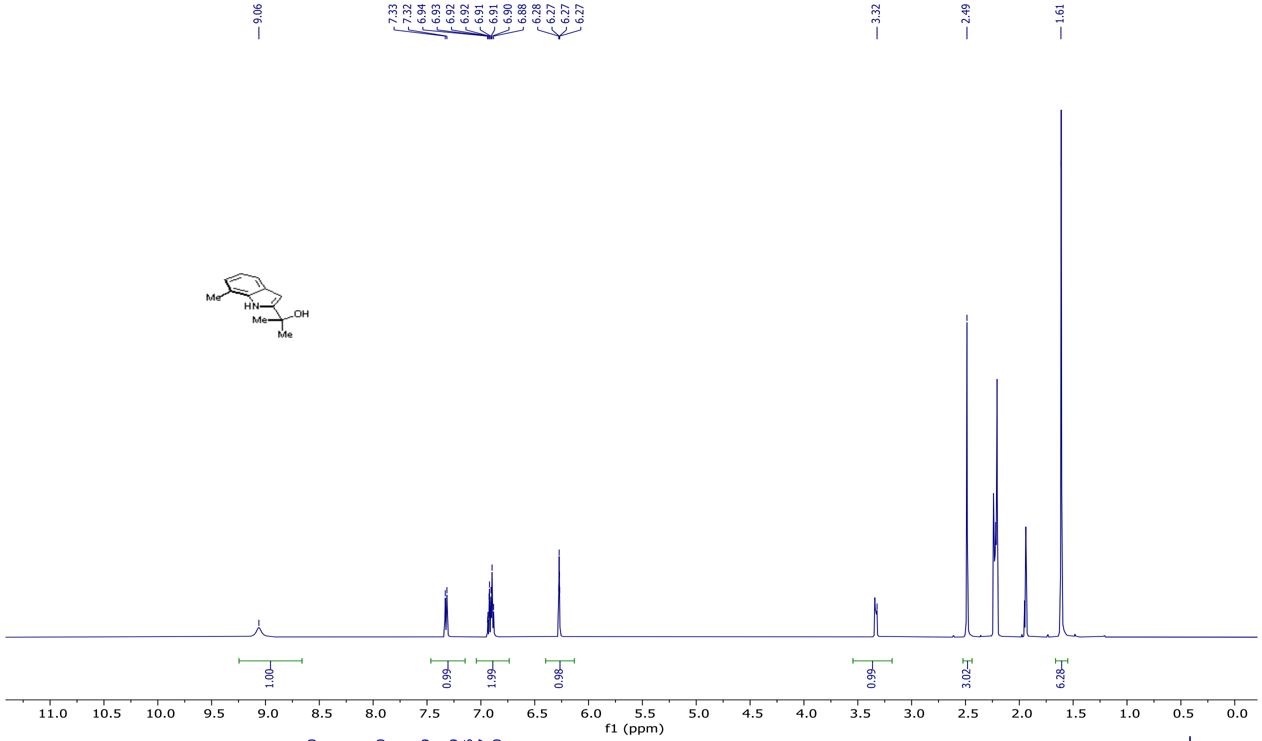


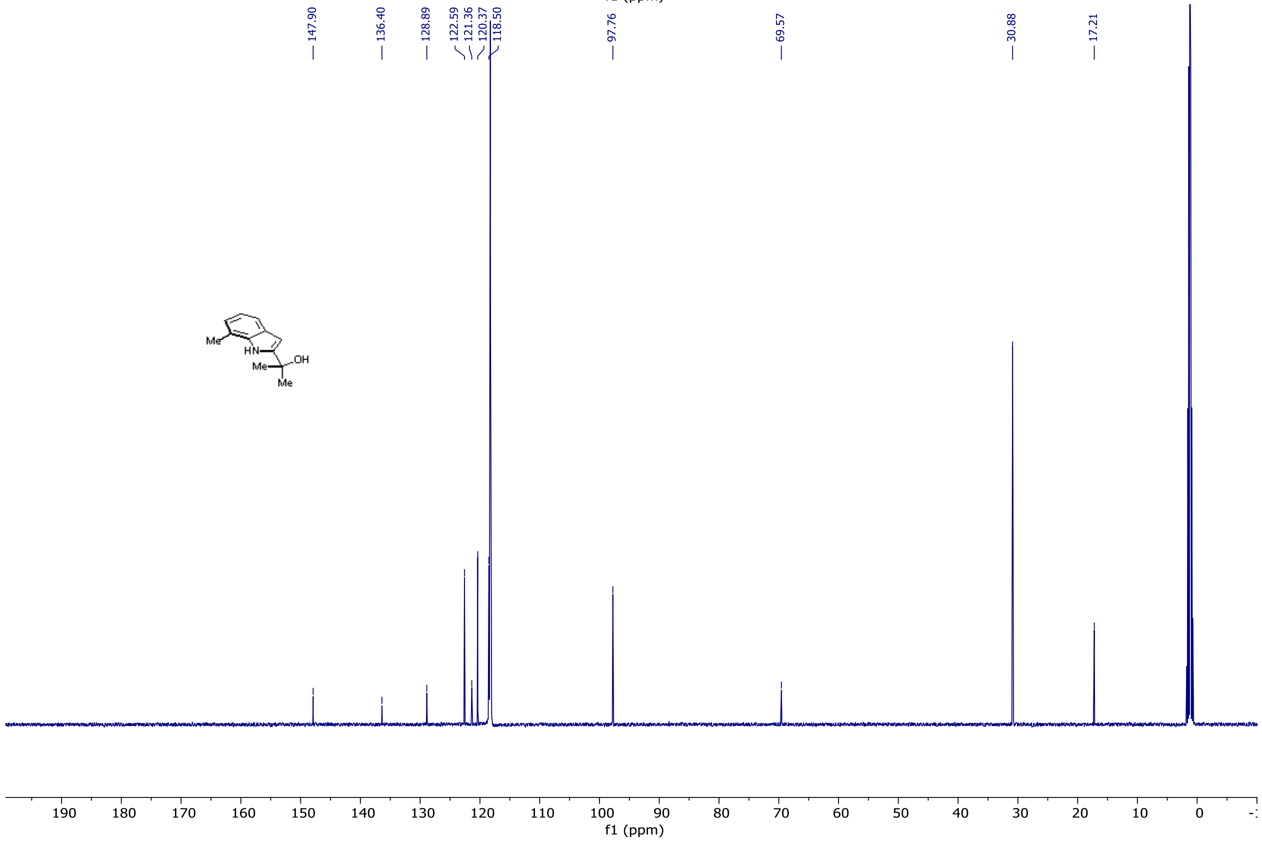

NMR spectra for compound **1f**: $^{1}H$ (501 MHz) and $^{13}C$ (126 MHz) in $CD_2Cl_2$.

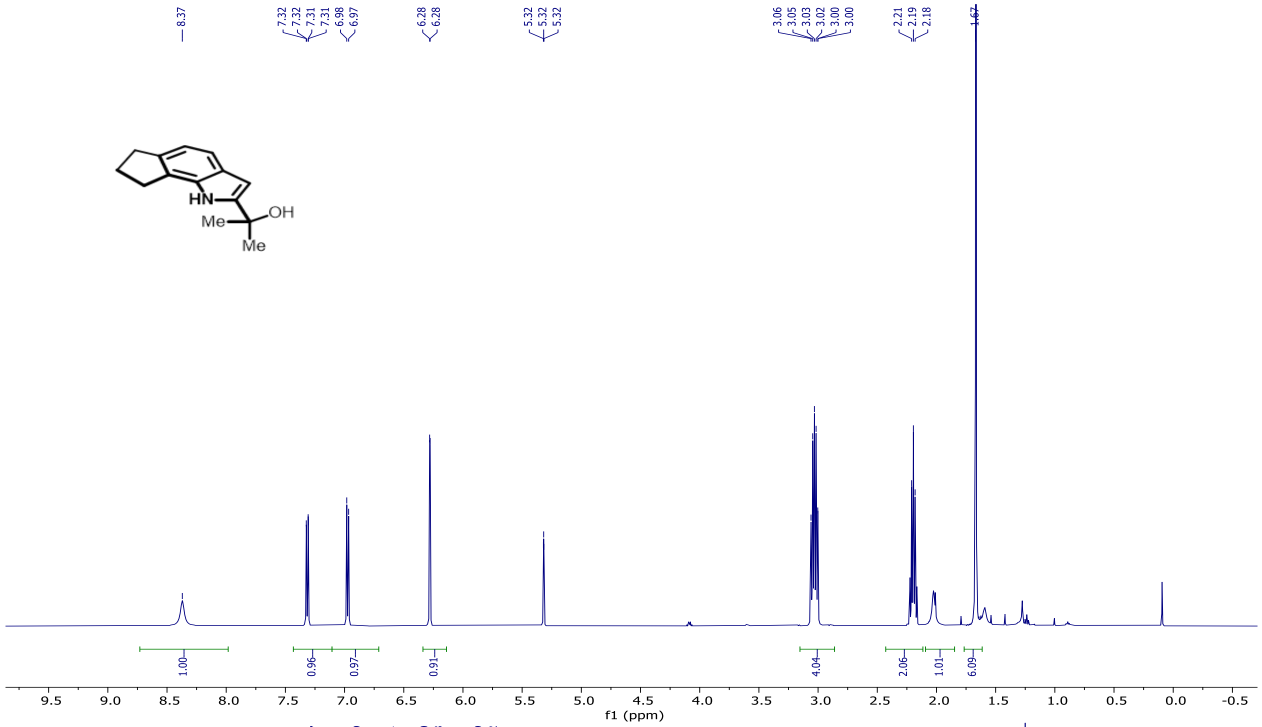


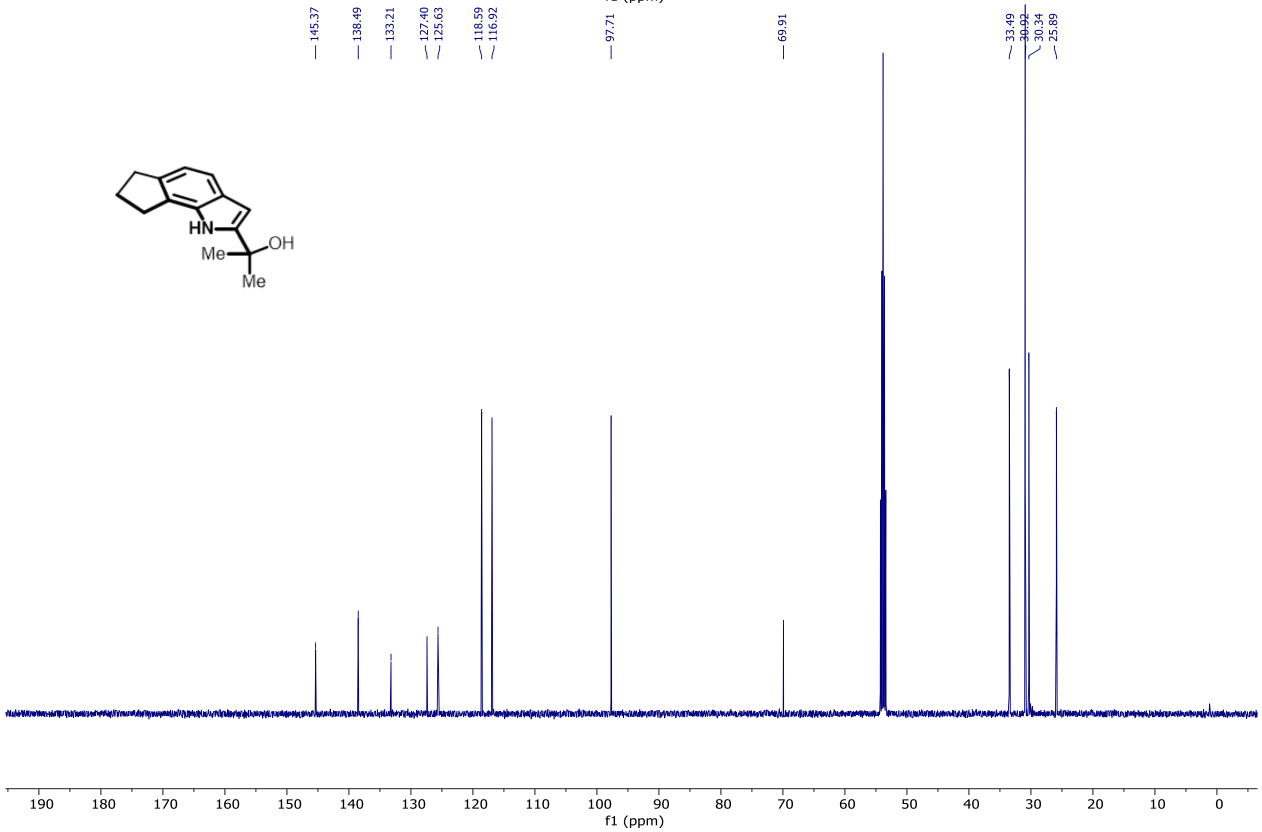

NMR spectra for compound **1g**: $^{1}$H (501 MHz) and $^{13}$C (126 MHz) in acetonitrile-d3.

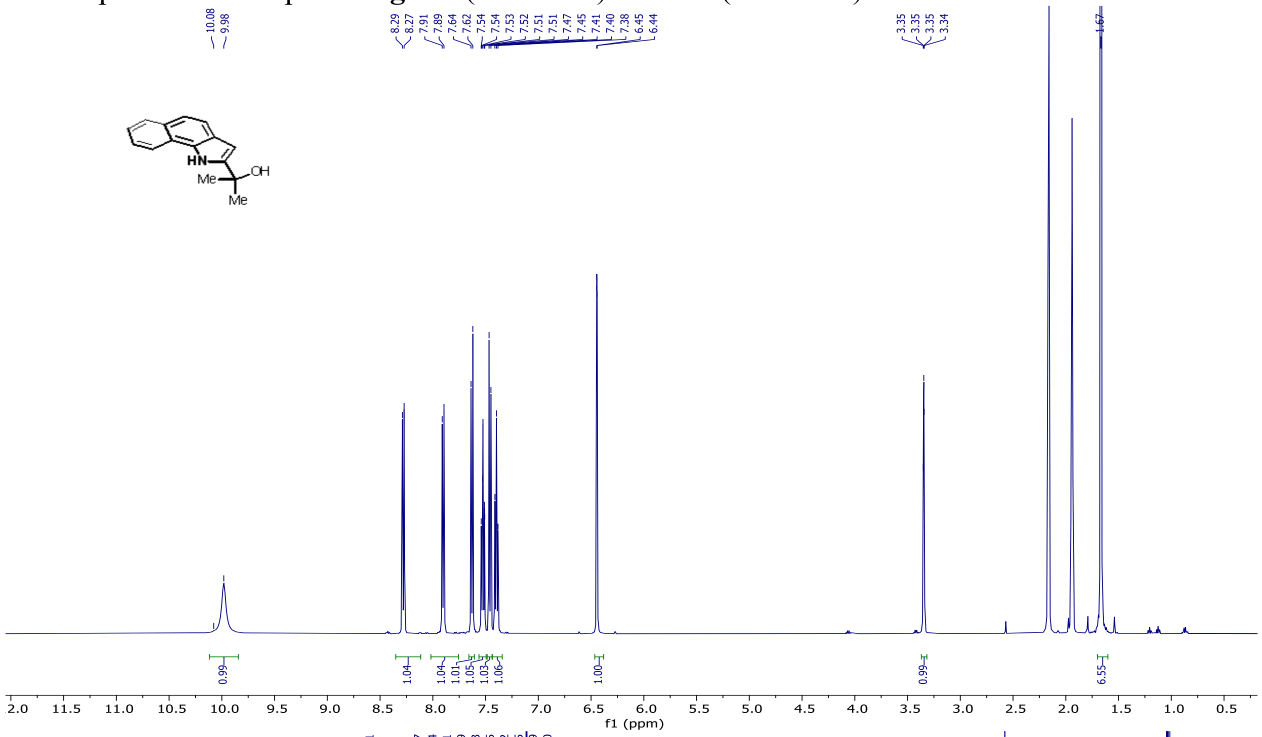


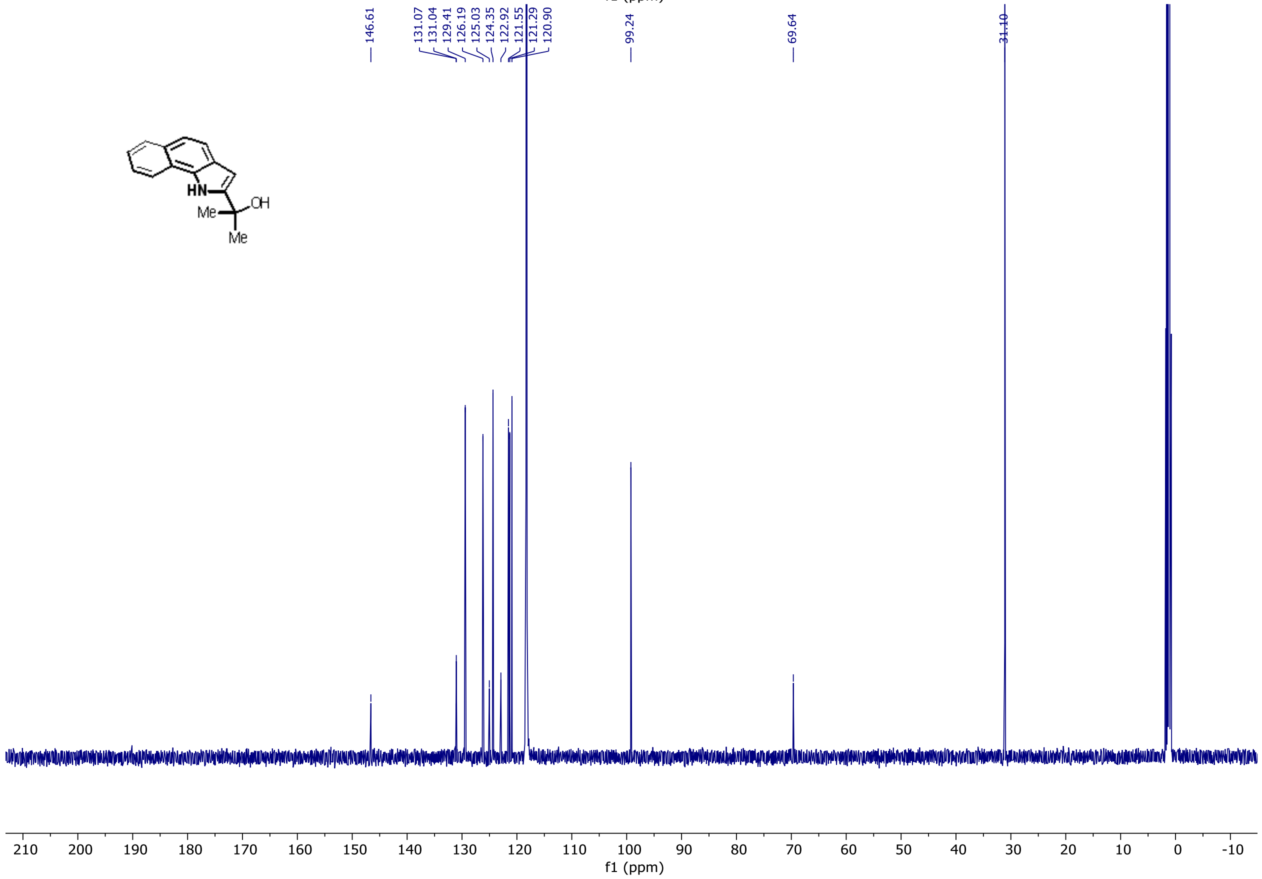

NMR spectra for compound **1h**: $^{1}H$ (501 MHz) and $^{13}C$ (126 MHz) in $CD_2Cl_2$.

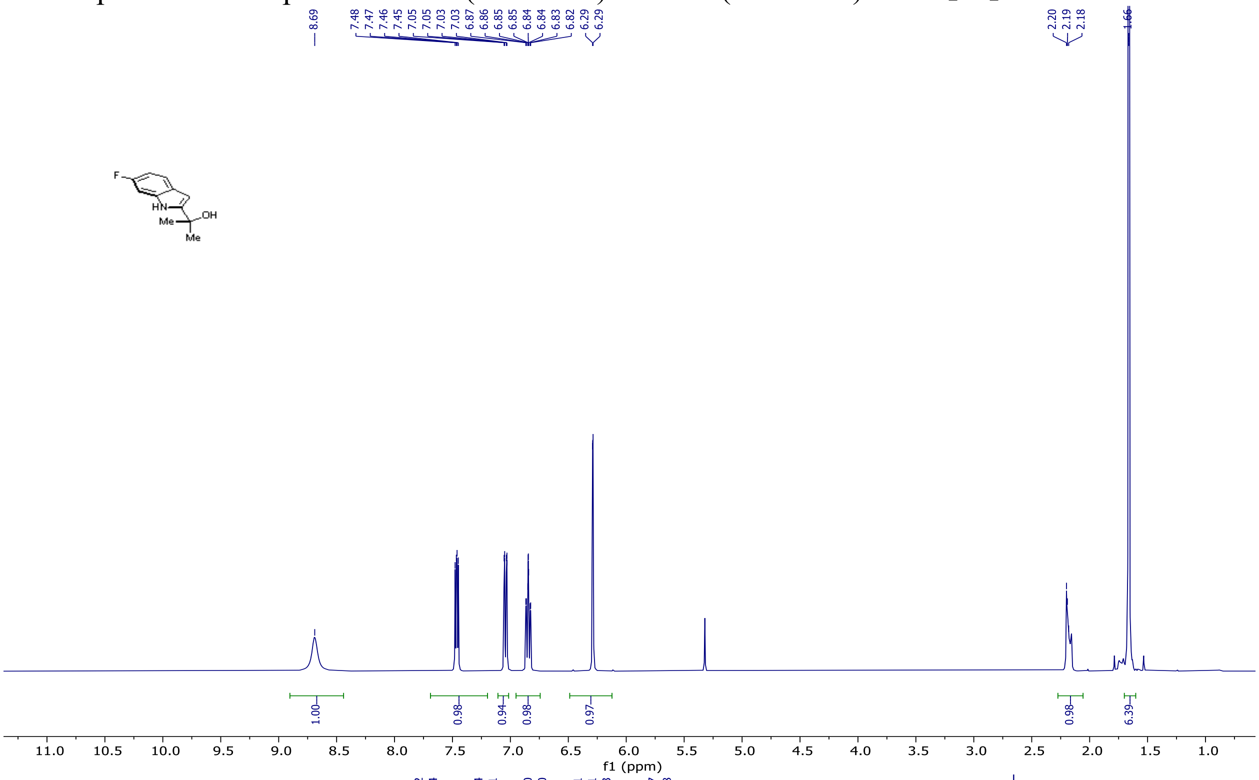


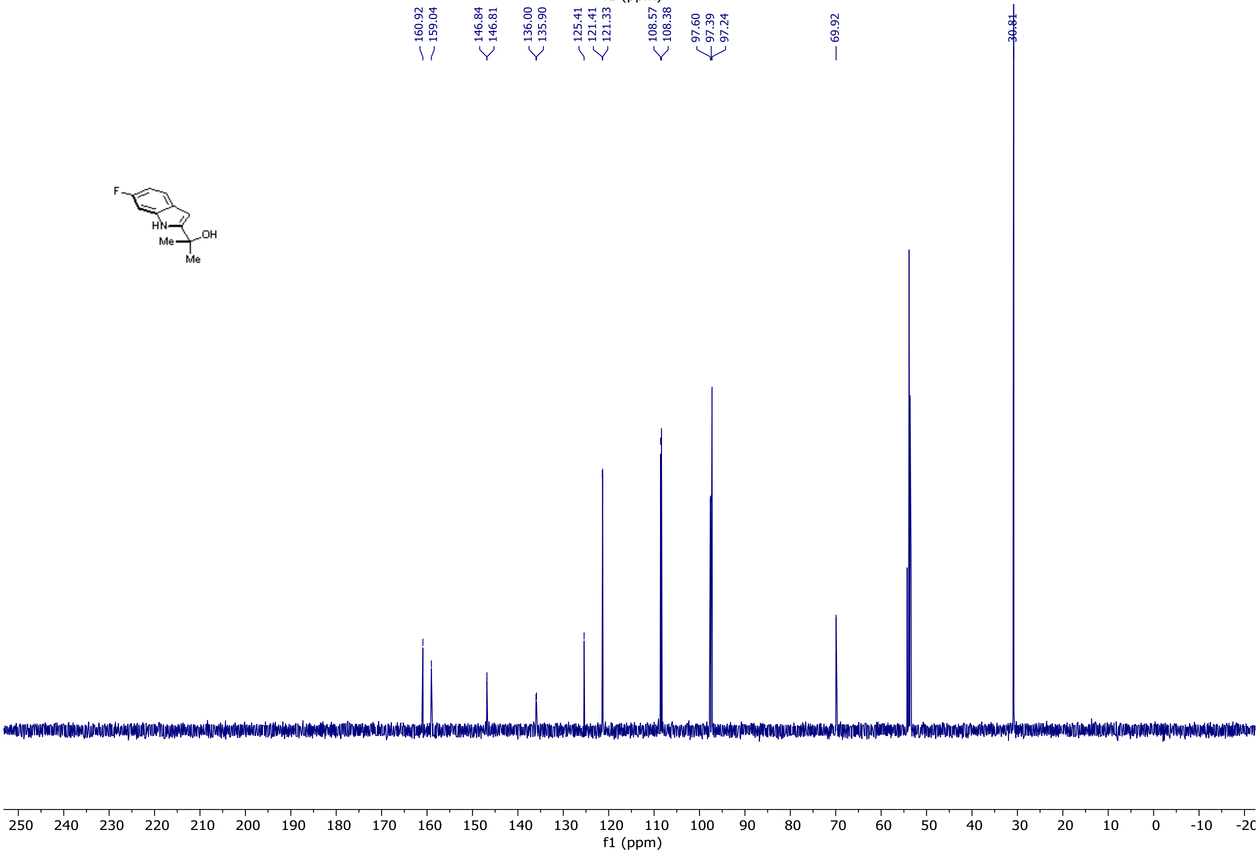

NMR spectra for compound **1i**: $^{1}$H (501 MHz) and $^{13}$C (126 MHz) in acetonitrile-d3.

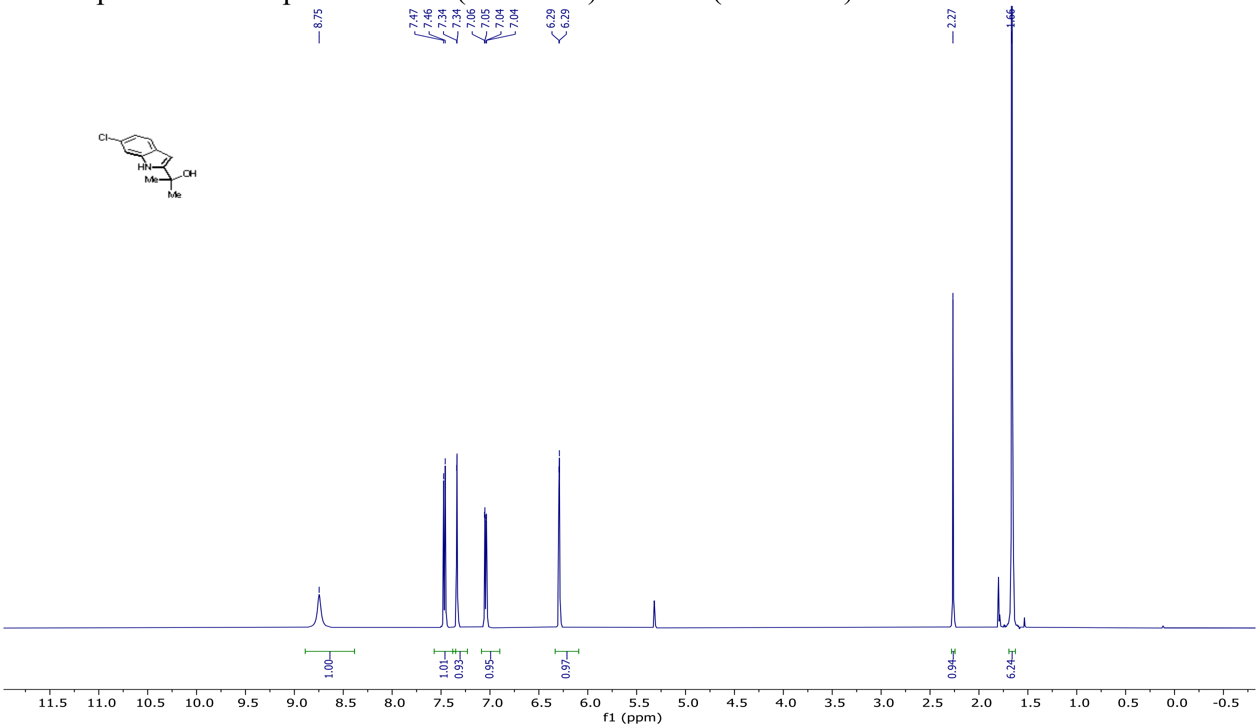


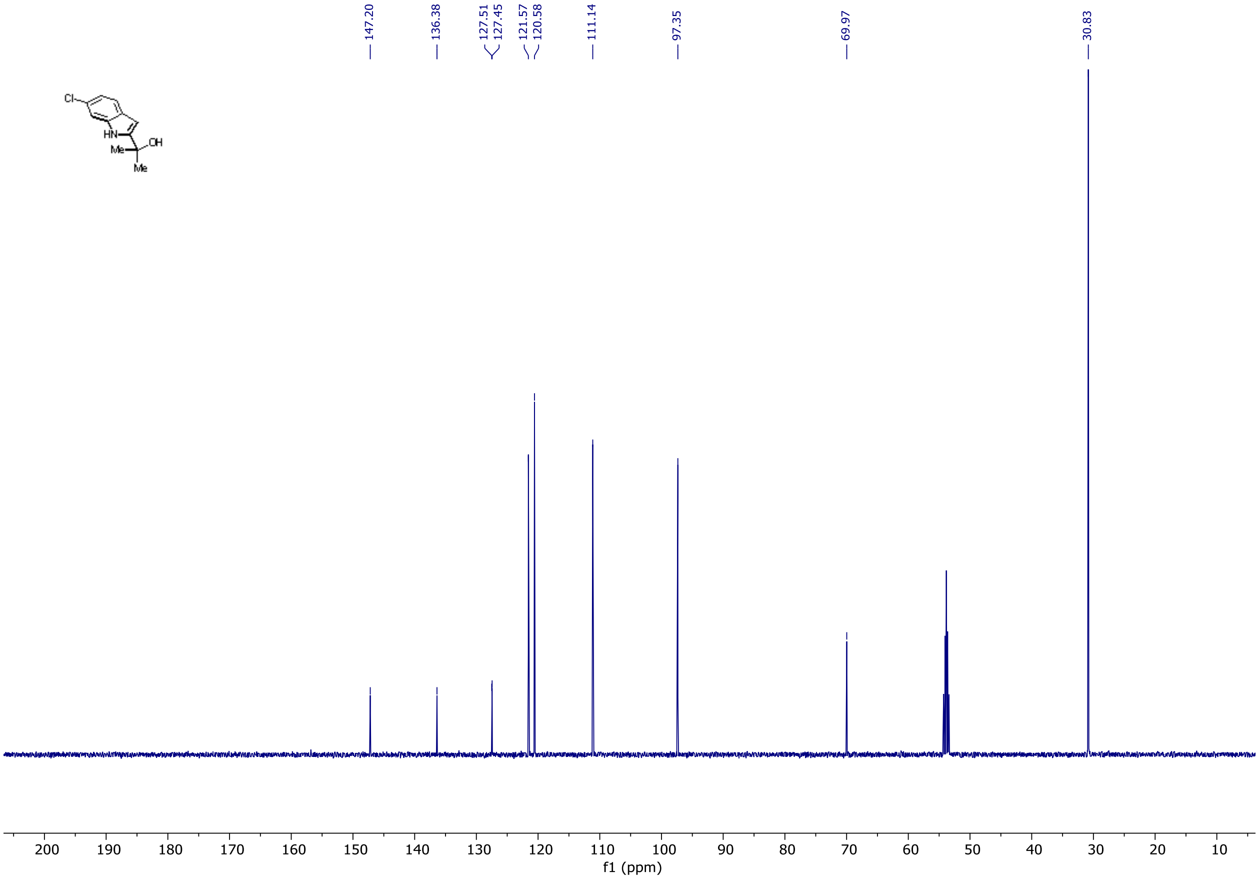

NMR spectra for compound **1j**: $^{1}$H (501 MHz) in acetonitrile-d3, and $^{13}$C (126 MHz) in $CD_2Cl_2$.

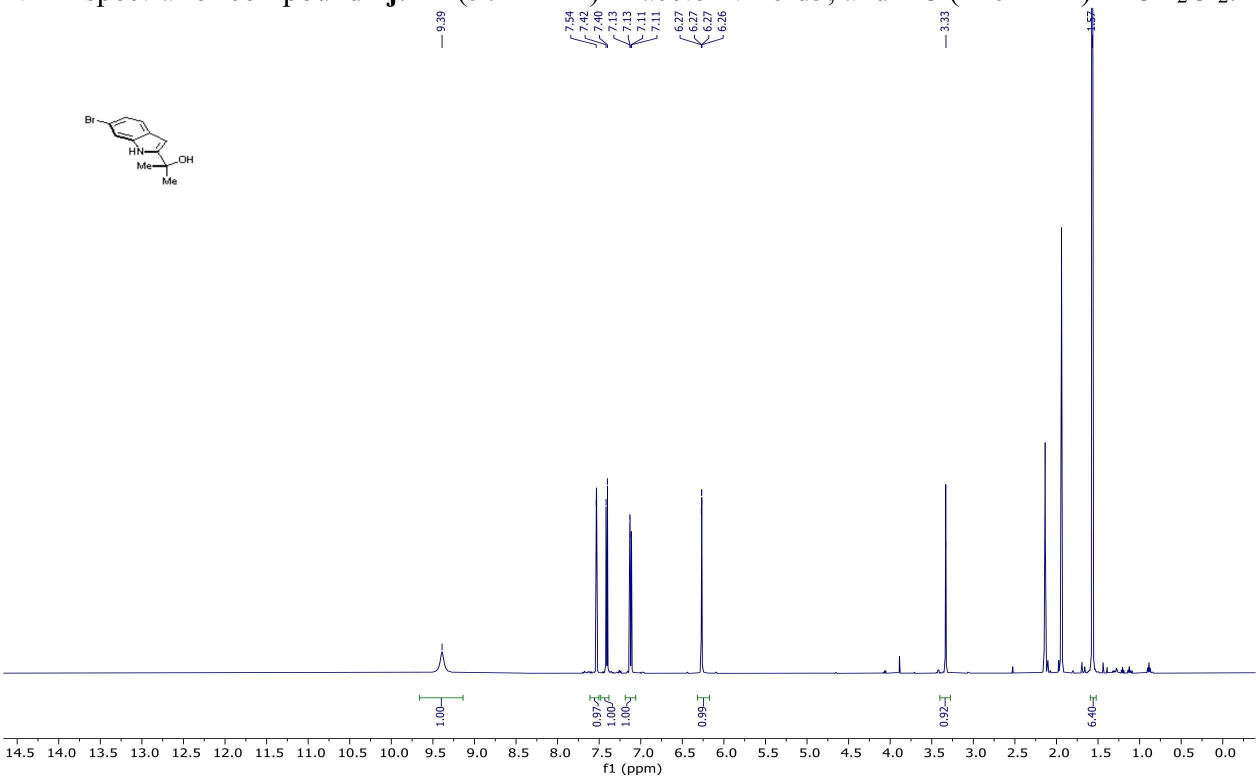


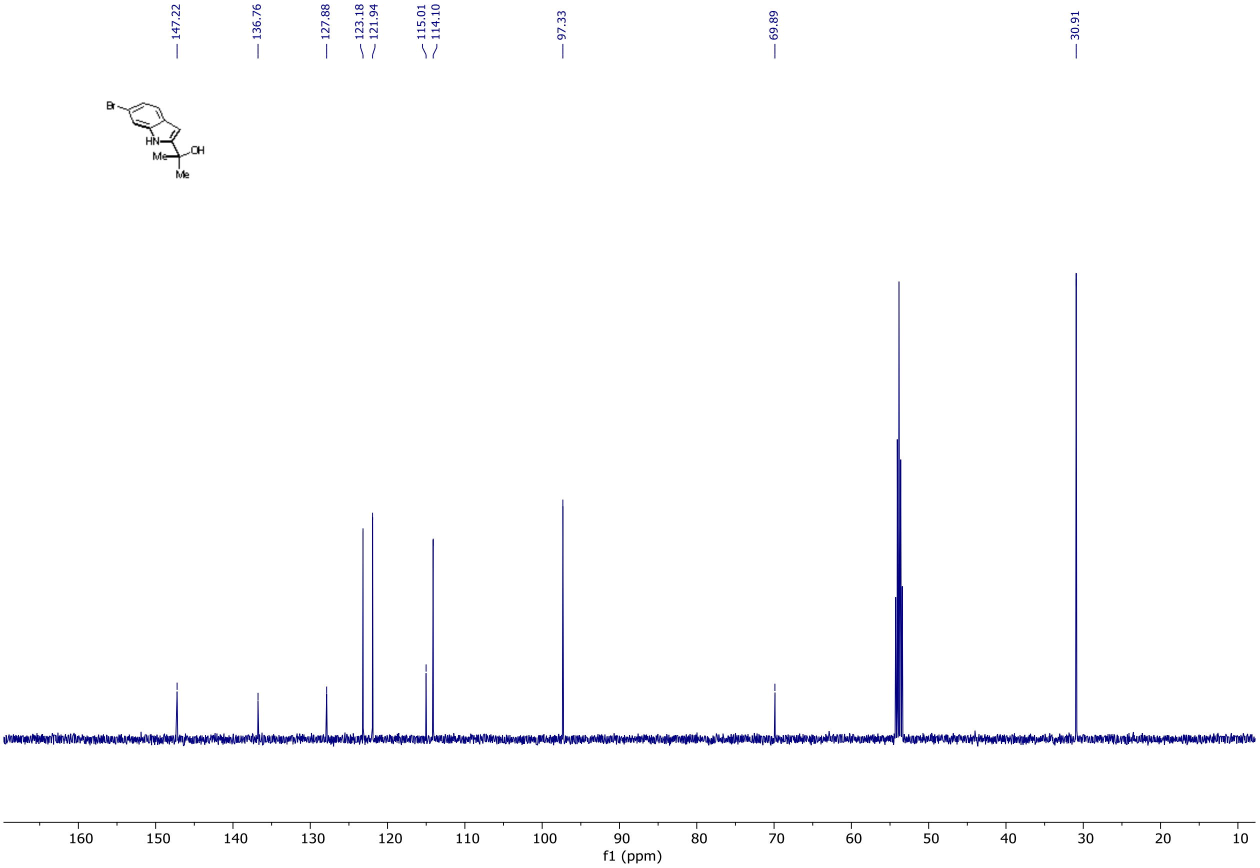

NMR spectra for compound **1k**: $^{1}H$ (501 MHz) and $^{13}C$ (126 MHz) in $CD_2Cl_2$.

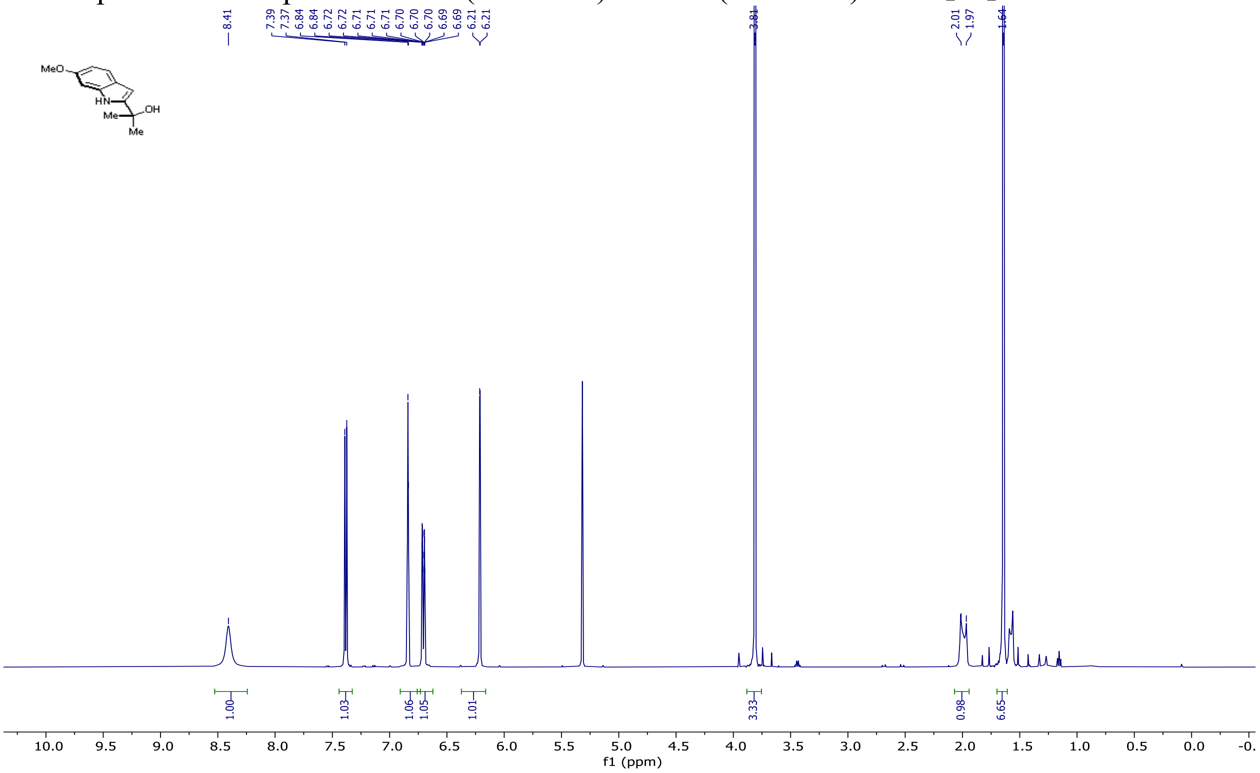

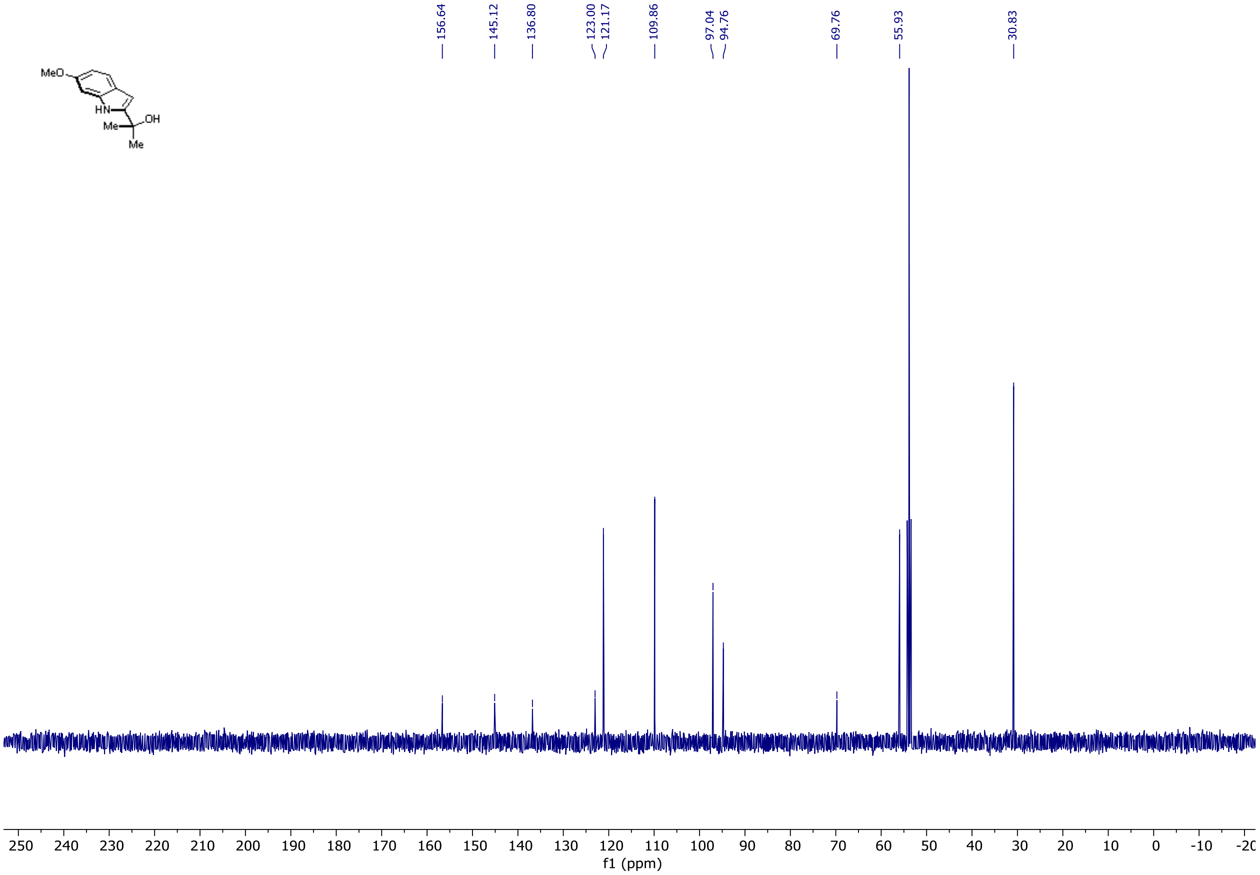

NMR spectra for compound **1l**: $^{1}$H (501 MHz) and $^{13}$C (126 MHz) in $CD_2Cl_2$.

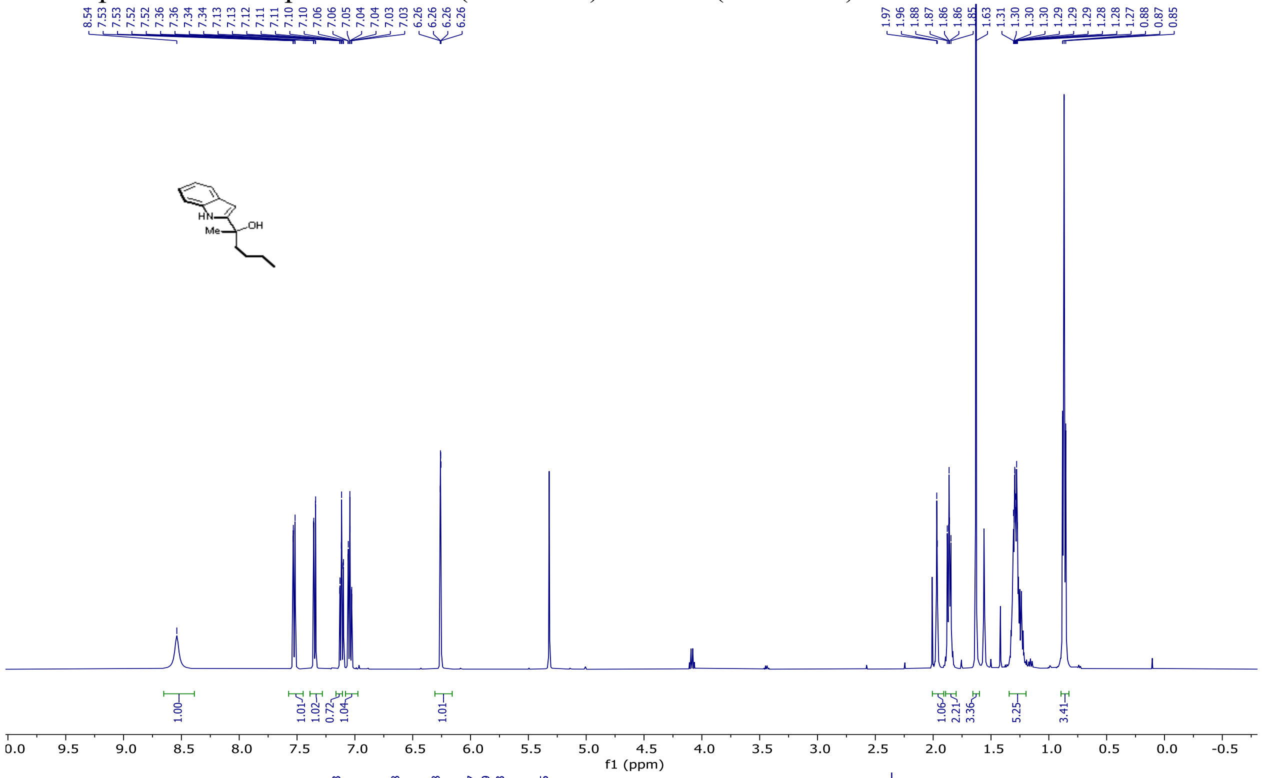

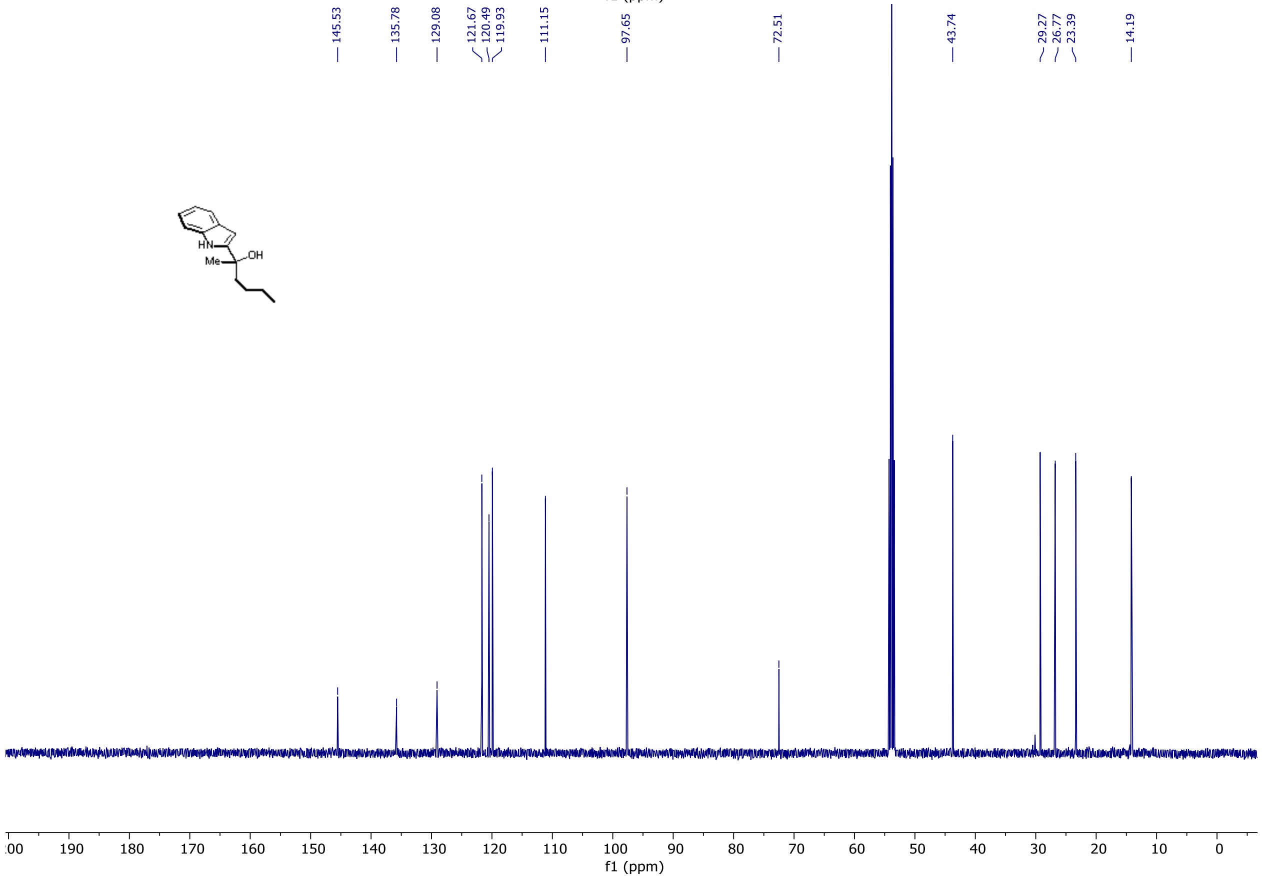

NMR spectra for compound **1m**: $^{1}$H (501 MHz) in and $^{13}$C (126 MHz) in $CD_2Cl_2$.

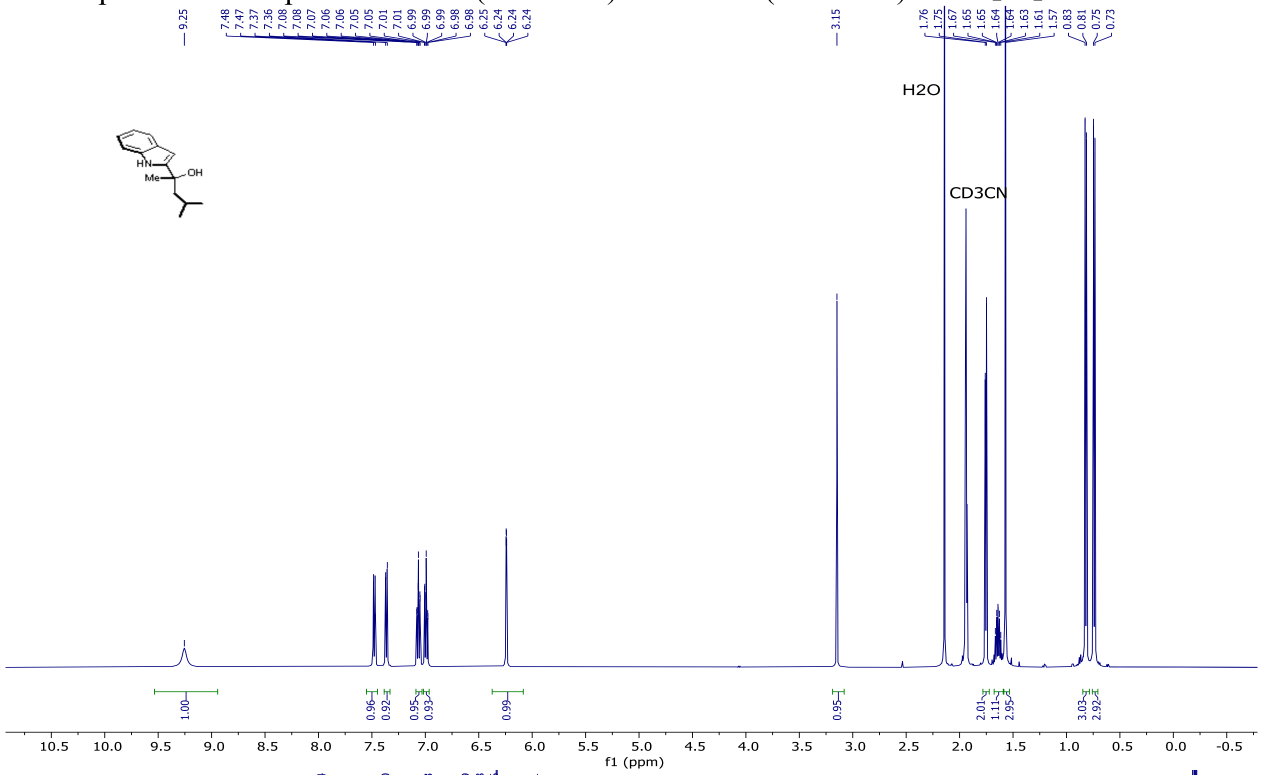


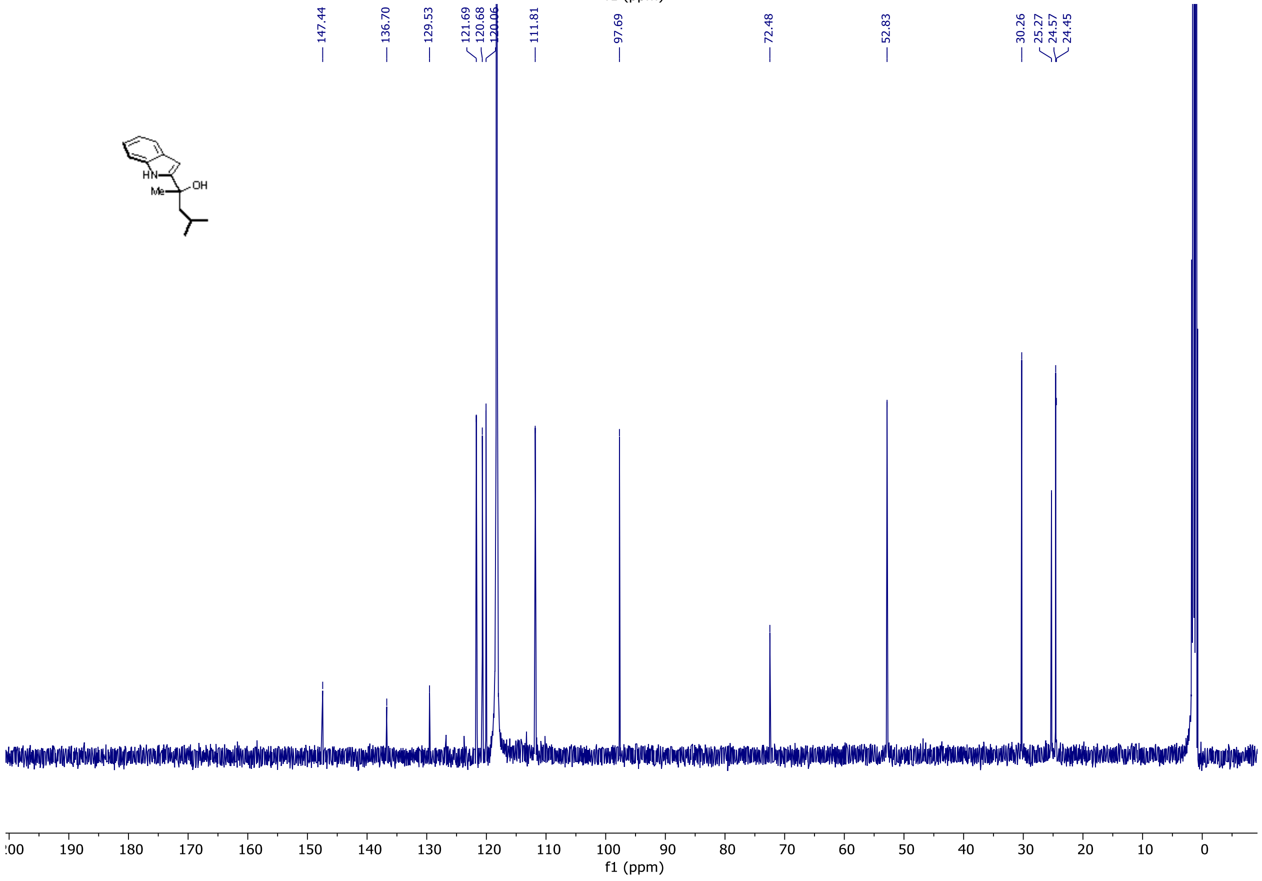

NMR spectra for compound **1n**: $^1$H (501 MHz) and $^{13}$C (126 MHz) in $CD_2Cl_2$.

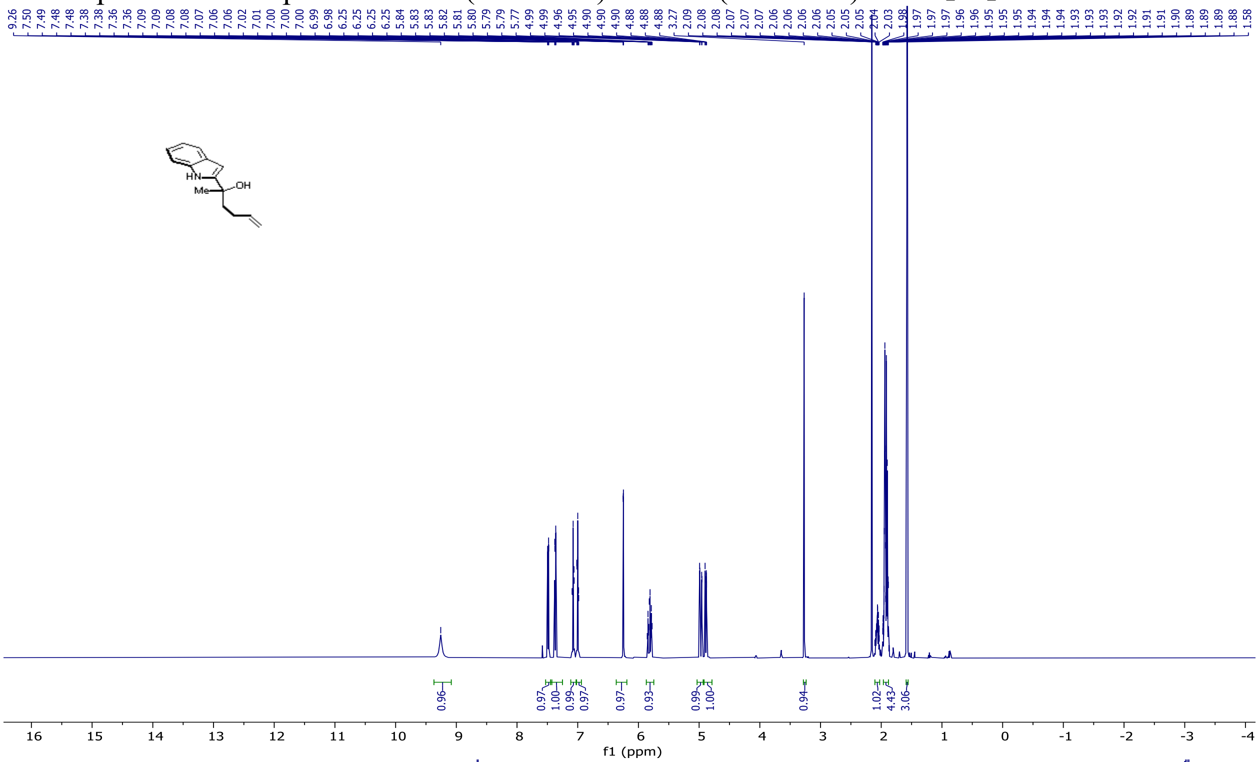

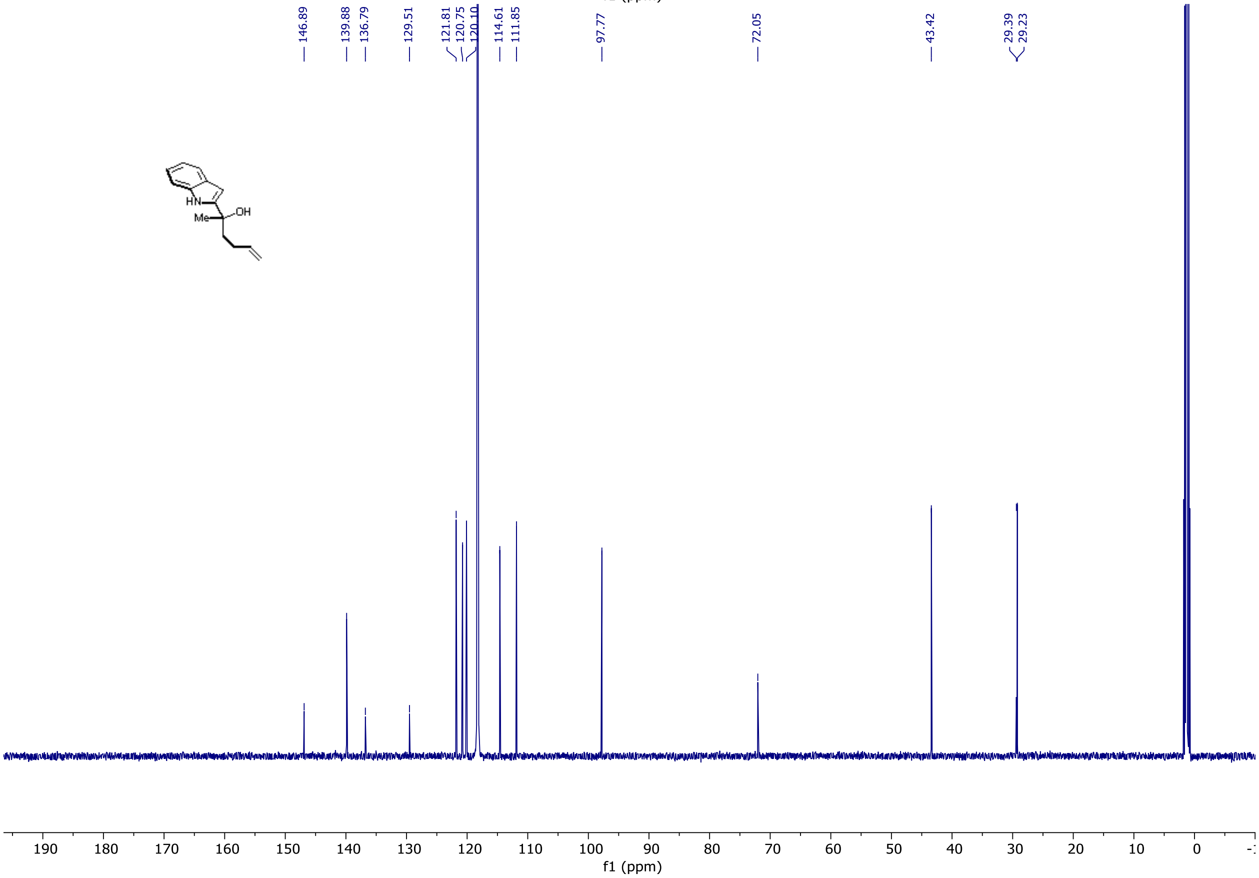

NMR spectra for compound **1o**: $^{1}$H (501 MHz) and $^{13}$C (126 MHz), in acetonitrile-d3.

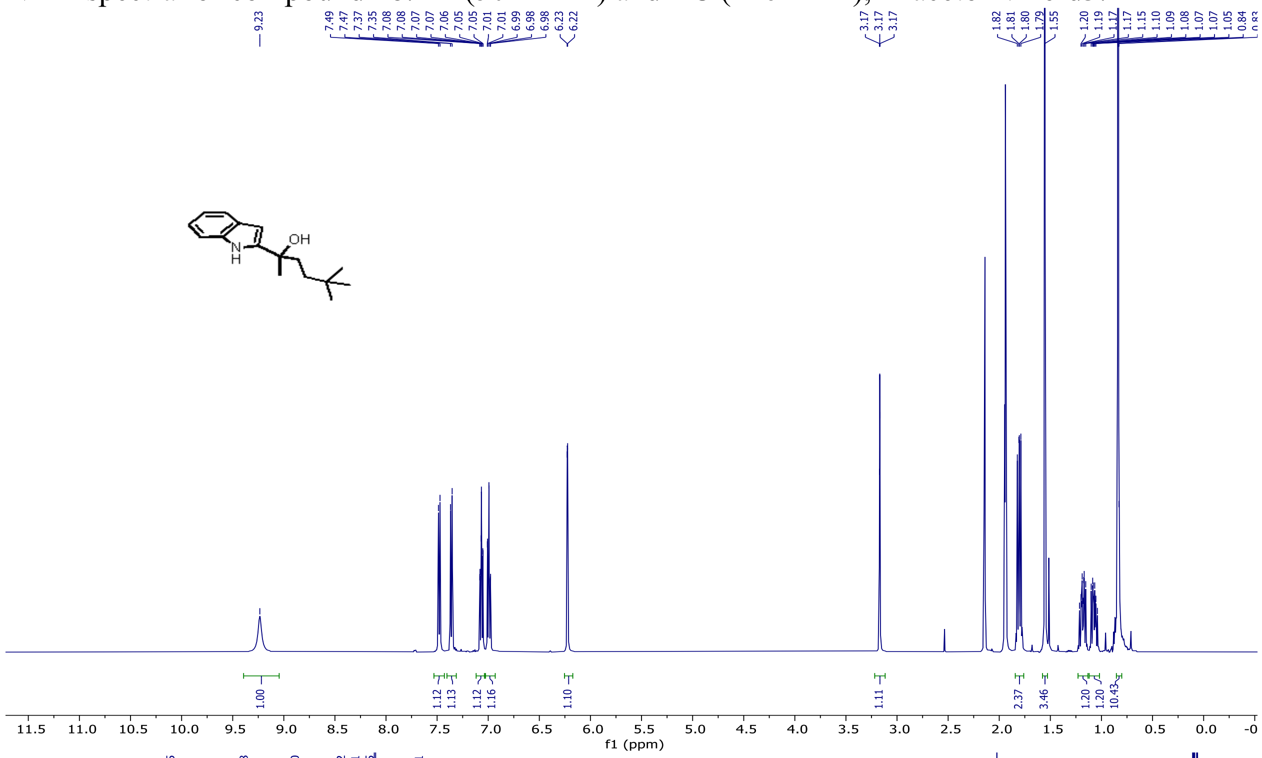

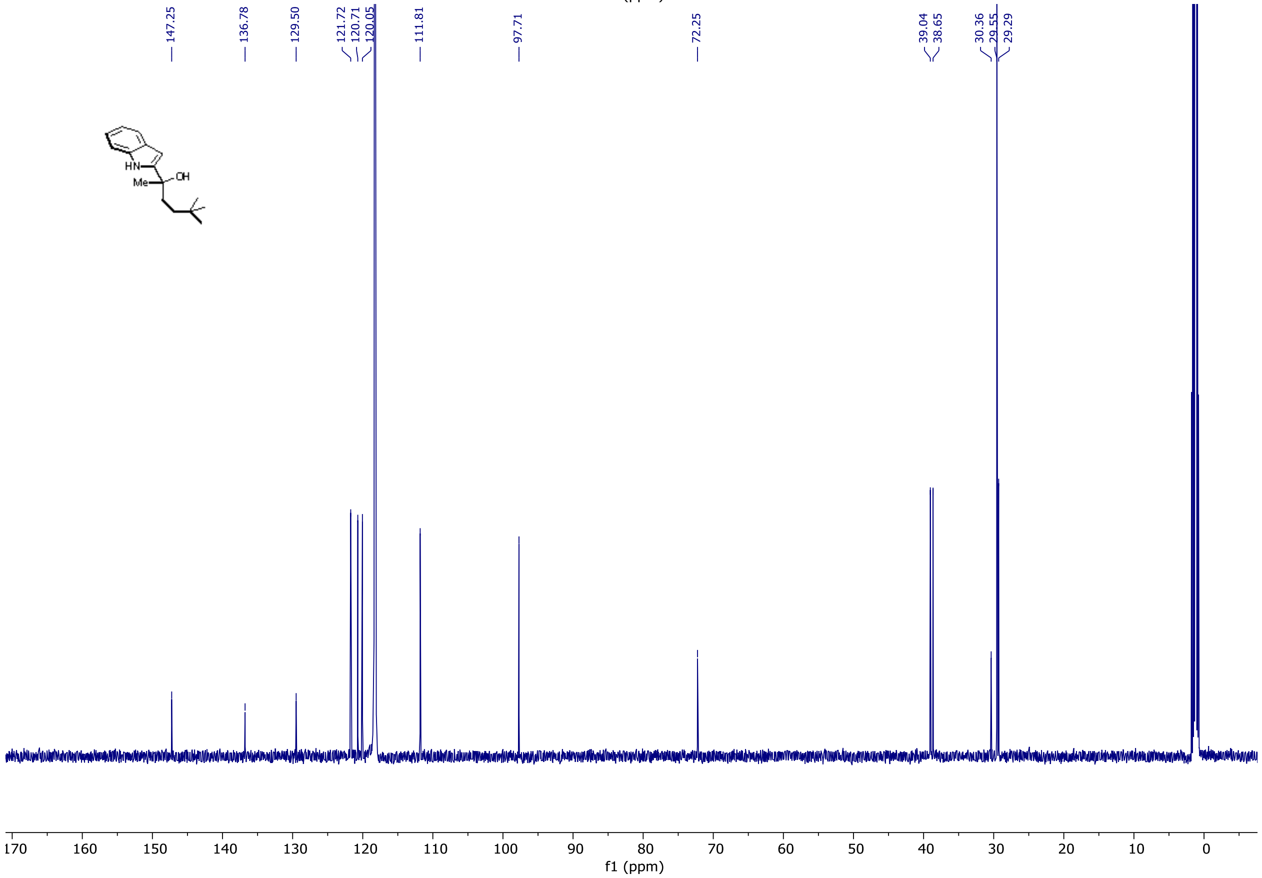

NMR spectra for compound **1p**: $^{1}H$ (501 MHz) and $^{13}C$ (126 MHz) in acetonitrile-d3.

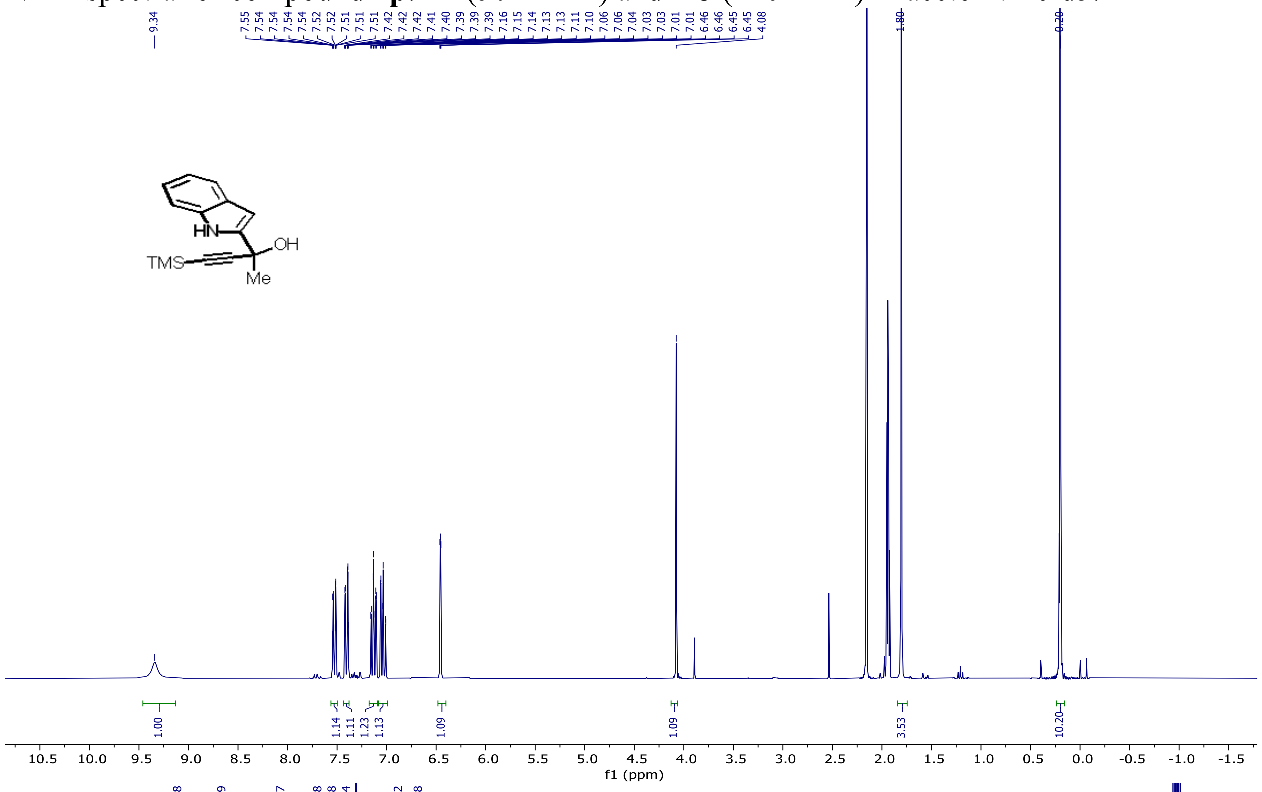


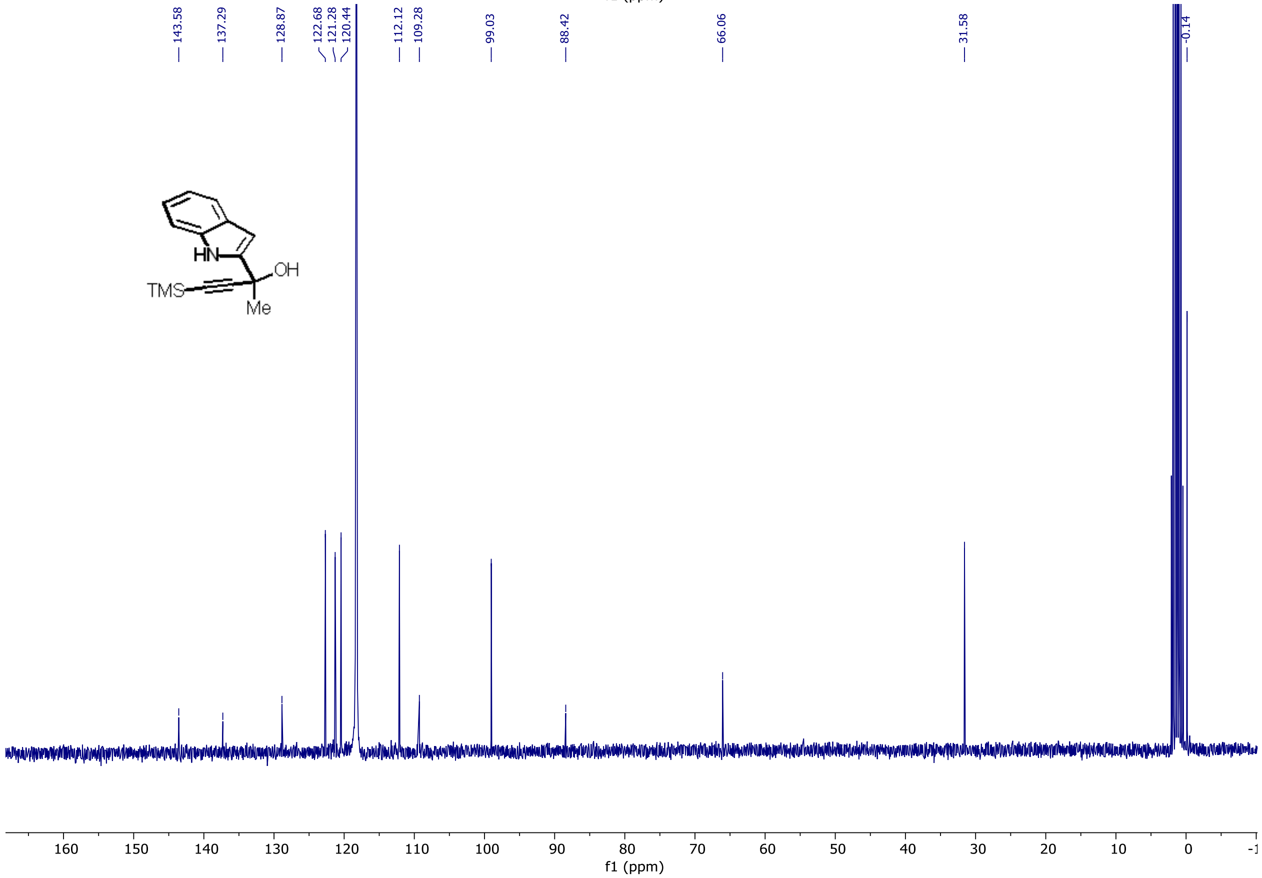

NMR spectra for compound **1q**: $^{1}$H (501 MHz) and $^{13}$C (126 MHz) in acetonitrile-d3.

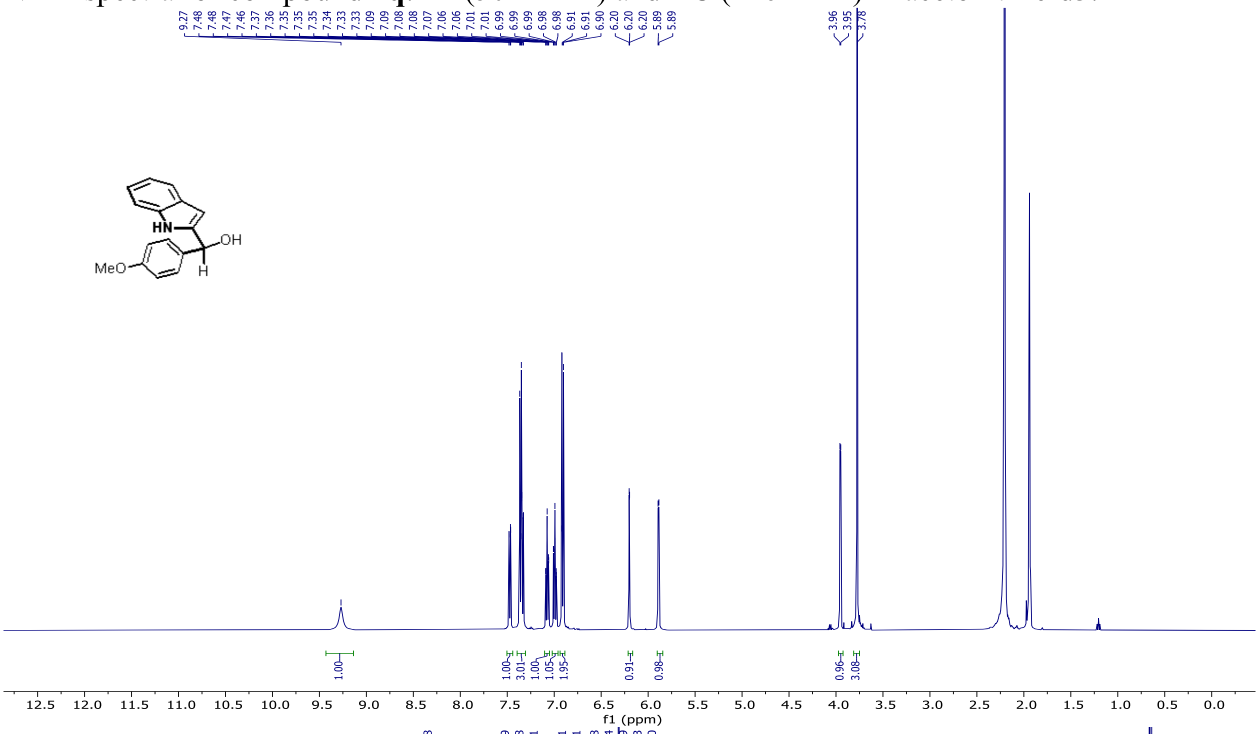

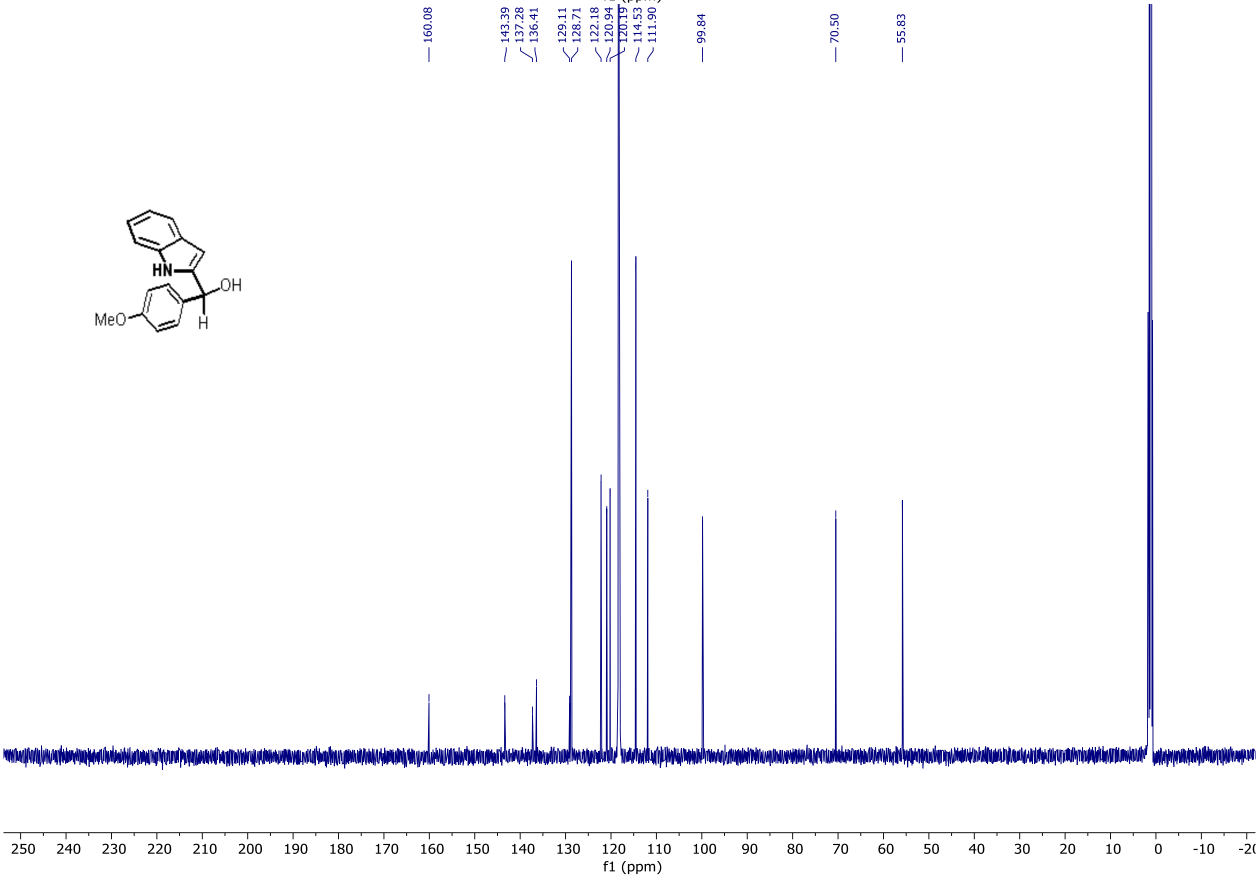

NMR spectra for compound **4a**: $^{1}H$ (501 MHz) and $^{13}C$ (126 MHz) in $CD_2Cl_2$.

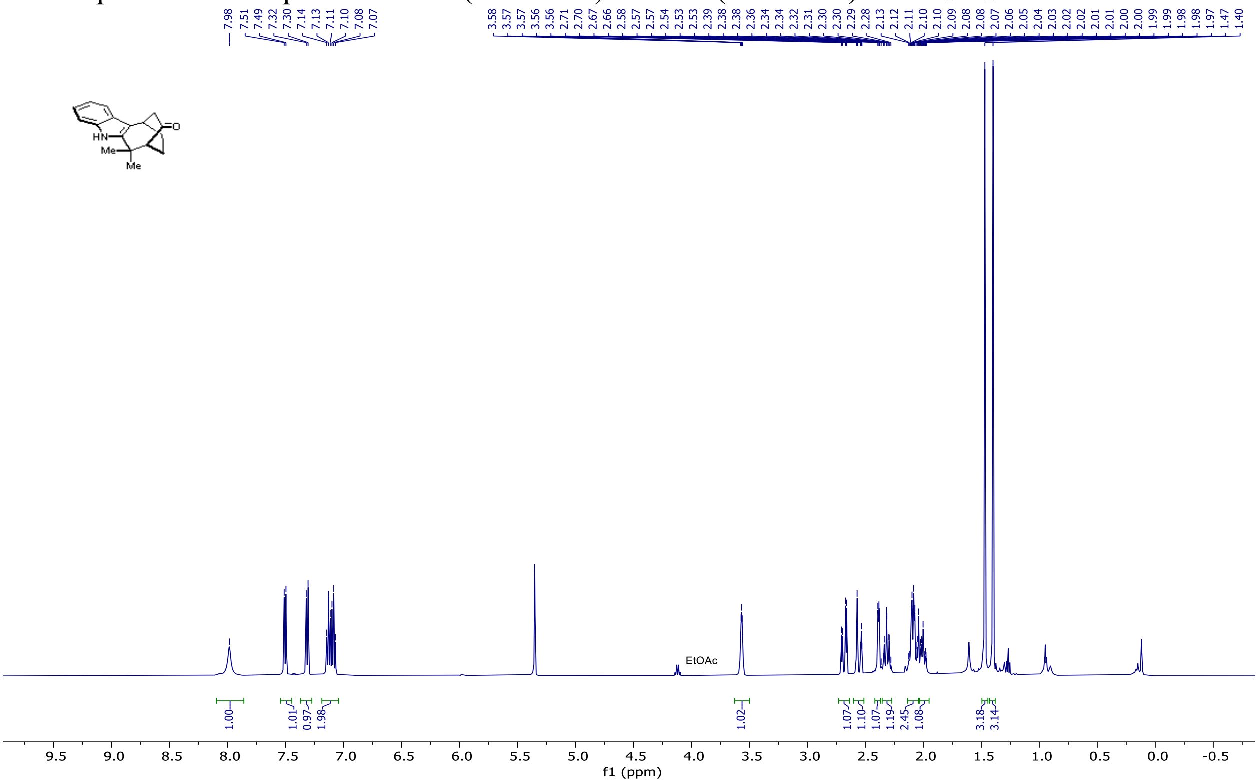

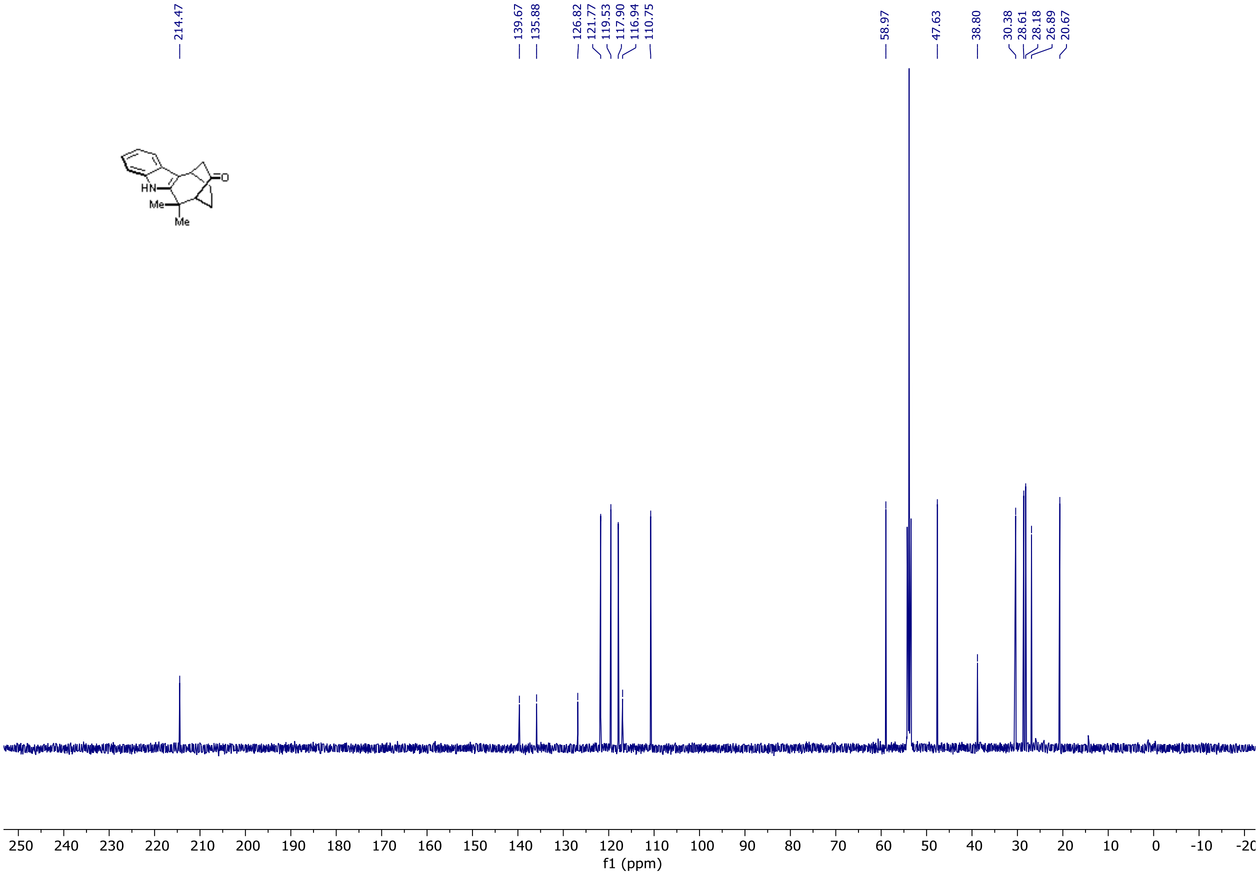

NMR spectra for compound **4b**: $^{1}H$ (501 MHz) and $^{13}C$ (126 MHz) in $CD_2Cl_2$.

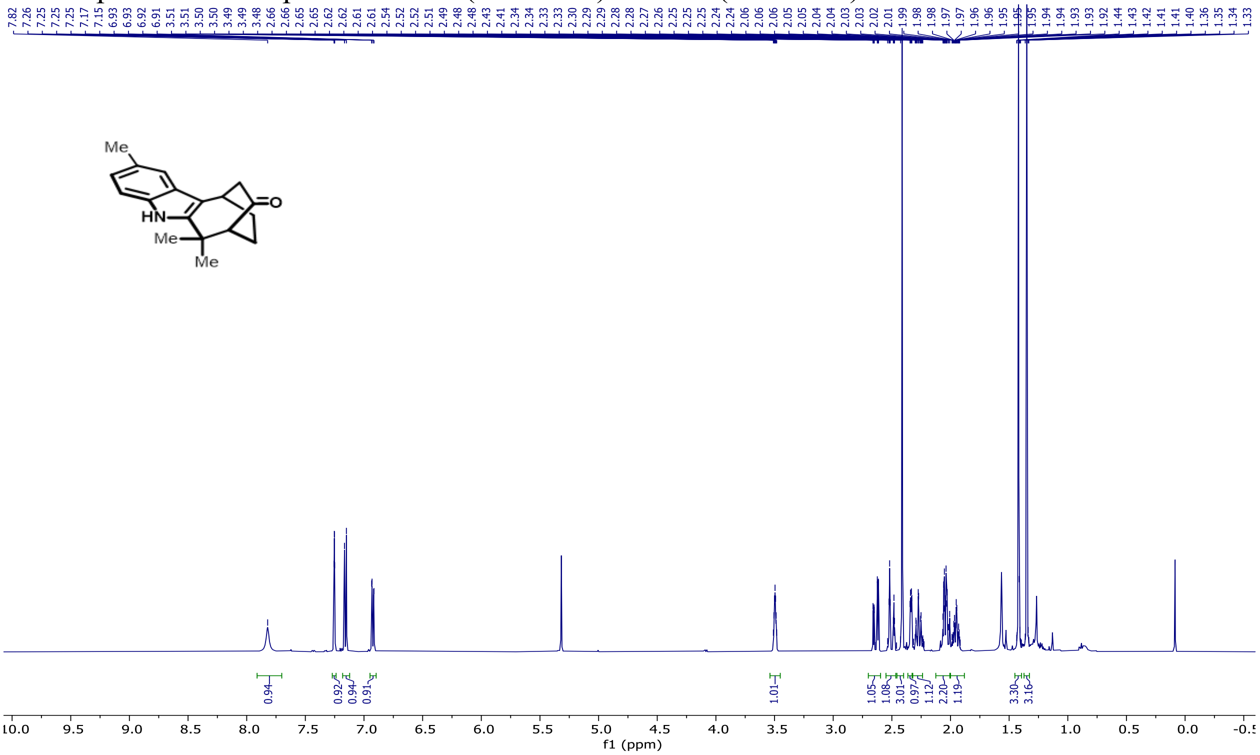


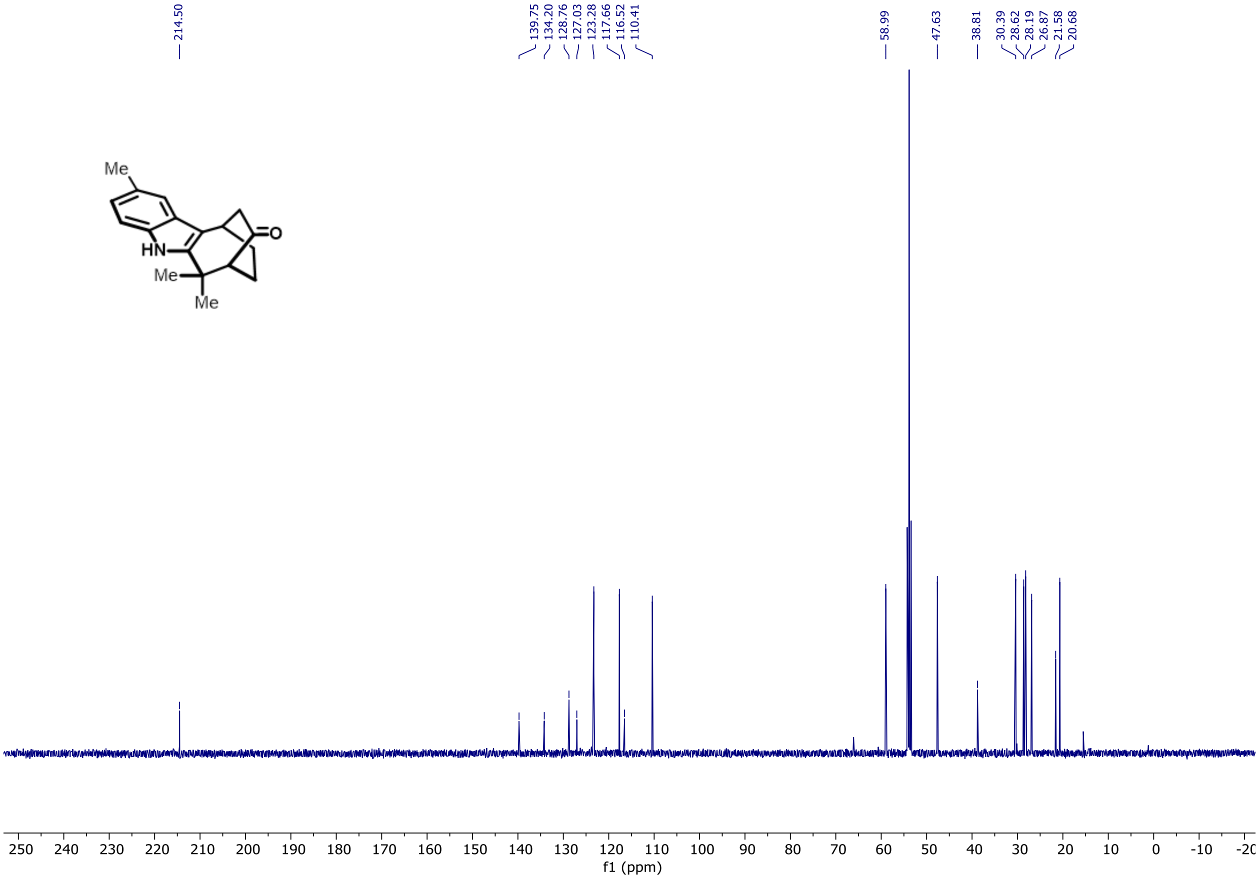

NMR spectra for compound **4c**: $^{1}$H (501 MHz) and $^{13}$C (126 MHz) in $CD_2Cl_2$.

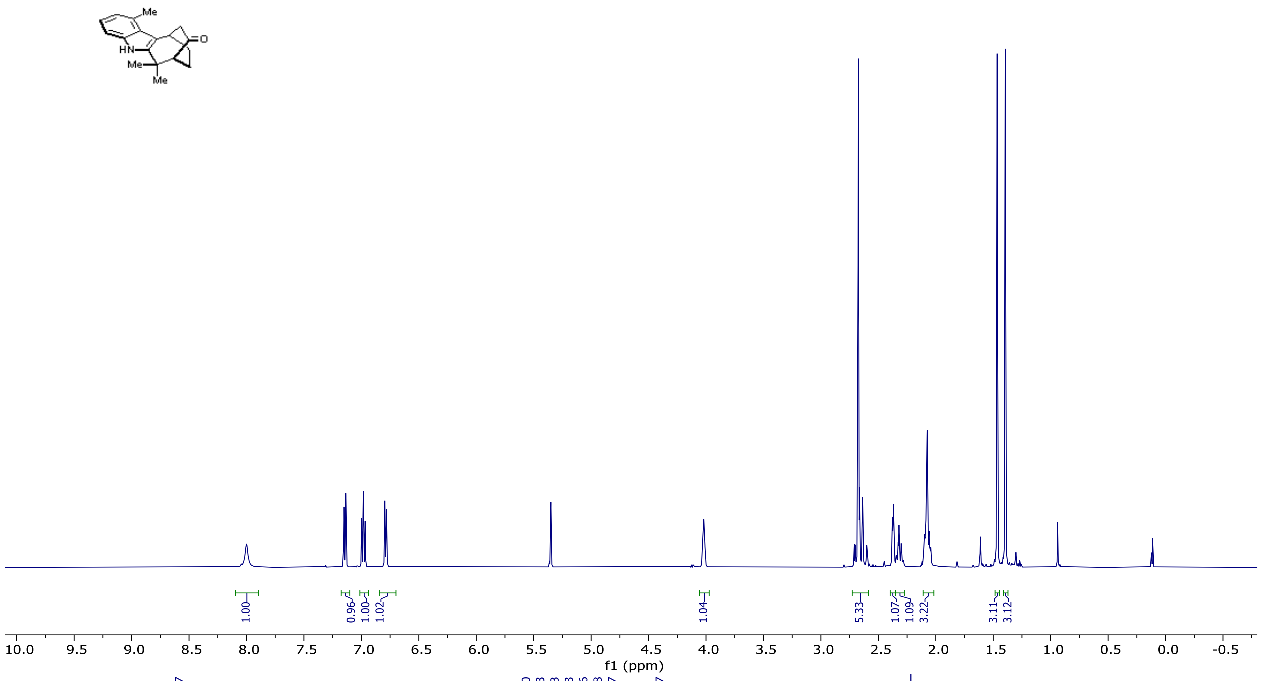


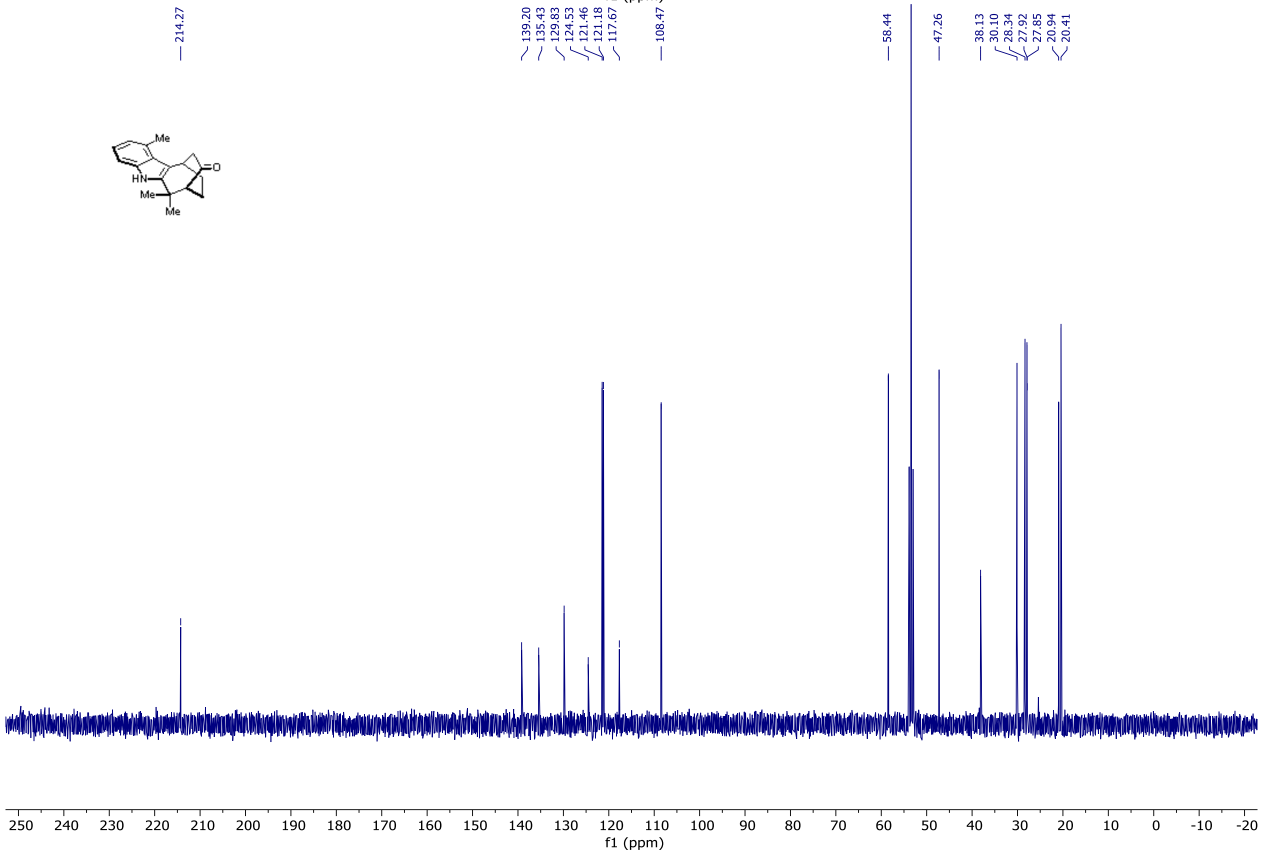

NMR spectra for compound **4d**: $^{1}H$ (501 MHz) and $^{13}C$ (126 MHz) in $CD_2Cl_2$.

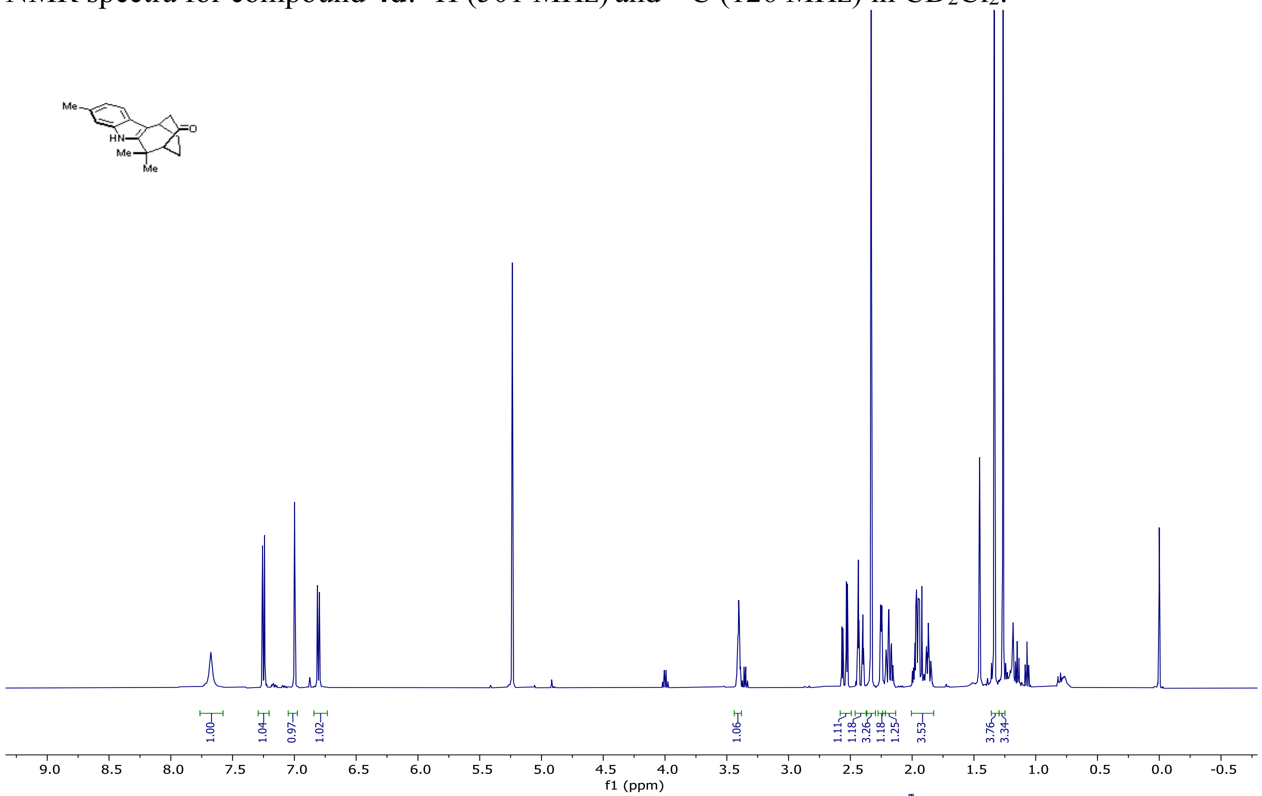


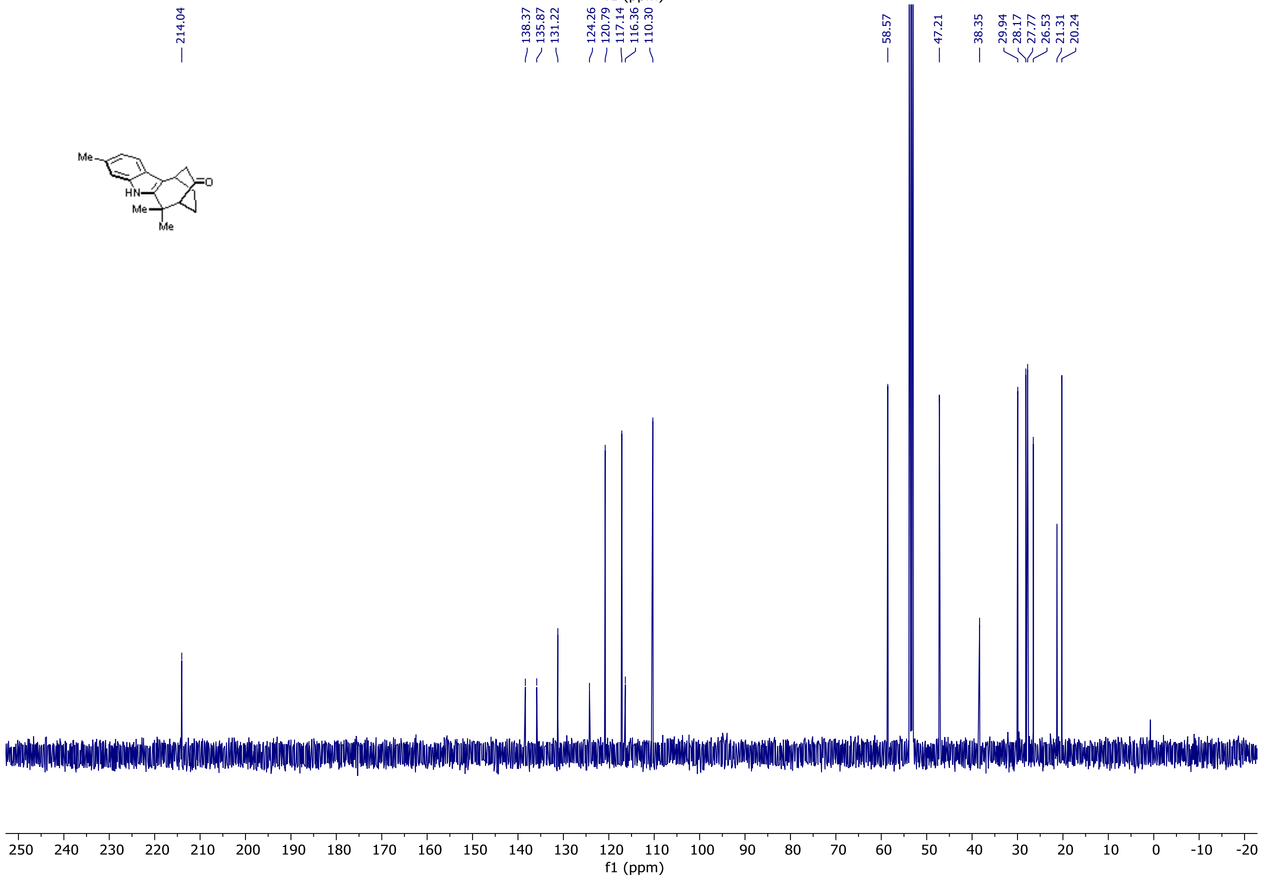

NMR spectra for compound **4e**: $^{1}H$ (501 MHz) and $^{13}C$ (126 MHz) in $CD_2Cl_2$.

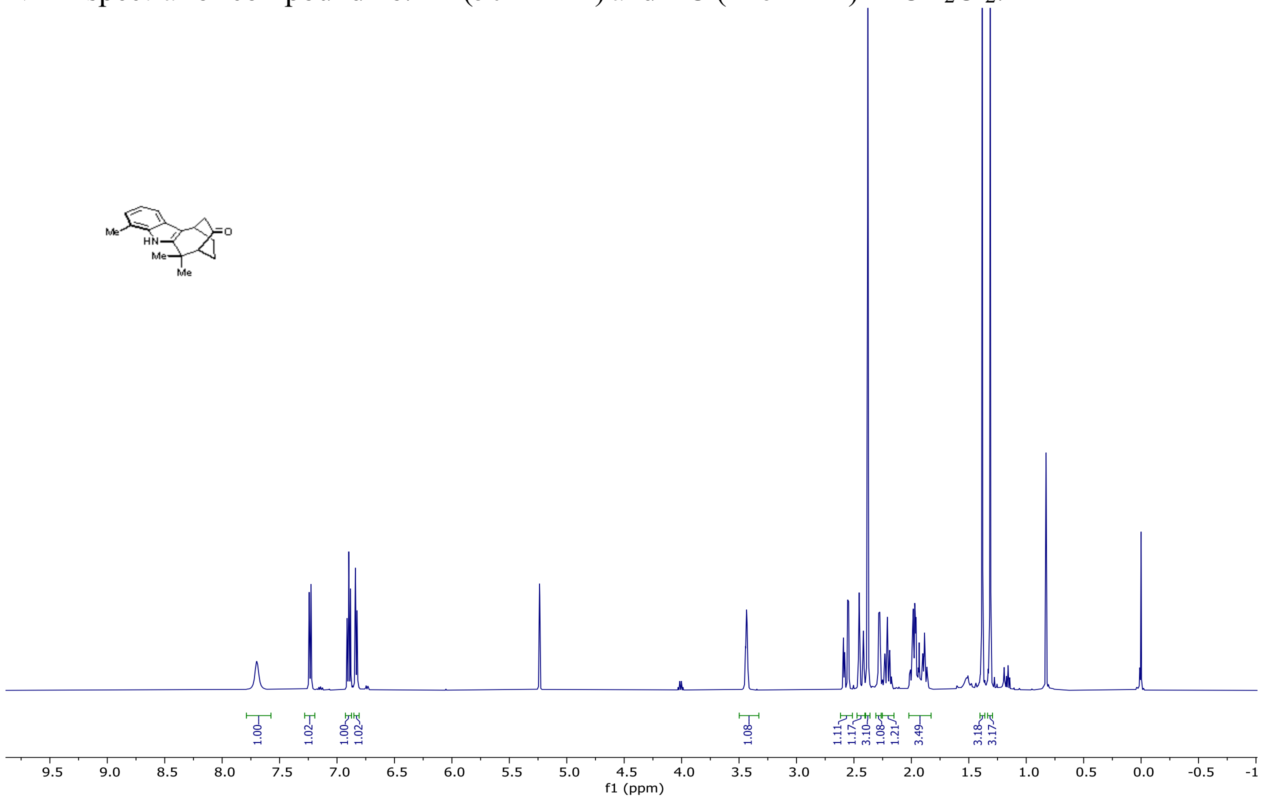


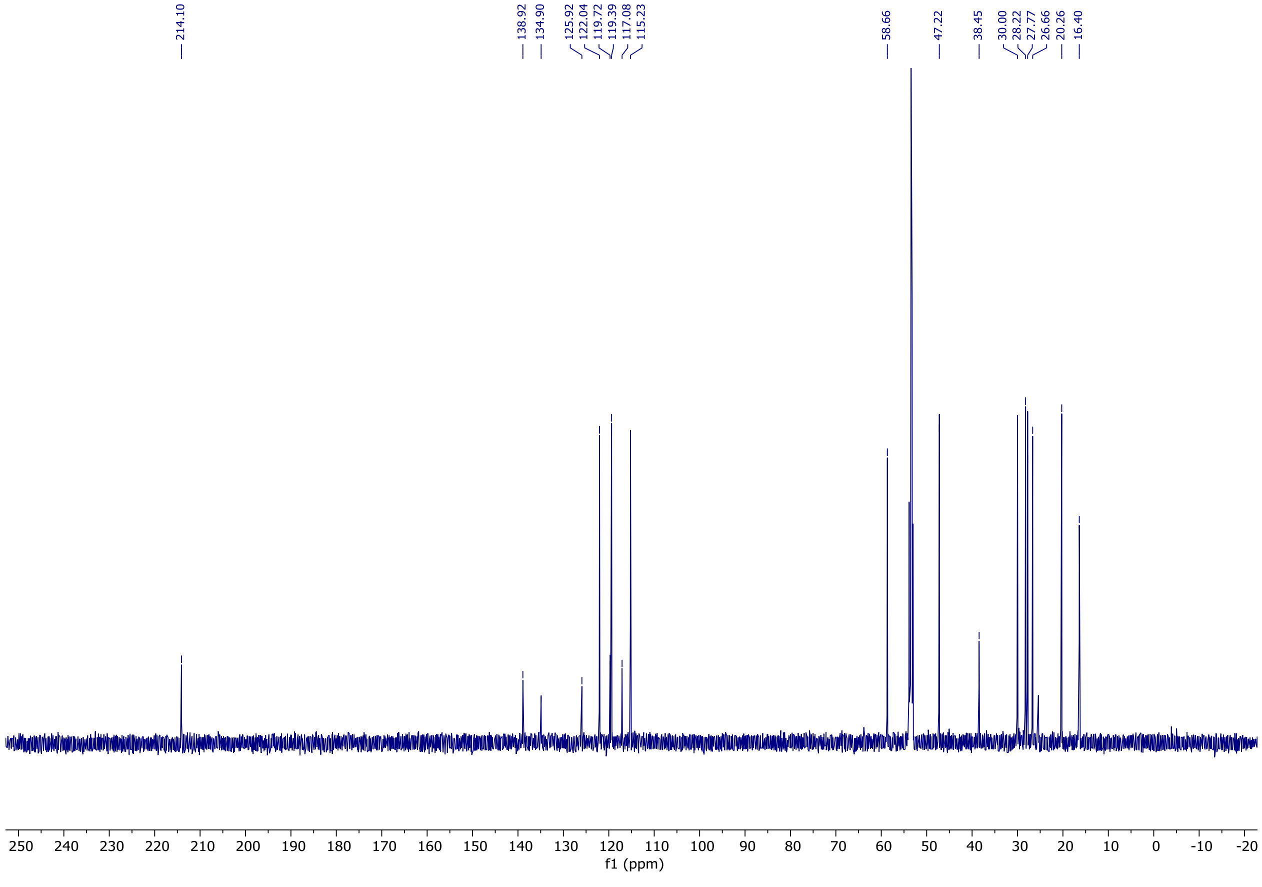

NMR spectra for compound **4f**: $^{1}$H (501 MHz) and $^{13}$C (126 MHz) in $CD_2Cl_2$.

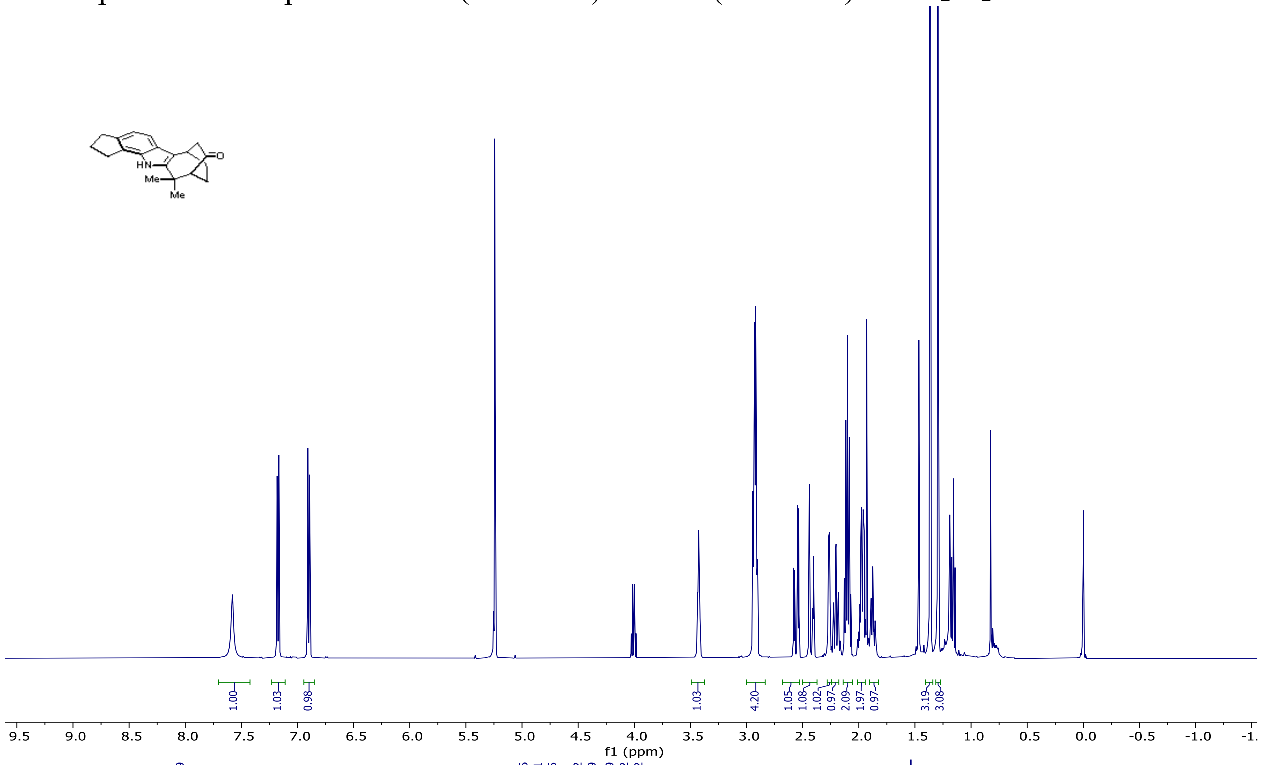


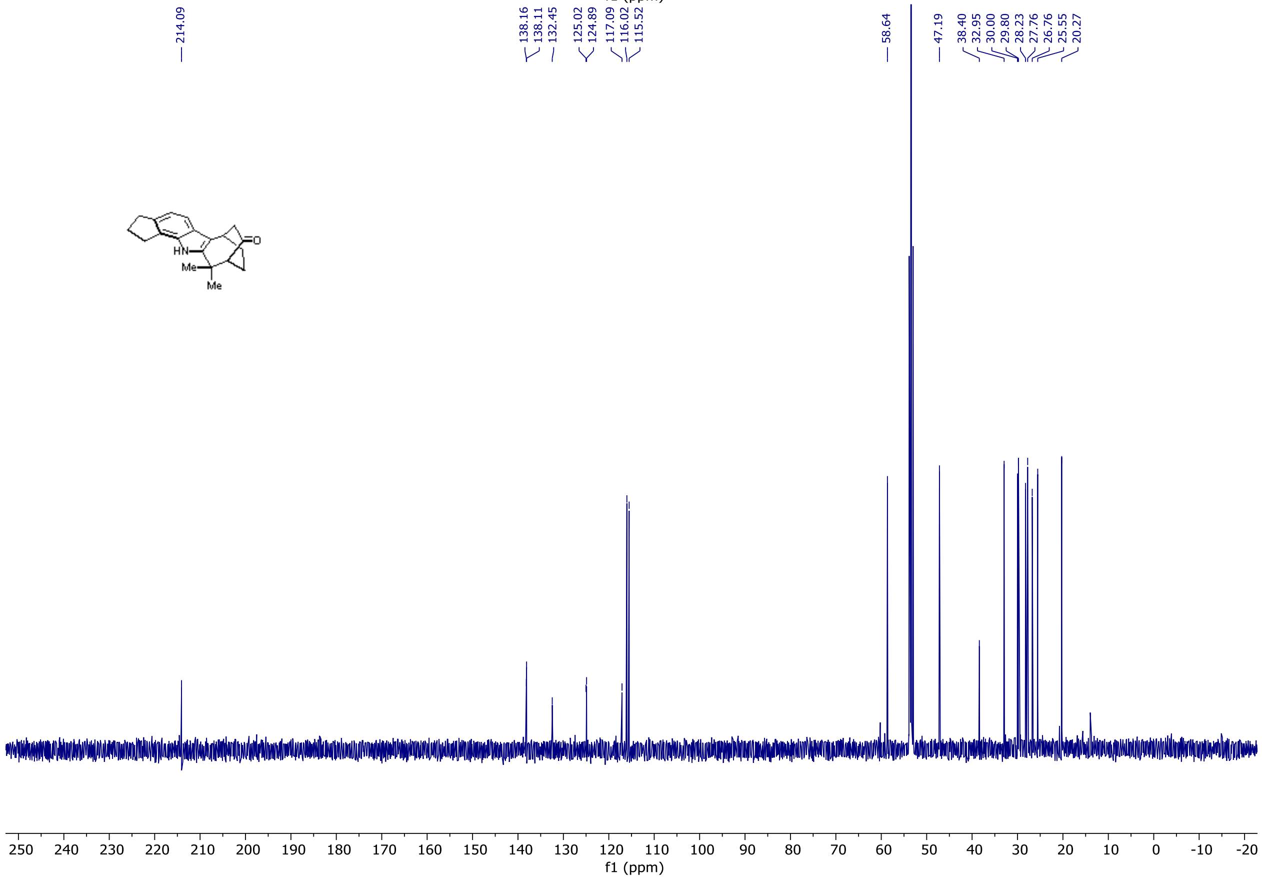

NMR spectra for compound **4g**: $^{1}$H (501 MHz) and $^{13}$C (126 MHz) in $CD_2Cl_2$.

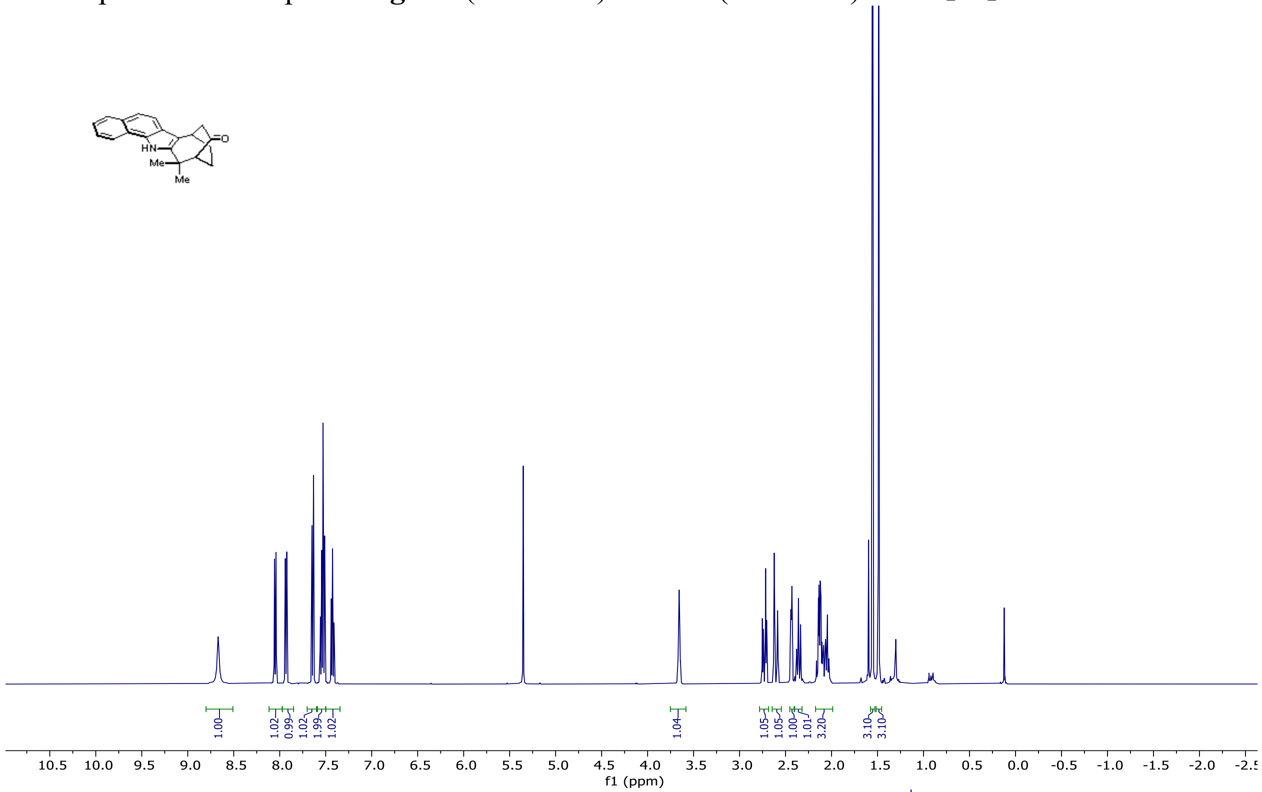


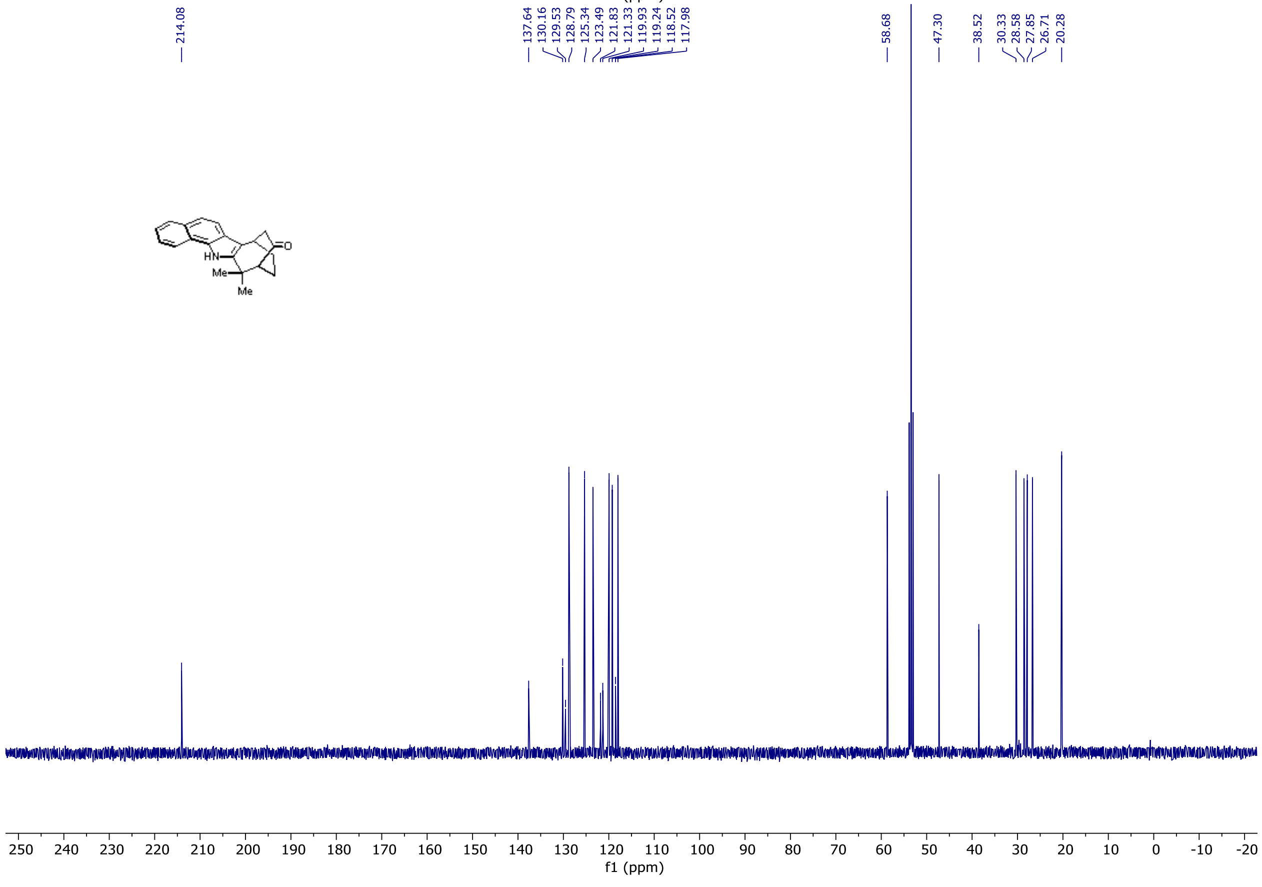

NMR spectra for compound **4h**: $^{1}H$ (501 MHz) and $^{13}C$ (126 MHz) in $CD_2Cl_2$.

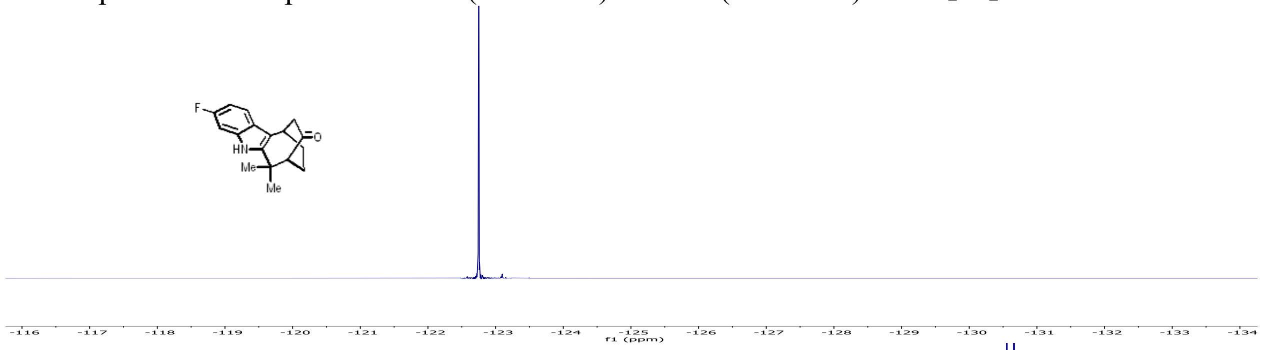


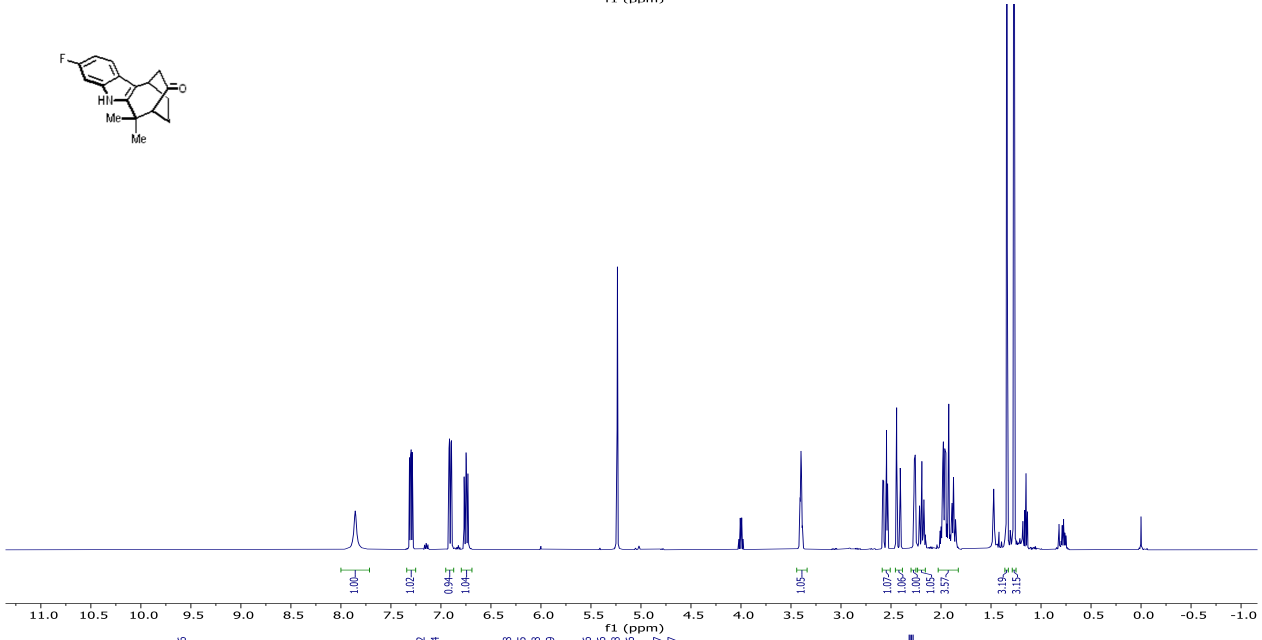


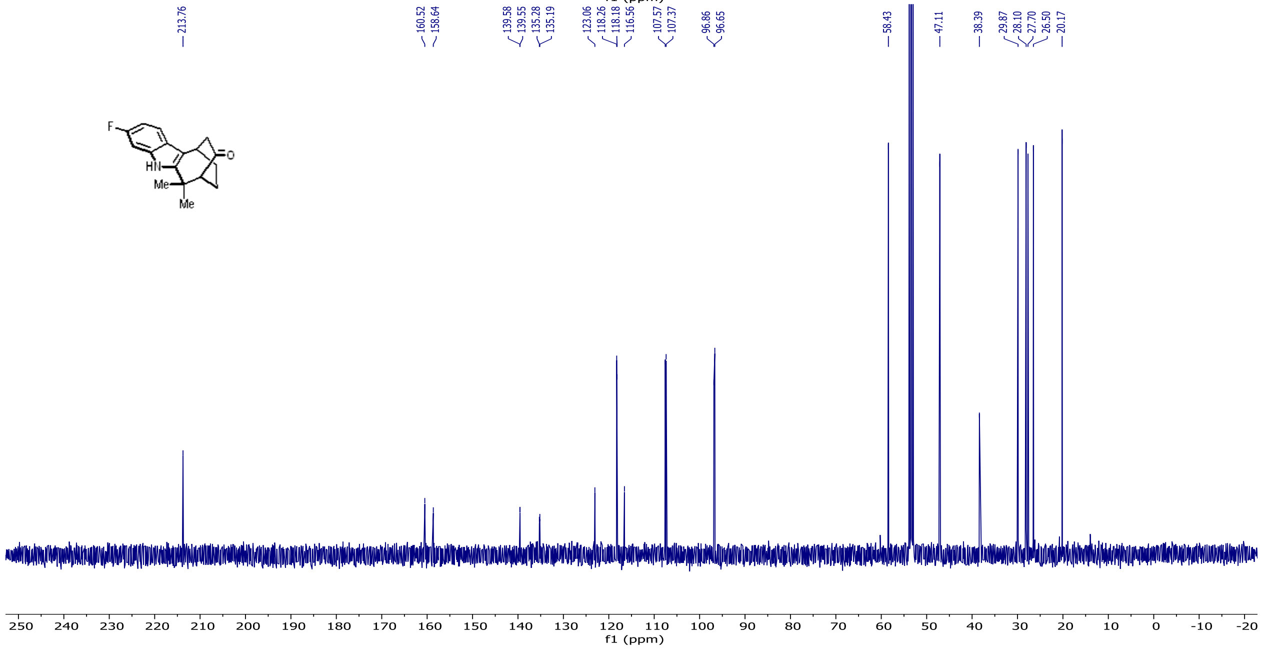

NMR spectra for compound **4i**: $^{1}H$ (501 MHz) and $^{13}C$ (126 MHz) in $CD_2Cl_2$.

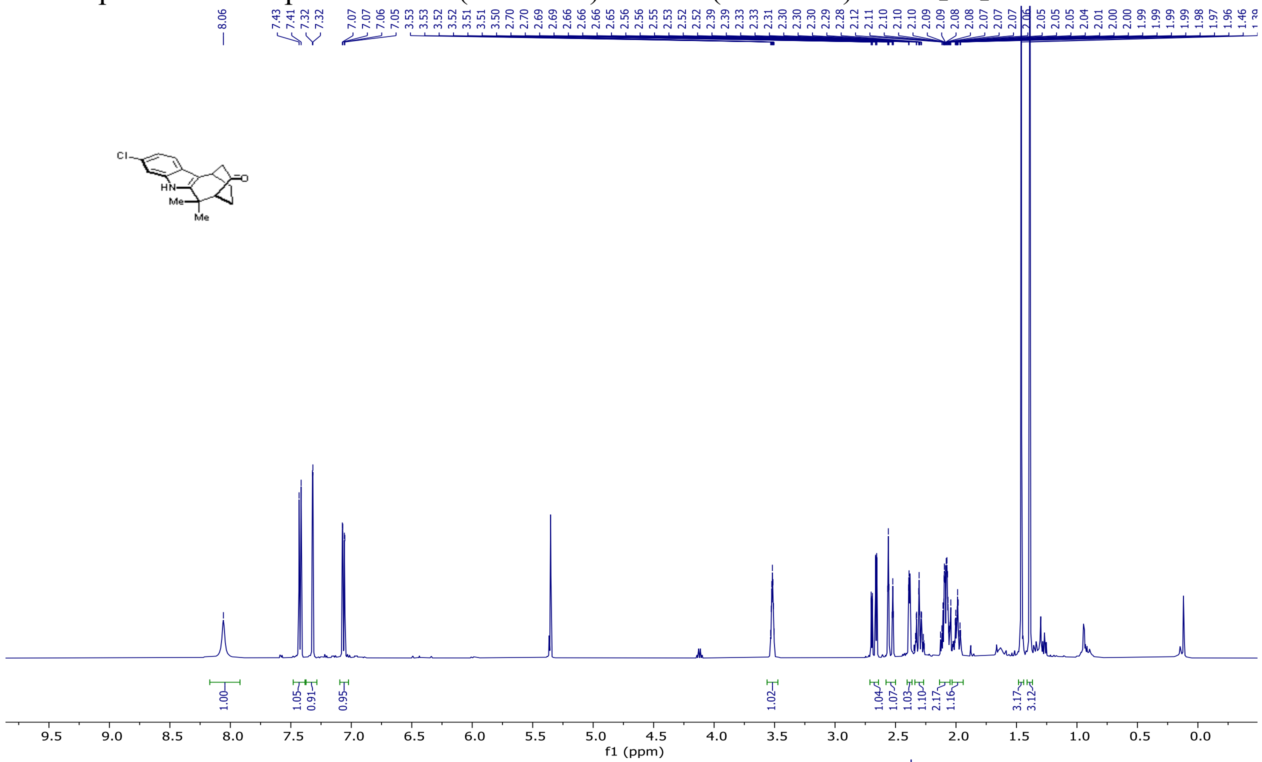


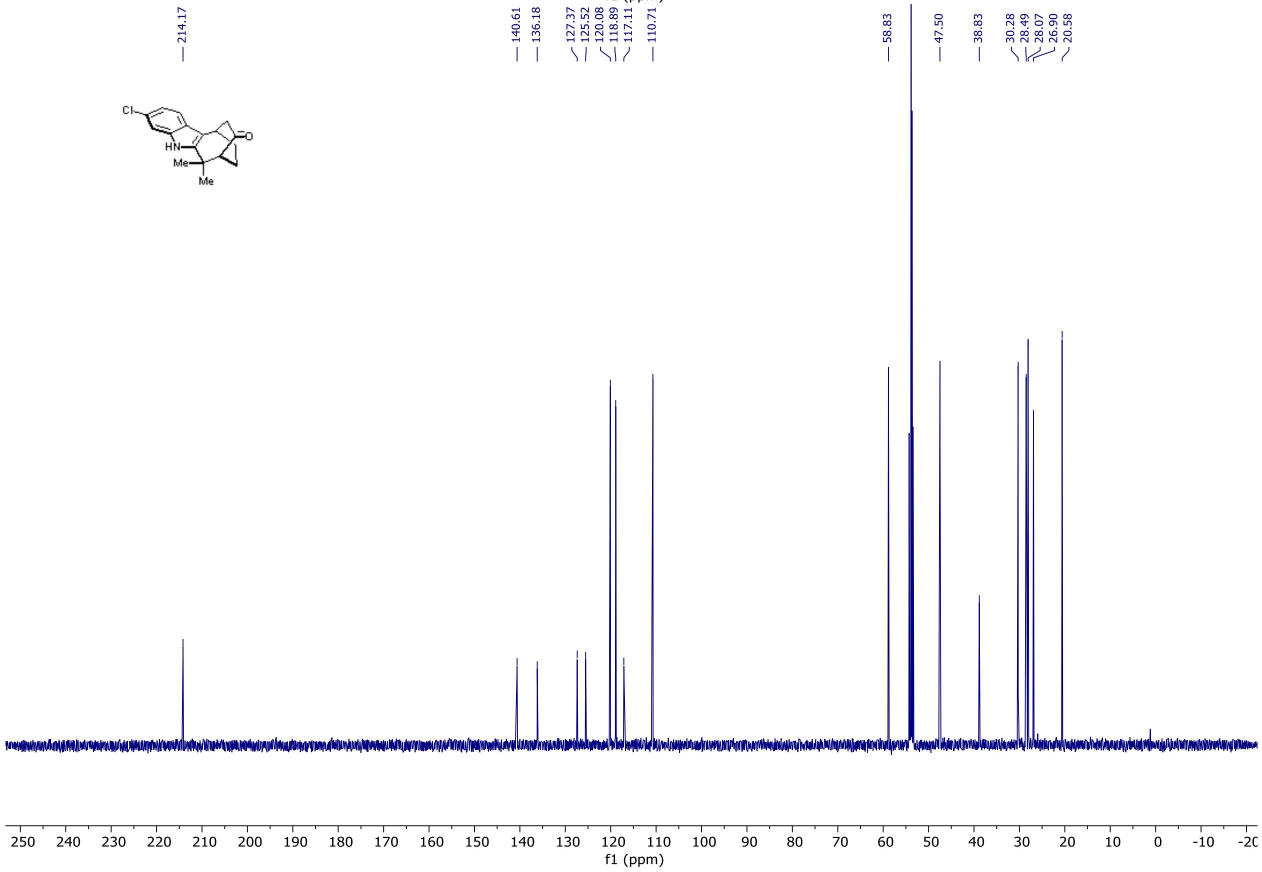

NMR spectra for compound **4j**: $^{1}$H (501 MHz) and $^{13}$C (126 MHz) in $CD_2Cl_2$.

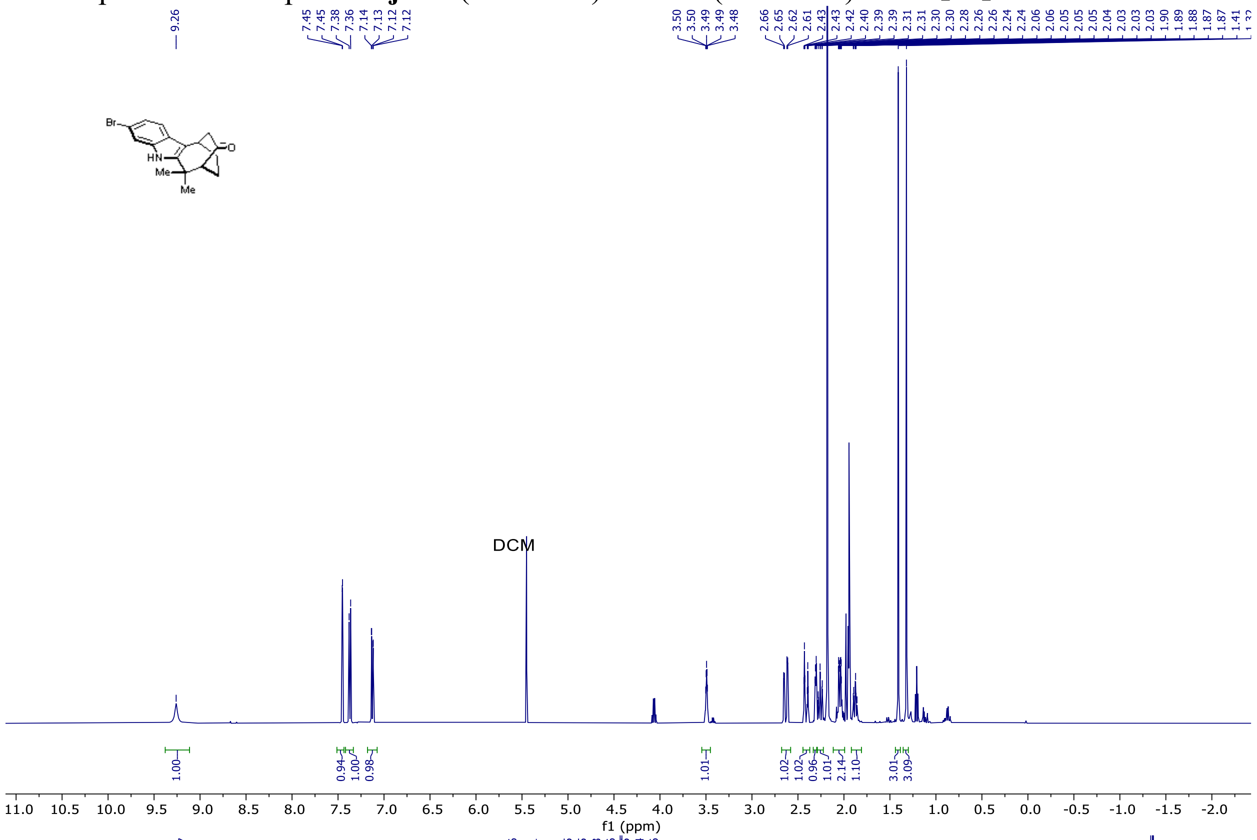


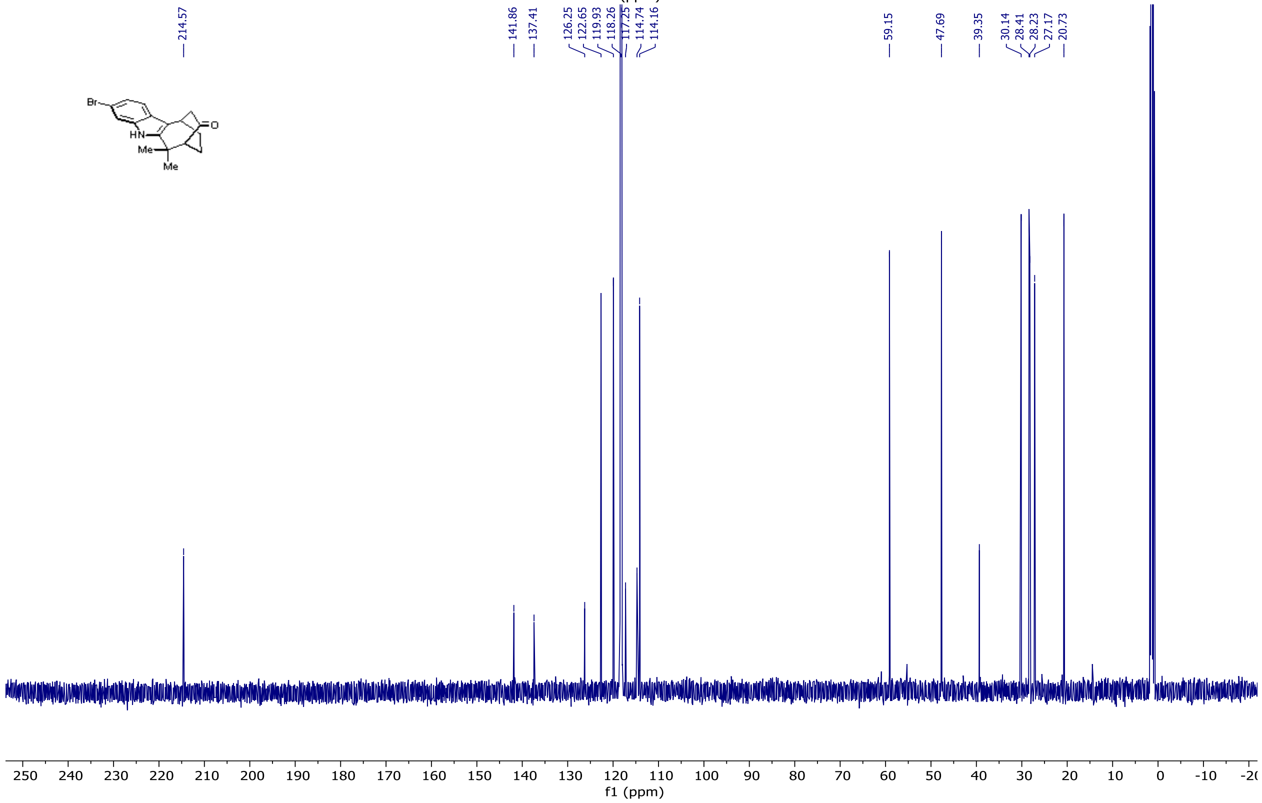

NMR spectra for compound **4k**: $^{1}H$ (501 MHz) and $^{13}C$ (126 MHz) in $CD_2Cl_2$.

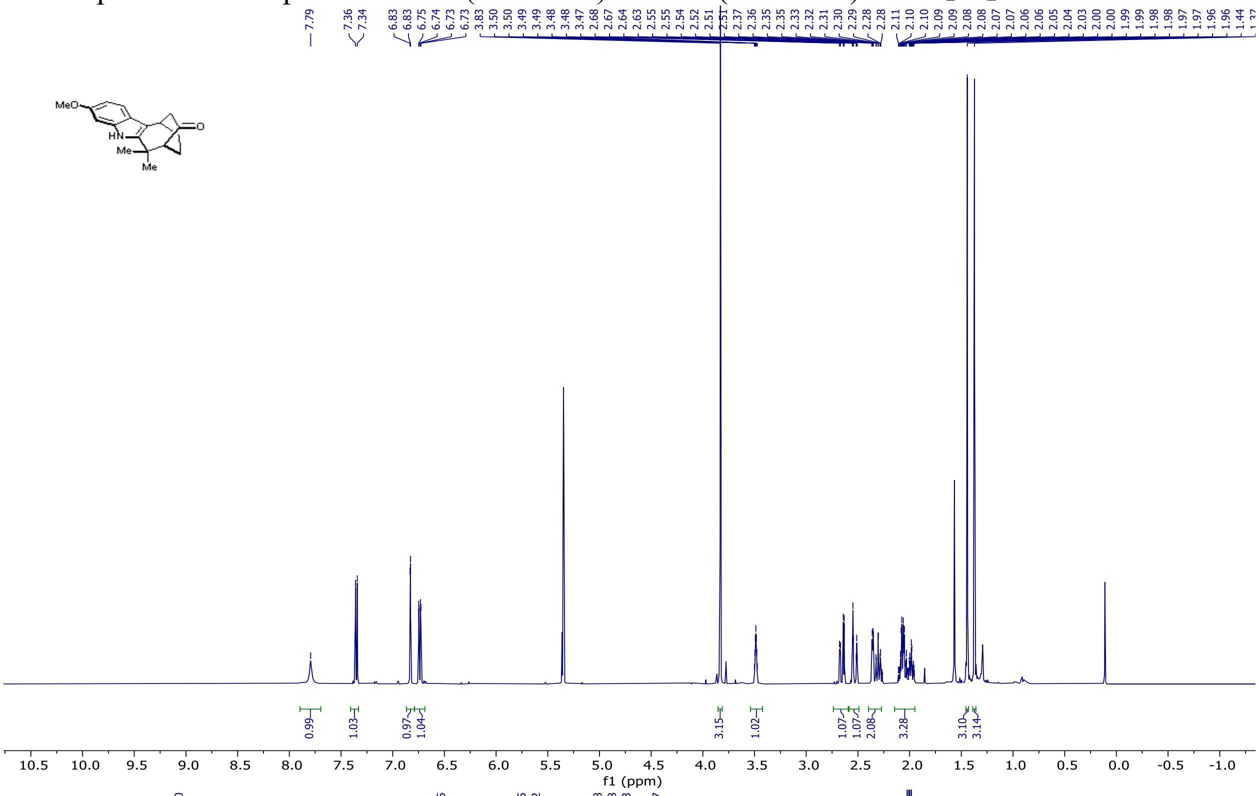

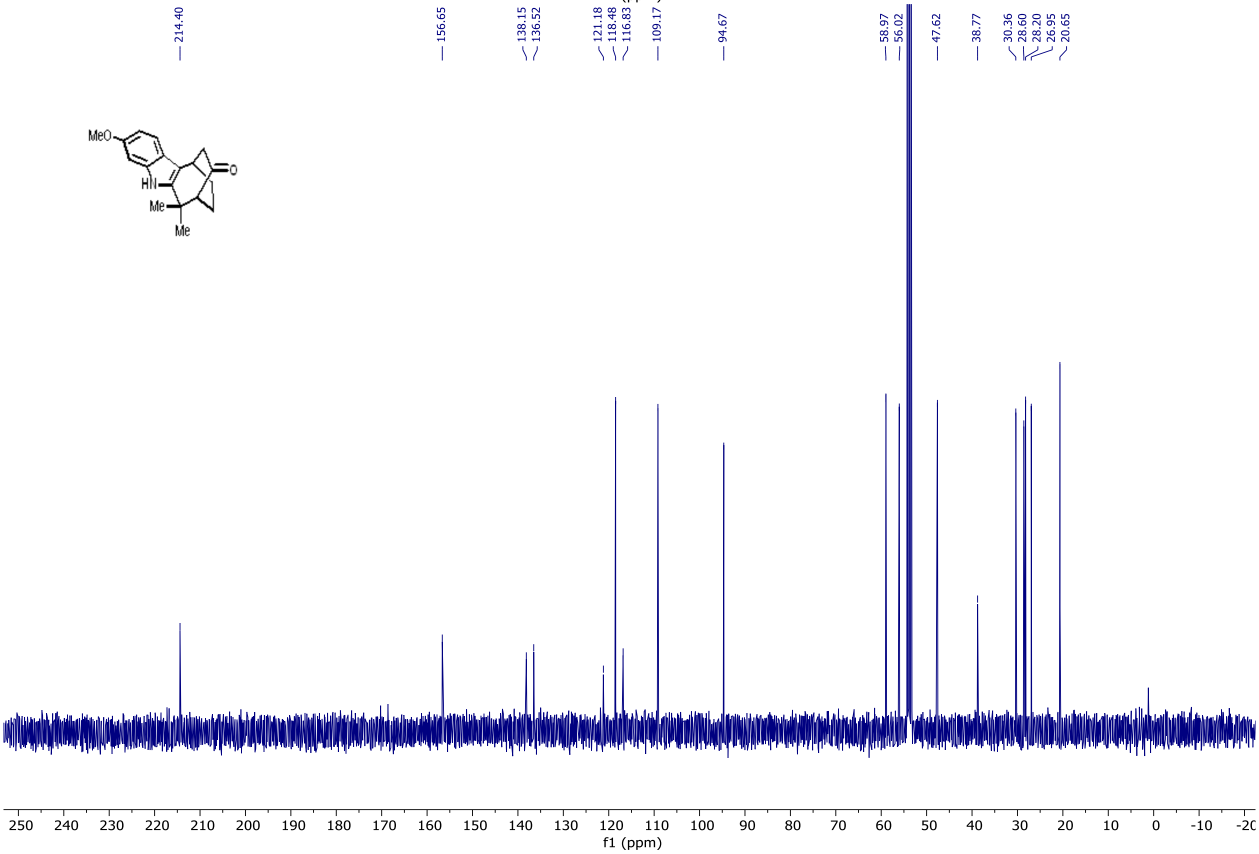

## Characterization of 4l

| Atom | δ (ppm) | J (Hz) | HSQC | HMQC | COSY | NOESY | $^{15}$N-HMBC |
|---|---|---|---|---|---|---|---|
| N1 | -259.2 | $^{1}J_{(1H,15N)}$ = 95 | | | | | 1 |
| H1 | 7.746 | s (br) | | | 6 | 3, 17, 18b | 1 |
| C2 | 135.31 | | | 4, 6 | | | |
| C3 | 110.57 | | 3 | 4, 5 | | | |
| H3 | 7.287 | d 8.0(4), d 1.1(5), d 0.8(6) | 3 | 5, 7 | 4, 5, 6 | 1 | |
| C4 | 121.74 | | 4 | 6 | | | |
| H4 | 7.139 | d 8.0(3), d 7.1(5), d 1.3(6) | 4 | 2, 3, 6 | 3, 5, 6 | | |
| C5 | 119.46 | | 5 | 3 | | | |
| H5 | 7.094 | d 7.8(6), d 7.1(4), d 1.1(3) | 5 | 3, 7 | 3, 4, 6 | 6 | |
| C6 | 117.75 | | 6 | 4 | | | |
| H6 | 7.491 | d 7.8(5), d 1.3(4), t 0.7(3, ?) | 6 | 2, 4, 8 | 1, 3, 4, 5 | 5, 14 | |
| C7 | 126.30 | | | 3, 5 | | | |
| C8 | 117.23 | | | 6, 13endo, 13exo, 15anti | | | |
| C9 | 139.92 | | | 11, 17, 18a, 18b | | | |
| C10 | 41.10 | | | 11, 16anti, 16syn, 17, 18a, 18b | | | |
| C11 | 54.78 | | 11 | 15anti, 16anti, 16syn, 17, 18a, 18b | | | |
| H11 | 2.549 | d 5.3(16anti), d 1.7(16syn) | 11 | 9, 10, 12, 16, 17 | 16anti, 16syn | 16anti, 16syn, 17, 18a, 19a, 19b | |
| C12 | 214.93 | | | 11, 13endo, 13exo, 16syn | | | |
| C13 | 47.22 | | 13endo, 13exo | | | | |
| H13endo | 2.571 | d 18.3(13exo), t 2.5(14, 15syn) | 13 | 8, 12, 14, 15 | 13exo, 14, 15syn | 14, 17 | |
| H13exo | 2.664 | d 18.3(13endo), d 4.1(14), d 0.6(?) | 13 | 8, 12, 14, 15 | 13endo, 14 | 14, 15anti, 16anti | |
| C14 | 26.34 | | 14 | 13endo, 13exo, 15anti, 16syn | | | |
| H14 | 3.563 | m | 14 | | 13endo, 13exo, 15anti | 6, 13endo, 13exo, 15anti, 15syn | |
| C15 | 27.99 | | 15anti, 15syn | 13endo, 13exo, 16anti, 16syn | | | |
| H15anti | 2.107 | m | 15 | 8, 11, 14, 16 | 14, 15syn, 16anti, 16syn | 13exo, 14 | |
| H15syn | 2.029 | m (overlapped) | 15 | | 13endo, 15anti, 16syn | 14, 16syn | |
| C16 | 19.79 | | 16anti, 16syn | 11, 15anti | | | |
| H16anti | 2.016 | m (overlapped) | 16 | 10, 11, 15 | 11, 15anti, 16syn | 11, 13exo, 16syn | |
| H16syn | 2.236 | m | 16 | 10, 11, 12, 14, 15 | 11, 15anti, 15syn, 16anti | 11, 15syn, 16anti, 18a, 19b | |
| C17 | 26.59 | | 17 | 11, 18a, 18b | | | |
| H17 | 1.370 | d 0.5(18a) | 17 | 9, 10, 11, 18 | | 1, 11, 13endo, 18b | |
| C18 | 40.54 | | 18a, 18b | 17 | | | |
| H18a | 1.833 | d 13.8(18b), d 12.2(19a), d 4.2(19b) | 18 | 9, 10, 11, 17, 19, 20 | 18b, 19a, 19b | 11, 16syn, 18b, 20 | |
| H18b | 1.693 | d 13.8(18a), d 11.9(19b), d 4.3(19a) | 18 | 9, 10, 11, 17, 19, 20 | 18a, 19a, 19b | 1, 17, 18a, 19a | |
| C19 | 26.10 | | 19a, 19b | 18a, 18b, 20, 21 | | | |
| H19a | 1.414 | m | 19 | 20, 21 | 18a, 18b, 19b, 20 | 11, 18b | |
| H19b | 1.312 | m (overlapped) | 19 | 20, 21 | 18a, 18b, 19a | 11, 16syn | |
| C20 | 23.44 | | 20 | 18a, 18b, 19a, 19b, 21 | | | |
| H20 | 1.339 | m (overlapped) | 20 | 19, 21 | 19a, 21 | 18a, 21 | |
| C21 | 14.19 | | 21 | 19a, 19b, 20 | | | |
| H21 | 0.939 | t 7.1(20) | 21 | 19, 20 | 20 | 20 | |

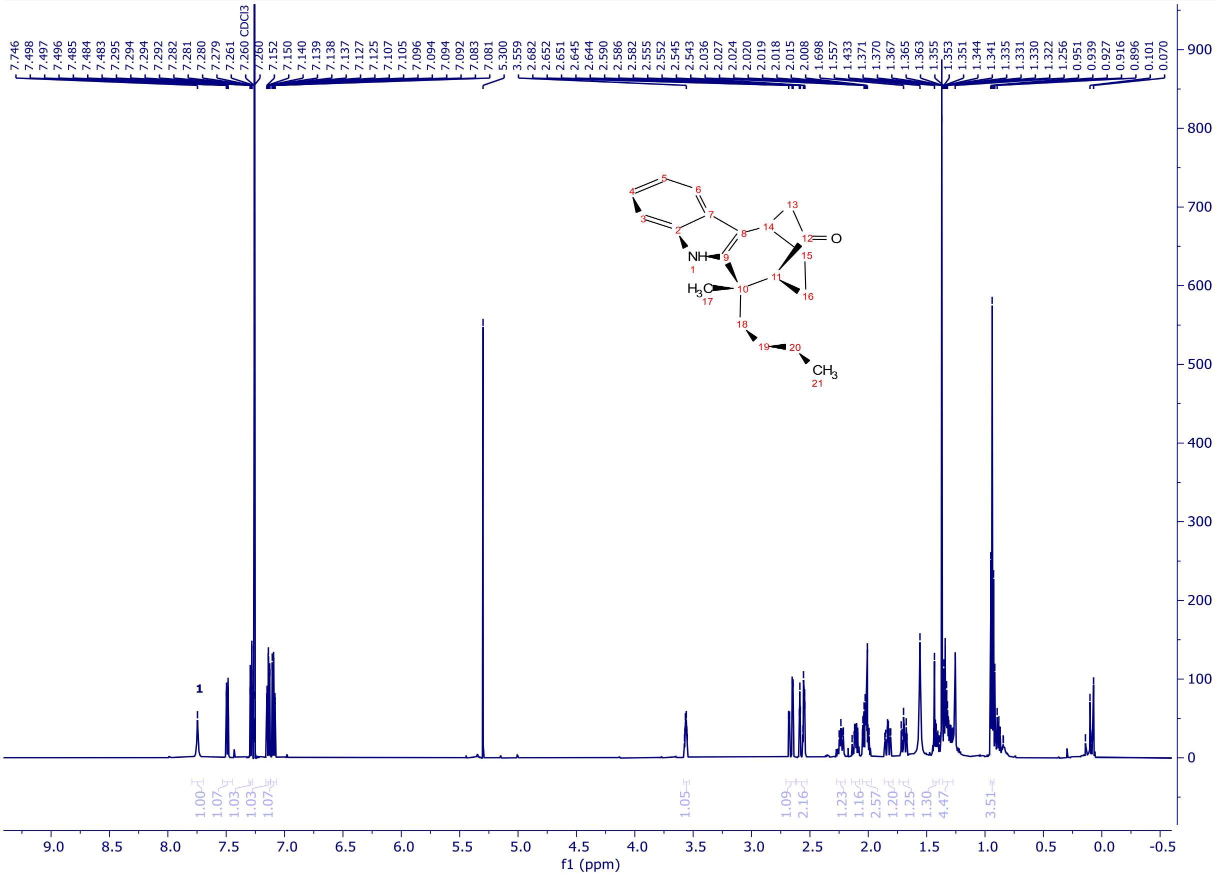

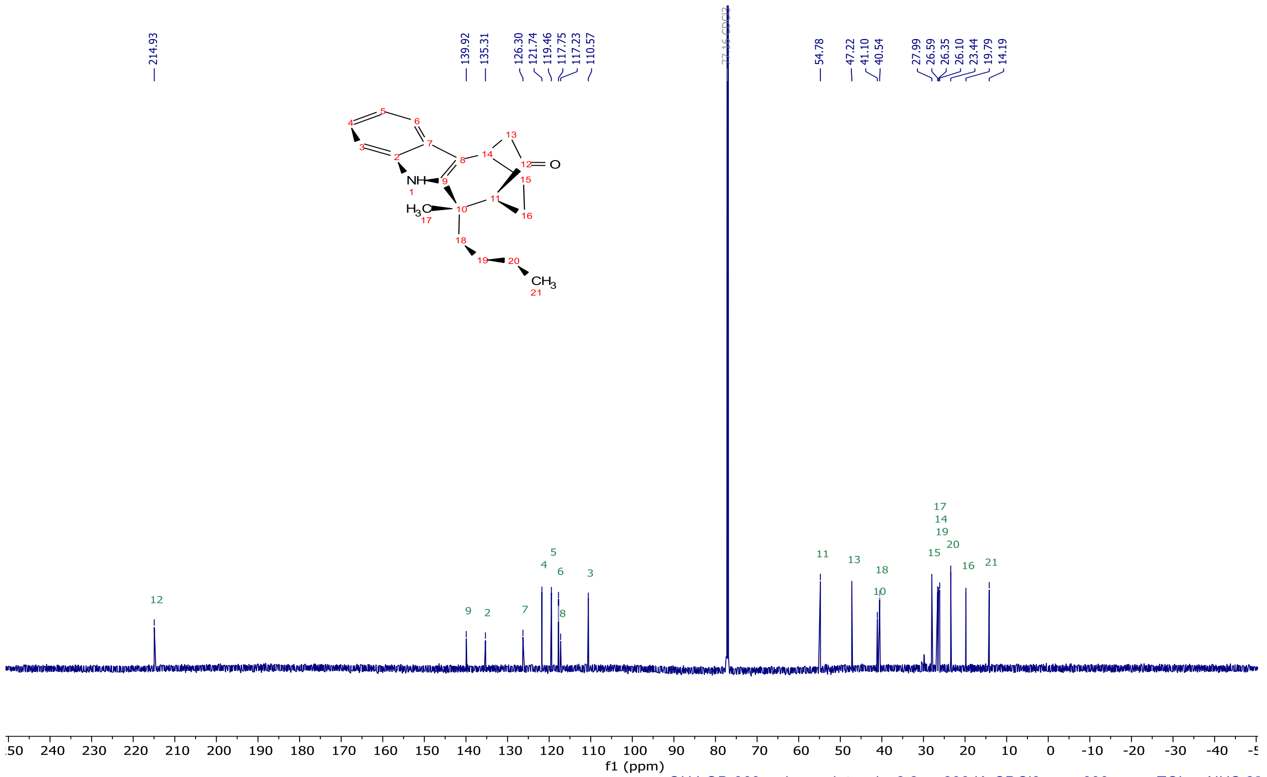

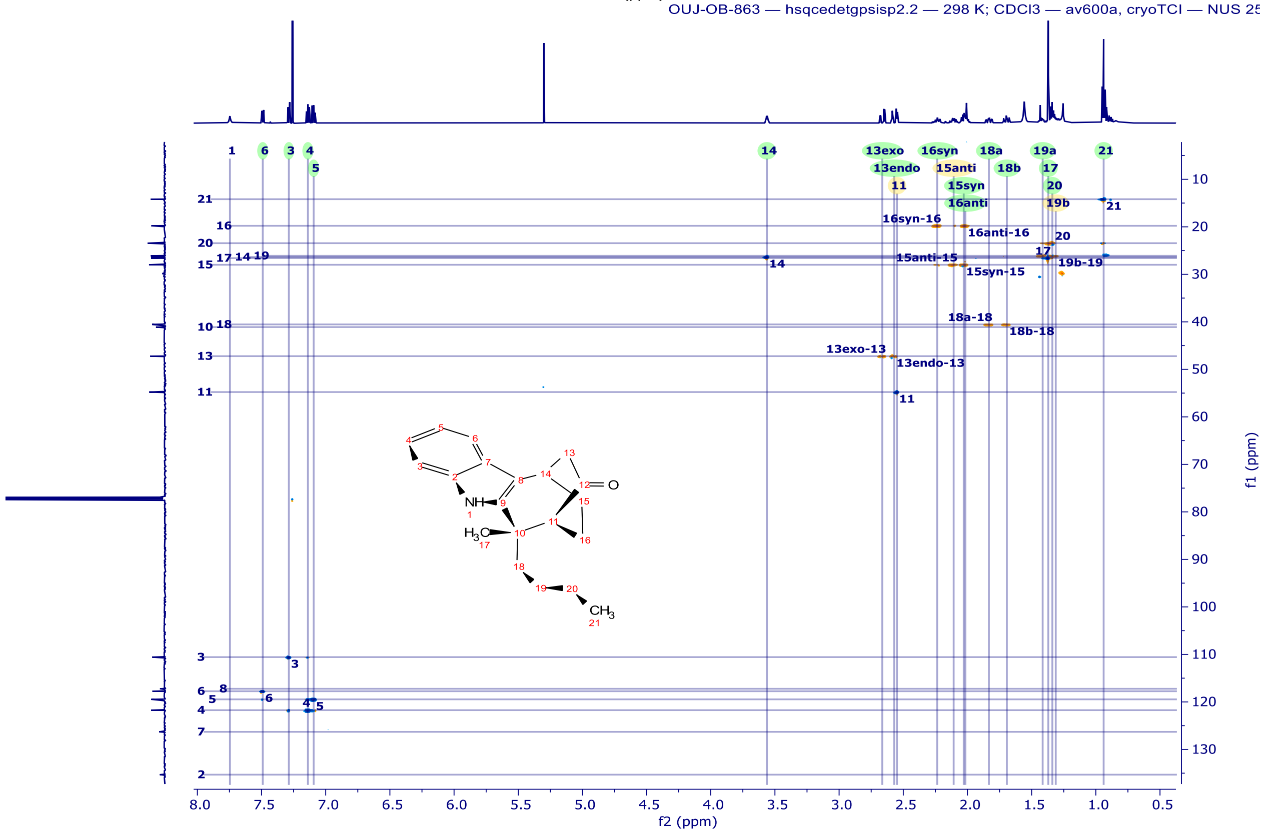

OUJ-OB-863 — hmqcgpqf — 298K; CDCl3 — av600a; cryoTCI — NUS 25

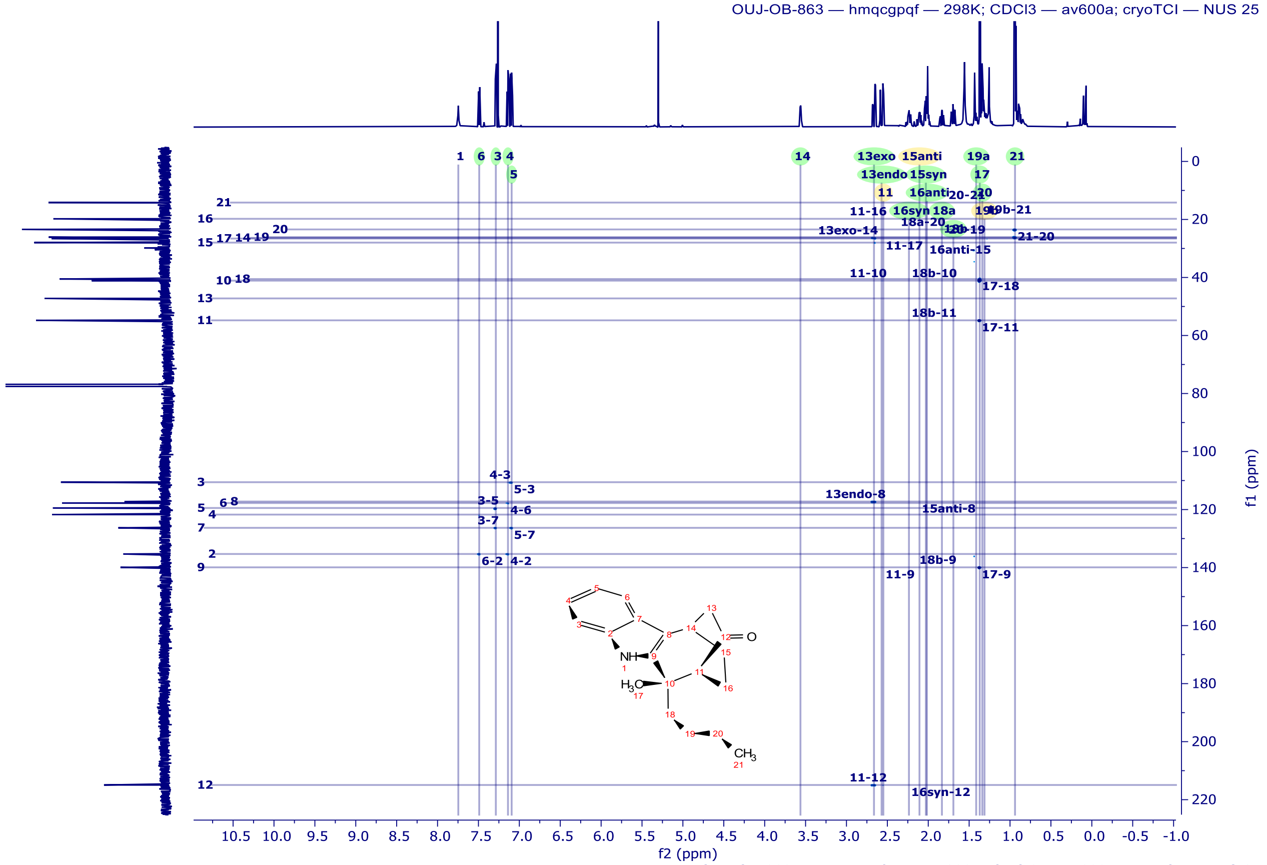

OUJ-OB-863 — cosygpmfphpp — 298K; CDCl3 — av600a; cryoTCI — NUS 25

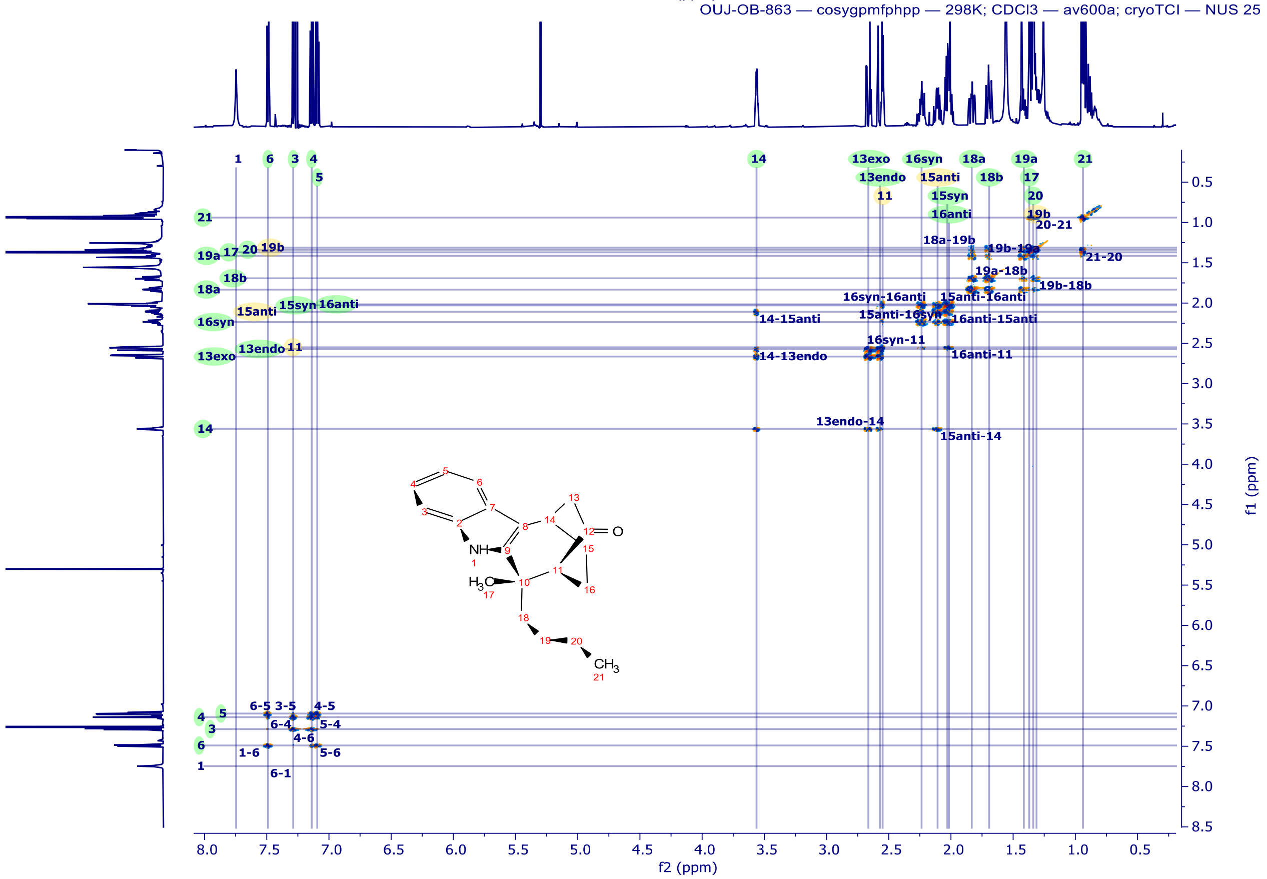

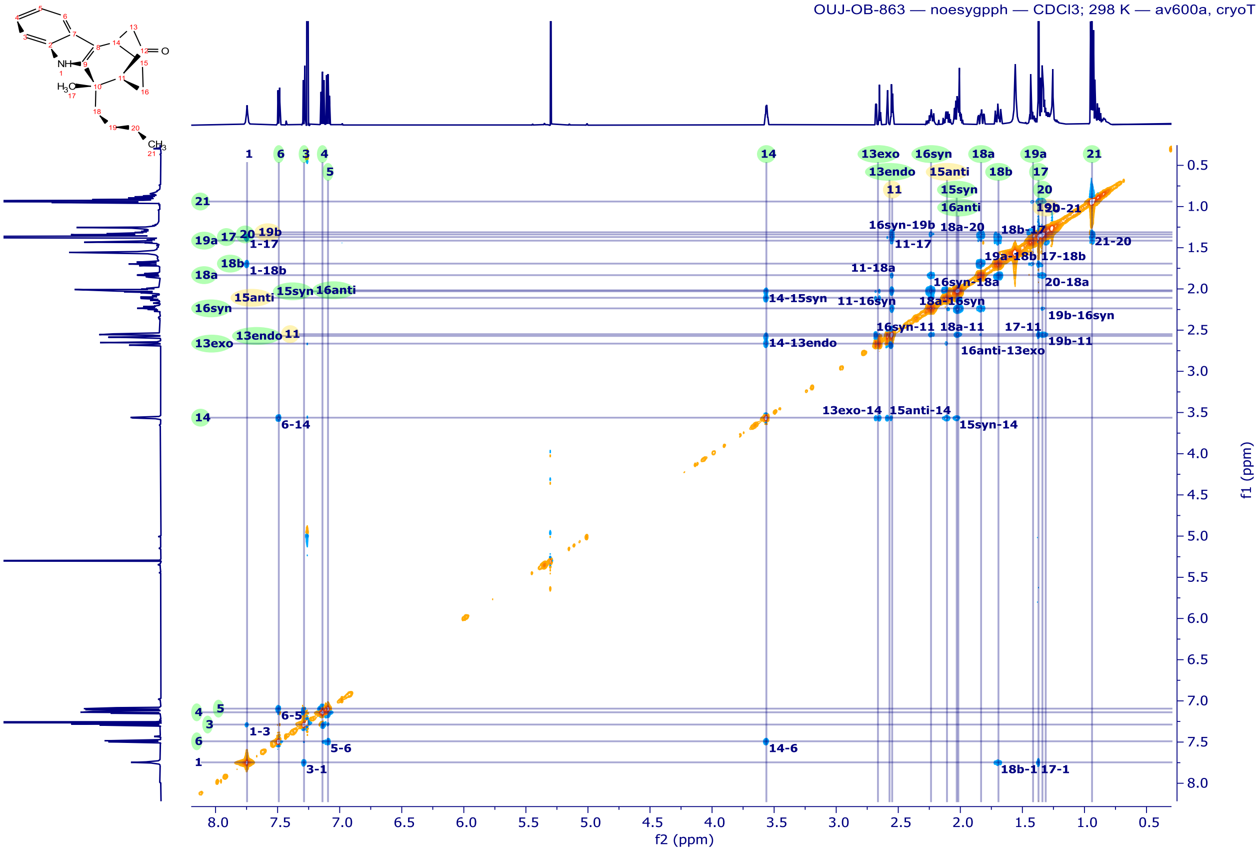


## Characterization of 4l'

**OUJ-OC-114-01**

**$CD_2Cl_2$; 298 K; 1 mg; av600a**

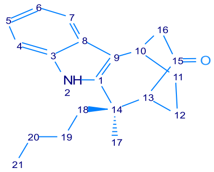

The NMR data are consistent with the proposed structure.

| Atom | δ (ppm) | J | HSQC | HMQC | COSY | NOESY | $^{15}$N-HMBC |
|---|---|---|---|---|---|---|---|
| C1 | 140.18 | | | 13, 17, 18a, 18b | | | |
| N2 | -258.7 | | | | | | 2 |
| H2 | 7.909 | br s | | | | 4, 17, 18a, 18b, 19a, 19b | 2 |
| C3 | 135.73 | | | 5, 7 | | | |
| C4 | 110.71 | | 4 | 6 | | | |
| H4 | 7.292 | d 1.1(6), d 8.0(5), d 0.8(7) | 4 | 6, 8 | 5, 6 | 2 | |
| C5 | 121.69 | | 5 | 7 | | | |
| H5 | 7.096 | d 7.1(6), d 1.2(7), d 8.0(4) | 5 | 3, 7 | 4, 6, 7 | | |
| C6 | 119.48 | | 6 | 4 | | | |
| H6 | 7.049 | d 7.8(7), d 7.1(5), d 1.1(4) | 6 | 4, 8 | 4, 5, 7 | | |
| C7 | 117.90 | | 7 | 5 | | | |
| H7 | 7.473 | dm 7.8(6) | 7 | 3, 5, 9 | 5, 6 | 10 | |
| C8 | 126.83 | | | 4, 6, 10 | | | |
| C9 | 116.40 | | | 7, 10, 11, 16a | | | |
| C10 | 27.39 | | 10 | 11, 12b, 16a | | | |
| H10 | 3.523 | m | 10 | 8, 9, 12, 16 | 11, 16a, 16b | 7, 11, 16a, 16b | |
| C11 | 28.13 | | 11 | 12a, 12b, 13, 16a, 16b | | | |
| H11 | 1.950 | m (o.l.) | 11 | 9, 10, 12, 16 | 10, 12a, 12b, 16b | 10, 16a | |
| C12 | 20.81 | | 12a, 12b | 10, 11, 13 | | | |
| H12a | 2.183 | m | 12 | 11, 13, 14, 15 | 11, 12b, 13 | 13, 17 | |
| H12b | 2.040 | m | 12 | 10, 11, 13, 14 | 11, 12a, 13 | 13, 16a | |
| C13 | 55.64 | | 13 | 12a, 12b, 17, 18a | | | |
| H13 | 2.572 | m (o.l.) | 13 | 1, 11, 12, 14, 15, 16, 17 | 12a, 12b | 12a, 12b, 17, 18a, 18b, 19a, 19b | |
| C14 | 42.03 | | | 12a, 12b, 13, 17, 18a, 18b | | | |
| C15 | 214.93 | | | 12a, 13, 16a, 16b | | | |
| C16 | 48.09 | | 16a, 16b | 10, 11, 13 | | | |
| H16a | 2.631 | d 4.8(10), d 18.5(16b), d 1.0(13?) | 16 | 9, 10, 11, 15 | 10, 16b | 10, 11, 12b | |
| H16b | 2.555 | m (o.l.) | 16 | 11, 15 | 10, 11, 16a | 10, 18b | |
| C17 | 25.17 | | 17 | 13, 18a, 18b, 19a, 19b | | | |
| H17 | 1.401 | s | 17 | 1, 13, 14, 18 | | 2, 12a, 13, 18a, 18b | |
| C18 | 43.04 | | 18a, 18b | 17 | | | |
| H18a | 1.625 | m | 18 | 1, 13, 14, 17, 19, 20 | 19a, 19b | 2, 13, 17, 20 | |
| H18b | 1.569 | m (o.l.) | 18 | 1, 14, 17, 19, 20 | 19a, 19b | 2, 13, 16b, 17, 20 | |
| C19 | 26.26 | | 19a, 19b | 18a, 18b, 20, 21 | | | |
| H19a | 1.564 | m (o.l.) | 19 | 17, 20 | 18a, 18b, 19b | 2, 13, 21 | |
| H19b | 1.310 | m (o.l.) | 19 | 17, 20 | 18a, 18b, 19a | 2, 13, 21 | |
| C20 | 23.70 | | 20 | 18a, 18b, 19a, 19b, 21 | | | |
| H20 | 1.270 | m (o.l.) | 20 | 19, 21 | 21 | 18a, 18b | |
| C21 | 14.18 | | 21 | 20 | | | |
| H21 | 0.902 | t 7.2(20) | 21 | 19, 20 | 20 | 19a, 19b | |

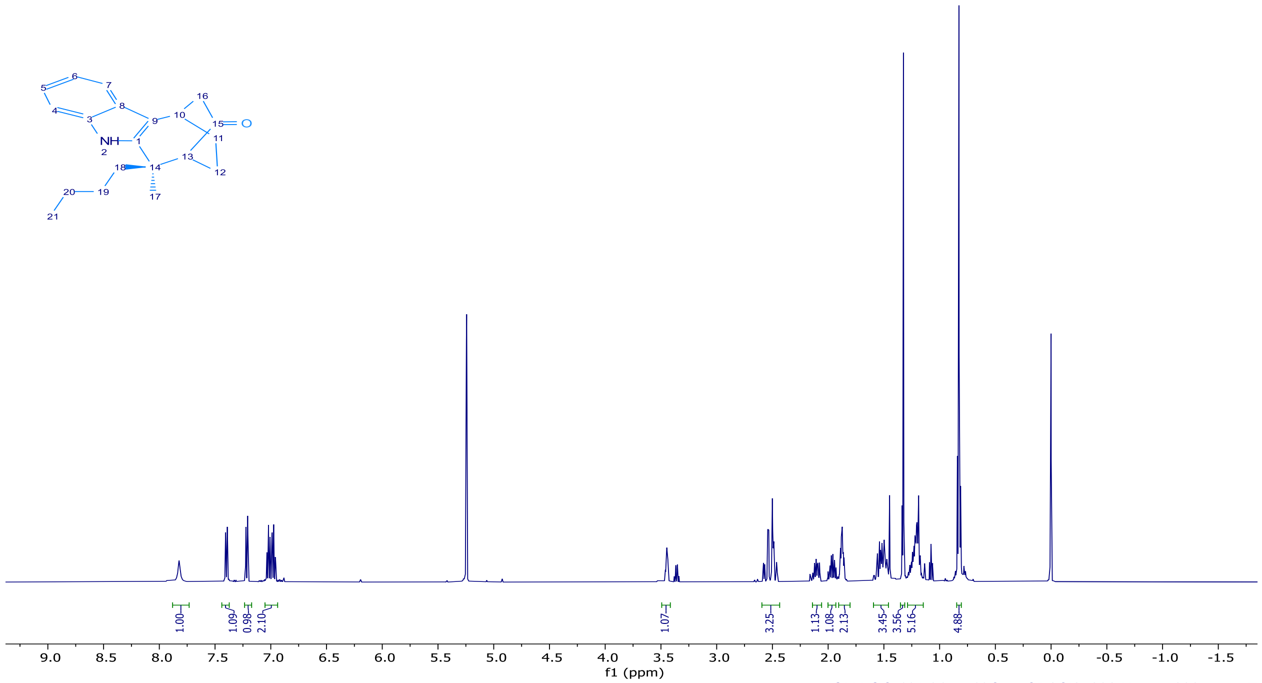

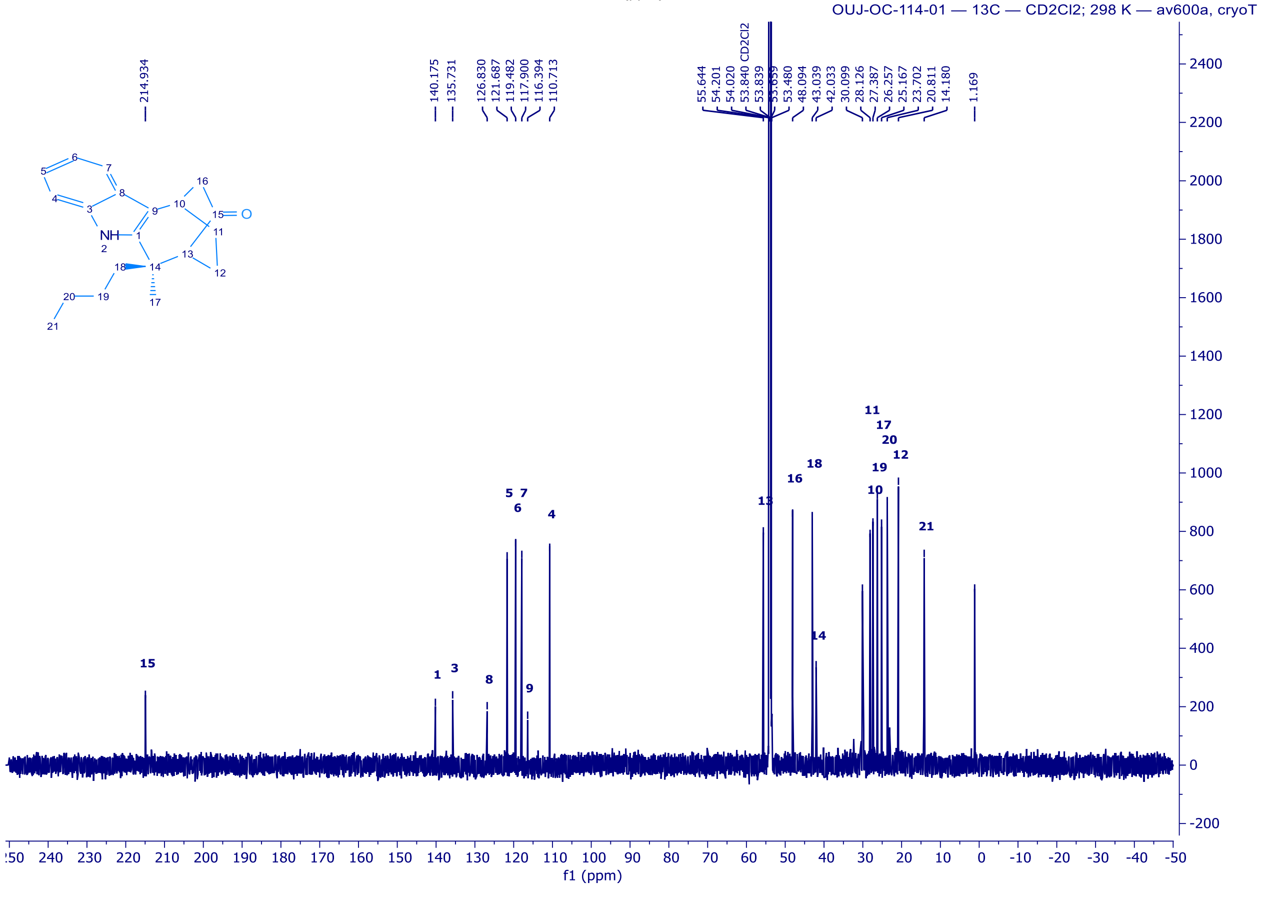

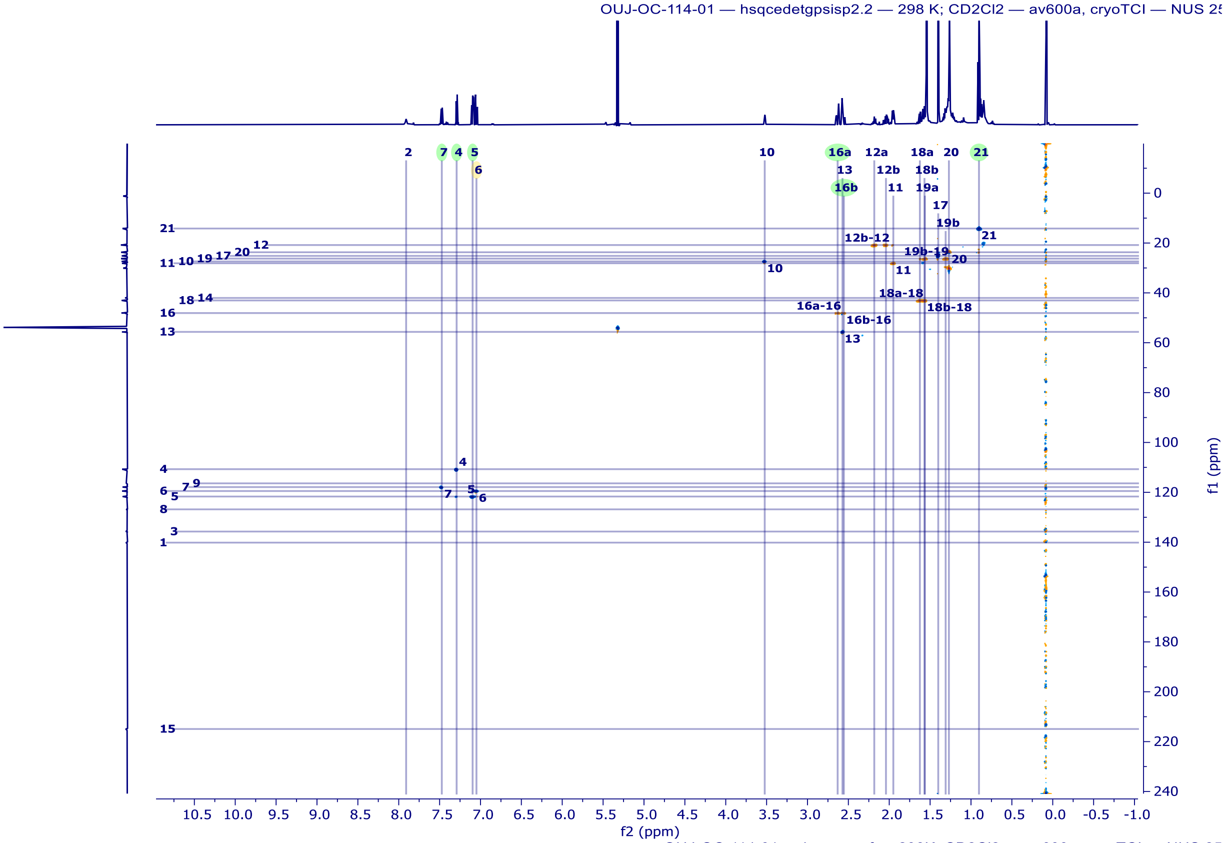
OUJ-OC-114-01 — hsqcedetgpsisp2.2 — 298 K; CD2Cl2 — av600a, cryoTCI — NUS 25
f2 (ppm)
f1 (ppm)

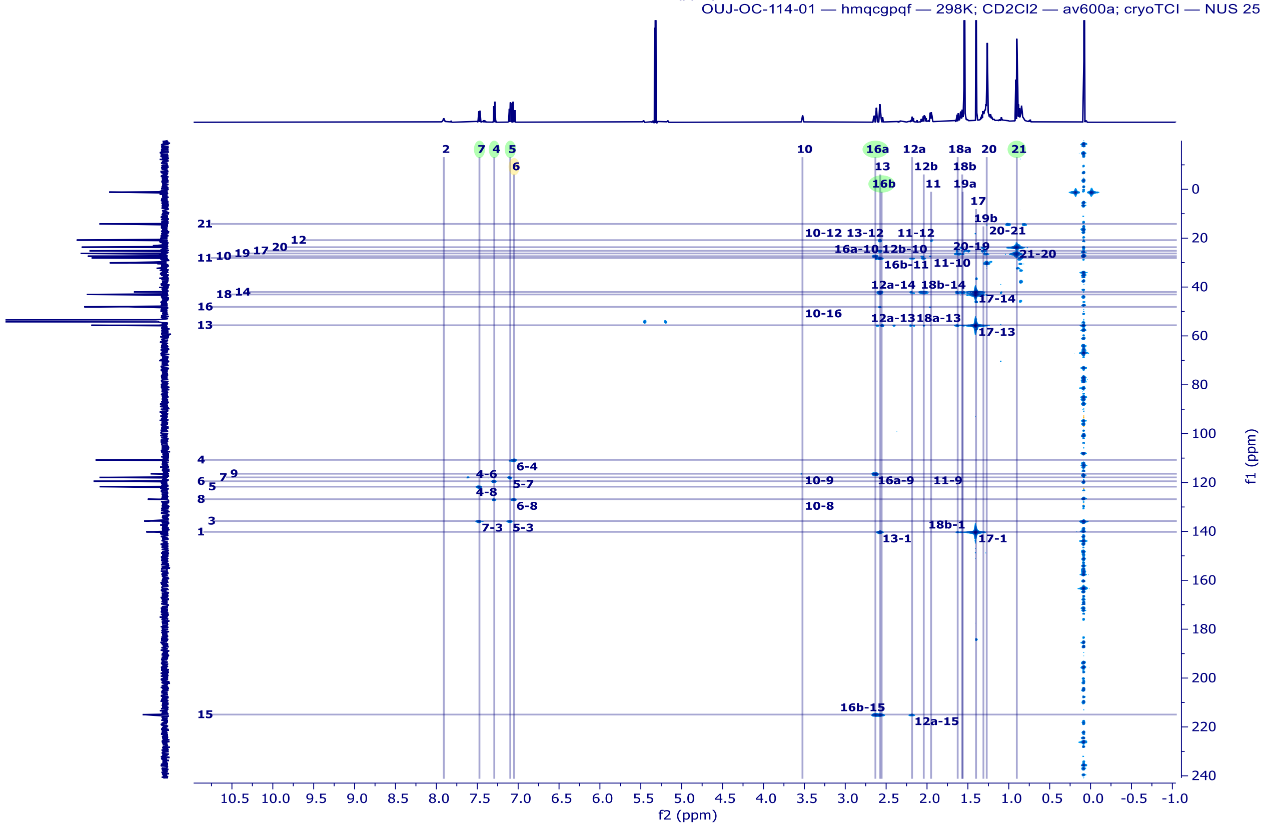
OUJ-OC-114-01 — hmqcgpqf — 298K; CD2Cl2 — av600a; cryoTCI — NUS 25
f2 (ppm)
f1 (ppm)

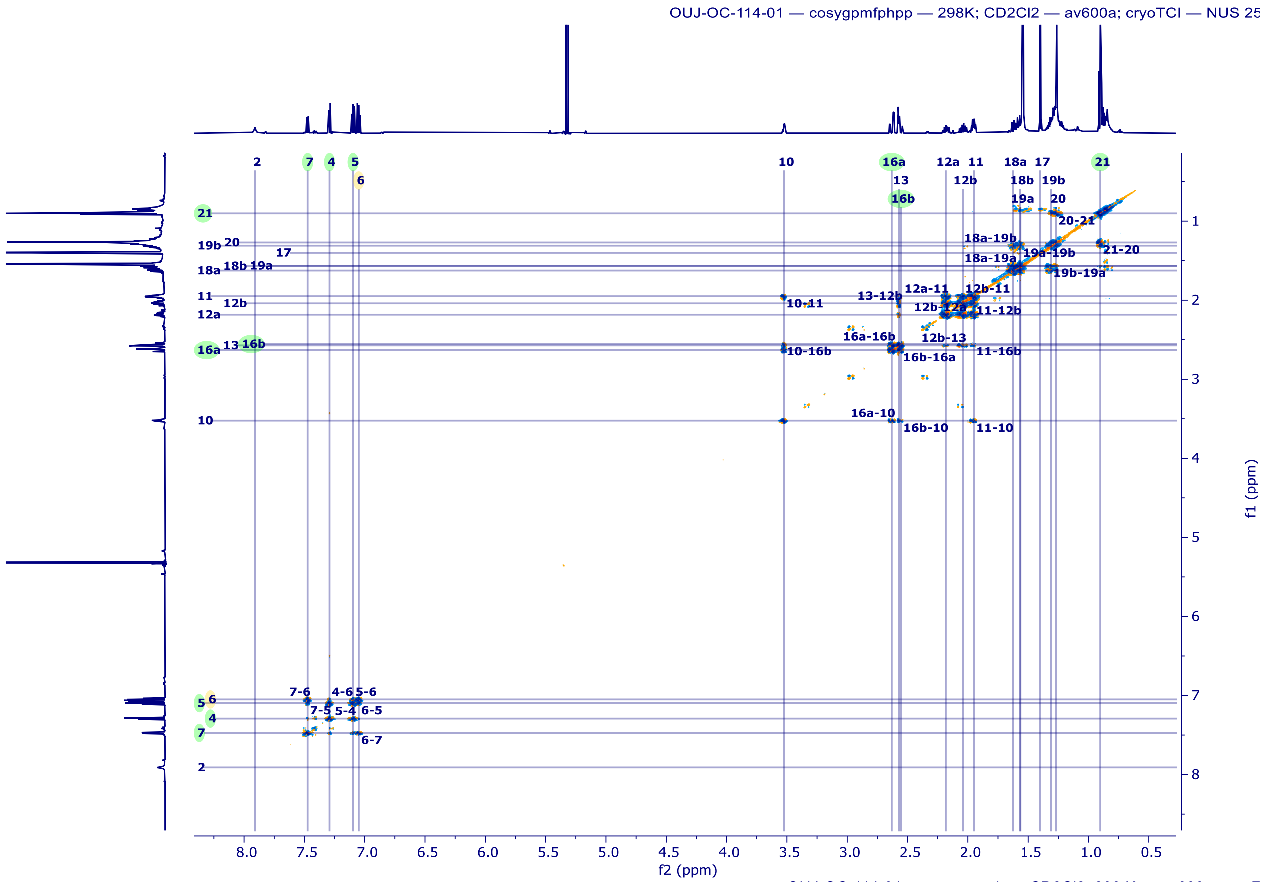
OUJ-OC-114-01 — cosygpmfphpp — 298K; CD2Cl2 — av600a; cryoTCI — NUS 25
f2 (ppm)
f1 (ppm)

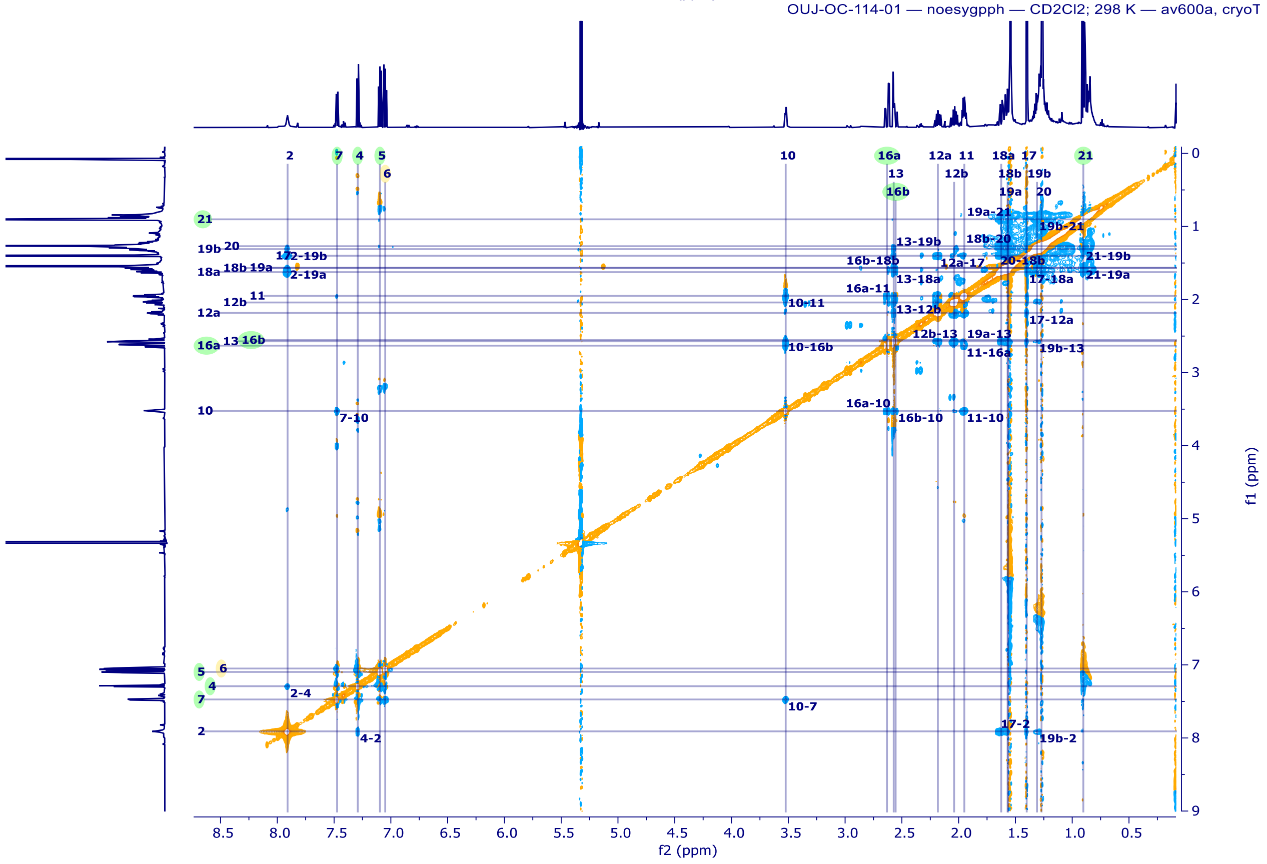
OUJ-OC-114-01 — noesygpph — CD2Cl2; 298 K — av600a, cryoT
f2 (ppm)
f1 (ppm)

## Characterization of 4m

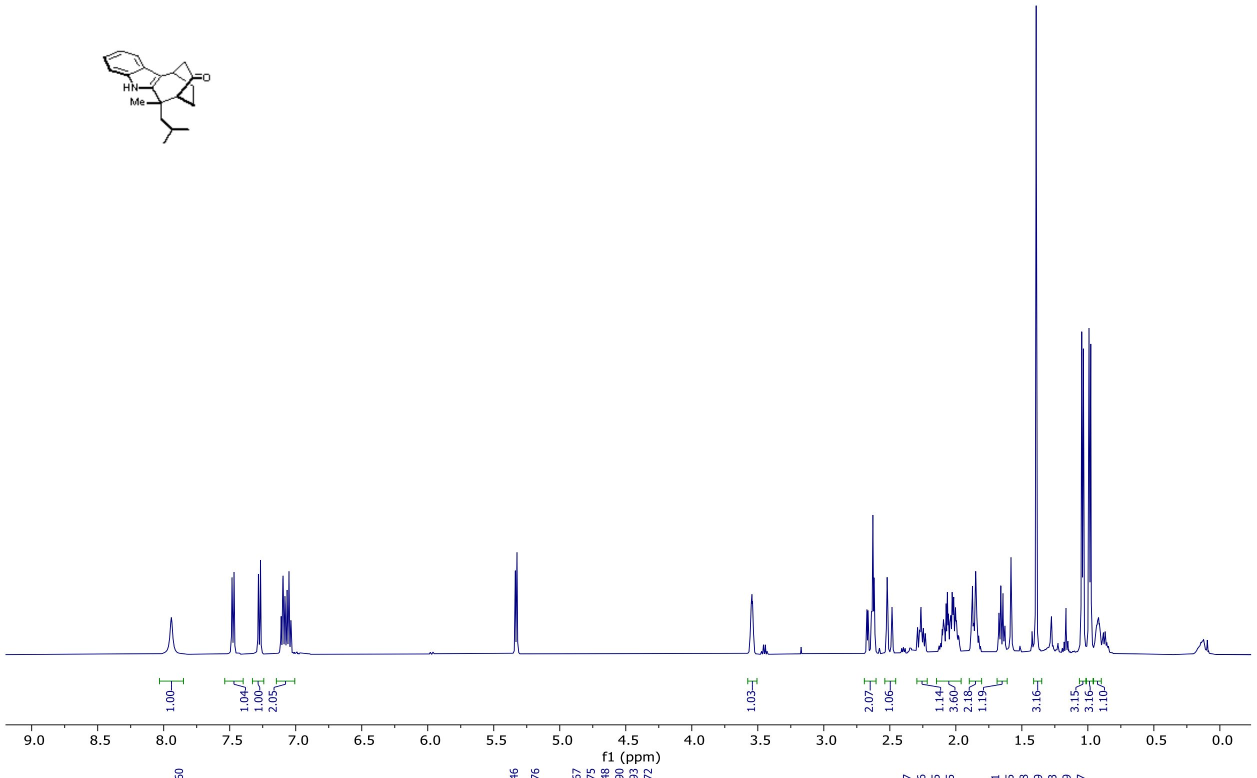


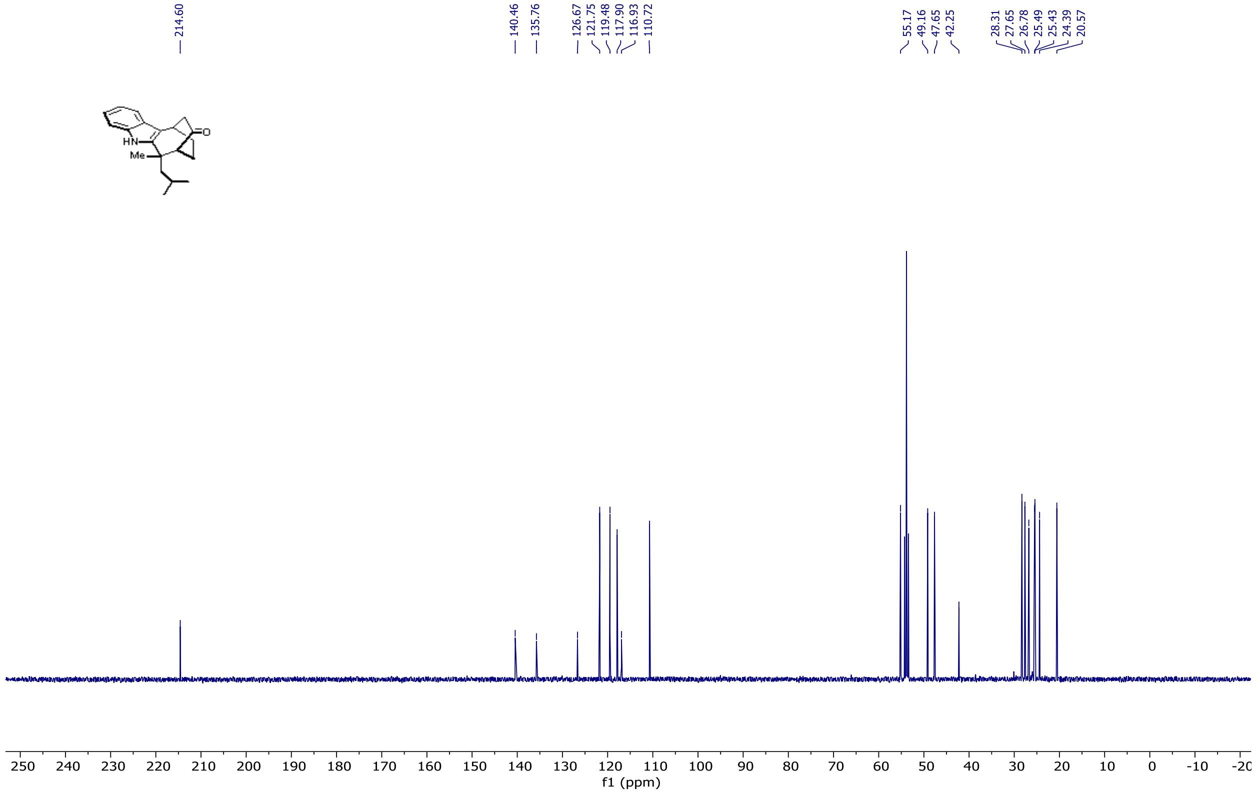

# Characterization of 4m’

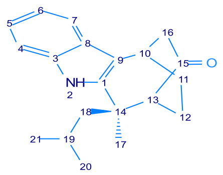


**OUJ-OC-121**

**$CD_2Cl_2$; 298 K; 2 mg; av600a**

| Atom | δ (ppm) | J | HSQC | HMQC | COSY | NOESY | ¹⁵N-HMBC |
|---|---|---|---|---|---|---|---|
| C1 | 140.18 | | | 13, 17, 18a, 18b | | | |
| N2 | -258.6 | | | | | | 2 |
| H2 | 7.933 | br s | | | | 4, 17, 18a, 18b, 19, 20, 21 | 2 |
| C3 | 135.72 | | | 5, 7 | | | |
| C4 | 110.72 | | 4 | 6 | | | |
| H4 | 7.291 | d 1.1(6), d 8.0(5), d 0.8(7) | 4 | 6, 8 | 5 | 2 | |
| C5 | 121.71 | | 5 | 7 | | | |
| H5 | 7.096 | d 7.1(6), d 1.3(7), d 8.0(4) | 5 | 3, 7 | 4, 6, 7 | | |
| C6 | 119.50 | | 6 | 4 | | | |
| H6 | 7.049 | d 7.8(7), d 7.1(5), d 1.1(4) | 6 | 4, 8 | 5, 7 | | |
| C7 | 117.92 | | 7 | 5 | | | |
| H7 | 7.469 | dm 7.8(6) | 7 | 3, 5, 9 | 5, 6 | 10 | |
| C8 | 126.79 | | | 4, 6 | | | |
| C9 | 116.47 | | | 7, 10, 11a, 16a | | | |
| C10 | 27.35 | | 10 | 11a, 12b, 16a | | | |
| H10 | 3.522 | t 4.7(11a, 16a), t 2.5(11b, 16b) | 10 | 9, 15, 16 | 11a, 11b, 16a, 16b | 7, 11a, 11b, 16a, 16b | |
| C11 | 28.16 | | 11a, 11b | 12a, 12b, 13, 16a, 16b | | | |
| H11a | 1.973 | m (o.l.) | 11 | 9, 10, 12, 16 | 10, 11b, 12a, 12b | 10, 16a | |
| H11b | 1.925 | m (o.l.) | 11 | 12 | 10, 11a, 12a, 12b, 16b | 10 | |
| C12 | 20.87 | | 12a, 12b | 11a, 11b, 13 | | | |
| H12a | 2.196 | d 14.5(12b), d 5.7(11a), d 10.0(11b), d 1.3(13) | 12 | 11, 13, 14, 15 | 11a, 11b, 12b, 13 | 13, 17 | |
| H12b | 2.041 | d 9.8(11a), d 4.8(11b), d 14.5(12a), d 6.9(13) | 12 | 10, 11, 14 | 11a, 11b, 12a, 13 | 13, 16a | |
| C13 | 56.00 | | 13 | 12a, 17, 18a, 18b | | | |
| H13 | 2.629 | d 1.3(12a), d 6.9(12b), d 1.0(16a?) | 13 | 1, 11, 12, 14, 15, 16, 17 | 12a, 12b | 12a, 12b, 17, 18a, 18b, 19, 20, 21 | |
| C14 | 42.74 | | | 12a, 12b, 13, 17, 18a, 18b, 19 | | | |
| C15 | 215.03 | | | 10, 12a, 13, 16a, 16b | | | |
| C16 | 48.03 | | 16a, 16b | 10, 11a, 13 | | | |
| H16a | 2.645 | d 18.6(16b), d 5.0(10), d 1.0(13?) | 16 | 9, 10, 11, 15 | 10, 16b | 10, 11a, 12b, 17 | |
| H16b | 2.555 | d 18.6(16a), t 2.3(10, 11b) | 16 | 11, 15 | 10, 11b, 16a | 10, 18a, 18b, 20, 21 | |
| C17 | 25.96 | | 17 | 13, 18a, 18b | | | |
| H17 | 1.446 | s | 17 | 1, 13, 14, 18 | 18a | 2, 12a, 13, 16a, 19, 21 | |
| C18 | 51.44 | | 18a, 18b | 17, 20, 21 | | | |
| H18a | 1.601 | d 14.4(18b), d 3.9(19) | 18 | 1, 13, 14, 17, 19, 20, 21 | 17, 19 | 2, 13, 16b, 20 | |
| H18b | 1.550 | d 6.6(19), d 14.4(18a) | 18 | 1, 13, 14, 17, 19, 20, 21 | 19 | 2, 13, 16b, 21 | |
| C19 | 24.50 | | 19 | 18a, 18b, 20, 21 | | | |
| H19 | 1.967 | m (o.l.) | 19 | 14, 20, 21 | 18a, 18b, 20, 21 | 2, 13, 17, 20 | |
| C20 | 25.28 | | 20 | 18a, 18b, 19, 21 | | | |
| H20 | 0.916 | d 6.7(19) | 20 | 18, 19, 21 | 19 | 2, 13, 16b, 18a, 19 | |
| C21 | 25.20 | | 21 | 18a, 18b, 19, 20 | | | |
| H21 | 0.930 | d 6.6(19) | 21 | 18, 19, 20 | 19 | 2, 13, 16b, 17, 18b | |

**The NMR data are consistent with the proposed structure.**

OUJ-OC-121-01 — 1H — CD2Cl2; 298 K; 2 mg; ELNA-6378 — av600a, cryoT

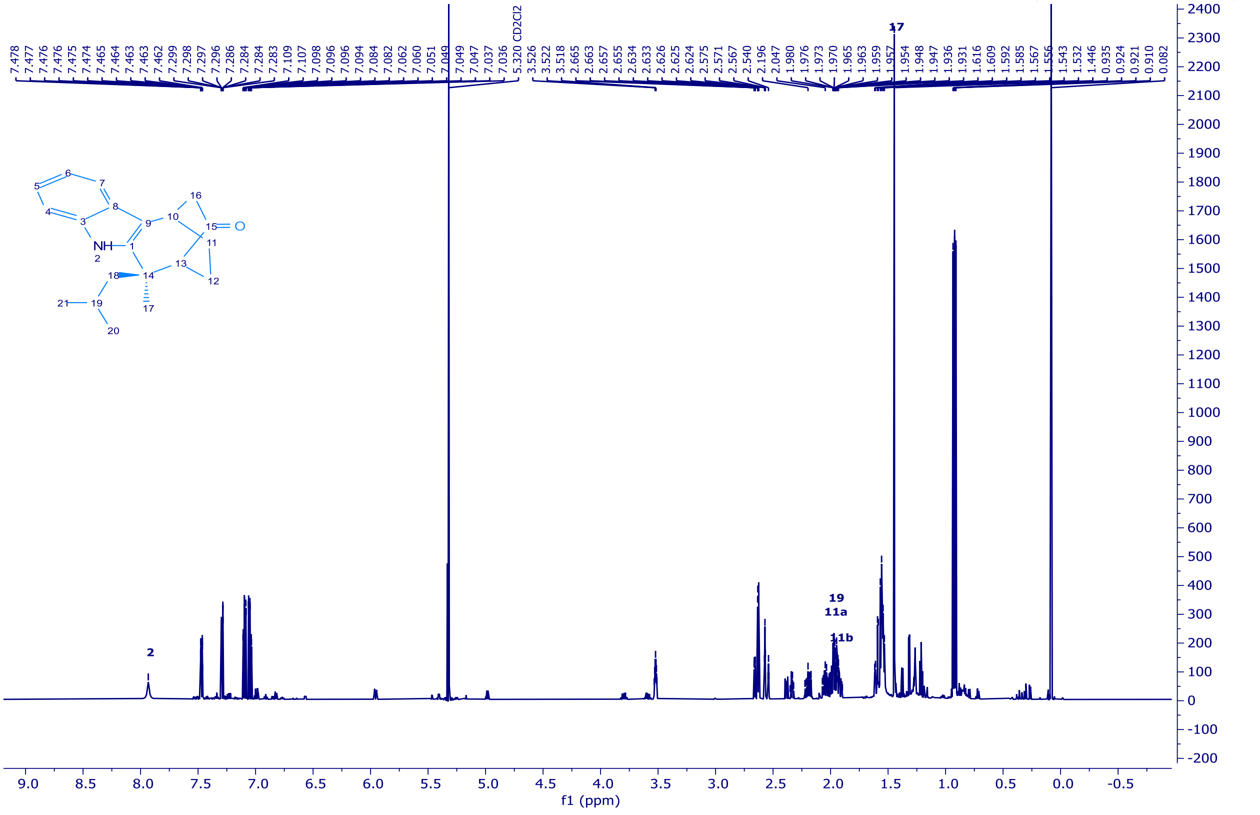

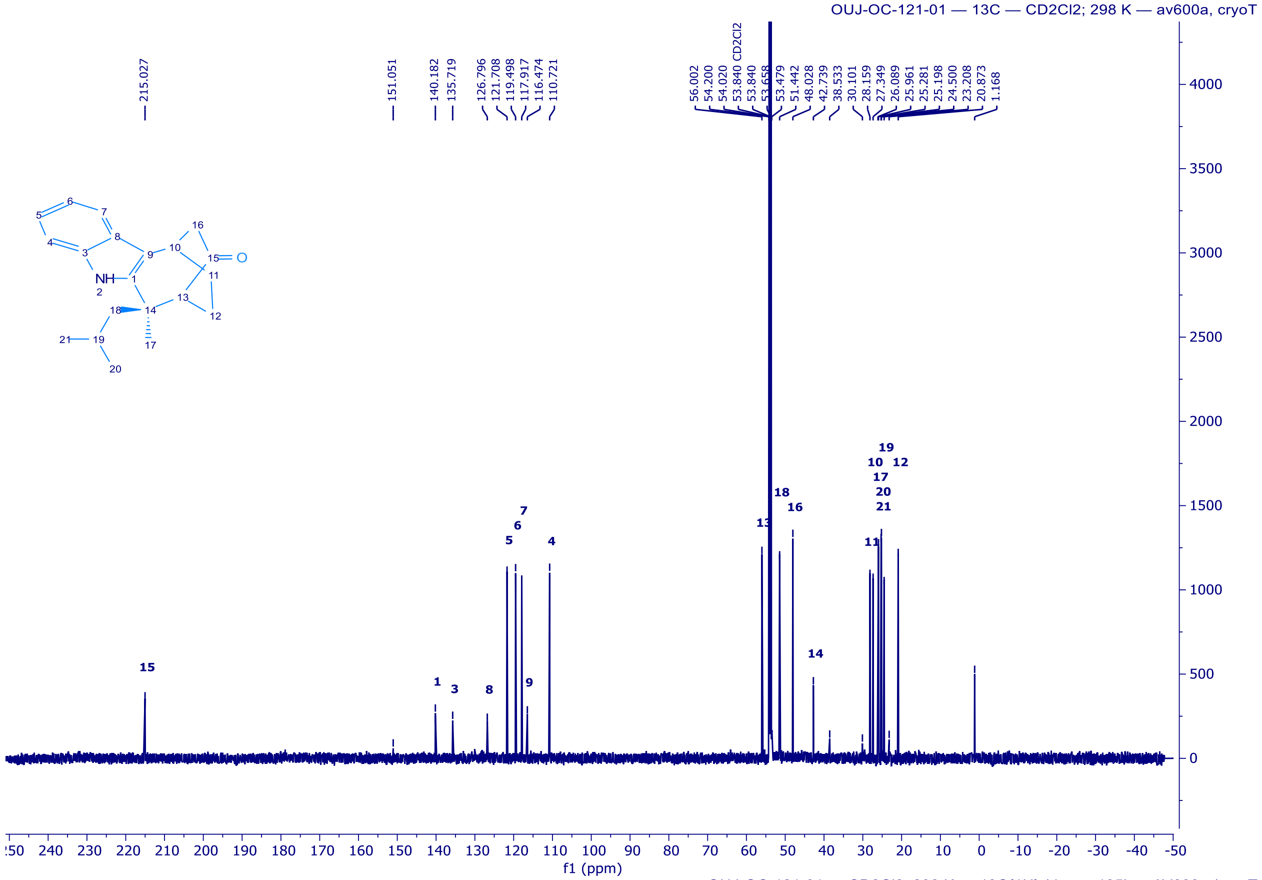
OUJ-OC-121-01 — 13C — CD2Cl2; 298 K — av600a, cryoT
215.027
151.051
140.182
135.719
126.796
121.708
119.498
117.917
116.474
110.721
56.002
54.200
54.020
53.840 CD2Cl2
53.840
53.658
53.479
51.442
48.028
42.739
38.533
30.101
28.159
27.349
26.089
25.961
25.281
25.198
24.500
23.208
20.873
1.168
f1 (ppm)

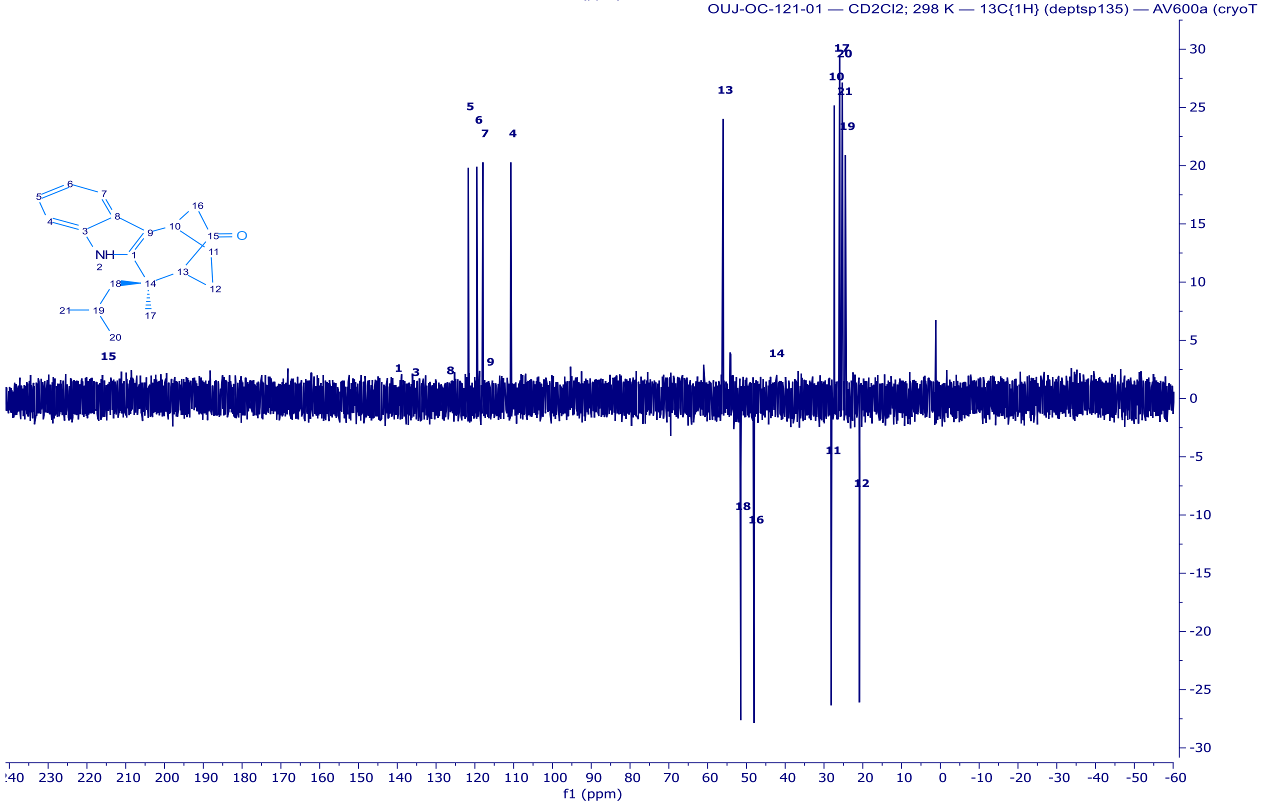
OUJ-OC-121-01 — CD2Cl2; 298 K — 13C{1H} (deptsp135) — AV600a (cryoT
f1 (ppm)

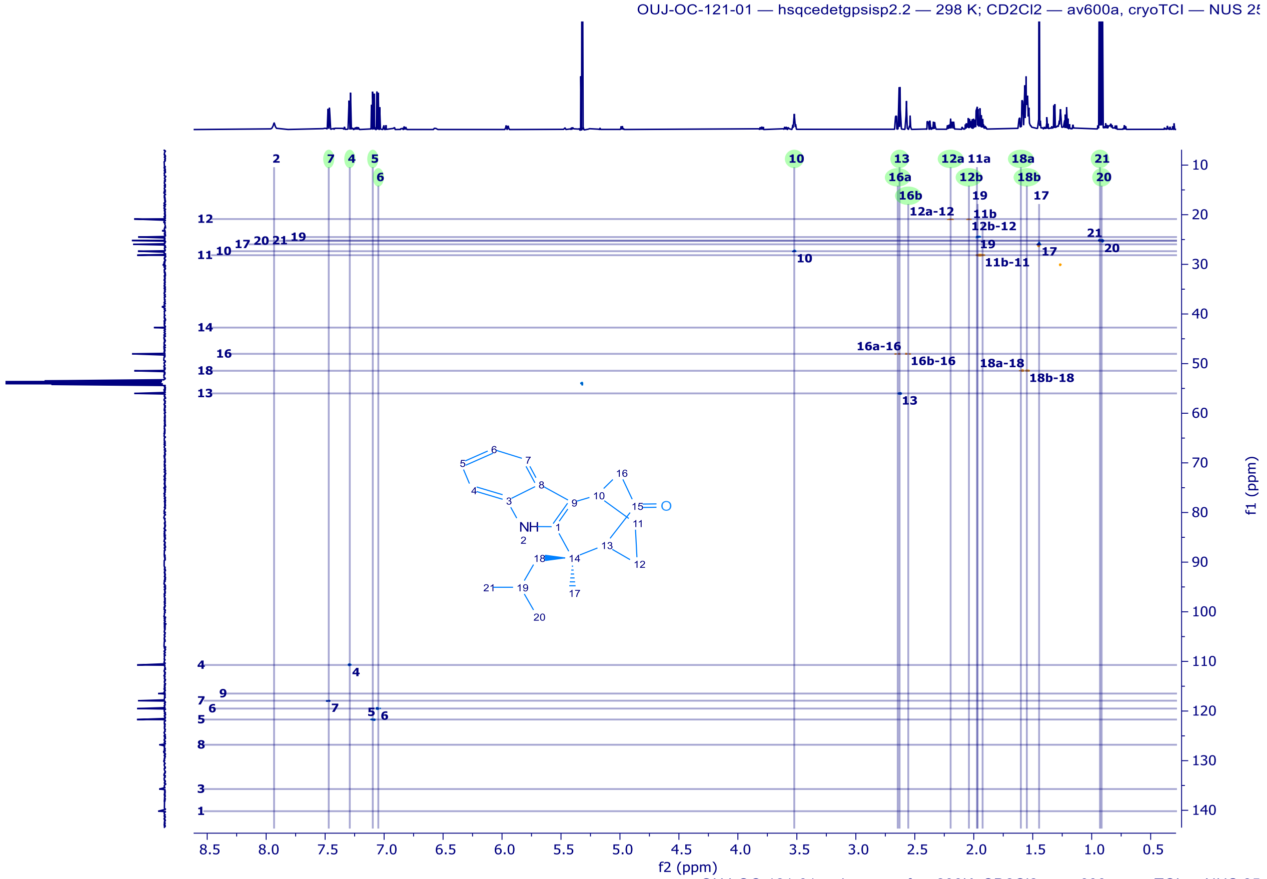
OUJ-OC-121-01 — hsqcedetgpsisp2.2 — 298 K; CD2Cl2 — av600a, cryoTCI — NUS 25
f2 (ppm)
f1 (ppm)

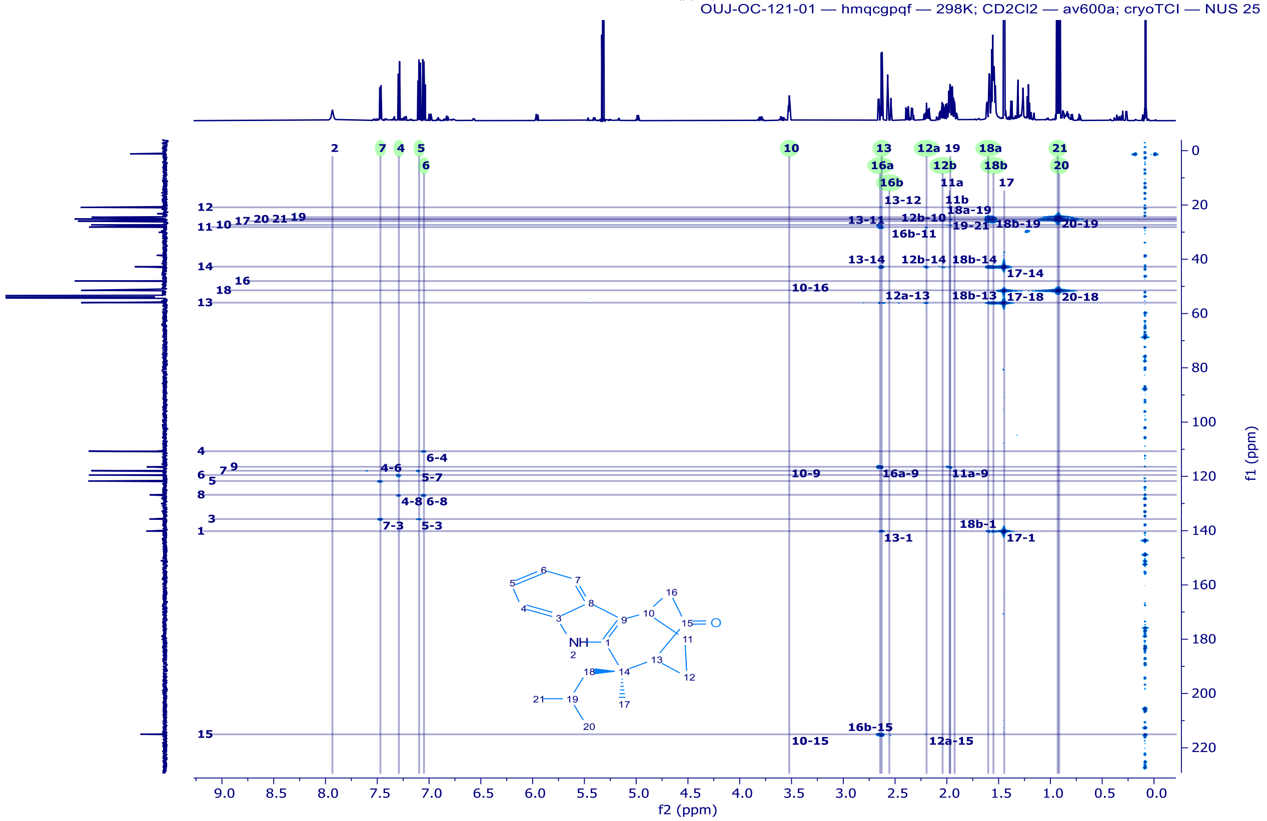
OUJ-OC-121-01 — hmqcgpqf — 298K; CD2Cl2 — av600a; cryoTCI — NUS 25
f2 (ppm)
f1 (ppm)

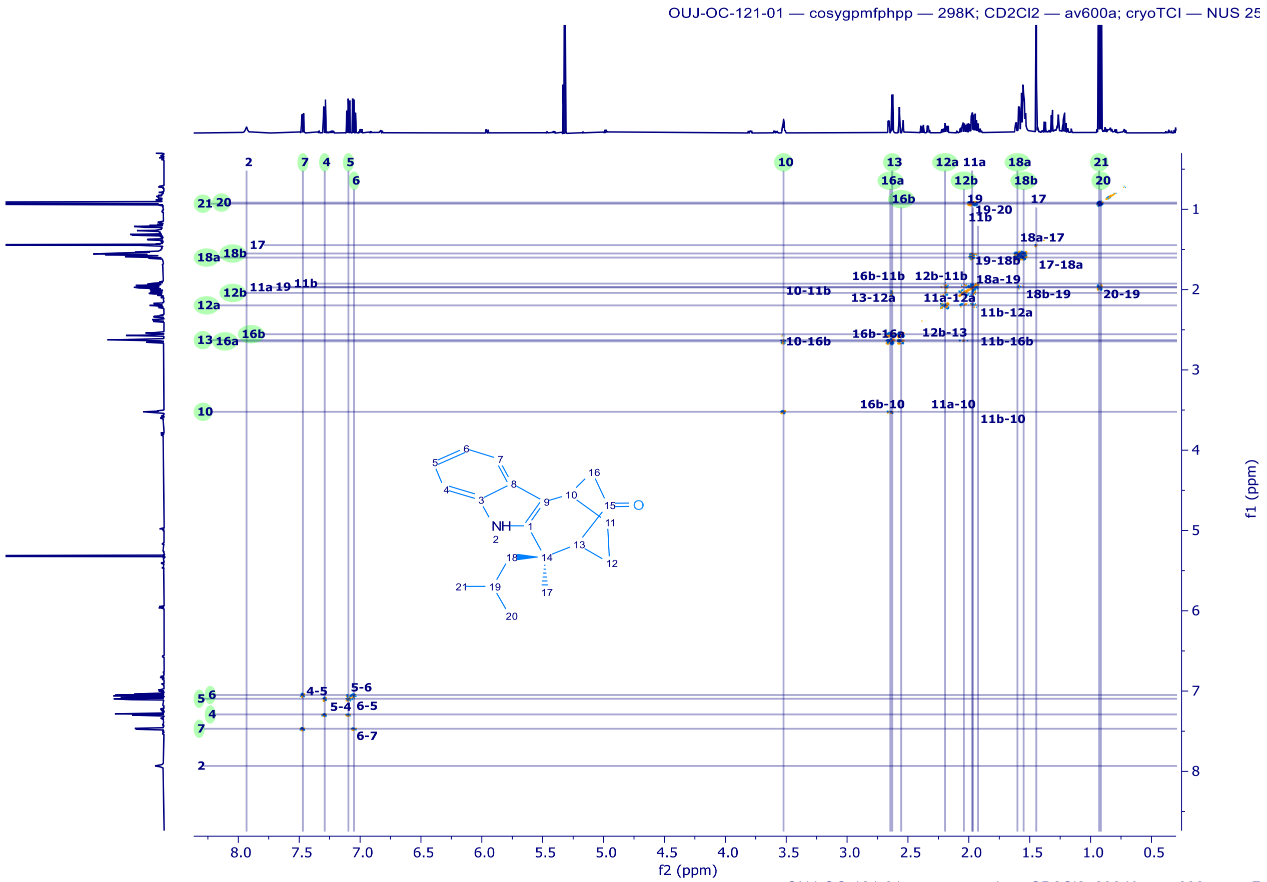
OUJ-OC-121-01 — cosygpmfphpp — 298K; CD2Cl2 — av600a; cryoTCI — NUS 25
f2 (ppm)
f1 (ppm)

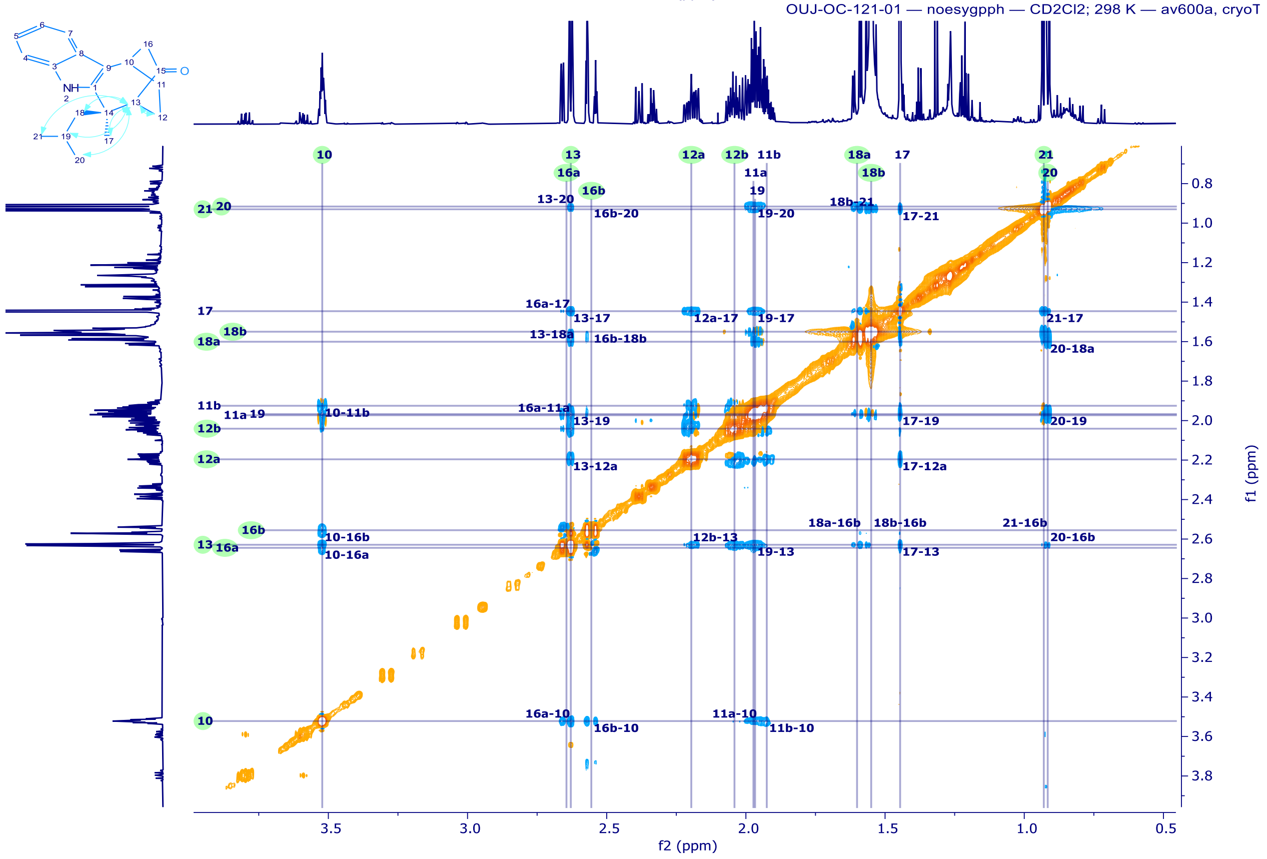
OUJ-OC-121-01 — noesygpph — CD2Cl2; 298 K — av600a, cryoT
f2 (ppm)
f1 (ppm)

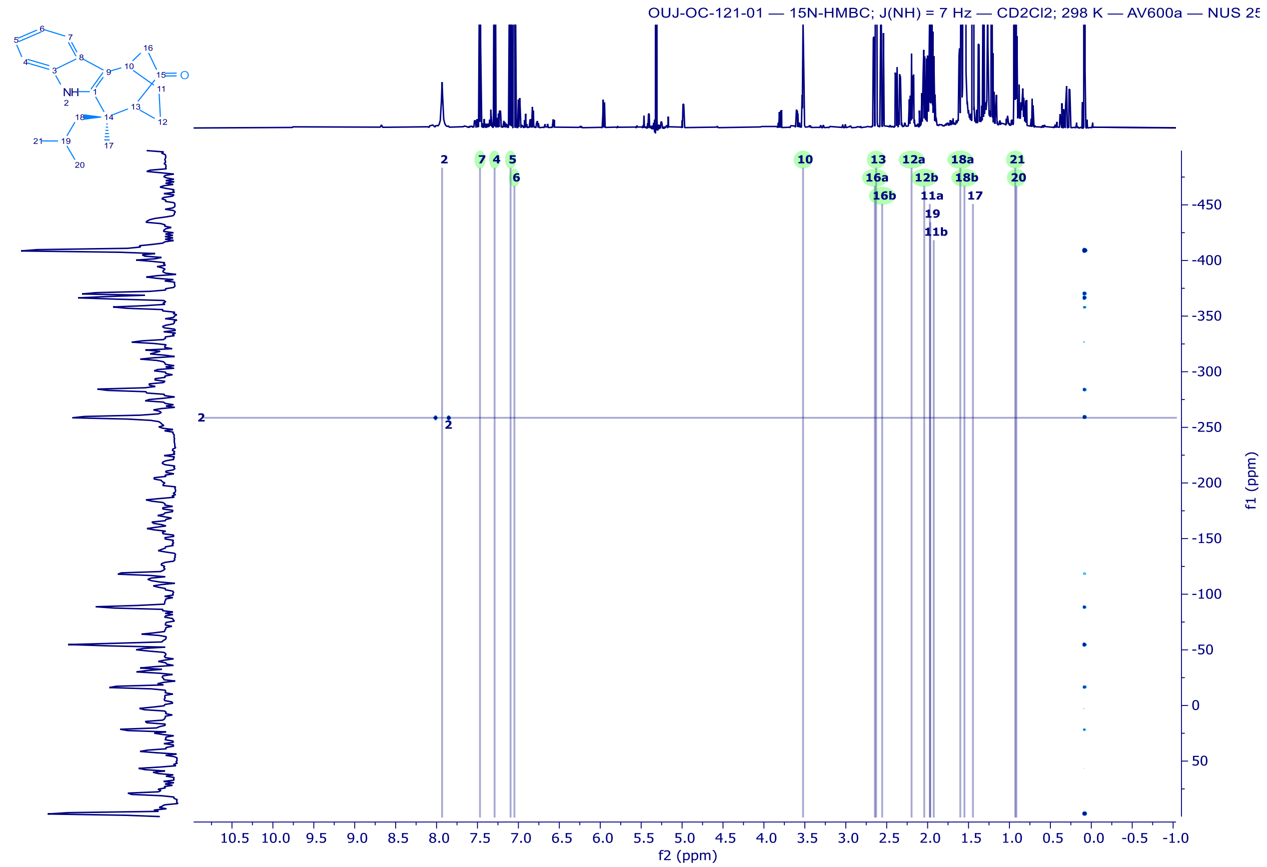


## Characterization of 4n

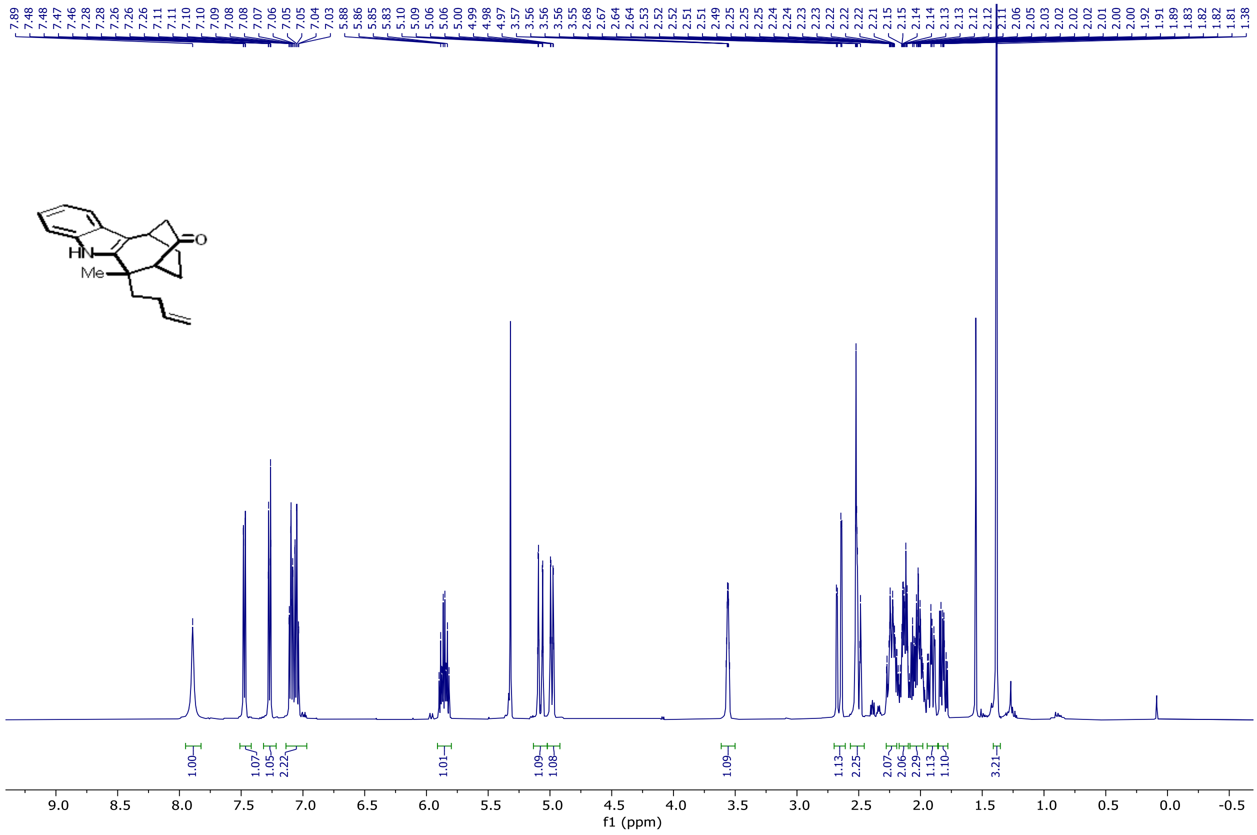

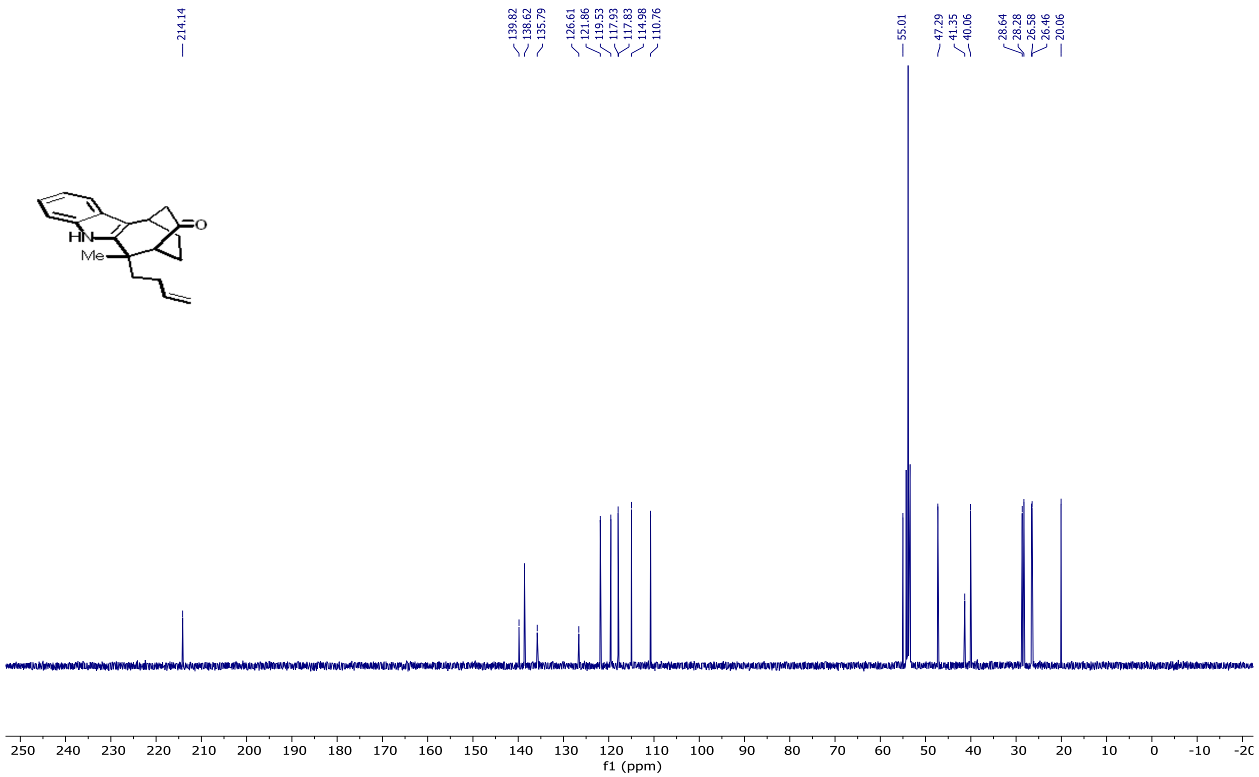


# Characterization of 4n'

## OUJ-OC-117-01

**$CD_2Cl_2$; 298 K; 0.5 mg; av600a**

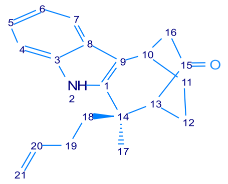


**The NMR data are consistent with the proposed structure.**

| Atom | δ (ppm) | J | HSQC | HMQC | COSY | NOESY | $^{15}$N-HMBC |
|---|---|---|---|---|---|---|---|
| C1 | 139.70 | | | 13, 17, 18a, 18b | | | |
| N2 | -259.0 | | | | | | 2 |
| H2 | 7.903 | brs | | | | 4, 17, 18a, 18b, 19a, 19b | 2 |
| C3 | 135.79 | | | 5, 7 | | | |
| C4 | 110.76 | | 4 | 6 | | | |
| H4 | 7.292 | d 1.1(6), d 0.8(7), d 8.0(5) | 4 | 6, 8 | 5, 6 | 2 | |
| C5 | 121.81 | | 5 | 7 | | | |
| H5 | 7.104 | d 7.1(6), d 8.0(4), d 1.3(7) | 5 | 3, 7 | 4, 6, 7 | | |
| C6 | 119.55 | | 6 | 4 | | | |
| H6 | 7.056 | d 7.8(7), d 1.1(4), d 7.1(5) | 6 | 4, 8 | 4, 5, 7 | | |
| C7 | 117.96 | | 7 | 5 | | | |
| H7 | 7.480 | dm 7.8(6) | 7 | 3, 5 | 5, 6 | 10 | |
| C8 | 126.81 | | | 4, 6 | | | |
| C9 | 116.59 | | | 10, 11, 16a | | | |
| C10 | 27.33 | | 10 | 11, 12b, 16a | | | |
| H10 | 3.534 | m | 10 | 9, 15 | 11, 16a, 16b | 7, 11, 16a, 16b | |
| C11 | 28.12 | | 11 | 12a, 12b, 13, 16a, 16b | | | |
| H11 | 1.964 | m (o.l.) | 11 | 9, 10, 12, 16 | 10, 12a, 12b, 16b | 10, 16a | |
| C12 | 20.72 | | 12a, 12b | 11, 13 | | | |
| H12a | 2.191 | m | 12 | 11, 14, 15 | 11, 12b, 13 | 13, 17 | |
| H12b | 2.058 | m | 12 | 10, 11, 13, 14 | 11, 12a, 13 | 13 | |
| C13 | 55.45 | | 13 | 12b, 17, 18a | | | |
| H13 | 2.587 | m (o.l.) | 13 | 1, 11, 12, 14, 15, 16, 17, 18 | 12a, 12b | 12a, 12b, 17, 18a, 18b, 19a | |
| C14 | 42.00 | | | 12a, 12b, 13, 17, 18a, 18b | | | |
| C15 | 214.81 | | | 10, 12a, 13, 16a, 16b | | | |
| C16 | 48.06 | | 16a, 16b | 11, 13 | | | |
| H16a | 2.642 | d 4.8(10), d 18.6(16b), 1.0(13?) | 16 | 9, 10, 11, 15 | 10, 16b | 10, 11 | |
| H16b | 2.569 | m (o.l.) | 16 | 11, 15 | 10, 11, 16a | 10, 18a, 18b | |
| C17 | 25.16 | | 17 | 13, 18a, 18b | | | |
| H17 | 1.429 | s | 17 | 1, 13, 14, 18 | 18b | 2, 12a, 13, 18a, 19a, 19b | |
| C18 | 42.38 | | 18a, 18b | 13, 17, 19a, 19b, 20 | | | |
| H18a | 1.736 | d 13.9(18b), d 12.2(19a), d 4.7(19b) | 18 | 1, 13, 14, 17, 19, 20 | 18b, 19a, 19b | 2, 13, 16b, 17, 19b, 20, 21a | |
| H18b | 1.665 | d 4.7(19a), d 12.2(19b), d 13.9(18a) | 18 | 1, 14, 17, 19, 20 | 17, 18a, 19a, 19b | 2, 13, 16b, 19a, 20, 21a | |
| C19 | 28.46 | | 19a, 19b | 18a, 18b, 20, 21a | | | |
| H19a | 2.349 | d 4.7(18b), d 1.2(21b), d 14.0(19b), d 12.2(18a), d 6.3(20), d 1.6(21a) | 19 | 18, 20, 21 | 18a, 18b, 19b, 20, 21a, 21b | 2, 13, 17, 18b, 19b, 21a | |
| H19b | 2.107 | d 12.2(18b), d 14.0(19a), d 1.2(21b), d 4.7(18a), d 6.8(20), d 1.6(21a) | 19 | 18, 20, 21 | 18a, 18b, 19a, 20, 21a, 21b | 2, 17, 18a, 19a, 21a | |
| C20 | 138.92 | | 20 | 18a, 18b, 19a, 19b | | | |
| H20 | 5.794 | d 6.3(19a), d 6.8(19b), d 10.2(21b), d 17.0(21a) | 20 | 18, 19 | 19a, 19b, 21a, 21b | 18a, 18b | |
| C21 | 114.71 | | 21a, 21b | 19a, 19b | | | |
| H21a | 5.026 | t 1.6(19a, b), d 17.0(20), d 2.0(21b) | 21 | 19 | 19a, 19b, 20, 21b | 18a, 18b, 19a, 19b | |
| H21b | 4.935 | t 1.2(19a, b), d 10.2(20), d 2.0(21a) | 21 | | 19a, 19b, 20, 21a | | |

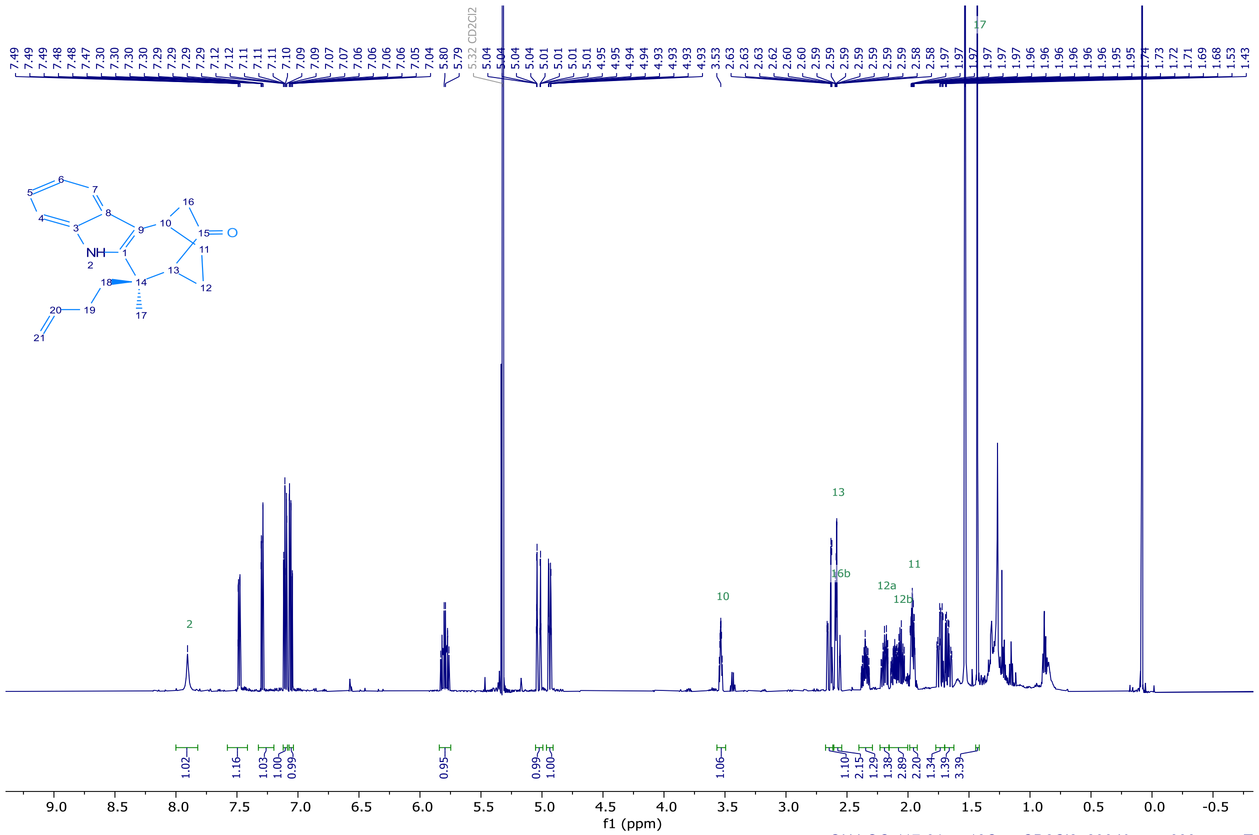
f1 (ppm)

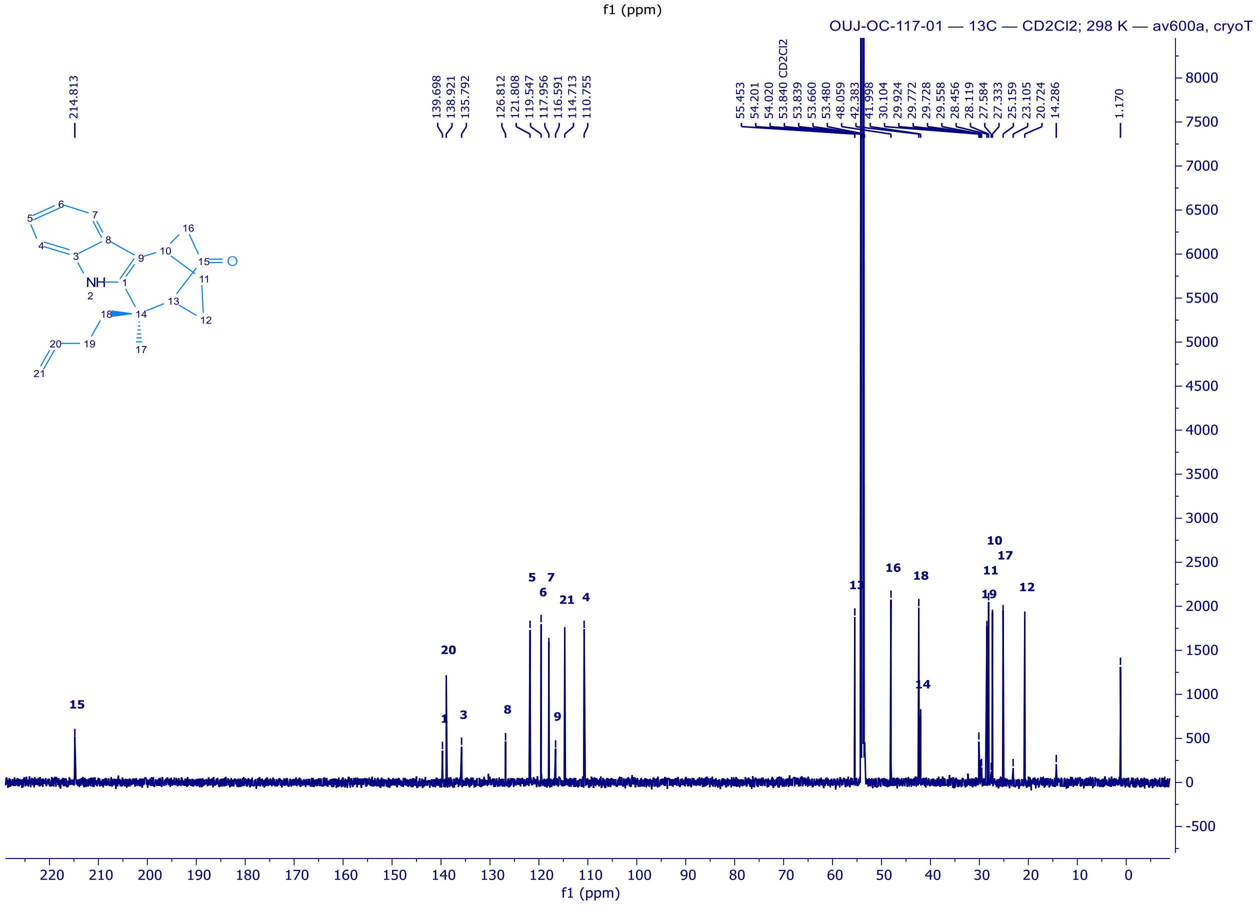
OUJ-OC-117-01 — 13C — CD2Cl2; 298 K — av600a, cryoT
f1 (ppm)

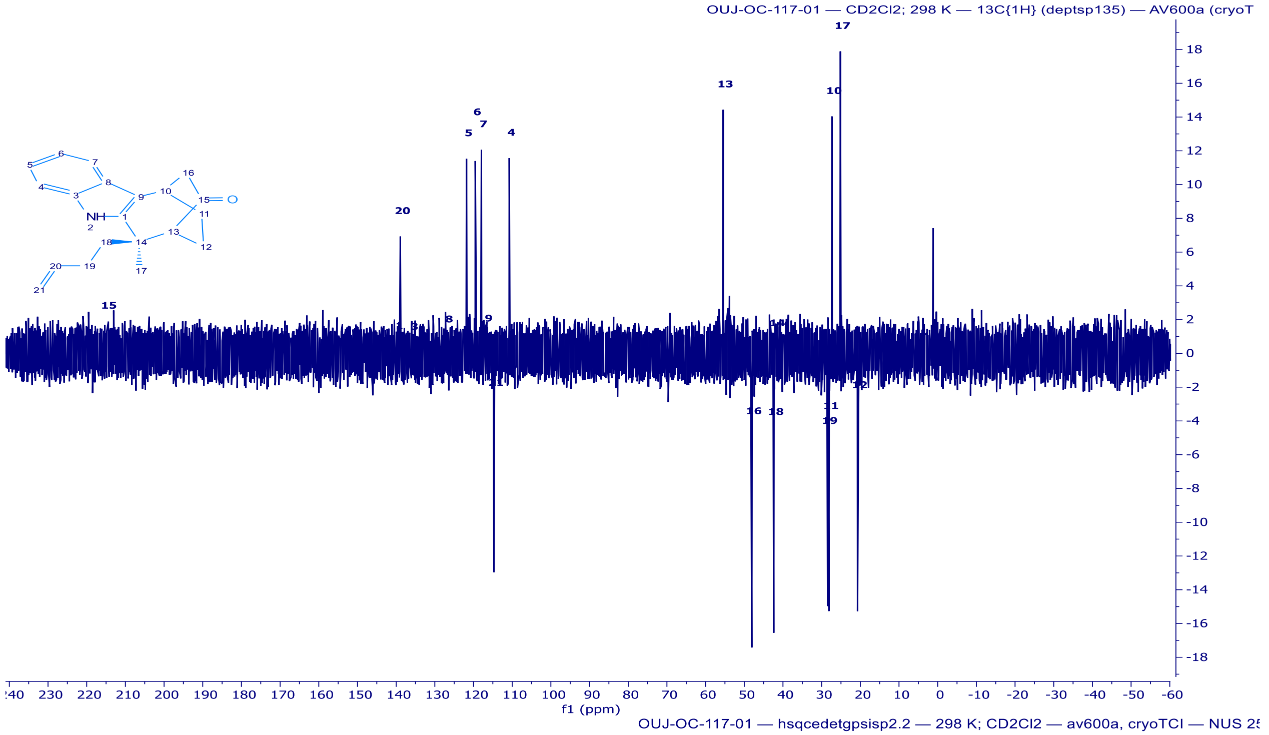
OUJ-OC-117-01 — CD2Cl2; 298 K — 13C{1H} (deptsp135) — AV600a (cryoT
f1 (ppm)

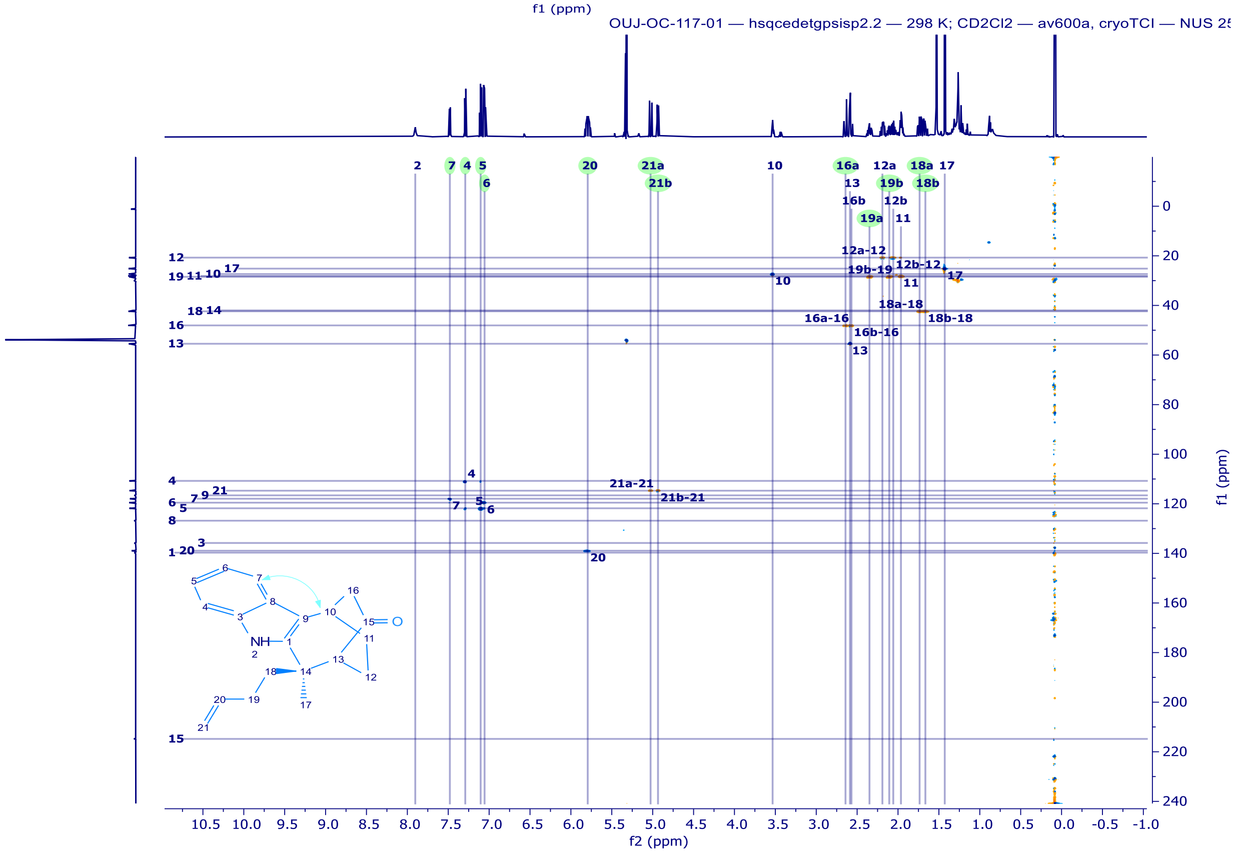
OUJ-OC-117-01 — hsqcedetgpsisp2.2 — 298 K; CD2Cl2 — av600a, cryoTCI — NUS 2!
f2 (ppm)
f1 (ppm)

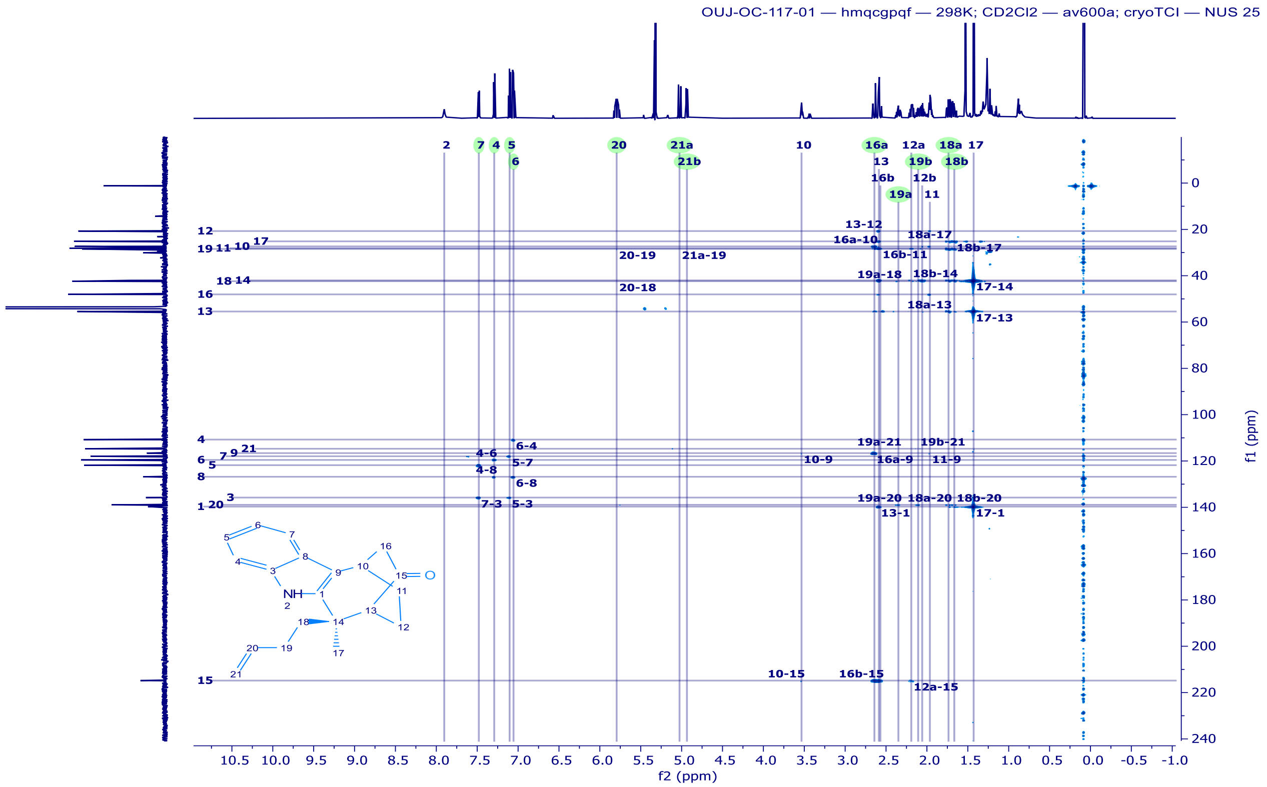
OUJ-OC-117-01 — hmqcgpqf — 298K; CD2Cl2 — av600a; cryoTCI — NUS 25
f2 (ppm)
f1 (ppm)

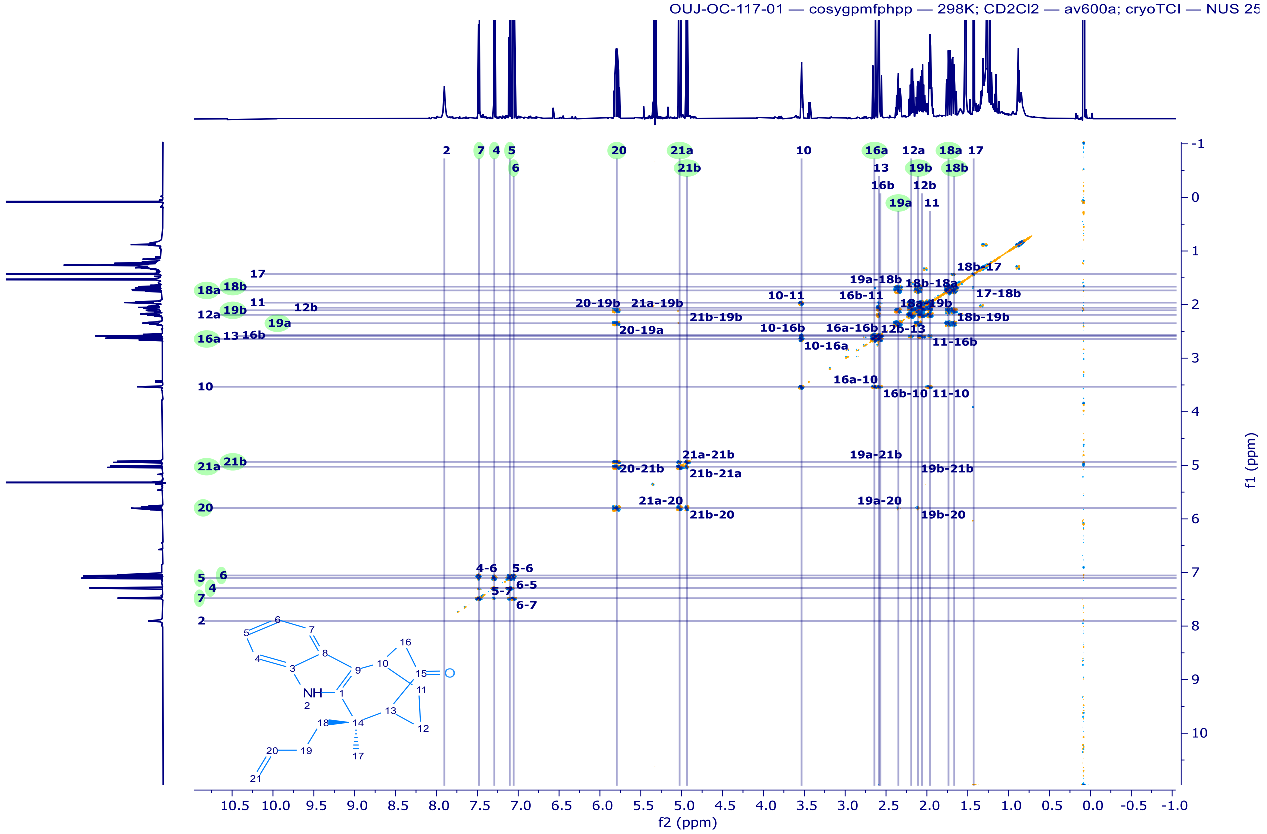
OUJ-OC-117-01 — cosygpmfphpp — 298K; CD2Cl2 — av600a; cryoTCI — NUS 25
f2 (ppm)
f1 (ppm)

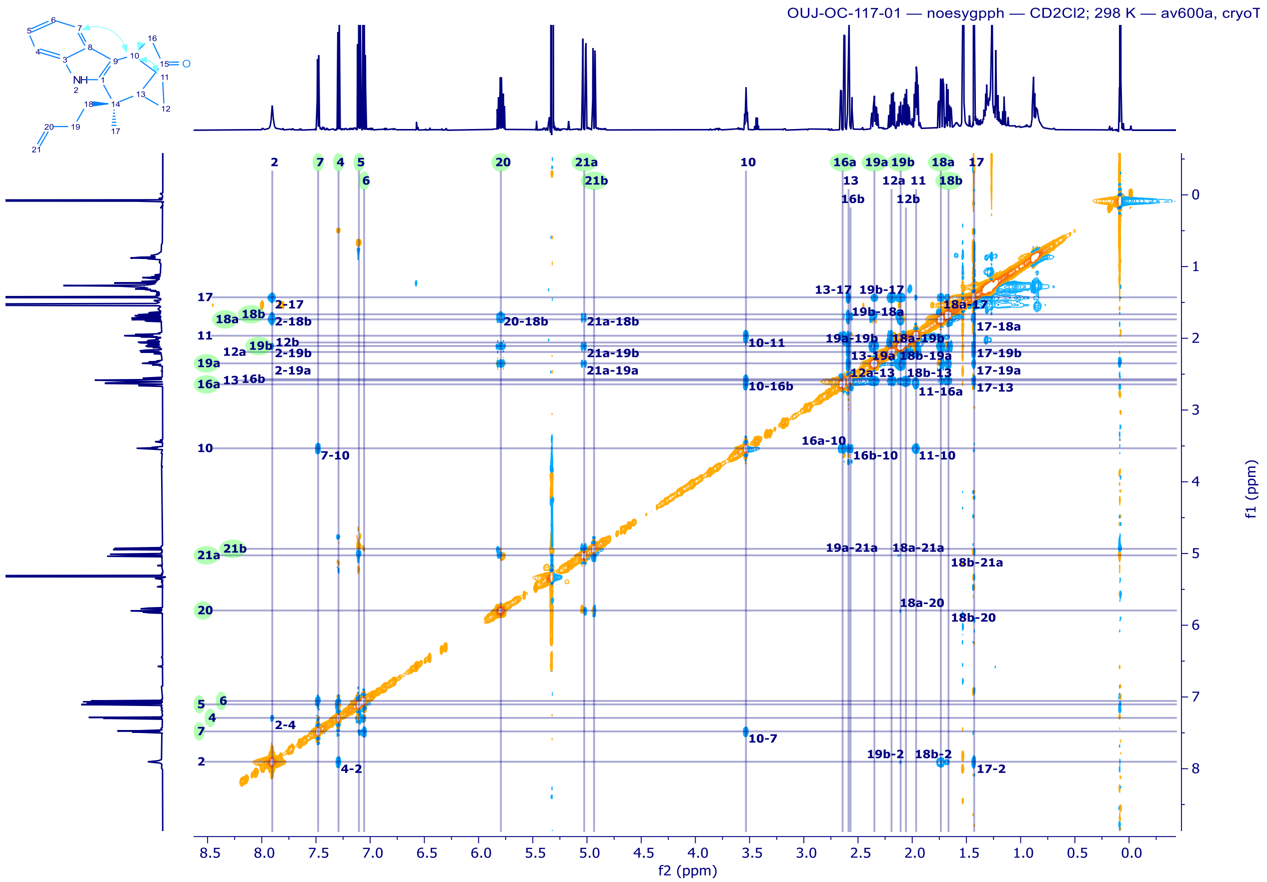

OUJ-OC-117-01 — noesygpph — CD2Cl2; 298 K — av600a, cryoT
f2 (ppm)
f1 (ppm)


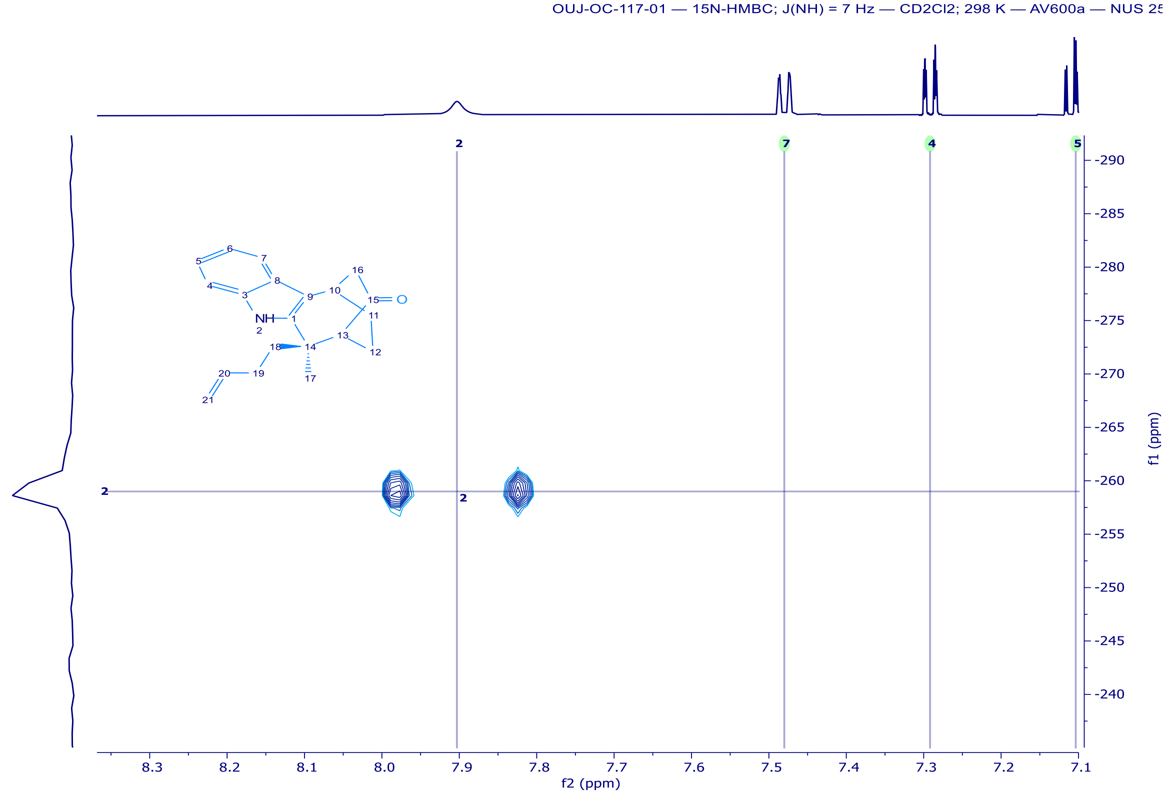

OUJ-OC-117-01 — 15N-HMBC; J(NH) = 7 Hz — CD2Cl2; 298 K — AV600a — NUS 25
f2 (ppm)
f1 (ppm)

# Characterization of 4o

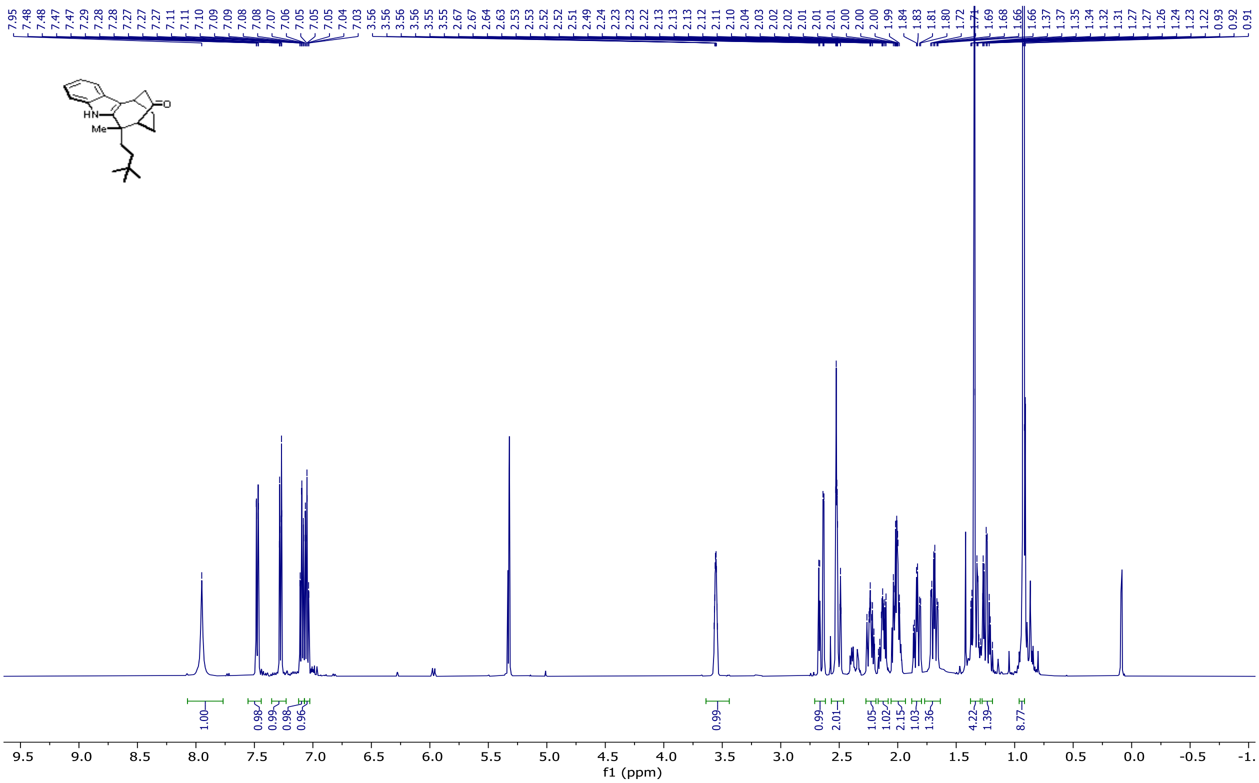


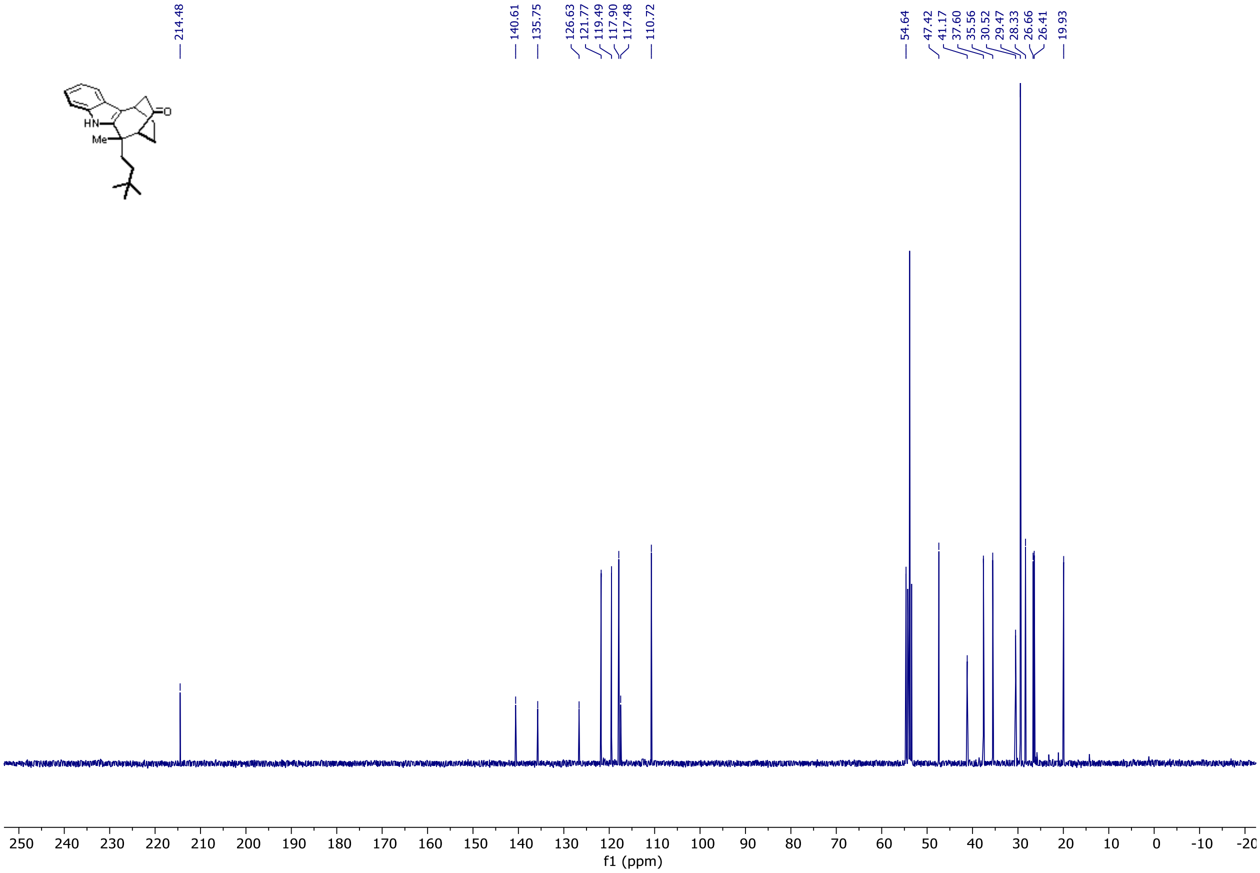

# Characterization of 4o’

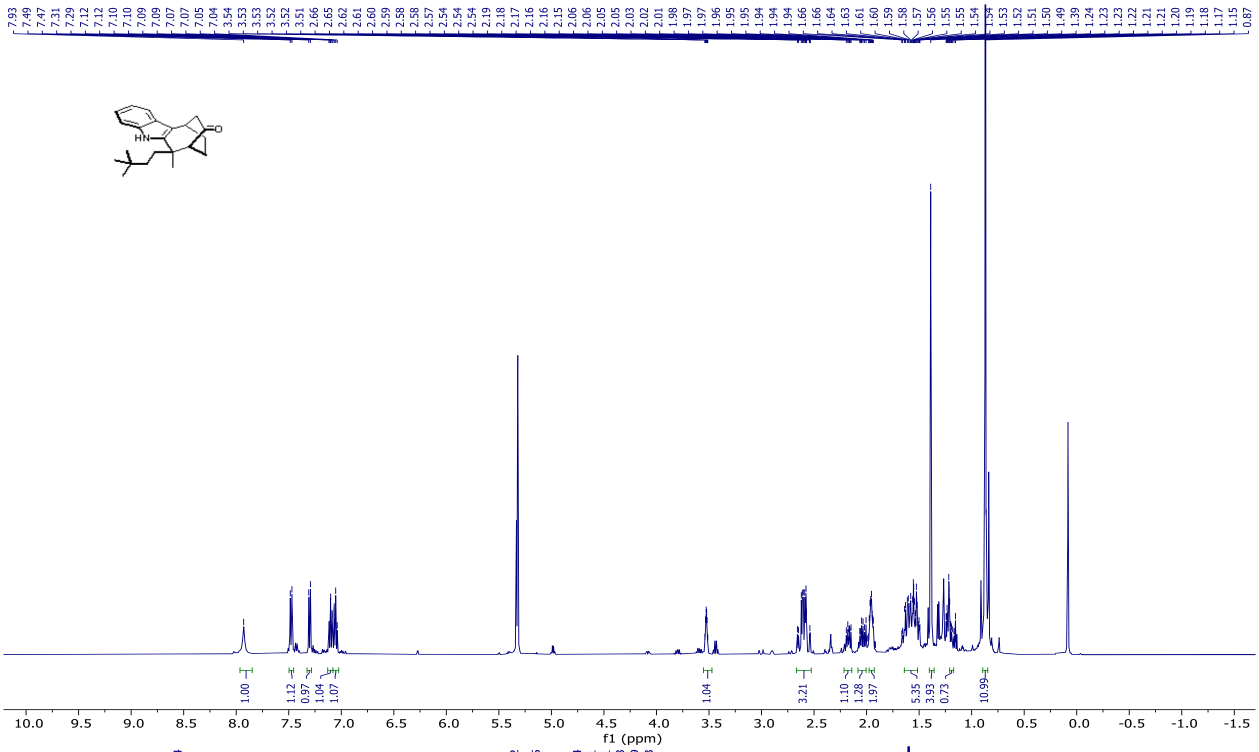


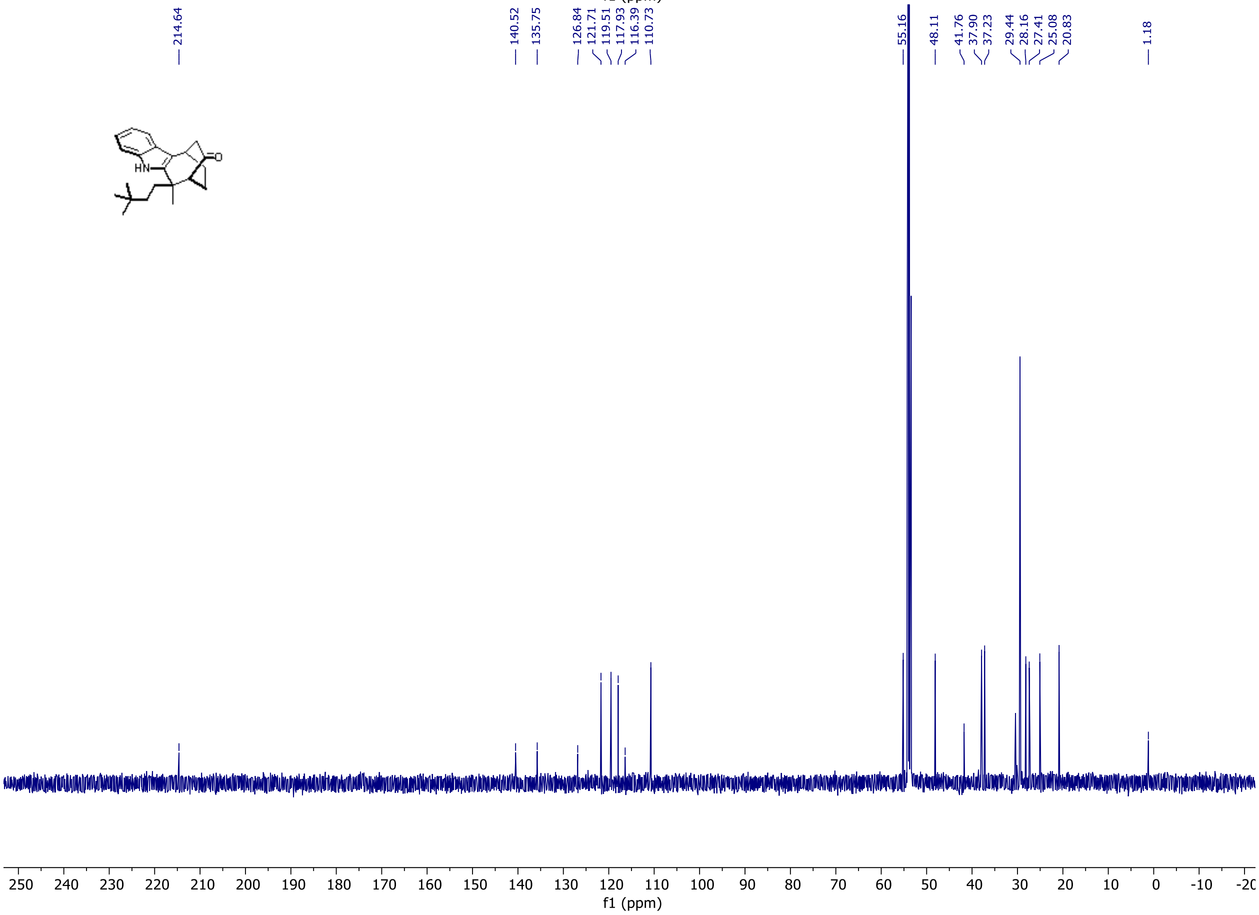

# Characterization of 4p

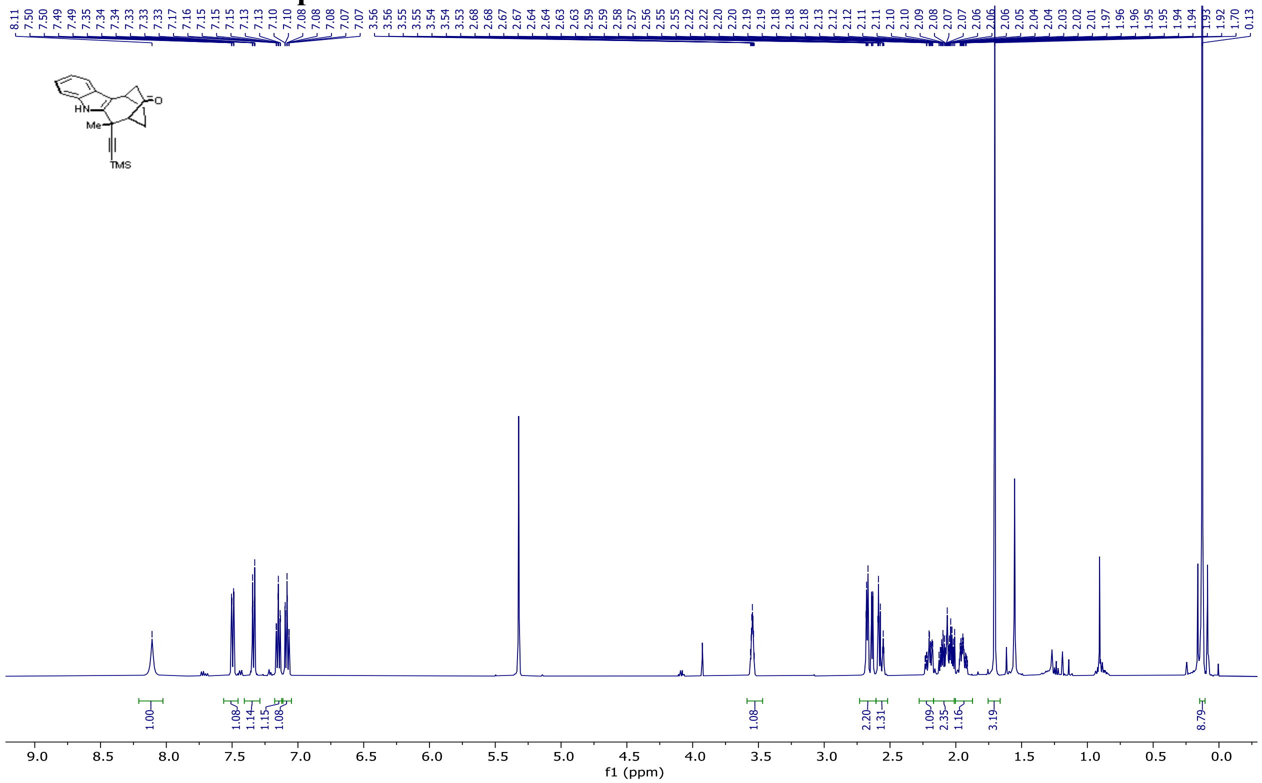


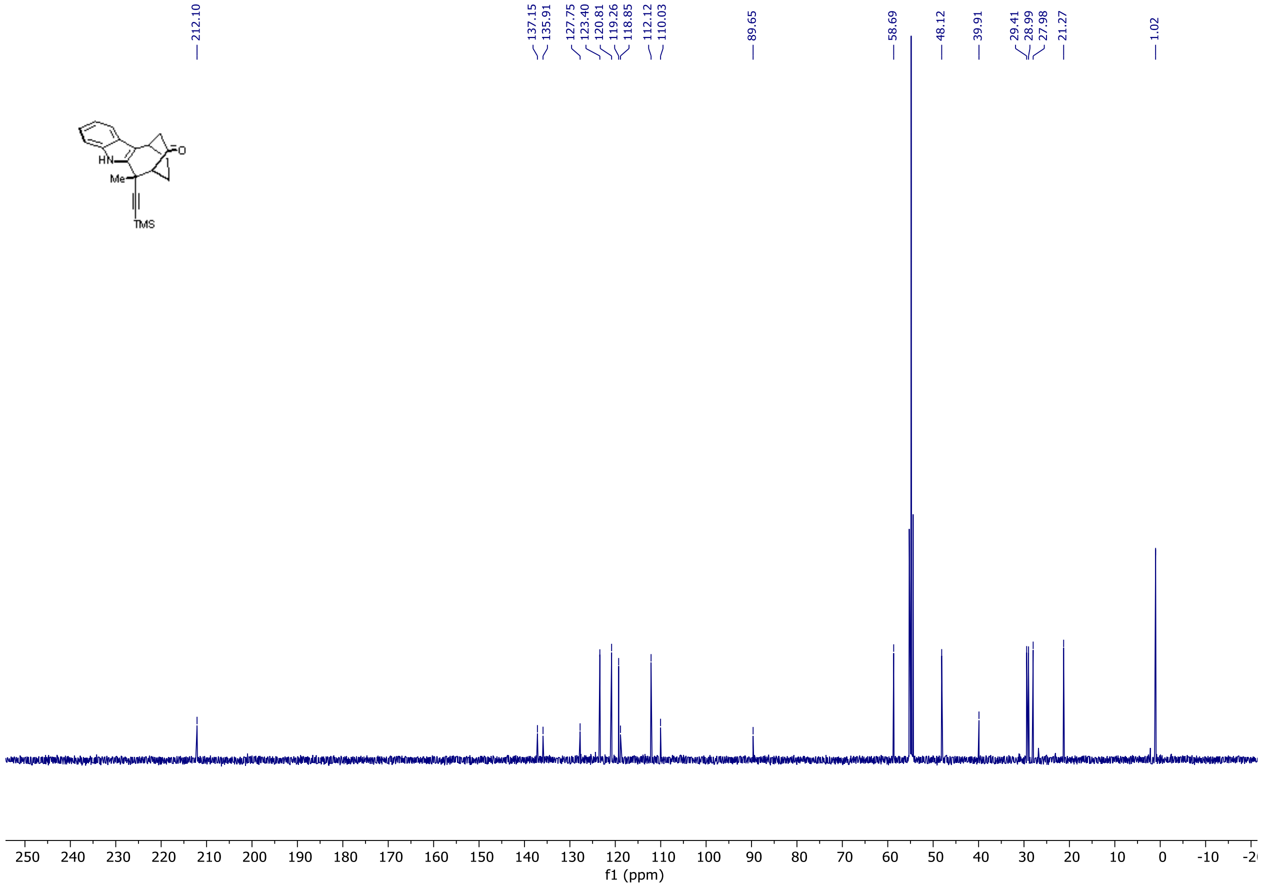

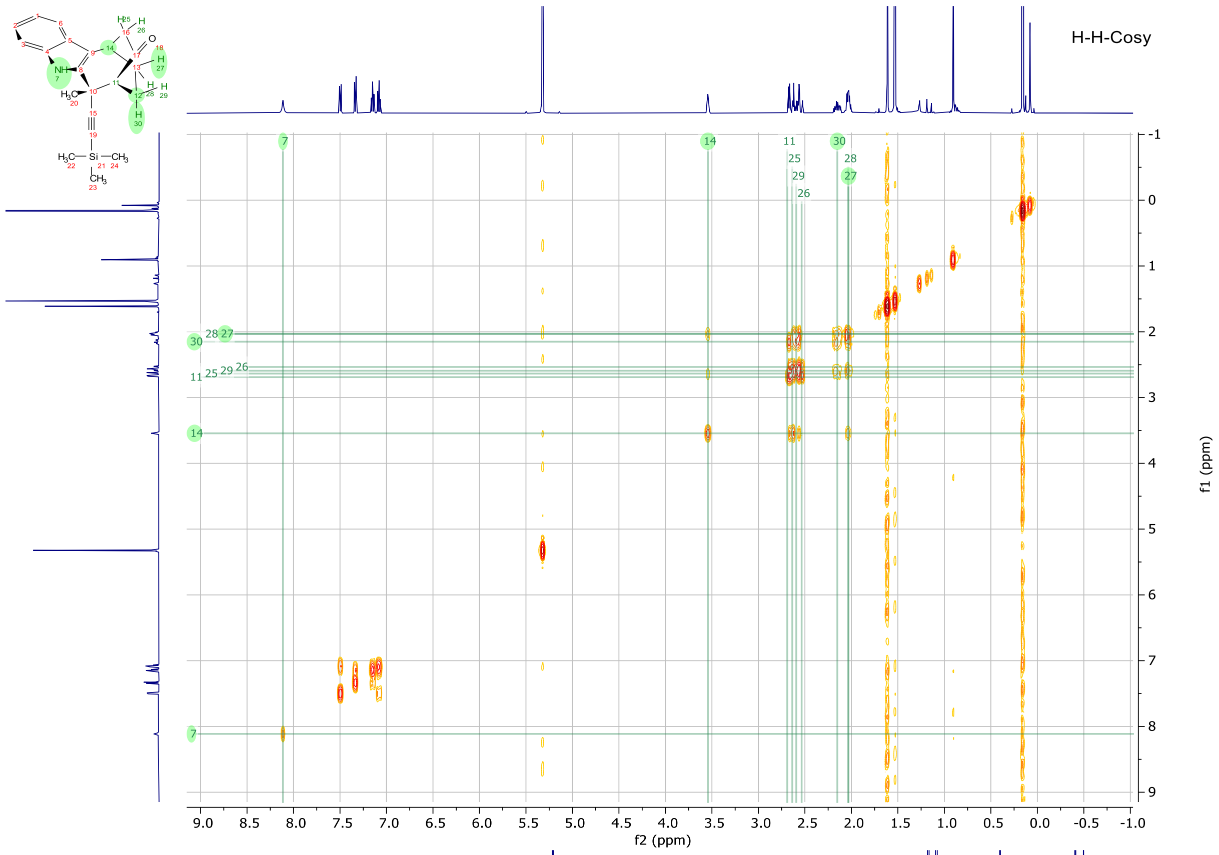
H-H-Cosy
f2 (ppm)
f1 (ppm)

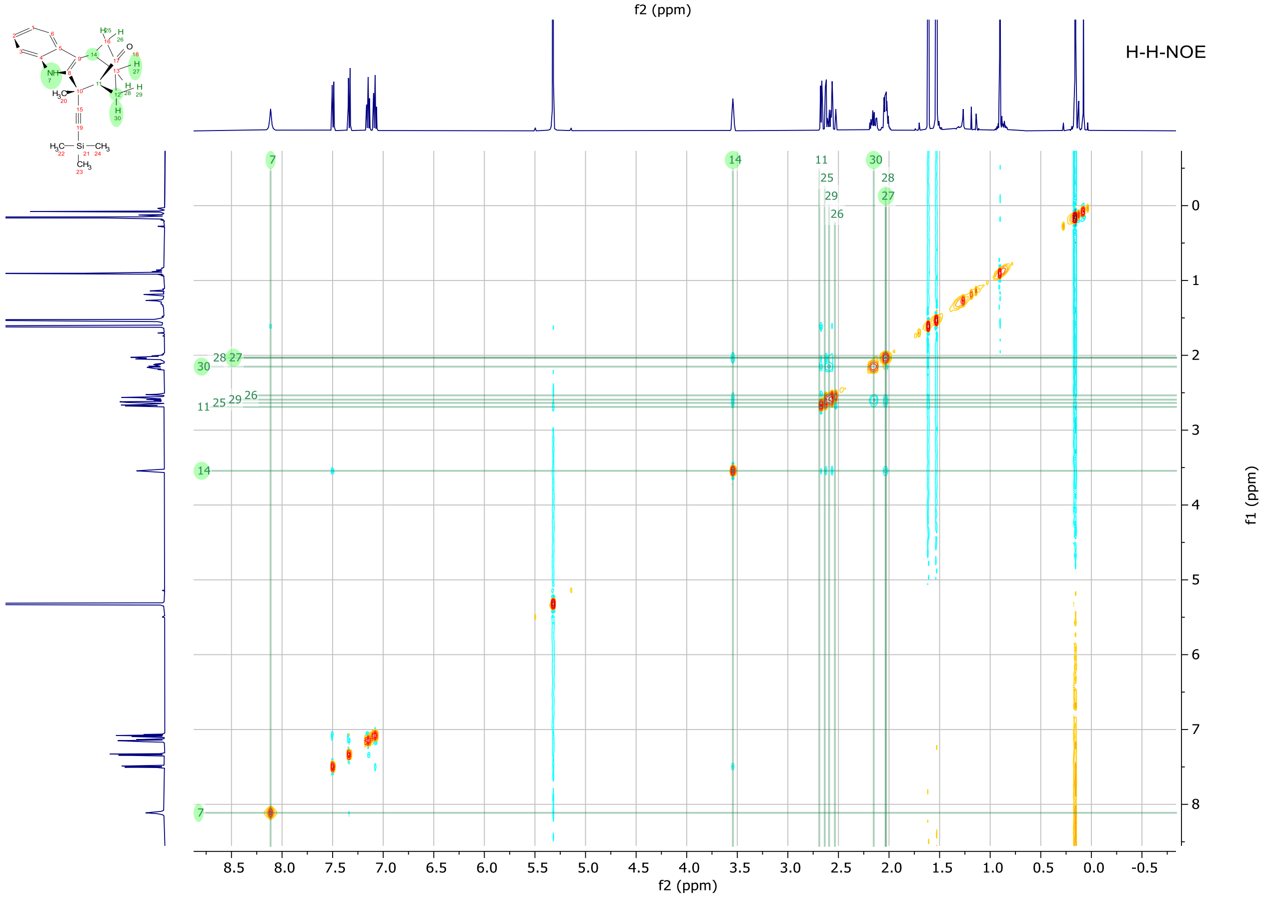
H-H-NOE
f2 (ppm)
f1 (ppm)

# Characterization of 4p’

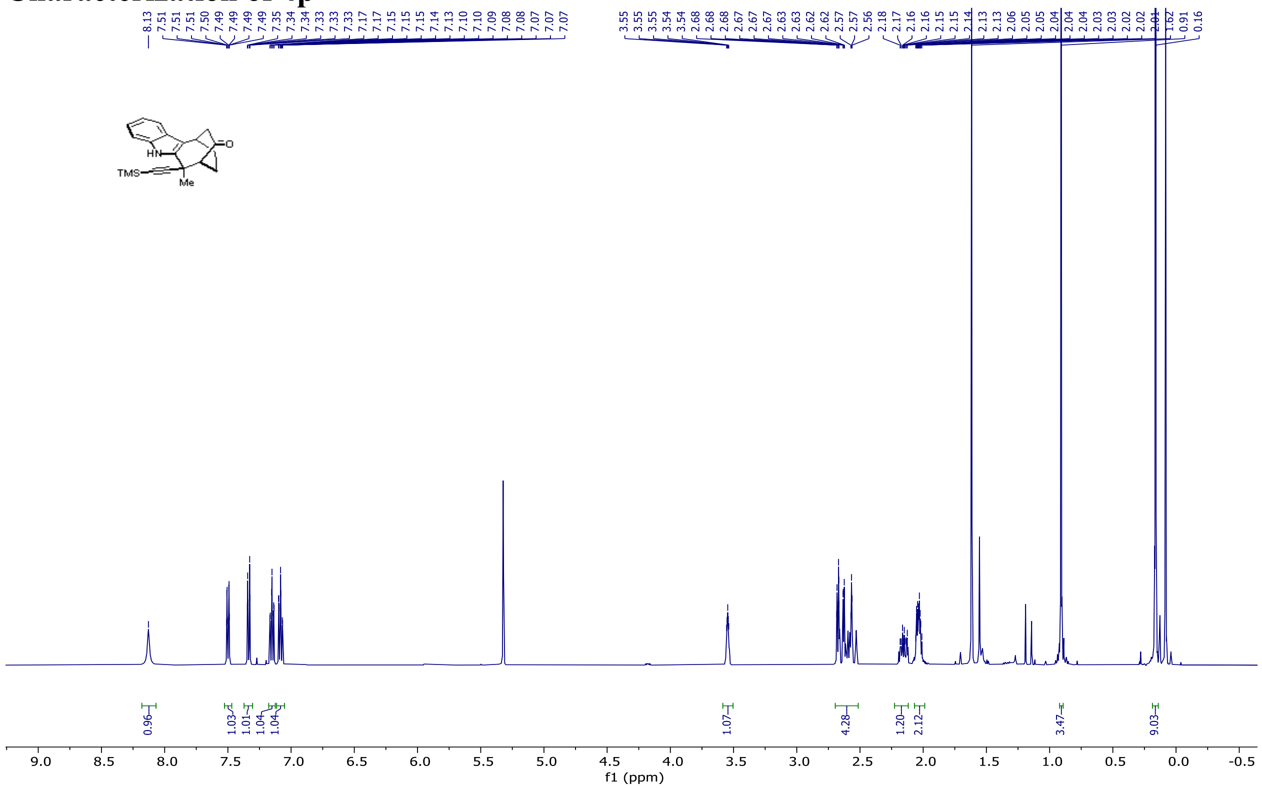


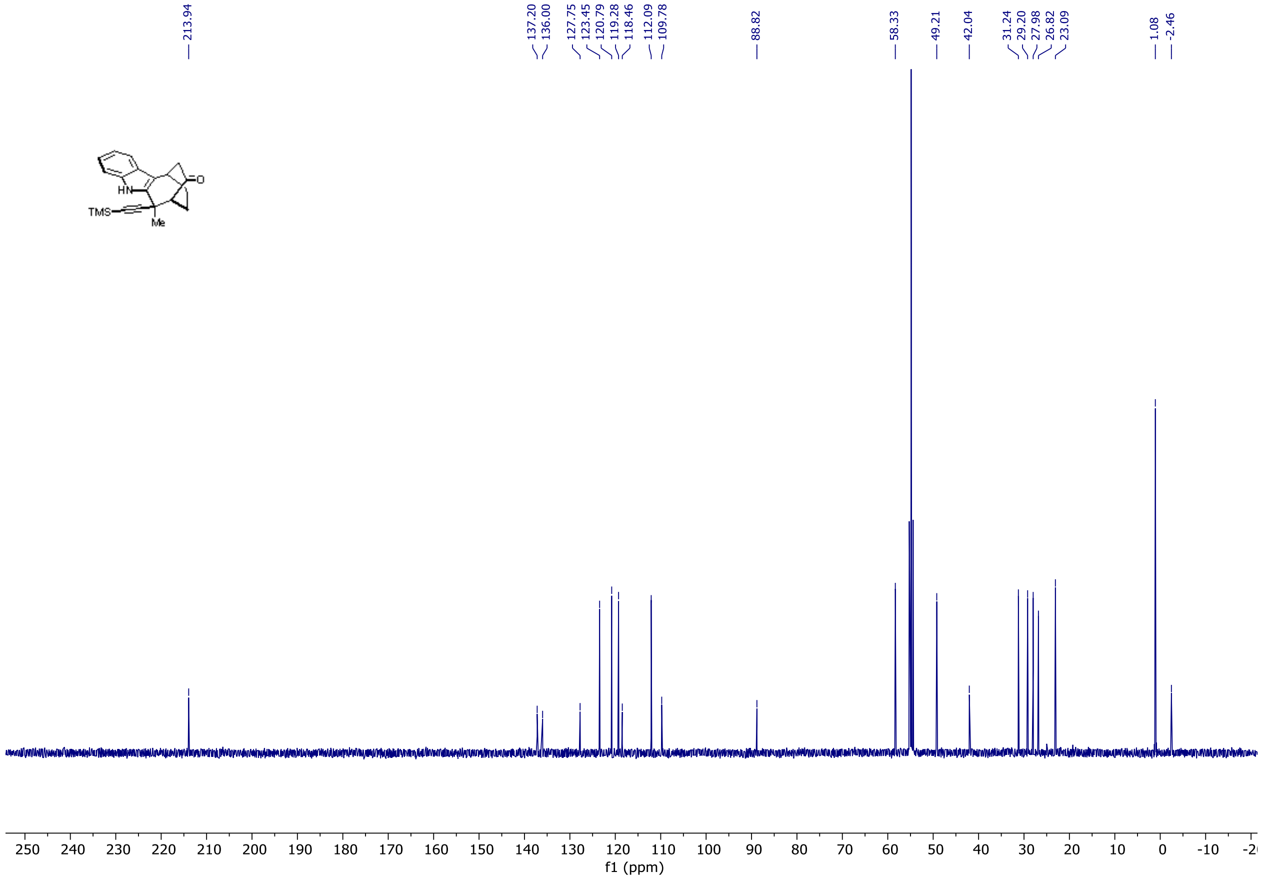

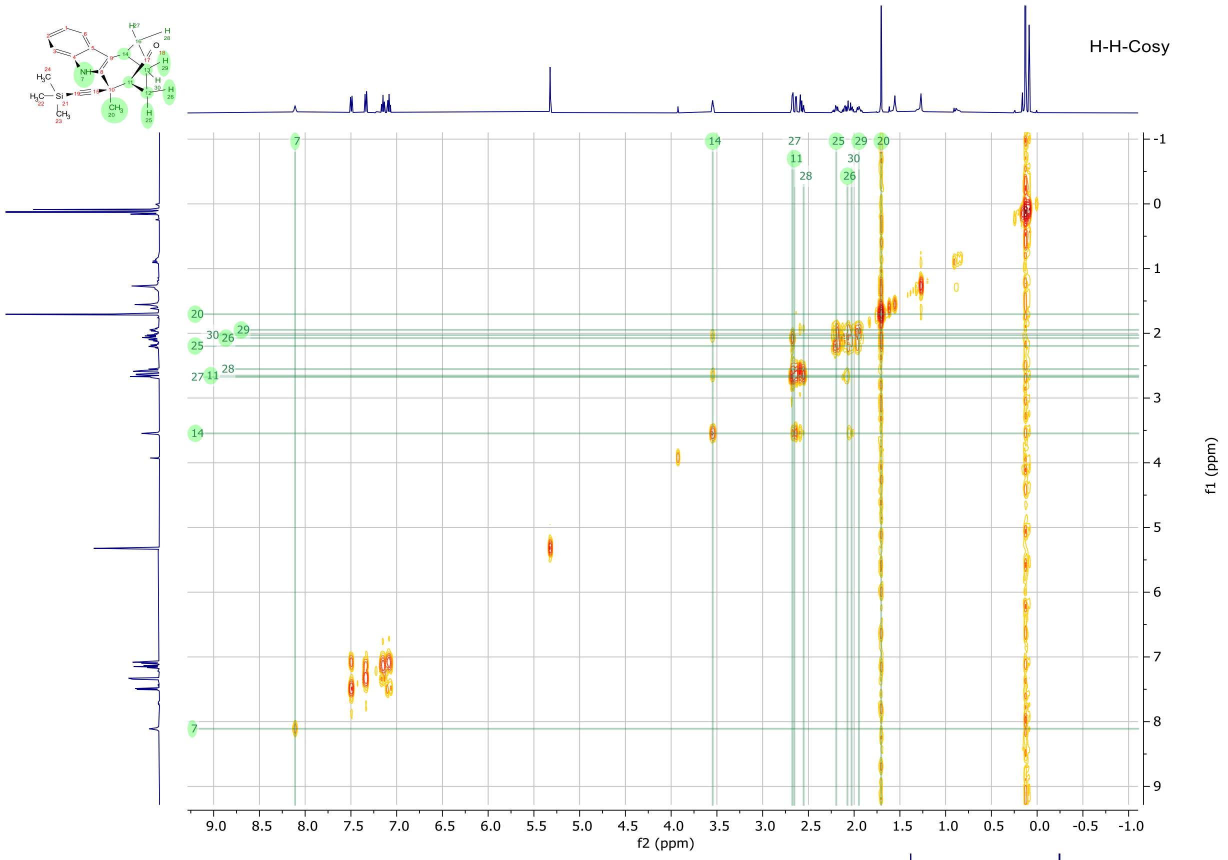
H-H-Cosy
f2 (ppm)
f1 (ppm)

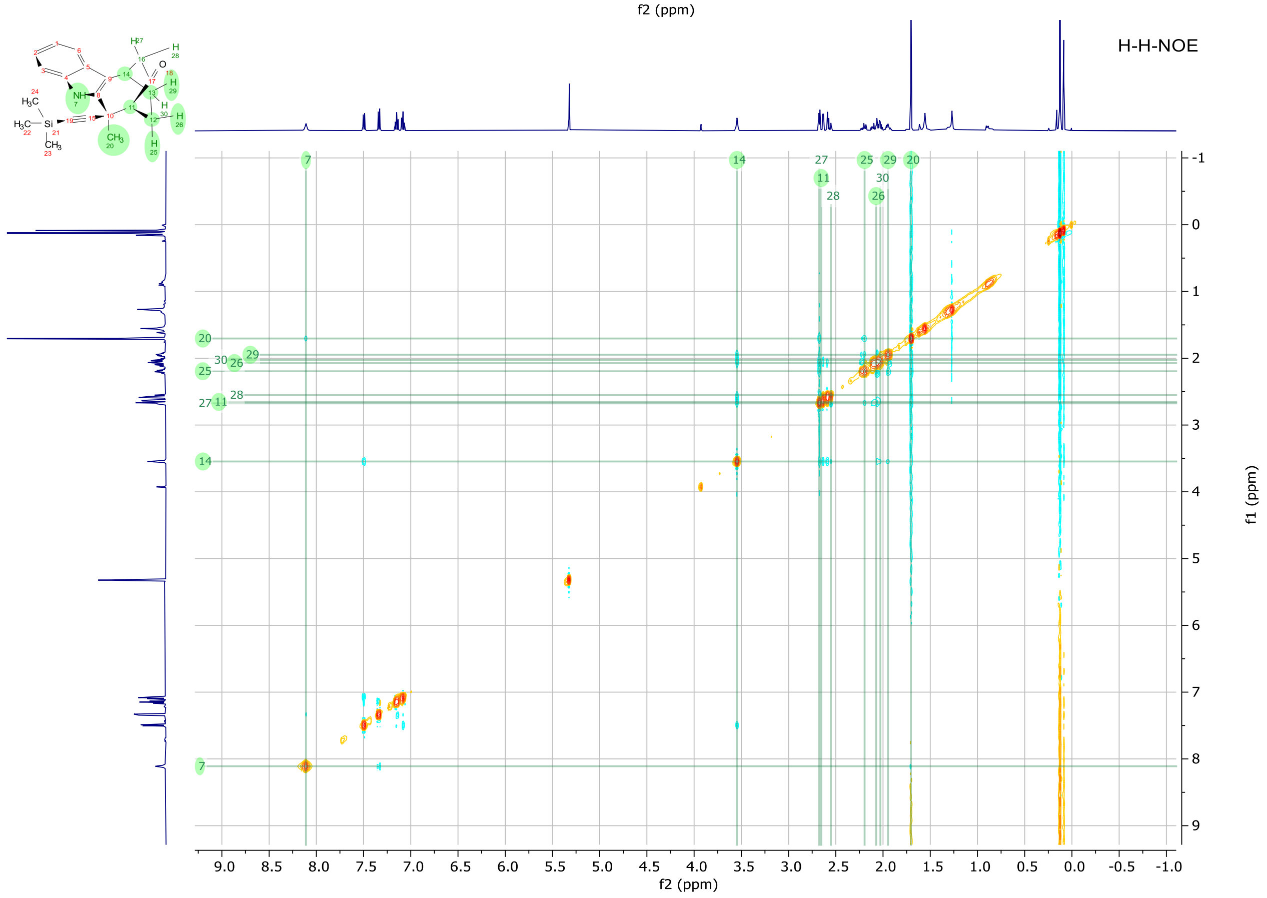
H-H-NOE
f2 (ppm)
f1 (ppm)

# Characterization of 4q

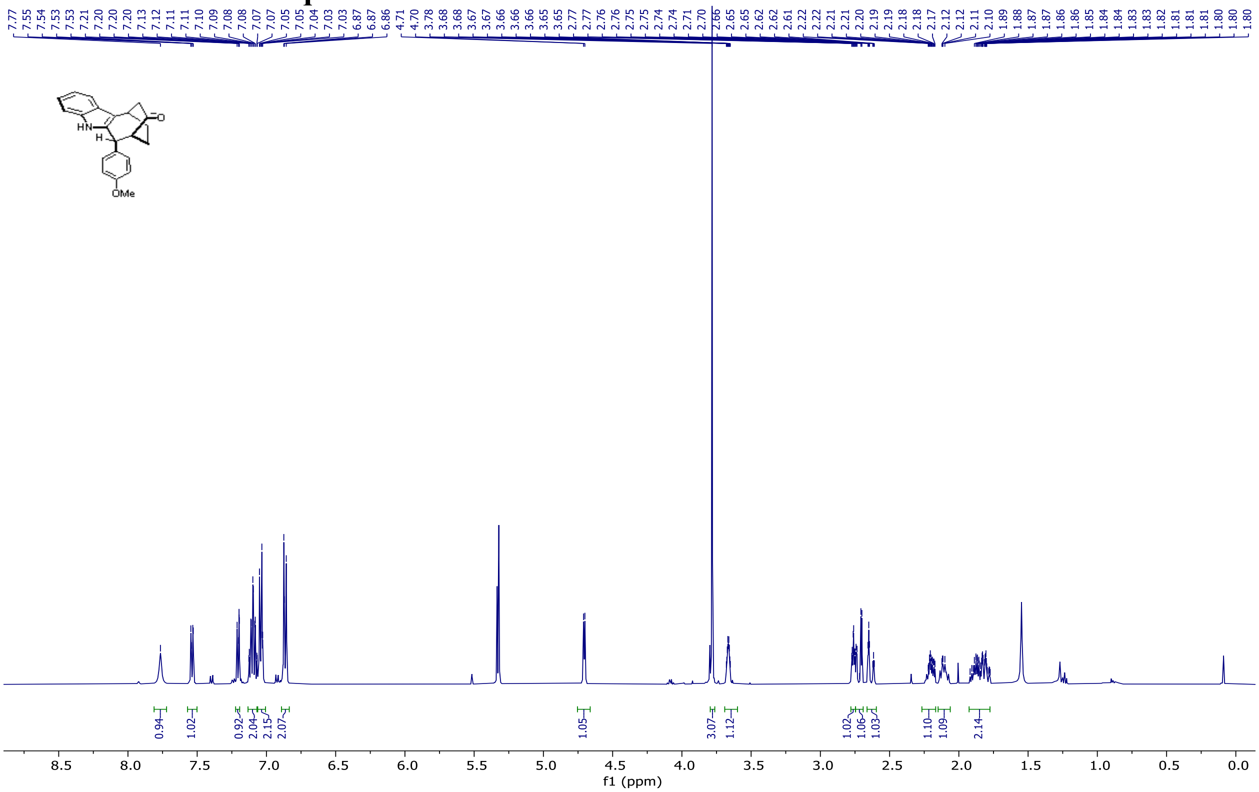


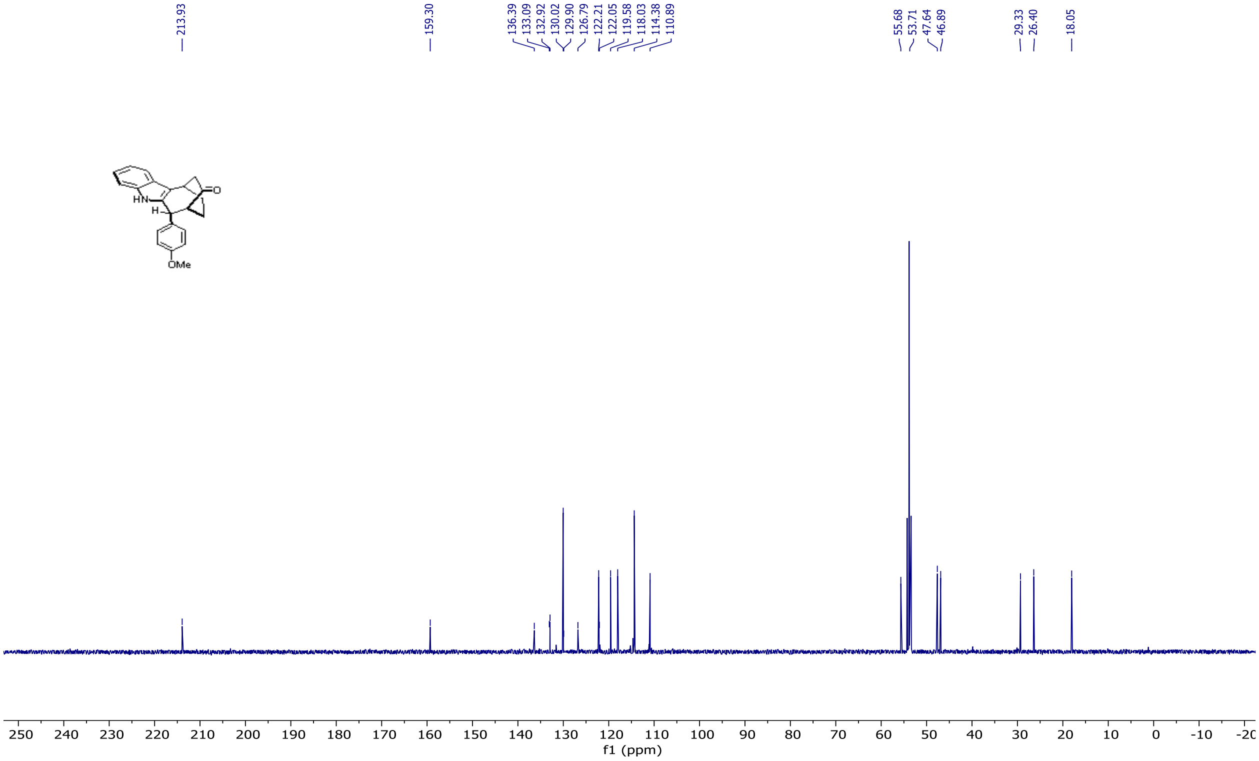

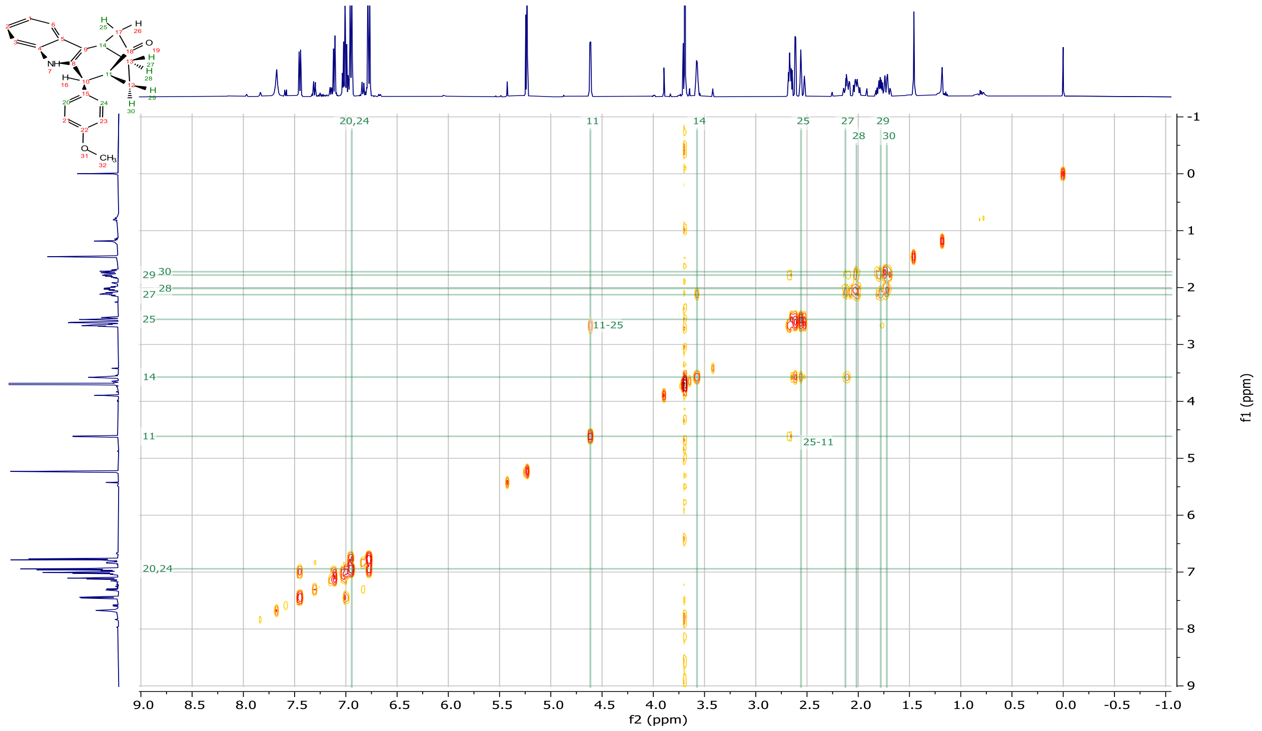

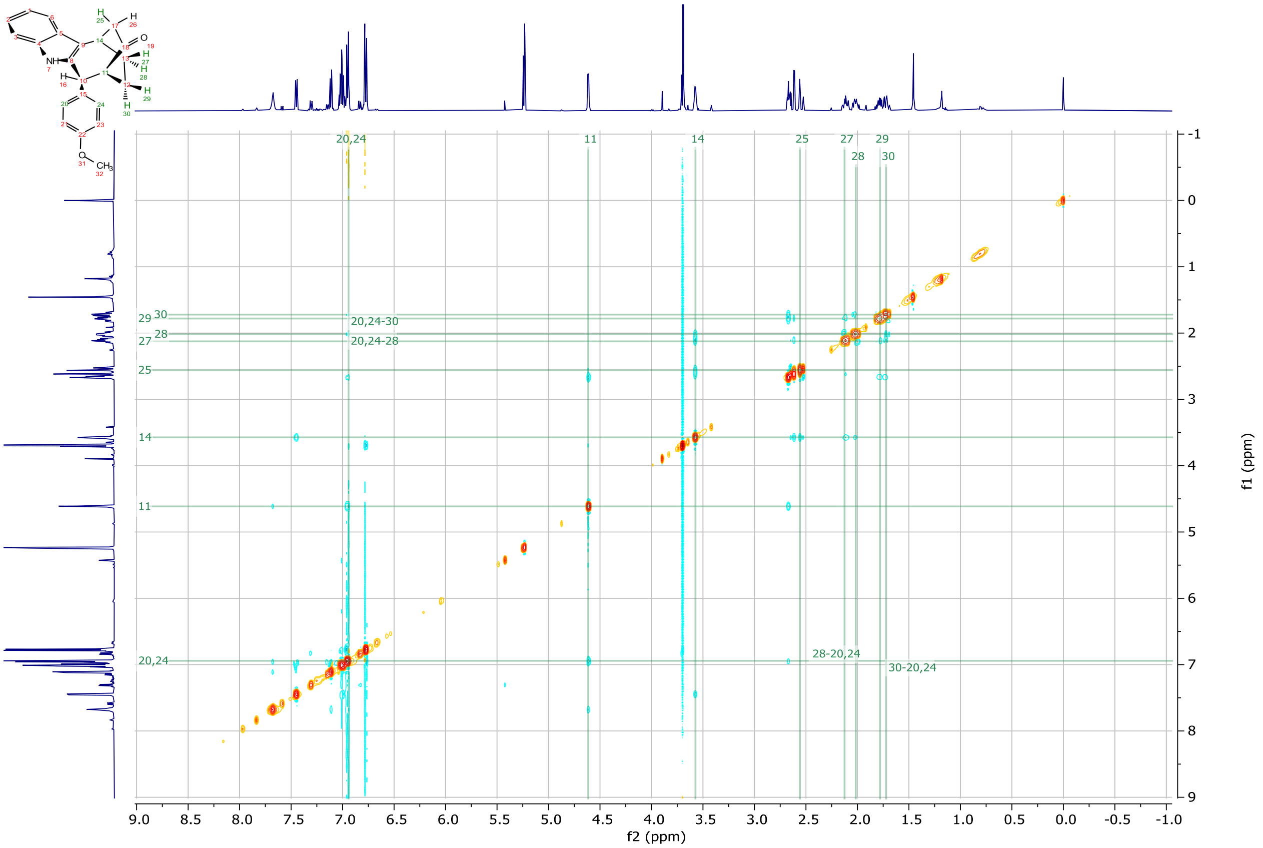

# Characterization of 4q’

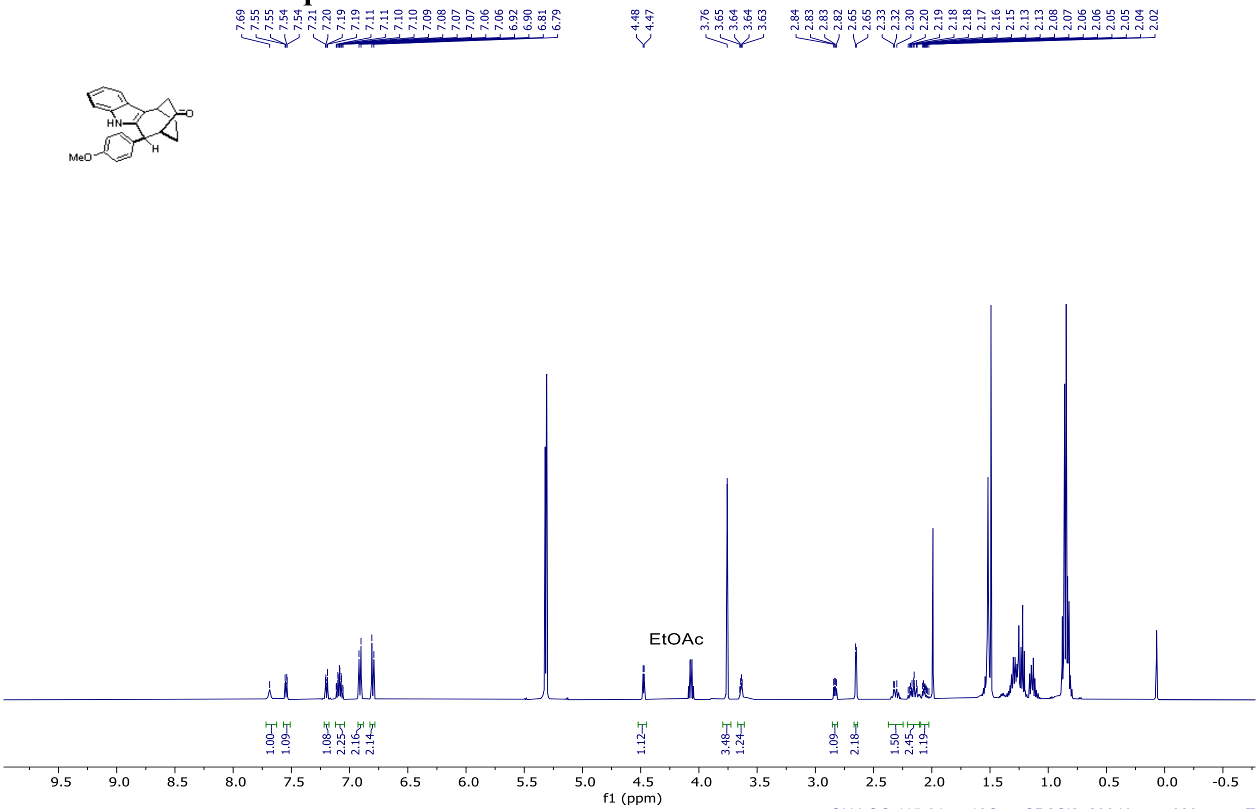


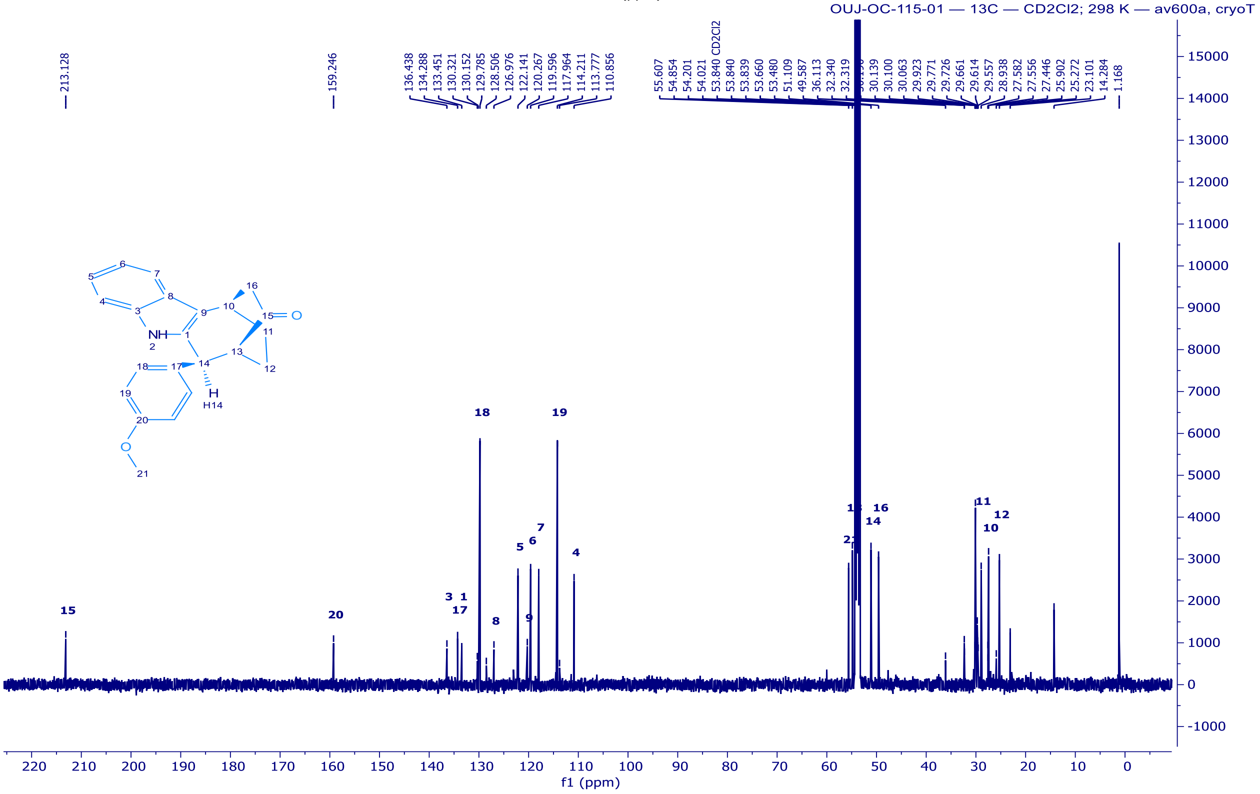

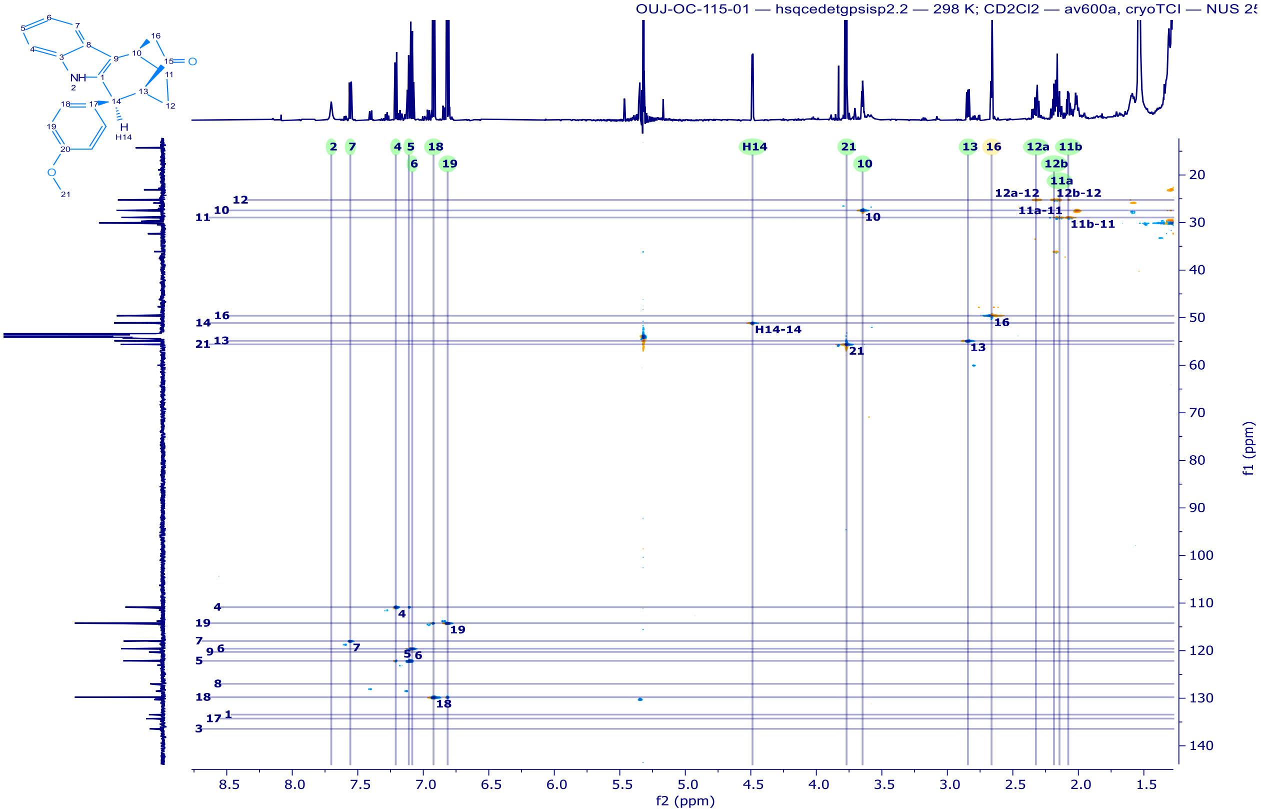
OUJ-OC-115-01 — hsqcedetgpsisp2.2 — 298 K; CD2Cl2 — av600a, cryoTCI — NUS 25
f2 (ppm)
f1 (ppm)

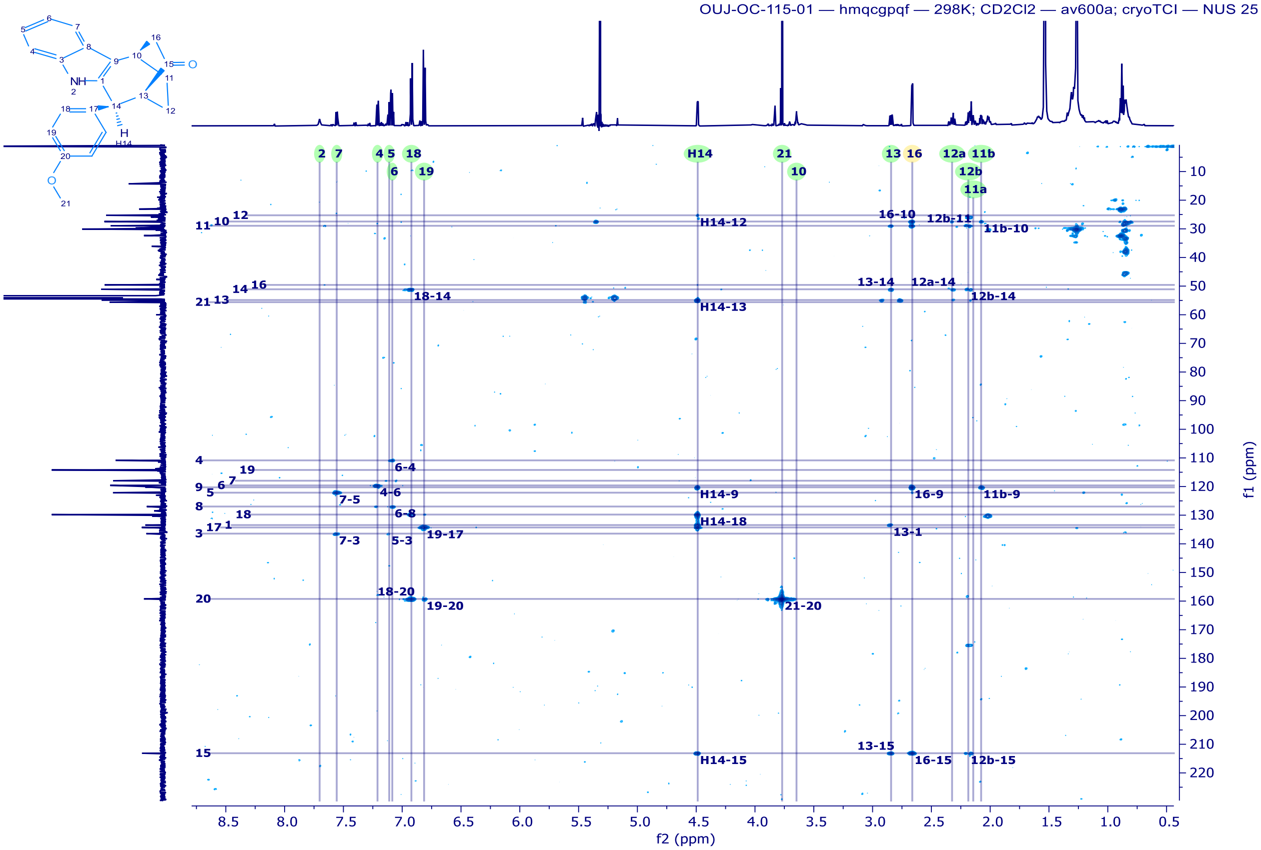
OUJ-OC-115-01 — hmqcgpqf — 298K; CD2Cl2 — av600a; cryoTCI — NUS 25
f2 (ppm)
f1 (ppm)

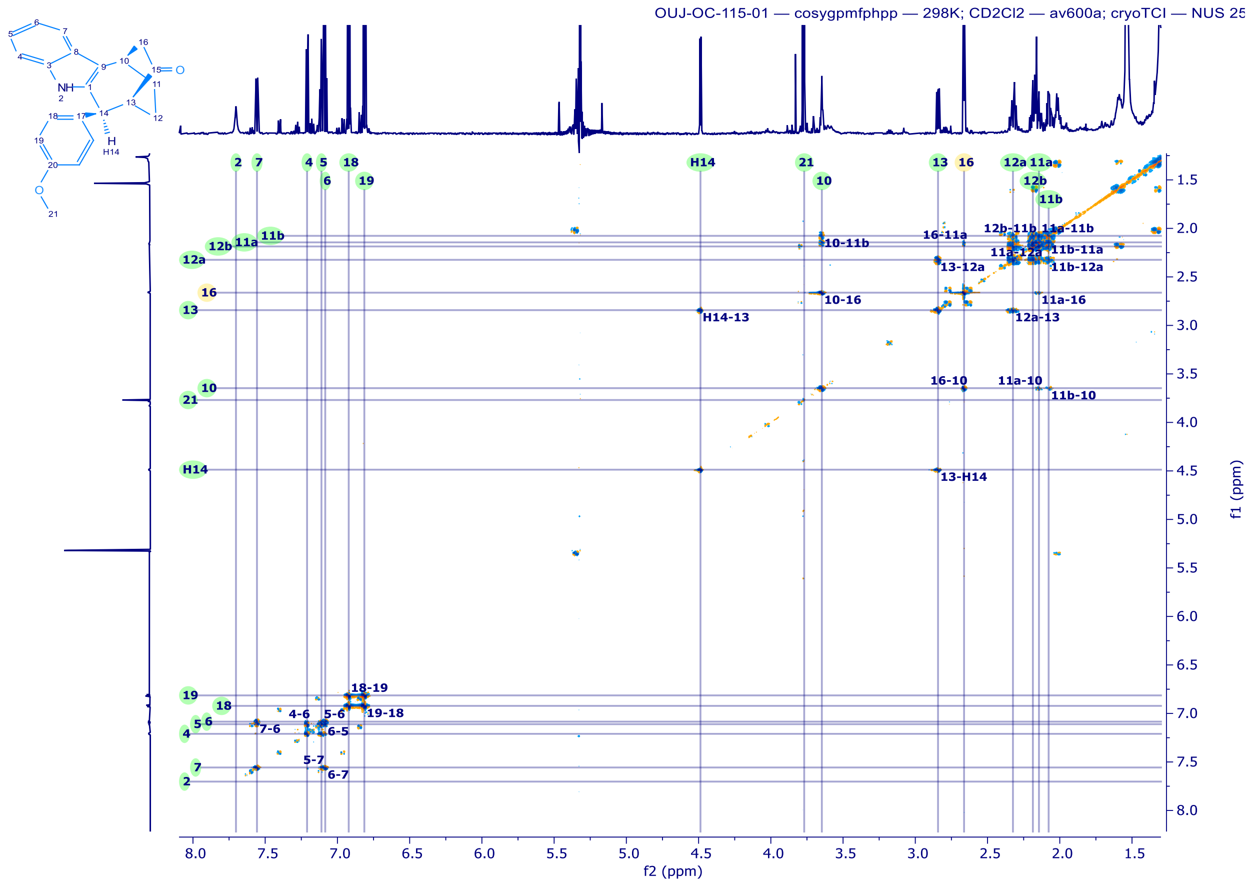
OUJ-OC-115-01 — cosygpmfphpp — 298K; CD2Cl2 — av600a; cryoTCI — NUS 25
NH
H14
f2 (ppm)
f1 (ppm)
10-11b
16-11a
12b-11b
11a-11b
11a-12a
11b-11a
13-12a
11b-12a
10-16
11a-16
H14-13
12a-13
16-10
11a-10
11b-10
13-H14
18-19
19-18
4-6
5-6
7-6
6-5
5-7
6-7

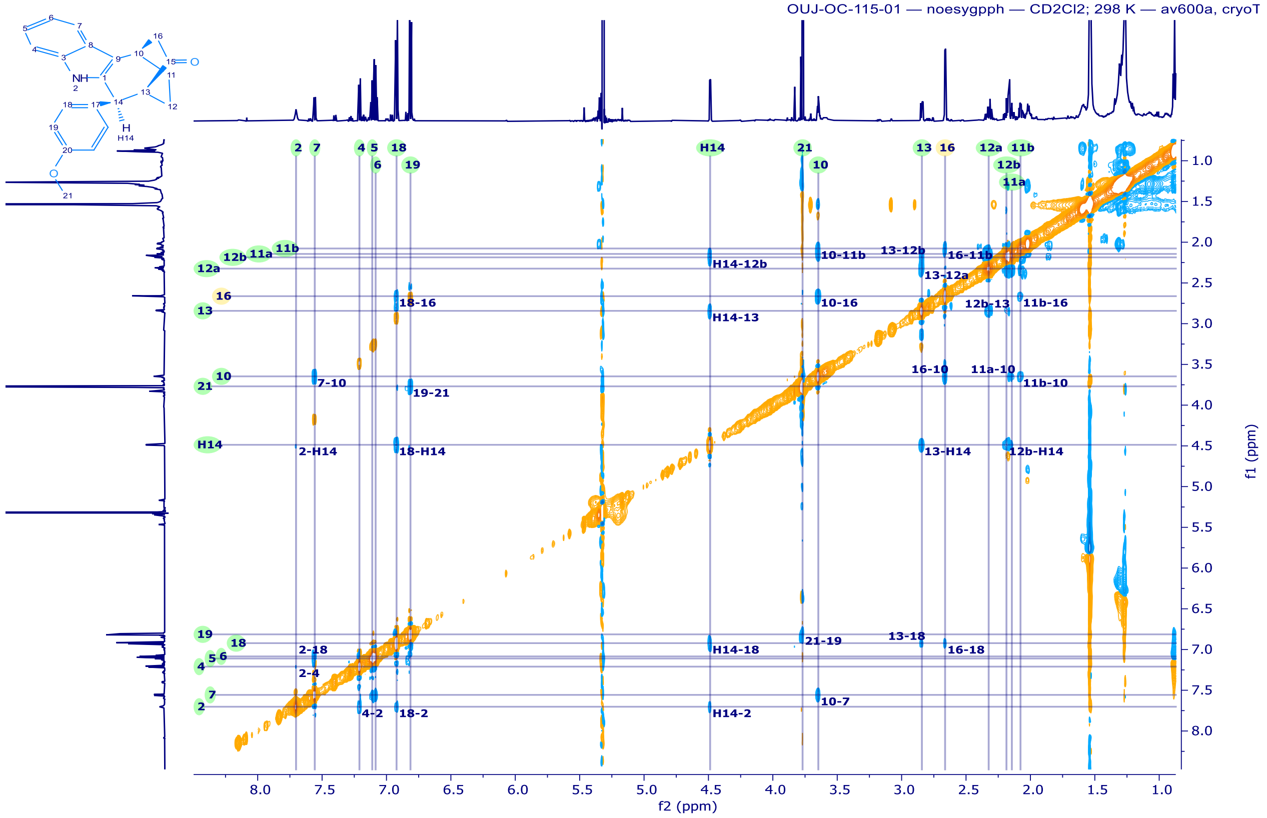
OUJ-OC-115-01 — noesygpph — CD2Cl2; 298 K — av600a, cryoT
NH
H14
f2 (ppm)
f1 (ppm)
H14-12b
10-11b
13-12b
16-11b
13-12a
18-16
10-16
12b-13
11b-16
H14-13
16-10
11a-10
7-10
11b-10
19-21
2-H14
18-H14
13-H14
12b-H14
2-18
H14-18
21-19
13-18
16-18
2-4
4-2
18-2
H14-2
10-7

NMR spectra for compound **8**: $^{1}$H (501 MHz) and $^{13}$C (126 MHz) in $CD_2Cl_2$.

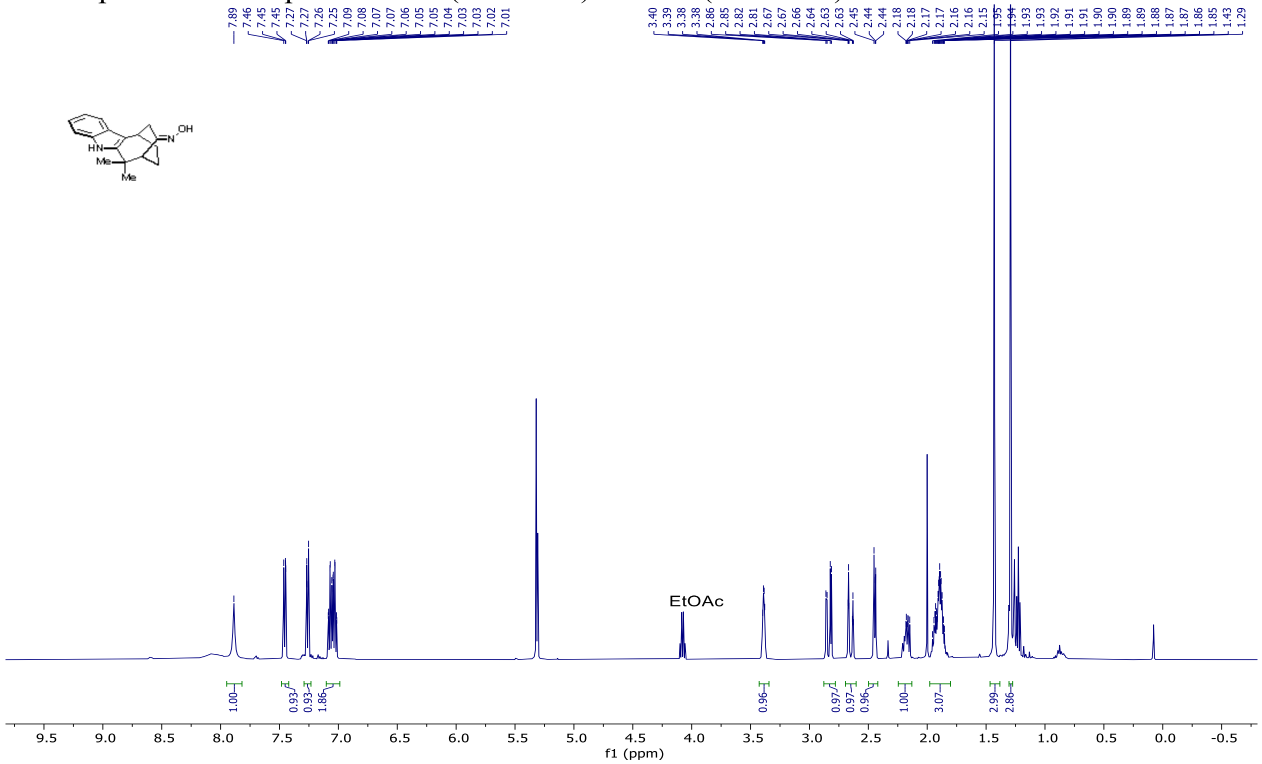


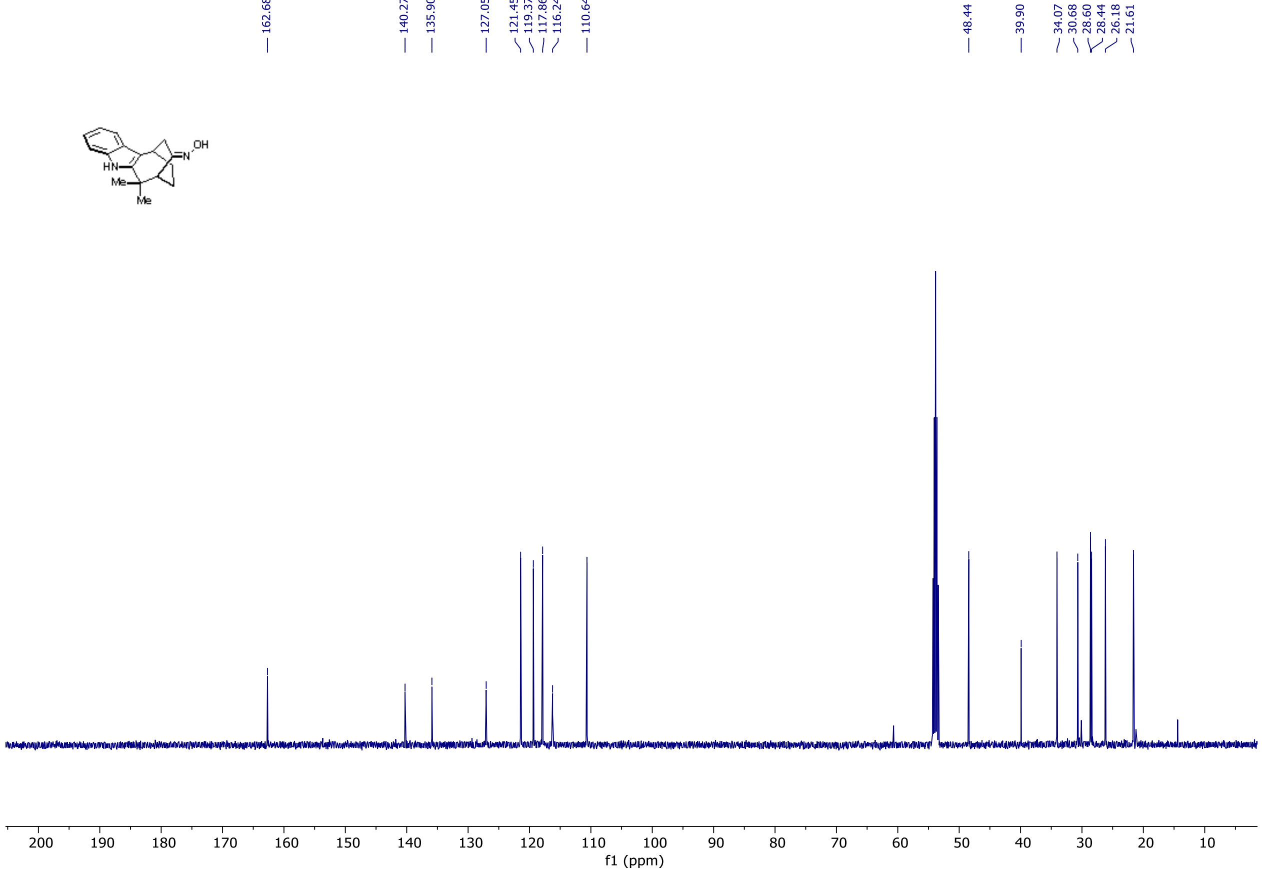

# Characterization of 9

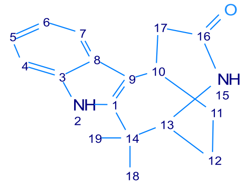


| Atom | δ (ppm) | J | HSQC | HMQC | COSY | NOESY | $^{15}$N-HMBC |
|---|---|---|---|---|---|---|---|
| C1 | 139.50 | | | 10, 13, 18, 19 | | | |
| N2 | -258.0 | | | | | | 2 |
| H2 | 7.911 | (br) s | | | | 4, 19 | 2 |
| C3 | 135.68 | | | 5, 7 | | | |
| C4 | 110.64 | | 4 | 6 | | | |
| H4 | 7.314 | d 8.0(5), t 0.9(6, 7) | 4 | 6, 8 | 5, 6, 7 | 2 | |
| C5 | 121.70 | | 5 | 7 | | | |
| H5 | 7.112 | d1.2(7), d 8.0(4), d 7.1(6) | 5 | 3, 7 | 4, 6, 7 | | |
| C6 | 119.62 | | 6 | 4 | | | |
| H6 | 7.060 | d7.1(5), d 1.1(4), d 7.9(7) | 6 | 4, 8 | 4, 5, 7 | | |
| C7 | 117.77 | | 7 | 5 | | | |
| H7 | 7.440 | d 1.2(5), t 0.7(4, ?), d 7.9(6) | 7 | 3, 5, 9 | 4, 5, 6 | 10, 17b | |
| C8 | 127.81 | | | 4, 6 | | | |
| C9 | 114.90 | | | 7, 10, 11a, 11b, 17a, 17b | | | |
| C10 | 28.92 | | 10 | 11a, 12a, 17a, 17b | | | |
| H10 | 3.514 | t 6.0(11a, 17b), d 2.0(11b), d 2.5(17a), d 0.7(12a?) | 10 | 1, 9 | 11a, 11b, 17a, 17b | 7, 11a, 11b, 17a, 17b | |
| C11 | 31.90 | | 11a, 11b | 12a, 12b, 13, 17a, 17b | | | |
| H11a | 2.190 | d 8.5(12b), d 14.0(11b), d 6.0(10), d 1.7(12a) | 11 | 9, 10, 12, 13 | 10, 11b, 12a, 12b | 10, 17a | |
| H11b | 2.127 | d 11.5(12b), d 14.0(11a), d 2.0(10), d 1.5(17b), d 8.7(12a) | 11 | 9, 17 | 10, 11a, 12a, 12b | 10, 18 | |
| C12 | 28.72 | | 12a, 12b | 11a, 13 | | | |
| H12a | 2.474 | d 6.0(13), d 15.5(12b), d 1.7(11a), d 8.7(11b), t 0.9(10?, 15?) | 12 | 10, 11, 13 | 11a, 11b, 12b, 13 | 13, 18 | 15 |
| H12b | 2.420 | d 2.3(13), d 11.5(11b), d 8.5(11a), d 15.5(12a) | 12 | 11, 14 | 11a, 11b, 12a, 13 | 13, 15, 17a | |
| C13 | 61.78 | | 13 | 11a, 12a, 18, 19 | | | |
| H13 | 3.218 | d 6.7(15), d 2.3(12b), d 6.0(12a) | 13 | 1, 11, 12, 14, 16 | 12a, 12b, 15 | 12a, 12b, 15, 18, 19 | |
| C14 | 41.71 | | | 12b, 13, 18, 19 | | | |
| N15 | -254.5 | | | | | | 12a, 15, 17b |
| H15 | 6.301 | (br) d 6.7(13) | | | 13, 17b | 12b, 13, 17a, 19 | 15 |
| C16 | 175.31 | | | 13, 17a, 17b | | | |
| C17 | 41.05 | | 17a, 17b | 11b | | | |
| H17a | 2.871 | d 2.5(10), d 17.5(17b) | 17 | 9, 10, 11, 16 | 10, 17b | 10, 11a, 12b, 15 | |
| H17b | 2.824 | d 1.5(11b), d 6.0(10), d 17.5(17a), d 1.1(15) | 17 | 9, 10, 11, 16 | 10, 15, 17a | 7, 10 | 15 |
| C18 | 27.45 | | 18 | 19 | | | |
| H18 | 1.382 | s | 18 | 1, 13, 14, 19 | | 11b, 12a, 13 | |
| C19 | 28.82 | | 19 | 18 | | | |
| H19 | 1.388 | s | 19 | 1, 13, 14, 18 | | 2, 13, 15 | |

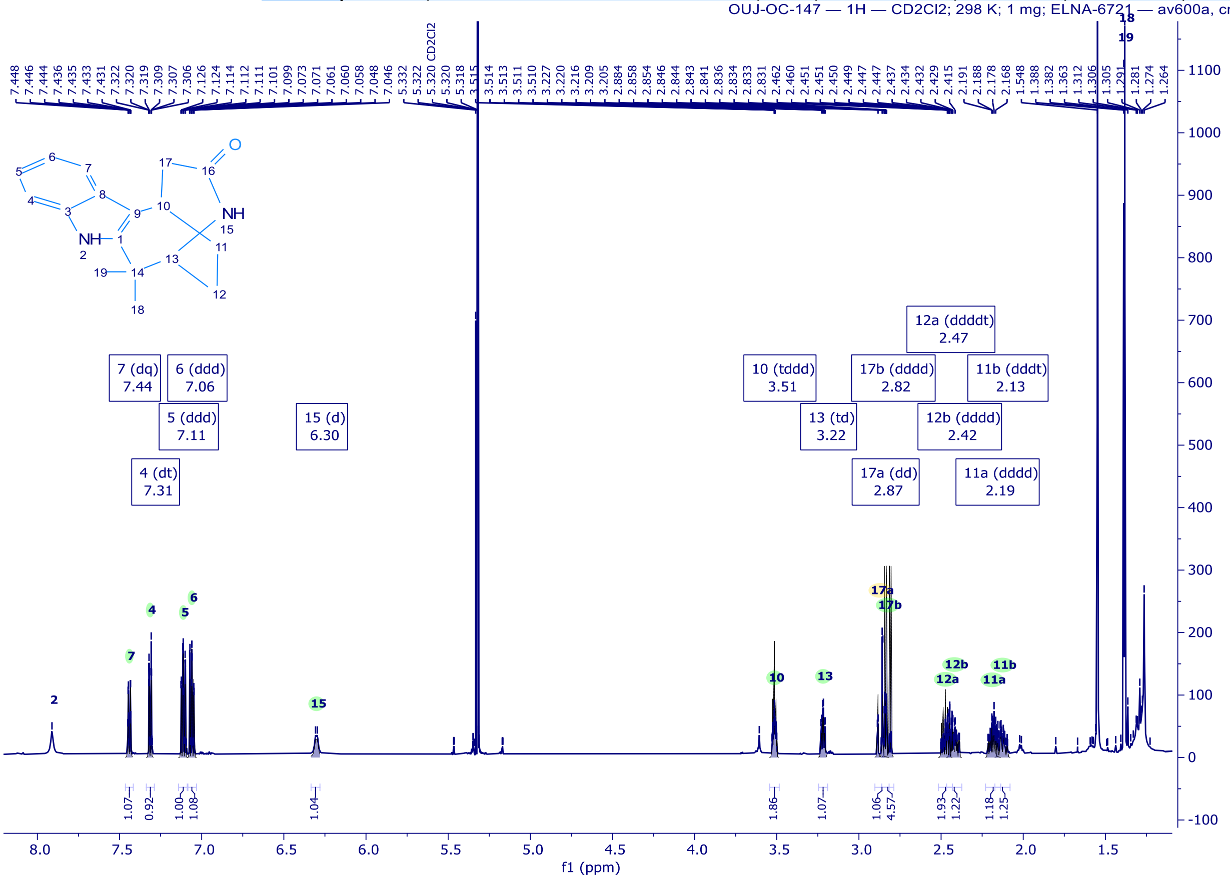

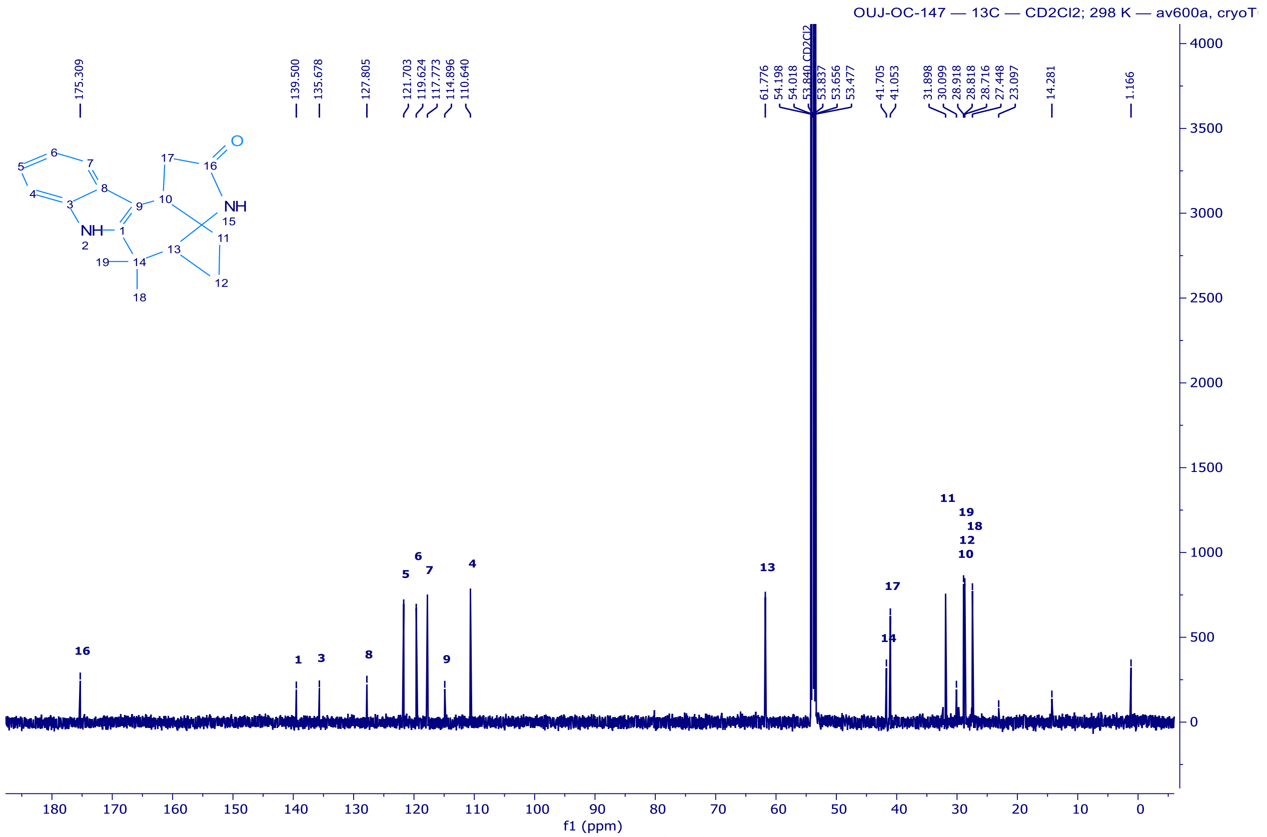
OUJ-OC-147 — 13C — CD2Cl2; 298 K — av600a, cryoT
175.309
139.500
135.678
127.805
121.703
119.624
117.773
114.896
110.640
61.776
54.198
54.018
53.840 CD2Cl2
53.837
53.656
53.477
41.705
41.053
31.898
30.099
28.918
28.818
28.716
27.448
23.097
14.281
1.166
f1 (ppm)

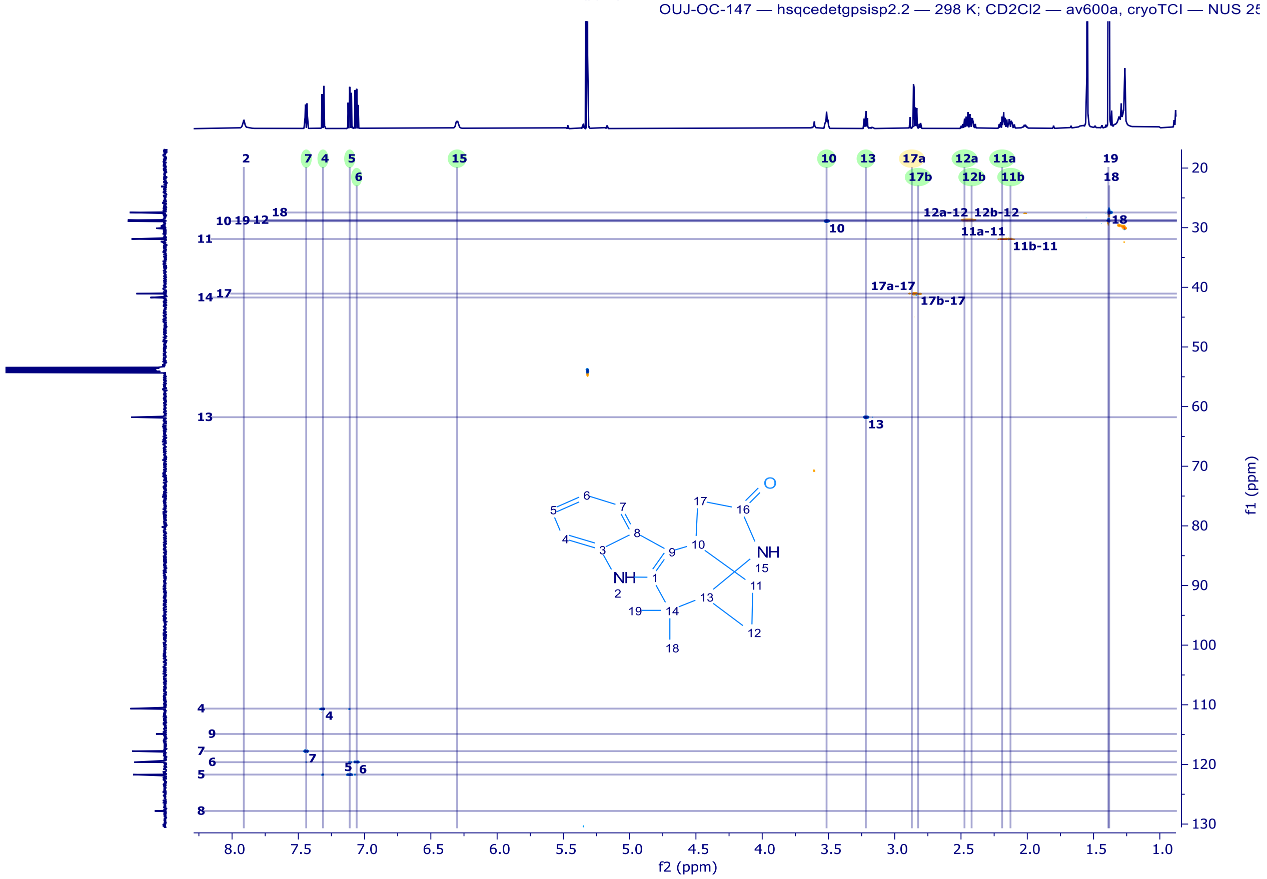
OUJ-OC-147 — hsqcedetgpsisp2.2 — 298 K; CD2Cl2 — av600a, cryoTCI — NUS 25
f2 (ppm)
f1 (ppm)

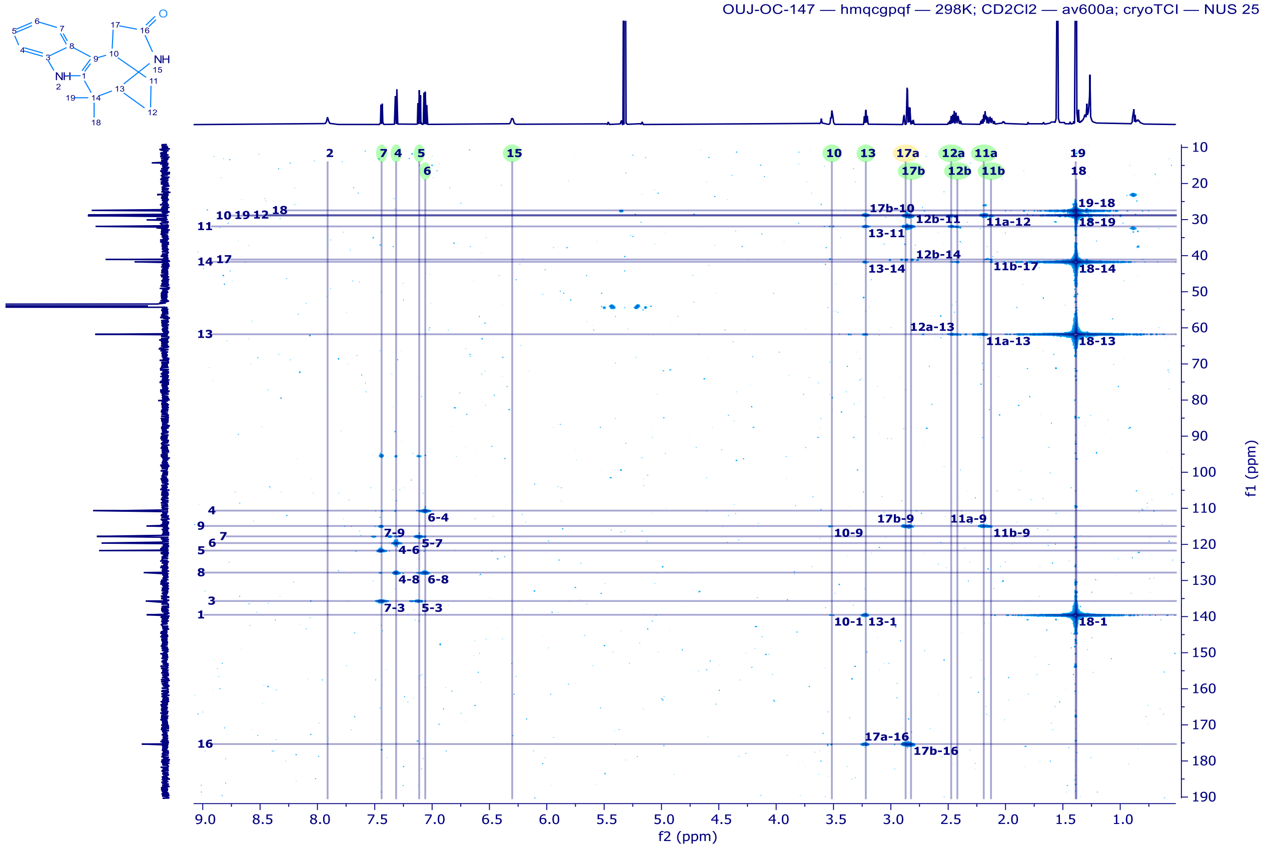

OUJ-OC-147 — hmqcgpqf — 298K; CD2Cl2 — av600a; cryoTCI — NUS 25
f2 (ppm)
f1 (ppm)


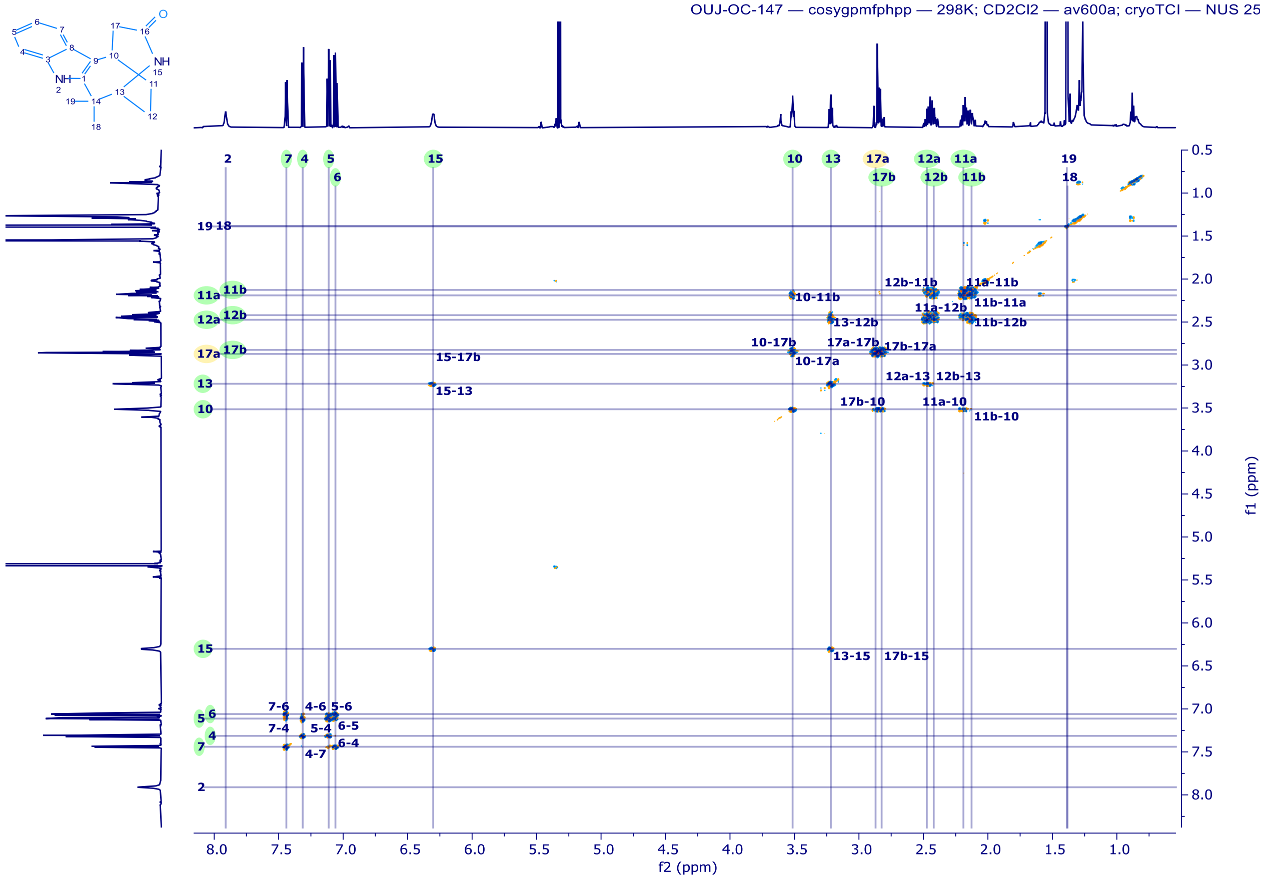

OUJ-OC-147 — cosygpmfphpp — 298K; CD2Cl2 — av600a; cryoTCI — NUS 25
f2 (ppm)
f1 (ppm)

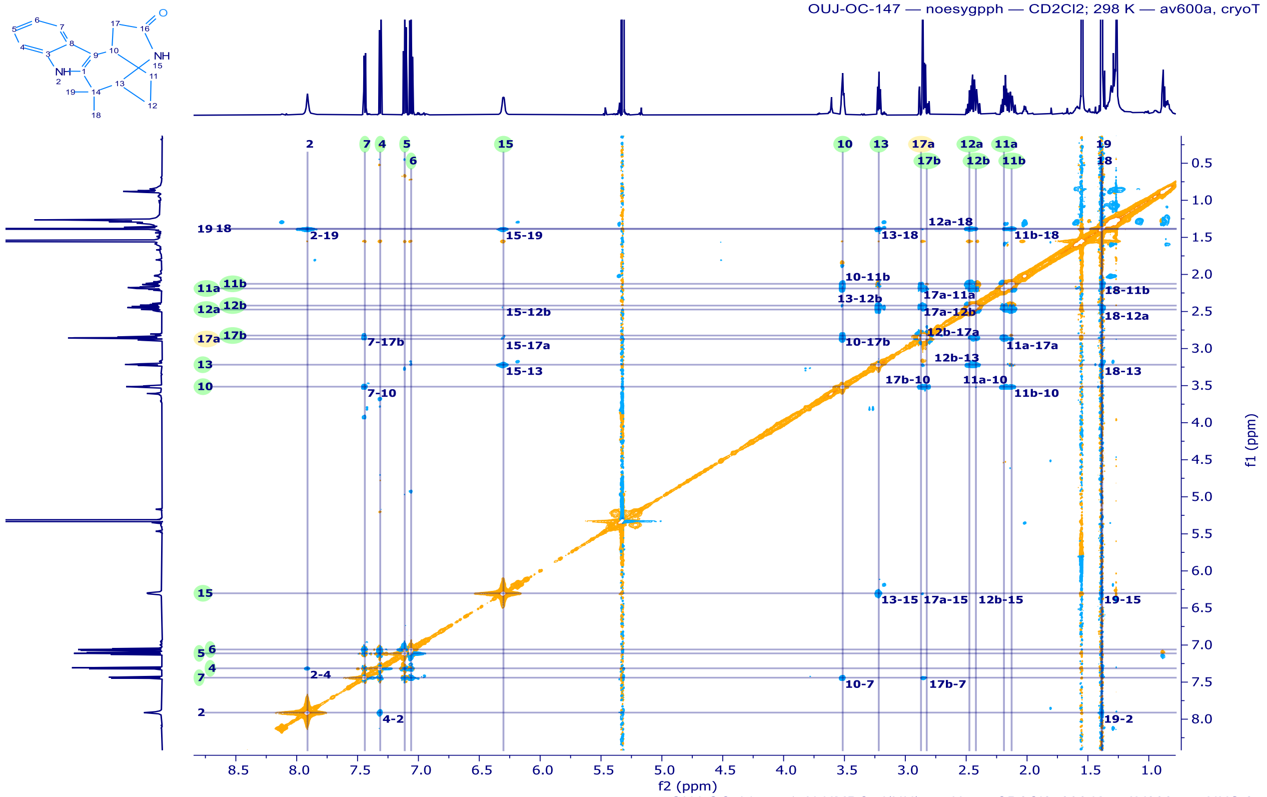
OUJ-OC-147 — noesygpph — CD2Cl2; 298 K — av600a, cryoT
f2 (ppm)
f1 (ppm)
2-19
15-19
13-18
12a-18
11b-18
10-11b
13-12b
17a-11a
17a-12b
18-11b
18-12a
15-12b
7-17b
15-17a
12b-17a
11a-17a
12b-13
15-13
18-13
17b-10
11a-10
7-10
11b-10
13-15
17a-15
12b-15
19-15
2-4
10-7
17b-7
4-2
19-2

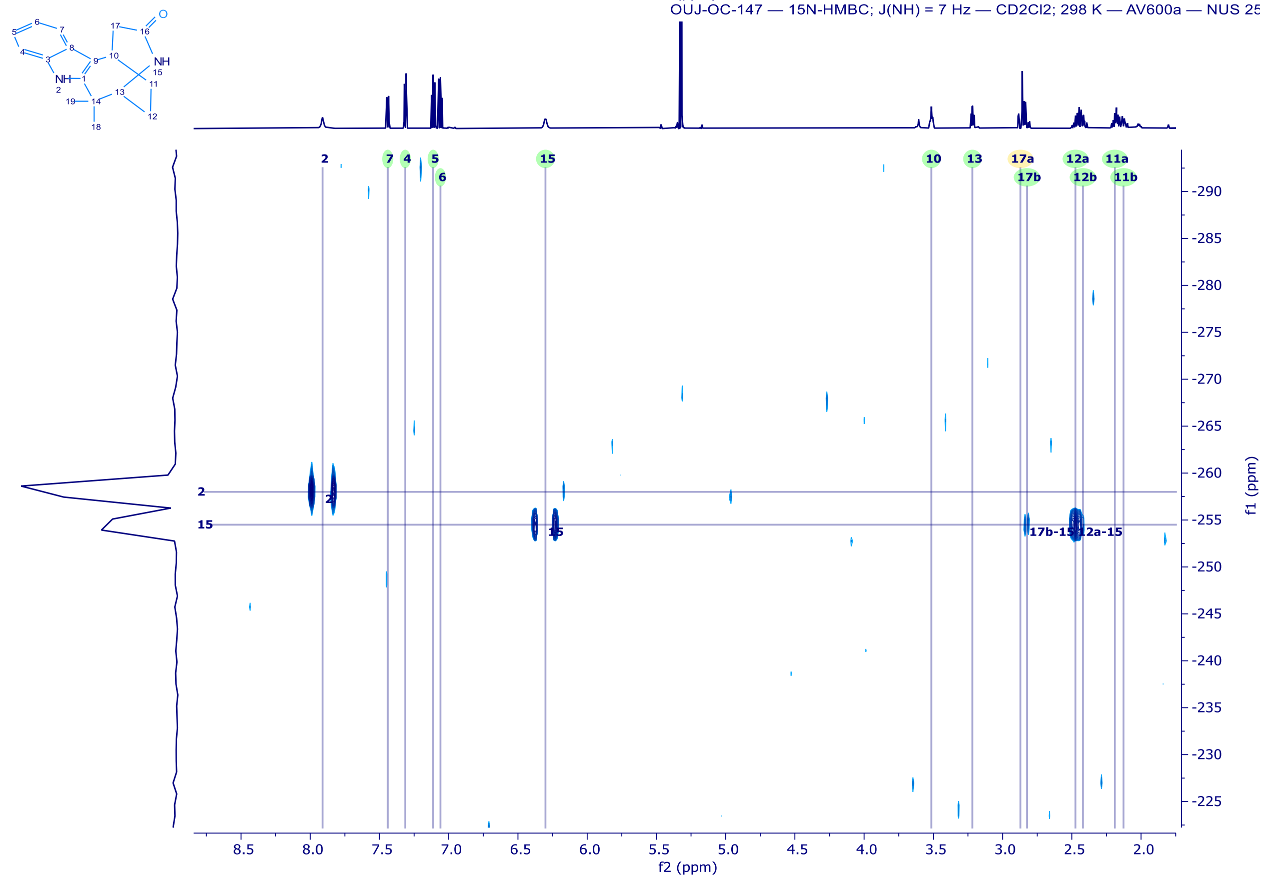
OUJ-OC-147 — 15N-HMBC; J(NH) = 7 Hz — CD2Cl2; 298 K — AV600a — NUS 25
f2 (ppm)
f1 (ppm)
17b-15
12a-15

NMR spectra for compound **10**: $^{1}H$ (501 MHz) and $^{13}C$ (126 MHz) in $CD_2Cl_2$.

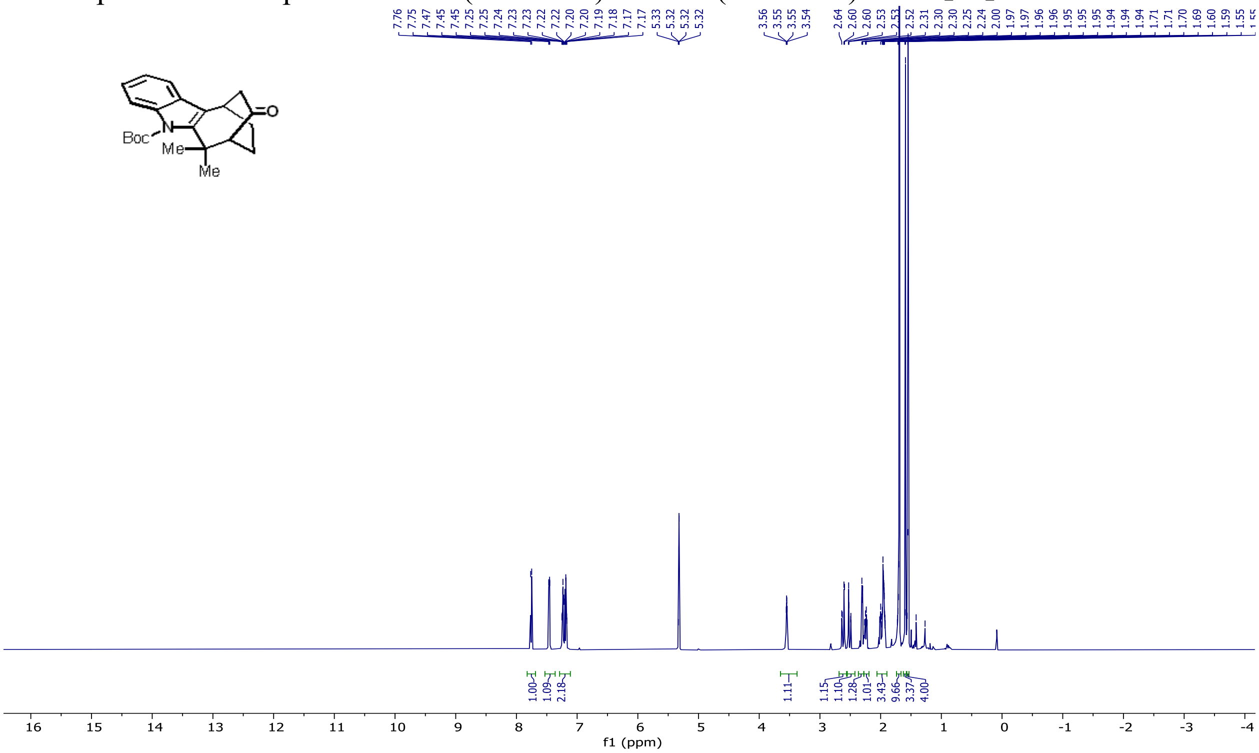


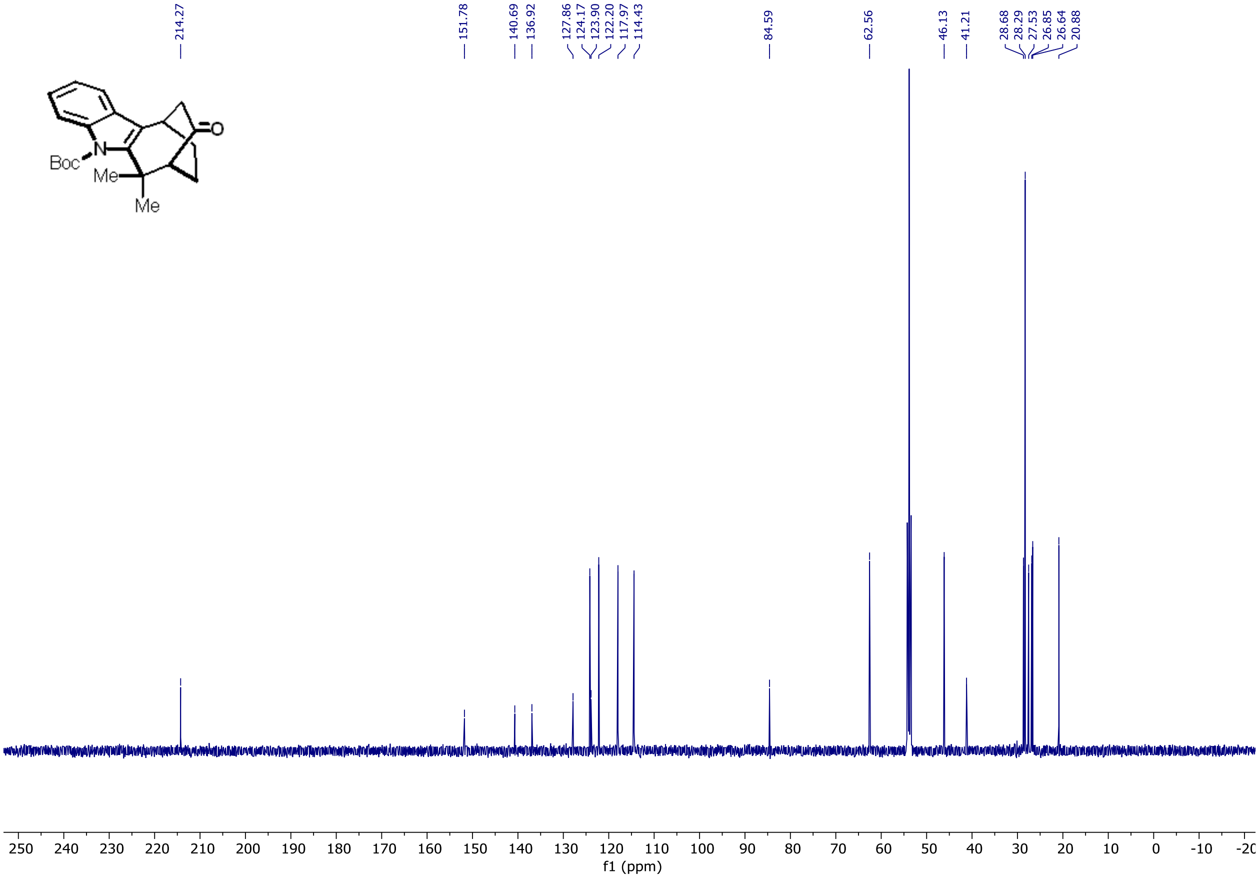

NMR spectra for compound **11**: $^{1}H$ (501 MHz) and $^{13}C$ (126 MHz) in $CD_2Cl_2$.

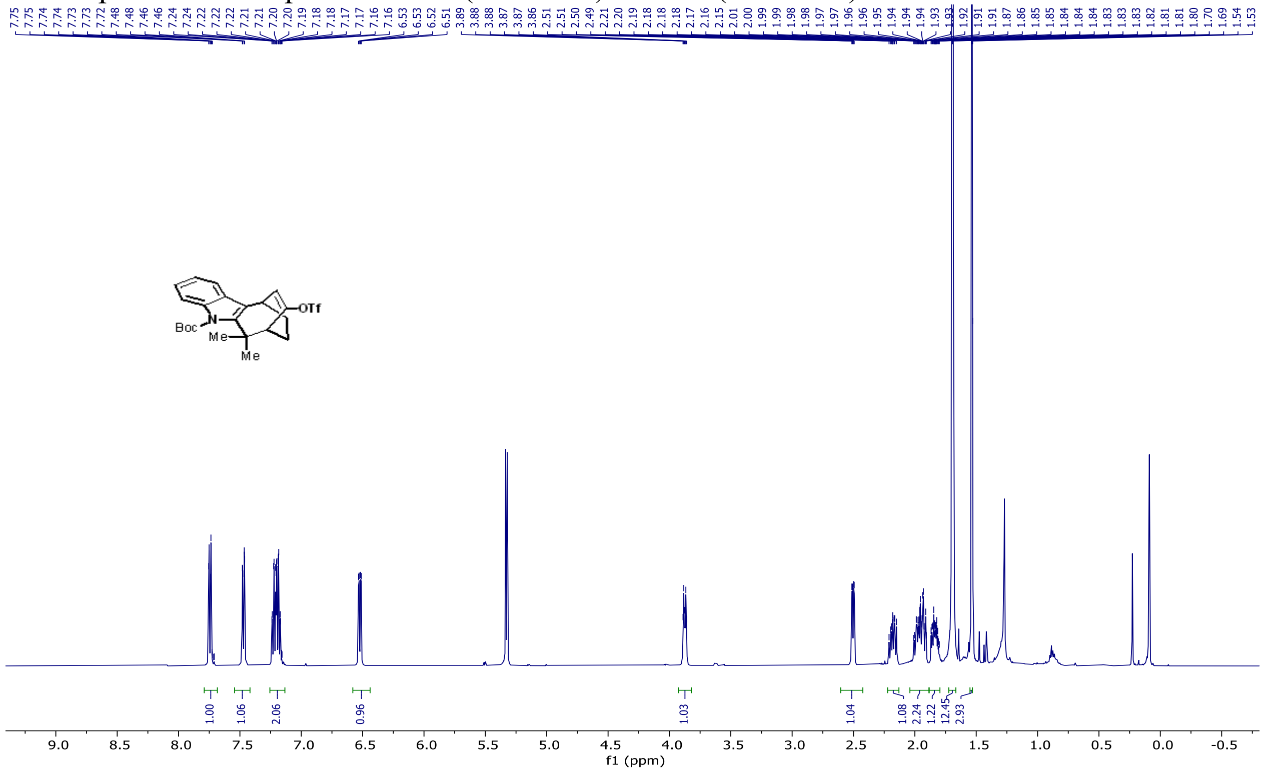

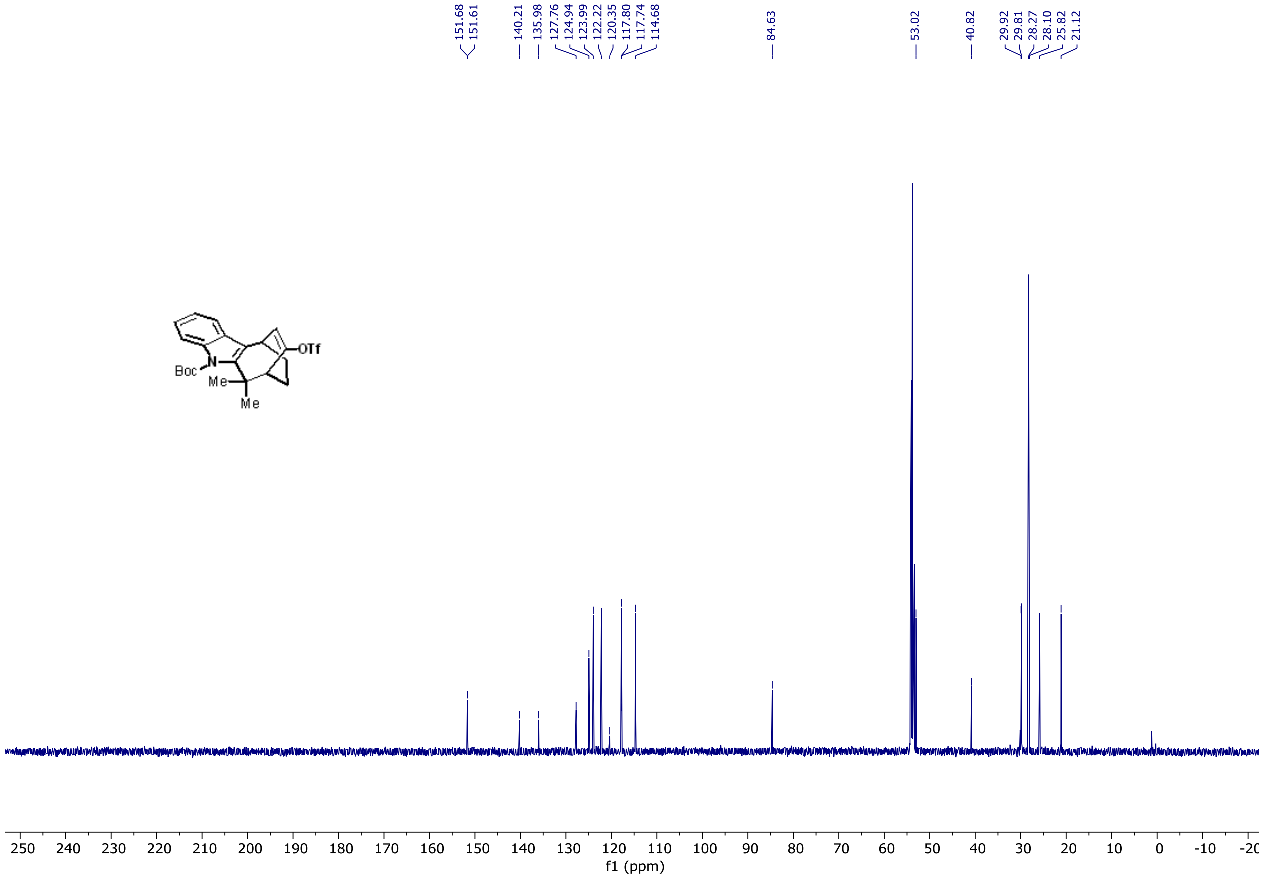

# Characterization of 12

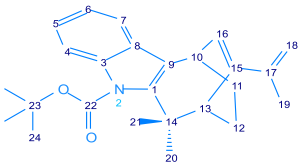

| Atom | δ (ppm) | J | HSQC | HMQC | COSY | NOESY |
|---|---|---|---|---|---|---|
| C1 | 141.18 | | | 10, 13, 20, 21 | | |
| C3 | 136.24 | | | 5, 7 | | |
| C4 | 114.47 | | 4 | 6 | | |
| H4 | 7.700 | m | 4 | 6, 8 | 5, 6, 7 | 24 |
| C5 | 123.40 | | 5 | 7 | | |
| H5 | 7.179 | m | 5 | 3, 7 | 4, 6, 7 | 24 |
| C6 | 121.88 | | 6 | 4 | | |
| H6 | 7.156 | m | 6 | 4, 8 | 4, 5, 7 | |
| C7 | 117.79 | | 7 | 5 | | |
| H7 | 7.487 | m | 7 | 3, 5, 9 | 4, 5, 6 | 10, 11a |
| C8 | 128.13 | | | 4, 6, 10 | | |
| C9 | 120.13 | | | 7, 10, 11b, 16 | | |
| C10 | 30.35 | | 10 | 11b, 16 | | |
| H10 | 3.758 | d 4.1(11b), d 2.3(11a), d 7.7(16) | 10 | 1, 8, 9, 11, 15, 16 | 11a, 11b, 16 | 7, 11a, 11b, 16 |
| C11 | 29.83 | | 11a, 11b | 10, 12a, 12b, 13 | | |
| H11a | 1.901 | d 12.3(11b), d 9.5(12a), t 2.7(10, 12b) | 11 | | 10, 11b, 12a, 12b | 7, 10, 11b, 12a |
| H11b | 1.710 | d 4.1(10), d 12.3(11a), d 5.5(12a), d 11.2(12b) | 11 | 9, 10 | 10, 11a, 12a, 12b | 10, 11a, 16 |
| C12 | 21.02 | | 12a, 12b | | | |
| H12a | 2.227 | d 1.1(13), d 9.5(11a), d 5.5(11b), d 14.1(12b) | 12 | 11, 13, 14, 15 | 11a, 11b, 12b, 13 | 11a, 12b, 13, 20 |
| H12b | 1.632 | d 6.8(13), d 14.1(12a), d 2.7(11a), d 11.2(11b) | 12 | 11 | 11a, 11b, 12a, 13 | 12a, 13, 16 |
| C13 | 47.72 | | 13 | 12a, 16, 20, 21 | | |
| H13 | 2.890 | d 6.8(12b), t 1.5(12a, 16) | 13 | 1, 11, 14, 15, 16, 17, 20, 21 | 12a, 12b, 16, 18a | 12a, 12b, 18a, 20, 21 |
| C14 | 41.36 | | | 12a, 13, 20, 21 | | |
| C15 | 143.17 | | | 10, 12a, 13, 18a, 18b, 19 | | |
| C16 | 131.68 | | 16 | 10, 13, 18a | | |
| H16 | 6.614 | d 1.9(13), d 7.7(10) | 16 | 9, 10, 13, 17 | 10, 13, 18a, 18b | 10, 11b, 12b, 19, 21 |
| C17 | 143.95 | | | 13, 16, 19 | | |
| C18 | 110.84 | | 18a, 18b | | | |
| H18a | 5.172 | d 1.4(18b?), q 0.6(19) | 18 | 15, 16, 19 | 13, 16, 18b, 19 | 13, 18b, 21 |
| H18b | 4.959 | q 1.4(19), d 2.2(18a?) | 18 | 15, 19 | 16, 18a, 19 | 18a, 19, 21 |
| C19 | 20.80 | | 19 | 18a, 18b | | |
| H19 | 1.938 | d 1.4(18b), d 0.6(18a) | 19 | 15, 17 | 18a, 18b | 16, 18b, 21 |
| C20 | 27.19 | | 20 | 13, 21 | | |
| H20 | 1.584 | s | 20 | 1, 13, 14, 21 | 21 | 12a, 13, 21 |
| C21 | 27.80 | | 21 | 13, 20 | | |
| H21 | 1.443 | s | 21 | 1, 13, 14, 20 | 20 | 13, 16, 18a, 18b, 19, 20 |
| C22 | 151.88 | | | | | |
| C23 | 84.12 | | | 24 | | |
| C24 | 28.26 | | 24 | | | |
| H24 | 1.671 | s | 24 | 23 | | 4, 5 |

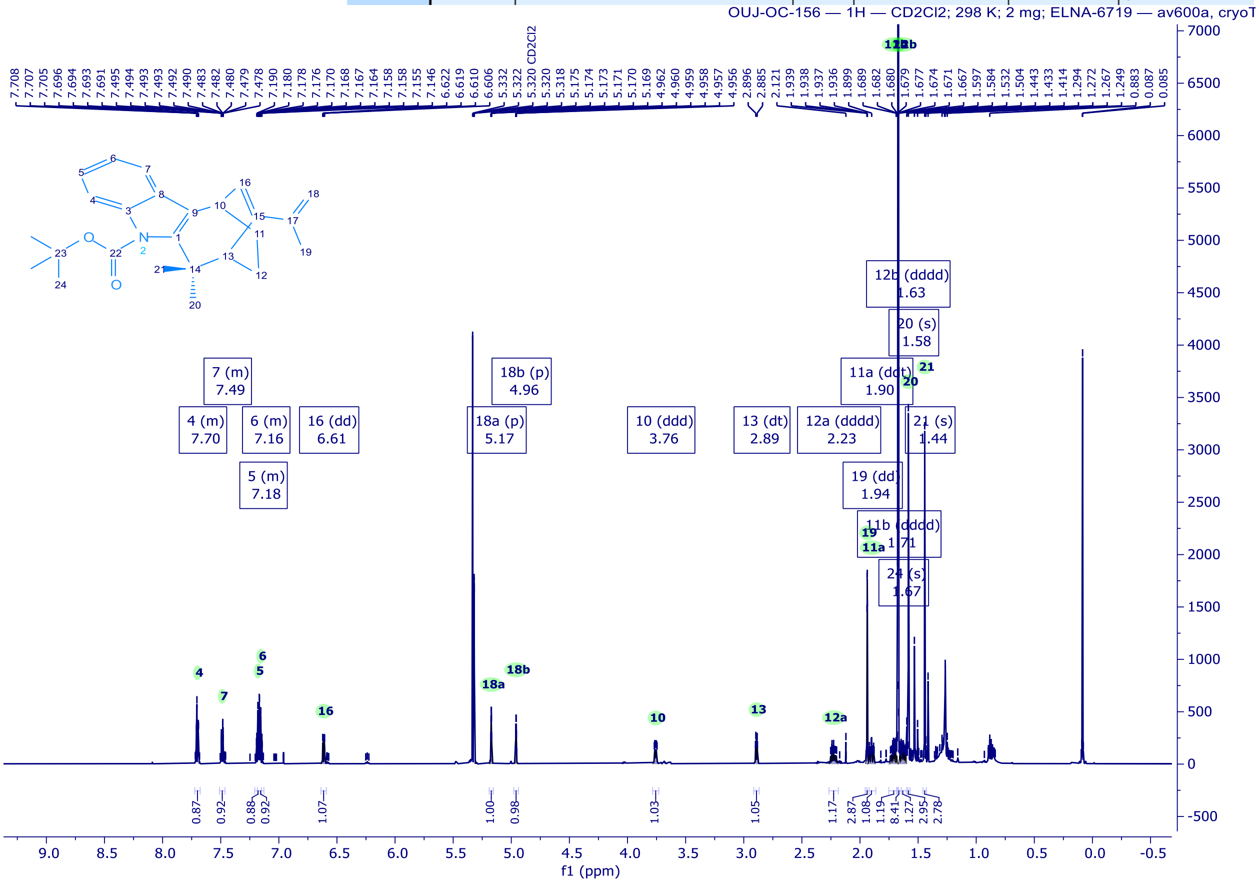

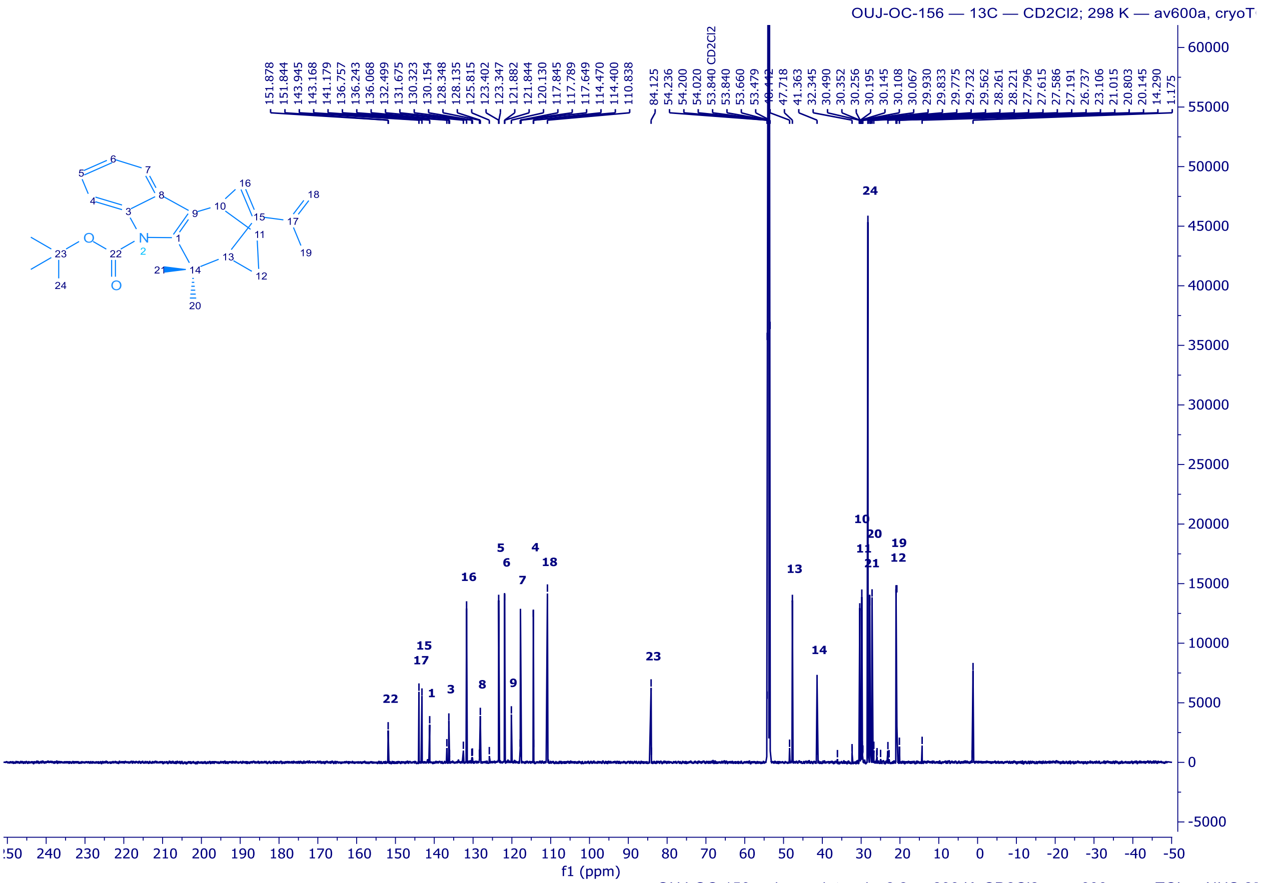

OUJ-OC-156 — 13C — CD2Cl2; 298 K — av600a, cryoT
151.878 151.844 143.945 143.168 141.179 136.757 136.243 136.068 132.499 131.675 130.323 130.154 128.348 128.135 125.815 123.402 123.347 121.882 121.844 120.130 117.845 117.789 117.649 114.470 114.400 110.838
84.125 54.236 54.200 54.020 53.840 CD2Cl2 53.840 53.660 53.479 48.112 47.718 41.363 32.345 30.490 30.352 30.256 30.195 30.145 30.108 30.067 29.930 29.833 29.775 29.732 29.562 28.261 28.221 27.796 27.615 27.586 27.191 26.737 23.106 21.015 20.803 20.145 14.290 1.175
f1 (ppm)


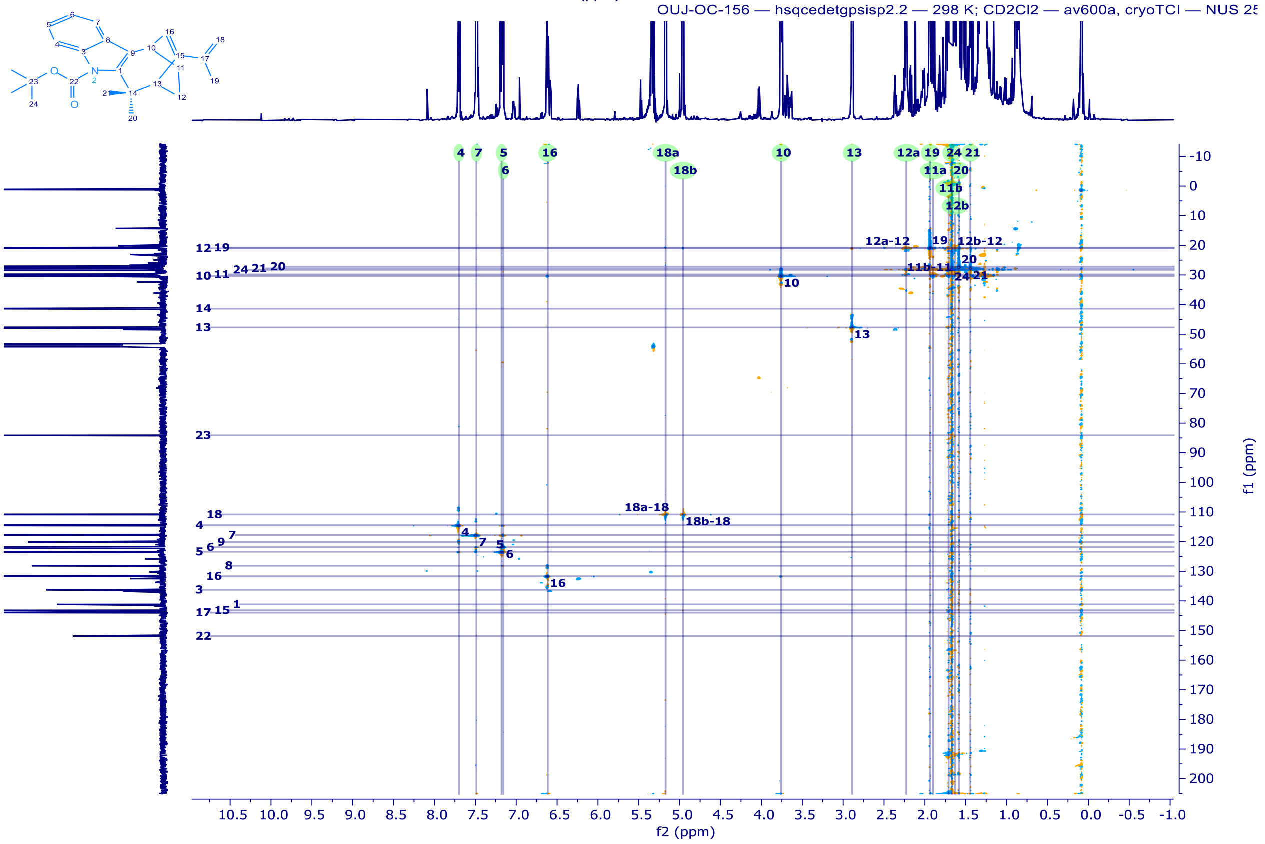

OUJ-OC-156 — hsqcedetgpsisp2.2 — 298 K; CD2Cl2 — av600a, cryoTCI — NUS 25
f2 (ppm)
f1 (ppm)

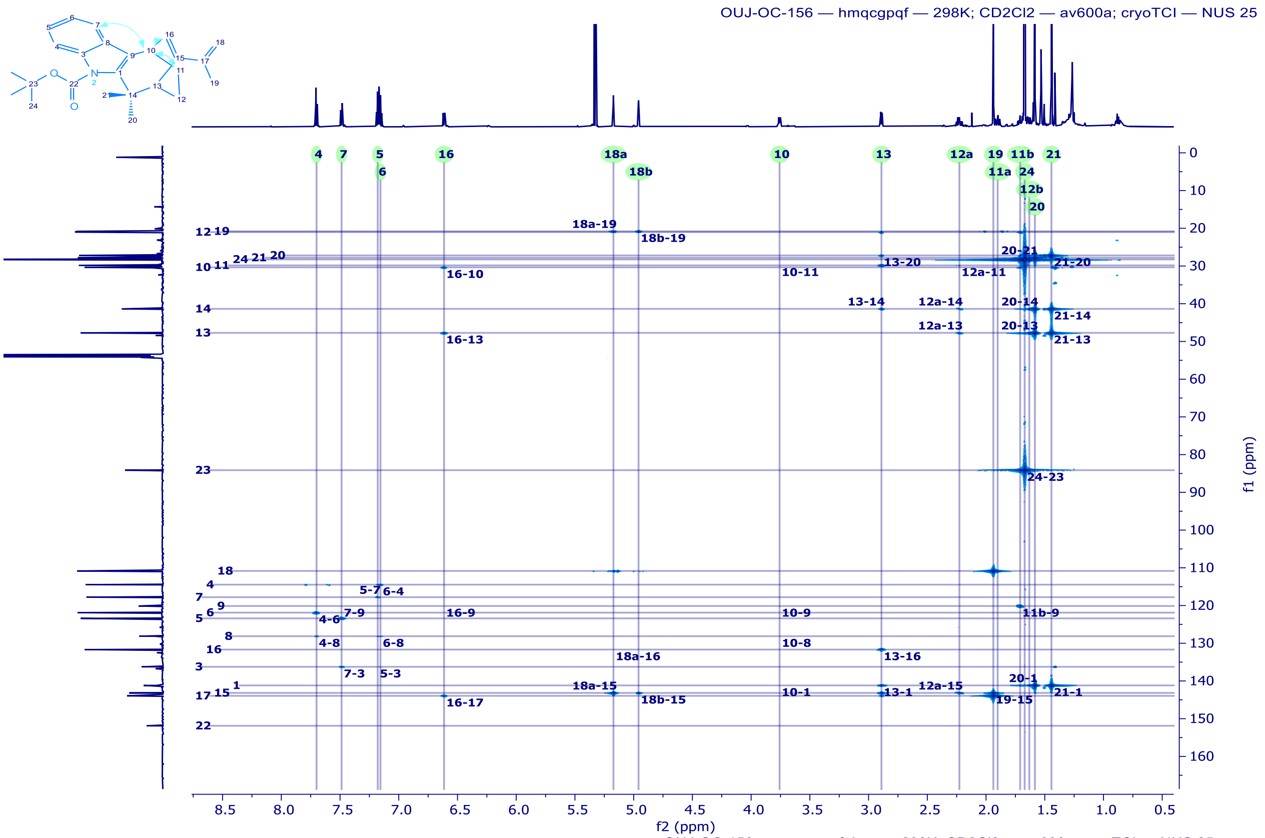
OUJ-OC-156 — hmqcgpqf — 298K; CD2Cl2 — av600a; cryoTCI — NUS 25
f2 (ppm)
f1 (ppm)

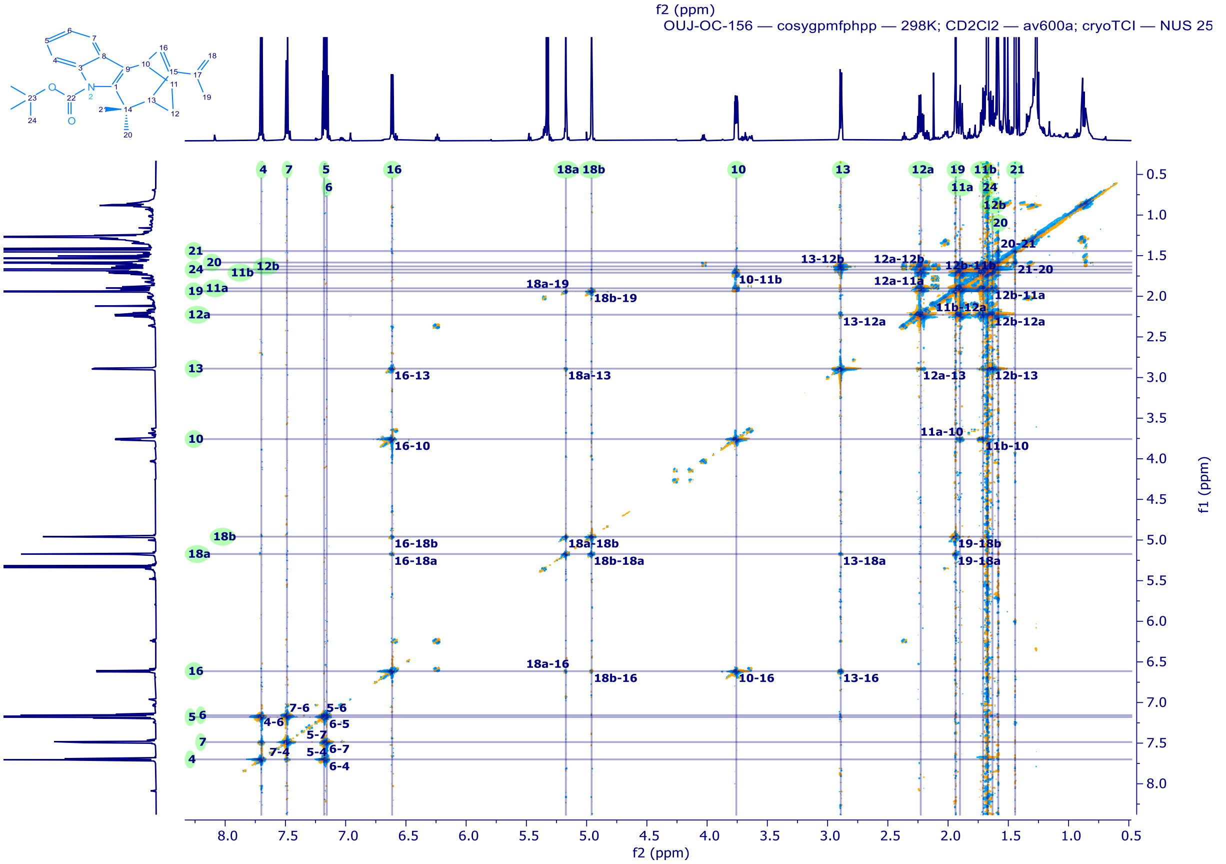
OUJ-OC-156 — cosygpmfphpp — 298K; CD2Cl2 — av600a; cryoTCI — NUS 25
f2 (ppm)
f1 (ppm)

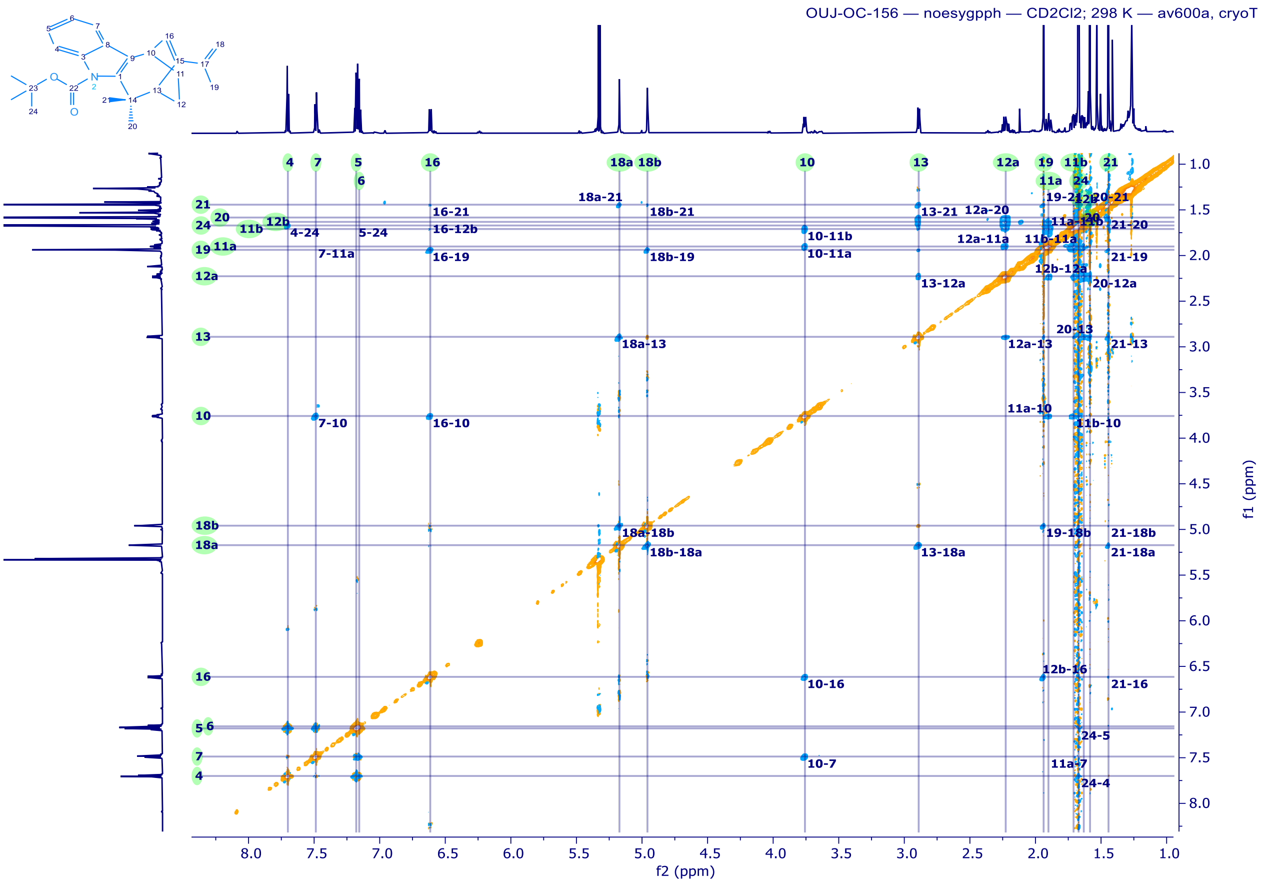


## Characterization of 13

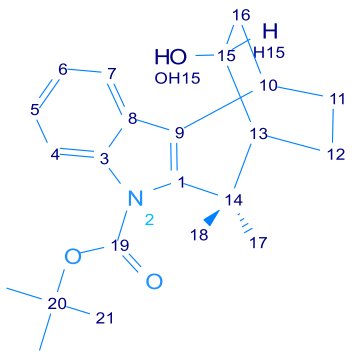

| Atom | δ (ppm) | J | HSQC | HMQC | COSY | NOESY |
|---|---|---|---|---|---|---|
| C1 | 142.12 | | | 17, 18 | | |
| C3 | 137.47 | | | 5, 7 | | |
| C4 | 114.50 | | 4 | 6 | | |
| H4 | 7.964 | d 8.3(5), d 1.0(6), d 0.7(7) | 4 | 6, 8 | 5 | 21 |
| C5 | 123.64 | | 5 | 7 | | |
| H5 | 7.270 | d 8.3(4), d 7.2(6), d 1.4(7) | 5 | 3, 7 | 4, 6 | |
| C6 | 122.08 | | 6 | 4 | | |
| H6 | 7.202 | d 7.6(7), d 7.2(5), d 1.0(4) | 6 | 4, 7, 8 | 5, 7 | |
| C7 | 117.96 | | 7 | 5, 6 | | |
| H7 | 7.352 | dm 7.6(6) | 7 | 3, 5, 9 | 6 | 10 |
| C8 | 128.43 | | | 4, 6 | | |
| C9 | 125.36 | | | 7, 16', 16" | | |
| C10 | 27.58 | | 10 | 16" | | |
| H10 | 3.068 | d 6.8(16'), d 3.3(11), d 3.0(11), d 1.3(16") | 10 | | 11, 16', 16" | 7, 11, 16', 16" |
| C11 | 24.66 | | 11 | 13, 16" | | |
| H11', 11" | 1.444 | m (o.l.) | 11 | | 10, 12', 12" | 15 10, 12', 16' |
| C12 | 22.91 | | 12', 12" | 13 | | |
| H12' (down) | 1.618 | m | 12 | 15 | 11, 12" | 11, 12" |
| H12" (up) | 1.380 | m | 12 | 14 | 11, 12', 13 | 15, 12', 13 |
| C13 | 51.87 | | 13 | 17, 18 | | |
| H13 | 1.679 | m | 13 | 11, 12, 15, 16 | 15, 12" | OH15, 15, 12", 17, 18 |
| C14 | 42.50 | | | 15, 12", 17, 18 | | |
| C15 | 71.78 | | 15 | 12', 13, 16', 16" | | |
| H15 (anti) | 3.952 | d 8.9(16'), d 8.2(16"), d 4.1(13) | 15 | 14 | OH15, 13, 16', 16" | OH15, 11, 12", 13, 16' |
| OH15 (syn) | 0.890 | br s | | | 15 | 15, 13, 16", 18 |
| C16 | 38.41 | | 16', 16" | 13 | | |
| H16' (anti) | 2.177 | d 13.2(16"), d 8.9(15), d 6.8(10), d 0.8(?) | 16 | 9, 15 | 15, 10, 16" | 15 10, 11 16" |
| H16" (syn) | 1.792 | d 13.2(16'), d 8.2(15), t 1.3(10, 11) | 16 | 9, 10, 11, 15 | 15, 10, 16' | OH15, 10, 16', 18 |
| C17 | 29.19 | | 17 | 18 | | |
| H17 | 1.589 | s | 17 | 1, 13, 14, 18 | 18 | 13, 18 |
| C18 | 29.98 | | 18 | 17 | | |
| H18 | 2.113 | s | 18 | 1, 13, 14, 17 | 17 | OH15, 13, 16", 17 |
| C19 | 152.02 | | | | | |
| C20 | 83.15 | | | 21 | | |
| C21 | 27.96 | | 21 | | | |
| H21 | 1.410 | s | 21 | 20 | | 4 |

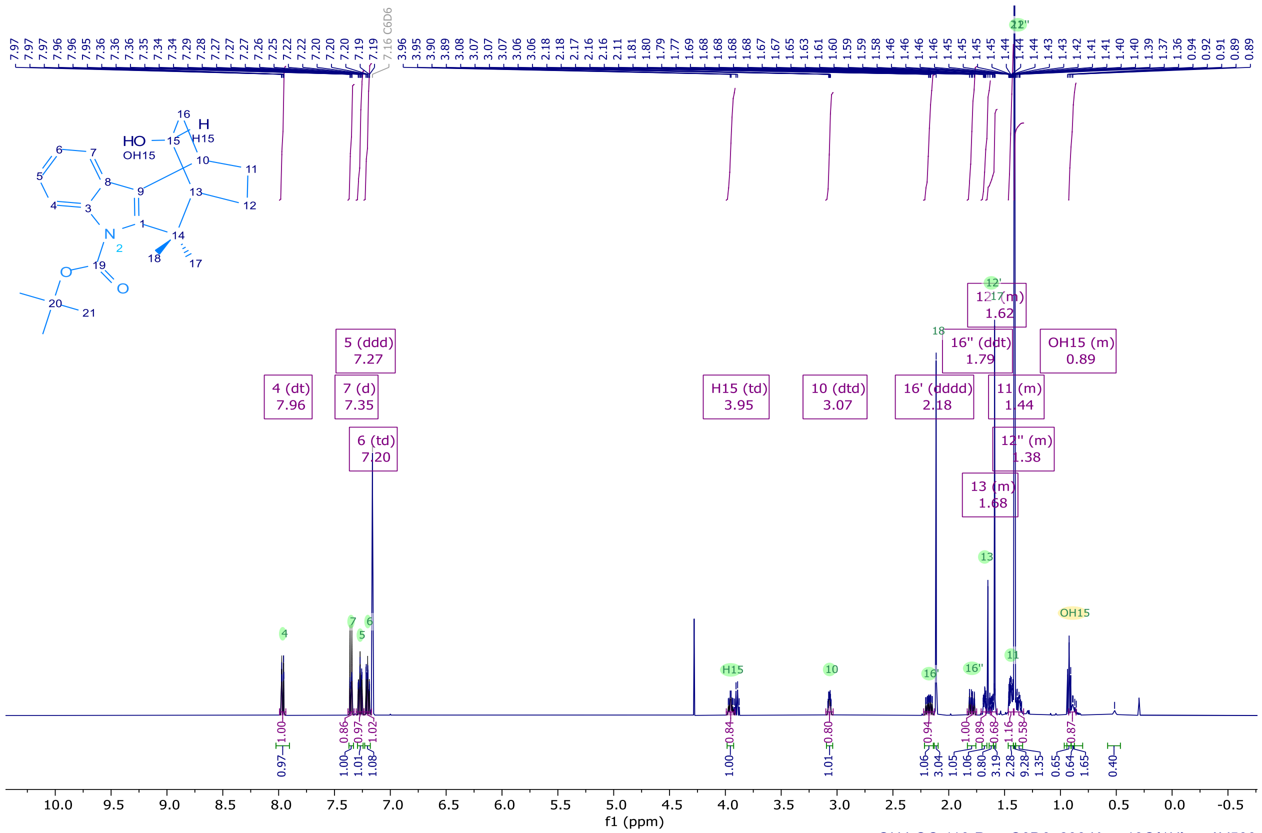

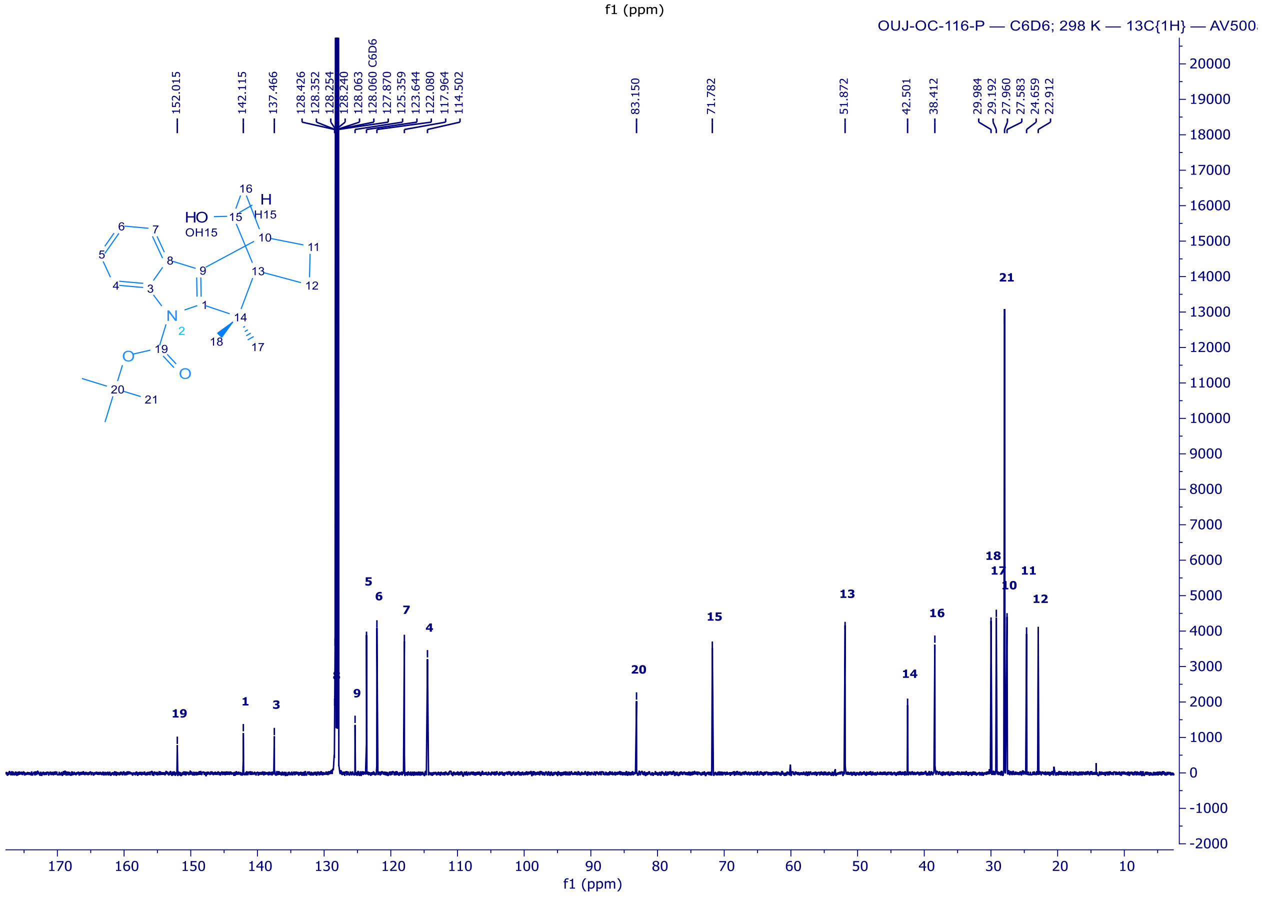

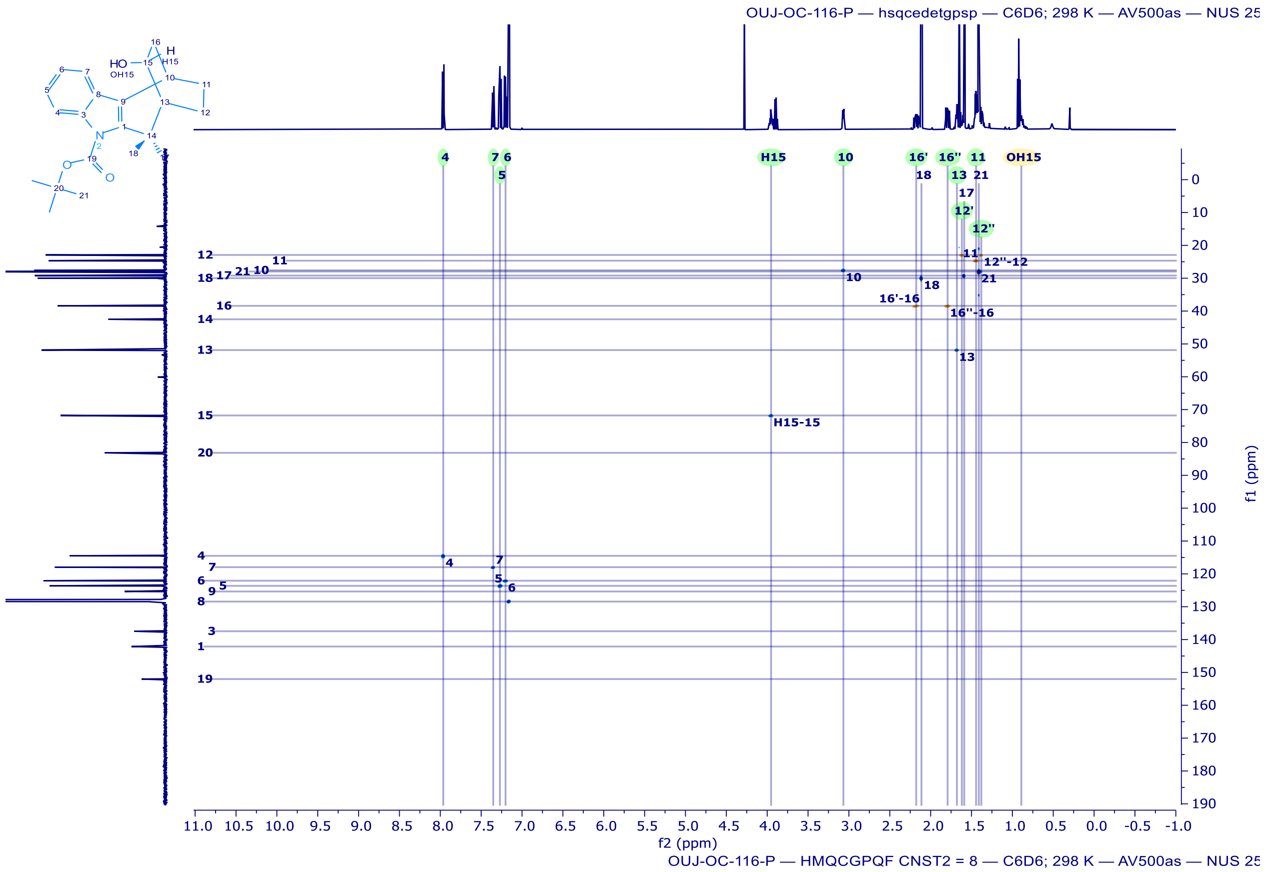
OUJ-OC-116-P — hsqcedetgpsp — C6D6; 298 K — AV500as — NUS 25
f2 (ppm)
f1 (ppm)

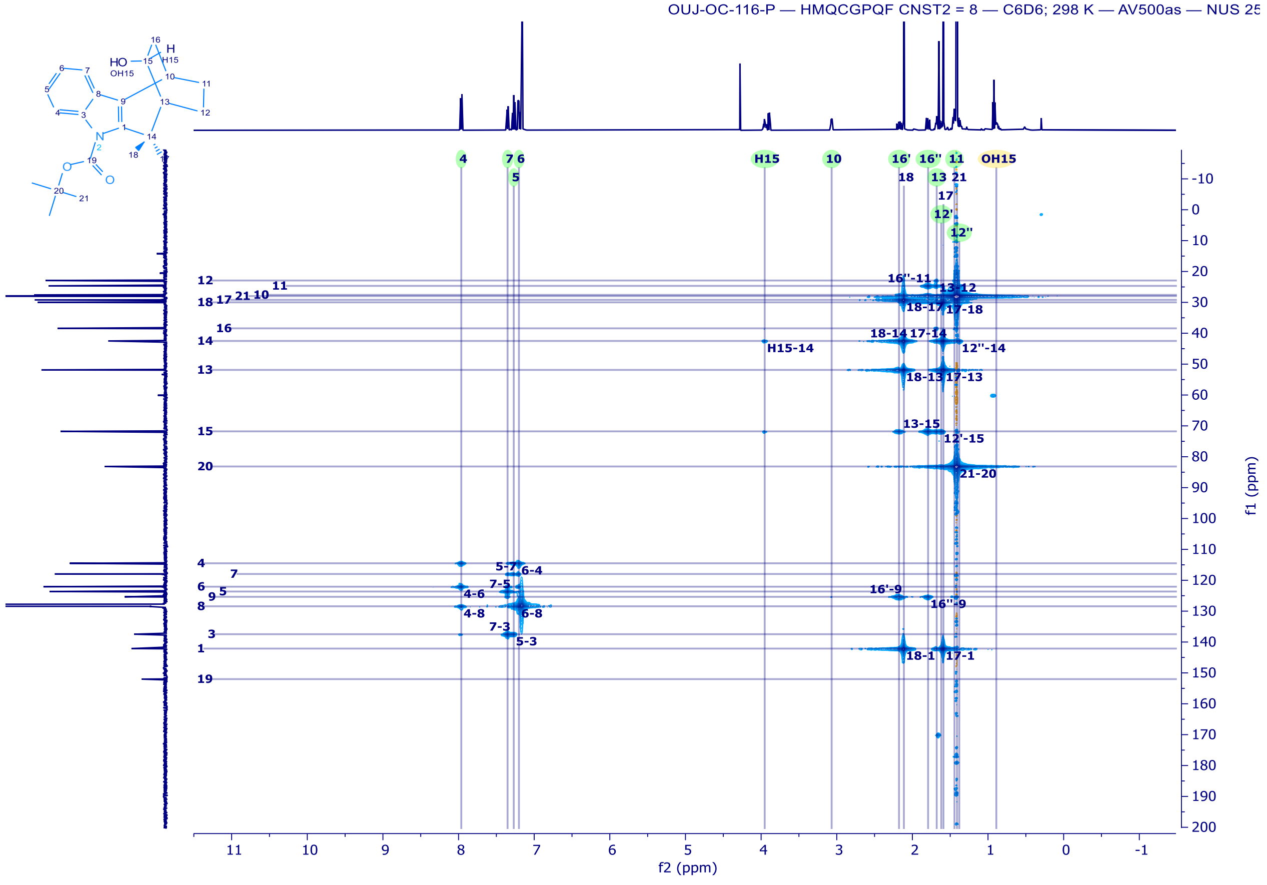
OUJ-OC-116-P — HMQCGPQF CNST2 = 8 — C6D6; 298 K — AV500as — NUS 25
f2 (ppm)
f1 (ppm)

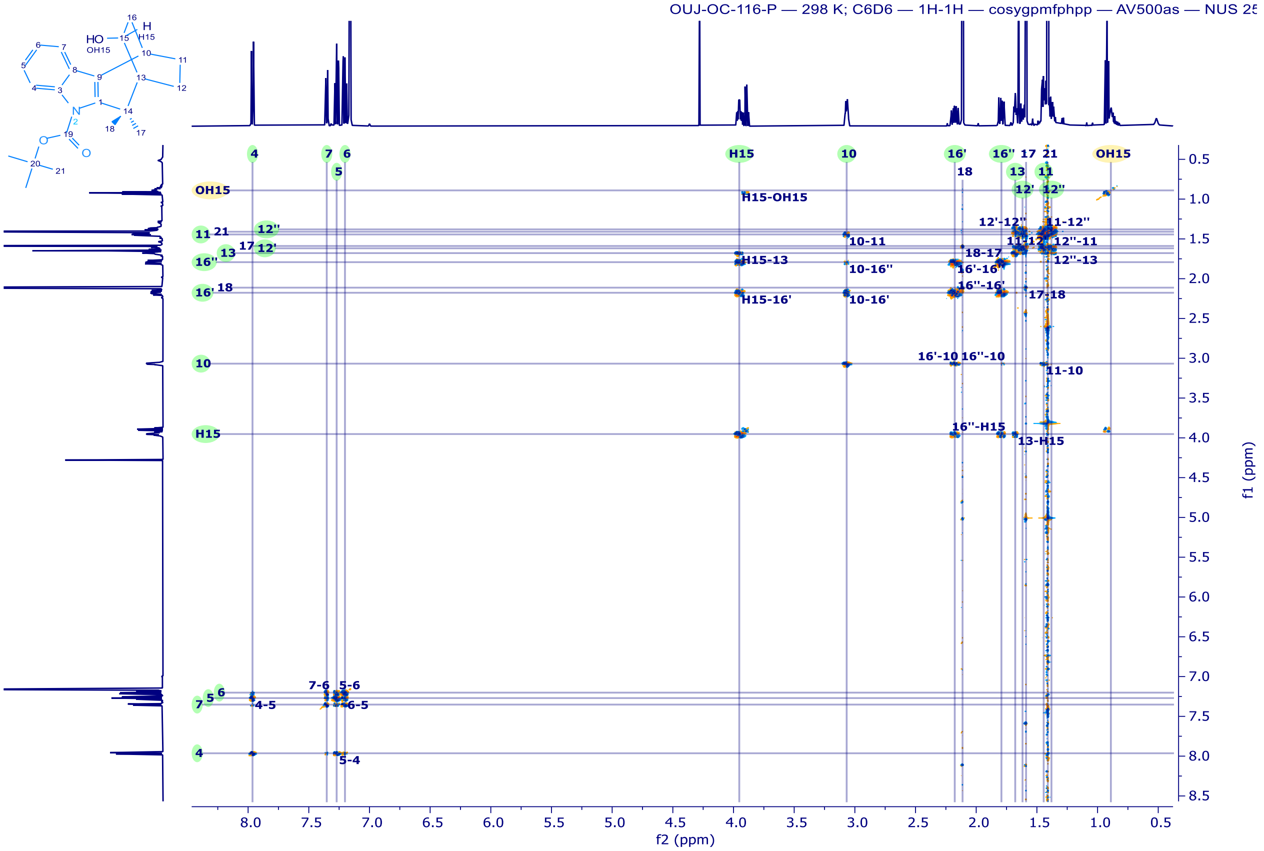
OUJ-OC-116-P — 298 K; C6D6 — 1H-1H — cosygpmfphpp — AV500as — NUS 25
f2 (ppm)
f1 (ppm)

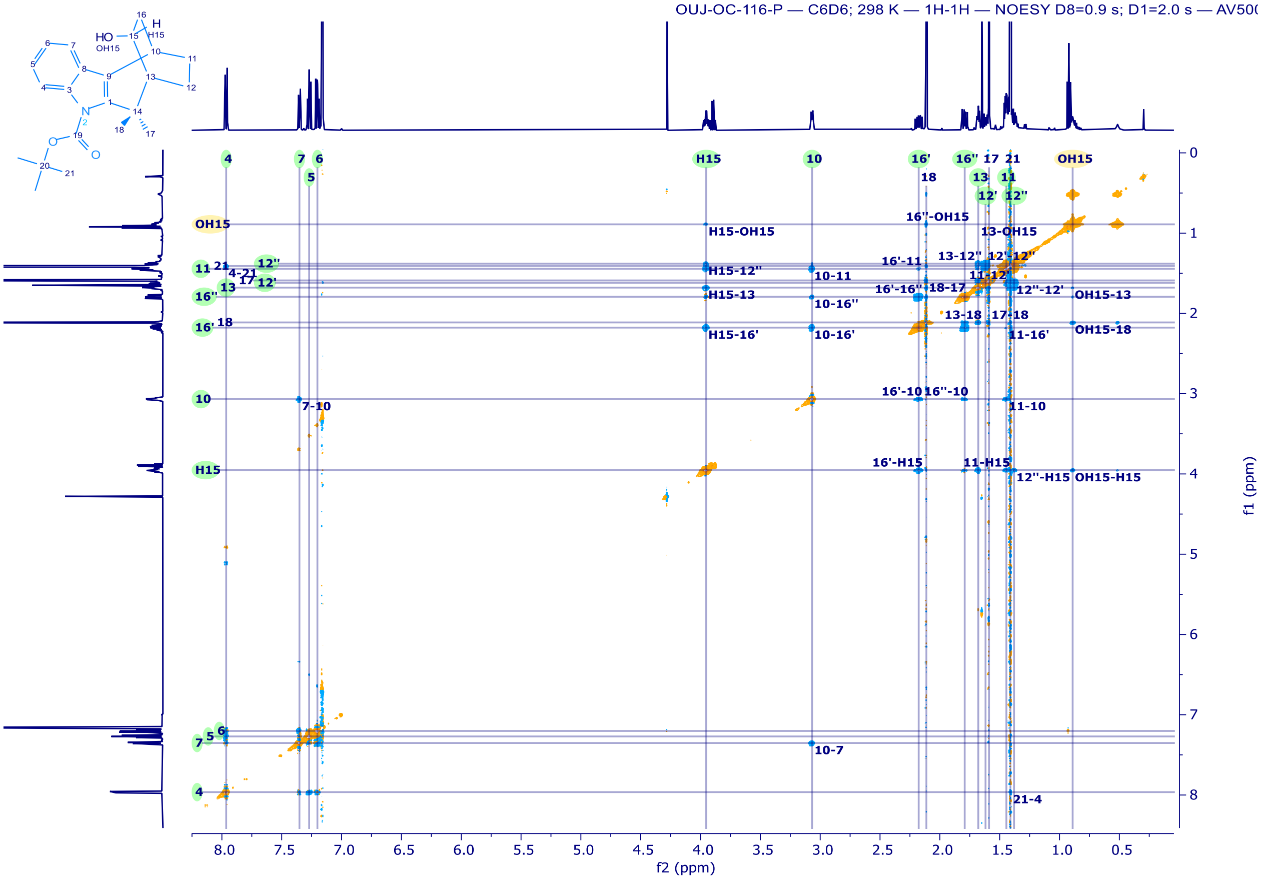
OUJ-OC-116-P — C6D6; 298 K — 1H-1H — NOESY D8=0.9 s; D1=2.0 s — AV50
f2 (ppm)
f1 (ppm)

## Characterization of 14

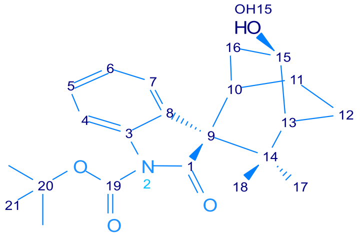


| Atom | δ (ppm) | J | HSQC | HMQC | COSY | NOESY |
|---|---|---|---|---|---|---|
| C1 | 178.18 | | | | | |
| C3 | 139.82 | | | 5, 7 | | |
| C4 | 114.42 | | 4 | 4, 6 | | |
| H4 | 7.761 | d 1.3(6), d 8.1(5) | 4 | 4, 6, 8 | 5, 6 | 5, 21 |
| C5 | 128.04 | | 5 | 7 | | |
| H5 | 7.285 | d 7.5(6), d 1.3(7), d 8.1(4) | 5 | 3, 7 | 4, 6, 7 | 4 |
| C6 | 123.33 | | 6 | 4 | | |
| H6 | 7.110 | d 7.9(7), d 7.5(5), d 1.3(4) | 6 | 4, 8 | 4, 5, 7 | 7 |
| C7 | 127.89 | | 7 | 5 | | |
| H7 | 7.689 | d 7.9(6), d 1.3(5), d 0.5(4) | 7 | 3, 5, 9 | 5, 6 | 6, 11', 12', 17 |
| C8 | 130.97 | | | 4, 6 | | |
| C9 | 57.06 | | | 7, 11", 16", 17, 18 | | |
| C10 | 36.13 | | 10 | 16' | | |
| H10 | 1.713 | quint 3.0(11', 11", 16', 16") | 10 | 15 | 11', 11", 16', 16" | 11', 11", 16', 16" |
| C11 | 21.51 | | 11', 11" | 16' | | |
| H11' (down) | 2.258 | m | 11 | | 10, 11", 12', 16' | 7, 10, 11", 17 |
| H11" (up) | 1.388 | m (o.l.) | 11 | 9 | 10, 11' | 10, 11', 16" |
| C12 | 21.94 | | 12', 12" | | | |
| H12' (down) | 2.094 | m | 12 | 13, 15 | 11', 12", 13 | 7, 12", 13, 17 |
| H12" (up) | 1.389 | m (o.l.) | 12 | 15 | 12', 13 | 12', 13, 15 |
| C13 | 44.38 | | 13 | 12', 17, 18 | | |
| H13 | 1.531 | m (o.l.) | 13 | 15 | 12', 12", 15 | 12', 12", 17, 18 |
| C14 | 36.15 | | | 17, 18 | | |
| C15 | 71.78 | | 15 | 10, 12', 12", 13, 16', 16" | | |
| H15 (anti) | 4.032 | br d 10.6(16") | 15 | | OH15, 13, 16', 16" | 12", 16', 16" |
| OH15 (syn) | 3.793 | br s | | | 15 | 16', 18 |
| C16 | 32.99 | | 16', 16" | | | |
| H16' (syn) | 2.617 | d 14.7(16"), q 3.5(10, 11', 15), d 0.7(?) | 16 | 10, 11, 15 | 10, 11', 15, 16" | OH15, 10, 15, 16", 18 |
| H16" (anti) | 1.915 | d 14.7(16'), d 10.6(15), d 2.9(10) | 16 | 9, 15 | 10, 15, 16' | 10, 11", 15, 16' |
| C17 | 31.53 | | 17 | 18 | | |
| H17 | 1.003 | s | 17 | 9, 13, 14, 18 | 18 | 7, 11', 12', 13, 18 |
| C18 | 29.58 | | 18 | 17 | | |
| H18 | 1.246 | s | 18 | 9, 13, 14, 17 | 17 | OH15, 13, 16', 17 |
| C19 | 149.40 | | | | | |
| C20 | 84.67 | | | 21 | | |
| C21 | 28.23 | | 21 | | | |
| H21 | 1.618 | s | 21 | 20 | | 4 |

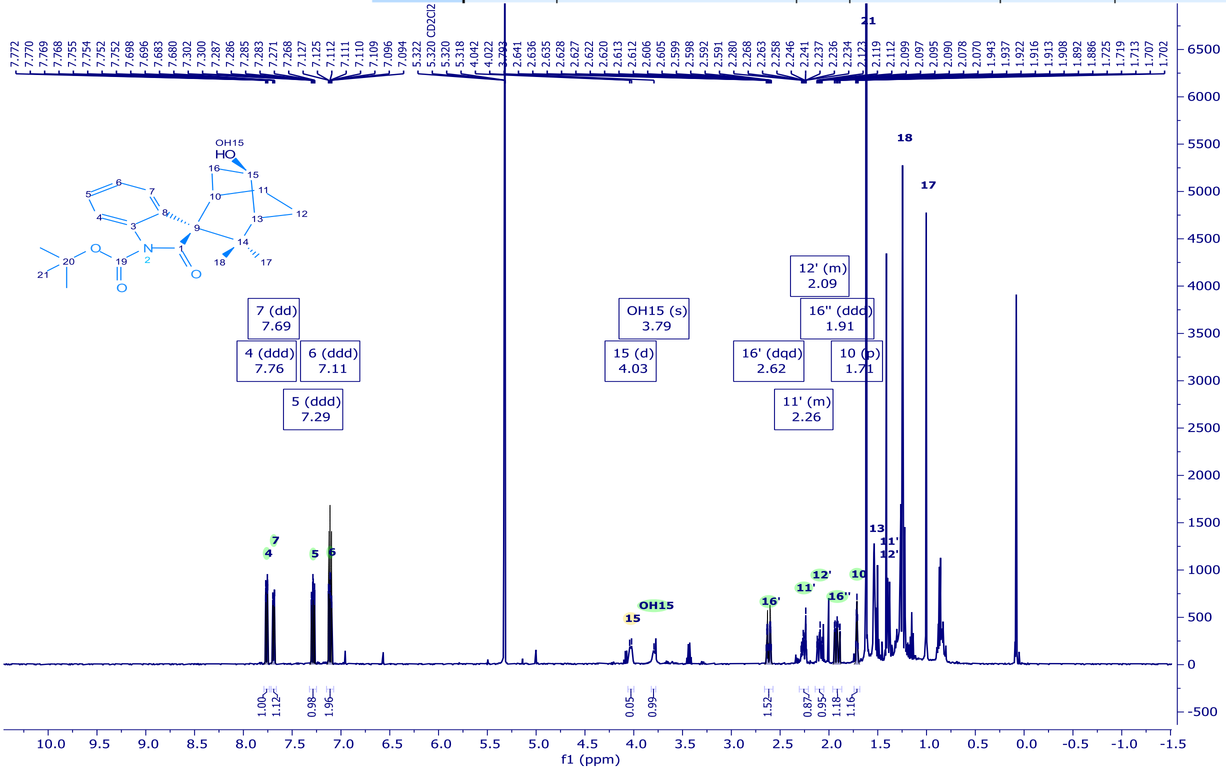

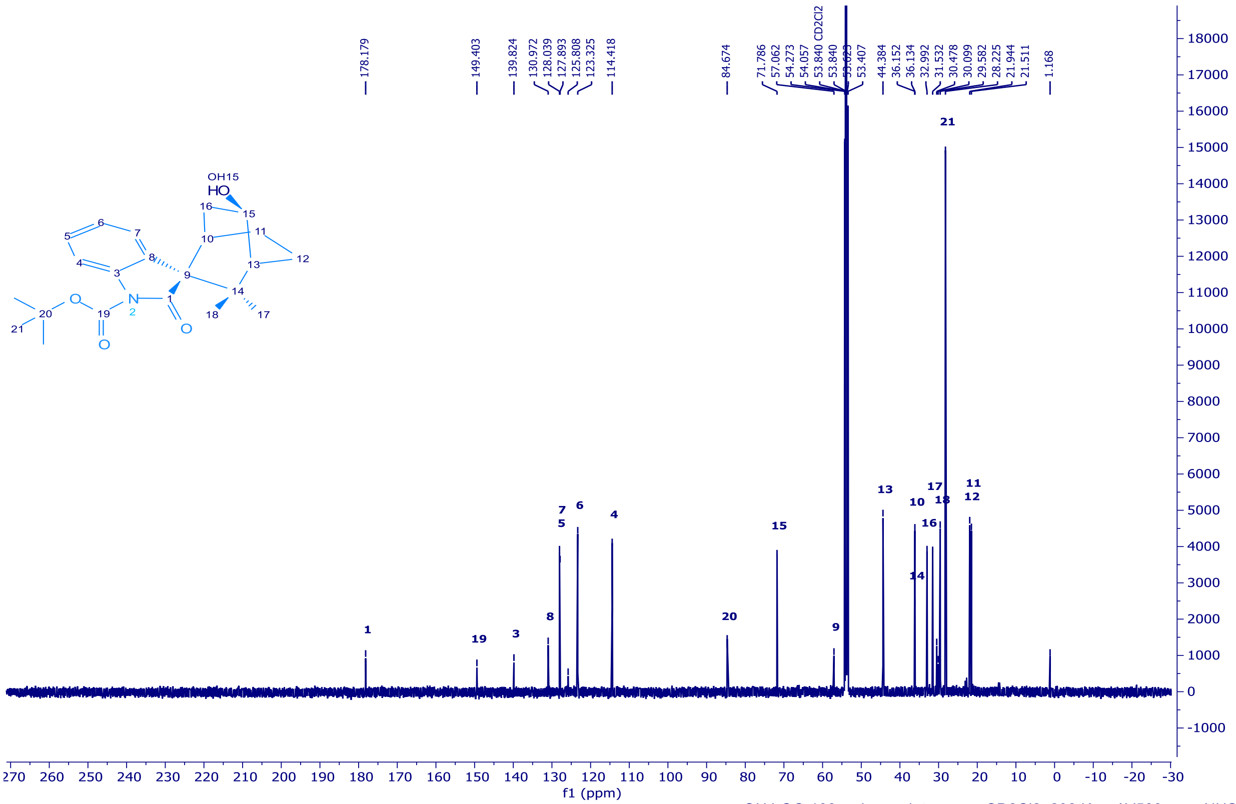
178.179
149.403
139.824
130.972
128.039
127.893
125.808
123.325
114.418
84.674
71.786
57.062
54.273
54.057
53.840 CD2Cl2
53.840
53.407
44.384
36.152
36.134
32.992
31.552
30.478
30.099
29.582
28.225
21.944
21.511
1.168
OH15
HO
f1 (ppm)

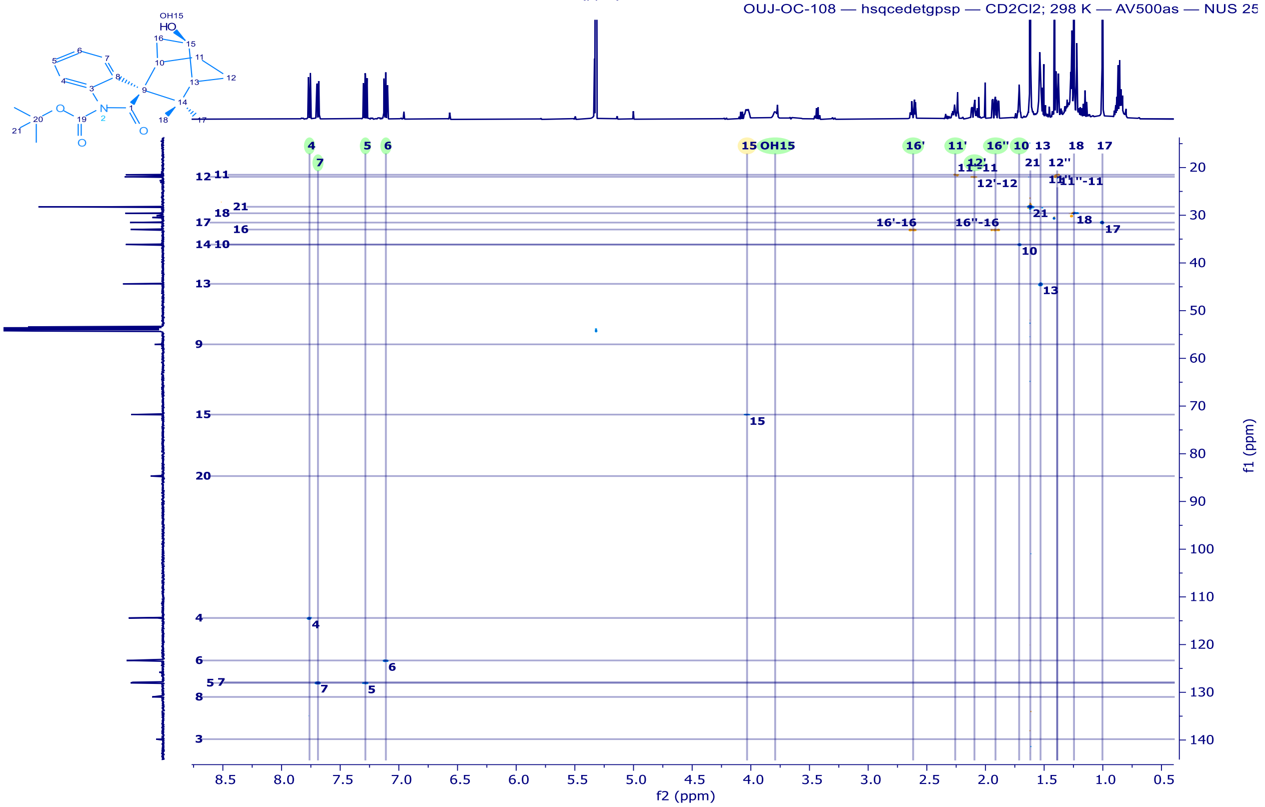
OUJ-OC-108 — hsqcedetgpsp — CD2Cl2; 298 K — AV500as — NUS 25
f2 (ppm)
f1 (ppm)

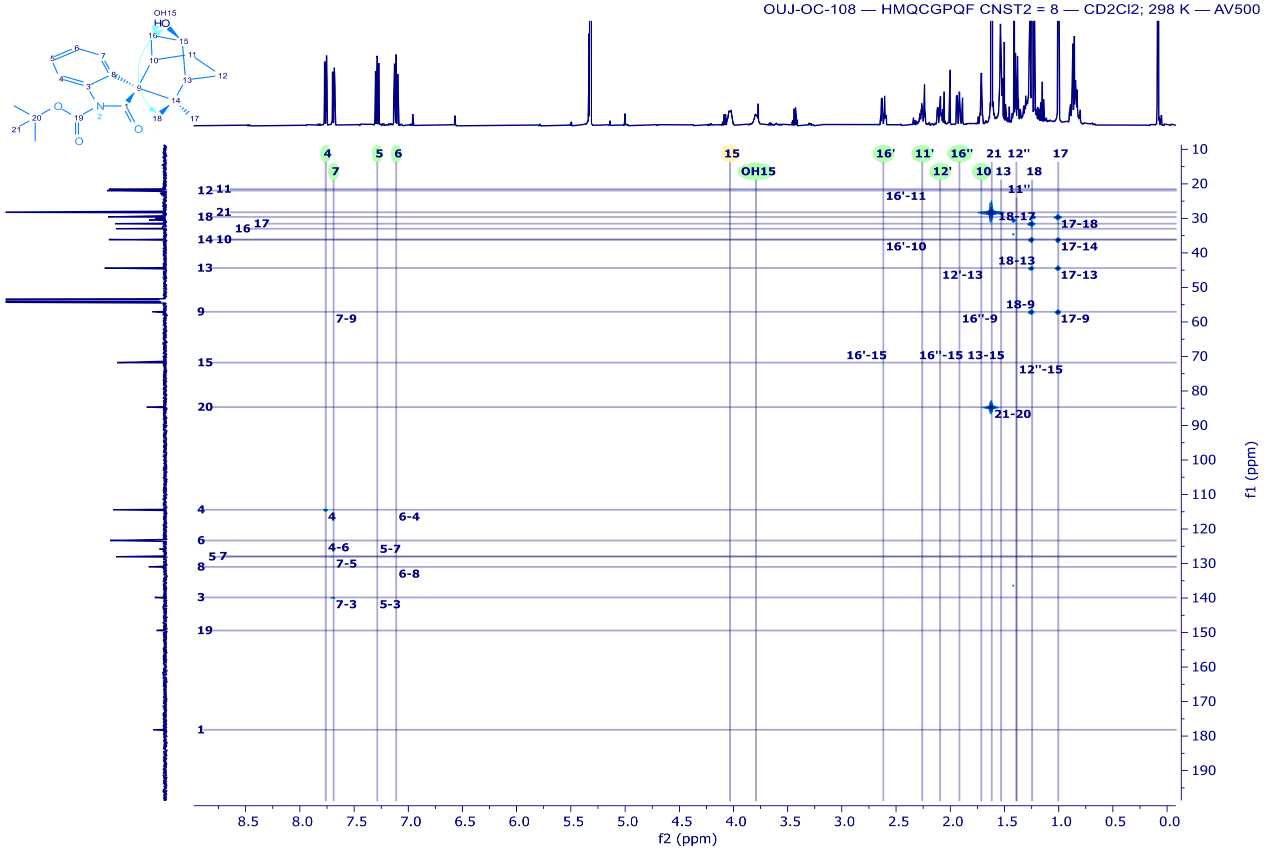
OUJ-OC-108 — HMQCGPQF CNST2 = 8 — CD2Cl2; 298 K — AV500
f2 (ppm)
f1 (ppm)

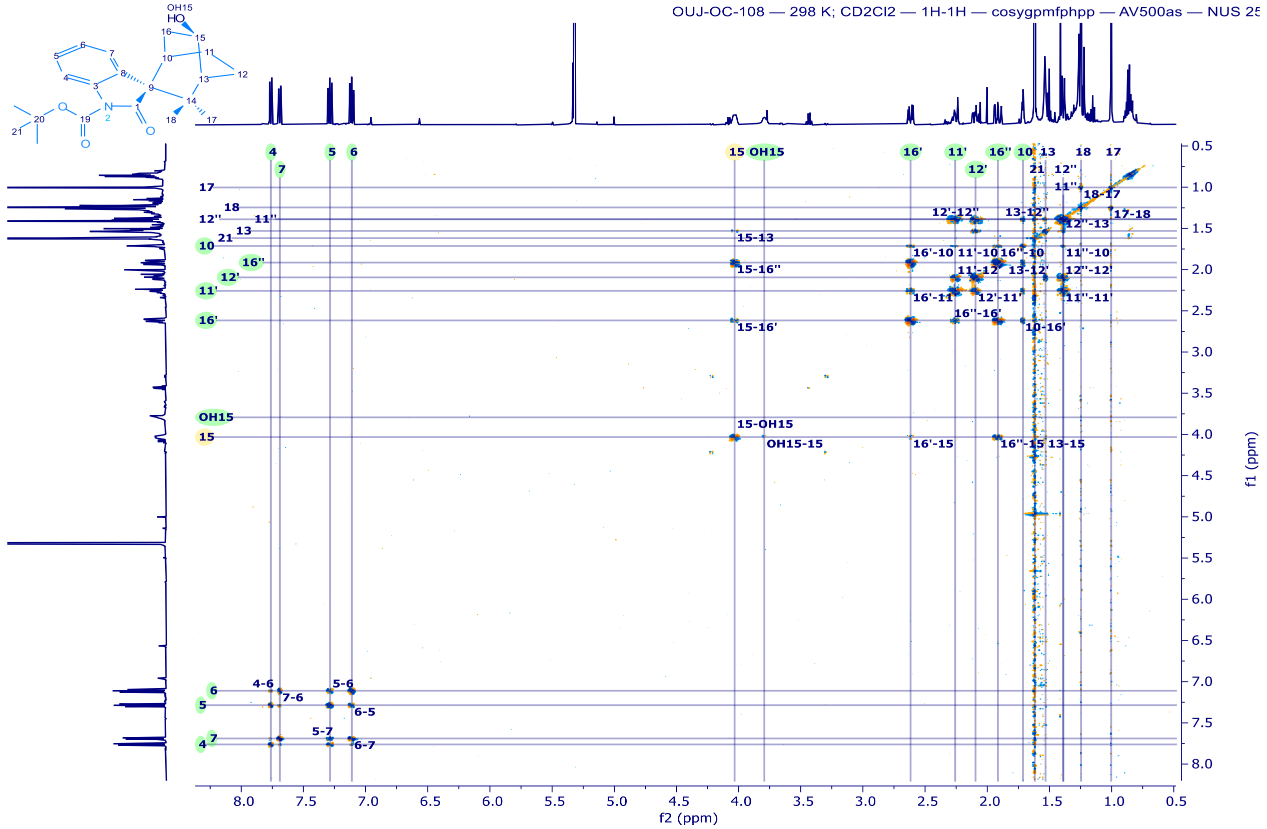
OUJ-OC-108 — 298 K; CD2Cl2 — 1H-1H — cosygpmfphpp — AV500as — NUS 25
f2 (ppm)
f1 (ppm)

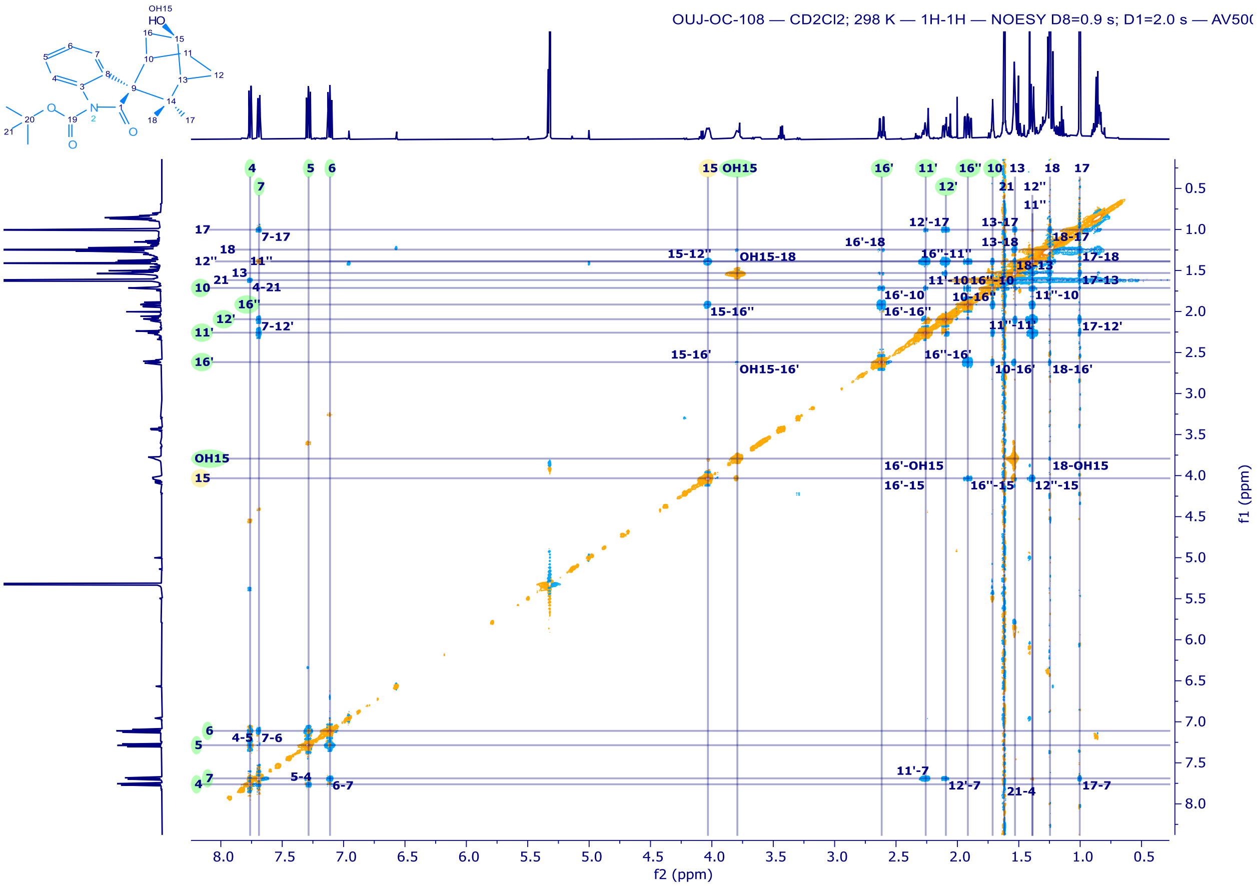


## Characterization of 15

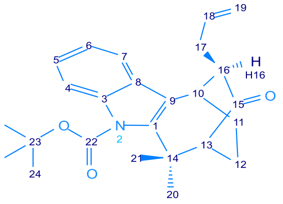

| Atom | δ (ppm) | J | HSQC | HMQC | COSY | NOESY |
|---|---|---|---|---|---|---|
| C1 | 140.85 | | | 10, 13, 20, 21 | | |
| C2 | 136.75 | | | 5, 7 | | |
| C4 | 114.22 | | 4 | 6 | | |
| H4 | 7.714 | d 1.0(6), d 8.3(5), d 0.7(7) | 4 | 6, 8 | 5, 6, 7 | 24 |
| C5 | 124.03 | | 5 | 7 | | |
| H5 | 7.215 | d 7.1(6), d 8.3(4), d 1.3(7) | 5 | 3, 7 | 4, 6, 7 | 24 |
| C6 | 121.98 | | 6 | 4 | | |
| H6 | 7.155 | d 7.1(5), d 1.0(4), d 7.8(7) | 6 | 4, 8 | 4, 5, 7 | |
| C7 | 118.72 | | 7 | 5 | | |
| H7 | 7.425 | d 7.8(6), d 1.3(5), t 0.6(4, ?) | 7 | 3, 5, 9 | 4, 5, 6 | 10, 17b, 18, 19cis, 19trans |
| C8 | 128.97 | | | 4, 6, 10 | | |
| C9 | 121.89 | | | 16, 7, 10 | | |
| C10 | 29.71 | | 10 | 16, 17b | | |
| H10 | 3.501 | d 5.2(11a/b), d 1.8(11a/b), d 3.4(16) | 10 | 1, 8, 9, 11, 12, 15, 16 | 16, 11a, 11b | 16, 7, 11a, 11b, 17b, 18, 19cis, 19trans |
| C11 | 28.10 | | 11a, 11b | 16, 10, 13 | | |
| H11a | 2.096 | m | 11 | 12 | 10, 11b, 12a, 12b | 16, 10 |
| H11b | 2.033 | m(o.l.) | 11 | 12, 14, 16 | 10, 11a, 12a, 12b | 10, 12a, 20 |
| C12 | 20.19 | | 12a, 12b | 10, 11a, 11b, 13 | | |
| H12a | 2.338 | m | 12 | 15 | 11a, 11b, 12b, 13 | 11b, 12b, 20 |
| H12b | 2.017 | m(o.l.) | 12 | | 11a, 11b, 12a, 13 | 16, 12a, 13 |
| C13 | 61.78 | | 13 | 20, 21 | | |
| H13 | 2.317 | m(o.l.) | 13 | 1, 11, 12, 14, 15, 16 | 12a, 12b | 16, 12b, 20, 21 |
| C14 | 41.42 | | | 11b, 13, 20, 21 | | |
| C15 | 214.53 | | | 16, 10, 12a, 13 | | |
| C16 | 54.24 | | 16 | 10, 11b, 13, 17b | | |
| H16 | 2.616 | d 10.4(17b), t 3.5(10, 17a) | 16 | 9, 10, 11, 15, 17, 18 | 10, 17a, 17b | 10, 11a, 12b, 13, 18, 19trans |
| C17 | 32.71 | | 17a, 17b | 16, 18, 19trans | | |
| H17a | 2.561 | d 3.6(16), d 14.7(17b), d 5.4(18), t 1.9 (19cis,trans) | 17 | | 16, 17b, 18, 19cis, 19trans | 17b |
| H17b | 1.592 | d 10.4(16), d 8.4(18), d 14.7(17a), t 1.0(19cis,trans) | 17 | 10, 16, 18, 19 | 16, 17a, 18, 19trans | 7, 10, 17a, 19trans |
| C18 | 137.48 | | 18 | 16, 17b | | |
| H18 | 5.863 | d 8.4(17b), d 5.4(17a), d 10.2(19cis), d 17.1(19trans) | 18 | 17 | 17a, 17b, 19cis, 19trans | 16, 7, 10 |
| C19 | 116.76 | | 19cis, 19trans | 17b | | |
| H19cis | 5.057 | d 0.8(17b), d 10.2(18), t 1.8(17a, 19trans) | 19 | | 17a, 18, 19trans | 7, 10 |
| H19trans | 4.915 | d 1.1(17b), d 17.1(18), t 1.9(17a, 19cis) | 19 | 17 | 17a, 17b, 18, 19cis | 16, 7, 10, 17b |
| C20 | 26.88 | | 20 | 21 | | |
| H20 | 1.645 | s | 20 | 1, 13, 14, 21 | 21 | 11b, 12a, 13, 21 |
| C21 | 28.33 | | 21 | 20 | | |
| H21 | 1.502 | s | 21 | 1, 13, 14, 20 | 20 | 13, 20, 24 |
| C22 | 151.73 | | | | | |
| C23 | 84.60 | | | 24 | | |
| C24 | 28.26 | | 24 | | | |
| H24 | 1.689 | s | 24 | 23 | | 4, 5, 21 |

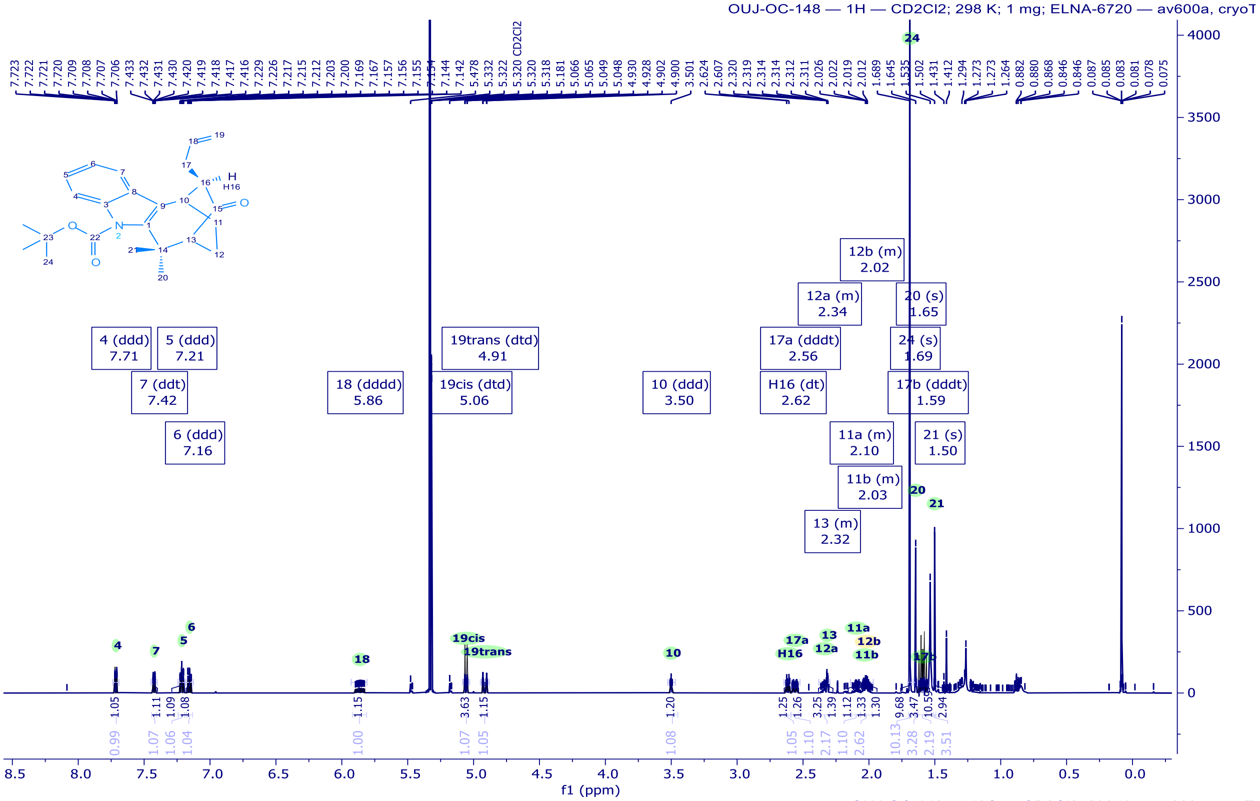
OUJ-OC-148 — 1H — CD2Cl2; 298 K; 1 mg; ELNA-6720 — av600a, cryoT
4 (ddd) 7.71
5 (ddd) 7.21
7 (ddt) 7.42
6 (ddd) 7.16
18 (dddd) 5.86
19trans (dtd) 4.91
19cis (dtd) 5.06
10 (ddd) 3.50
17a (dddt) 2.56
H16 (dt) 2.62
12a (m) 2.34
12b (m) 2.02
11a (m) 2.10
11b (m) 2.03
13 (m) 2.32
20 (s) 1.65
24 (s) 1.69
17b (dddt) 1.59
21 (s) 1.50
f1 (ppm)

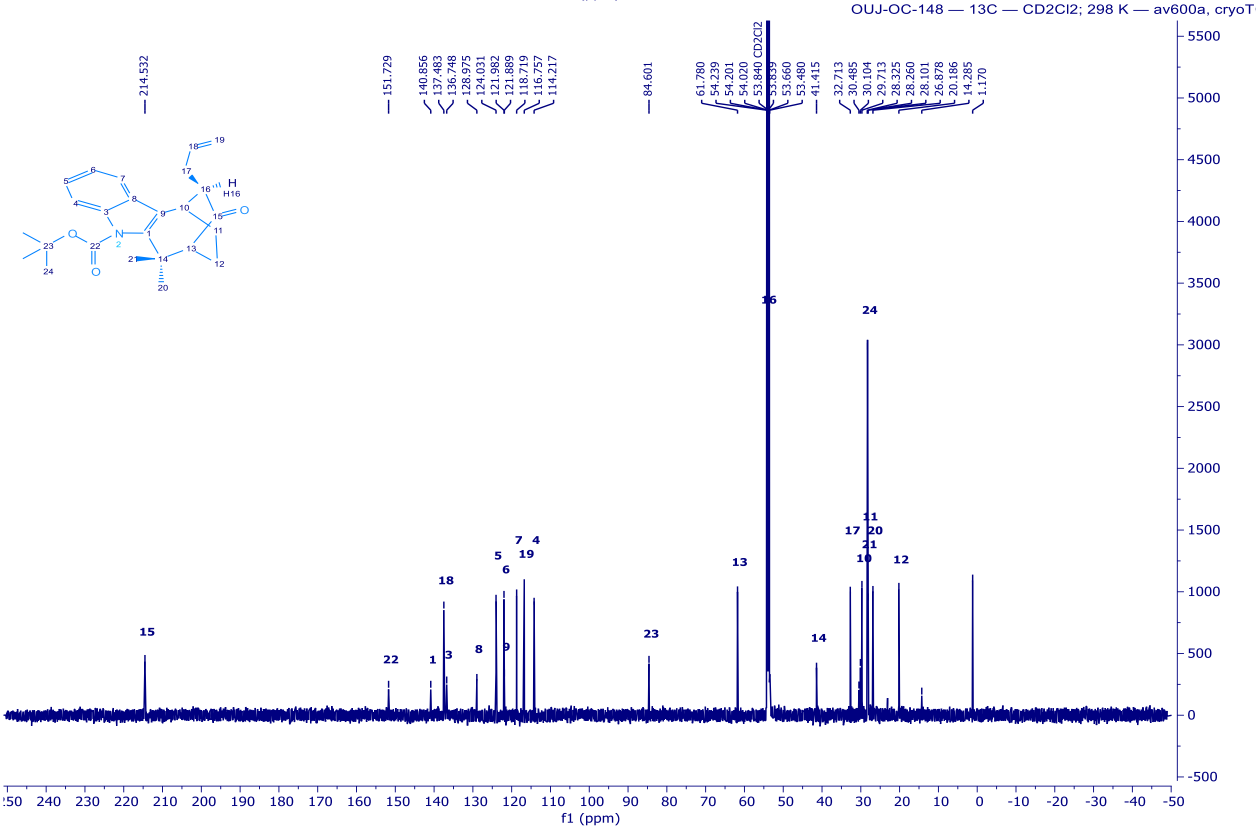
OUJ-OC-148 — 13C — CD2Cl2; 298 K — av600a, cryoT
f1 (ppm)

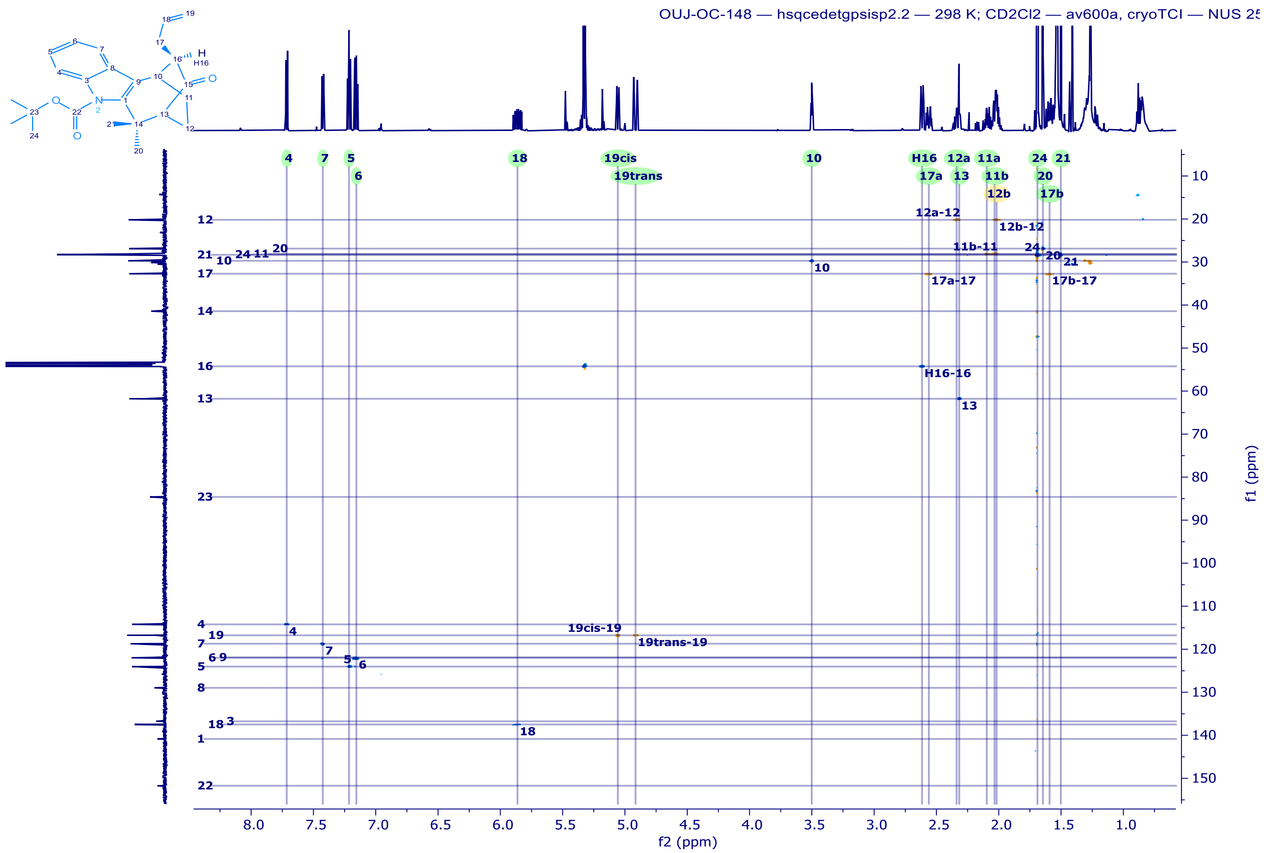
OUJ-OC-148 — hsqcedetgpsisp2.2 — 298 K; CD2Cl2 — av600a, cryoTCI — NUS 25
f2 (ppm)
f1 (ppm)

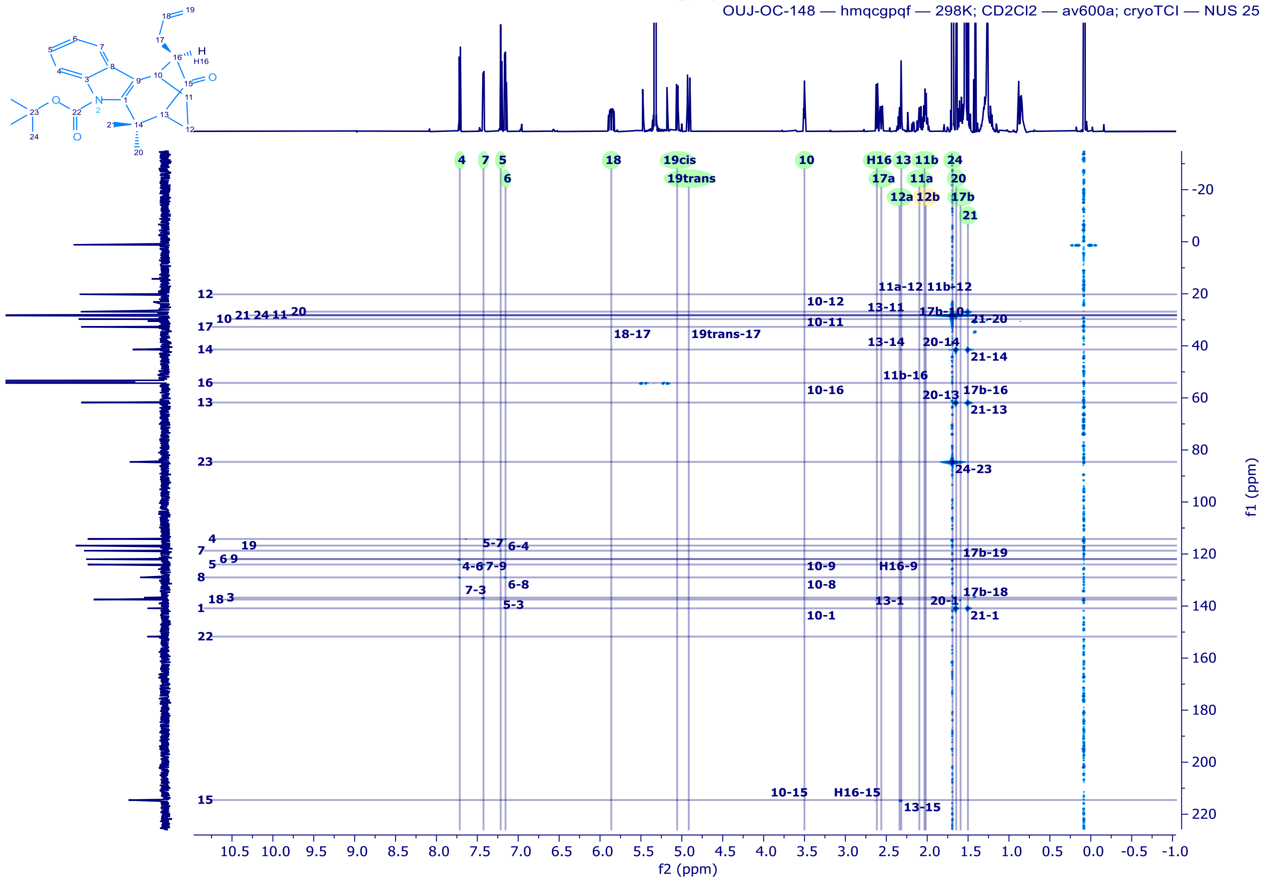
OUJ-OC-148 — hmqcgpqf — 298K; CD2Cl2 — av600a; cryoTCI — NUS 25
f2 (ppm)
f1 (ppm)

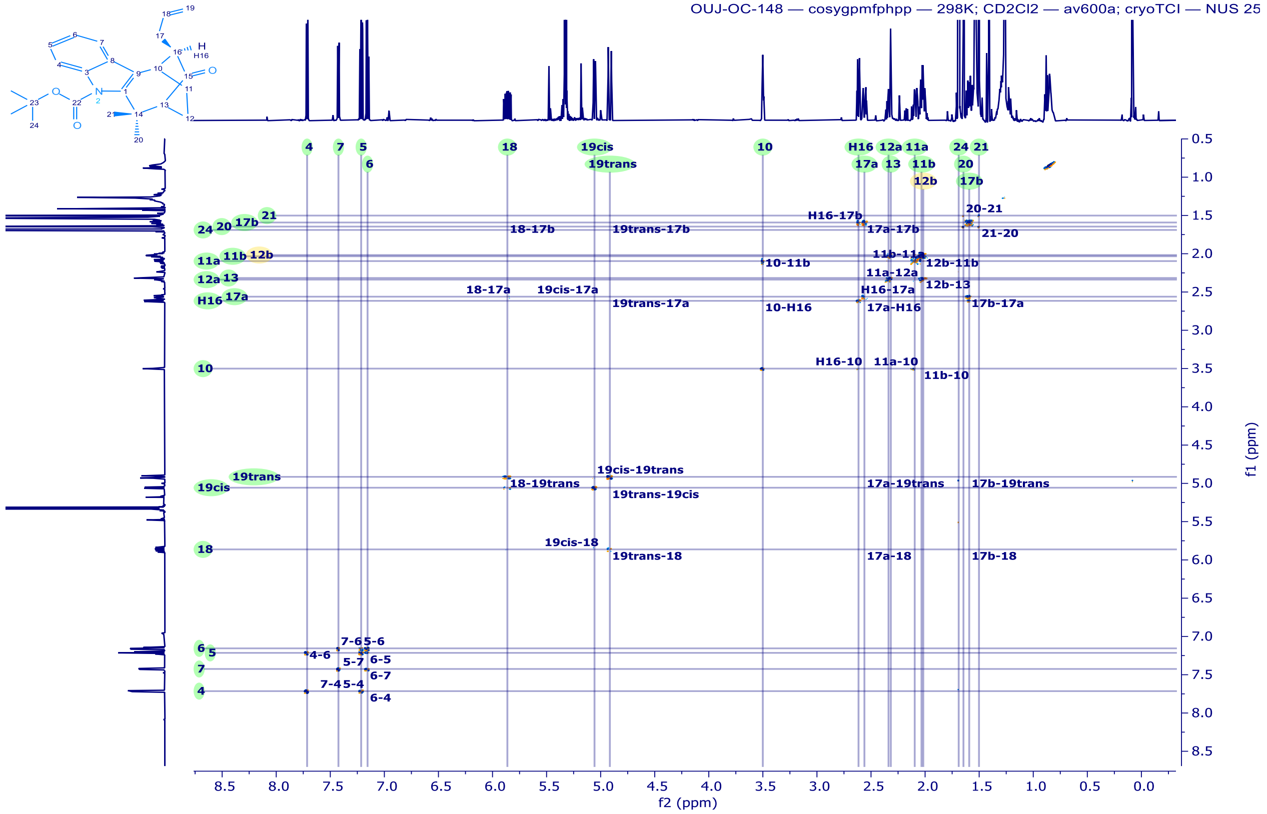
OUJ-OC-148 — cosygpmfphpp — 298K; CD2Cl2 — av600a; cryoTCI — NUS 25
f2 (ppm)
f1 (ppm)

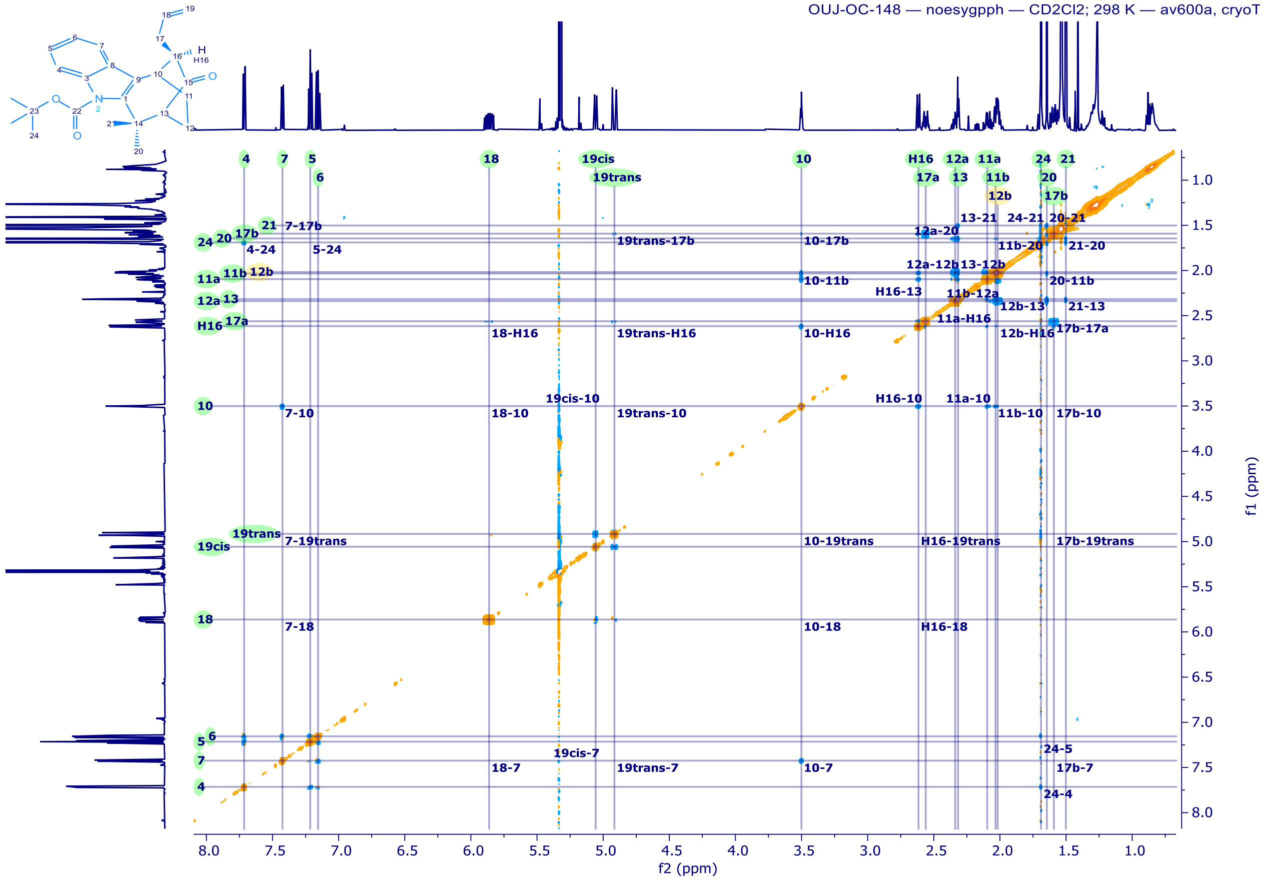
OUJ-OC-148 — noesygpph — CD2Cl2; 298 K — av600a, cryoT
f2 (ppm)
f1 (ppm)

NMR spectra for compound **(S)- 5,5''',6,6''',7,7''',8,8'''-octahydro-[2,2':4',1'':3'',2'''-quaternaphthalene]-2'',3'-diol**: $^{1}$H (501 MHz) and $^{13}$C (126 MHz) in $CD_2Cl_2$.

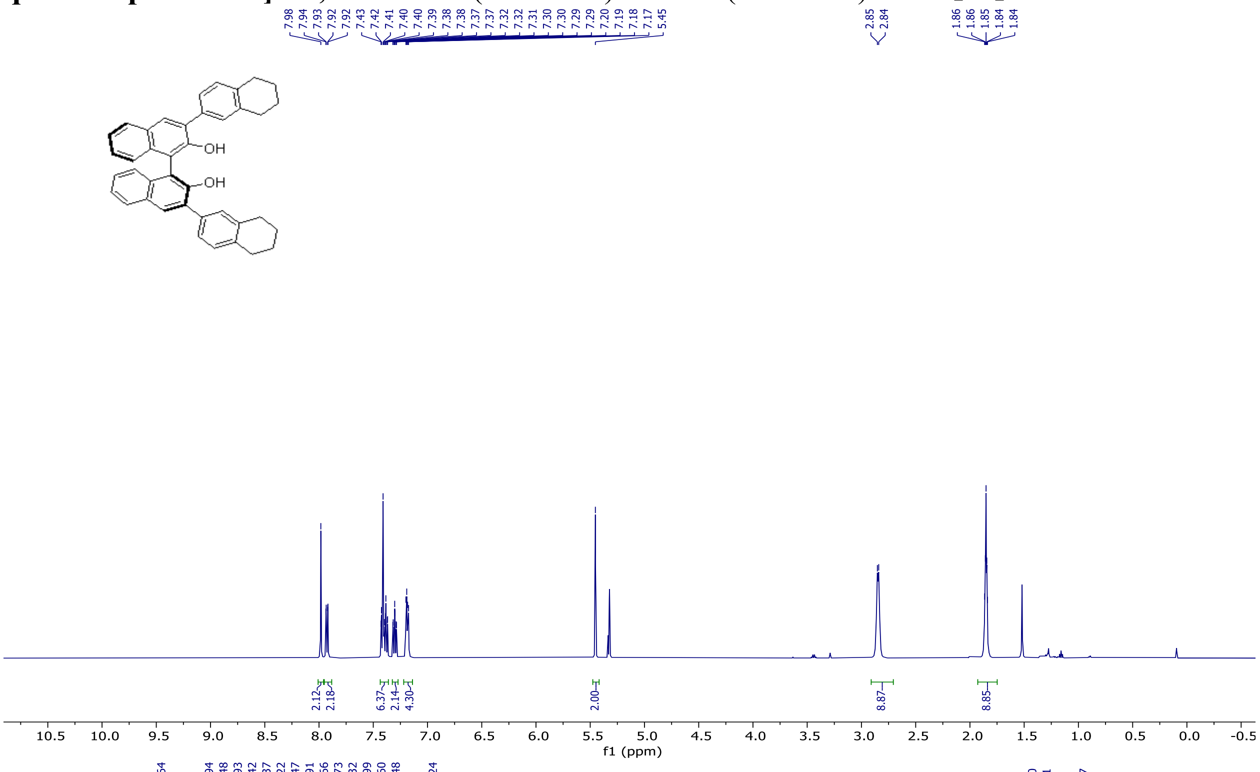


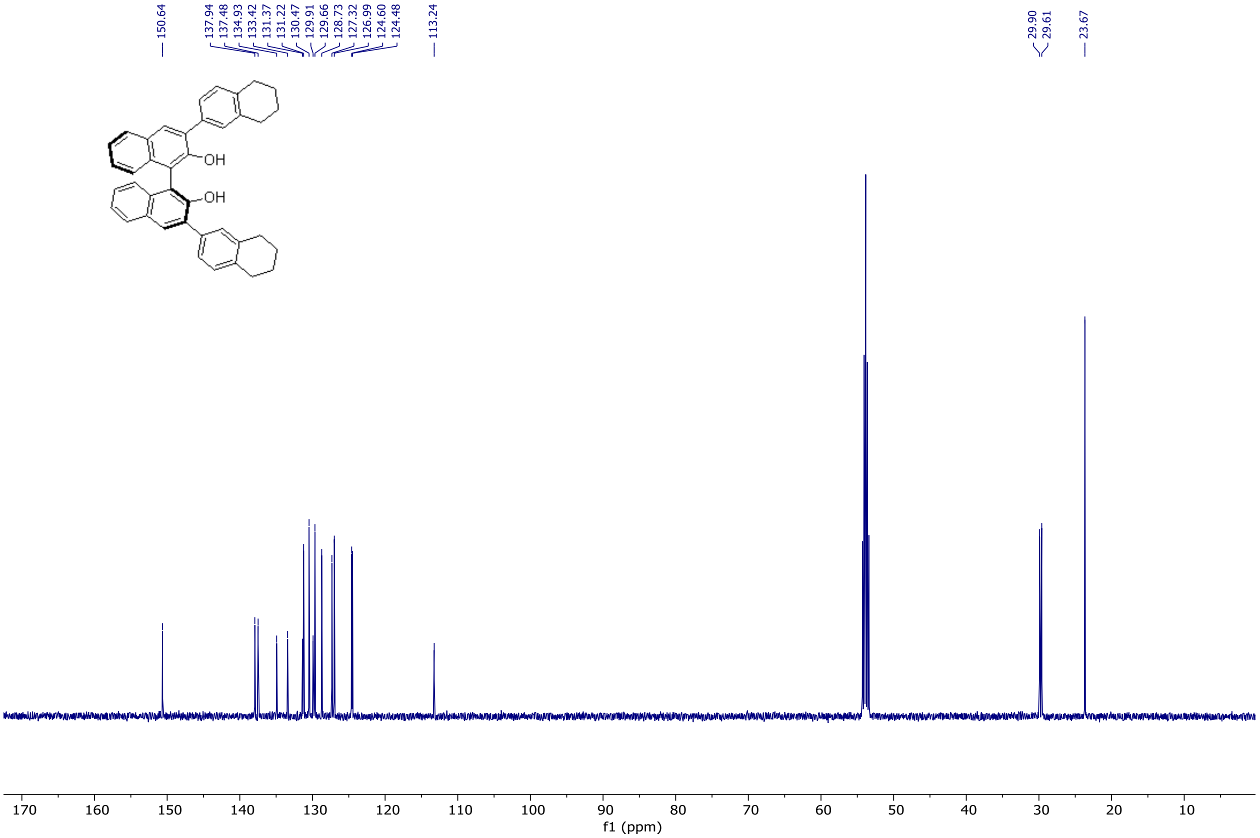

NMR spectra for Catalyst **6b** : $^{19}$F NMR (471 MHz) in $CD_2Cl_2$.

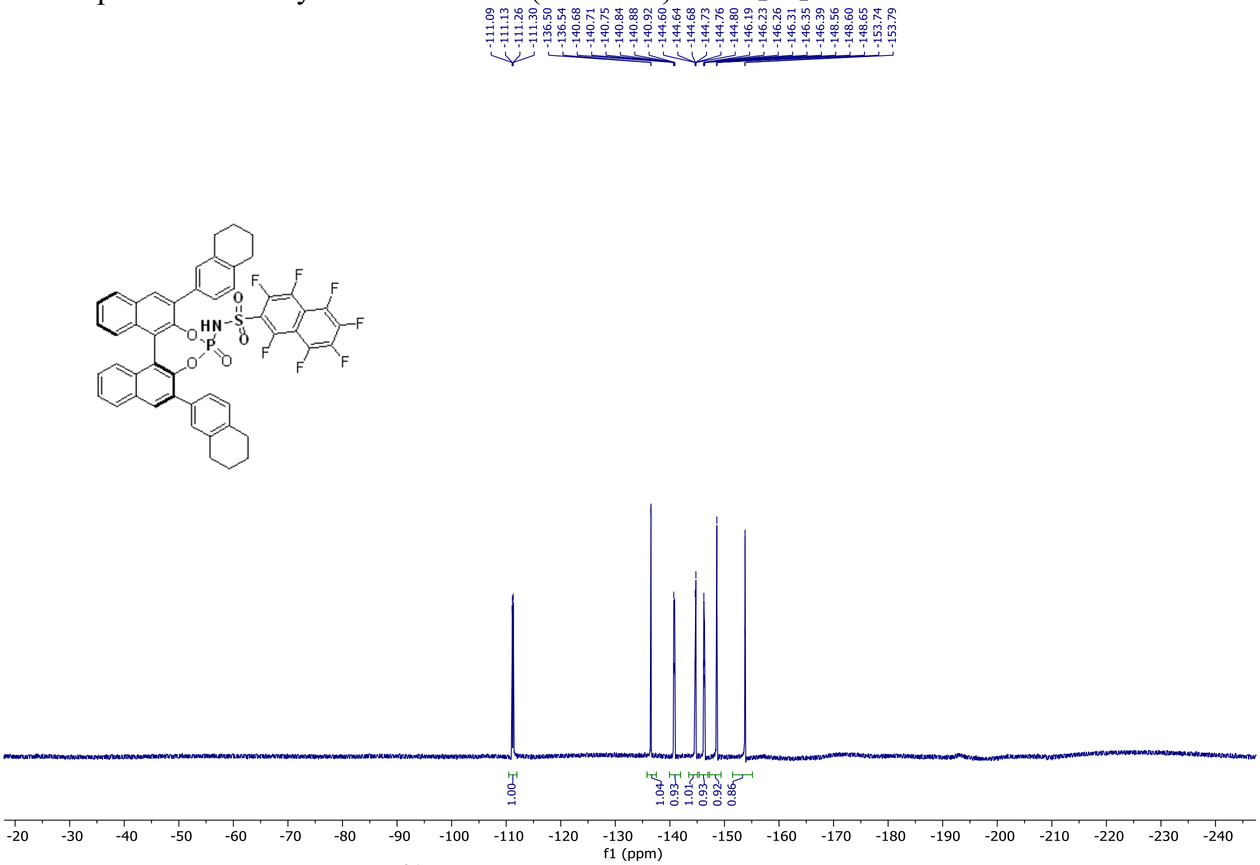


NMR spectra for Catalyst **6b** : $^{31}$P NMR (203 MHz) in $CD_2Cl_2$.

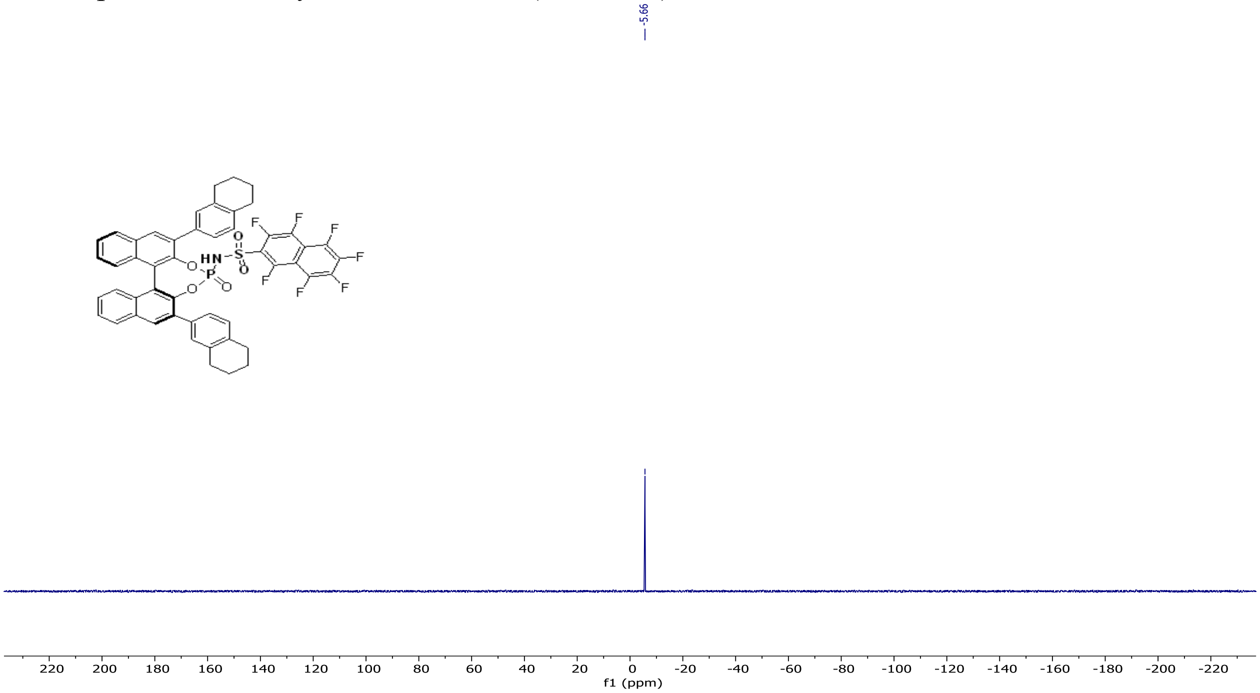

NMR spectra for Catalyst **6b** : $^{1}H$ NMR (501 MHz) in $CD_2Cl_2$.

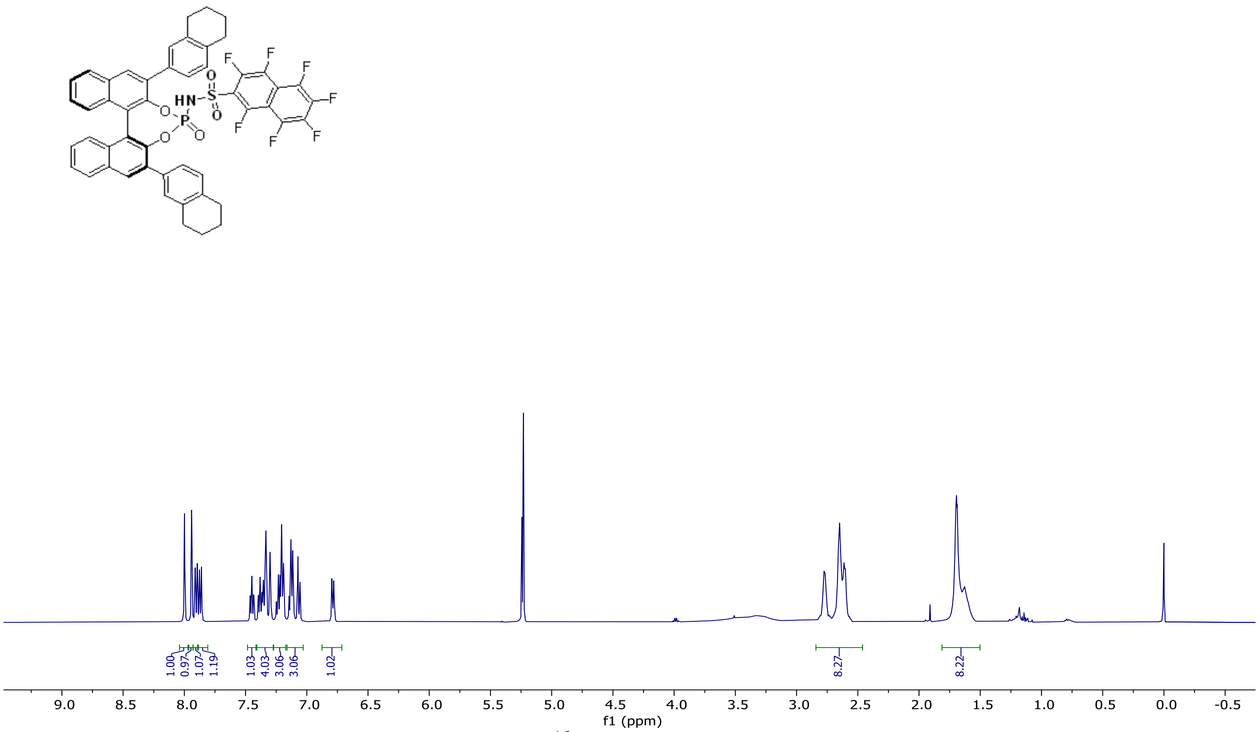


NMR spectra for compound  Catalyst **6b** : $^{13}C$ NMR (126 MHz) in $CD_2Cl_2$.

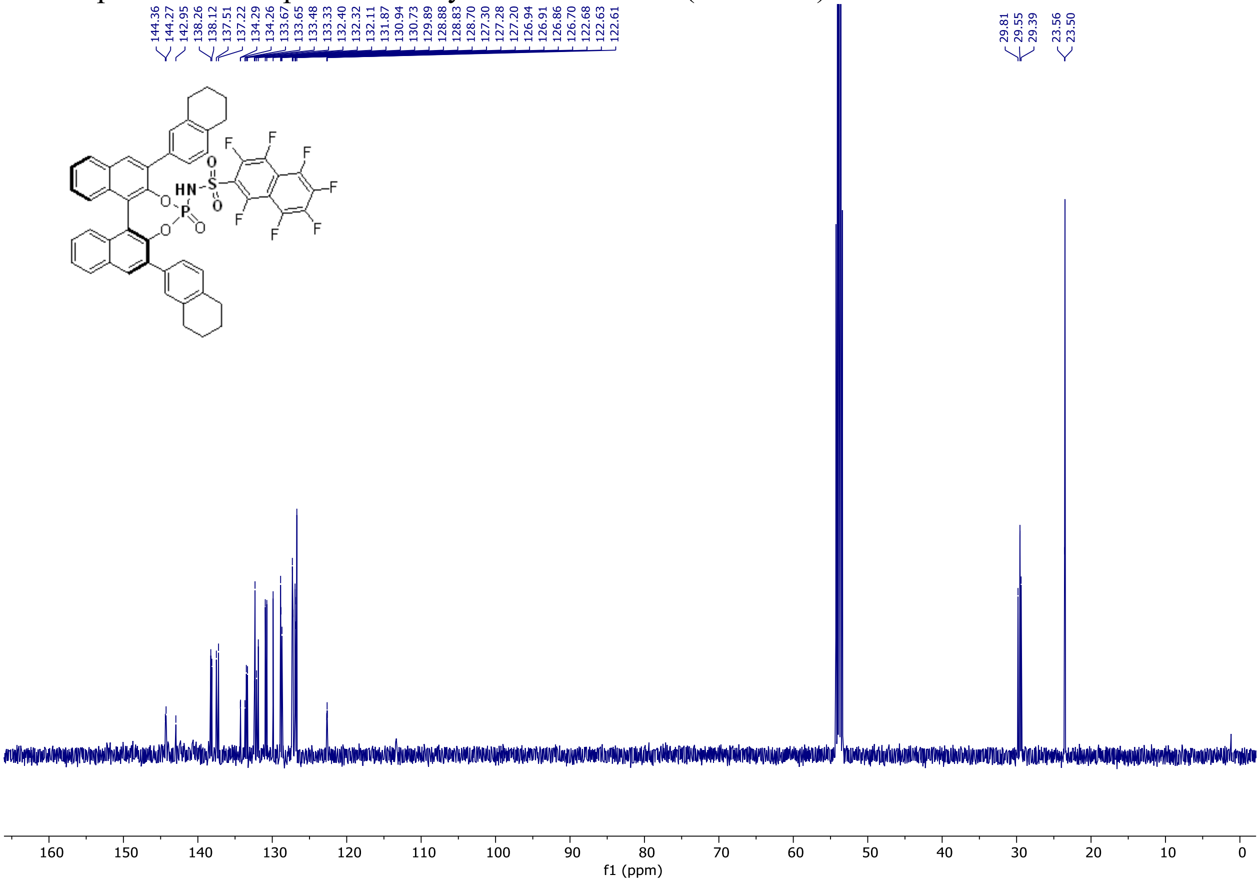

NMR spectra for IDPi-**6f** : $^{1}$H (501 MHz) in $CD_2Cl_2$.

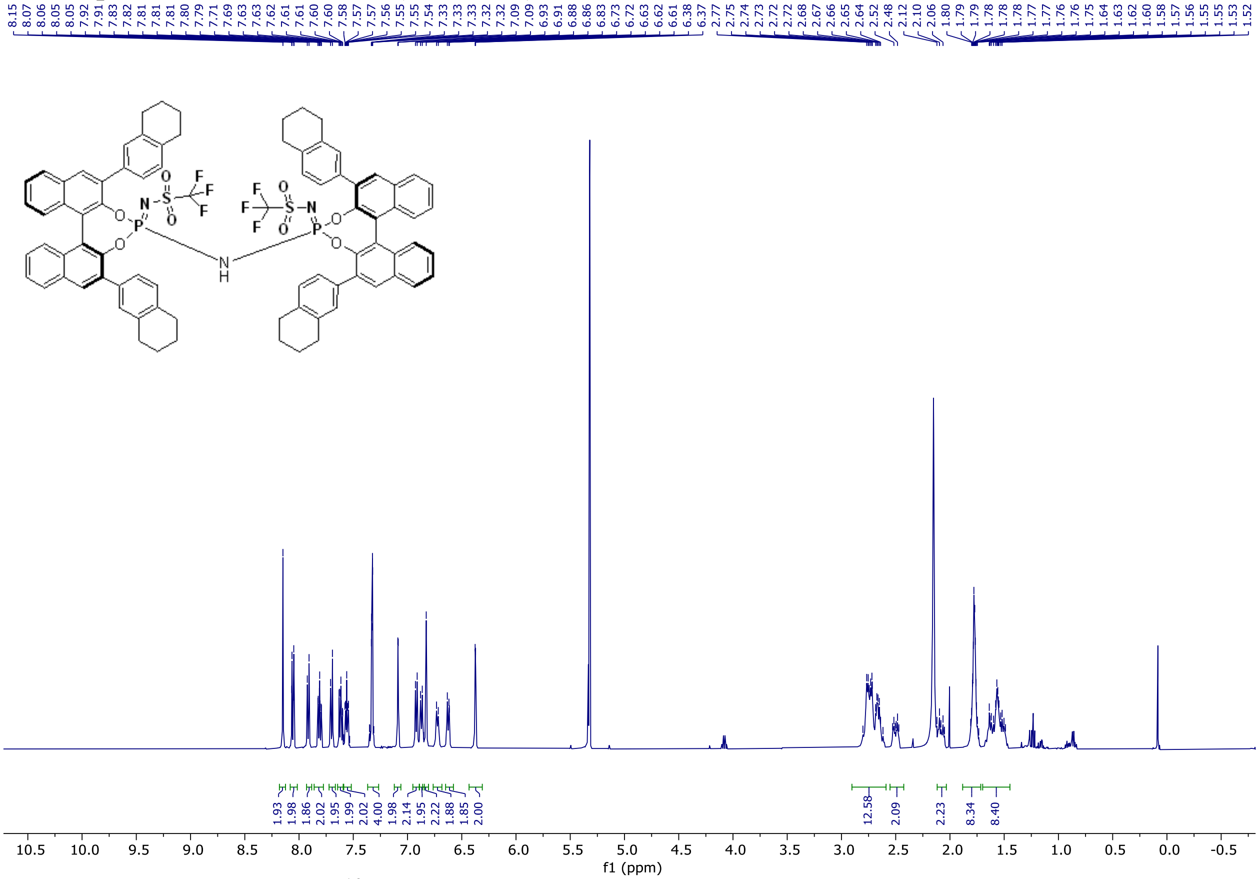


NMR spectra for IDPi-**6f**: $^{19}$F NMR (471 MHz) in $CD_2Cl_2$.

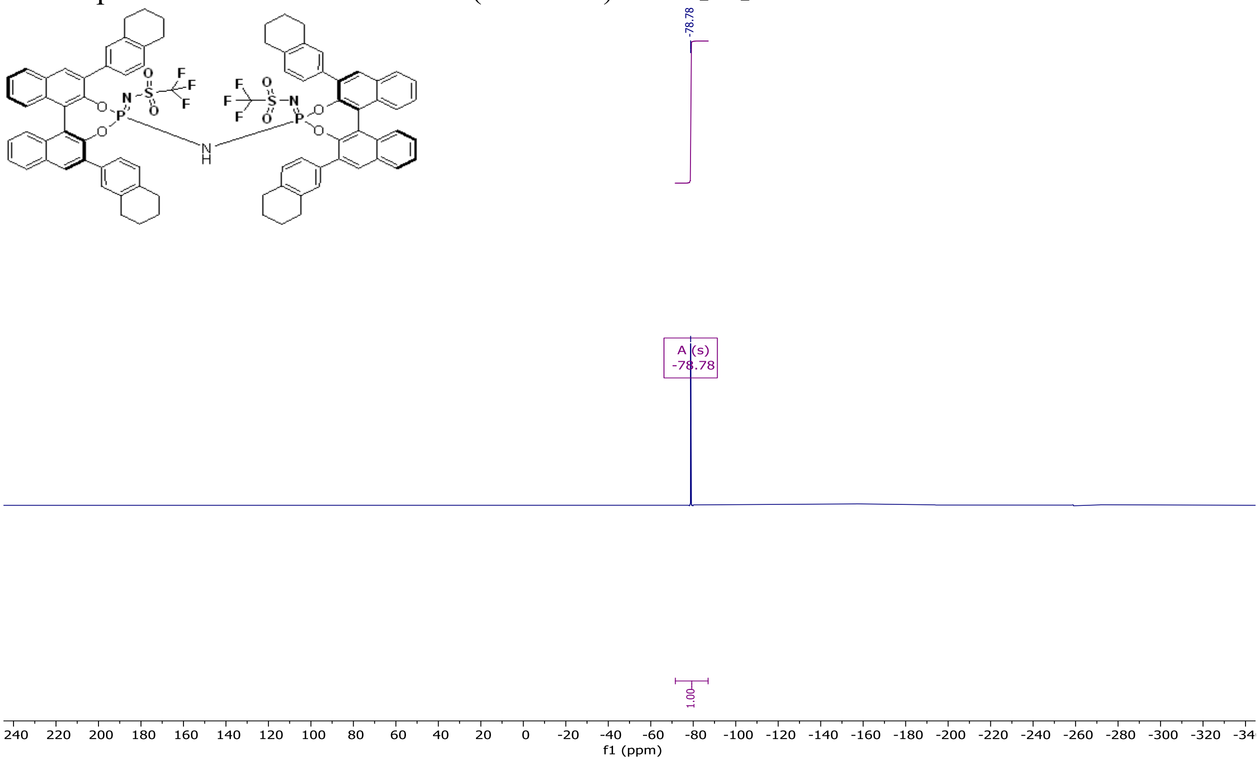

NMR spectra for IDPi-**6f**: $^{31}P$ NMR (203 MHz) in $CD_2Cl_2$.

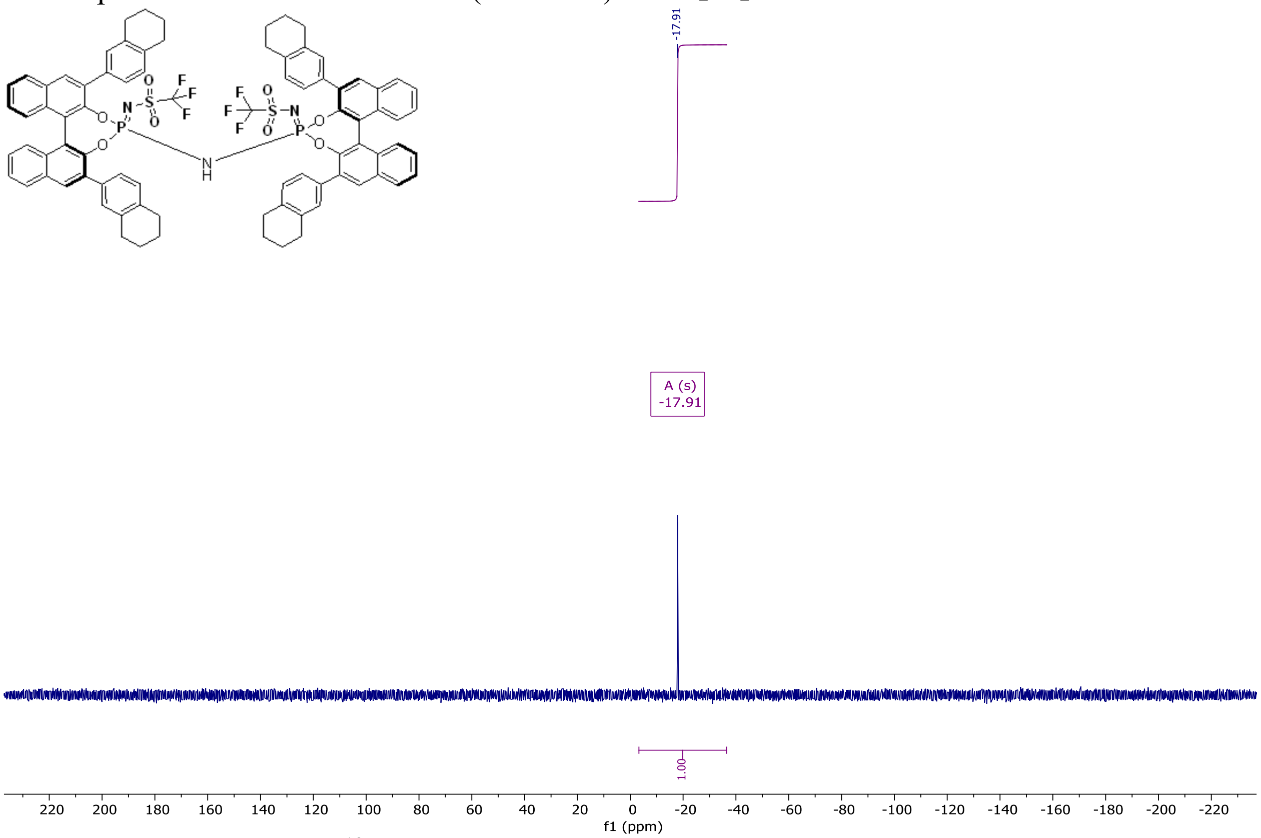


NMR spectra for IDPi-**6f**: $^{13}C$ NMR (126 MHz) in $CD_2Cl_2$.

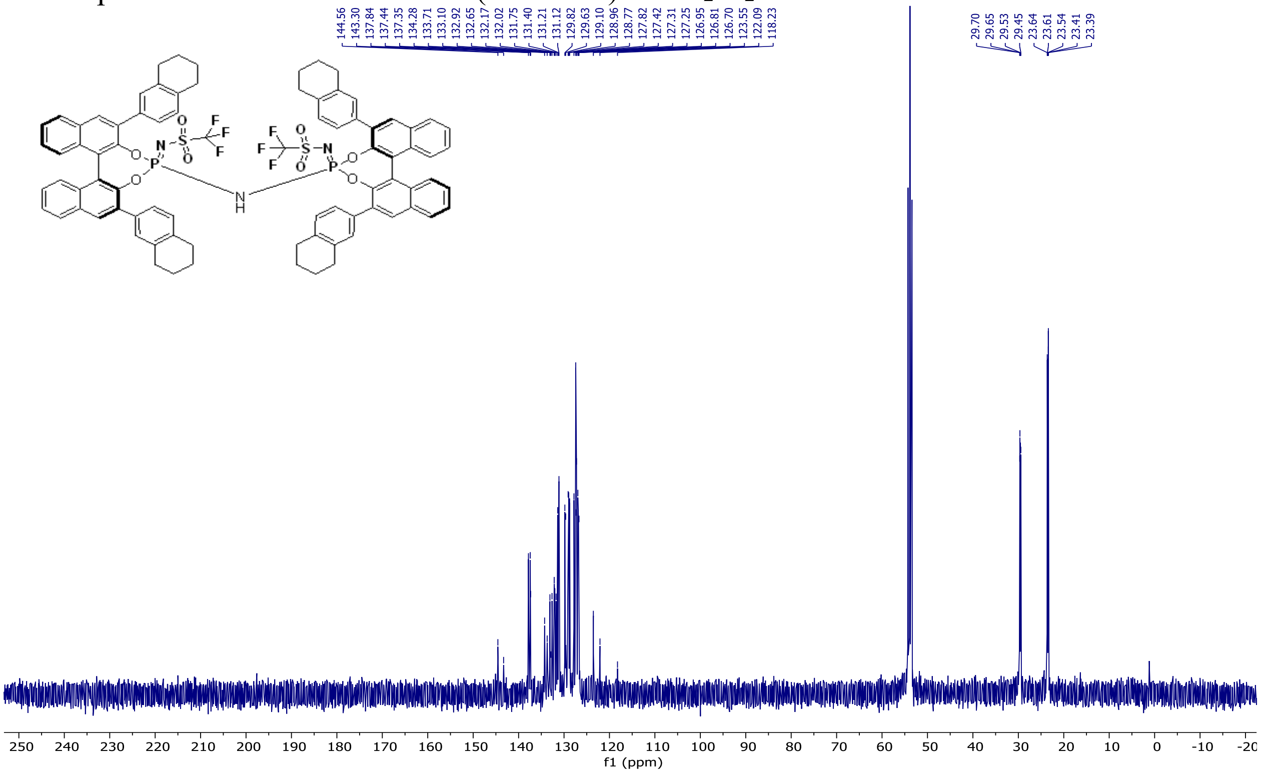

NMR spectra for IDPi-**6g** : $^{1}H$ NMR (501 MHz) in $CD_2Cl_2$.

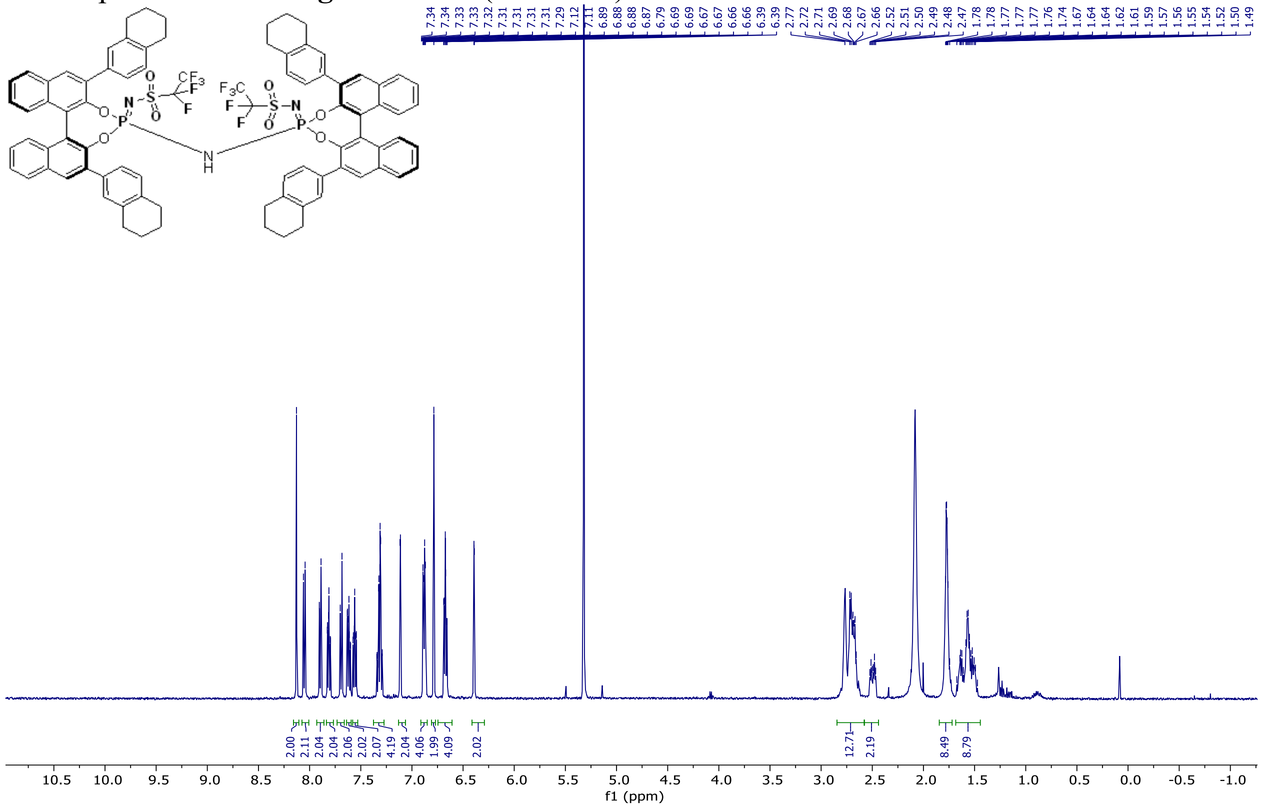


NMR spectra for IDPi-**6g** : $^{19}F$ NMR(471 MHz) in $CD_2Cl_2$.

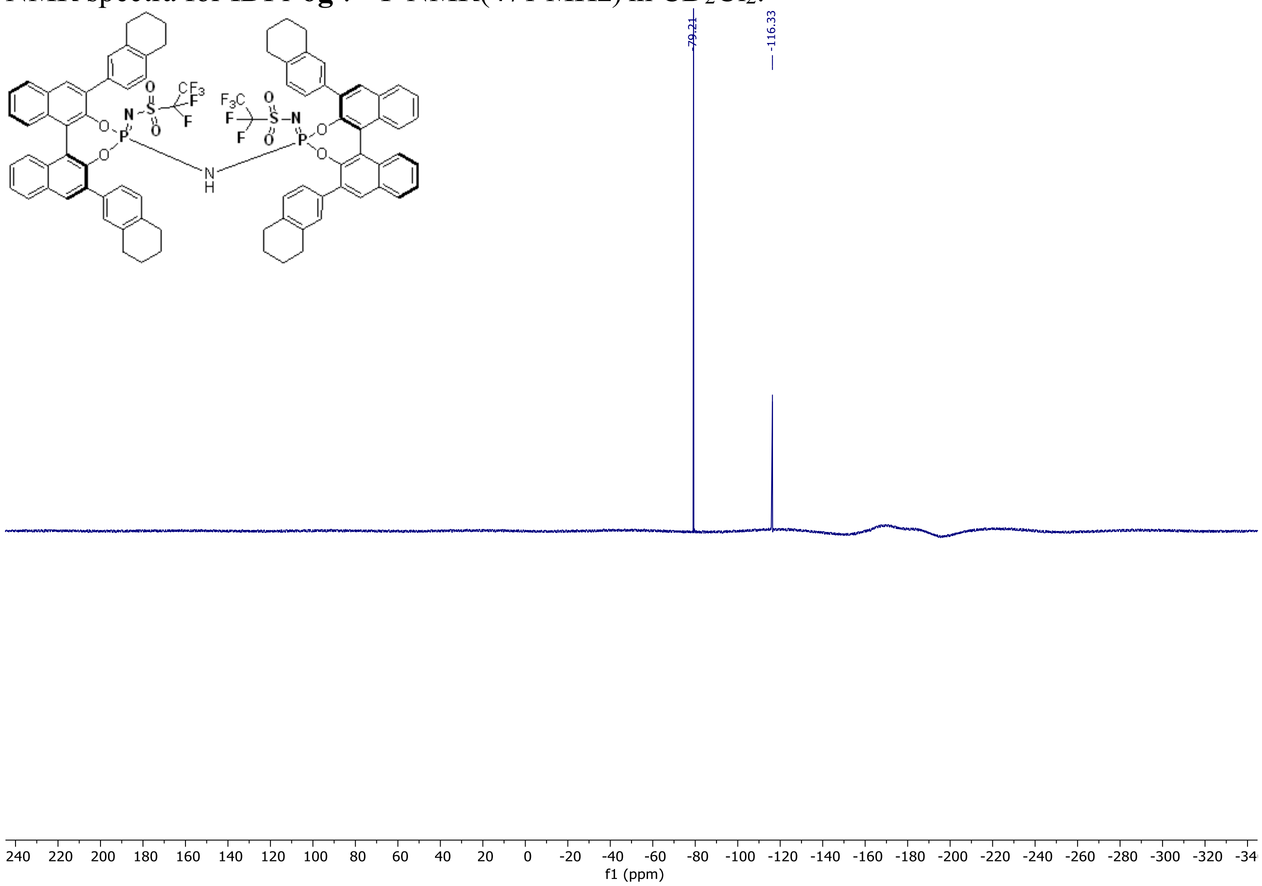

NMR spectra for IDPi-**6g** : $^{31}$P NMR (203 MHz) in $CD_2Cl_2$.

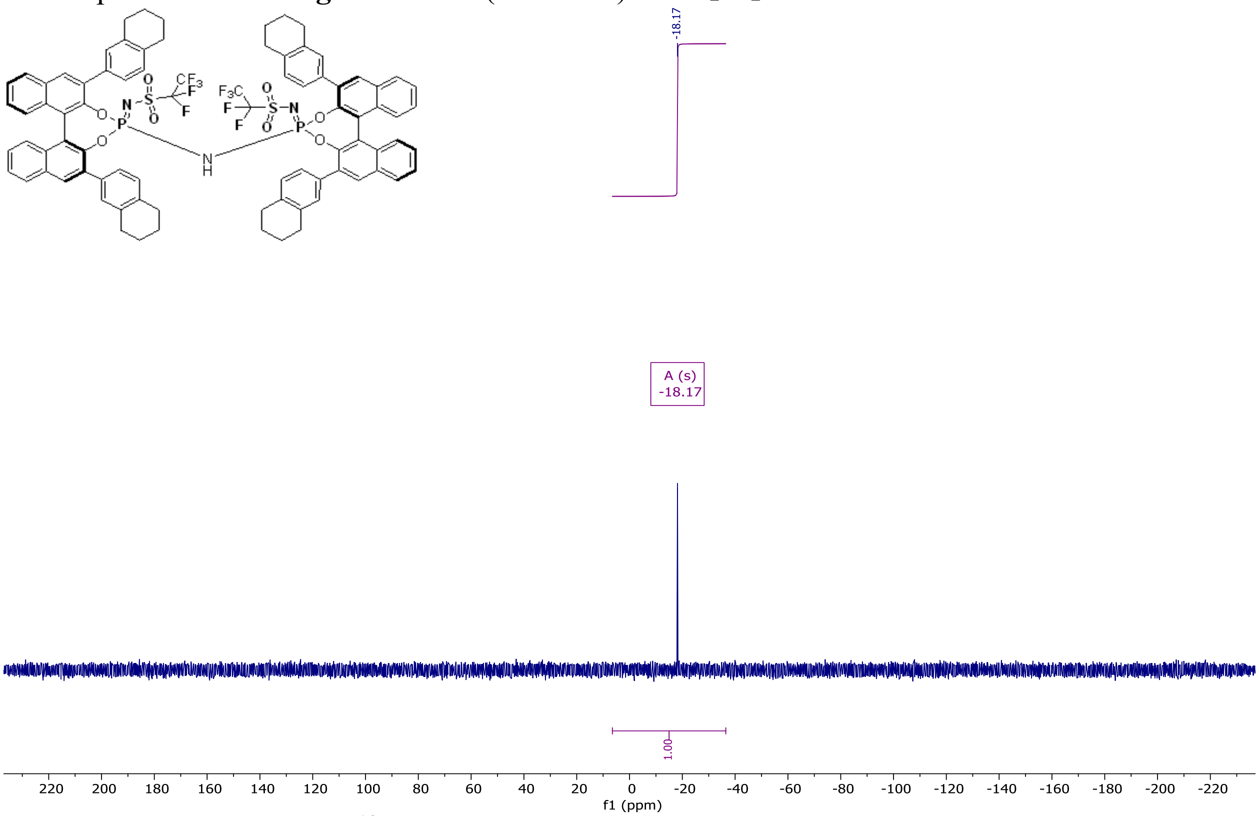

NMR spectra for IDPi-**6g** : $^{13}$C NMR (126 MHz) in $CD_2Cl_2$.

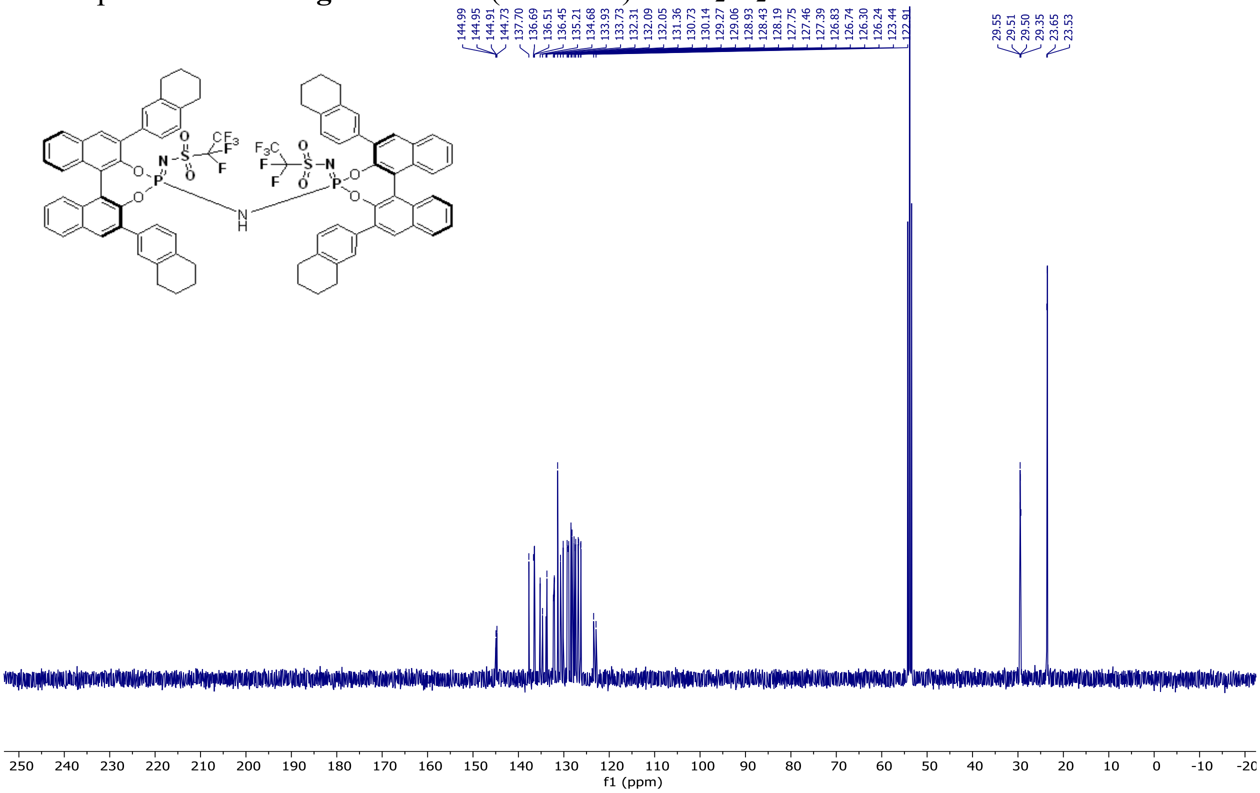

NMR spectra for IDPi-**6h** : $^{31}$P NMR (203 MHz) in $CD_2Cl_2$.

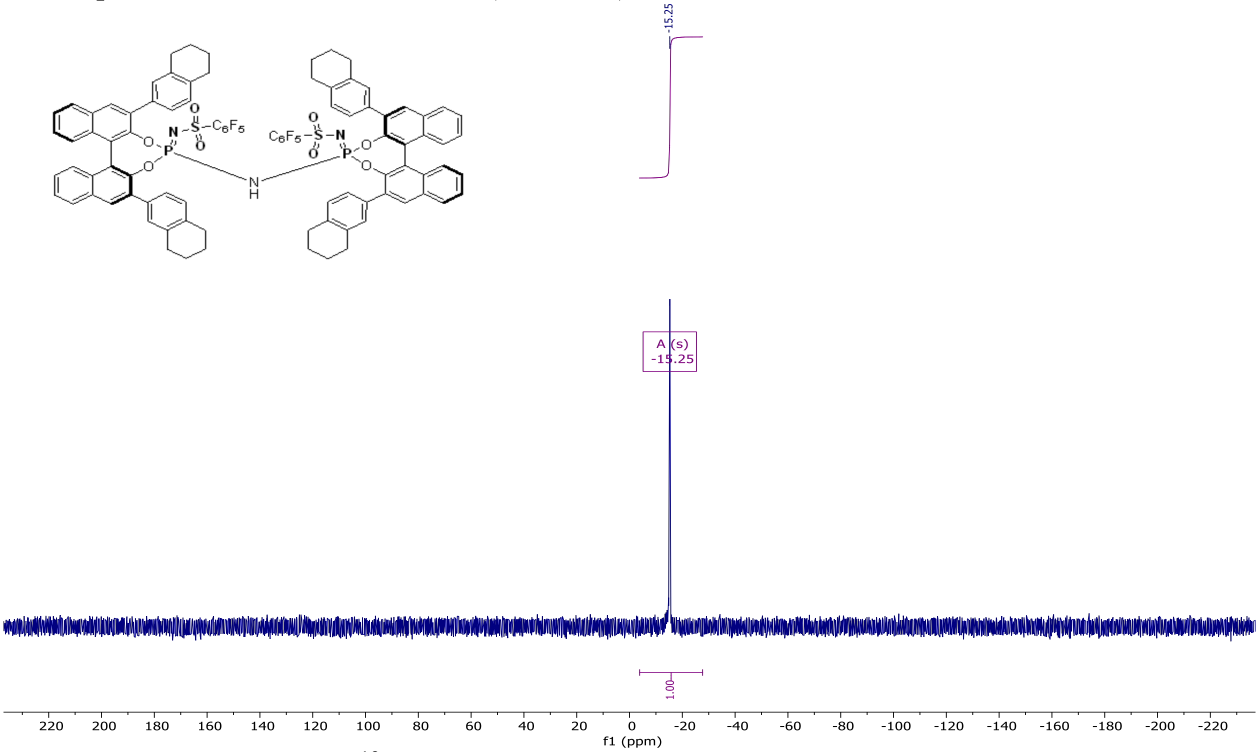


NMR spectra for IDPi-**6h** : $^{19}$F NMR (471 MHz) in $CD_2Cl_2$.

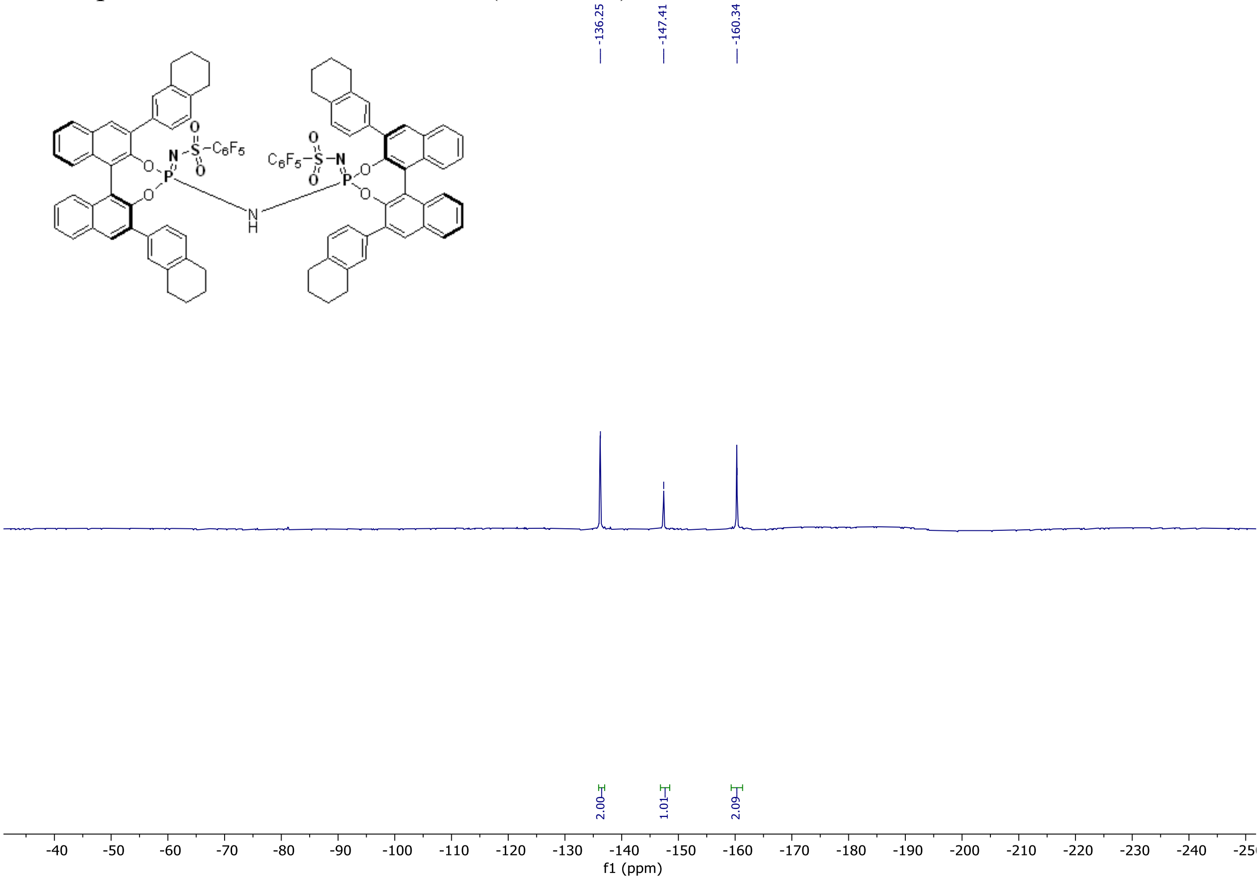

NMR spectra for IDPi-**6h**: $^{1}$H NMR (203 MHz) in $CD_2Cl_2$.

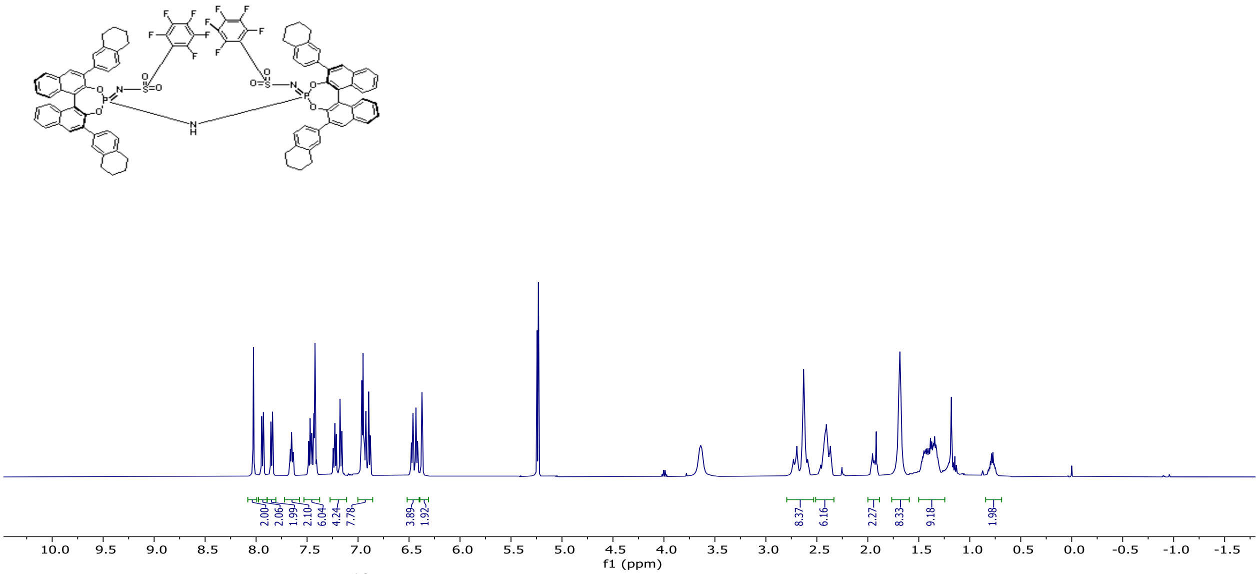


NMR spectra for IDPi-**6h**: $^{13}$C NMR (126 MHz) in $CD_2Cl_2$.

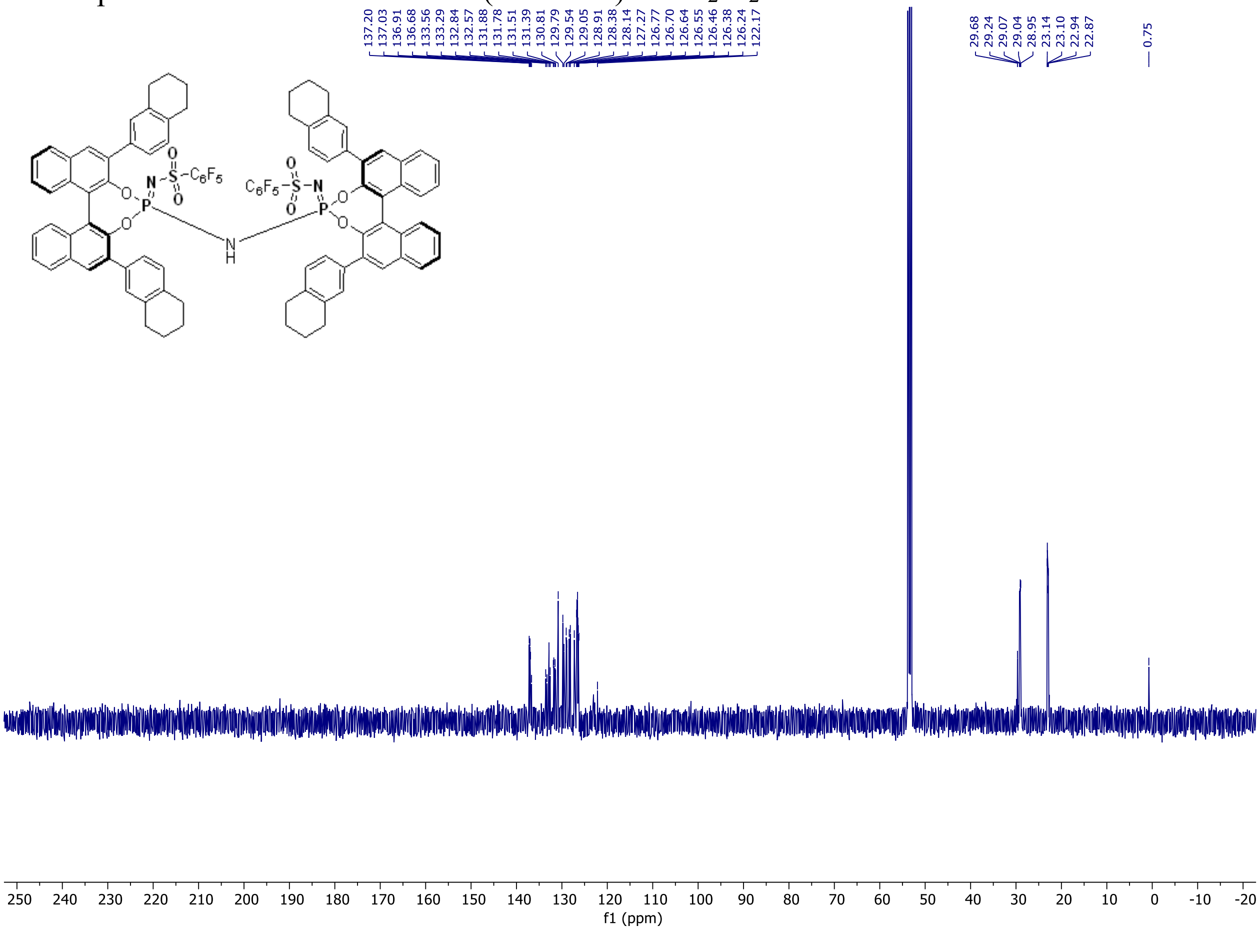

NMR spectra for IDPi-**6i**: $^{31}$P NMR (203 MHz) in $CD_2Cl_2$.

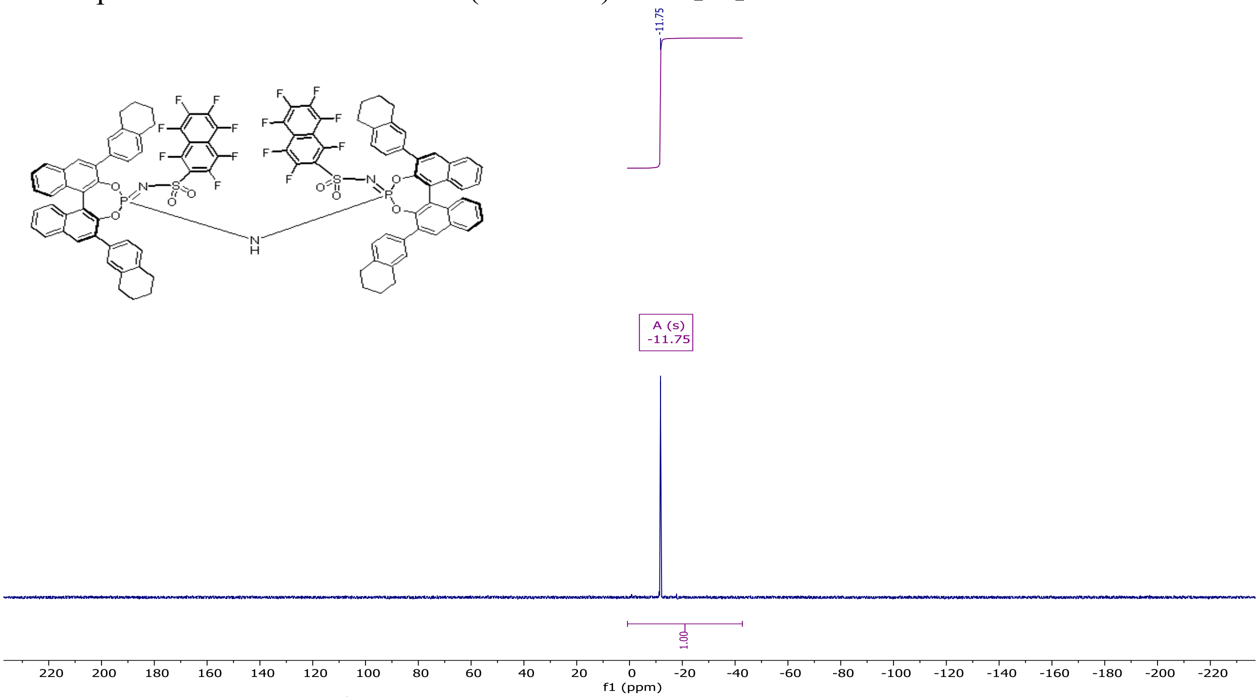


NMR spectra for IDPi-**6i**: $^{1}$H NMR (501 MHz) in $CD_2Cl_2$.

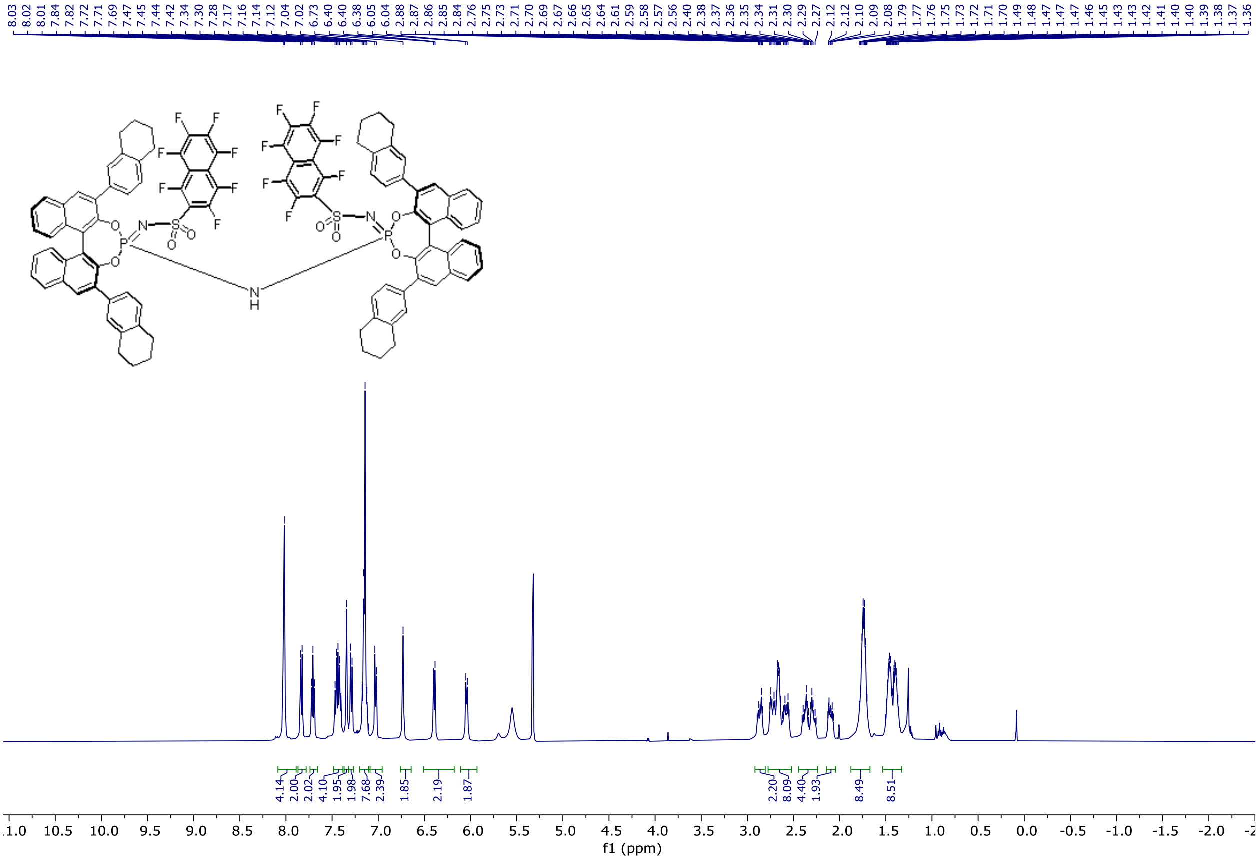

NMR spectra for IDPi-**6i**: $^{19}F$ NMR (471 MHz) in $CD_2Cl_2$.

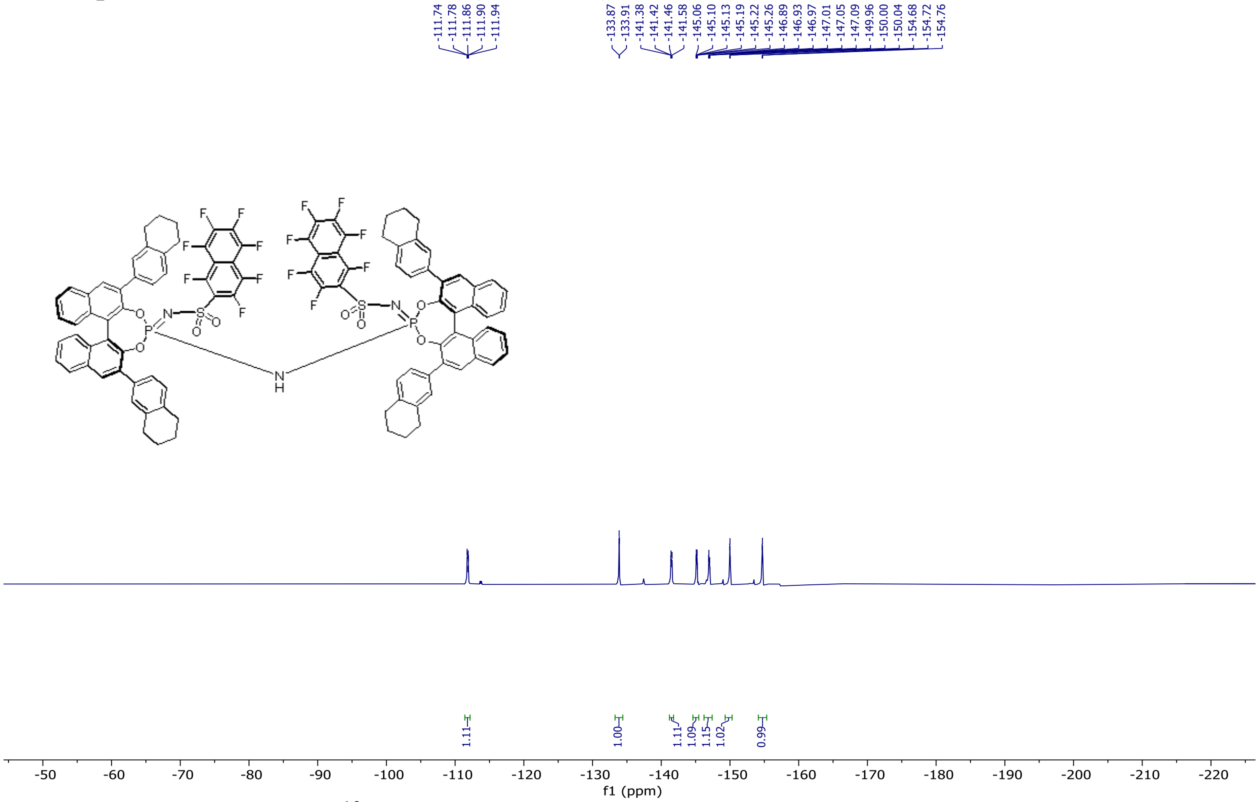


NMR spectra for IDPi-**6i**: $^{13}C$ NMR (126 MHz) in $CD_2Cl_2$.

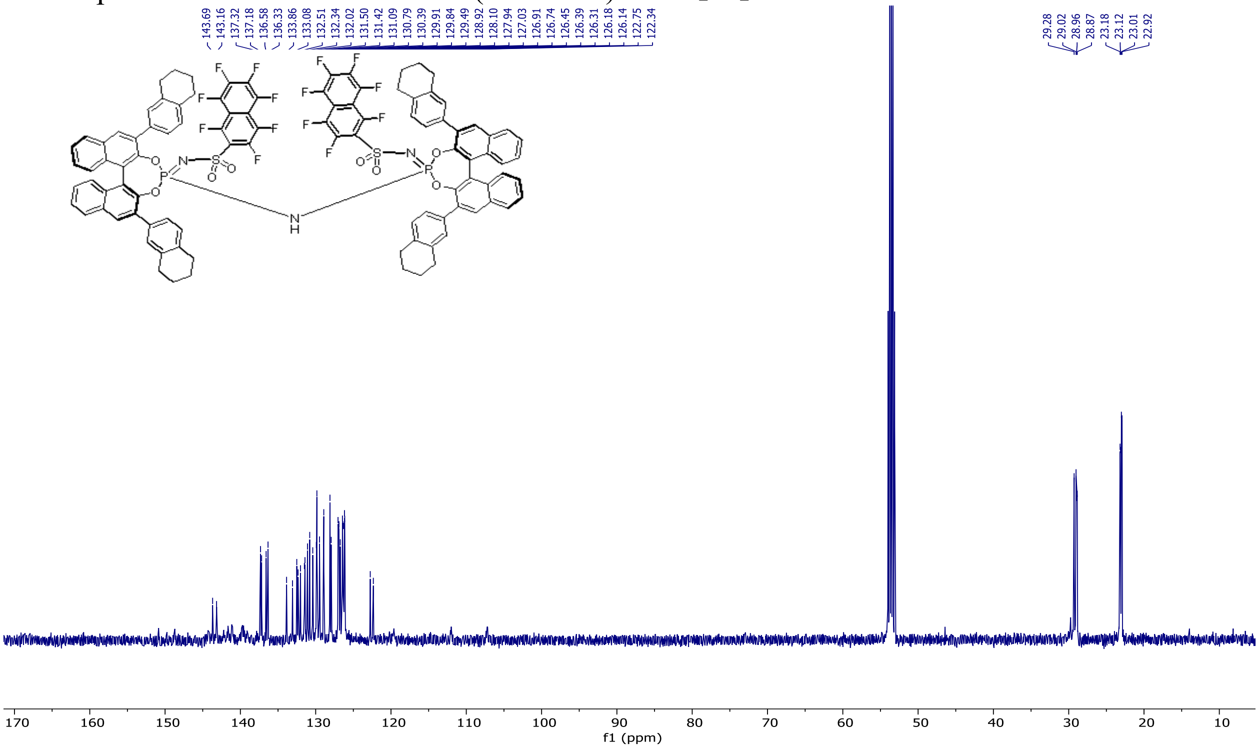

## HPLC Trace

Datafile Name:OUJ-OC-non-sub-RAC.lcd
Sample Name:OUJ-OC-non-sub-RAC
Sample ID:eq4

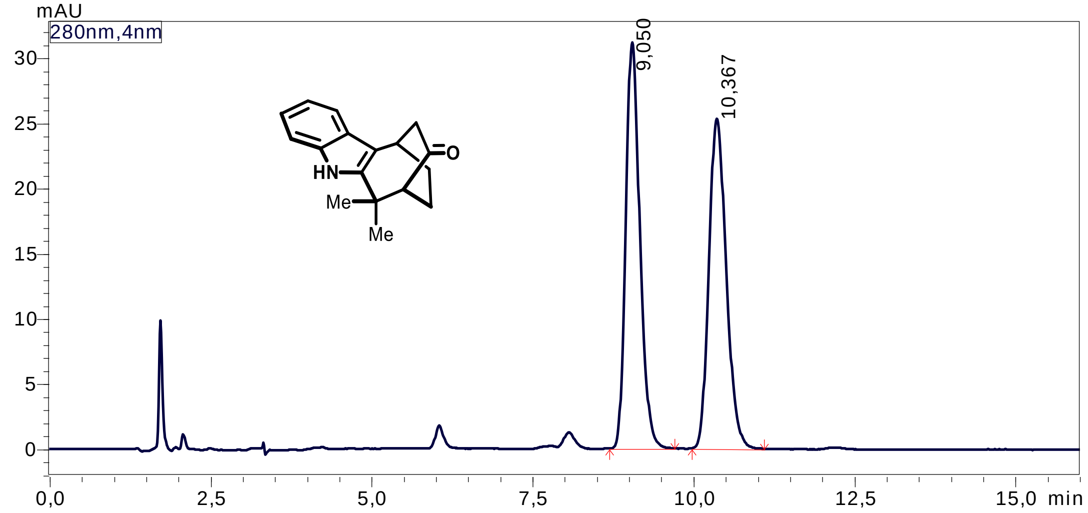


| Peak# | Ret. Time | Area% |
|---|---|---|
| 1 | 9,050 | 49,971 |
| 2 | 10,367 | 50,029 |
| Total | | 100,000 |

Datafile Name:OUJ-OC-122-nonsubst-20 mg-02.lcd
Sample Name:OUJ-OC-122-nonsubst-20 mg-02

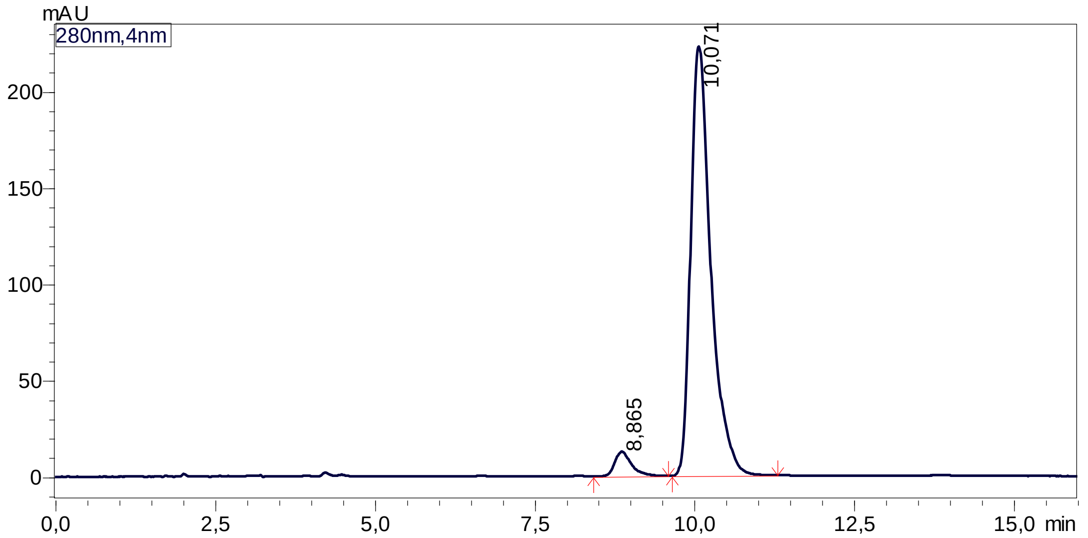


| Peak# | Ret. Time | Area% |
|---|---|---|
| 1 | 8,865 | 4,535 |
| 2 | 10,071 | 95,465 |
| Total | | 100,000 |

Datafile Name:OUJ-OC-5-Me-RAC.lcd
Sample Name:OUJ-OC-5-Me-RAC
Sample ID:eq5

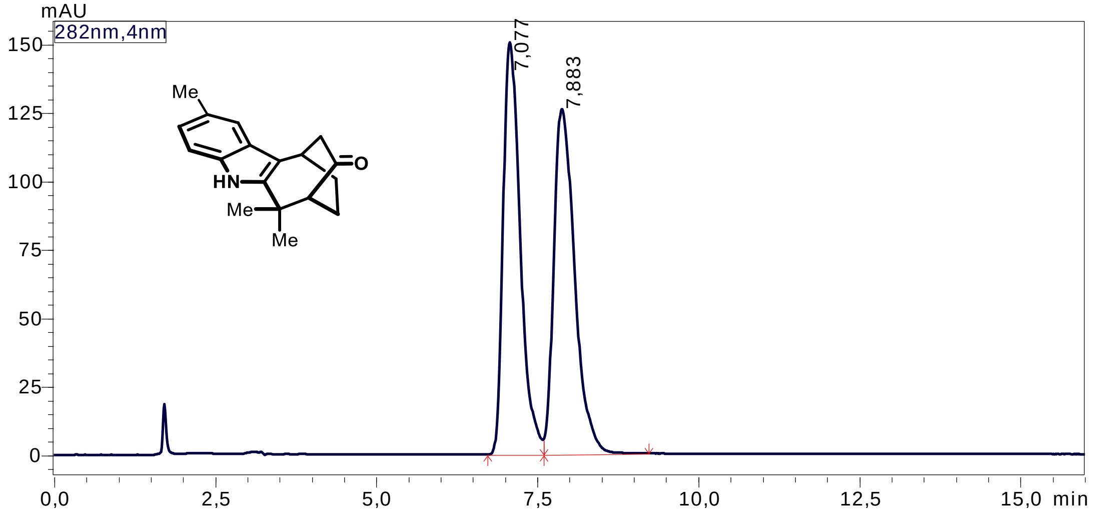


| Peak# | Ret. Time | Area% |
|---|---|---|
| 1 | 7,077 | 49,575 |
| 2 | 7,883 | 50,425 |
| Total | | 100,000 |

Datafile Name:OUJ-OC-011-5-Me-20 mg.lcd
Sample Name:OUJ-OC-011-5-Me-20 mg
Sample ID:eq12

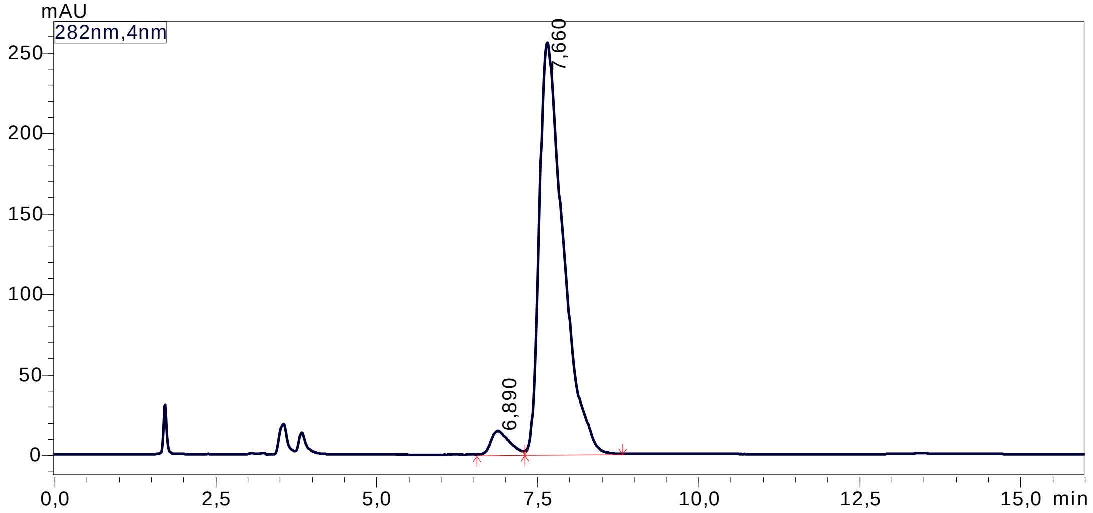


| Peak# | Ret. Time | Area% |
|---|---|---|
| 1 | 6,890 | 4,214 |
| 2 | 7,660 | 95,786 |
| Total | | 100,000 |

Datafile Name:OUJ-OB-779-4-Me-RAC.lcd
Sample Name:OUJ-OB-779-4-Me-RAC

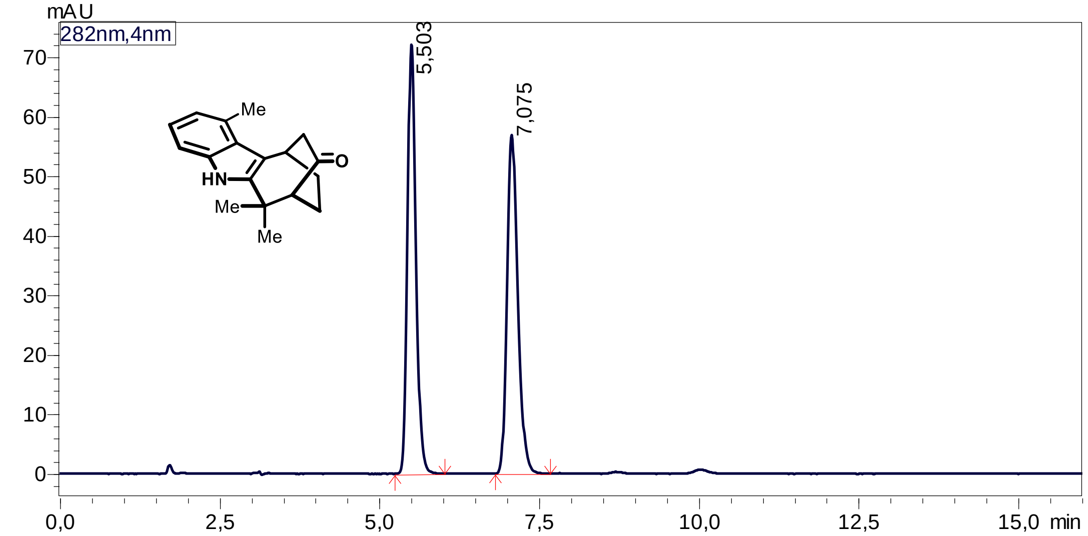


| Peak# | Ret. Time | Area% |
|---|---|---|
| 1 | 5,503 | 49,915 |
| 2 | 7,075 | 50,085 |
| Total | | 100,000 |

Datafile Name:OUJ-OC-072-4-Me-20 mg ee.lcd
Sample Name:OUJ-OC-072-4-Me-20 mg ee
Sample ID:eq007

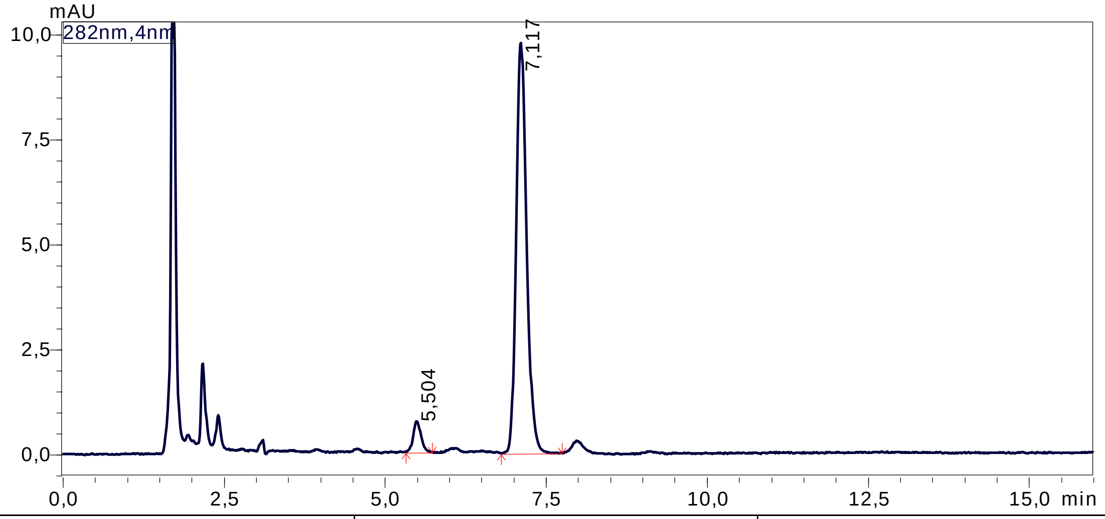


| Peak# | Ret. Time | Area% |
|---|---|---|
| 1 | 5,504 | 5,340 |
| 2 | 7,117 | 94,660 |
| Total | | 100,000 |

Datafile Name:OUJ-OC-6-Me-RAC.lcd
Sample Name:OUJ-OC-6-Me-RAC
Sample ID:eq6

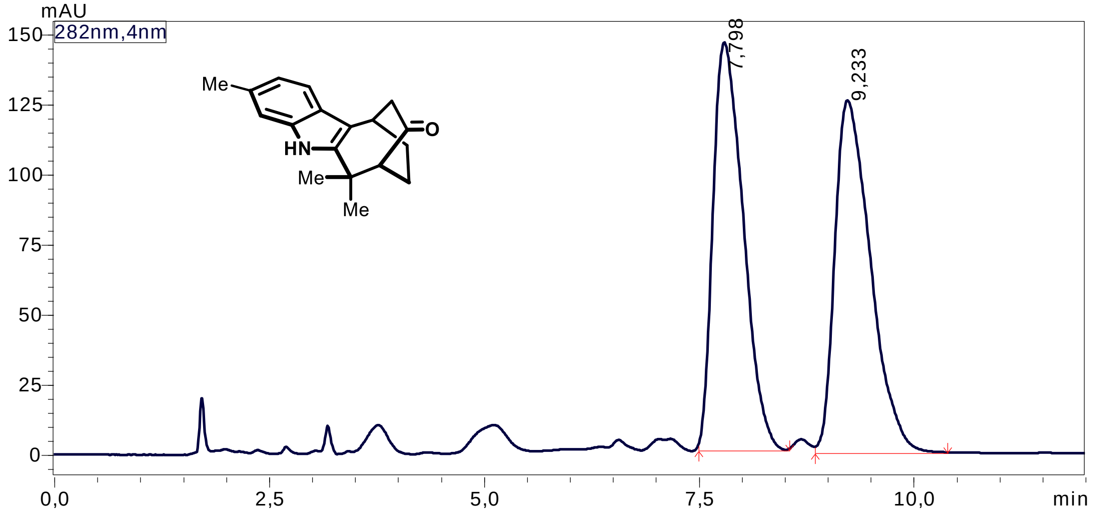


| Peak# | Ret. Time | Area% |
|---|---|---|
| 1 | 7,798 | 49,530 |
| 2 | 9,233 | 50,470 |
| Total | | 100,000 |

Datafile Name:OUJ-OC-012-6-Me-20 mg-001.lcd
Sample Name:OUJ-OC-012-6-Me-20 mg-001

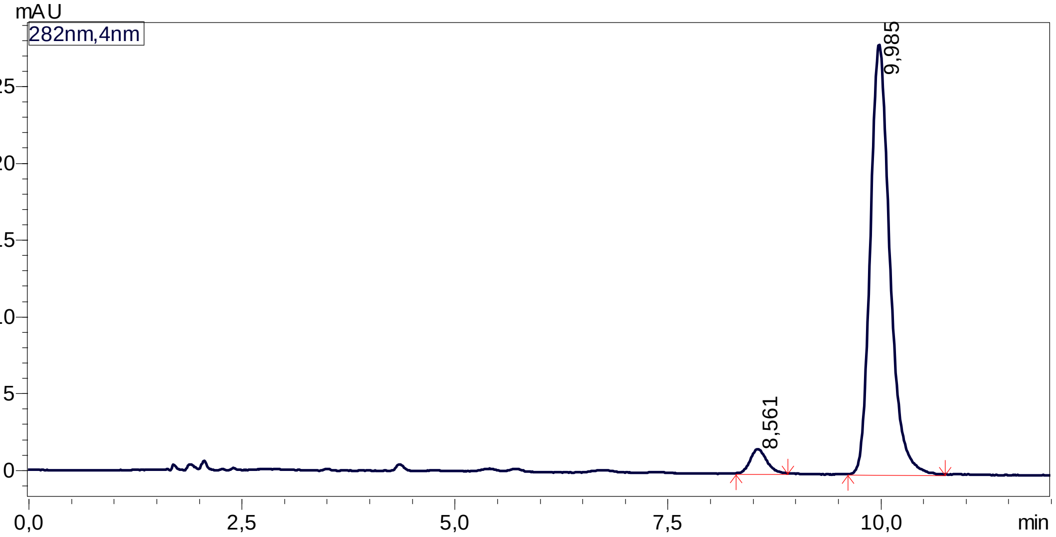


| Peak# | Ret. Time | Area% |
|---|---|---|
| 1 | 8,561 | 4,564 |
| 2 | 9,985 | 95,436 |
| Total | | 100,000 |

Datafile Name:OUJ-OC-7-Me-RAC-02.lcd
Sample Name:OUJ-OC-7-Me-RAC-02
Sample ID:eq7

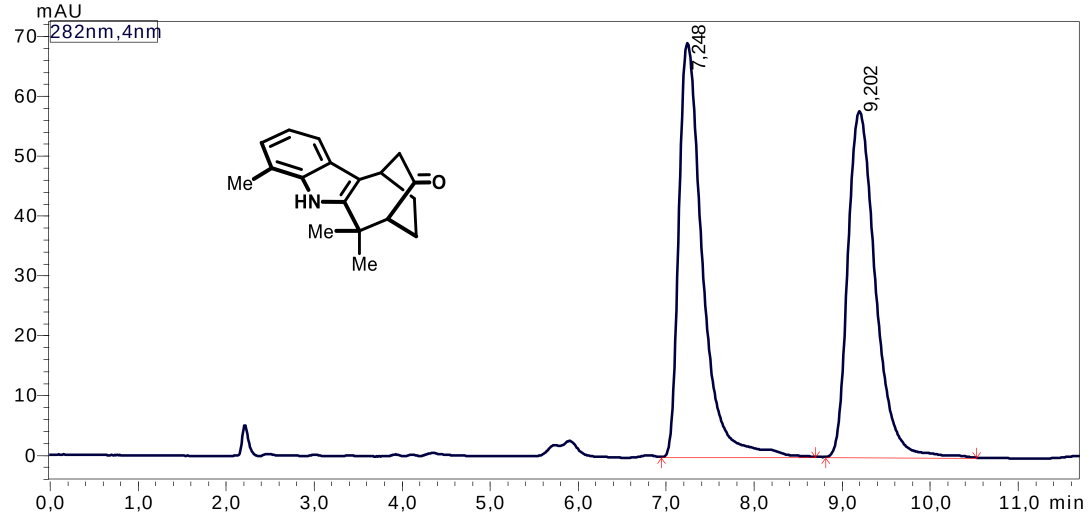


| Peak# | Ret. Time | Area% |
|---|---|---|
| 1 | 7,248 | 50,706 |
| 2 | 9,202 | 49,294 |
| Total | | 100,000 |

Datafile Name:OUJ-OB-986-7-Me-20 mg -03.lcd
Sample Name:OUJ-OB-986-7-Me-20 mg -03

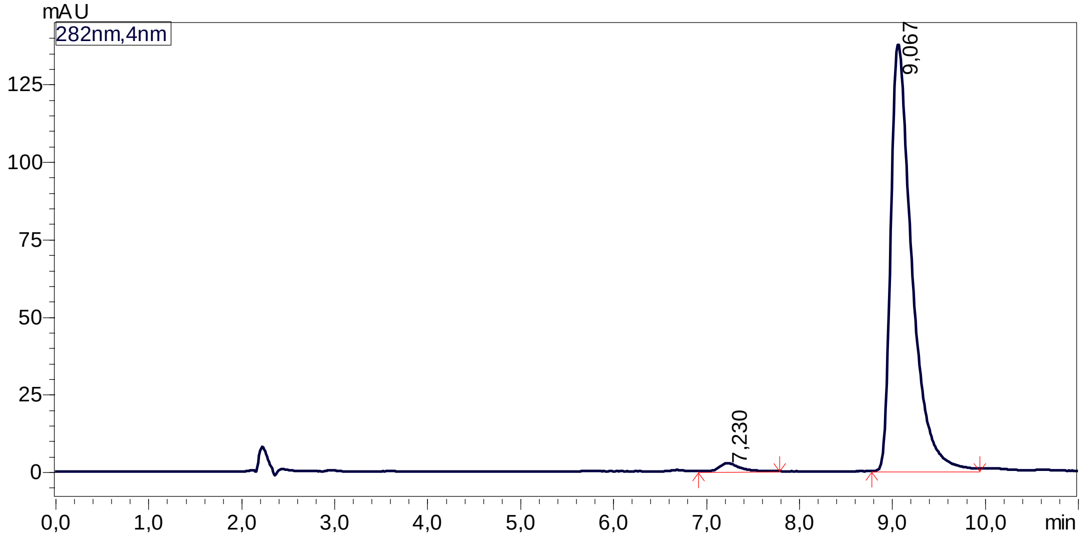


| Peak# | Ret. Time | Area% |
|---|---|---|
| 1 | 7,230 | 1,742 |
| 2 | 9,067 | 98,258 |
| Total | | 100,000 |

Datafile Name:OUJ-OB-cycloPent-RAC.lcd
Sample Name:OUJ-OB-cycloPent-RAC

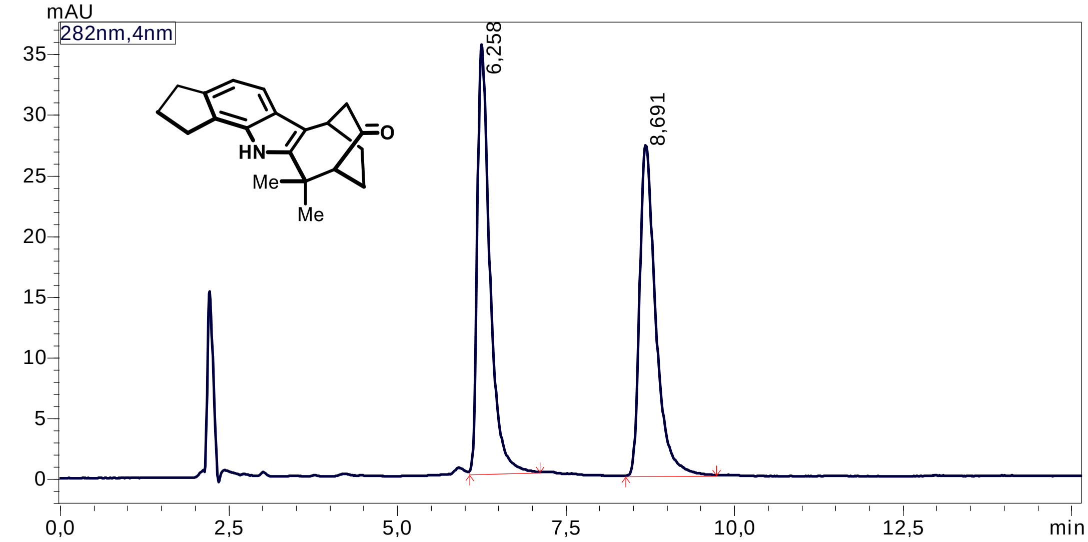


| Peak# | Ret. Time | Area% |
|---|---|---|
| 1 | 6,258 | 50,462 |
| 2 | 8,691 | 49,538 |
| Total | | 100,000 |

Datafile Name:OUJ-OB-913-Cyclopent-C6F5.lcd
Sample Name:OUJ-OB-913-Cyclopent-C6F5

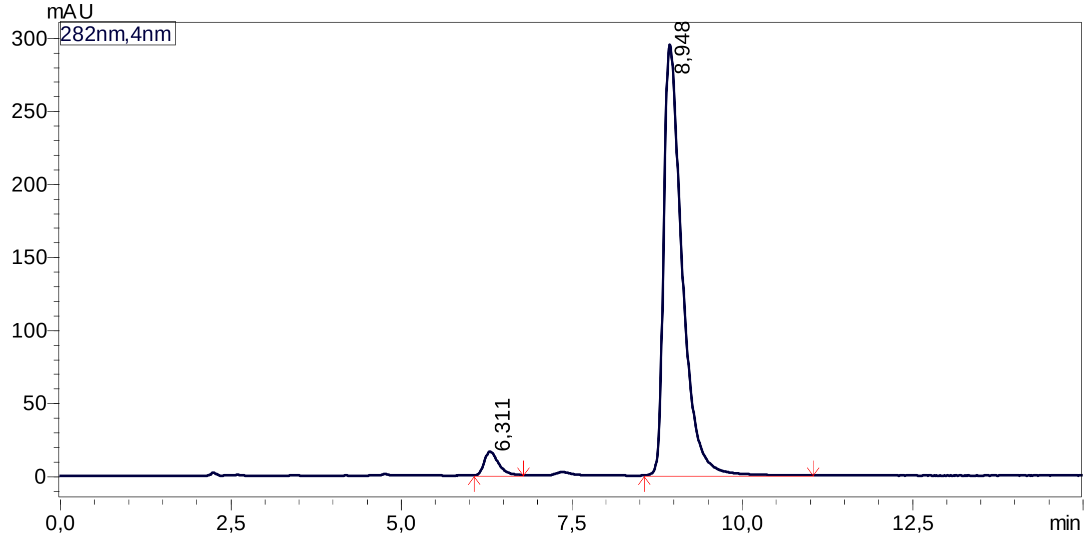


| Peak# | Ret. Time | Area% |
|---|---|---|
| 1 | 6,311 | 4,066 |
| 2 | 8,948 | 95,934 |
| Total | | 100,000 |

Datafile Name:OUJ-OC-093--RAC-67-Ph.lcd
Sample Name:OUJ-OC-093--RAC-67-Ph

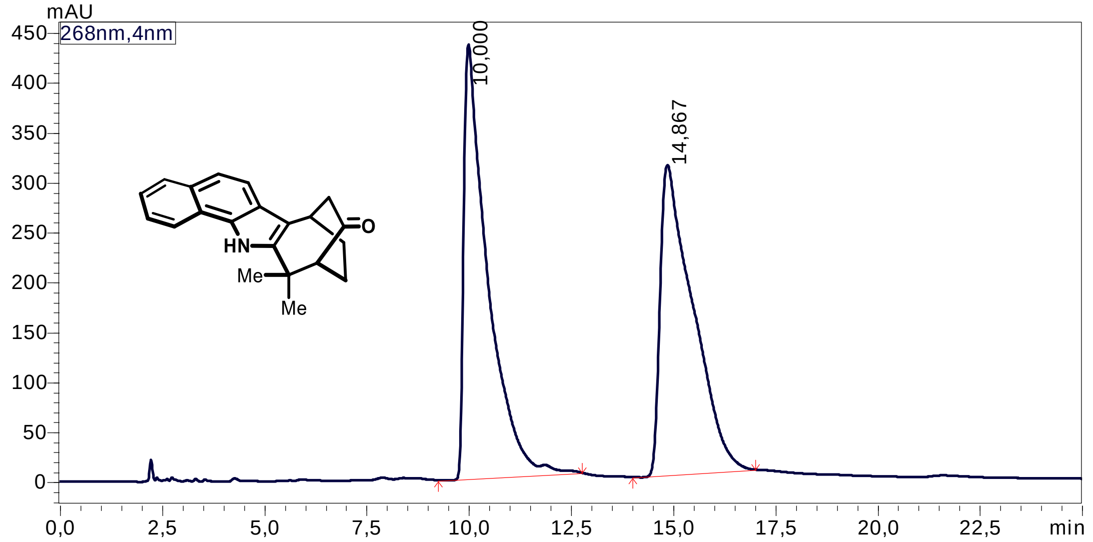


| Peak# | Ret. Time | Area% |
|---|---|---|
| 1 | 10,000 | 50,475 |
| 2 | 14,867 | 49,525 |
| Total | | 100,000 |

Datafile Name:OUJ-OC-0112-6-5-Phenyl-20mg.lcd
Sample Name:OUJ-OC-0112-6-5-Phenyl-20mg

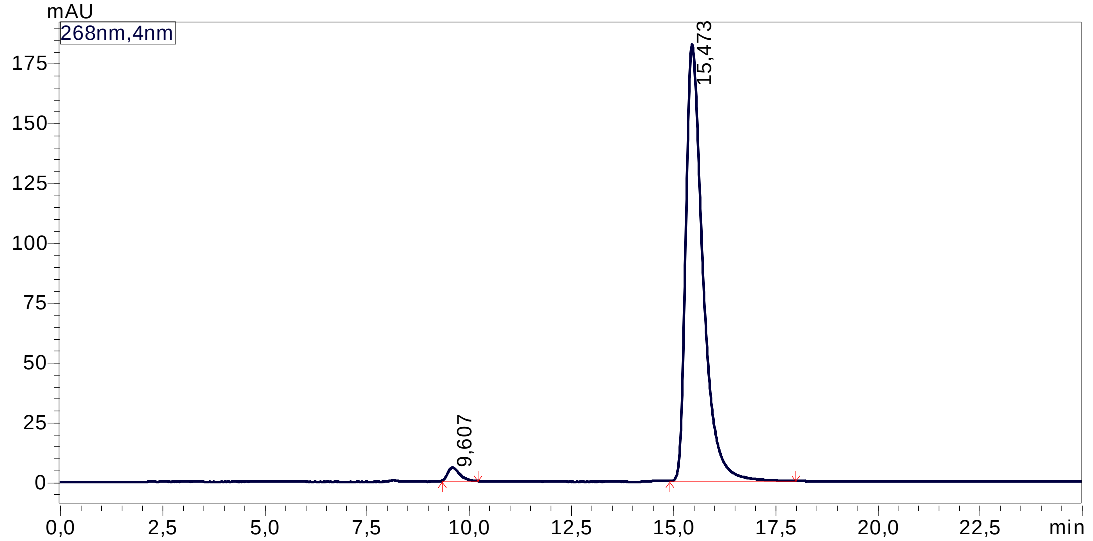


| Peak# | Ret. Time | Area% |
|---|---|---|
| 1 | 9,607 | 2,113 |
| 2 | 15,473 | 97,887 |
| Total | | 100,000 |

Datafile Name:OUJ-OC-6-F-RAC.lcd
Sample Name:OUJ-OC-6-F-RAC
Sample ID:eq9

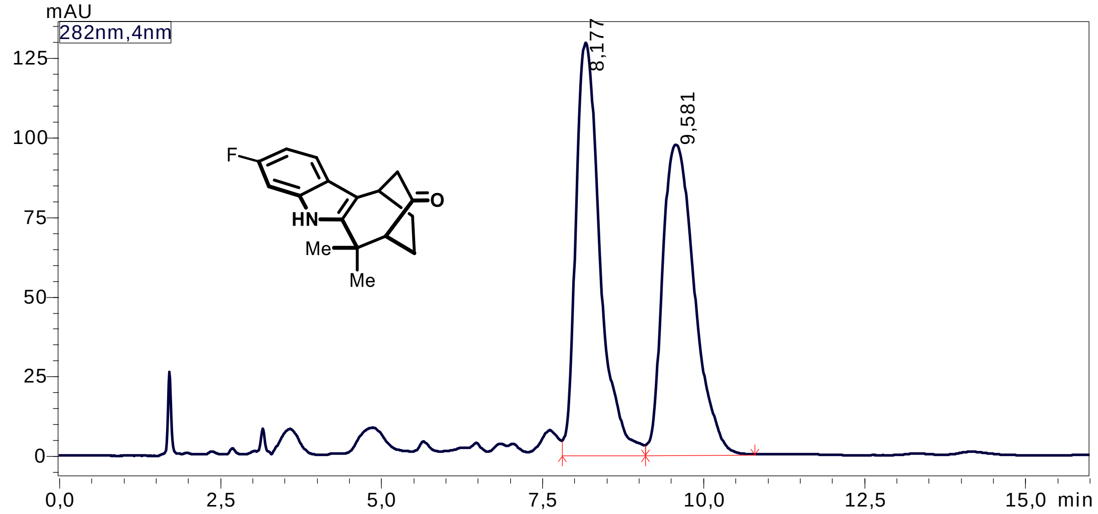


| Peak# | Ret. Time | Area% |
|---|---|---|
| 1 | 8,177 | 50,276 |
| 2 | 9,581 | 49,724 |
| Total | | 100,000 |

Datafile Name:OUJ-OC-013-6-F-20 mg.lcd
Sample Name:OUJ-OC-013-6-F-20 mg

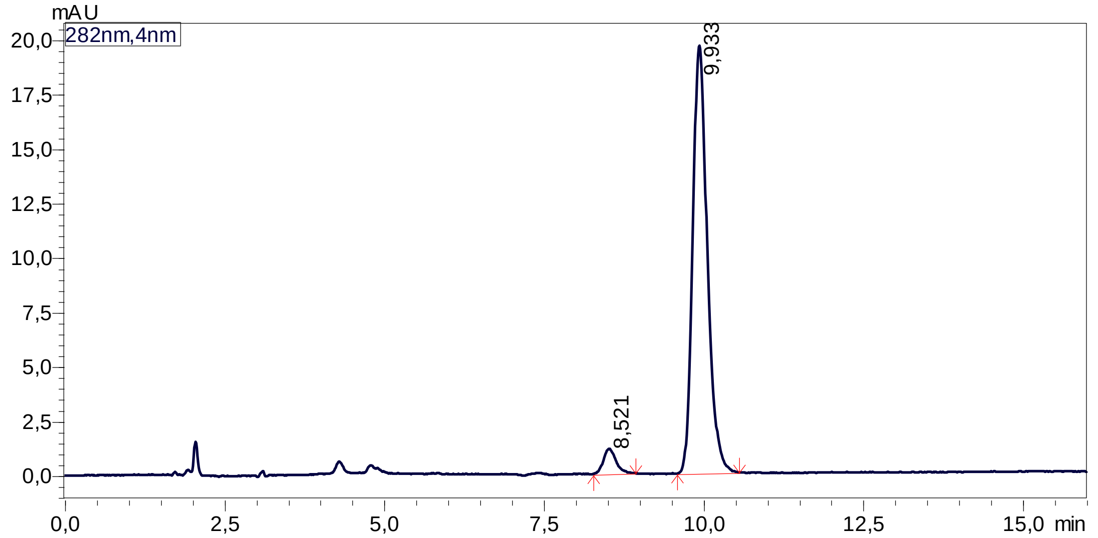


| Peak# | Ret. Time | Area% |
|---|---|---|
| 1 | 8,521 | 4,806 |
| 2 | 9,933 | 95,194 |
| Total | | 100,000 |

Datafile Name:OUJ-OC-6-Cl-RAC.lcd
Sample Name:OUJ-OC-6-Cl-RAC
Sample ID:eq10

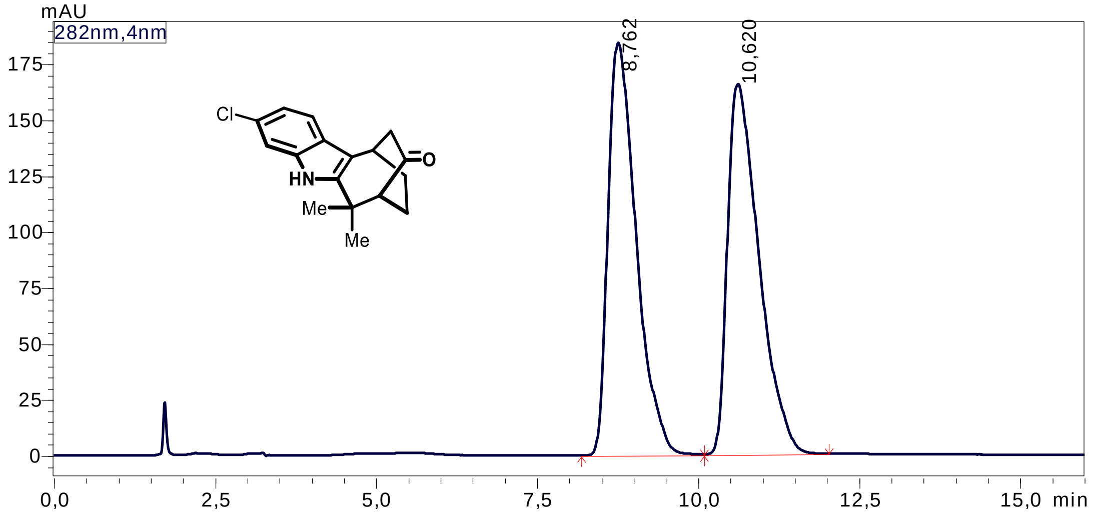


| Peak# | Ret. Time | Area% |
|---|---|---|
| 1 | 8,762 | 49,988 |
| 2 | 10,620 | 50,012 |
| Total | | 100,000 |

Datafile Name:OUJ-OC-097-6-Cl-20 mg001.lcd
Sample Name:OUJ-OC-097-6-Cl-20 mg001

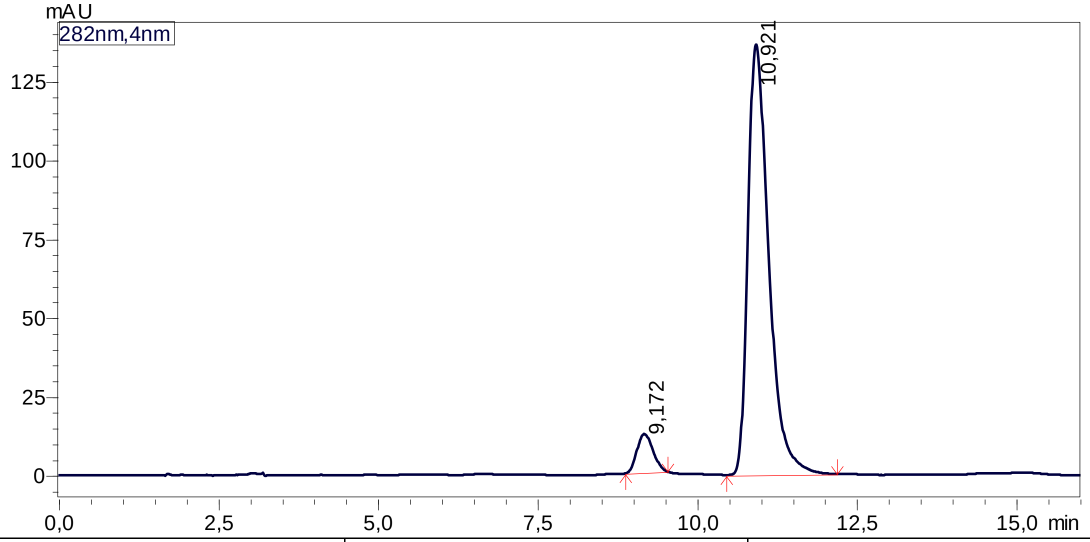


| Peak# | Ret. Time | Area% |
|---|---|---|
| 1 | 9,172 | 6,482 |
| 2 | 10,921 | 93,518 |
| Total | | 100,000 |

Datafile Name:OUJ-OB-805-6-Br-RAC.lcd
Sample Name:OUJ-OB-805-6-Br-RAC

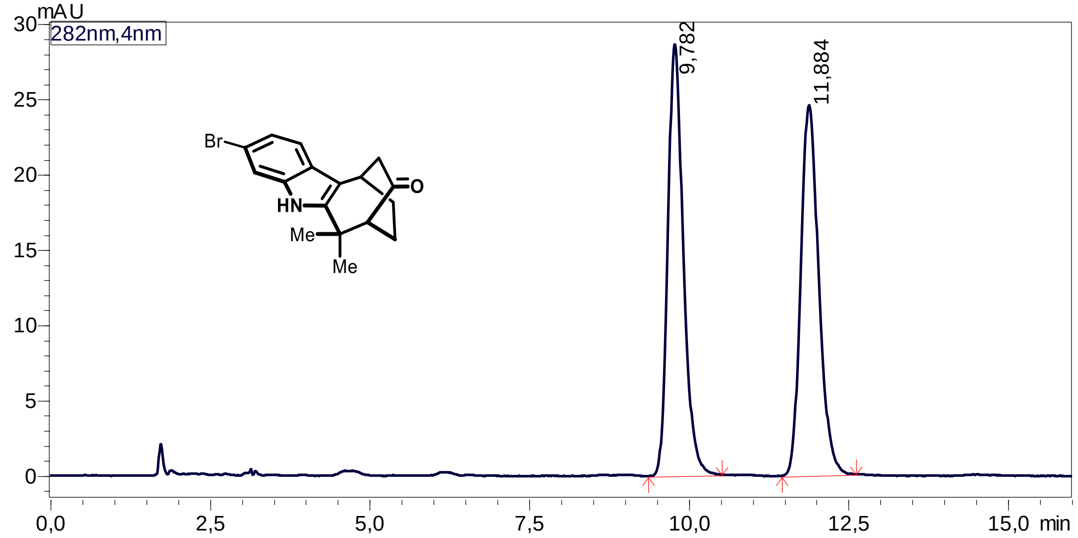


| Peak# | Ret. Time | Area% |
|---|---|---|
| 1 | 9,782 | 50,022 |
| 2 | 11,884 | 49,978 |
| Total | | 100,000 |

Datafile Name:OUJ-OC-103-6-Br-20 mg002.lcd
Sample Name:OUJ-OC-103-6-Br-20 mg002

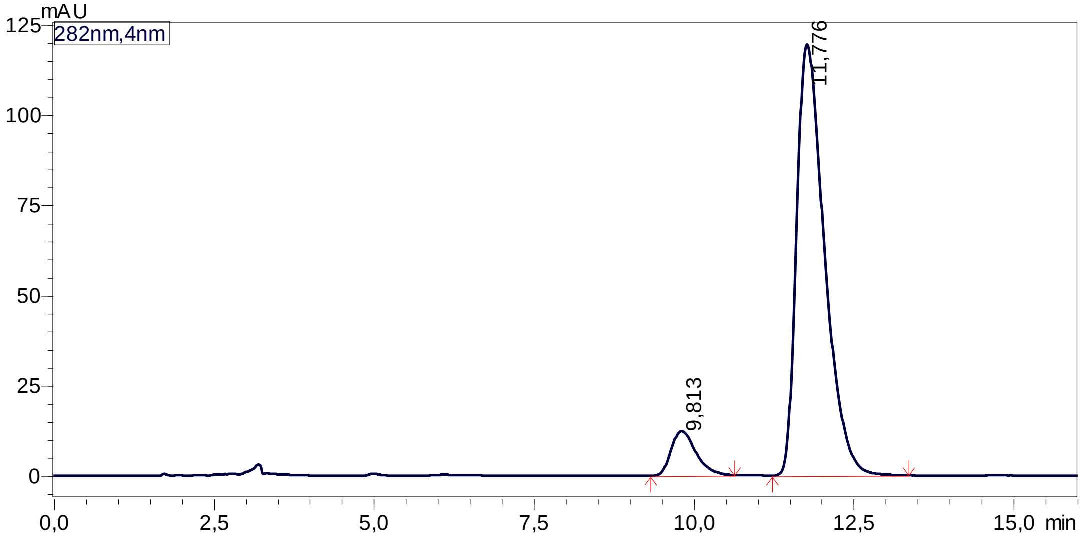


| Peak# | Ret. Time | Area% |
|---|---|---|
| 1 | 9,813 | 8,371 |
| 2 | 11,776 | 91,629 |
| Total | | 100,000 |

Datafile Name:OUJ-OB-6-MeO-RAC.lcd
Sample Name:OUJ-OB-6-MeO-RAC

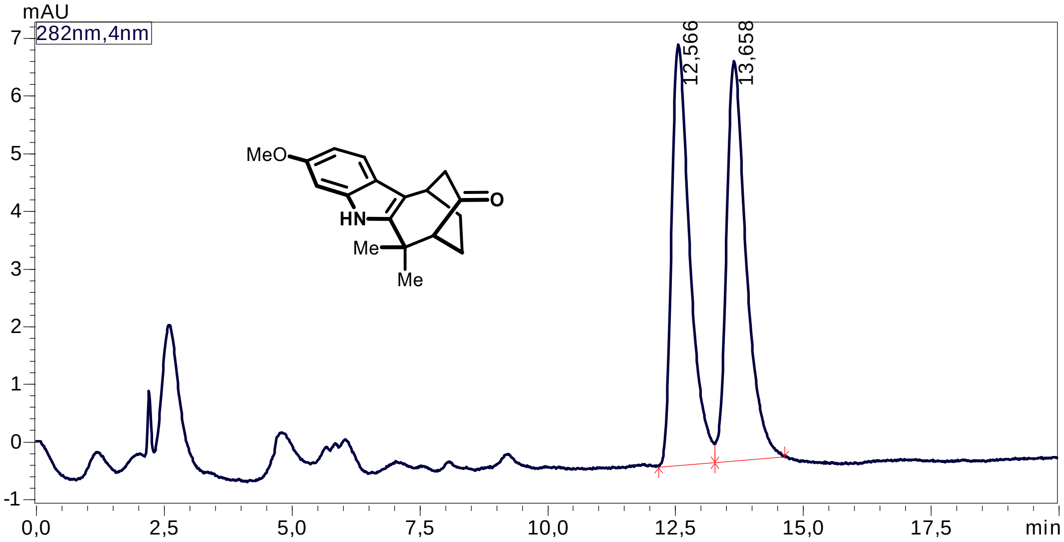


| Peak# | Ret. Time | Area% |
|---|---|---|
| 1 | 12,566 | 49,285 |
| 2 | 13,658 | 50,715 |
| Total | | 100,000 |

Datafile Name:OUJ-OC-073-6MeO-20 mg.lcd
Sample Name:OUJ-OC-073-6MeO-20 mg

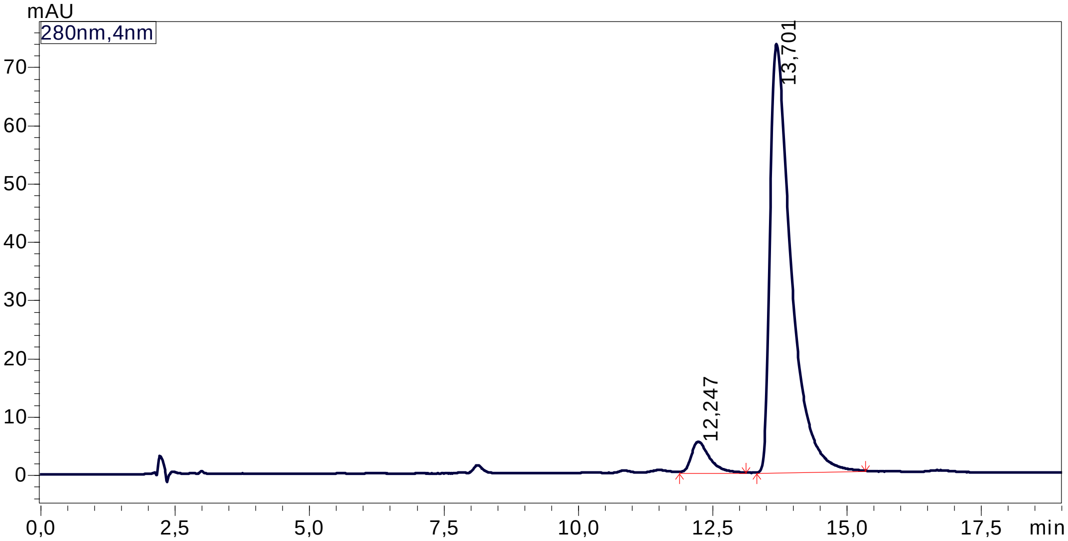


| Peak# | Ret. Time | Area% |
|---|---|---|
| 1 | 12,247 | 5,867 |
| 2 | 13,701 | 94,133 |
| Total | | 100,000 |

Datafile Name:OUJ-OC-128-nBu-RAC-P2.lcd
Sample Name:OUJ-OC-128-nBu-RAC-P2

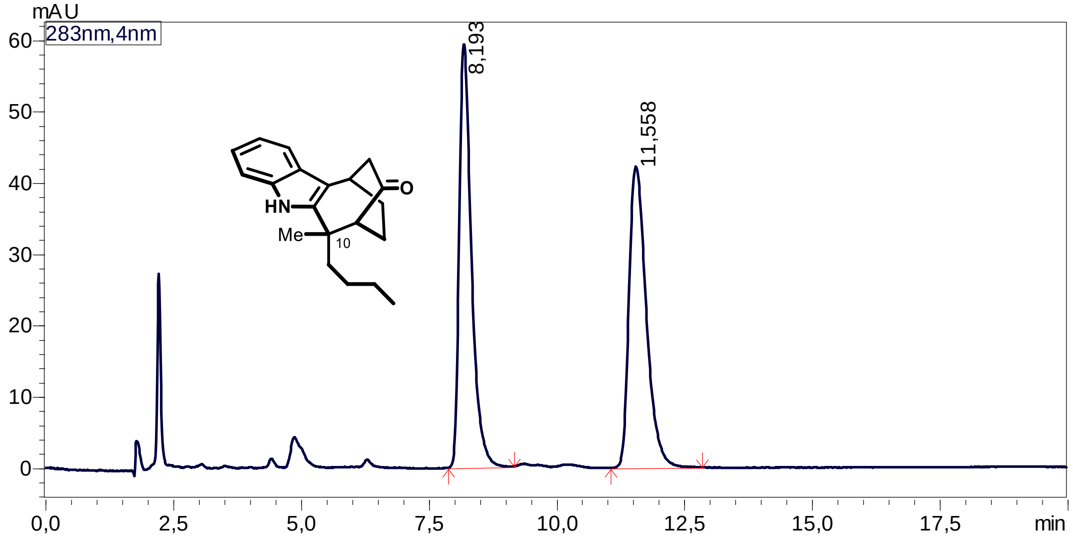


| Peak# | Ret. Time | Area% |
|---|---|---|
| 1 | 8,193 | 49,873 |
| 2 | 11,558 | 50,127 |
| Total | | 100,000 |

Datafile Name:OUJ-OB-130-n-Bu-P2-ee.lcd
Sample Name:OUJ-OB-130-n-Bu-P2-ee

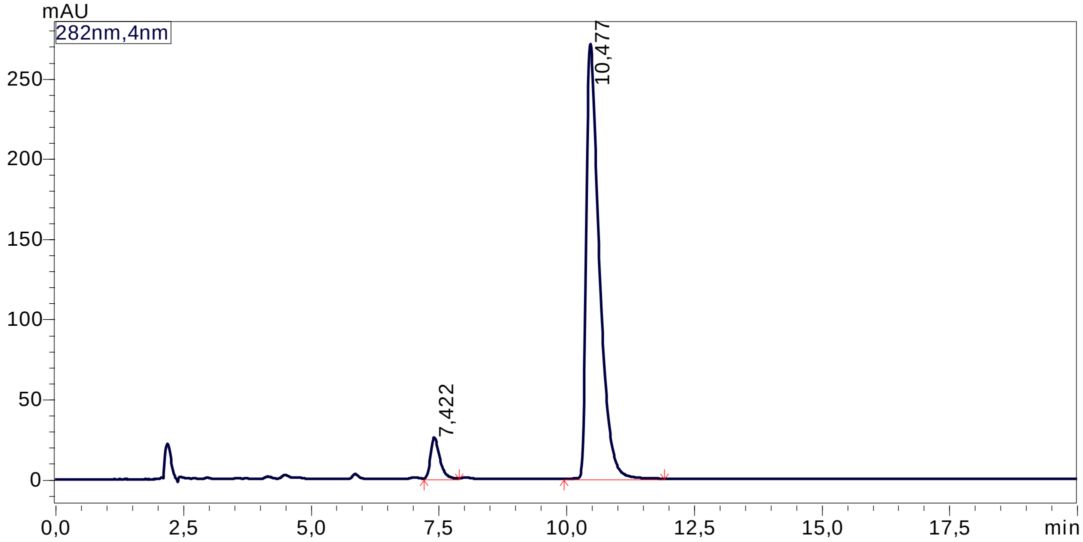


| Peak# | Ret. Time | Area% |
|---|---|---|
| 1 | 7,422 | 6,420 |
| 2 | 10,477 | 93,580 |
| Total | | 100,000 |

Datafile Name:OUJ-OC-439-P1-Cis-RAC.lcd
Sample Name:OUJ-OC-439-P1-Cis-RAC

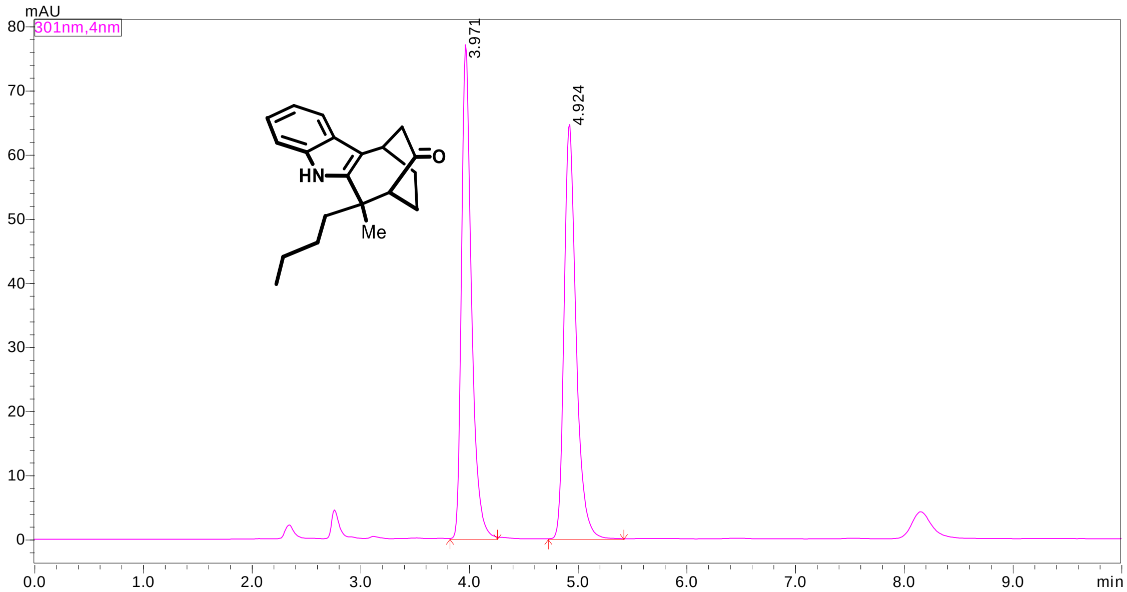


| Peak# | Ret. Time | Area% |
|---|---|---|
| 1 | 3.971 | 49.956 |
| 2 | 4.924 | 50.044 |
| Total | | 100.000 |

Datafile Name:OUJ-OC-439-P1-Cis-ee.lcd
Sample Name:OUJ-OC-439-P1-Cis-ee

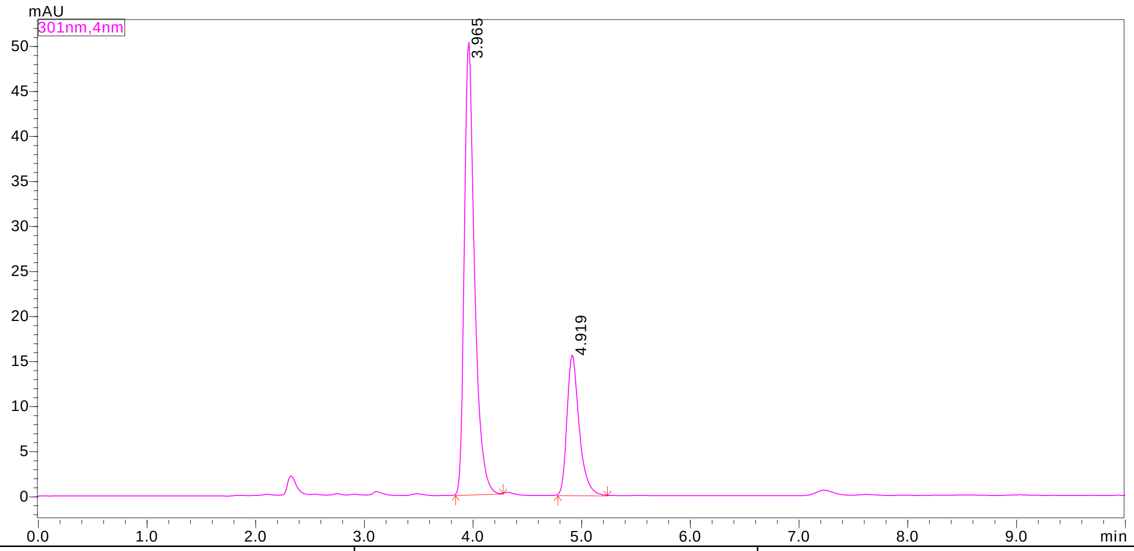


| Peak# | Ret. Time | Area% |
|---|---|---|
| 1 | 3.965 | 73.138 |
| 2 | 4.919 | 26.862 |
| Total | | 100.000 |

Datafile Name:snpD33F.tmp
Sample Name:OUJ-OC-121-iso-Bu-RAC-05

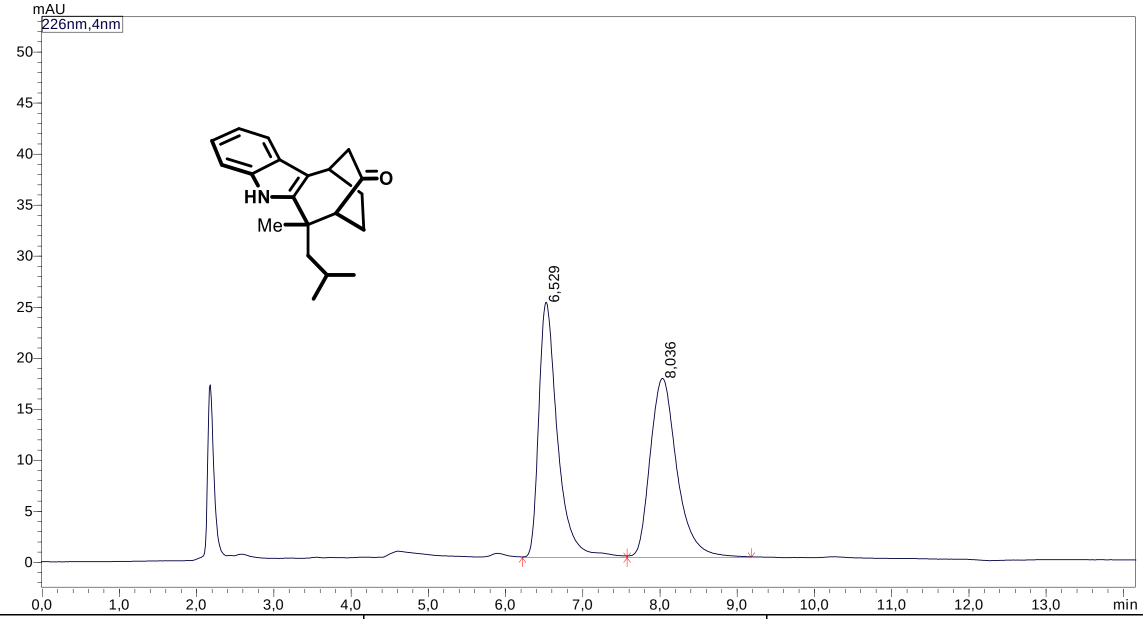


| Peak# | Ret. Time | Area% |
|---|---|---|
| 1 | 6,529 | 50,027 |
| 2 | 8,036 | 49,973 |
| Total | | 100,000 |

Datafile Name:OUJ-OC-121-iso-Bu-ee.lcd
Sample Name:OUJ-OC-121-iso-Bu-ee

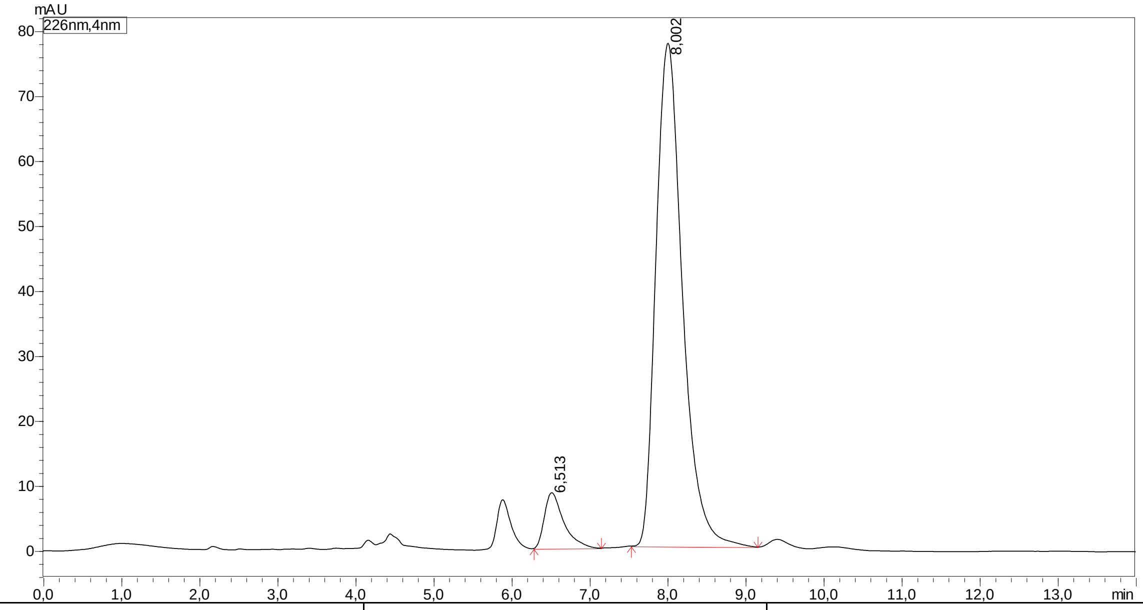


| Peak# | Ret. Time | Area% |
|---|---|---|
| 1 | 6,513 | 7,513 |
| 2 | 8,002 | 92,487 |
| Total | | 100,000 |

Datafile Name:OUJ-OC-121-iso-But-P1-RAC.lcd
Sample Name:OUJ-OC-121-iso-But-P1-RAC
Sample ID:eq010

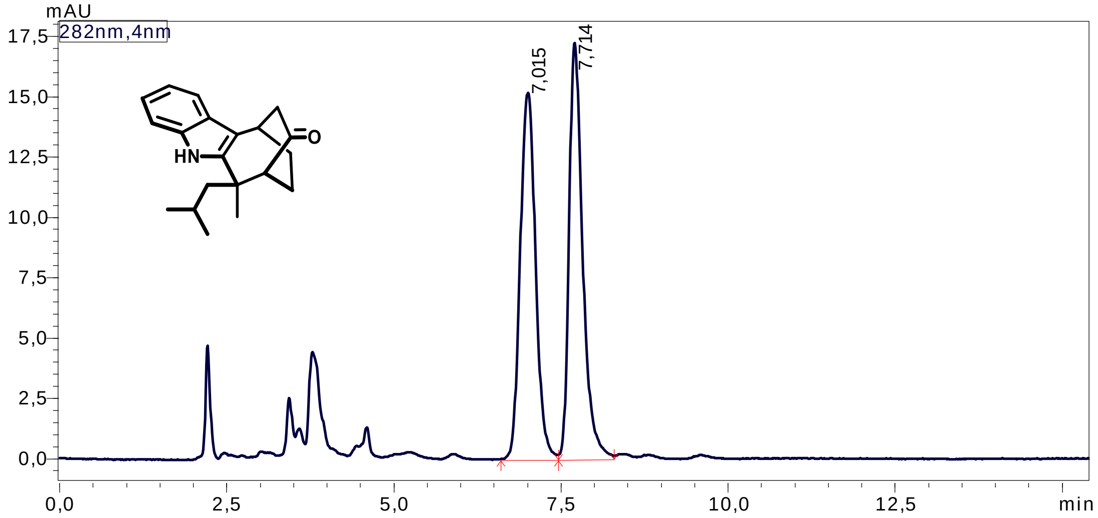


| Peak# | Ret. Time | Area% |
|---|---|---|
| 1 | 7,015 | 49,886 |
| 2 | 7,714 | 50,114 |
| Total | | 100,000 |

Datafile Name:OUJ-OB-121-P1-isoBut.lcd
Sample Name:OUJ-OB-121-P1-isoBut

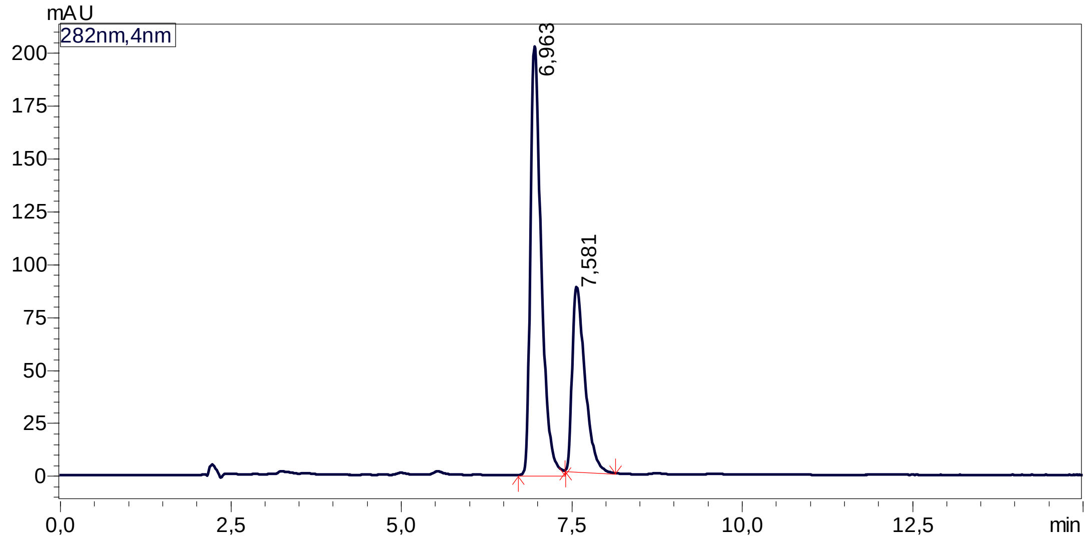


| Peak# | Ret. Time | Area% |
|---|---|---|
| 1 | 6,963 | 66,702 |
| 2 | 7,581 | 33,298 |
| Total | | 100,000 |

Datafile Name:OUJ-OC-117-n-Butene-P2-RAC001.lcd
Sample Name:OUJ-OC-117-n-Butene-P2-RAC001
Sample ID:eq009

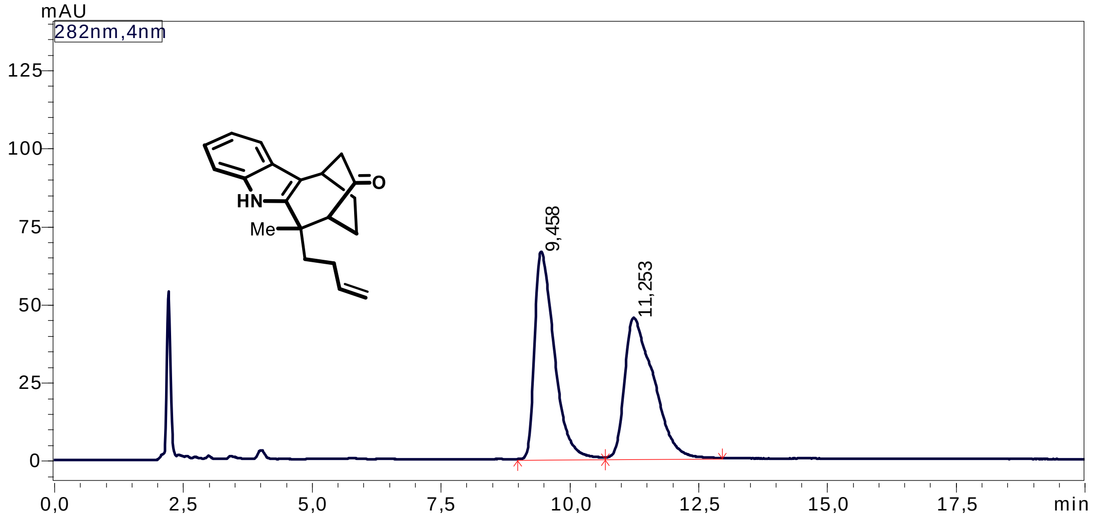


| Peak# | Ret. Time | Area% |
|---|---|---|
| 1 | 9,458 | 49,907 |
| 2 | 11,253 | 50,093 |
| Total | | 100,000 |

Datafile Name:OUJ-OC-117-P2-n-Butene.lcd
Sample Name:OUJ-OC-117-P2-n-Butene

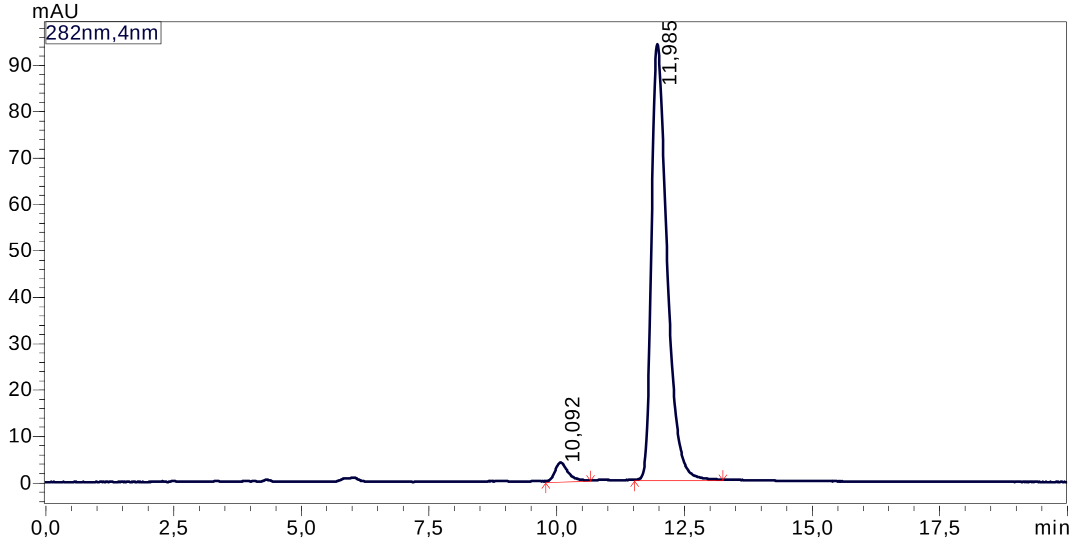


| Peak# | Ret. Time | Area% |
|---|---|---|
| 1 | 10,092 | 3,281 |
| 2 | 11,985 | 96,719 |
| Total | | 100,000 |

Datafile Name:OUJ-OC-117-n-Butene-P1-RAC.lcd
Sample Name:OUJ-OC-117-n-Butene-P1-RAC
Sample ID:eq008

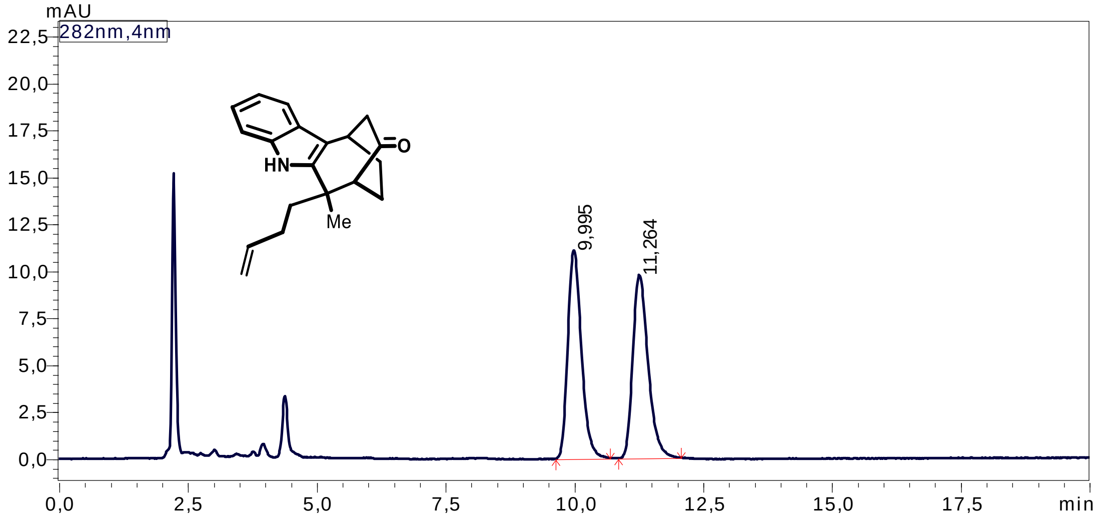


| Peak# | Ret. Time | Area% |
|---|---|---|
| 1 | 9,995 | 50,244 |
| 2 | 11,264 | 49,756 |
| Total | | 100,000 |

Datafile Name:OUJ-OC-117-P1.lcd
Sample Name:OUJ-OC-117-P1
Sample ID:eq004

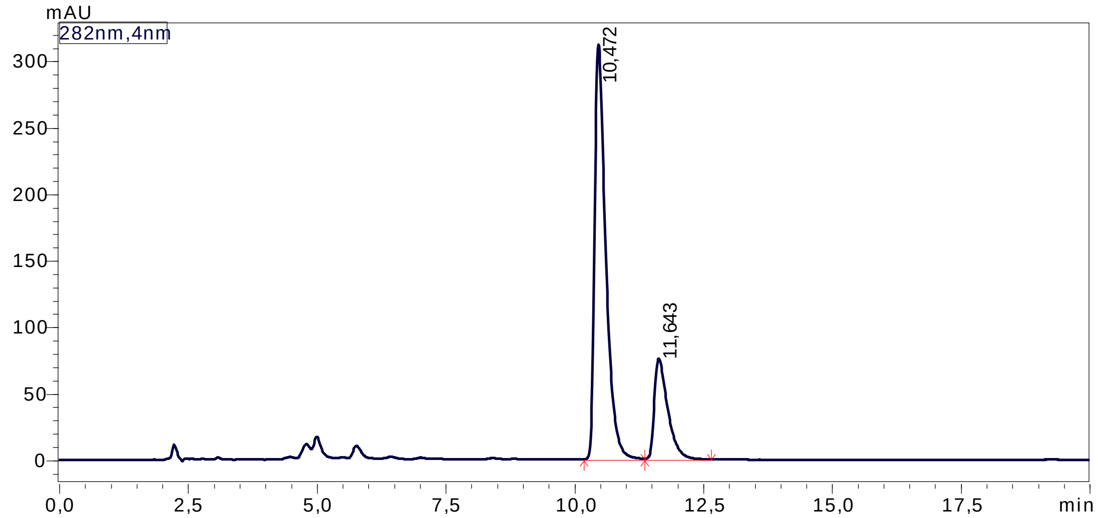


| Peak# | Ret. Time | Area% |
|---|---|---|
| 1 | 10,472 | 77,459 |
| 2 | 11,643 | 22,541 |
| Total | | 100,000 |

Datafile Name:OUJ-OC-089-P2-tBu-P2-RAC.lcd
Sample Name:OUJ-OC-089-P2-tBu-P2-RAC

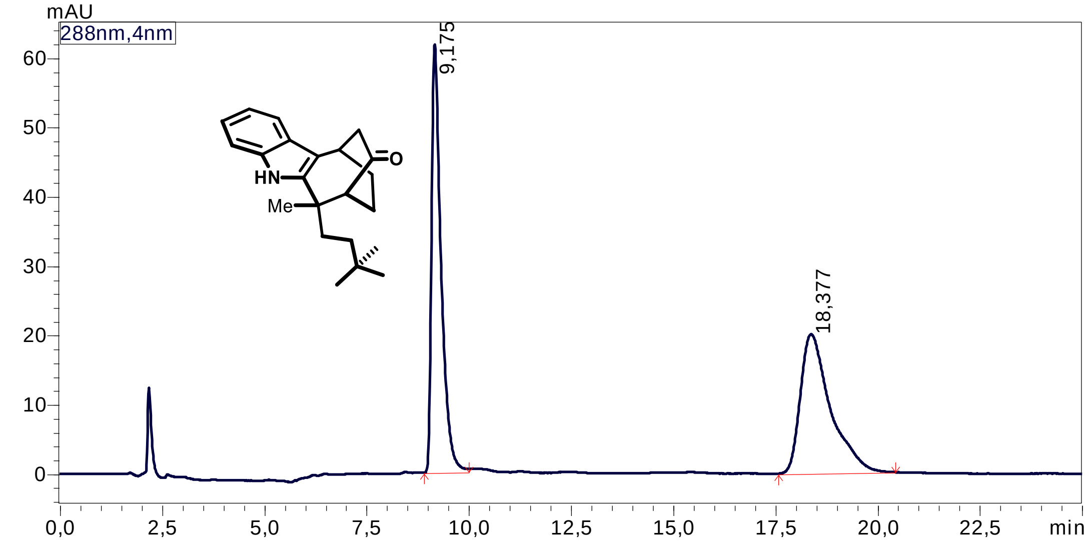


| Peak# | Ret. Time | Area% |
|---|---|---|
| 1 | 9,175 | 49,506 |
| 2 | 18,377 | 50,494 |
| Total | | 100,000 |

Datafile Name:OUJ-OC-079-P2-tBu-05.lcd
Sample Name:OUJ-OC-079-P2-tBu-05

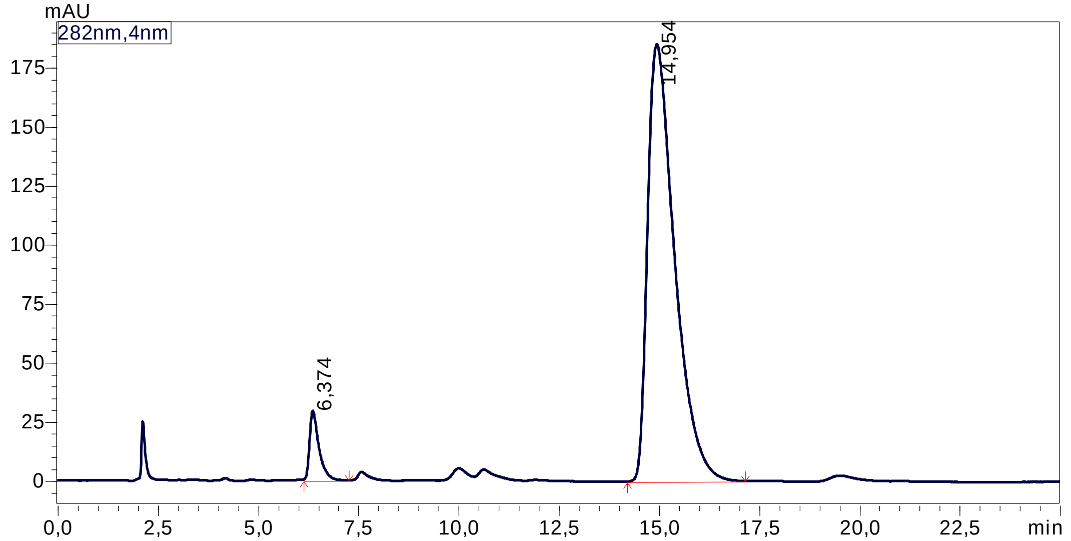


| Peak# | Ret. Time | Area% |
|---|---|---|
| 1 | 6,374 | 5,304 |
| 2 | 14,954 | 94,696 |
| Total | | 100,000 |

Datafile Name:OUJ-OC-089-P1-RAC-01.lcd
Sample Name:OUJ-OC-089-P1-RAC-01

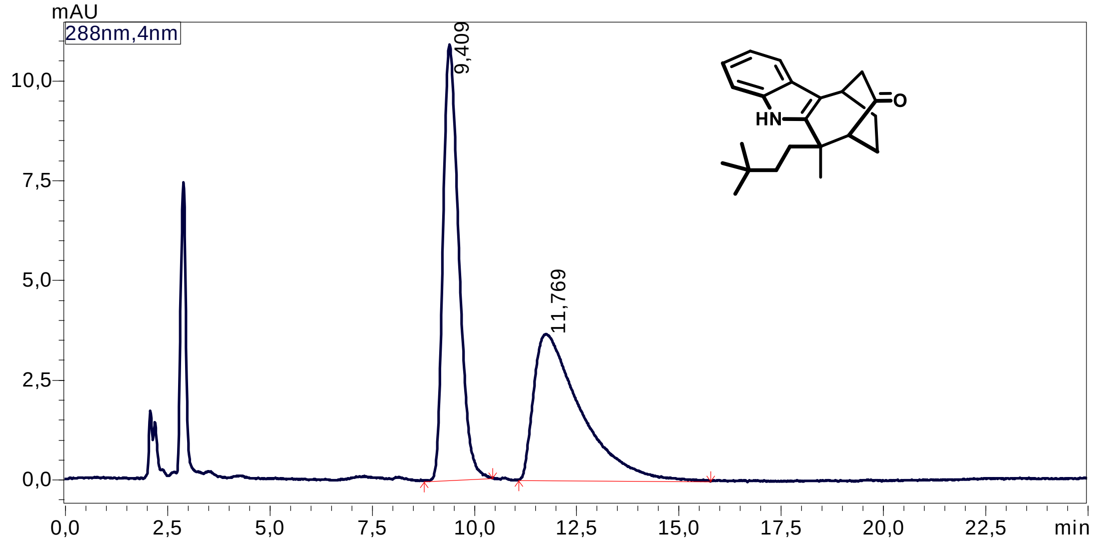


| Peak# | Ret. Time | Area% |
|---|---|---|
| 1 | 9,409 | 49,955 |
| 2 | 11,769 | 50,045 |
| Total | | 100,000 |

Datafile Name:OUJ-OC-079-P1-50-ee.lcd
Sample Name:OUJ-OC-079-P1-50-ee

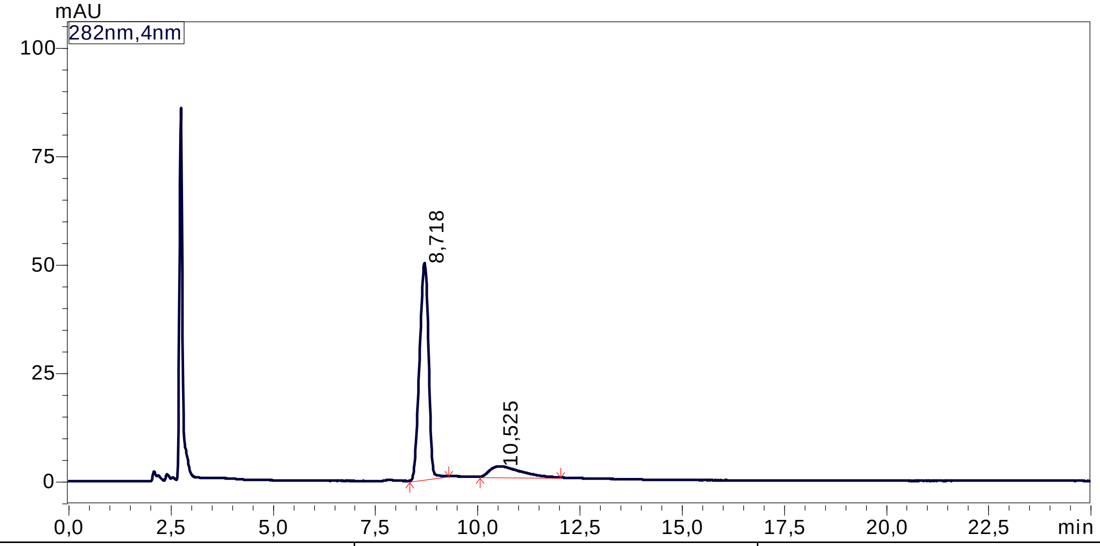


| Peak# | Ret. Time | Area% |
|---|---|---|
| 1 | 8,718 | 85,330 |
| 2 | 10,525 | 14,670 |
| Total | | 100,000 |

Datafile Name:OUJ-OC-102-TMS-P1-RAC001.lcd
Sample Name:OUJ-OC-102-TMS-P1-RAC001

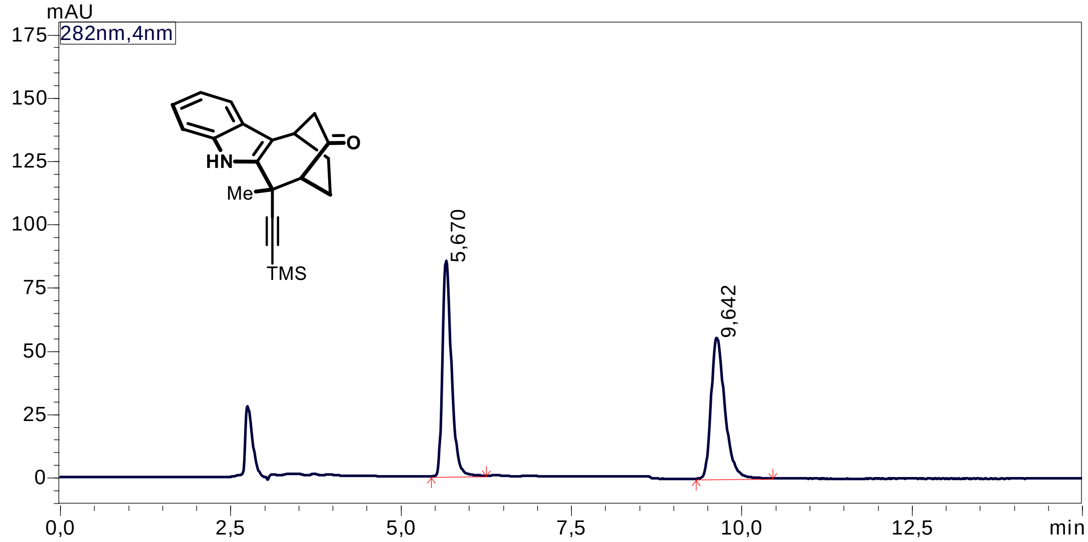


| Peak# | Ret. Time | Area% |
|---|---|---|
| 1 | 5,670 | 49,773 |
| 2 | 9,642 | 50,227 |
| Total | | 100,000 |

Datafile Name:OUJ-OC-102-TMS-P1-02-ee.lcd
Sample Name:OUJ-OC-102-TMS-P1-02-ee

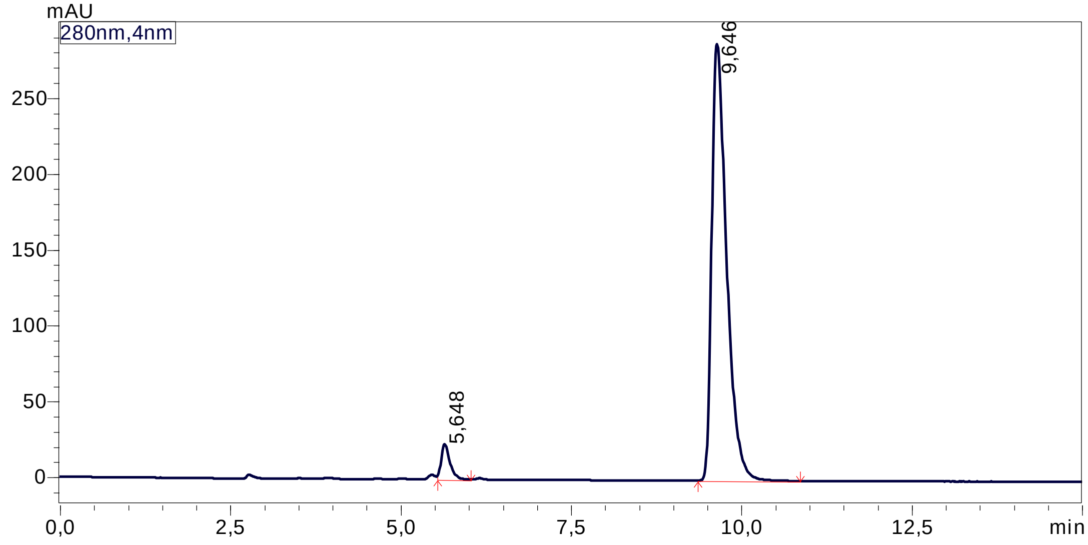


| Peak# | Ret. Time | Area% |
|---|---|---|
| 1 | 5,648 | 4,562 |
| 2 | 9,646 | 95,438 |
| Total | | 100,000 |

Datafile Name:OUJ-OC-102-TMS-P2-RAC-02.lcd
Sample Name:OUJ-OC-102-TMS-P2-RAC-02
Sample ID:eq012

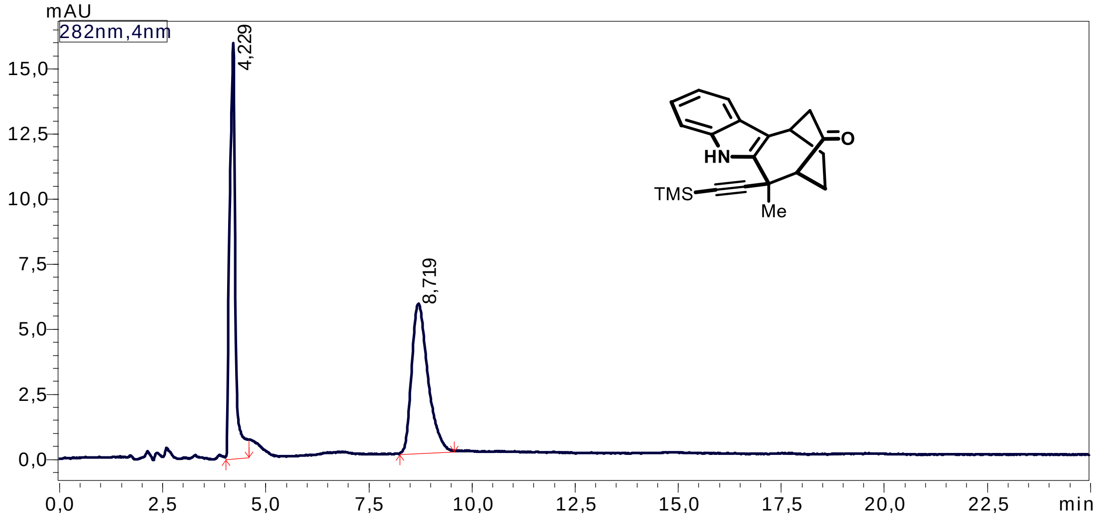


| Peak# | Ret. Time | Area% |
|---|---|---|
| 1 | 4,229 | 49,370 |
| 2 | 8,719 | 50,630 |
| Total | | 100,000 |

Datafile Name:OUJ-OB-102-TMS-P2-ee.lcd
Sample Name:OUJ-OB-102-TMS-P2-ee

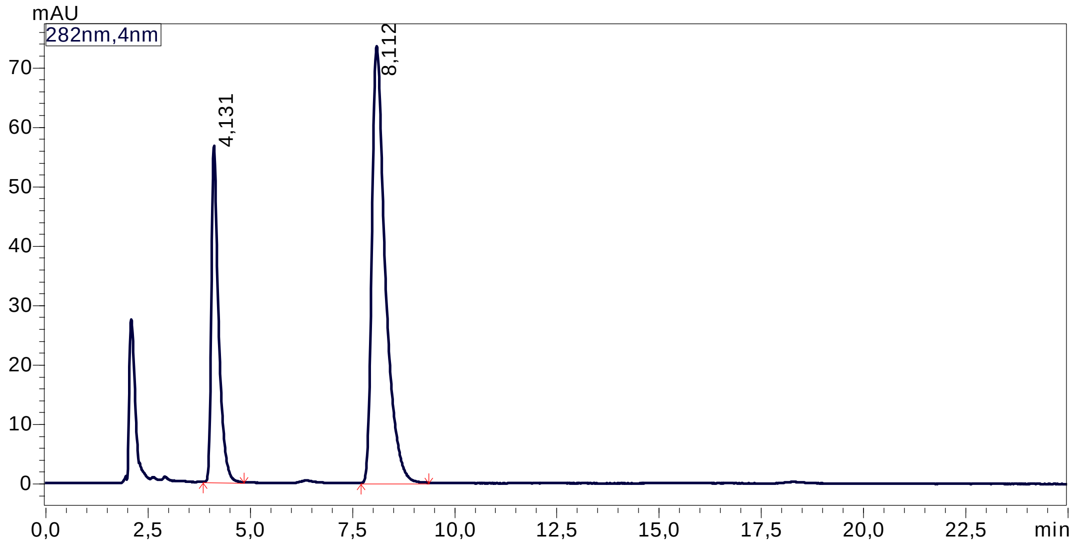


| Peak# | Ret. Time | Area% |
|---|---|---|
| 1 | 4,131 | 29,368 |
| 2 | 8,112 | 70,632 |
| Total | | 100,000 |

Datafile Name:OUJ-OC-115-P1-RAC002.lcd
Sample Name:OUJ-OC-115-P1-RAC002

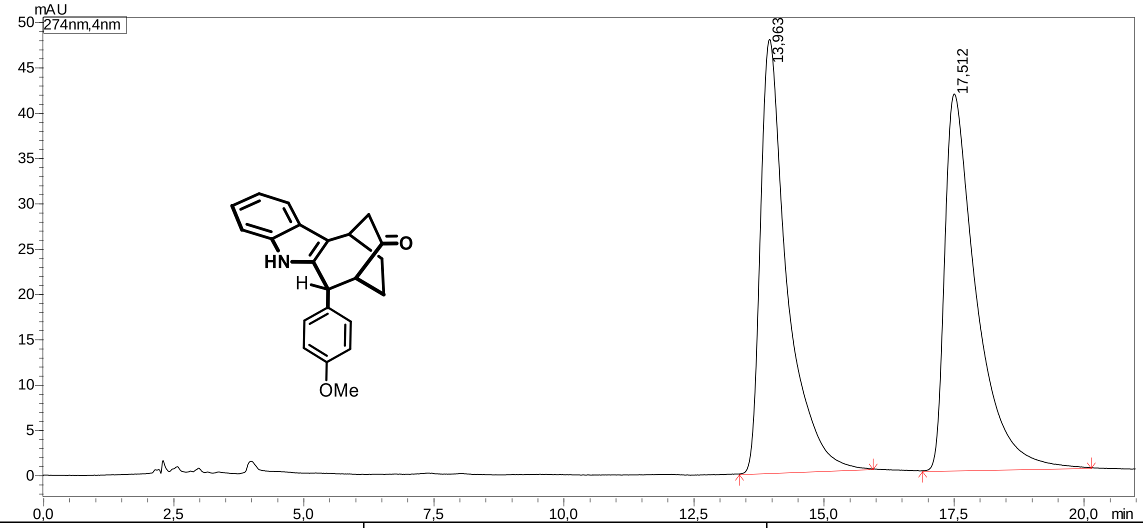


| Peak# | Ret. Time | Area% |
|---|---|---|
| 1 | 13,963 | 49,236 |
| 2 | 17,512 | 50,764 |
| Total | | 100,000 |

Datafile Name:snp364B.tmp
Sample Name:OUJ-OC-115-P1-ee-01

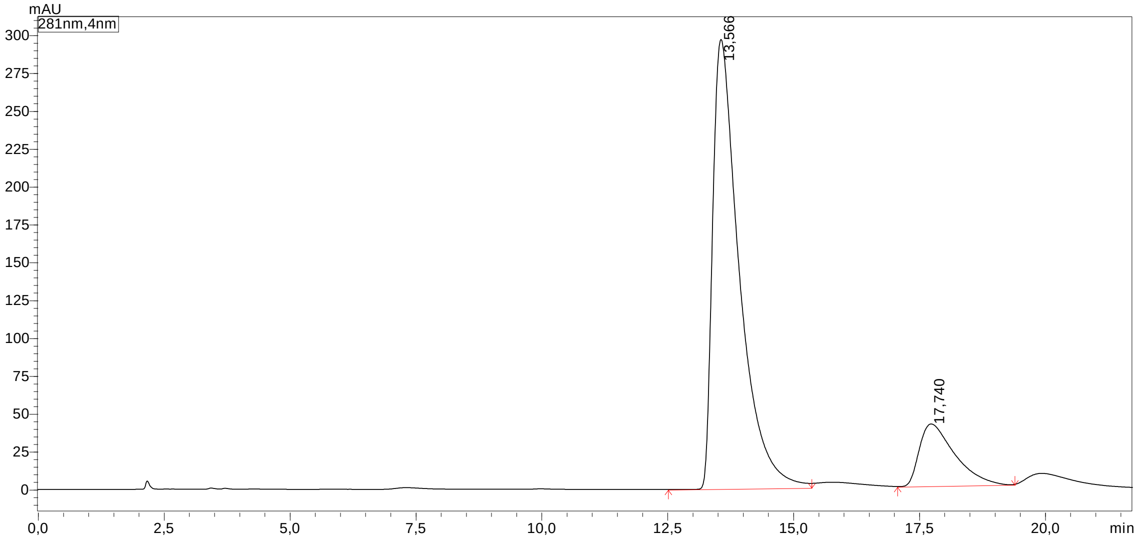


| Peak# | Ret. Time | Area% |
|---|---|---|
| 1 | 13,566 | 84,638 |
| 2 | 17,740 | 15,362 |
| Total | | 100,000 |

Datafile Name:OUJ-OC-115-P2-RAC001.lcd
Sample Name:OUJ-OC-115-P2-RAC001

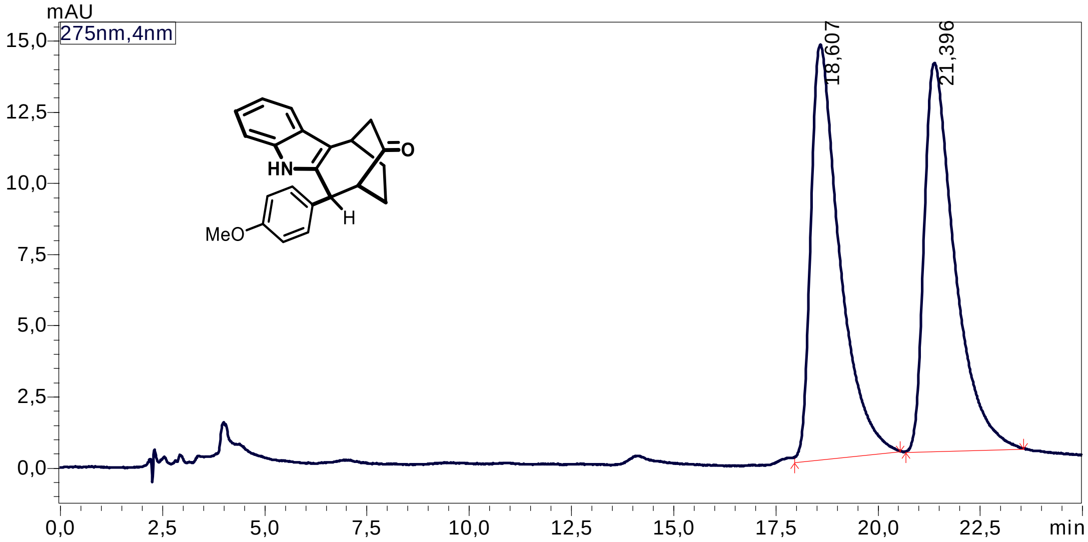


| Peak# | Ret. Time | Area% |
|---|---|---|
| 1 | 18,607 | 50,188 |
| 2 | 21,396 | 49,812 |
| Total | | 100,000 |

Datafile Name:OUJ-OC-115-P2.lcd
Sample Name:OUJ-OC-115-P2

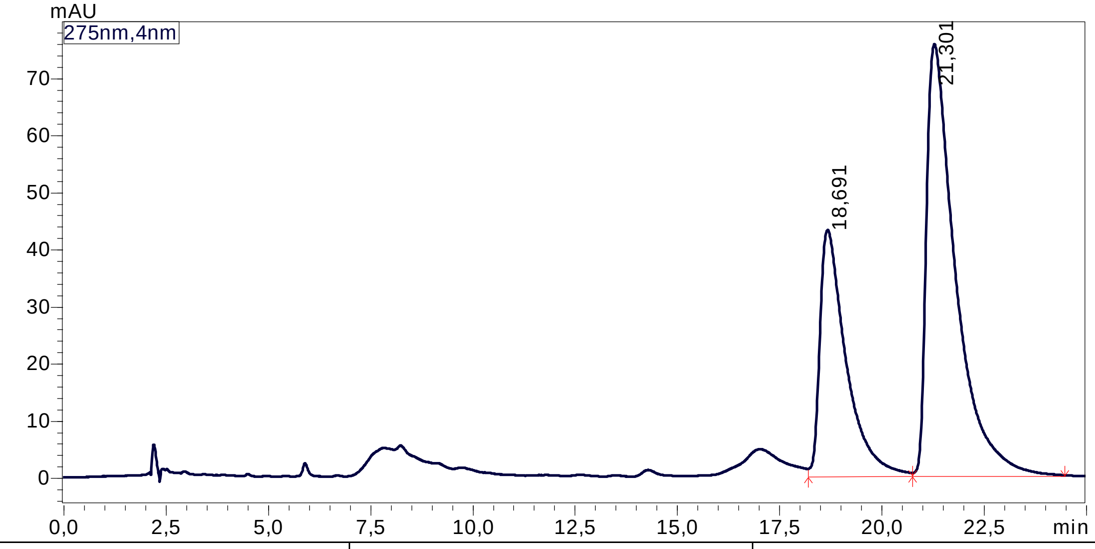


| Peak# | Ret. Time | Area% |
|---|---|---|
| 1 | 18,691 | 33,877 |
| 2 | 21,301 | 66,123 |
| Total | | 100,000 |

Datafile Name:OUJ-OC-Beckman-Rac-02.lcd
Sample Name:OUJ-Beckman-RAC-02

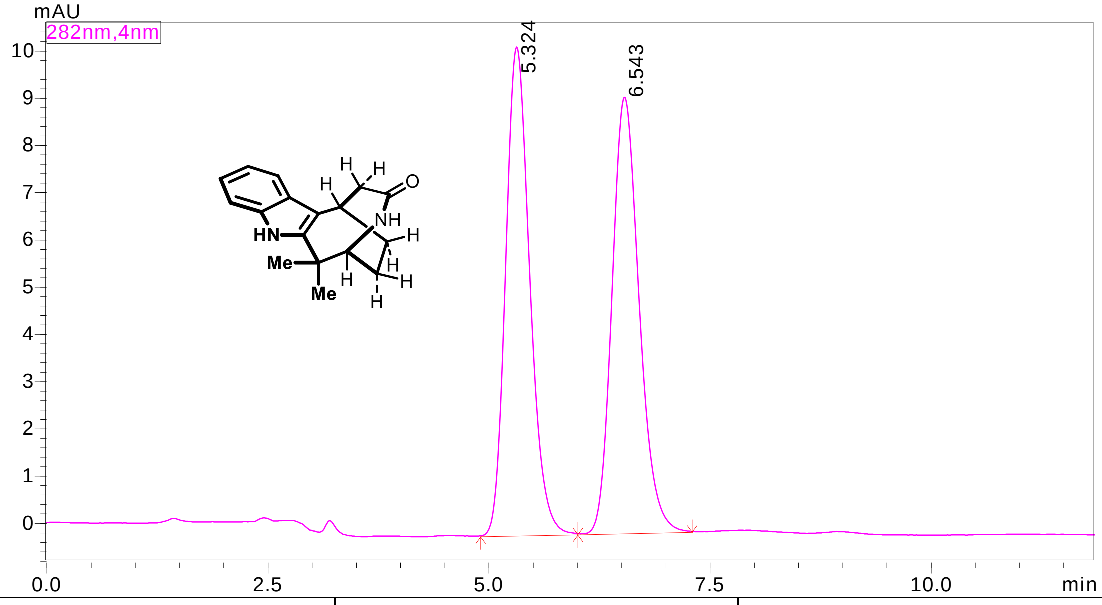


| Peak# | Ret. Time | Area% |
|---|---|---|
| 1 | 5.324 | 49.906 |
| 2 | 6.543 | 50.094 |
| Total | | 100.000 |

Datafile Name:OUJ-OC-147-02-Ee-Beckmann001.lcd
Sample Name:OUJ-OC-147-02-Ee-Beckmann001

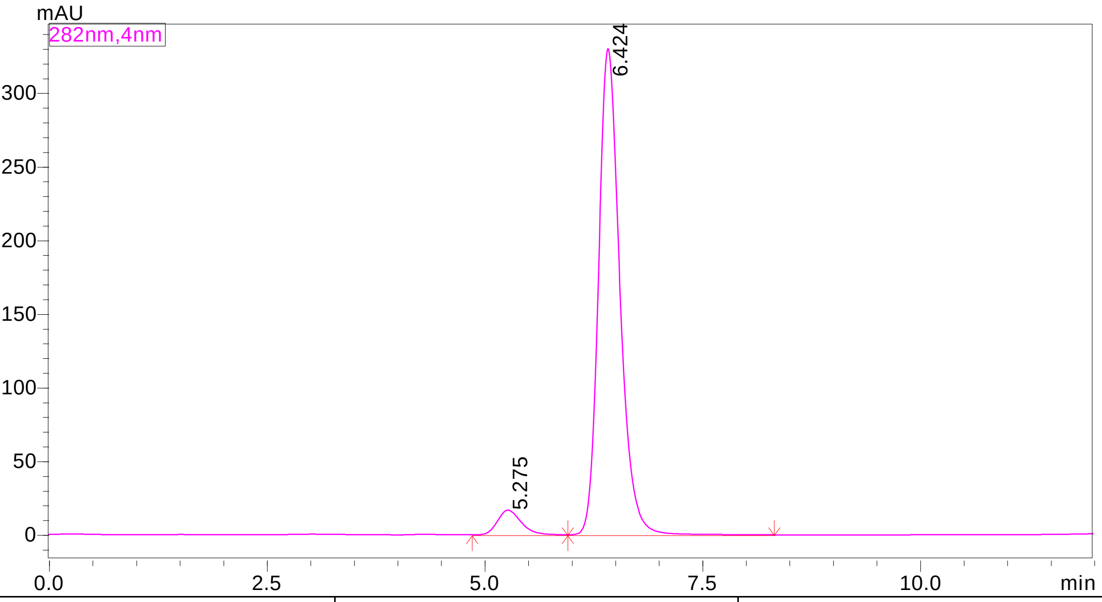


| Peak# | Ret. Time | Area% |
|---|---|---|
| 1 | 5.275 | 5.326 |
| 2 | 6.424 | 94.674 |
| Total | | 100.000 |

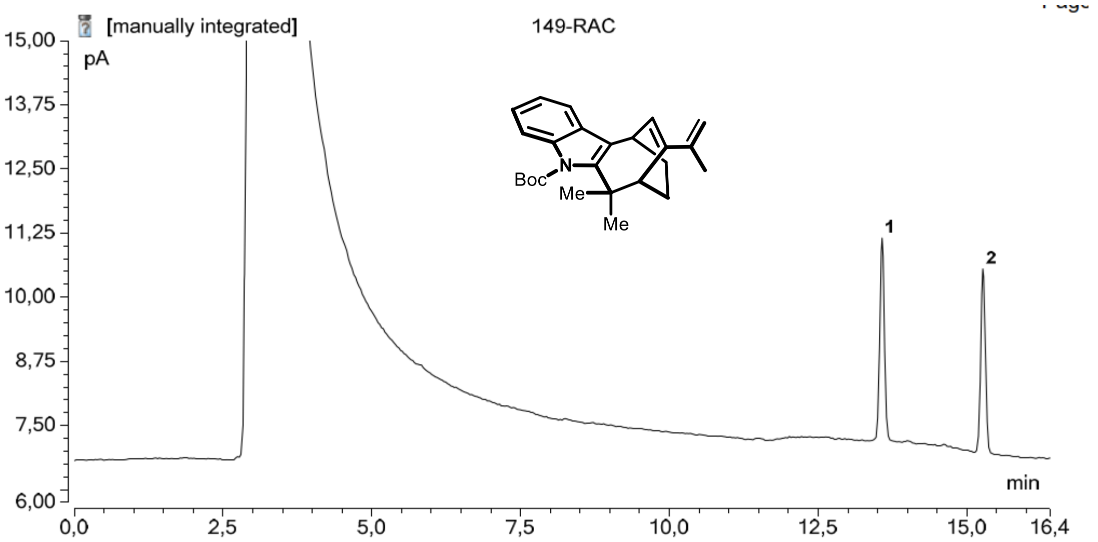


| | |
|---|---|
| Sample: | **149-RAC** |
| Sequenz: | **OUJ-OC-149-RAC** |
| Sequenz date: | **30.07.21** |
| Instrument: | **GC_Front** |
| Measured: | **30.07.21 14:39** |
| Processing M.: | **MPI** |
| Report-File: | **GC3 overview** |

| No. | Ret.Time min | area-% % | Peak Name |
|---|---|---|---|
| 1 | 13,58 | 50,72 | |
| 2 | 15,27 | 49,28 | |

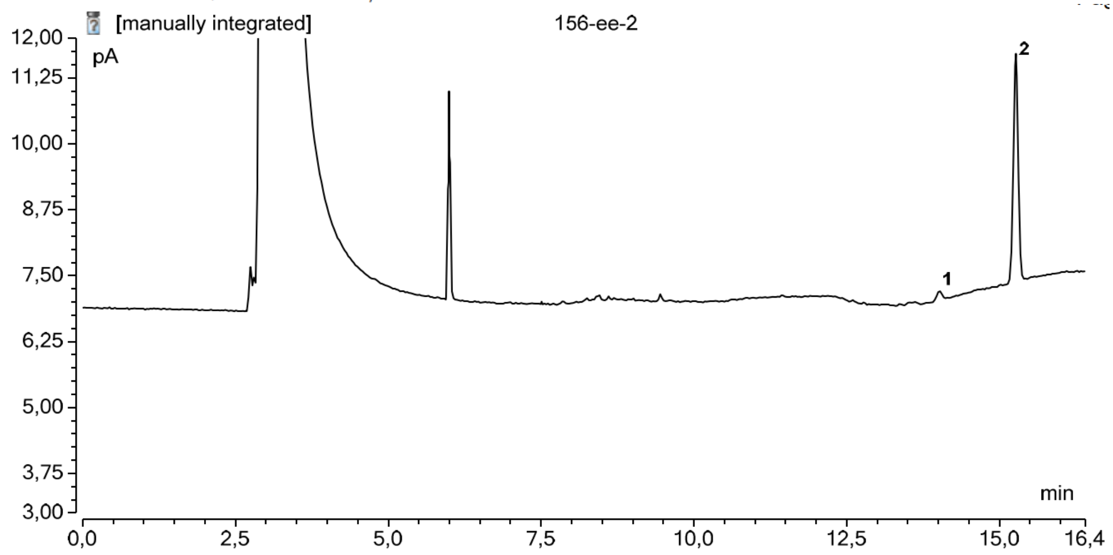


| | |
|---|---|
| Sample: | **156-ee-2** |
| Sequenz: | **OUJ-OC-149-RAC** |
| Sequenz date: | **30.07.21** |
| Instrument: | **GC_Front** |
| Measured: | **02.08.21 11:50** |
| Processing M.: | **MPI** |
| Report-File: | **GC3 overview** |

| No. | Ret.Time min | area-% % | Peak Name |
|---|---|---|---|
| 1 | 14,01 | 4,39 | |
| 2 | 15,27 | 95,61 | |

Datafile Name:OUJ-OC-108-RAC.lcd
Sample Name:OUJ-OC-108-RAC

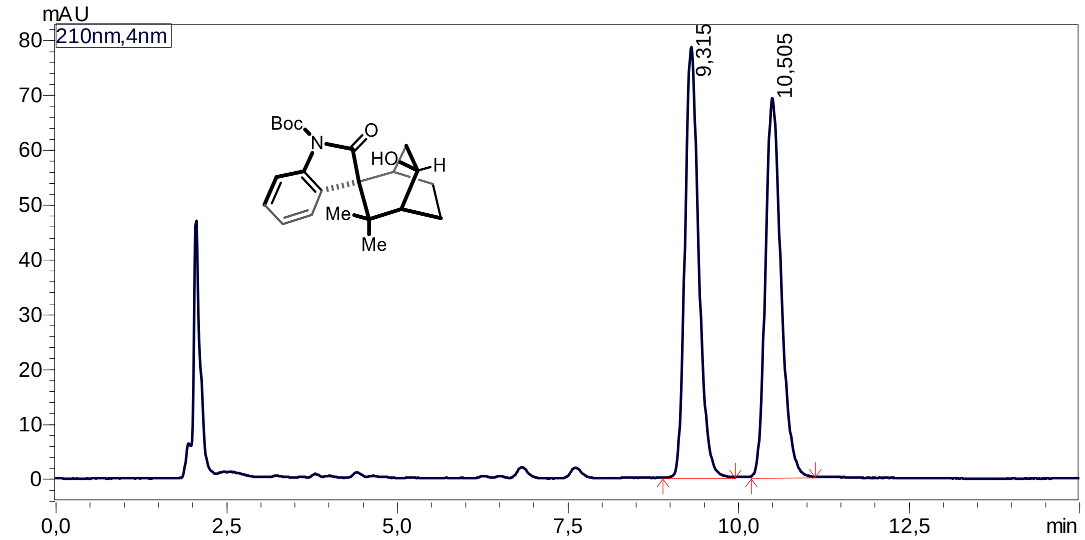


| Peak# | Ret. Time | Area% |
|---|---|---|
| 1 | 9,315 | 50,165 |
| 2 | 10,505 | 49,835 |
| Total | | 100,000 |

Datafile Name:OUJ-OC-155-P2-ee.lcd
Sample Name:OUJ-OC-155-P2-ee

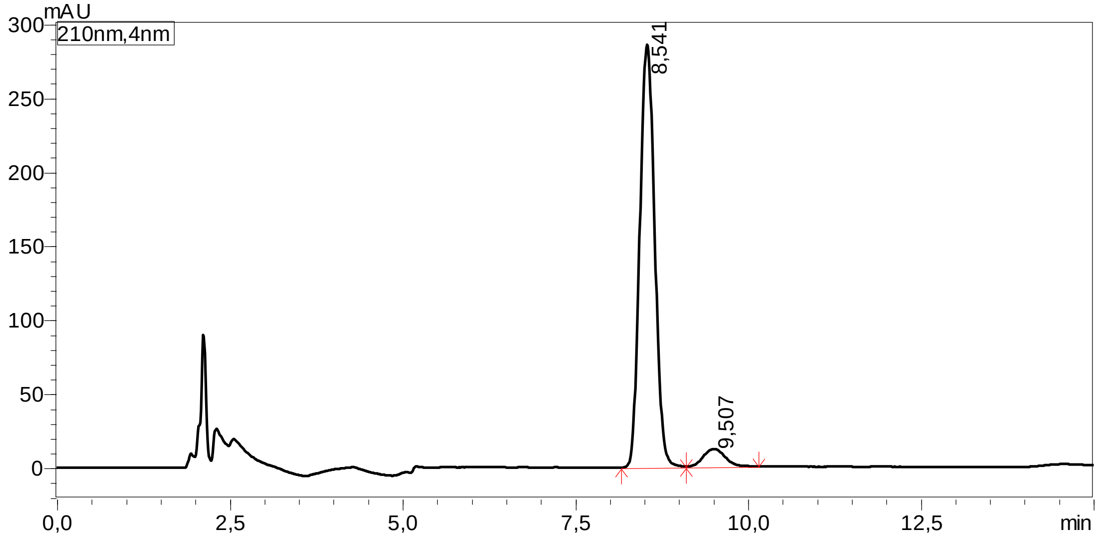


| Peak# | Ret. Time | Area% |
|---|---|---|
| 1 | 8,541 | 94,238 |
| 2 | 9,507 | 5,762 |
| Total | | 100,000 |

Datafile Name:OUJ-OC-141-allylation-RAC-08.lcd
Sample Name:OUJ-OC-141-allylation-RAC-08

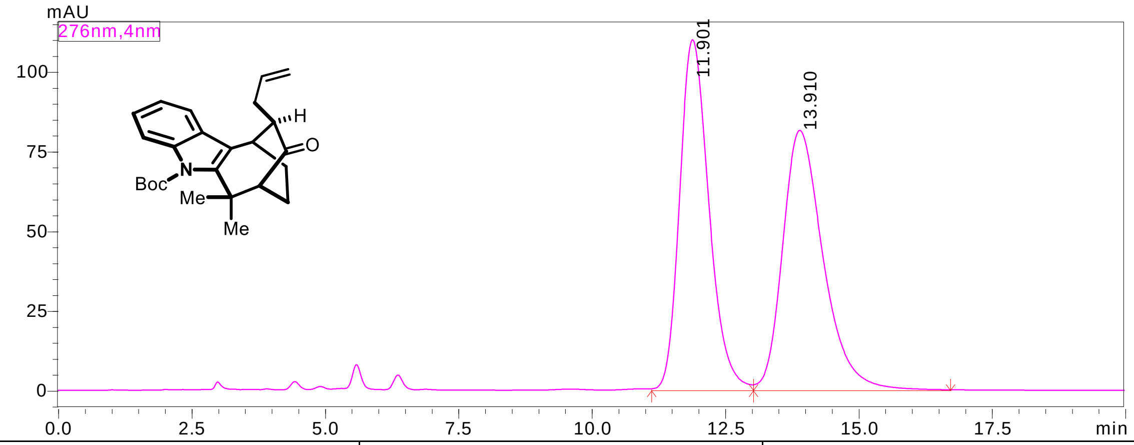


| Peak# | Ret. Time | Area% |
|---|---|---|
| 1 | 11.901 | 49.399 |
| 2 | 13.910 | 50.601 |
| Total | | 100.000 |

Datafile Name:OUJ-OC-148-allylation ee-01.lcd
Sample Name:OUJ-OC-148-allylation ee-01

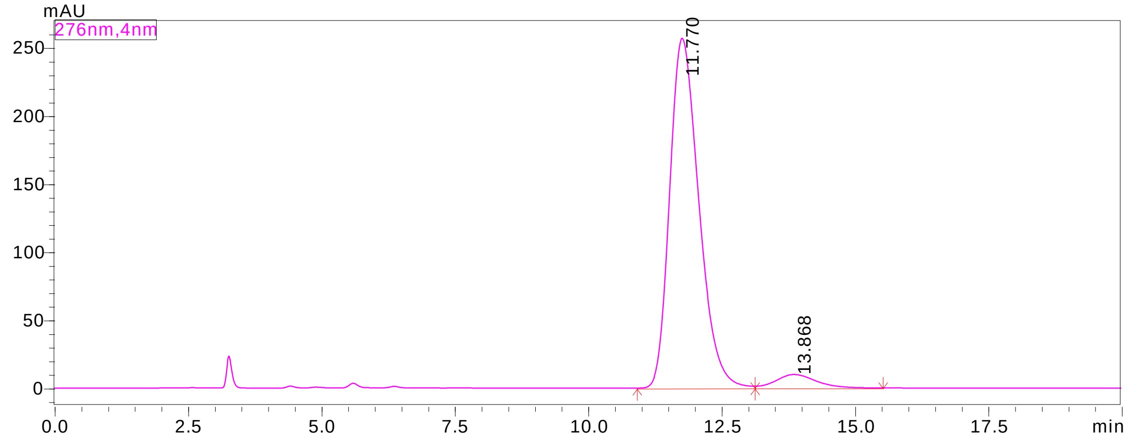


| Peak# | Ret. Time | Area% |
|---|---|---|
| 1 | 11.770 | 94.972 |
| 2 | 13.868 | 5.028 |
| Total | | 100.000 |
